# Enhancing the clinical diagnosis of the acute and subacute phases of autoimmune encephalitis and predicting the risk factors: the potential advantages of 18F-FDG PET/CT

**DOI:** 10.1186/s12880-023-01148-6

**Published:** 2023-11-20

**Authors:** Lili Liu, Zhehao Lyu, Huimin Li, Lin Bai, Yong Wan, Ping Li

**Affiliations:** 1 Department of PET/CT, The Second Affiliated Hospital of Harbin Medical University, No.246 Xuefu Road, Harbin, 150001 Heilongjiang People’s Republic of China; 2 Department of Nuclear Medicine, The First Affiliated Hospital of Harbin Medical University, Postal Street No.23, Harbin, 150001 Heilongjiang People’s Republic of China; 3 Department of Nuclear Medicine, Inner Mongolia Autonomous Region People’s Hospital, No.20 Zhaowuda Road, Hohhot, 010017 People’s Republic of China

**Keywords:** Autoimmune encephalitis, 18F-FDG PET/CT, Clinical diagnosis, Risk factors

## Abstract

**Background:**

2-deoxy-2-[18F]fluoro-D-glucose positron emission tomography (18F-FDG PET) could help evaluate metabolic abnormalities by semi-quantitative measurement to identify autoimmune encephalitis (AE). Few studies have been conducted to analyze the prognostic factors of AE. The study aimed to explore the values of diagnosis and treatment evaluation by 18F-FDG PET and preliminarily discussed the potential value in predicting the prognosis of AE patients.

**Methods:**

AE patients underwent 18F-FDG PET/CT and magnetic resonance imaging (MRI). There were two steps to analyse 18F-FDG PET imaging data. The first step was visual assessment. The second step was to analyse 18F-FDG PET parameters using Scenium software (Siemens Molecular Imaging Ltd). The mean standardized uptake value (SUV_mean_) and maximum standardized uptake value (SUV_max_) of brain relative regional metabolism (BRRM) were quantified in the case and control groups according to the anatomical automatic labeling (AAL) partition. The main statistical method was the Kruskal–Wallis test. Finally, the simple linear regression method was used to analyse the relationships between 18F-FDG PET parameters and the modified Rankin Scale (mRS) scores before and after treatment.

**Results:**

The results on 18F-FDG PET showed that visual assessment abnormalities were in the mesial temporal lobe (MTL) (70.8%), (mainly infringing on the hippocampus and amygdala), basal ganglia (62.5%), frontal lobes (37.5%), occipital lobes (29.2%), and parietal lobes (12.5%). The positive rate of abnormalities on 18F-FDG PET was more sensitive than that on MRI (95.5% vs 32.2%, *p* = 0.001). The number of lesions on PET was positively correlated with the mRS scores before and after treatment, and the correlation before treatment was more significant. Before treatment, the SUV_mean_ of the left occipital lobe was the most remarkable (SUV_mean_, R^2^ = 0.082, *p* > 0.05) factor associated with the mRS score, and the correlation was negative. With regard to prognosis, the SUV_max_ of the MTL was the most notable (R^2^ = 0.1471, *p* > 0.05) factor associated with the mRS score after treatment, and the correlation was positive.

**Conclusions:**

18F-FDG PET could be more sensitive and informative than MRI in the early phases of AE. The common pattern of AE was high MTL metabolism on 18F-FDG PET, which was associated with hypometabolism of the occipital lobe, and the number of lesions on PET before treatment may be significant factors in assessing disease severity. The SUV_max_ of MTL hypermetabolism may serve as a prognostic biomarker in AE.

**Supplementary Information:**

The online version contains supplementary material available at 10.1186/s12880-023-01148-6.

## Background

Autoimmune encephalitis (AE) is a non-infectious, immune-mediated inflammatory disease of the cerebrum parenchyma; this subacute presentation is highlighted in the Graus criteria and is a hallmark of the disorder, which is different from acute encephalitis developing as a rapidly progressive encephalopathy (usually in less than 6 weeks) [1–5]. Recent studies have found that the prevalence was 13.7 per 100,000 in Europe [6]. However, the mechanism underlying AE development is still unclear. It may be triggered by herpes simplex virus (HSV) encephalitis or specific immune-modulating therapies such as immune-checkpoint inhibitors (ICIs); the former is commonly associated with some common preceding factors such as viral infection, fever, or viral-like prodrome at the onset of this disorder [7], and the latter could result from an accelerated form of paraneoplastic encephalitis with advanced cancers [8]. The pace of disease progression may include acute and subacute presentations, and the median time from symptom onset to clinical assessment usually lasts several weeks [9, 10]. As noted, an individual may seem to have a precipitous deterioration concerning AE, but after further history-taking, it becomes apparent that there has been milder cognitive impairment over months or even years [11].

Imaging examinations of AE are based on magnetic resonance imaging (MRI), which can rule out stroke, tumours, and other infectious encephalitides. 2-deoxy-2-[18F] fluoro-D-glucose positron emission tomography (18F-FDG PET) is used as an auxiliary tool to be performed with more sensitivity and information related to brain abnormalities [12] when the results are negative or patients have contraindications for MRI. On the basis of background conditions, we conducted semi-quantitative analysis of 18F-FDG PET to verify the metabolic characteristics and to explore prognostic factors of AE.

## Methods

### Patients

**Fig. 1:**
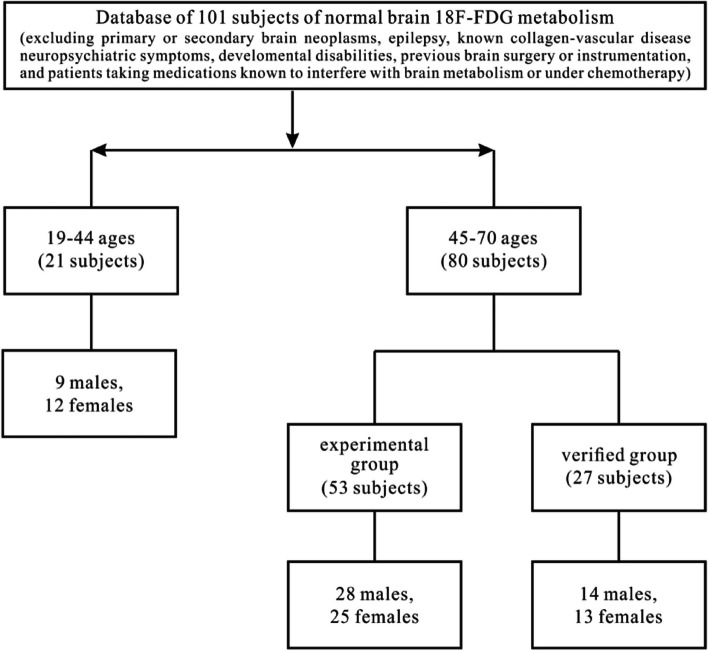
The establishment of normal control group and excluding criterion. The normal data of brain was included 101 subjects, which were divided into two groups, 19–44 years old group ( named control group-1) and 45–70 years old group, the latter was separated into experimental group and verified group (named control group-2)

A total of 32 patients with AE were retrospectively reviewed from the Second and First Affiliated Hospitals of Harbin Medical University between January 2017 and June 2022. All patients fulfilled the clinical diagnostic criteria and were positive for AE-related antibodies in the serum or cerebrospinal fluid (CSF). Thirty-one patients underwent MRI, and 24/31 patients underwent 18F-FDG PET (both anti-GAD65 and anti-LGI1 patients were re-examined after treatment in one year). Neuroimaging examinations (all MRIs and the remaining 22 patients’ PETs) were carried out in the acute and subacute stages after symptom onset. For the group analysis of 18F-FDG PET imaging, we identified 101 healthy controls without neurological anomalies, dividing them into two groups (Fig. 1) [13]. The 19 to 44-year-old group was named control group-1 (9 males, 12 females, the average age of males was 34.78 ± 8.00 years old [mean ± standard deviation, mean ± SD] and that of females was 36.92 ± 6.69 years old). The 45 to 70-year-old group was named control group-2, including the development group (28 males, 54.25 ± 5.5 years old, 25 females, 56.0 ± 7.2 years old) and verified groups (14 males, 51.43 ± 4.3 years old, 13 females, 55.8 ± 8.0 years old).

The demographic and clinical information, laboratory test results, and electroencephalograph (EEG) findings for individual patients and the comparison of the results are presented in (Table 1).

**Table 1:** Clinical characteristics of AE patients

*Item (total 32 subjects)*	Anti-NMDAR	Anti-GABABR	Anti-LGI1	Anti-HU	Anti-GAD65	Anti-PNMA2 + /Ma2/Ta	Anti-Amphiphysin	Anti-CASPR2	Anti-GFAP	*P *value
	*N* = 6	*N* = 5	*N* = 8	*N* = 7 (anti-HU *N* = 4) (anti-HUandSOX1 n = 2) (anti-HU and Ri n = 1)	*N* = 2	*N* = 1	*N* = 1	*N* = 1	*N* = 1	
***Age, years*** ***{media IQR}***	24.5 {13–36}	56.8 {41–66}	59.5 {40–67}	60.5 {51–77}	59.5 {55–64}	48	63	55	35	0.005*
***Female/male***	4/2	1/4	4/4	6/1	2/0	0/1	0/1	0/1	1/0	0.229
***Seizures***	6	5	8	2	0	1	1	1	0	0.001*
***Encephalalgia and dizziness***	6	3	3	5	2	1	0	1	1	0.201
***Decreased level of Consciousness***	2	2	3	1	1	1	0	0	0	0.906
***Cognitive impairment***	2	3	4	2	1	1	1	0	0	0.906
***Psychiatric symptoms***	2	0	1	3	0	0	0	0	1	0.550
***Metamorphopsia***	1	0	0	2	2	0	0	0	1	0.033*
***Speech disorder***	2	0	1	1	0	0	0	0	1	0.811
***Auditory hallucination***	1	0	0	0	0	0	0	1	0	0.317
***WBC ↑ (4.0–10.0 10***^***9***^***/L)***	3	0	5	1	2	1	0	0	0	0.052
***NUET% ↑ (50.0–70.0%)***	4	2	7	4	2	1	0	1	1	0.523
***LYMPH% ↓ (20.0–40.0%)***	3	2	6	3	2	1	0	1	1	0.598
***CPR ↑ (mg/L)***	4	5	7	6	2	1	0	1	1	0.811
***CSF-TPC ↑ (mg/L)***	2	3	4	0	1	1	1	1	1	0.021*
***Abnormal EEG-the sharp and slow waves in frontal or temporal***	6	5	3	–	1	0	0	1	0	0.007*
***Anti-ANA spectrum: nuclear particle type 1:100***	2	2	4	4	1	—	—	—	1	0.849
***Tumor markers*** ***{CEA ↑or SCC ↑ or CA125 ↑or NSE ↑*** ***or CYFRA21-1 ↑or CA724 ↑, CA199 ↑}***	0	2	3	5	0	—	1	1	0	0.370
***Abnormal brain MRI before treatment***	3	2	4	1	1	1	—	—	1	0.409
***mRS scores at the time of 18F-FDG PET/CT*** ***{media IQR}***	2 {1,4}	3.2 {1.4}	2.6 {1,4}	3.6 {3,4}	2.5 {2,3}	5	3	2	3	0.217
***Treated with steroids before 18F-FDG PET or MRI***	0	1	0	0	0	0	0	0	0	0.344
***Treated with AED before 18F-FDG PET or MR***	6	5	8	0	0	1	1	1	1	0.001*
***First-line treatment***	6	5	8	7	2	1	1	1	1	1
***Long immune treatment***	0	0	1	0	1	0	0	1	1	0.047*
***Follow-up time*** ***{months}***	14.3 {5–30}	16.7 {5–50}	15.2 {7–26}	18.9 {6–48}	15 {4–26}	42	13	16	—	0.001*
***mRS at the last follow up*** ***{media IQR}***	1.7 {1–3}	5 {2–6}	2.4 {0–6}	4.1 {1–6}	1 {1}	3	1	1	0	0.001*

*Abbreviations*: *AE* Autoimmune Encephalitis, *18F-FDG PET/CT* 2-deoxy-2-[18F]fluoro-D-glucose positron emission tomography-computed tomography, *MRI* magnetic resonance imaging, *mRS* modified Rankin Scale, *CRP* C-reactive protein, *CSF* cerebrospinal fluid, *TPC* total protein counts, *EEG* electroencephalogram, *WBC* White Blood Cell, *CEA* Carcinoembryonic antigen, the normal value < 5 ng/mL, *SCC* squamous cell carcinoma antigen, the normal value < 1.5 ng/mL, *CYFRA21-1* Cytokeratin-19-fragment, the normal value < 2.5 ng/mL, *NSE* neuron-specific enolase, the normal value < 17 ng/mL, *CA125* the normal value < 35U/ml, *CA199* the normal value < 37U/ml, *AED* anti-epileptic drugs; The first line of treatment included steroids, IVIg, and plasma exchange; Long immune treatment included Mycophenolate Mofetil and Azathioprine; The certain group: included four patients, anti-PNMA2 + /Ma2/ta, anti-Amphiphysin, anti-CASPR2, anti-GFAP, respectively one case, however, only anti-PNMA2 + /Ma2/ta, anti-Amphiphysin performed PET examination; *NMDAR* N-methyl-D-aspartate receptor, *LGI1* leucine-rich glioma inactivated-1, *CASPR2* contacting-associated protein-2, *GABABR* gamma-aminobutyric acid receptor, *AMPAR* α-amino-3-hydroxy-5-methyl-4-isoxazole propionic acid receptor, *GAD65* glutamic acid decarboxylase 65.*p < 0.05

### Antibody testing

All 32 patients underwent serum and CSF antibody testing, including tests for classic paraneoplastic antibodies (Hu, Yo, Ri, Ma2, CV2, Amphiphysin) and N-methyl-D-aspartate receptor (NMDAR), leucine-rich glioma inactivated-1 (LGI1), contacting-associated protein-2 (CASPR2), gamma-aminobutyric acid receptor (GABABR), α-amino-3-hydroxy-5-methyl-4-isoxazole propionic acid receptor (AMPAR), and glutamic acid decarboxylase 65 (GAD65) antibodies. Serum and CSF samples were analyzed using cell-based assays (Euroimmun, Lübeck, Germany), immunohistochemical analyses in the neuroimmunology laboratory of the Peking Union Medical College Hospital and Heilongjiang Kingmed for Clinical Laboratory.

### MRI

The MRI scanner was a 3.0 Tesla Discovery 750w MRI (GE Healthcare, USA). The standard MRI protocols included T1-weighted imaging (T1WI), T2-weighted imaging (T2WI), fluid-attenuated inversion recovery (FLAIR), and diffusion-weighted imaging (DWI). For T1WI [repetition time (TR) = 2203 ms, echo time (TE) = 25 ms, field of view (FOV) = 240 mm × 240 mm], T2WI (TR = 4356 ms, TE = 90 ms, FOV = 240 mm × 240 mm), FLAIR (TR = 6525 ms, TE = 83 ms, FOV = 240 mm × 240 mm), and DWI (TR = 3686 ms, TE = 77 ms, FOV = 240 mm × 240 mm), axial images were obtained, and the slice thickness was 5 mm. Two experienced radiologists independently evaluated the MRI results. If there was obvious discordance at the beginning of the evaluations, an informed consensus was achieved.

### 18F-FDG PET/CT

The PET/CT scanner was a Siemens Biograph 64 time-of-flight scanner. All patients were asked to fast for at least 6 h, and fasting blood glucose levels could not exceed 8 mmol/L. The injection dose was 0.12 mCi/kg, and the imaging agent was 18F-FDG. After injection, they were required to rest quietly and were isolated in a dedicated room to ensure minimal auditory and visual stimulation. The brain and whole-body imaging acquisition time was 40 min after injection. The brain acquisition time was 3 min/bed, and the speed of the whole body acquisition was 1.5 mm/s. Slice thickness was 3 mm and 1 mm.

### Analysis of 18F-FDG PET imaging

#### Visual assessment and Scenium software methods in case and control groups

Visual assessment was performed by two board-certified radiologists (10 years). 18F-FDG PET image analysis should be performed by drawing the region of interest (ROI) and then calculating the SUV and asymmetric index (AI) [14]:

$$AI=\frac{2\times (SUV\left(ipsilateral\right)-SUV(contralateral))}{SUV\left(ipsilateral\right)+SUV(contralateral)}$$

If the value of AI was larger than the threshold (e.g., 0.15) for three consecutive slices, the focus was determined to be a metabolic abnormality [15]. Encephalitis was suspected if there were manifestations of numerous focal cortical and/or subcortical abnormalities on MRI and hyper and/or hypometabolism on 18F-FDG PET. Scenium software provides quantification tools for the assessment of FDG-PET to calculate a statistical analysis of patients versus normal subjects and colour-coded statistical analysis, highlighting patterns of unusual radiopharmaceutical uptake. This software uses a deformable fusion algorithm to fuse the patient to the normal subject image to give an accurate match for cortical structures. Minimum, maximum and mean intensity values are computed for each region together with statistical information [16]. Regions of interest are licenced from CEA/Groupe d’ Imagerie Fonctionnelle [17]. The cerebrum was divided into 53 regions (excluding the cerebellum and brainstem) according to automated anatomical labelling (AAL) standards. Brain relative regional metabolism (BRRM) values of case groups, control group-1 and 2 were calculated. Excel forms were created, including data on the mean standardized uptake value (SUV_mean_), standard deviation of the SUV_mean_ (SUV_meanstd_), maximum standardized uptake value (SUV_max_), and standard deviation of the SUV_max_ (SUV_maxstd_) of each brain ROI [seen in Supplemental tables (1–12) ]. The mean value and 95% confidence interval were obtained. Simultaneously, the whole-body PET was used to screen for tumours.

A score of 1 was given for a focal anomaly in a lobe or increased uptake in the basal ganglia, and a score of 0 was given for the absence of a lobar anomaly or increased uptake in the basal ganglia through Scenium analysis.

### Follow-up and prognosis analysis

The modified Rankin Scale (mRS) scores were used to assess neurological disability at the onset and the last follow-up for this disorder. The mRS scores ≤ 2 indicated a good outcome, and the mRS scores of 3 to 6 indicated a poor outcome. The relationships among 18F-FDG PET parameters, severity degrees of the disease, and the outcome at the last follow-up after treatment were assessed.

### Statistical analysis

SPSS 25.0 software package for Windows (IBM Corp) and GraphPad Prism 9.4.1 (GraphPad Software, USA) were used for statistical analysis and charts. Categorical variables were compared and analyzed by Fisher’s exact test. Data are presented as the mean ± SD for continuous variables with a normal distribution, and non-normally distributed variables are expressed as the median (interquartile range [IQR]). Continuous variables were compared using the *t* test or nonparametric Mann‒Whitney U test. The Kruskal‒Wallis test was used to analyze multiple groups of constant variable comparisons. The relationships between continuous variables of SUV_S_ (SUV_mean_, SUV_max_) and the mRS scores (before and after treatment) were explored by simple linear regression. A two-tailed *p* value less than 0.05 (*p* < 0.05) was considered statistically significant.

### Standard protocol approvals, registrations, and patient consent

All patients signed informed consent forms, and the study was approved by the ethics committee of the Second Affiliated Hospital of Harbin Medical University (number KY2022-188).

## Results

### Clinical data

The average age of the anti-NMDAR group was close to 30 years old, the others were close to 60 years old (*p* = 0.005) (Table 1). Seizures (24/32,75%) were the most common symptom, excluding the anti-GAD65 group (*n* = 2) (*p* = 0.001). EEG, blood, and CSF analyses were performed before treatment, and all CSF bacterial and viral cultures were negative. There were statistically significant differences in EEG and CSF-TPC among the groups (*p* = 0.007, *p* = 0.021), because normal results accounted for a portion. Evidence of inflammation was verified in routine blood test results, including WBC↑ (12/32, 37.5%), NUET%↑ (22/32, 68.7%), LYMPH%↓ (19/32, 59.3%), and CPR↑ (27/32, 84.3%). Tumours were identified in 7 patients, including lung carcinoma in 6 anti-HU patients and one ovarian tumour in an anti-HU and Ri patient. The MRI was completed at a median of 8.5 days (*P*_25_ = 6, *P*_75_ = 30), and 18F-FDG PET was completed at a median of 30 days (*P*_25_ = 14, *P*_75_ = 60). There was a significant difference in the duration of symptoms to imaging between MRI and 18F-FDG PET/CT (*p* = 0.001).

**Fig. 2:**
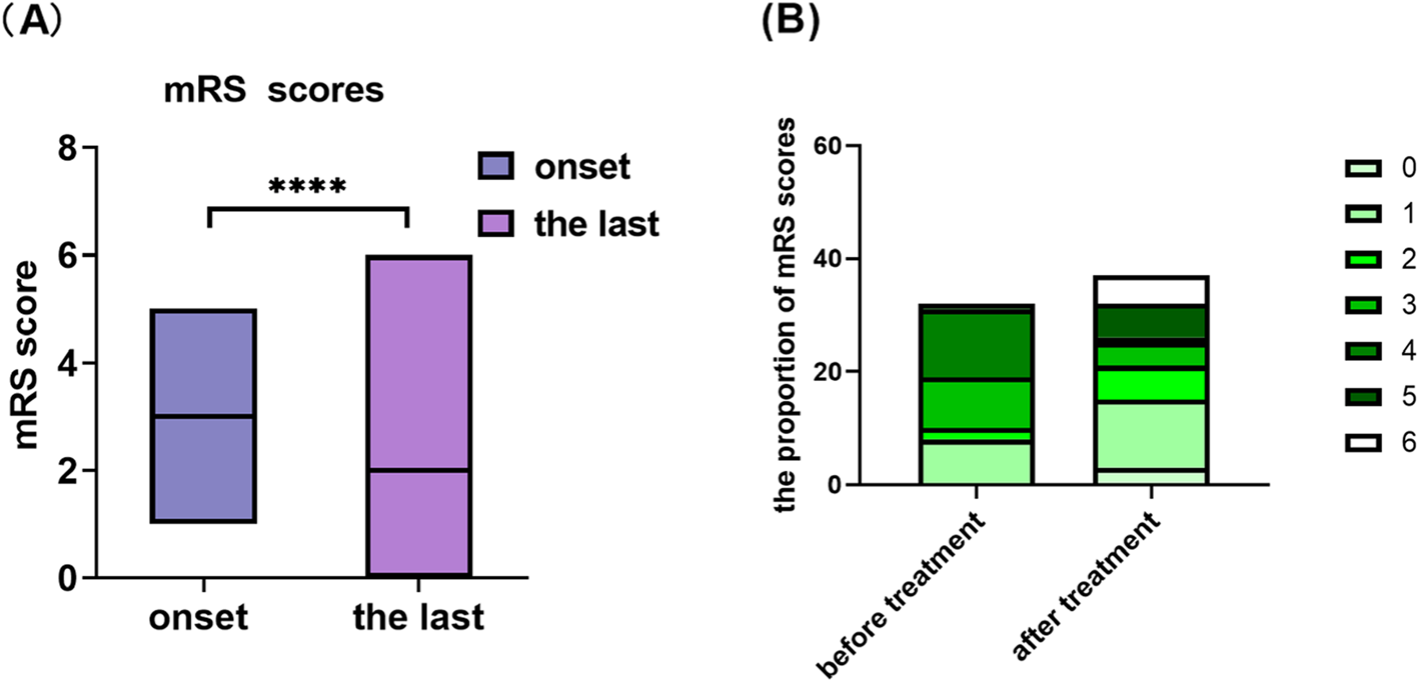
**A** The mRS scores of onset and the last follow up. The median of mRS score after treatment was lower than the onset.and it had significant statistical difference(*p* = 0.001). **B** The mRS score of 4 (37.5%,12/32) was the most proportion before treatment, however, the mRS score of 1 (37.5%,12/32) was the most proportion after treatment

The proportion of patients with an mRS score of 4 (37.5%, 12/32) was the highest before treatment. All patients accepted the first line of treatment, and long-term immune treatment was performed in four patients (anti-LGI1, anti-GAD65, anti-CASPR2, anti-GFAP) (*p* = 0.047). The prognosis was obviously improved, and the mRS score of 1 (37.5%, 12/32) was dramatically decreased after treatment (Fig. 2). Due to the death of tumours, the mRS scores after treatment at the last follow-up were higher in the anti-HU group (*p* = 0.001).

### Comparisons among MRI, visual and Scenium analysis of 18F-FDG PET findings in case groups

**Fig. 3:**
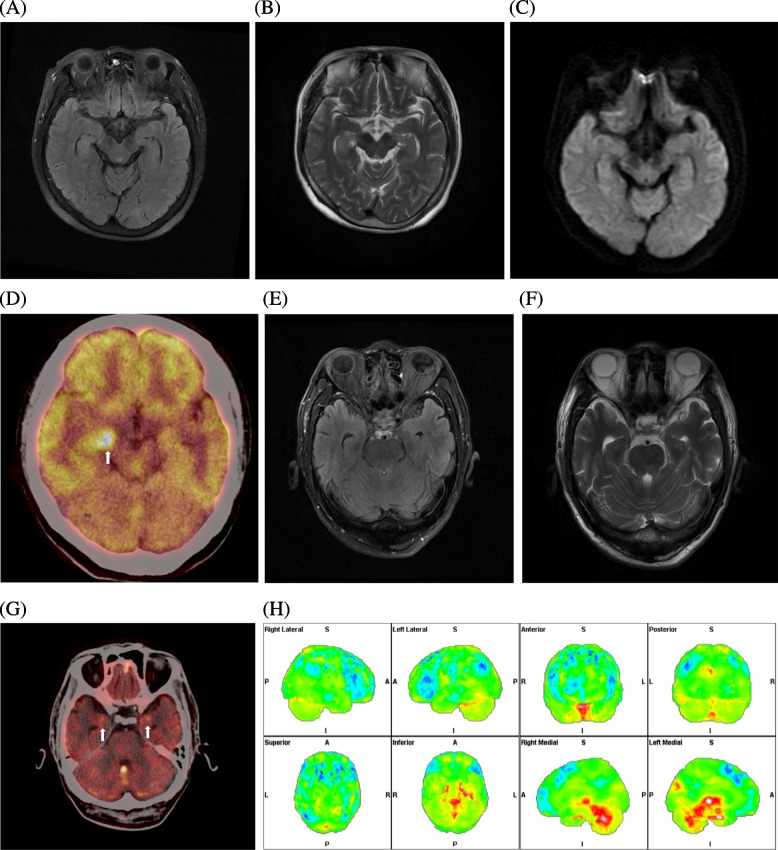
No.17, anti-GABABR patient, female, 56 years old, accompanying with seizures for 2 weeks, with negative MR and positive PET manifestations. **A**-**C** bilateral hippocampu in axial FLAIR, T2WI and DWI in MRI showed normal signal. **D** Hypermetabolism of right hippocampus in 18F-FDG PET (thick white arrow). No.18, anti-GABABR patient, male, 64 years old, with seizures for 2 weeks, with negative MR and positive PET manifestations. **E** and **F** bilateral hippocampus in axial FLAIR and T2WI in MRI showed normal signal. **G** Hypermetabolism of bilateral hippocampus in 18F-FDG PET (thick white arrow). **H** Through the Scenium software analysis, the bilateral MTL showed hypermetabolism in18F-FDG PET compared with the normal group, the Standard Deviation from SUV_mean_ was 11.8 (left) and 6.5 (right) respectively

We observed accordance analysis results on MRI and 18F-FDG PET and compared them (Table 2). The proportion of abnormal MRI findings was 32.2% (10/31), whereas that of 18F-FDG PET was 95.5% (21/22) (*P* = 0.001) (Fig. 3). Following the principle of symmetrical distribution of brain metabolism and calculating AI, visual assessment of 18F-FDG PET showed abnormalities of the temporal lobes (mainly infringing on the hippocampus and amygdala) in 17 patients, the basal ganglia in 15 patients, the frontal lobes in 9 patients, the occipital lobes in 7 patients, and the parietal lobes in 3 patients (Table 3A). Parietal lobes were more affected by anti-NMDAR than by anti-LGI1 (*p* = 0.036) (Table 3B), resembling ischaemic changes caused by anti-NMDAR (No. 1, No. 2) encephalitis.

**Table 2:** The comparison between 18F-FDG PET and MRI diagnosis

Patient number	Antibody type	Sex	Age (years)	18F-FDG PET diagnoses	18F-FDG PET delay (days)	MR diagnoses	MR delay (days)	Accordance between 18F-FDG PET and MR
**1**	NMDAR	Female	36	Encephalitis	30	Encephalitis	20	Yes
**2**	NMDAR	Male	34	Encephalitis	20	Encephalitis	20	Yes
**3**	NMDAR	Female	13	—	—	Normal	8	—
**4**	NMDAR	Female	14	—	—	Demyelination	3	—
**5**	NMDAR	Male	25	—	—	—	—	—
**6**	NMDAR	Female	25	—	—	Normal	60	—
**7**	LGI1	Female	64	Encephalitis	30	Encephalitis	4	Yes
**8**	LGI1	Female	56	—	—	Normal	9	—
**9**	LGI1	Male	67	—	—	Normal	60	—
**10**	LGI1	Female	61	—	—	Normal	90	—
**11**	LGI1	Male	54	Encephalitis	21	Normal	11	No
**12**	LGI1	Female	40	Encephalitis	6	Normal	2	No
**13**	LGI1	Male	54	Encephalitis	30	Encephalitis	1	Yes
**14**	LGI1	Male	55	Encephalitis	30	Normal	30	No
**15**	GABABR	Male	41	—	—	Encephalitis	14	—
**16**	GABABR	Male	66	Encephalitis	30	Encephalitis	1	Yes
**17**	GABABR	Female	56	Encephalitis	24	Normal	21	No
**18**	GABABR	Male	64	Encephalitis	16	Normal	5	No
**19**	GABABR	Male	57	Encephalitis	14	Normal	1	No
**20**	HU	Female	55	Encephalitis	14	Normal	1	No
**21**	HU	Female	67	Normal	183	Normal	150	No
**22**	HU	Male	77	Encephalitis	30	Normal	30	No
**23**	HU	Female	60	Encephalitis	90	Normal	90	No
**24**	SOX1 and HU	Female	51	Encephalitis	60	Encephalitis	30	No
**25**	SOX1 and HU	Female	63	Encephalitis	14	Normal	7	No
**26**	HU and RI	Female	66	Encephalitis	183	Normal	7	No
**27**	GAD65-Ab +	Female	64	Encephalitis	60	Encephalitis	50	Yes
**28**	GAD65-Ab +	Female	55	Encephalitis	300	Normal	300	No
**29**	PNMA2 + /Ma2/Ta	Female	48	Encephalitis	14	Encephalitis	2	Yes
**30**	Amphiphysin	Male	63	Encephalitis	7	Normal	1	No
**31**	CASPR2	Male	55	—	—	Normal	14	—
**32**	GFAP	Female	35	—	—	Encephalitis	7	—

*Abbreviations*: *18F-FDG PET* 2-deoxy-2-[18F]fluoro-D-glucose positron emission tomography, *MRI* magnetic resonance imaging, *NMDAR* N-methyl-D-aspartate receptor, *LGI1* leucine-rich glioma inactivated-1, *CASPR2* contacting-associated protein-2, *GABABR* gamma-aminobutyric acid receptor, *AMPAR* α-amino-3-hydroxy-5-methyl-4-isoxazole propionic acid receptor, *GAD65* glutamic acid decarboxylase

**Table 3:** Visual assessment on 18F-FDG PET: affected lobes with regions of hyper and/or hypometabolism and increased uptake in basal ganglia

*Affected lobes*	Anti-NMDAR	Anti-GABABR	Anti-LGI1	Anti-HU	Anti-GAD65	Anti-PNMA2 + /Ma2/ta	Anti-Amphiphysin
***A: Patient details***							
***Total cases***	*N* = 2	*N* = 4	*N* = 6	*N* = 7	*N* = 3	*N* = 1	*N* = 1
***Frontal anomalies***	2	1	1	5	0	0	0
***Temporal anomalies***	2	4	3	4	2	1	1
***Parietal anomalies***	2	0	0	0	0	1	0
***Occipital anomalies***	1	0	1	2	2	0	1
***Increased basal ganglia***	0	1	5	5	2	1	1
***B: Statistical analysis***							
***Frontal lobes***	Anti-NMDAR	Anti-GABABR	Anti-LGI1	Anti-HU	Anti-GAD65		
***Anti-GABABR***	*p* = 0.40						
***Anti-LGI1***	*p* = 0.464	*p* = 1.00					
***Anti-HU***	*p* = 1.00	*p* = 0.242	*p* = 0.103				
***Anti-GAD65***	*p* = 0.40	*p* = 1.00	*p* = 1.00	*p* = 0.167			
***Anti-the certain group***	*p* = 1.00	*p* = 1.00	*p* = 1.00	*p* = 0.167	*p* = 1.00		
***Temporal lobes***	Anti-NMDAR	Anti-GABABR	Anti-LGI1	Anti-HU	Anti-GAD65		
***Anti-GABABR***	*p* = 1.00						
***Anti-LGI1***	*p* = 0.464	*p* = 0.20					
***Anti-HU***	*p* = 0.50	*p* = 0.234	*p* = 1.00				
***Anti-GAD65***	*p* = 1.00	*p* = 0.429	*p* = 1.00	*p* = 1.00			
***Anti-the certain group***	*p* = 1.00	*p* = 1.00	*p* = 0.50	*p* = 0.167	*p* = 1.00		
***Parietal lobes***	Anti-NMDAR	Anti-GABABR	Anti-LGI1	Anti-HU	Anti-GAD65		
***Anti-GABABR***	*p* = 0.067						
***Anti-LGI1***	*p* = 0.036*	*p* = 1.00					
***Anti-HU***	*p* = 0.083	*p* = 1.00	*p* = 1.00				
***Anti-GAD65***	*p* = 0.1	*p* = 1.00	*p* = 1.00	*p* = 1.00			
***Anti-the certain group***	*p* = 0.33	*p* = 1.00	*p* = 0.50	*p* = 1.00	*p* = 1.00		
***Occipital lobes***	Anti-NMDAR	Anti-GABABR	Anti-LGI1	Anti-HU	Anti-GAD65		
***Anti-GABABR***	*p* = 0.33						
***Anti-LGI1***	*p* = 0.464	*p* = 0.048*					
***Anti-HU***	*p* = 1.00	*p* = 0.061	*p* = 1.00				
***Anti-GAD65***	*p* = 1.00	*p* = 0.429	*p* = 0.226	*p* = 0.50			
***Anti-the certain group***	*p* = 1.00	*p* = 0.333	*p* = 0.464	*p* = 1.00	*p* = 1.00		
***Basial ganglia***	Anti-NMDAR	Anti-GABABR	Anti-LGI1	Anti-HU	Anti-GAD65		
***Anti-GABABR***	*p* = 1.00						
***Anti-LGI1***	*p* = 0.107	*p* = 0.190					
***Anti-HU***	*p* = 0.167	*p* = 0.242	*p* = 1.00				
***Anti-GAD65***	*p* = 0.40	*p* = 0.486	*p* = 1.00	*p* = 1.00			
***Anti-the certain group***	*p* = 0.333	*p* = 0.40	*p* = 1.00	*p* = 1.00	*p* = 1.00		

*Abbreviations*: *18F-FDG PET* 2-deoxy-2-[18F]fluoro-D-glucose positron emission tomography, *NMDAR* N-methyl-D-aspartate receptor, *LGI1* leucine-rich glioma inactivated-1, *CASPR2* contacting-associated protein-2, *GABABR* gamma-aminobutyric acid receptor, *AMPAR* α-amino-3-hydroxy-5-methyl-4-isoxazole propionic acid receptor, *GAD65* glutamic acid decarboxylase

* *p*=0.036*: the metabolism results through visual assessment of parietal lobes was statistically significant between anti-NMDAR group and anti-LGI1 group. *p*=0.048*: The metabolism results through visual assessment of occipital lobes was statistically significant between anti-GABABR group and anti-LGI1group

The MRI and 18F-FDG PET/CT (through Scenium analysis) manifestations in the case groups are summarized in (Table 4). The 18F-FDG PET result was negative in one patient (No. 21), and a single abnormal uptake region was observed in 5 patients, three involving the hippocampus (No. 13, No. 14, No. 27), one each involving the basal ganglia (No. 23) and the cingulate gyrus (No. 26). Multiple abnormal uptakes of cortical regions were observed in 16 patients.

**Table 4:** The detailed MRI and 18F-FDG PET/CT (through Scenium analysis) results of AE patients

**Patient number**	**Antibody type**	**Gender**	**Age**	**MRI results** **T2WI/Flair/perfusion hyper- intesities**	18F-FDG **PET results of Scenium analysis**	
					**Hyper metabolism**	**Hypo metabolism**
**1**	NMDAR	Female	36	Right cerebrum, brainstem, right thalamus and basal ganglia	Right supplementary motor area and middle cingulate	Right frontal, temporal, parietal, occipital lobes and left parietal lobe
**2**	NMDAR	Male	34	Right frontal, parietal, temporal lobe, especially temporal lobe	Right frontal, temporal, insula lobe and anterior and middle cingulate left hippocampus and brainstem	Bilateral parietal lobe and occipital lobe, left frontal lobe
**3**	NMDAR	Female	13	Normal	—	
**4**	NMDAR	Female	14	Demyelination	—	
**5**	NMDAR	Male	25	—	—	
**6**	NMDAR	Female	25	Normal	—	
**7**	LGI1	Female	64	Left temporal and occipital lobe, left hippocampus and brainstem	Bilateral basal ganglia, amygdala, hippocampus, para- hippocampus and anterior cingulate	
**8**	LGI1	Female	56	Normal	—	
**9**	LGI1	Male	67	Normal	—	
**10**	LGI1	Female	61	Normal	—	
**11**	LGI1	Male	54	Normal	Bilateral basal ganglia, amygdala, hippocampus, para- hippocampus and anterior cingulate	
**12**	LGI1	Female	40	Normal	Bilateral basal ganglia, amygdala, hippocampus, para- hippocampus and anterior cingulate	
**13**	LGI1	Male	54	Bilateral hippocampus, insula and temporal lobe	Bilateral hippocampus and insula	
**14**	LGI1	Male	55	Normal	Bilateral hippocampus and insula	
**15**	GABABR	Male	41	Left hippocampus, basal ganglia and temporal lobe	—	
**16**	GABABR	Male	66	Bilateral hippocampus, insula and temporal lobe	Bilateral basal ganglia, amygdala, hippocampus, para- hippocampus	
**17**	GABABR	Female	56	Normal	Bilateral basal ganglia, amygdala, hippocampus, para-hippocampus, anterior and middle cingulate, right insula and inferior frontal gyrus	
**18**	GABABR	Male	64	Normal	Bilateral hippocampus and thalamus	
**19**	GABABR	Male	57	Normal	Bilateral basal ganglia, amygdala, hippocampus, para-hippocampus	
**20**	HU	Female	55	Normal	Bilateral basal ganglia, amygdala, left hippocampus, para- hippocampus, bilateral anterior cingulate, right central region and right gyrus rectus and cuneus gyrus	
**21**	HU	Female	67	Normal	Normal	
**22**	HU	Male	77	Normal	Bilateral basal ganglia, amygdala, left hippocampus, para- hippocampus, right orbital gyrus, bilateral central region and anterior central gyrus and brainstem	
**23**	HU	Female	60	Normal	Bilateral basal ganglia	
**24**	SOX1 and HU	Female	51	Bilateral hippocampus, left insula and temporal lobe	Bilateral basal ganglia, amygdala, hippocampus, para- hippocampus, left paracentral lobe and brainstem	
**25**	SOX1 and HU	Female	63	Normal	Bilateral amygdala, hippocampus, para-hippocampus, left paracentral lobe, left lingual gyrus and bilateral occipital lobe	
**26**	HU and RI	Female	66	Normal	Bilateral cingulate gyrus	
**27**	GAD65-Ab +	Female	64	Bilateral hippocampus, insula and temporal lobe	Bilateral amygdala, hippocampus	
**28**	GAD65-Ab +	Female	55	Normal	Bilateral parietal and occipital lobe, bilateral paracentral lobe, left hippocampus, para-hippocampus	
**29**	PNMA2 + /Ma2/Ta	Female	48	Left temporal and parietal lobe, left insula and hippocampus	Bilateral amygdala, hippocampus, para-hippocampus, insula and anterior central gyrus, left basal ganglia, cingulate gyrus, olfactory cortex	Left temporal and parietal lobe
**30**	Amphiphysin	Male	63	Normal	Bilateral occipital lobe and thalamus, anterior central gyrus	
**31**	CASPR2	Male	55	Normal	—	—
**32**	GFAP	Female	35	Right temporal and occipital lobe, right cerebellum	—	—

*Abbreviations*: *AE* Autoimmune Encephalitis, *18F-FDG PET* 2-deoxy-2-[18F]fluoro-D-glucose positron emission tomography, *MRI* magnetic resonance imaging, *NMDAR* N-methyl-D-aspartate receptor, *LGI1* leucine-rich glioma inactivated-1, *CASPR2* contacting-associated protein-2, *GABABR* gamma-aminobutyric acid receptor, *AMPAR* α-amino-3-hydroxy-5-methyl-4-isoxazole propionic acid receptor, *GAD65* glutamic acid decarboxylase 65

### Comparison of BRRM SUVs between case and control groups

**Fig. 4:**
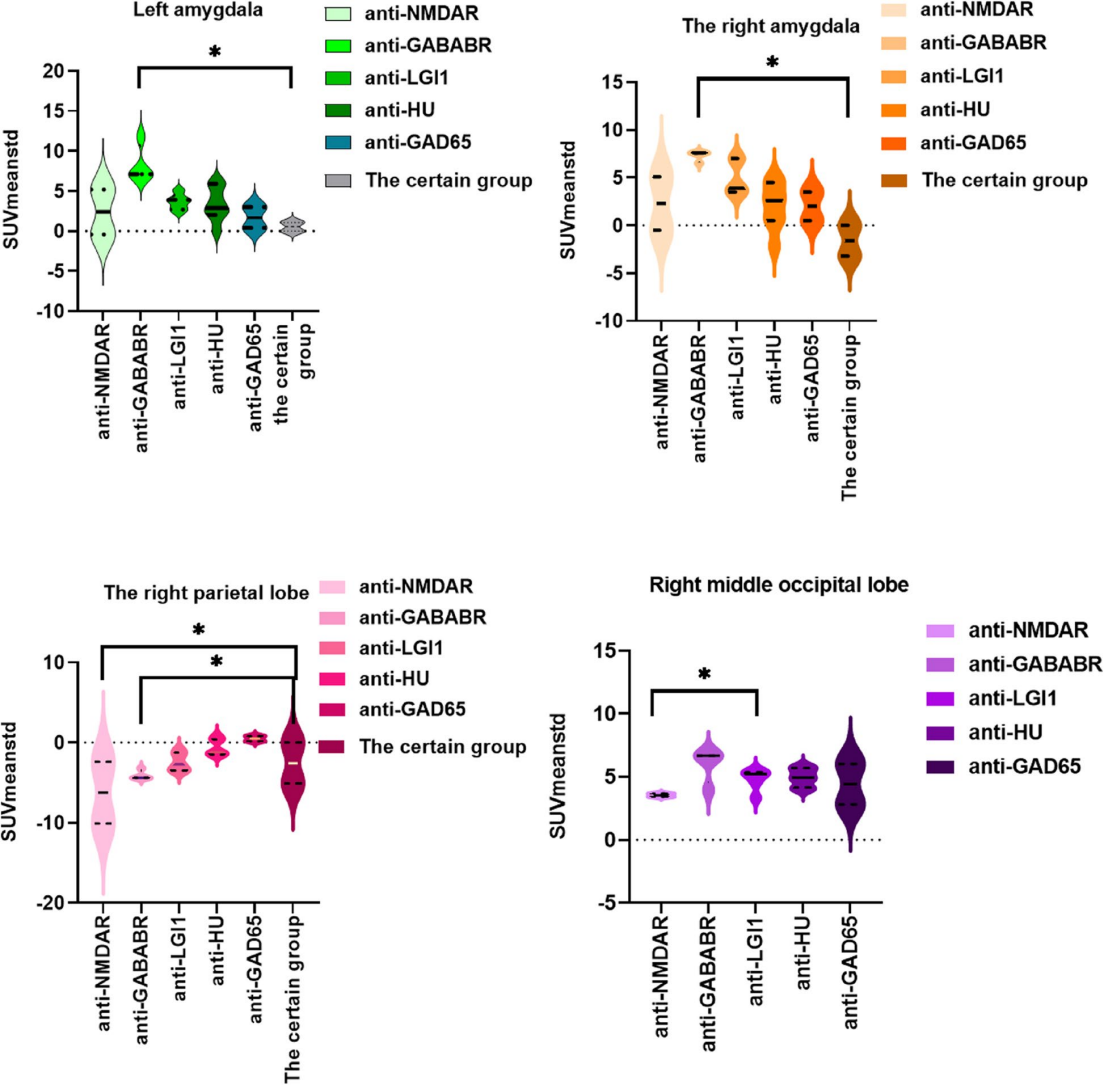
Results of comparative BRRM across different sites in the case groups

As for the SUV_max_, the results revealed significantly high uptakes of the left inferior frontal gyrus (orbital part), left inferior and middle temporal gyri in the anti-GABABR group compared with the certain group (*p* = 0.029, *p* = 0.023, *p* = 0.04), and the results of the former group were higher. There was a significant difference (*p* = 0.023) in the right fusiform gyrus between the anti-GABABR and anti-NMDAR groups (Fig. 4, Supplemental Fig. 1).

For the SUV_mean_, hypermetabolism of the bilateral hippocampus and amygdala were significantly different (left *p* = 0.033, right *p* = 0.029) between the anti-GABABR group and the certain group, accompanied by higher SUVs in the anti-GABABR group. The hypometabolism of the right middle occipital gyrus was significantly different between the anti-NMDAR and anti-LGI1 groups (*p* = 0.018), with that being lower in the former group. There were significant differences in the left supramarginal gyrus and right parietal lobe in the anti-GABABR group, the anti-HU group (*p* = 0.016), and the certain group (*p* = 0.030); the former was lower.

The SUV_mean_ and SUV_max_ of the parietal and occipital lobes in the anti-NMDAR group were lower than those in control group-1, without hypermetabolism of the frontal lobe. The difference was the hypermetabolism of the unilateral hippocampus and cingulate gyrus in patient (No. 2). The other groups were also compared with control group-2, and the top four affected sites were the MTL (hippocampus), basal ganglia, other parts of the temporal lobe, and frontal lobe.

### 18F-FDG PET parameters to predict the severity of this disorder and evaluate the prognosis

**Fig. 5:**
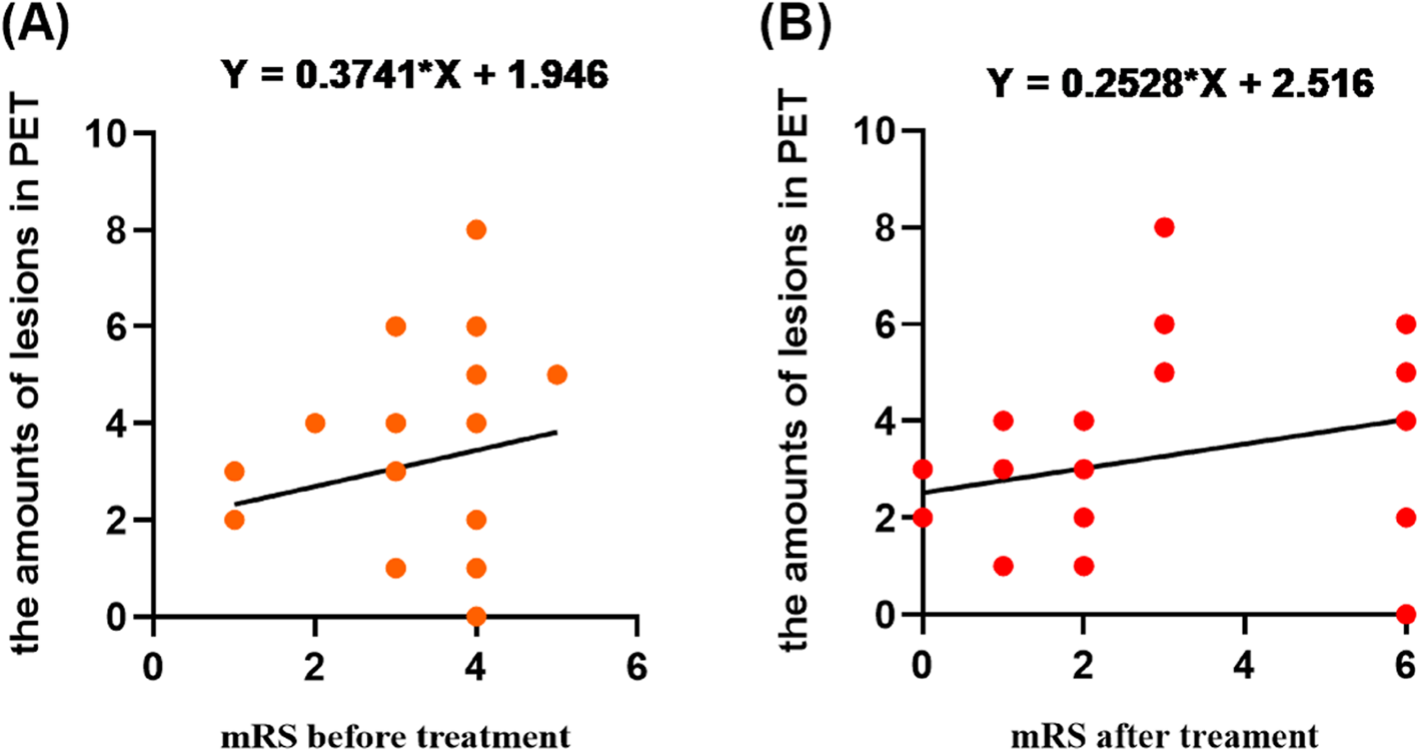
**A** The positive correlation between the amounts of lesions in PET and mRS score before treatment; **B** The positive correlation between the amounts of lesions in PET and mRS score after treatment; however, the former was significant than the latter

**Fig. 6:**
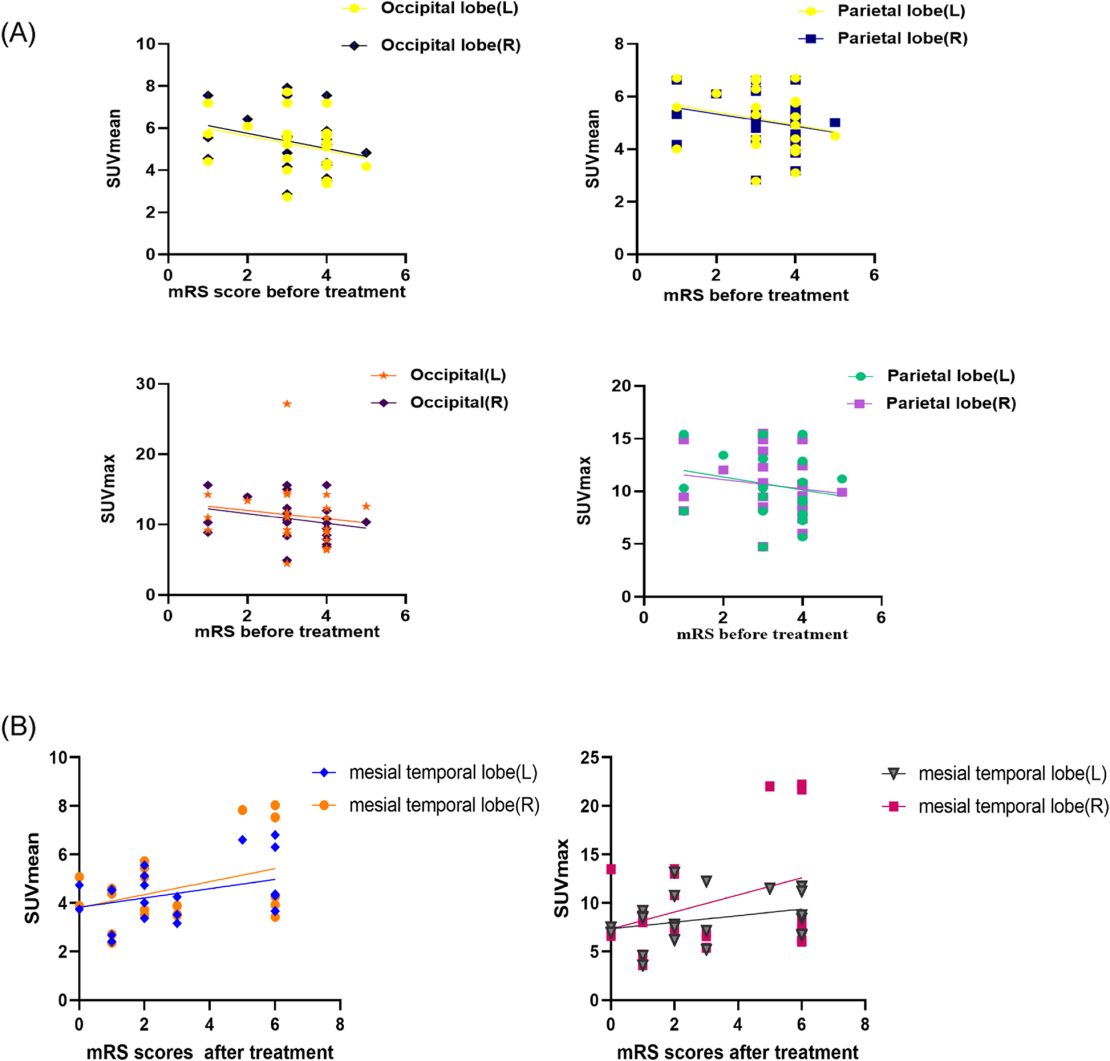
**A** Simple linear regression, to evaluate the relationship among SUV_mean_ and SUV_max_ of BRRM and mRS scores before treatment. SUV_mean_ and SUV_max_ of parietal and occipital lobe had the negative correlation. **B** Simple linear regression, to evaluate the relationship among SUV_mean_ and SUV_max_ of BRRM and mRS scores after treatment. SUV_mean_ and SUV_max_ of MTL had the positive correlation with the mRS score after treatment

As confirmed, the increased mRS scores before and after treatment might be associated with the number of lesions on 18F-FDG PET before treatment (*P* > 0.05). The correlations were positive, which was more significant before the treatment (Fig. 5), (Table 5). It is necessary to find evidence from the SUVs of case groups to evaluate the severity of this disorder before treatment. The SUV_mean_ and SUV_max_ of the unilateral parietal (SUV_mean_, R^2^ = 0.05, *p* > 0.05) and occipital lobes (SUV_mean_, R^2^ = 0.082, *p* > 0.05) were negatively correlated with the mRS scores before treatment (Fig. 6A, Supplemental Fig. 2), and the SUV_mean_ of the unilateral superior temporal gyrus, caudate nucleus, cingulate gyrus, paracingulate gyrus, and frontal gyrus were positively correlated. The SUV_max_ of the bilateral or unilateral basal ganglia (especially the lenticular nucleus and pallidum), amygdala, and frontal gyrus (orbital part) were positively correlated with the mRS scores before treatment, and the SUV_mean_ of the left occipital lobe was the most remarkable result.

**Table 5:** The detailed descriptions of the mRS score before treatment, the mRS score after treatment and the number of lesions on 18F-FDG PET of AE patients

Patient number	Antibody type	Sex	Age (years)	The mRS score before treatment	The mRS score after treatment	The number of lesions on 18F-FDG PET
**1**	NMDAR	Female	36	4	3	8
**2**	NMDAR	Male	34	4	3	6
**3**	LGI1	Female	64	3	2	3
**4**	LGI1	Male	54	1	0	3
**5**	LGI1	Female	40	1	0	3
**6**	LGI1	Male	54	4	1	1
**7**	LGI1	Male	55	3	1	1
**8**	GABABR	Male	66	4	6	2
**9**	GABABR	Female	56	3	6	4
**10**	GABABR	Male	64	4	2	2
**11**	GABABR	Male	57	1	5	2
**12**	HU	Female	55	3	6	6
**13**	HU	Female	67	4	6	0
**14**	HU	Male	77	4	6	5
**15**	HU	Female	60	3	2	1
**16**	SOX1 and HU	Female	51	4	2	4
**17**	SOX1 and HU	Female	63	4	6	4
**18**	HU and RI	Female	66	3	1	1
**19**	GAD65-Ab +	Female	64	3	1	1
**20**	GAD65-Ab +	Female	55	2	1	4
**21**	PNMA2 + /Ma2/Ta	Female	48	5	3	5
**22**	Amphiphysin	Male	63	3	1	3

*Abbreviations*: *AE* Autoimmune Encephalitis, *18F-FDG PET* 2-deoxy-2-[18F]fluoro-D-glucose positron emission tomography, *mRS* modified Rankin Scale, *NMDAR* N-methyl-D-aspartate receptor, *LGI1* leucine-rich glioma inactivated-1, *GABABR* gamma-aminobutyric acid receptor, *GAD65* glutamic acid decarboxylase 65

With respect to prognosis. The SUV_mean_ and SUV_max_ of the MTL, frontal lobe, basal ganglia and parietal lobes before treatment on 18F-FDG PET were positively correlated with the mRS scores after treatment (Fig. 6B, Supplemental Fig. 3), and the SUV_max_ of the MTL was the most notable result (R^2^ = 0.1471, *p* > 0.05) factor.

## Discussion

There were three major highlights and clinical implications in our study. First, it was revealed that the most vulnerable site was the MTL (especially the hippocampus) in AE patients, which showed hypermetabolism by semi-quantitative brain 18F-FDG PET. The results were more convincing because of the large number of controls. The basal ganglia was the second most involved area, which was typical in anti-GABABR, LGI1, HU, and PNMA2 + /Ma2/Ta types in our study. The results of other lobes metabolism were as follows: the metabolism of the frontal lobe in the anti-GABABR group was higher, and the SUV_mean_ and SUV_max_ of the parietal and occipital lobes were lower than controls in the anti-NMDAR group, which were in accordance with the results of Liu X et al. [18] and multiple studies related to anti-NMDAR [19–22]. Second, it was confirmed that 18F-FDG PET can show abnormalities with more sensitivity than MRI in most AE patients. A review of the literature identified 139 patients with AE, 86% with abnormal 18F-FDG PET findings and MRI findings in 59.6% (68/114) [23], whereas there was no MRI abnormality in 10–25% of patients [24]. 18F-FDG PET seems more meticulous and precise.

Third and most importantly, 18F-FDG PET parameters were used to evaluate the severity degree and prognosis. The numbers of focuses on 18F-FDG PET before treatment were more important factors in association with the mRS scores before and after treatment, which was rarely reported in previous literature. It is easy to explain that the more parts of the cerebral cortex involved, the worse the ability to recover function, as seen in our anti-NMDAR and anti-PNMA2 + /Ma2/Ta patients who developed encephalitis. Hypermetabolism of the MTL was common in imaging diagnosis, accompanied by hypometabolism of occipital or parietal lobes. This feature aggravates the severity of this disorder. We also found that the SUV_max_ of the MTL was the most notable factor associated with the mRS scores after treatment.

The hypermetabolism of the MTL was the most remarkable feature in our study diagnosed with anti-GABABR, LGI1, HU, anti-Ma and anti-Ta, and anti-NMDAR encephalitis, which was similar to prior reports [18, 19, 25–30]. The SUV_mean_ of the MTL in the anti-GABABR group was higher than that in the other groups, which might be a reminder that the former more easily involves the MTL. Meanwhile, this manifestation might combine to trigger different types of tumours in the anti-HU group, which was confirmed in this study. Metabolic changes on 18F-FDG PET in the extralimbic regions, consisting of the basal ganglia and occipital, parietal, and frontal lobes. Our three patients in the anti-LGI1 group, who also had faciobrachial dystonic seizures (FBDs); two patients in the anti-GAD65 group; and four patients in the anti-HU group without focal motor status epilepticus (FMSE) all showed hypermetabolism of the basal ganglia, which was as described in the previous literature [31–36]. The different viewpoint was that Valerio Frazzini et al. [37] studied anti-HU patients with FMSE.

It is worth noting that multiple focal infiltrates of inflammatory cells lead to the development of neuronal hyperexcitability and that myoclonic jerks may arise from an atypical propagation of neuronal activity along various networks. Such propagation may differ from that observed in typical motor seizures, resembling the FBDS [38]. Our anti-NMDAR cases without basal ganglia hypermetabolism resemble those reported by Tripathi et al. [39]. In general, neocortical hypometabolism may result from functional impairment propagated along cortical and subcortical networks arising from the sites of primary abnormalities in the MTL and basal ganglia [40]. Overall, hypermetabolism of the MTL and basal ganglia on 18F-FDG PET may be referred as a marker of neuroinflammation in some types of AE [13, 16, 25].

Generally, previous studies [41, 42] have demonstrated that older age, tumours, and convulsive status epilepticus are related to poor prognosis. Liu X et al. [18] and Xinyue Zhang et al. [42] found involvement of the limbic system in the anti-GABAB group on 18F-FDG PET and MRI, which was more common in the poor prognosis group than in the favourable prognosis group, contrary to the viewpoint of Qian Zhao et al. [43] in LGI1 encephalitis. However, in our study, the SUV_max_ of the MTL was the most notable result in six types of AE for prognosis, which was different from one type of antibody. Future prospective studies will be required to verify these findings and explore pathogenic mechanisms.

This study is limited by its retrospective nature and selection bias. Twenty-two patients only underwent 18F-FDG PET in the acute and subacute phases of disease, and two patients with anti-LGI1 and anti-GAD65 group underwent 18F-FDG PET after 1 year of treatment; thus, it will be difficult to evaluate treatment effects. Further prospective and longitudinal cohort studies should be performed.

## Conclusions

In summary, this study provided detailed descriptions of distinct cerebrum metabolic patterns related to acute and subacute phases of AE on 18F-FDG PET, which was more sensitive than MRI. The common pattern of AE was high MTL metabolism on 18F-FDG PET, which was associated with a decreasing SUV_mean_ of the occipital lobe, and the number of lesions on PET before treatment may be significant factors in assessing disease severity. The increasing SUV_max_ of the MTL may serve as a prognostic biomarker in AE. Future prospective studies are required to verify these manifestations and to identify more accurate prognostic factors.

### Supplementary Information

**Additional file 1: Supplemental Fig. 1** Other results of comparative BRRM across different sites in the case groups. **Supplemental Fig. 2 **Simple linear regression, to evaluate the relationship among SUV_mean_ and SUV_max_ of BRRM and mRS scores before treatment, superior temporal lobe(R), caudate nucleus(R), middle frontal gyrus, orbital part (R), pallidums and basal ganglia had the positive relationship before treatment. **Supplemental Fig. 3 **Simple linear regression, to evaluate the relationship among SUV_mean_ and SUV_max_ of BRRM and mRS scores after treatment. SUV_mean_ and SUV_max_ of MTL had the positive correlation with the mRS score after treatment.

**Additional file 2: Supplementary Table 1.** The SUV_max_ of case groups according to AAL standards. **Supplementary Table 2.** The SUV_mean_ of case groups according to AAL standards. **Supplementary Table 3.** The SUV_maxstd_ of case groups according to AAL standards. **Supplementary Table 4.** The SUV_meanstd_ of case groups according to AAL standards. **Supplementary Table 5.** The SUV_mean_ of normal 19-44 years old group according to AAL standards. **Supplementary Table 6.** The SUV_max_ of normal 19-44 years old group according to AAL standards. **Supplementary Table 7.** The SUV_meanstd_ of normal 19-44 years old group according to AAL standard. **Supplementary Table 8.** The SUV_maxstd_ of normal 19-44 years old group according to AAL standard. **Supplementary Table 9.** The SUV_mean_ of normal 45-70 years old group according to AAL standard. **Supplementary Table 10.** The SUV_max_ of normal 45-70 years old group according to AAL standard. **Supplementary Table 11.** The SUV_meanstd_ of normal 45-70 years old group according to AAL standard. **Supplementary Table 12.** The SUV_maxstd_ of normal 45-70 years old group according to AAL standard.

## Data Availability

The datasets generated and analyzed during the study are not publicly available due to patient privacy, but are available from the corresponding author upon reasonable request.

## Abbreviations

AE: Autoimmune Encephalitis
18F-FDG PET: 2-Deoxy-2-[18F]fluoro-D-glucose positron emission tomography
MRI: Magnetic Resonance Imaging
AAL: Anatomical Automatic Labeling
mRS: Modified Rankin Scale
MTL: Mesial Temporal Lobe
SUV_mean_: Mean standardized uptake valve
SUV_max_: Max standardized uptake valve
SUV_meanstd_: Standardized deviation from the SUV_mean_
SUV_maxstd_: Standardized deviation from the SUV_max_
HSV: Herpes simplex virus
ICIs: Immune-checkpoint inhibitors
CRP: C-reactive protein
CSF: Cerebrospinal fluid
TPC: Total protein counts
EEG: Electroencephalogram
WBC: White Blood Cell
CEA: Carcinoembryonic antigen, the normal value < 5 ng/mL
SCC: Squamous cell carcinoma antigen, the normal value < 1.5 ng/mL
CYFRA21-1: Cytokeratin-19-fragment, the normal value < 2.5 ng/mL
NSE: Neuron-specific enolase, the normal value < 17 ng/mL
CA125: The normal value < 35U/ml
CA199: The normal value < 37U/ml
AED: Anti-epileptic drugs; The first line of treatment included steroids, IVIg, and plasma exchange; Long immune treatment included Mycophenolate Mofetil and Azathioprine; The certain group: included four patients, anti-PNMA2+/Ma2/ta, anti-Amphiphysin, anti-CASPR2, anti-GFAP, respectively one case, however, only anti-PNMA2+/Ma2/ta, anti-Amphiphysin performed PET examination
NMDAR: N-methyl-D-aspartate receptor
LGI1: Leucine-rich glioma inactivated-1
CASPR2: Contacting-associated protein-2
GABABR: Gamma-aminobutyric acid receptor
AMPAR: α-Amino-3-hydroxy-5-methyl-4-isoxazole propionic acid receptor
GAD65: Glutamic acid decarboxylase 65
BRRM: Brain relative regional metabolism
T1WI: T1 weighted image
T2WI: T2 weighted image
FLAIR: Fluid attenuated inversion recovery
TR: Repetition time
TE: Echo time
FOV: Field of view
AI: Asymmetric Index
FBDs: Faciobrachial Dystonic Seizure
FMSE: Focal Motor Status Epilepticus
