# Supplementary material for: Enhancing the clinical diagnosis of the acute and subacute phases of autoimmune encephalitis and predicting the risk factors: the potential advantages of 18F-FDG PET/CT
Source: BMC Med Imaging. 2023 Nov 20;23:193. doi: 10.1186/s12880-023-01148-6 (PMC10662540; doi:10.1186/s12880-023-01148-6)
Supplement: Supplementary file 1 — Additional file 1: Supplemental Fig. 1 Other results of comparative BRRM across different sites in the case groups. Supplemental Fig. 2 Simple linear regression, to evaluate the relationship among SUVmean and SUVmax of BRRM and mRS scores before treatment, superior temporal lobe(R), caudate nucleus(R), middle frontal gyrus, orbital part (R), pallidums and basal ganglia had the positive relationship before treatment. Supplemental Fig. 3 Simple linear regression, to evaluate the relationship among SUVmean and SUVmax of BRRM and mRS scores after treatment. SUVmean and SUVmax of MTL had the positive correlation with the mRS score after treatment. [file 12880_2023_1148_MOESM1_ESM.docx]

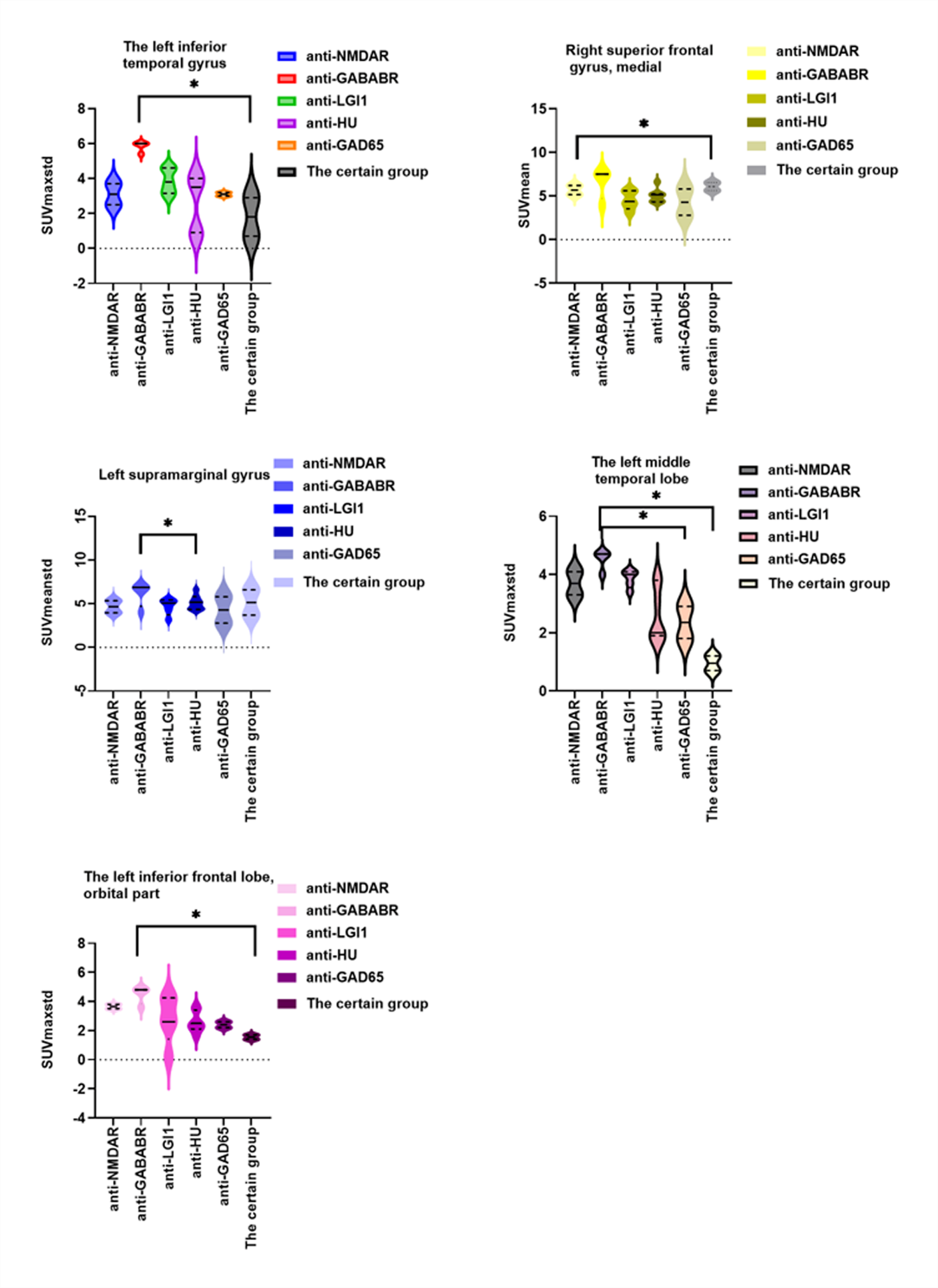


**Supplemental Fig.1**  Other results of comparative BRRM across different sites in the case groups.


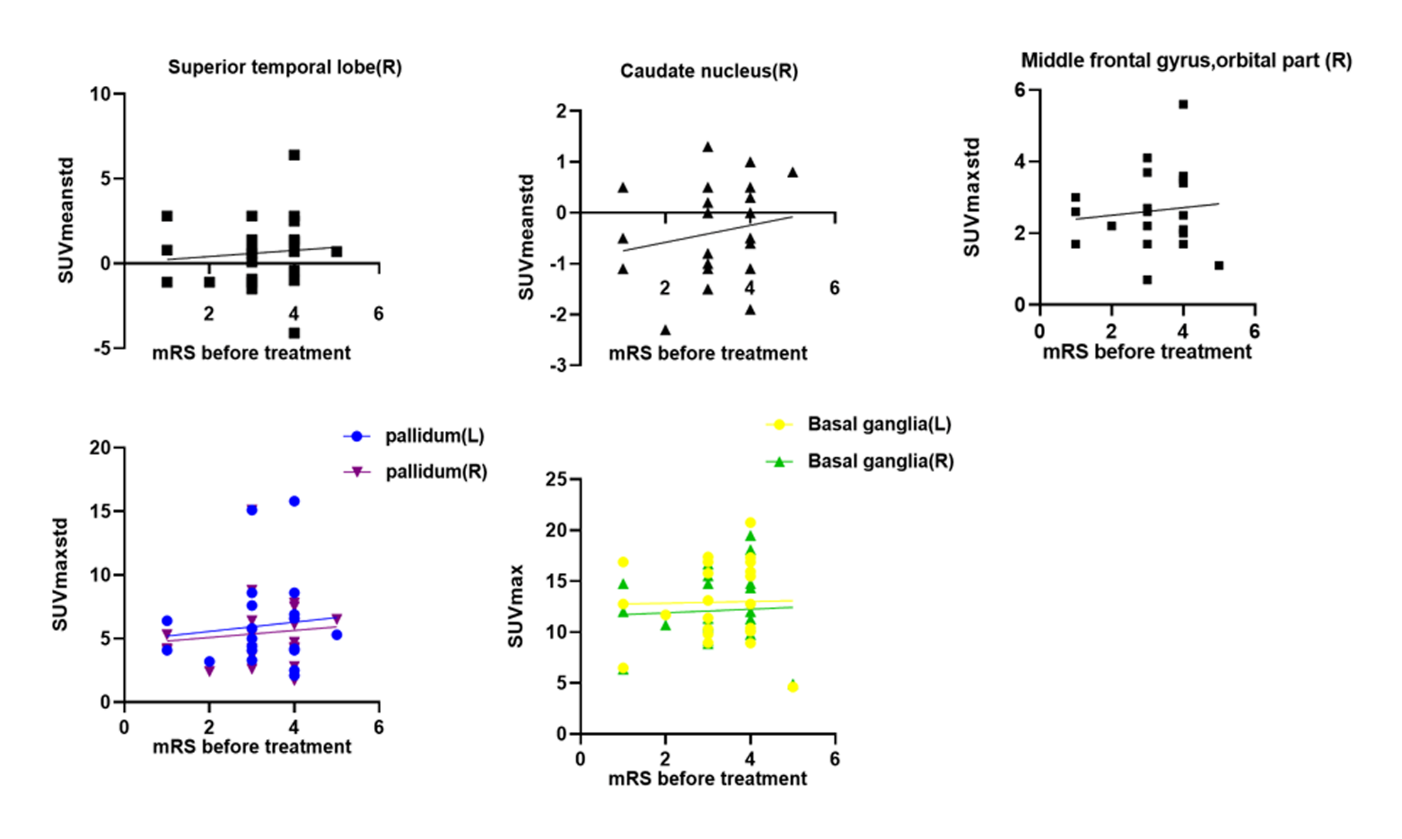


**Supplemental Fig.2** Simple linear regression, to evaluate the relationship among SUV_mean_ and SUV_max_ of BRRM and mRS scores before treatment, superior temporal lobe(R), caudate nucleus(R), middle frontal gyrus, orbital part (R), pallidums and basal ganglia had the positive relationship before treatment.


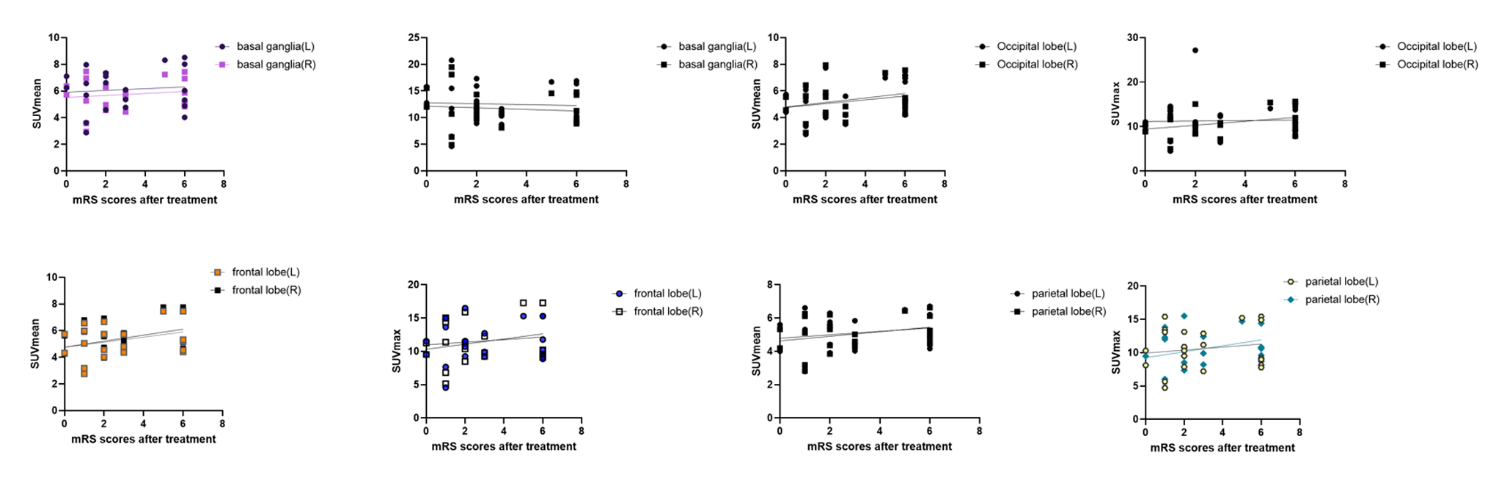


**Supplemental Fig.3** Simple linear regression, to evaluate the relationship among SUV_mean_ and SUV_max_ of BRRM and mRS scores after treatment. SUV_mean_ and SUV_max_ of MTL had the positive correlation with the mRS score after treatment.
