# Supplementary material for: Enhancing the clinical diagnosis of the acute and subacute phases of autoimmune encephalitis and predicting the risk factors: the potential advantages of 18F-FDG PET/CT
Source: BMC Med Imaging. 2023 Nov 20;23:193. doi: 10.1186/s12880-023-01148-6 (PMC10662540; doi:10.1186/s12880-023-01148-6)
Supplement: Supplementary file 2 — Additional file 2: Supplementary Table 1. The SUVmax of case groups according to AAL standards. Supplementary Table 2. The SUVmean of case groups according to AAL standards. Supplementary Table 3. The SUVmaxstd of case groups according to AAL standards. Supplementary Table 4. The SUVmeanstd of case groups according to AAL standards. Supplementary Table 5. The SUVmean of normal 19-44 years old group according to AAL standards. Supplementary Table 6. The SUVmax of normal 19-44 years old group according to AAL standards. Supplementary Table 7. The SUVmeanstd of normal 19-44 years old group according to AAL standard. Supplementary Table 8. The SUVmaxstd of normal 19-44 years old group according to AAL standard. Supplementary Table 9. The SUVmean of normal 45-70 years old group according to AAL standard. Supplementary Table 10. The SUVmax of normal 45-70 years old group according to AAL standard. Supplementary Table 11. The SUVmeanstd of normal 45-70 years old group according to AAL standard. Supplementary Table 12. The SUVmaxstd of normal 45-70 years old group according to AAL standard. [file 12880_2023_1148_MOESM2_ESM.docx]

Supplementary Table 1 The SUV_max_ of case groups according to AAL standards.

| Region of Interest | Amygdala | Amygdala | Angular gyrus | Angular gyrus | Anterior cingulate and paracingulate gyri | Anterior cingulate and paracingulate gyri | Basal ganglia | Basal ganglia | Calcarine fissure and surrounding cortex | Calcarine fissure and surrounding cortex | Caudate nucleus | Caudate nucleus | Central region | Central region | Cingulate and paracingulate gyri | Cingulate and paracingulate gyri | Cuneus | Cuneus |
| --- | --- | --- | --- | --- | --- | --- | --- | --- | --- | --- | --- | --- | --- | --- | --- | --- | --- | --- |
| Side | L | R | L | R | L | R | L | R | L | R | L | R | L | R | L | R | L | R |
| GABABR-1 | 10.25 | 16.92 | 11.72 | 10.84 | 13.65 | 14.4 | 16.89 | 14.75 | 14.25 | 15.61 | 13.59 | 12.43 | 12.75 | 15.42 | 15.29 | 14.4 | 14.07 | 15.46 |
| GABABR-2 | 7.77 | 6.96 | 6.87 | 6.31 | 6.62 | 6.65 | 8.92 | 9.75 | 8.61 | 8.49 | 6.72 | 7.27 | 7.6 | 7.74 | 7.65 | 7.21 | 8.36 | 8.19 |
| GABABR-3 | 9.75 | 16.42 | 11.22 | 10.34 | 13.15 | 13.9 | 16.39 | 14.25 | 13.75 | 15.11 | 13.09 | 11.93 | 12.25 | 14.92 | 14.79 | 13.9 | 13.57 | 14.96 |
| GABABR-4 | 10.05 | 16.72 | 11.52 | 10.64 | 13.45 | 14.2 | 16.69 | 14.55 | 14.05 | 15.41 | 13.39 | 12.23 | 12.55 | 15.22 | 15.09 | 14.2 | 13.87 | 15.26 |
| GAD65-5 | 6.24 | 6.14 | 10.72 | 10.29 | 9.32 | 9.25 | 11.69 | 10.69 | 13.39 | 13.96 | 10.21 | 9.61 | 11.34 | 11.75 | 12.13 | 13.16 | 12.41 | 11.66 |
| GAD65-6 | 8.97 | 8.39 | 12.84 | 13.84 | 13.29 | 14.07 | 17.39 | 16.68 | 17.16 | 18.72 | 16.09 | 15.52 | 17.09 | 17.23 | 16.65 | 17 | 17.25 | 16.27 |
| GAD65-7 | 2.89 | 2.98 | 4.14 | 4.25 | 3.74 | 3.98 | 4.61 | 4.91 | 4.45 | 4.97 | 4.33 | 4.56 | 4.79 | 4.46 | 4.88 | 4.52 | 4.23 | 4.44 |
| PNMA2-8 | 11.53 | 4.71 | 11.18 | 8.79 | 8.2 | 9.54 | 10.32 | 10.69 | 9.12 | 9.98 | 8.45 | 9.69 | 10.71 | 10.32 | 9.35 | 10.78 | 12.57 | 10.33 |
| AMPHIN-9 | 8.74 | 7.84 | 9.12 | 12.86 | 11.8 | 11.54 | 15.94 | 14.35 | 27.16 | 14.95 | 11.45 | 12.69 | 13.78 | 15.97 | 12.81 | 15.54 | 23.5 | 14.15 |
| HU+SOX1-10 | 6.9 | 6.98 | 7.17 | 8.18 | 7.27 | 8.25 | 10.41 | 9.75 | 10.16 | 11.96 | 9.01 | 8.42 | 10.64 | 9.27 | 9.29 | 9.59 | 10.44 | 9.53 |
| HU+SOX1-11 | 10.96 | 8.46 | 8.92 | 9.35 | 8.79 | 8.61 | 13.11 | 11.35 | 10.58 | 10 | 11.63 | 10.59 | 11.2 | 11.27 | 10.22 | 10.04 | 9.44 | 9.87 |
| HU-12 | 6.08 | 5.37 | 7.82 | 7.91 | 6.42 | 6.81 | 17.32 | 10.6 | 8.08 | 7.74 | 17.32 | 8.24 | 10.29 | 10.38 | 9.51 | 9.32 | 8.63 | 8.36 |
| HU-13 | 5.6 | 5.51 | 7.83 | 8.9 | 7.84 | 7.52 | 9.81 | 9.16 | 8.42 | 8.06 | 8.72 | 8.93 | 9.1 | 8.7 | 10.35 | 10.31 | 8.87 | 9.42 |
| HU-14 | 5.08 | 5.12 | 7.74 | 8.39 | 10.9 | 8.5 | 10.07 | 11.28 | 8.95 | 8.53 | 8.62 | 9.46 | 7.99 | 10.39 | 10.9 | 9.2 | 9.24 | 10.63 |
| HU-15 | 6.13 | 6.64 | 6.19 | 6.87 | 6.43 | 5.96 | 8.94 | 8.86 | 6.94 | 7.37 | 8.05 | 8.35 | 8.21 | 8.82 | 8.09 | 7.99 | 7.52 | 7.88 |
| HU-16 | 7.58 | 6.34 | 14.39 | 12.3 | 11.62 | 11.99 | 20.76 | 19.49 | 12.27 | 10.01 | 14.69 | 17.03 | 13.48 | 13.91 | 15.08 | 15.01 | 14.54 | 11.58 |
| LGI1-17 | 3.58 | 3.65 | 5.13 | 6.03 | 4.78 | 5.19 | 6.49 | 6.36 | 6.58 | 6.4 | 6.49 | 6.31 | 6.5 | 6.47 | 5.29 | 5.49 | 5.1 | 5.86 |
| LGI1-18 | 6.04 | 8.13 | 9.55 | 9.48 | 9.55 | 8.72 | 12.77 | 12.02 | 11 | 10.14 | 11.26 | 11.22 | 11.24 | 11.02 | 10.67 | 10.58 | 10.39 | 10.28 |
| LGI1-19 | 6.17 | 5.64 | 7.34 | 7.63 | 7.75 | 7.45 | 15.78 | 15.54 | 7.59 | 7.55 | 15.78 | 11.86 | 8.75 | 8.68 | 8.04 | 7.45 | 7.81 | 6.93 |
| LGI1-20 | 6.53 | 5.6 | 9.94 | 10.5 | 7.98 | 8.32 | 11.38 | 10.52 | 11.65 | 11.15 | 9.4 | 10.52 | 11.25 | 11.7 | 10.2 | 11.03 | 10.88 | 11.86 |
| LGI1-21 | 7.27 | 7.9 | 9.82 | 10.17 | 9.67 | 11.62 | 15.49 | 18.09 | 10.55 | 11.09 | 10.79 | 16.85 | 16.32 | 14.28 | 11.73 | 13.23 | 11.67 | 11.77 |
| LGI1-22 | 6 | 8.09 | 9.51 | 9.44 | 9.51 | 8.68 | 12.73 | 11.98 | 10.96 | 10.1 | 11.22 | 11.18 | 11.2 | 10.98 | 10.63 | 10.54 | 10.35 | 10.24 |
| NMDAR-23 | 5.2 | 5.38 | 5.99 | 6.14 | 7.42 | 8.92 | 8.72 | 8.11 | 6.23 | 7.05 | 7.26 | 6.8 | 7.76 | 8.23 | 8.81 | 8.92 | 6.39 | 6.18 |
| NMDAR-24 | 5.32 | 5.22 | 9.85 | 8.09 | 8.64 | 9.95 | 11.66 | 10.93 | 11.08 | 9.75 | 11.66 | 10.6 | 11.48 | 9.19 | 12.97 | 12.62 | 12.28 | 10.78 |

| Region of Interest | Frontal lobe | Frontal lobe | Fusiform gyrus | Fusiform gyrus | Gyrus rectus | Gyrus rectus | Heschl gyrus | Heschl gyrus | Hippocampus | Hippocampus | Inferior frontal gyrus, opercular part | Inferior frontal gyrus, opercular part | Inferior frontal gyrus, orbital part | Inferior frontal gyrus, orbital part | Inferior frontal gyrus, triangular part | Inferior frontal gyrus, triangular part | Inferior occipital gyrus | Inferior occipital gyrus |
| --- | --- | --- | --- | --- | --- | --- | --- | --- | --- | --- | --- | --- | --- | --- | --- | --- | --- | --- |
| Side | L | R | L | R | L | R | L | R | L | R | L | R | L | R | L | R | L | R |
| GABABR-1 | 15.29 | 17.27 | 13.2 | 14.75 | 14.56 | 13.25 | 12.08 | 16.07 | 10.48 | 22.2 | 13.29 | 17.27 | 13.95 | 13.7 | 13.48 | 16.77 | 12.31 | 12.98 |
| GABABR-2 | 9.25 | 8.47 | 7.8 | 7.93 | 7.74 | 6.48 | 7.57 | 6.76 | 7.24 | 6.75 | 7.32 | 7.51 | 7.32 | 7.69 | 7.15 | 6.65 | 7.47 | 7.77 |
| GABABR-3 | 14.79 | 16.77 | 12.7 | 14.25 | 14.06 | 12.75 | 11.58 | 15.57 | 9.98 | 21.7 | 12.79 | 16.77 | 13.45 | 13.2 | 12.98 | 16.27 | 11.81 | 12.48 |
| GABABR-4 | 15.09 | 17.07 | 13 | 14.55 | 14.36 | 13.05 | 11.88 | 15.87 | 10.28 | 22 | 13.09 | 17.07 | 13.75 | 13.5 | 13.28 | 16.57 | 12.11 | 12.78 |
| GAD65-5 | 13.64 | 11.37 | 9.75 | 9.92 | 8.82 | 8.03 | 10.46 | 11.68 | 8.04 | 7.29 | 10.48 | 10.19 | 10.22 | 11.36 | 10.84 | 10.97 | 10.68 | 11.37 |
| GAD65-6 | 19.78 | 18.6 | 13.2 | 12.98 | 12.01 | 11.89 | 14.93 | 17.18 | 10.13 | 9.5 | 15.26 | 16.71 | 14.92 | 16.64 | 15.32 | 16.38 | 16.56 | 17.47 |
| GAD65-7 | 4.56 | 5.14 | 3.77 | 3.82 | 3.99 | 3.85 | 4.08 | 4.1 | 3.58 | 3.38 | 4.09 | 4.1 | 4.31 | 4.41 | 4.15 | 4.44 | 4.09 | 4.41 |
| PNMA2-8 | 9.91 | 9.78 | 5.97 | 7.26 | 7.7 | 8.09 | 9.82 | 8.47 | 12.22 | 6.7 | 7.79 | 9.43 | 8.45 | 9.11 | 9.07 | 8.41 | 7.56 | 7.11 |
| AMPHIN-9 | 16.49 | 15.89 | 12.39 | 13.37 | 11 | 12.96 | 13.05 | 16.79 | 9.06 | 9.6 | 13.84 | 13.54 | 12.39 | 13.6 | 12.84 | 14.05 | 15.21 | 14.37 |
| HU+SOX1-10 | 10.2 | 10.22 | 10.33 | 9.58 | 7.27 | 7.29 | 8.98 | 8.41 | 6 | 7.06 | 9.32 | 8.68 | 8.89 | 8.28 | 8.82 | 9.82 | 10.48 | 11.85 |
| HU+SOX1-11 | 11.54 | 11.16 | 9.95 | 9.86 | 8.51 | 7.4 | 11.5 | 10.92 | 13.14 | 13.03 | 10.02 | 8.9 | 8.68 | 8.26 | 10.78 | 9.22 | 9.97 | 9.94 |
| HU-12 | 10.71 | 10.38 | 6.24 | 6.54 | 6.3 | 7.26 | 8.47 | 8.61 | 6.18 | 6.94 | 8.49 | 7.63 | 7.6 | 8.3 | 8.72 | 8.05 | 6.12 | 6.65 |
| HU-13 | 9.23 | 9.07 | 7.16 | 7.37 | 7.61 | 7.19 | 6.83 | 7.79 | 6.62 | 6.04 | 8.29 | 8.02 | 8.76 | 8.48 | 8.19 | 8.52 | 7.67 | 7.58 |
| HU-14 | 11.78 | 9.23 | 8.07 | 7.4 | 6.48 | 7.03 | 6.44 | 7.7 | 7.63 | 5.88 | 6.43 | 7.91 | 7.92 | 9.19 | 8.32 | 8.65 | 7.31 | 7.15 |
| HU-15 | 8.87 | 9.6 | 5.69 | 5.99 | 8.13 | 8.3 | 7.05 | 7.67 | 6.74 | 7.28 | 6.69 | 7.12 | 7.54 | 8.2 | 7.33 | 7.07 | 5.85 | 5.8 |
| HU-16 | 14.99 | 15.04 | 8.62 | 8.8 | 11.71 | 10.6 | 9.53 | 9.61 | 8.55 | 7.66 | 11.72 | 13.37 | 12.89 | 13.64 | 13.29 | 11.97 | 10.11 | 10.33 |
| LGI1-17 | 7.65 | 6.81 | 6 | 5.48 | 5.1 | 4.58 | 5.83 | 5.13 | 4.16 | 4.08 | 6.17 | 5.83 | 5.14 | 4.99 | 5.31 | 5.92 | 6.03 | 6.06 |
| LGI1-18 | 11.54 | 11.26 | 8.41 | 8.77 | 8.93 | 8 | 11 | 10.26 | 7.49 | 13.48 | 10.61 | 9.88 | 9.45 | 8.78 | 11.1 | 9.6 | 9.14 | 9.92 |
| LGI1-19 | 9.52 | 9.52 | 7.59 | 7.85 | 7.45 | 7.42 | 7.56 | 7.38 | 6.48 | 6.52 | 8.28 | 8.35 | 7.61 | 7.24 | 8.22 | 7.67 | 7.62 | 8.86 |
| LGI1-20 | 12.49 | 11.39 | 9.4 | 9.58 | 9.47 | 8.23 | 9.76 | 8.76 | 6.93 | 7.01 | 12.08 | 9.58 | 9.49 | 10.78 | 11.48 | 10.65 | 12.25 | 11.79 |
| LGI1-21 | 14.96 | 14.36 | 10.17 | 11.32 | 7.9 | 8.8 | 10.61 | 12.78 | 8.47 | 7.43 | 12.35 | 11.98 | 9.26 | 10.4 | 12.41 | 11.31 | 11.04 | 9.83 |
| LGI1-22 | 11.5 | 11.22 | 8.37 | 8.73 | 8.89 | 7.96 | 10.96 | 10.22 | 7.45 | 13.44 | 10.57 | 9.84 | 9.41 | 8.74 | 11.06 | 9.56 | 9.1 | 9.88 |
| NMDAR-23 | 9.2 | 9.24 | 5.98 | 5.66 | 7.78 | 7.66 | 5.93 | 7.53 | 5.2 | 5.27 | 8.08 | 8.1 | 7.1 | 9.21 | 7.25 | 9.24 | 5.66 | 5.97 |
| NMDAR-24 | 12.71 | 12.26 | 8.08 | 6.25 | 9.73 | 10.64 | 10.57 | 6.27 | 7.13 | 6.58 | 10.8 | 9.6 | 11.41 | 11.26 | 10.37 | 9.27 | 8.81 | 6.48 |

| Region of Interest | Inferior parietal, but supramarginal and angular gyri | Inferior parietal, but supramarginal and angular gyri | Inferior temporal gyrus | Inferior temporal gyrus | Insula | Insula | Lenticular nucleus, pallidum | Lenticular nucleus, pallidum | Lenticular nucleus, putamen | Lenticular nucleus, putamen | Lingual gyrus | Lingual gyrus | Mesial temporal lobe | Mesial temporal lobe | Middle cingulate and paracingulate gyri | Middle cingulate and paracingulate gyri | Middle frontal gyrus | Middle frontal gyrus |
| --- | --- | --- | --- | --- | --- | --- | --- | --- | --- | --- | --- | --- | --- | --- | --- | --- | --- | --- |
| Side | L | R | L | R | L | R | L | R | L | R | L | R | L | R | L | R | L | R |
| GABABR-1 | 11.12 | 10.38 | 13.21 | 14.67 | 13.45 | 16.95 | 10.82 | 8.03 | 16.89 | 14.75 | 12.95 | 14.4 | 11.69 | 22.2 | 15.29 | 14.39 | 13.99 | 14.39 |
| GABABR-2 | 6.8 | 7.29 | 8.3 | 8.07 | 7.3 | 6.42 | 7.04 | 6.46 | 8.92 | 9.75 | 9.36 | 8.07 | 7.77 | 7.52 | 7.65 | 7.21 | 8.98 | 8.47 |
| GABABR-3 | 10.62 | 9.88 | 12.71 | 14.17 | 12.95 | 16.45 | 10.32 | 7.53 | 16.39 | 14.25 | 12.45 | 13.9 | 11.19 | 21.7 | 14.79 | 13.89 | 13.49 | 13.89 |
| GABABR-4 | 10.92 | 10.18 | 13.01 | 14.47 | 13.25 | 16.75 | 10.62 | 7.83 | 16.69 | 14.55 | 12.75 | 14.2 | 11.49 | 22 | 15.09 | 14.19 | 13.79 | 14.19 |
| GAD65-5 | 10.84 | 10.3 | 9.88 | 9.31 | 10.47 | 9.87 | 8.87 | 7.09 | 11.69 | 10.69 | 12.79 | 13.24 | 9.17 | 8.79 | 12.13 | 13.16 | 13.64 | 11.23 |
| GAD65-6 | 15.27 | 15.03 | 13.37 | 12.97 | 15.88 | 14.17 | 12.7 | 10.37 | 17.39 | 16.68 | 16.29 | 15.41 | 12.65 | 11.55 | 16.65 | 17 | 19.78 | 17.7 |
| GAD65-7 | 4.36 | 4.33 | 3.78 | 4 | 4.06 | 3.95 | 3.9 | 3.52 | 4.61 | 4.91 | 3.97 | 4.1 | 3.58 | 3.6 | 4.88 | 4.52 | 4.44 | 5.14 |
| PNMA2-8 | 7.77 | 7.48 | 5.71 | 8.2 | 9.34 | 8.99 | 9.29 | 8.09 | 10.32 | 10.69 | 7.97 | 8.45 | 12.22 | 6.81 | 9.24 | 10.78 | 9.63 | 9.78 |
| AMPHIN-9 | 10.85 | 11.91 | 10.38 | 11.23 | 11.3 | 13.73 | 9.03 | 8.42 | 15.94 | 14.35 | 15.85 | 15.04 | 10.74 | 10.83 | 12.81 | 15.54 | 14.8 | 14.78 |
| HU+SOX1-10 | 7.97 | 8.19 | 9.13 | 9.41 | 8.7 | 8.81 | 7.88 | 6.64 | 10.41 | 9.75 | 10.91 | 10.32 | 8.39 | 8.22 | 8.78 | 9.59 | 10.2 | 9.53 |
| HU+SOX1-11 | 9.93 | 8.97 | 9.11 | 9.78 | 9.12 | 9.04 | 10.08 | 7.6 | 13.11 | 11.35 | 9.47 | 9.39 | 13.14 | 13.03 | 10.22 | 10.04 | 11.54 | 10.62 |
| HU-12 | 7.6 | 8.16 | 6.68 | 7.09 | 7.97 | 7.56 | 10.85 | 5.41 | 16.11 | 10.6 | 8.32 | 7.83 | 6.18 | 6.94 | 9.51 | 9.32 | 9.28 | 9.44 |
| HU-13 | 8.75 | 9.16 | 8.28 | 7.97 | 8.42 | 8.23 | 7.73 | 6.74 | 9.81 | 9.16 | 7.72 | 7.69 | 6.64 | 6.15 | 10.35 | 10.31 | 9.23 | 9.07 |
| HU-14 | 7.41 | 7.44 | 6.6 | 7.17 | 7.28 | 7.78 | 6.1 | 7.77 | 10.07 | 11.28 | 7.28 | 7.85 | 8.73 | 6.01 | 8.22 | 9.2 | 8.9 | 8.86 |
| HU-15 | 6.67 | 7.15 | 7.33 | 7.16 | 6.36 | 6.5 | 6.78 | 6.65 | 8.94 | 8.86 | 6.23 | 6.29 | 6.74 | 7.28 | 8.09 | 7.99 | 7.76 | 7.82 |
| HU-16 | 13.28 | 12 | 11.89 | 10.8 | 11.12 | 10.95 | 16.02 | 12.06 | 20.76 | 19.49 | 9.5 | 9.34 | 8.55 | 8.03 | 14.66 | 15.01 | 13.22 | 15.04 |
| LGI1-17 | 5.44 | 5.71 | 5.78 | 5.37 | 5.77 | 5.53 | 5.4 | 4.73 | 6.35 | 6.36 | 6.62 | 6.85 | 4.56 | 4.69 | 5.29 | 5.49 | 6.32 | 6.53 |
| LGI1-18 | 10.3 | 8.86 | 8.86 | 8.53 | 9.28 | 9.52 | 8.33 | 7.5 | 12.77 | 12.02 | 8.84 | 9.64 | 7.49 | 13.48 | 10.22 | 10.58 | 11.54 | 11.1 |
| LGI1-19 | 7.49 | 7.72 | 7.27 | 7.41 | 8.16 | 7.61 | 9.18 | 8.67 | 14.08 | 15.54 | 7.2 | 7.58 | 6.94 | 6.62 | 8.04 | 7.39 | 8.69 | 8.53 |
| LGI1-20 | 9.91 | 10.34 | 9.72 | 9.33 | 9.33 | 8.35 | 8.14 | 8.05 | 11.38 | 10.32 | 9.69 | 10.64 | 7.82 | 7.62 | 10.2 | 11.03 | 12.29 | 11.23 |
| LGI1-21 | 11.79 | 12.13 | 9.8 | 10.59 | 11.93 | 10.85 | 9.98 | 6.85 | 15.49 | 18.09 | 10.64 | 11.37 | 8.47 | 8.74 | 11.73 | 13.23 | 12.21 | 14.07 |
| LGI1-22 | 10.26 | 8.82 | 8.82 | 8.49 | 9.24 | 9.48 | 8.29 | 7.46 | 12.73 | 11.98 | 8.8 | 9.6 | 7.45 | 13.44 | 10.18 | 10.54 | 11.5 | 11.06 |
| NMDAR-23 | 6.01 | 7.51 | 6.3 | 7.05 | 7.21 | 9.57 | 5.9 | 5.94 | 8.72 | 8.11 | 6.41 | 7.15 | 5.2 | 5.38 | 8.81 | 8.66 | 6.97 | 9.2 |
| NMDAR-24 | 10.29 | 7.29 | 9.16 | 5.52 | 11.05 | 10.64 | 7.48 | 5.32 | 10.54 | 10.93 | 9.1 | 8.13 | 7.13 | 6.58 | 12.97 | 12.62 | 11.16 | 10 |

| Region of Interest | Middle frontal gyrus, orbital part | Middle frontal gyrus, orbital part | Middle occipital gyrus | Middle occipital gyrus | Middle temporal gyrus | Middle temporal gyrus | Occipital lobe | Occipital lobe | Olfactory cortex | Olfactory cortex | Paracentral lobule | Paracentral lobule | Parahippocampal gyrus | Parahippocampal gyrus | Parietal lobe | Parietal lobe | Postcentral gyrus | Postcentral gyrus |
| --- | --- | --- | --- | --- | --- | --- | --- | --- | --- | --- | --- | --- | --- | --- | --- | --- | --- | --- |
| Side | L | R | L | R | L | R | L | R | L | R | L | R | L | R | L | R | L | R |
| GABABR-1 | 15.29 | 13.92 | 12.21 | 12.53 | 12.3 | 14.67 | 14.25 | 15.61 | 12.48 | 11.76 | 14.84 | 12.22 | 11.69 | 15.78 | 15.43 | 14.9 | 12.75 | 14.36 |
| GABABR-2 | 9.25 | 8.2 | 8.2 | 7.79 | 8.08 | 8.41 | 9.36 | 8.49 | 5.19 | 5.37 | 7.19 | 6.38 | 6.32 | 7.52 | 7.88 | 7.35 | 7.15 | 7.21 |
| GABABR-3 | 14.79 | 13.42 | 11.71 | 12.03 | 11.8 | 14.17 | 13.75 | 15.11 | 11.98 | 11.26 | 14.34 | 11.72 | 11.19 | 15.28 | 14.93 | 14.4 | 12.25 | 13.86 |
| GABABR-4 | 15.09 | 13.72 | 12.01 | 12.33 | 12.1 | 14.47 | 14.05 | 15.41 | 12.28 | 11.56 | 14.64 | 12.02 | 11.49 | 15.58 | 15.23 | 14.7 | 12.55 | 14.16 |
| GAD65-5 | 10.45 | 8.79 | 11.61 | 10.72 | 10.29 | 10.1 | 13.39 | 13.96 | 7.72 | 8.1 | 11.44 | 11.34 | 9.17 | 8.79 | 13.43 | 12.02 | 10.93 | 10.39 |
| GAD65-6 | 15.34 | 13.53 | 17.68 | 18.4 | 13.37 | 12.99 | 17.68 | 18.72 | 12.11 | 11.06 | 15.46 | 15.34 | 12.65 | 11.55 | 15.83 | 16.1 | 14.97 | 16.66 |
| GAD65-7 | 4.07 | 4.21 | 4.3 | 4.27 | 4.4 | 4.21 | 4.45 | 4.97 | 3.56 | 3.2 | 4.56 | 4.57 | 3.23 | 3.6 | 4.73 | 4.76 | 4.59 | 4.27 |
| PNMA2-8 | 8.02 | 9.21 | 12 | 7.77 | 9.75 | 8.69 | 12.57 | 10.33 | 5.77 | 7.17 | 9.91 | 8.86 | 10.15 | 6.81 | 11.18 | 9.91 | 10.71 | 9.67 |
| AMPHIN-9 | 13.07 | 13.33 | 21.61 | 13.27 | 13.13 | 13.09 | 27.16 | 15.04 | 9.04 | 8.38 | 13.81 | 12.53 | 10.74 | 10.83 | 13.12 | 15.51 | 13.78 | 13.71 |
| HU+SOX1-10 | 8.67 | 8.32 | 9.14 | 11.16 | 9.73 | 9.53 | 10.91 | 11.96 | 7.11 | 6.8 | 8.56 | 8.51 | 8.39 | 8.22 | 9.2 | 10.62 | 8.69 | 8.59 |
| HU+SOX1-11 | 8.64 | 9.11 | 10.96 | 9.92 | 11.15 | 10.44 | 10.96 | 10.16 | 8.77 | 7.65 | 11.28 | 9.97 | 11.57 | 12.05 | 10.87 | 10.71 | 10.39 | 10.28 |
| HU-12 | 7.87 | 8.37 | 6.59 | 7.64 | 8.23 | 8.37 | 8.63 | 8.36 | 6.7 | 6.67 | 10.71 | 9.05 | 5.69 | 6.05 | 9.51 | 8.52 | 9.61 | 10.38 |
| HU-13 | 8.69 | 8.45 | 8.62 | 8 | 8.4 | 8.47 | 8.87 | 9.42 | 7.79 | 7.1 | 7.89 | 8.08 | 6.64 | 6.15 | 8.98 | 9.61 | 8.51 | 7.75 |
| HU-14 | 8.07 | 8.54 | 8.28 | 8.42 | 7.76 | 8.34 | 9.24 | 10.63 | 5.41 | 6.47 | 8.46 | 8.5 | 8.73 | 6.01 | 8.13 | 10.82 | 7.5 | 10.39 |
| HU-15 | 6.81 | 9.6 | 7.09 | 6.54 | 7.05 | 7.6 | 7.76 | 7.88 | 5.81 | 6.23 | 7.89 | 7.42 | 5.62 | 6.88 | 7.77 | 8.69 | 8 | 7.49 |
| HU-16 | 14.99 | 14.52 | 13.45 | 11.59 | 11.88 | 11.86 | 14.54 | 11.59 | 8.24 | 9.87 | 12.26 | 11.6 | 8.36 | 8.03 | 15.41 | 13.8 | 12.85 | 12.23 |
| LGI1-17 | 5.09 | 5.3 | 5.69 | 6.36 | 5.53 | 5.74 | 6.62 | 6.85 | 4.49 | 3.98 | 5.87 | 5.38 | 4.56 | 4.69 | 5.66 | 6.03 | 6.5 | 6.13 |
| LGI1-18 | 10.09 | 9.39 | 10.15 | 9.58 | 9.33 | 9.19 | 11 | 10.28 | 7.79 | 6.45 | 10.08 | 9.01 | 7.49 | 9.56 | 10.3 | 9.48 | 10.74 | 11.02 |
| LGI1-19 | 7.99 | 7.25 | 9.18 | 8.56 | 8.23 | 8.63 | 9.18 | 8.86 | 6.78 | 7.03 | 9.52 | 9.52 | 6.94 | 6.62 | 8.1 | 8.15 | 8.24 | 8.62 |
| LGI1-20 | 9.87 | 9.6 | 11.36 | 10.86 | 10.07 | 9.44 | 12.25 | 11.86 | 6.71 | 7.08 | 10.47 | 9.96 | 7.82 | 7.62 | 14.16 | 12.09 | 11.2 | 11.7 |
| LGI1-21 | 9.66 | 9.26 | 11.46 | 12.33 | 11.09 | 11.38 | 11.67 | 12.33 | 7.8 | 8.29 | 13.66 | 12.74 | 8.36 | 8.74 | 13.07 | 12.29 | 14.3 | 13.84 |
| LGI1-22 | 10.05 | 9.35 | 10.11 | 9.54 | 9.29 | 9.15 | 10.96 | 10.24 | 7.75 | 6.41 | 10.04 | 8.97 | 7.45 | 9.52 | 10.26 | 9.44 | 10.7 | 10.98 |
| NMDAR-23 | 7.24 | 8.87 | 5.82 | 5.85 | 6.54 | 8.14 | 6.41 | 7.15 | 6.78 | 7.85 | 7.79 | 7.34 | 5.19 | 5.27 | 7.21 | 8.21 | 7.76 | 7.82 |
| NMDAR-24 | 10.86 | 11.31 | 9.51 | 8.69 | 9.79 | 4.72 | 12.28 | 10.78 | 7.14 | 7.41 | 9.99 | 9.32 | 6.75 | 6.42 | 12.87 | 12.4 | 10.33 | 9.19 |

| Region of Interest | Posterior cingulate and paracingulate gyri | Posterior cingulate and paracingulate gyri | Precentral gyrus | Precentral gyrus | Precuneus | Precuneus | Rolandic operculum | Rolandic operculum | Superior frontal gyrus, dorsolateral | Superior frontal gyrus, dorsolateral | Superior frontal gyrus, medial | Superior frontal gyrus, medial | Superior frontal gyrus, medial orbital | Superior frontal gyrus, medial orbital | Superior frontal gyrus, orbital part | Superior frontal gyrus, orbital part | Superior occipital gyrus | Superior occipital gyrus |
| --- | --- | --- | --- | --- | --- | --- | --- | --- | --- | --- | --- | --- | --- | --- | --- | --- | --- | --- |
| Side | L | R | L | R | L | R | L | R | L | R | L | R | L | R | L | R | L | R |
| GABABR-1 | 13.17 | 12.17 | 12.63 | 15.42 | 15.43 | 14.9 | 12.66 | 14.31 | 15.01 | 15.4 | 14.44 | 14.05 | 14.04 | 14.19 | 14.71 | 14.3 | 14.1 | 13.59 |
| GABABR-2 | 6.91 | 6.83 | 7.6 | 6.86 | 7.88 | 7.24 | 7.57 | 7.74 | 9.13 | 8.11 | 8.02 | 6.52 | 7.76 | 6.86 | 8.25 | 7.73 | 7.38 | 7.34 |
| GABABR-3 | 12.67 | 11.67 | 12.13 | 14.92 | 14.93 | 14.4 | 12.16 | 13.81 | 14.51 | 14.9 | 13.94 | 13.55 | 13.54 | 13.69 | 14.21 | 13.8 | 13.6 | 13.09 |
| GABABR-4 | 12.97 | 11.97 | 12.43 | 15.22 | 15.23 | 14.7 | 12.46 | 14.11 | 14.81 | 15.2 | 14.24 | 13.85 | 13.84 | 13.99 | 14.51 | 14.1 | 13.9 | 13.39 |
| GAD65-5 | 11.95 | 11.17 | 11.34 | 11.75 | 13.43 | 12.02 | 11.31 | 10.51 | 11.66 | 11.16 | 10.02 | 10.12 | 8.87 | 8.73 | 9.48 | 9.26 | 10.88 | 11.2 |
| GAD65-6 | 15.81 | 15.66 | 17.09 | 17.23 | 15.83 | 16.1 | 16.27 | 16.61 | 17.37 | 17.4 | 15.04 | 14.64 | 13.5 | 13.25 | 13.81 | 15.52 | 14.9 | 16.17 |
| GAD65-7 | 4.57 | 4.36 | 4.79 | 4.46 | 4.73 | 4.76 | 4.16 | 4.02 | 4.42 | 4.45 | 4.25 | 4.01 | 4.32 | 3.81 | 4.23 | 4 | 4.05 | 4.11 |
| PNMA2-8 | 9.35 | 9.15 | 10 | 10.32 | 8.89 | 9.91 | 10.08 | 8.74 | 9.6 | 8.9 | 8.15 | 8.98 | 7.55 | 8.51 | 8.06 | 8.55 | 11.43 | 8.07 |
| AMPHIN-9 | 12 | 13.75 | 13.51 | 15.97 | 13.12 | 15.51 | 12.1 | 13.55 | 16.49 | 15.16 | 13.27 | 13.67 | 12.88 | 13.43 | 11.9 | 14.22 | 20.49 | 13.99 |
| HU+SOX1-10 | 9.29 | 8.92 | 10.64 | 9.27 | 9.2 | 10.62 | 9.09 | 8.06 | 9.15 | 9.68 | 8.74 | 8.54 | 7.4 | 6.97 | 8.24 | 8.2 | 9.86 | 9.89 |
| HU+SOX1-11 | 7.82 | 8.1 | 11.2 | 11.27 | 10.17 | 9.8 | 9.67 | 9.4 | 10.67 | 10.14 | 9.59 | 8.58 | 8.8 | 8.4 | 10.05 | 9.65 | 9.56 | 10.16 |
| HU-12 | 9.04 | 7.6 | 10.29 | 9.79 | 9.51 | 8.51 | 8.74 | 8.9 | 9.26 | 10.38 | 8.5 | 8.28 | 6.66 | 7.35 | 8.24 | 8.56 | 7.69 | 8.34 |
| HU-13 | 9.6 | 8.55 | 9.1 | 8.7 | 8.98 | 9.61 | 7.75 | 8.04 | 8.8 | 8.39 | 7.99 | 7.65 | 8.04 | 7.68 | 8.15 | 8.15 | 8.48 | 7.85 |
| HU-14 | 6.75 | 6.89 | 7.99 | 9.56 | 8.13 | 10.82 | 7.04 | 10.09 | 7.83 | 8.97 | 11.78 | 7.76 | 7.18 | 7.69 | 8.69 | 9.23 | 8.9 | 9.64 |
| HU-15 | 7.14 | 6.19 | 8.21 | 8.82 | 7.77 | 8.69 | 6.6 | 7.32 | 8.84 | 9.2 | 7.13 | 8.62 | 6.11 | 6.61 | 7.21 | 9.49 | 7.76 | 6.76 |
| HU-16 | 15.08 | 13.26 | 13.48 | 13.91 | 15.41 | 13.8 | 10.46 | 10.38 | 14.02 | 14.23 | 12.68 | 14.87 | 11.15 | 11.92 | 12.98 | 13.62 | 12.19 | 11.1 |
| LGI1-17 | 5.01 | 4.61 | 6.36 | 6.47 | 5.66 | 5.73 | 5.29 | 5.24 | 7.65 | 6.29 | 5.71 | 6.24 | 4.15 | 4.5 | 5.56 | 4.84 | 5.48 | 6.1 |
| LGI1-18 | 10.67 | 9.41 | 11.24 | 10.82 | 10.28 | 9.06 | 10.73 | 8.95 | 10.61 | 11.26 | 10.15 | 9.86 | 8.89 | 8.75 | 9.52 | 8.83 | 9.36 | 8.91 |
| LGI1-19 | 6.37 | 6.53 | 8.75 | 8.68 | 8.1 | 8.15 | 7.92 | 7.95 | 8.52 | 8.63 | 7.87 | 8.11 | 7.45 | 7.22 | 7.51 | 7.07 | 8.96 | 7.71 |
| LGI1-20 | 8.91 | 7.82 | 11.25 | 10.43 | 14.16 | 12.09 | 9.02 | 9.15 | 11.96 | 11.32 | 10.27 | 10.15 | 9.32 | 9.48 | 10.26 | 9.38 | 10.91 | 10.26 |
| LGI1-21 | 10.88 | 8.71 | 16.32 | 14.28 | 13.07 | 12.29 | 11.91 | 11.76 | 12.18 | 14.36 | 11.51 | 11.81 | 9.54 | 9.63 | 9.05 | 9.83 | 10.33 | 11.38 |
| LGI1-22 | 10.63 | 9.37 | 11.2 | 10.78 | 10.24 | 9.02 | 10.69 | 8.91 | 10.57 | 11.22 | 10.11 | 9.82 | 8.85 | 8.71 | 9.48 | 8.79 | 9.32 | 8.87 |
| NMDAR-23 | 6.44 | 6.19 | 7.73 | 8.23 | 7.18 | 7.16 | 7.44 | 8 | 7.84 | 9.06 | 7.29 | 8.59 | 7.61 | 7.65 | 7.54 | 8.65 | 5.89 | 5.63 |
| NMDAR-24 | 12.09 | 11.83 | 11 | 8.81 | 12.87 | 12.4 | 11.48 | 7.44 | 11.03 | 11.15 | 12.71 | 12.26 | 9.67 | 10.62 | 9.98 | 10.64 | 11.42 | 9.4 |

| Region of Interest | Superior parietal gyrus | Superior parietal gyrus | Superior temporal gyrus | Superior temporal gyrus | Supplementary motor area | Supplementary motor area | Supramarginal gyrus | Supramarginal gyrus | Temporal lobe | Temporal lobe | Temporal pole: middle temporal gyrus | Temporal pole: middle temporal gyrus | Temporal pole: superior temporal gyrus | Temporal pole: superior temporal gyrus | Thalamus | Thalamus |
| --- | --- | --- | --- | --- | --- | --- | --- | --- | --- | --- | --- | --- | --- | --- | --- | --- |
| Side | L | R | L | R | L | R | L | R | L | R | L | R | L | R | L | R |
| GABABR-1 | 12.92 | 12.02 | 12.53 | 14.13 | 14.14 | 15.47 | 11.38 | 14.68 | 13.21 | 16.07 | 13.42 | 13.68 | 11.9 | 12.47 | 12.09 | 12.48 |
| GABABR-2 | 6.48 | 7.35 | 7.57 | 7.86 | 6.78 | 6.93 | 6.84 | 6.57 | 8.3 | 8.41 | 6.68 | 6.76 | 5.82 | 7.05 | 11.59 | 11.19 |
| GABABR-3 | 12.42 | 11.52 | 12.03 | 13.63 | 13.64 | 14.97 | 10.88 | 14.18 | 12.71 | 15.57 | 12.92 | 13.18 | 11.4 | 11.97 | 11.59 | 11.98 |
| GABABR-4 | 12.72 | 11.82 | 12.33 | 13.93 | 13.94 | 15.27 | 11.18 | 14.48 | 13.01 | 15.87 | 13.22 | 13.48 | 11.7 | 12.27 | 11.89 | 12.28 |
| GAD65-5 | 10.61 | 10.31 | 11.79 | 12.01 | 11.39 | 11.37 | 9.92 | 9.99 | 11.79 | 12.01 | 7.58 | 7.75 | 9.17 | 7.61 | 13.06 | 12.7 |
| GAD65-6 | 13.52 | 15.37 | 14.71 | 17.86 | 16.63 | 18.6 | 13.06 | 14.05 | 14.93 | 17.86 | 9.56 | 10.92 | 11.92 | 12.27 | 16.85 | 16.58 |
| GAD65-7 | 4.17 | 4.2 | 4.11 | 4.08 | 4.36 | 4.45 | 3.81 | 3.92 | 4.4 | 4.21 | 3.44 | 3.68 | 3.65 | 4.19 | 4.68 | 4.77 |
| PNMA2-8 | 8.13 | 7.49 | 10.28 | 9.16 | 9.54 | 9.49 | 6.9 | 7.91 | 10.28 | 9.16 | 3.88 | 8.04 | 7.66 | 7.11 | 9.8 | 11.53 |
| AMPHIN-9 | 11.36 | 12.63 | 12.1 | 14.61 | 16.41 | 15.89 | 12.73 | 11.51 | 13.13 | 16.79 | 8.49 | 10.11 | 8.47 | 9.82 | 16.97 | 19.58 |
| HU+SOX1-10 | 8.08 | 8.64 | 8.52 | 8.78 | 9.91 | 10.22 | 9.13 | 9.01 | 9.73 | 9.53 | 7.94 | 8.62 | 8.08 | 7.91 | 9.86 | 10.62 |
| HU+SOX1-11 | 10.87 | 10.71 | 11.79 | 10.86 | 11.05 | 11.16 | 10.01 | 9.46 | 11.79 | 10.92 | 7.2 | 7.99 | 8.05 | 7.77 | 10.12 | 8.82 |
| HU-12 | 7.95 | 7.68 | 8.58 | 8.84 | 9.04 | 10.35 | 7.79 | 8.52 | 8.58 | 8.84 | 6.22 | 6.79 | 6.96 | 7.26 | 9.03 | 8.87 |
| HU-13 | 7.9 | 8.28 | 7.77 | 8.12 | 8.67 | 8.18 | 8.05 | 8.08 | 8.4 | 8.47 | 6.53 | 6.85 | 7.19 | 6.86 | 8.77 | 9.69 |
| HU-14 | 7 | 7.43 | 7.58 | 8.38 | 7.8 | 8.62 | 7.5 | 8.03 | 7.76 | 8.38 | 6.16 | 6.23 | 6.14 | 6.83 | 6.65 | 8.27 |
| HU-15 | 7.01 | 6.44 | 7.49 | 8.18 | 8.87 | 8.29 | 7.23 | 8.24 | 7.49 | 8.18 | 5.55 | 5.65 | 6.11 | 6.42 | 8.81 | 10.75 |
| HU-16 | 12.18 | 12.07 | 10.24 | 11.01 | 12.6 | 13.77 | 13.19 | 12.12 | 11.89 | 11.86 | 8.47 | 9.62 | 9.95 | 8.84 | 11.98 | 12.46 |
| LGI1-17 | 5.6 | 5.49 | 6.03 | 5.24 | 6.93 | 6.81 | 5.6 | 5.26 | 6.03 | 5.74 | 4.44 | 4.69 | 4.3 | 4.9 | 6.04 | 6.3 |
| LGI1-18 | 9.63 | 8.47 | 9.4 | 9.81 | 10.81 | 9.27 | 9.35 | 8.65 | 11 | 10.26 | 8.28 | 7.32 | 7.98 | 8.1 | 12 | 11.62 |
| LGI1-19 | 7.88 | 7.99 | 7.48 | 8.19 | 8.44 | 8.71 | 6.38 | 6.82 | 8.23 | 8.63 | 6.03 | 6.65 | 5.91 | 6.26 | 8.07 | 8.69 |
| LGI1-20 | 9.76 | 9.81 | 9.58 | 9.79 | 12.49 | 11.39 | 8.84 | 8.78 | 10.07 | 9.79 | 7.05 | 7.38 | 7.14 | 6.05 | 11.1 | 12.86 |
| LGI1-21 | 11.56 | 10.19 | 13.28 | 10.85 | 14.96 | 13.24 | 9.35 | 10.09 | 13.28 | 12.78 | 8.35 | 9.58 | 8.56 | 11.27 | 12.95 | 11.23 |
| LGI1-22 | 9.59 | 8.43 | 9.36 | 9.77 | 10.77 | 9.23 | 9.31 | 8.61 | 10.96 | 10.22 | 8.24 | 7.28 | 7.94 | 8.06 | 11.96 | 11.58 |
| NMDAR-23 | 7.21 | 7.79 | 6.92 | 8.37 | 9.2 | 8.29 | 6.31 | 8.21 | 6.92 | 8.37 | 6.05 | 7.83 | 5.93 | 7.87 | 7.03 | 6.25 |
| NMDAR-24 | 9.96 | 9.71 | 10.7 | 5.95 | 10.94 | 12.22 | 10.42 | 6.89 | 10.7 | 6.27 | 8.51 | 5.66 | 9.18 | 5.21 | 11.39 | 10.38 |

Abbreviation: NMDAR: N-methyl-D-aspartate receptor; LGI1: leucine-rich glioma inactivated-1; CASPR2: contacting-associated protein-2; GABABR: gamma-aminobutyric acid receptor; AMPAR: α-amino-3-hydroxy-5-methyl-4-isoxazole propionic acid receptor; GAD65: glutamic acid decarboxylase 65, SUVmax: maximum standardized uptake value, AAL: automated anatomical labelling.

Supplementary Table 2 The SUV_mean_ of case groups according to AAL standards.

| Region of Interest | Amygdala | Amygdala | Angular gyrus | Angular gyrus | Anterior cingulate and paracingulate gyri | Anterior cingulate and paracingulate gyri | Basal ganglia | Basal ganglia | Calcarine fissure and surrounding cortex | Calcarine fissure and surrounding cortex | Caudate nucleus | Caudate nucleus | Central region | Central region | Cingulate and paracingulate gyri | Cingulate and paracingulate gyri | Cuneus | Cuneus |
| --- | --- | --- | --- | --- | --- | --- | --- | --- | --- | --- | --- | --- | --- | --- | --- | --- | --- | --- |
| Side | L | R | L | R | L | R | L | R | L | R | L | R | L | R | L | R | L | R |
| GABABR-1 | 7.58 | 7.48 | 6.5 | 5.7 | 7.92 | 8.5 | 8.51 | 7.44 | 7.8 | 8.28 | 6.97 | 6.11 | 7 | 7.27 | 8.14 | 8.45 | 7.87 | 8.48 |
| GABABR-2 | 4.97 | 4 | 3.56 | 3.64 | 3.86 | 3.97 | 4.59 | 4.55 | 4.6 | 4.87 | 4.07 | 4.02 | 3.89 | 3.97 | 4.24 | 4.19 | 4.97 | 4.87 |
| GABABR-3 | 7.08 | 6.98 | 6 | 5.2 | 7.42 | 8 | 8.01 | 6.94 | 7.3 | 7.78 | 6.47 | 5.61 | 6.5 | 6.77 | 7.64 | 7.95 | 7.37 | 7.98 |
| GABABR-4 | 7.38 | 7.28 | 6.3 | 5.5 | 7.72 | 8.3 | 8.31 | 7.24 | 7.6 | 8.08 | 6.77 | 5.91 | 6.8 | 7.07 | 7.94 | 8.25 | 7.67 | 8.28 |
| GAD65-5 | 4.51 | 4.32 | 5.76 | 6.15 | 4.81 | 4.95 | 5.69 | 5.26 | 6.92 | 8.27 | 4.41 | 3.93 | 5.74 | 5.61 | 5.62 | 5.63 | 6.83 | 7.35 |
| GAD65-6 | 6.32 | 5.91 | 7.55 | 8.06 | 6.96 | 7.33 | 8.63 | 7.91 | 9.2 | 10.69 | 7.13 | 6.14 | 8.07 | 8.39 | 7.99 | 8.18 | 8.9 | 9.32 |
| GAD65-7 | 2.32 | 2.28 | 2.79 | 2.84 | 2.65 | 2.73 | 2.88 | 2.95 | 2.89 | 3.14 | 2.43 | 2.6 | 2.7 | 2.74 | 2.91 | 3.01 | 2.89 | 3.21 |
| PNMA2-8 | 3.86 | 2.87 | 4.69 | 5.01 | 4.39 | 5.54 | 5.36 | 5.96 | 4.56 | 5.65 | 4.76 | 5.22 | 5.06 | 5.07 | 4.69 | 5.68 | 4.66 | 5.49 |
| AMPHIN-9 | 5.33 | 5.05 | 4.53 | 6.54 | 6.21 | 6.34 | 7.34 | 7.28 | 8.18 | 9.71 | 5.66 | 5.52 | 7.18 | 7.42 | 7.29 | 7.38 | 8.51 | 8.53 |
| HU+SOX1-10 | 4.92 | 4.16 | 4.41 | 4.83 | 4.61 | 4.72 | 5.32 | 5.01 | 6.22 | 6.6 | 4.55 | 4.53 | 5.22 | 5.03 | 5.18 | 5.33 | 5.58 | 5.84 |
| HU+SOX1-11 | 5.52 | 5.08 | 5.48 | 5.32 | 5.25 | 5.4 | 7.35 | 6.29 | 5.79 | 6.38 | 6.7 | 5.79 | 5.82 | 5.69 | 5.8 | 5.92 | 5.67 | 6.35 |
| HU-12 | 3.77 | 3.3 | 4.23 | 4.13 | 3.86 | 4.19 | 6.62 | 4.96 | 3.99 | 4.74 | 5.54 | 3.83 | 4.48 | 4.66 | 4.44 | 4.59 | 4.58 | 4.67 |
| HU-13 | 4.32 | 4.1 | 5.06 | 5.27 | 4.97 | 5.01 | 6.04 | 5.86 | 5.83 | 5.79 | 5.46 | 5.5 | 4.97 | 5.05 | 5.45 | 5.53 | 5.36 | 5.74 |
| HU-14 | 3.75 | 3.31 | 3.97 | 4.91 | 4.1 | 4.27 | 4.02 | 5.16 | 4.92 | 5.48 | 2.16 | 3.79 | 4.14 | 4.88 | 4.25 | 4.67 | 5.03 | 5.56 |
| HU-15 | 3.91 | 3.83 | 4.26 | 4.42 | 3.87 | 3.69 | 4.88 | 4.84 | 4.67 | 4.78 | 3.94 | 3.83 | 4.56 | 4.64 | 4.38 | 4.31 | 4.74 | 5.05 |
| HU-16 | 4.73 | 3.95 | 6.86 | 6.49 | 6.2 | 6.32 | 7.97 | 7.46 | 5.73 | 5.93 | 6.99 | 6.25 | 6.28 | 6.55 | 6.88 | 7.02 | 6.67 | 6.15 |
| LGI1-17 | 2.68 | 2.67 | 2.94 | 3.26 | 2.69 | 2.89 | 3.62 | 3.58 | 3.78 | 3.83 | 3.25 | 3.18 | 3.1 | 3.12 | 2.93 | 3.05 | 3.47 | 3.7 |
| LGI1-18 | 4.67 | 5.28 | 5.9 | 5.39 | 6.03 | 5.82 | 7.1 | 6.37 | 6.94 | 6.22 | 6.39 | 5.6 | 5.64 | 5.62 | 5.97 | 6.04 | 6.06 | 6.15 |
| LGI1-19 | 4.04 | 3.67 | 4.32 | 4.73 | 4.3 | 4.33 | 6.24 | 5.72 | 4.63 | 4.66 | 5 | 3.84 | 4.26 | 4.27 | 4.29 | 4.31 | 4.56 | 4.64 |
| LGI1-20 | 4.08 | 3.55 | 5.42 | 6.01 | 4.44 | 4.57 | 4.73 | 4.67 | 5.82 | 5.88 | 2.82 | 3.32 | 4.93 | 4.69 | 4.65 | 4.64 | 5.43 | 5.63 |
| LGI1-21 | 4.66 | 4.67 | 4.8 | 4.77 | 4.87 | 4.93 | 6.57 | 6.97 | 5.91 | 6.55 | 5.04 | 6.13 | 5.92 | 6 | 5.55 | 5.73 | 5.41 | 5.94 |
| LGI1-22 | 4.56 | 5.17 | 5.79 | 5.28 | 5.92 | 5.71 | 6.99 | 6.26 | 6.83 | 6.11 | 6.28 | 5.49 | 5.53 | 5.51 | 5.86 | 5.93 | 5.95 | 6.04 |
| NMDAR-23 | 3.9 | 3.8 | 4.01 | 3.84 | 4.84 | 5.24 | 4.77 | 4.44 | 3.68 | 3.76 | 4.28 | 3.95 | 4 | 4.68 | 4.66 | 5.08 | 3.86 | 3.99 |
| NMDAR-24 | 3.74 | 3.57 | 6.06 | 3.67 | 5.17 | 5.36 | 6.09 | 5.55 | 6.5 | 5.47 | 5.26 | 4.75 | 5.73 | 4.49 | 5.75 | 6.07 | 6.4 | 5.93 |

| Region of Interest | Frontal lobe | Frontal lobe | Fusiform gyrus | Fusiform gyrus | Gyrus rectus | Gyrus rectus | Heschl gyrus | Heschl gyrus | Hippocampus | Hippocampus | Inferior frontal gyrus, opercular part | Inferior frontal gyrus, opercular part | Inferior frontal gyrus, orbital part | Inferior frontal gyrus, orbital part | Inferior frontal gyrus, triangular part | Inferior frontal gyrus, triangular part | Inferior occipital gyrus | Inferior occipital gyrus |
| --- | --- | --- | --- | --- | --- | --- | --- | --- | --- | --- | --- | --- | --- | --- | --- | --- | --- | --- |
| Side | L | R | L | R | L | R | L | R | L | R | L | R | L | R | L | R | L | R |
| GABABR-1 | 7.46 | 7.76 | 7.42 | 8.25 | 8.97 | 9.37 | 8.69 | 9.4 | 7.02 | 10.29 | 6.7 | 8.35 | 7.94 | 7.6 | 7.02 | 8 | 7.18 | 7.28 |
| GABABR-2 | 4.02 | 3.95 | 4.62 | 4.46 | 4.15 | 4.31 | 4.71 | 4.67 | 4.17 | 3.88 | 4.29 | 4.03 | 3.79 | 3.65 | 3.86 | 3.63 | 3.99 | 3.89 |
| GABABR-3 | 6.96 | 7.26 | 6.92 | 7.75 | 8.47 | 8.87 | 8.19 | 8.9 | 6.52 | 9.79 | 6.2 | 7.85 | 7.44 | 7.1 | 6.52 | 7.5 | 6.68 | 6.78 |
| GABABR-4 | 7.26 | 7.56 | 7.22 | 8.05 | 8.77 | 9.17 | 8.49 | 9.2 | 6.82 | 10.09 | 6.5 | 8.15 | 7.74 | 7.4 | 6.82 | 7.8 | 6.98 | 7.08 |
| GAD65-5 | 5.98 | 5.91 | 5.74 | 5.63 | 5.86 | 5.87 | 7.14 | 7.23 | 4.29 | 4.11 | 5.99 | 5.84 | 5.85 | 5.63 | 5.95 | 5.99 | 5.37 | 5.61 |
| GAD65-6 | 8.4 | 8.51 | 8 | 8.04 | 8.04 | 8.13 | 10.87 | 10.61 | 5.59 | 5.69 | 8.59 | 8.67 | 8.05 | 7.97 | 8.08 | 8.46 | 7.97 | 8.44 |
| GAD65-7 | 2.76 | 2.85 | 2.76 | 2.76 | 2.75 | 2.85 | 2.83 | 3.11 | 2.51 | 2.37 | 2.73 | 2.89 | 2.59 | 2.68 | 2.66 | 2.83 | 2.72 | 2.81 |
| PNMA2-8 | 4.8 | 5.26 | 3.37 | 4.49 | 4.17 | 5.95 | 6.08 | 6.26 | 3.15 | 4.07 | 4.52 | 4.92 | 4.35 | 4.86 | 4.5 | 4.95 | 3.89 | 4.44 |
| AMPHIN-9 | 6.66 | 6.93 | 7.32 | 7.59 | 7.06 | 7.42 | 9.21 | 10.49 | 5.06 | 5.41 | 6.73 | 7.08 | 6.24 | 6.4 | 6.28 | 6.45 | 8.21 | 7.06 |
| HU+SOX1-10 | 5.2 | 5.14 | 5.23 | 5.34 | 5.29 | 5.23 | 6.55 | 6.48 | 4.1 | 4.24 | 5.15 | 5.3 | 4.98 | 4.45 | 5.13 | 5.01 | 5.86 | 5.71 |
| HU+SOX1-11 | 5.72 | 5.57 | 5.83 | 5.69 | 5.37 | 5.23 | 6.95 | 7.04 | 6.19 | 6.32 | 5.78 | 5.46 | 5.54 | 5 | 5.83 | 5.33 | 5.84 | 5.83 |
| HU-12 | 4.56 | 4.74 | 3.93 | 4.01 | 4.39 | 4.56 | 5.15 | 5.74 | 3.32 | 3.56 | 4.25 | 4.72 | 3.97 | 4.02 | 4.43 | 4.4 | 3.88 | 3.89 |
| HU-13 | 5.32 | 5.34 | 4.9 | 5.06 | 5.55 | 5.37 | 5.58 | 5.61 | 4.51 | 4.28 | 5.13 | 5.32 | 5.27 | 4.9 | 5.29 | 5.27 | 5.04 | 4.92 |
| HU-14 | 4.4 | 4.67 | 4.36 | 4.39 | 4.41 | 4.79 | 3.82 | 5.31 | 3.34 | 3.24 | 3.94 | 4.67 | 3.8 | 3.49 | 4.21 | 4.6 | 4.28 | 4.23 |
| HU-15 | 4.53 | 4.59 | 3.92 | 3.98 | 5.04 | 5.46 | 4.91 | 5 | 4.04 | 4.23 | 4.41 | 4.45 | 4.76 | 4.31 | 4.36 | 4.3 | 3.97 | 3.83 |
| HU-16 | 6.56 | 6.79 | 5.15 | 5.45 | 6.63 | 6.66 | 5.75 | 6.26 | 4.63 | 4.51 | 6.08 | 6.57 | 6.19 | 6.05 | 6.47 | 6.45 | 5.36 | 5.45 |
| LGI1-17 | 3.17 | 3.21 | 3.31 | 3.46 | 3.17 | 3.06 | 4.02 | 3.78 | 2.71 | 2.8 | 3.21 | 3.36 | 3.14 | 2.94 | 3.14 | 3.17 | 3.4 | 3.11 |
| LGI1-18 | 5.74 | 5.63 | 5.32 | 5.02 | 5.91 | 5.51 | 7.36 | 6.73 | 4.86 | 5.32 | 5.49 | 5.49 | 5.66 | 4.9 | 5.64 | 5.28 | 5.67 | 5.54 |
| LGI1-19 | 4.33 | 4.26 | 4.59 | 4.66 | 5.01 | 4.83 | 5.08 | 4.84 | 3.6 | 3.73 | 4.38 | 4.36 | 4.21 | 4.14 | 4.44 | 4.24 | 4.67 | 4.51 |
| LGI1-20 | 5.09 | 5.01 | 5.33 | 5.18 | 5.53 | 5.36 | 5.88 | 5.03 | 3.76 | 3.71 | 5.02 | 4.85 | 4.9 | 4.76 | 5.53 | 5.08 | 5.94 | 5.53 |
| LGI1-21 | 5.05 | 5.01 | 5.27 | 5.73 | 4.83 | 4.8 | 6.79 | 7.89 | 4.82 | 4.49 | 5.21 | 5.3 | 4.35 | 4.12 | 5.15 | 4.77 | 5.31 | 4.8 |
| LGI1-22 | 5.63 | 5.52 | 5.21 | 4.91 | 5.8 | 5.4 | 7.25 | 6.62 | 4.75 | 5.21 | 5.38 | 5.38 | 5.55 | 4.79 | 5.53 | 5.17 | 5.56 | 5.43 |
| NMDAR-23 | 4.36 | 4.86 | 3.51 | 3.25 | 5.15 | 5.36 | 4.71 | 5.79 | 3.47 | 3.47 | 4.19 | 4.57 | 4.35 | 4.89 | 4.03 | 4.66 | 3.29 | 3.5 |
| NMDAR-24 | 5.73 | 5.82 | 5.04 | 3.34 | 6.07 | 6.28 | 7.11 | 3.6 | 4.45 | 3.4 | 4.9 | 5 | 6.11 | 5.37 | 5.46 | 5.14 | 5.48 | 3.17 |

| Region of Interest | Inferior parietal, but supramarginal and angular gyri | Inferior parietal, but supramarginal and angular gyri | Inferior temporal gyrus | Inferior temporal gyrus | Insula | Insula | Lenticular nucleus, pallidum | Lenticular nucleus, pallidum | Lenticular nucleus, putamen | Lenticular nucleus, putamen | Lingual gyrus | Lingual gyrus | Mesial temporal lobe | Mesial temporal lobe | Middle cingulate and paracingulate gyri | Middle cingulate and paracingulate gyri | Middle frontal gyrus | Middle frontal gyrus |
| --- | --- | --- | --- | --- | --- | --- | --- | --- | --- | --- | --- | --- | --- | --- | --- | --- | --- | --- |
| Side | L | R | L | R | L | R | L | R | L | R | L | R | L | R | L | R | L | R |
| GABABR-1 | 5.86 | 6.21 | 7.54 | 7.56 | 7.83 | 8.13 | 6.93 | 5.81 | 10.43 | 9.11 | 8.13 | 6.95 | 6.8 | 8.03 | 8.37 | 8.72 | 6.89 | 7.73 |
| GABABR-2 | 3.94 | 3.94 | 4.42 | 4.31 | 4.2 | 3.84 | 3.89 | 3.72 | 5.3 | 5.27 | 4.74 | 4.44 | 4.01 | 3.71 | 4.59 | 4.41 | 4.19 | 4.19 |
| GABABR-3 | 5.36 | 5.71 | 7.04 | 7.06 | 7.33 | 7.63 | 6.43 | 5.31 | 9.93 | 8.61 | 7.63 | 6.45 | 6.3 | 7.53 | 7.87 | 8.22 | 6.39 | 7.23 |
| GABABR-4 | 5.66 | 6.01 | 7.34 | 7.36 | 7.63 | 7.93 | 6.73 | 5.61 | 10.23 | 8.91 | 7.93 | 6.75 | 6.6 | 7.83 | 8.17 | 8.52 | 6.69 | 7.53 |
| GAD65-5 | 5.99 | 6.36 | 5.83 | 5.63 | 5.66 | 5.29 | 5.12 | 4.74 | 7.08 | 6.64 | 6.66 | 6.26 | 4.52 | 4.58 | 6.18 | 6.14 | 6.47 | 6.15 |
| GAD65-6 | 8.32 | 8.6 | 7.81 | 7.89 | 8.1 | 7.99 | 7.33 | 6.51 | 10.44 | 9.93 | 9.02 | 8.2 | 6.12 | 6.39 | 8.76 | 8.9 | 9 | 9.02 |
| GAD65-7 | 2.68 | 2.95 | 2.59 | 2.74 | 2.59 | 2.65 | 2.58 | 2.44 | 3.39 | 3.42 | 2.82 | 2.77 | 2.4 | 2.36 | 3.1 | 3.2 | 2.8 | 2.98 |
| PNMA2-8 | 4.59 | 5.07 | 2.77 | 5.05 | 3.89 | 5.54 | 4.85 | 4.55 | 6.09 | 7.02 | 4.28 | 4.88 | 3.16 | 3.89 | 4.94 | 5.84 | 5.06 | 5.39 |
| AMPHIN-9 | 6.06 | 6.45 | 5.93 | 6.55 | 6.62 | 6.77 | 6.24 | 6.07 | 9.26 | 9.23 | 8.58 | 8.03 | 5.12 | 5.45 | 7.91 | 8.11 | 6.72 | 7.22 |
| HU+SOX1-10 | 4.83 | 5 | 5.18 | 5.05 | 5.27 | 5.25 | 4.48 | 4.01 | 6.3 | 5.73 | 5.77 | 5.39 | 4.36 | 4.28 | 5.5 | 5.75 | 5.47 | 5.36 |
| HU+SOX1-11 | 5.83 | 5.34 | 5.46 | 5.46 | 5.64 | 5.32 | 5.76 | 5.11 | 8.42 | 7.07 | 6.27 | 5.93 | 5.55 | 5.72 | 6.24 | 6.38 | 5.97 | 5.85 |
| HU-12 | 4.09 | 4.33 | 3.89 | 4.01 | 4.12 | 4.2 | 5.13 | 3.59 | 8.08 | 6.37 | 4.24 | 4.14 | 3.37 | 3.5 | 4.81 | 4.94 | 4.69 | 5.05 |
| HU-13 | 5.13 | 5.65 | 5.06 | 5.11 | 5.18 | 5.13 | 5.2 | 4.66 | 6.84 | 6.52 | 5.11 | 4.86 | 4.3 | 4.26 | 5.64 | 5.81 | 5.47 | 5.65 |
| HU-14 | 4.03 | 4.77 | 3.78 | 4.41 | 4.25 | 4.74 | 3.89 | 4.45 | 5.83 | 6.64 | 4.86 | 4.72 | 3.67 | 3.43 | 4.47 | 4.95 | 4.48 | 5.01 |
| HU-15 | 4.29 | 4.68 | 4.24 | 4.39 | 3.94 | 4.05 | 4.6 | 4.2 | 5.86 | 5.95 | 4.14 | 4.04 | 3.66 | 3.92 | 4.69 | 4.68 | 4.67 | 4.67 |
| HU-16 | 6.5 | 6.41 | 5.71 | 5.59 | 5.86 | 5.66 | 6.16 | 5.53 | 9.43 | 9.1 | 5.42 | 5.3 | 4.55 | 4.59 | 7.35 | 7.62 | 6.84 | 7.34 |
| LGI1-17 | 3.06 | 3.25 | 3.14 | 3.27 | 3.27 | 3.07 | 3.22 | 3.08 | 4.1 | 4.08 | 3.4 | 3.68 | 2.68 | 2.71 | 3.05 | 3.15 | 3.23 | 3.38 |
| LGI1-18 | 5.92 | 5.64 | 5.36 | 5.02 | 5.52 | 4.91 | 5.15 | 4.5 | 8.33 | 7.58 | 5.9 | 5.65 | 4.74 | 5.07 | 6 | 6.43 | 5.9 | 6.05 |
| LGI1-19 | 3.81 | 4.2 | 4.56 | 4.63 | 4.52 | 4.45 | 5 | 4.87 | 7.78 | 7.69 | 4.6 | 4.49 | 3.74 | 3.88 | 4.34 | 4.33 | 4.48 | 4.52 |
| LGI1-20 | 4.68 | 5.13 | 5.4 | 5.37 | 4.76 | 4.44 | 4.65 | 4.35 | 6.57 | 6.01 | 5.75 | 5.33 | 3.97 | 3.98 | 4.84 | 4.7 | 5.51 | 5.5 |
| LGI1-21 | 5.32 | 5.17 | 4.94 | 5.24 | 5.46 | 5.46 | 4.6 | 4.22 | 8.61 | 8.49 | 5.57 | 5.39 | 4.53 | 4.39 | 6.11 | 6.42 | 5.15 | 5.08 |
| LGI1-22 | 5.81 | 5.53 | 5.25 | 4.91 | 5.41 | 4.8 | 5.04 | 4.39 | 8.22 | 7.47 | 5.79 | 5.54 | 4.63 | 4.96 | 5.89 | 6.32 | 5.79 | 5.94 |
| NMDAR-23 | 3.99 | 4.78 | 3.74 | 3.77 | 4.44 | 5.65 | 3.6 | 3.59 | 5.58 | 5.12 | 3.51 | 3.92 | 3.52 | 3.49 | 4.77 | 5.25 | 4.17 | 5.09 |
| NMDAR-24 | 5.59 | 3.74 | 5.08 | 3.2 | 5.68 | 4.67 | 4.63 | 4.04 | 7.29 | 6.7 | 5.64 | 3.98 | 4.25 | 3.54 | 6.23 | 6.71 | 5.62 | 5.92 |

| Region of Interest | Middle frontal gyrus, orbital part | Middle frontal gyrus, orbital part | Middle occipital gyrus | Middle occipital gyrus | Middle temporal gyrus | Middle temporal gyrus | Occipital lobe | Occipital lobe | Olfactory cortex | Olfactory cortex | Paracentral lobule | Paracentral lobule | Parahippocampal gyrus | Parahippocampal gyrus | Parietal lobe | Parietal lobe | Postcentral gyrus | Postcentral gyrus |
| --- | --- | --- | --- | --- | --- | --- | --- | --- | --- | --- | --- | --- | --- | --- | --- | --- | --- | --- |
| Side | L | R | L | R | L | R | L | R | L | R | L | R | L | R | L | R | L | R |
| GABABR-1 | 9.18 | 7.8 | 6.11 | 6.67 | 7.16 | 7.53 | 7.19 | 7.56 | 6.59 | 6.87 | 7.25 | 7.31 | 6.43 | 6.26 | 6.7 | 6.63 | 6.81 | 6.69 |
| GABABR-2 | 4.88 | 4.44 | 3.67 | 3.94 | 4.25 | 4.47 | 4.34 | 4.38 | 3.29 | 2.99 | 4.07 | 3.85 | 3.65 | 3.5 | 3.9 | 3.85 | 3.68 | 3.9 |
| GABABR-3 | 8.68 | 7.3 | 5.61 | 6.17 | 6.66 | 7.03 | 6.69 | 7.06 | 6.09 | 6.37 | 6.75 | 6.81 | 5.93 | 5.76 | 6.2 | 6.13 | 6.31 | 6.19 |
| GABABR-4 | 8.98 | 7.6 | 5.91 | 6.47 | 6.96 | 7.33 | 6.99 | 7.36 | 6.39 | 6.67 | 7.05 | 7.11 | 6.23 | 6.06 | 6.5 | 6.43 | 6.61 | 6.49 |
| GAD65-5 | 6.18 | 5.79 | 5.47 | 6.03 | 5.93 | 5.84 | 6.09 | 6.43 | 5.04 | 5.14 | 6.5 | 6.34 | 4.74 | 5.04 | 6.12 | 6.11 | 5.41 | 5.26 |
| GAD65-6 | 8.74 | 8.17 | 7.62 | 8.52 | 7.76 | 7.95 | 8.31 | 8.72 | 7.17 | 7.18 | 9.13 | 9.04 | 6.58 | 7.09 | 8.2 | 8.11 | 7.52 | 7.75 |
| GAD65-7 | 2.86 | 2.9 | 2.59 | 2.81 | 2.78 | 2.88 | 2.74 | 2.87 | 2.4 | 2.3 | 2.94 | 3.07 | 2.32 | 2.37 | 2.79 | 2.83 | 2.67 | 2.71 |
| PNMA2-8 | 4.87 | 4.9 | 4.2 | 4.72 | 3.55 | 5.34 | 4.18 | 4.84 | 3.32 | 4.73 | 5.51 | 5.47 | 3 | 3.97 | 4.5 | 5.02 | 5 | 4.66 |
| AMPHIN-9 | 7.14 | 7.21 | 6.74 | 7.26 | 4.87 | 6.87 | 7.73 | 7.94 | 5.01 | 4.45 | 6.84 | 6.78 | 5.13 | 5.58 | 6.29 | 6.2 | 7.26 | 7.19 |
| HU+SOX1-10 | 5.69 | 5.44 | 4.53 | 4.94 | 5.05 | 5.06 | 5.29 | 5.49 | 4.19 | 4.51 | 4.97 | 4.87 | 4.49 | 4.34 | 4.92 | 4.99 | 4.95 | 4.65 |
| HU+SOX1-11 | 5.81 | 6 | 5.55 | 5.69 | 6.01 | 5.8 | 5.77 | 5.89 | 5.31 | 4.96 | 6.04 | 5.83 | 4.95 | 5.35 | 5.76 | 5.51 | 5.55 | 5.41 |
| HU-12 | 4.66 | 4.99 | 3.68 | 3.98 | 4.22 | 4.38 | 4 | 4.17 | 3.51 | 3.5 | 5.07 | 5.03 | 3.33 | 3.5 | 4.41 | 4.36 | 4.33 | 4.39 |
| HU-13 | 5.59 | 5.72 | 4.98 | 5.3 | 5.3 | 5.39 | 5.14 | 5.23 | 4.42 | 4.49 | 4.99 | 5.38 | 4.09 | 4.28 | 5.24 | 5.24 | 4.77 | 4.91 |
| HU-14 | 4.85 | 5.17 | 4.24 | 4.71 | 4.26 | 4.96 | 4.57 | 4.83 | 3.06 | 3.27 | 4.65 | 4.9 | 3.97 | 3.62 | 4.17 | 4.8 | 4.23 | 4.68 |
| HU-15 | 4.91 | 5.38 | 3.96 | 4.15 | 4.43 | 4.54 | 4.2 | 4.27 | 3.77 | 3.94 | 4.61 | 4.27 | 3.24 | 3.69 | 4.4 | 4.46 | 4.32 | 4.38 |
| HU-16 | 7.42 | 7.33 | 5.63 | 5.82 | 6.39 | 6.68 | 5.56 | 5.63 | 4.6 | 4.85 | 6.42 | 6.57 | 4.45 | 4.8 | 6.61 | 6.25 | 6.36 | 6.22 |
| LGI1-17 | 3.47 | 3.52 | 3.01 | 3.33 | 3.14 | 3.19 | 3.35 | 3.52 | 2.93 | 2.57 | 3.28 | 3.19 | 2.66 | 2.63 | 3.1 | 3.18 | 2.98 | 2.91 |
| LGI1-18 | 6.05 | 5.83 | 5.32 | 5.37 | 5.8 | 5.4 | 5.73 | 5.55 | 5.1 | 4.82 | 5.51 | 5.23 | 4.65 | 4.8 | 5.6 | 5.32 | 5.52 | 5.51 |
| LGI1-19 | 5 | 4.81 | 4.06 | 4.59 | 4.6 | 4.67 | 4.42 | 4.56 | 4.15 | 4 | 3.89 | 2.91 | 3.8 | 4.06 | 4.01 | 4.19 | 4.01 | 3.99 |
| LGI1-20 | 5.97 | 5.82 | 4.87 | 5.29 | 5.28 | 5.28 | 5.36 | 5.37 | 3.88 | 3.86 | 4.49 | 3.65 | 4.14 | 4.3 | 4.91 | 5.02 | 4.75 | 4.53 |
| LGI1-21 | 5.12 | 5.11 | 4.68 | 5.22 | 5.27 | 5.48 | 5.23 | 5.57 | 4.11 | 4.38 | 6.44 | 6.26 | 4.22 | 4.24 | 5.31 | 5.11 | 5.65 | 5.84 |
| LGI1-22 | 5.94 | 5.72 | 5.21 | 5.26 | 5.69 | 5.29 | 5.62 | 5.44 | 4.99 | 4.71 | 5.4 | 5.12 | 4.54 | 4.69 | 5.49 | 5.21 | 5.41 | 5.4 |
| NMDAR-23 | 4.63 | 4.98 | 3.36 | 3.67 | 3.98 | 4.52 | 3.5 | 3.64 | 4.61 | 4.58 | 4.26 | 4.18 | 3.48 | 3.44 | 4.03 | 4.28 | 3.94 | 4.51 |
| NMDAR-24 | 6.29 | 6.49 | 5.36 | 3.42 | 5.72 | 2.99 | 5.6 | 4.22 | 4.85 | 4.5 | 5.28 | 5.36 | 4.17 | 3.66 | 5.84 | 4.58 | 5.66 | 4.45 |

| Region of Interest | Posterior cingulate and paracingulate gyri | Posterior cingulate and paracingulate gyri | Precentral gyrus | Precentral gyrus | Precuneus | Precuneus | Rolandic operculum | Rolandic operculum | Superior frontal gyrus, dorsolateral | Superior frontal gyrus, dorsolateral | Superior frontal gyrus, medial | Superior frontal gyrus, medial | Superior frontal gyrus, medial orbital | Superior frontal gyrus, medial orbital | Superior frontal gyrus, orbital part | Superior frontal gyrus, orbital part | Superior occipital gyrus | Superior occipital gyrus |
| --- | --- | --- | --- | --- | --- | --- | --- | --- | --- | --- | --- | --- | --- | --- | --- | --- | --- | --- |
| Side | L | R | L | R | L | R | L | R | L | R | L | R | L | R | L | R | L | R |
| GABABR-1 | 7.81 | 6.49 | 6.84 | 7.29 | 7.91 | 7.66 | 8.32 | 8.86 | 6.79 | 6.79 | 7.76 | 7.5 | 9.11 | 8.84 | 9.1 | 8.67 | 6.14 | 6.97 |
| GABABR-2 | 3.92 | 3.65 | 4.01 | 4.01 | 4.31 | 4.13 | 4.3 | 4.03 | 3.84 | 3.68 | 3.85 | 3.93 | 4.26 | 4.25 | 4.54 | 4.31 | 3.98 | 3.96 |
| GABABR-3 | 7.31 | 5.99 | 6.34 | 6.79 | 7.41 | 7.16 | 7.82 | 8.36 | 6.29 | 6.29 | 7.26 | 7 | 8.61 | 8.34 | 8.6 | 8.17 | 5.64 | 6.47 |
| GABABR-4 | 7.61 | 6.29 | 6.64 | 7.09 | 7.71 | 7.46 | 8.12 | 8.66 | 6.59 | 6.59 | 7.56 | 7.3 | 8.91 | 8.64 | 8.9 | 8.47 | 5.94 | 6.77 |
| GAD65-5 | 5.75 | 4.91 | 5.99 | 5.87 | 7.12 | 6.92 | 6.12 | 5.95 | 5.7 | 5.7 | 5.41 | 5.8 | 6.11 | 5.93 | 5.84 | 5.5 | 5.64 | 5.97 |
| GAD65-6 | 7.85 | 6.8 | 8.52 | 8.89 | 9.28 | 9.02 | 8.65 | 8.94 | 8.14 | 8.26 | 7.64 | 8.14 | 8.45 | 8.39 | 8.47 | 8.09 | 7.52 | 8.09 |
| GAD65-7 | 2.91 | 2.79 | 2.71 | 2.76 | 3.07 | 3.13 | 2.75 | 2.81 | 2.62 | 2.71 | 2.81 | 2.79 | 2.9 | 2.83 | 2.85 | 2.91 | 2.49 | 2.67 |
| PNMA2-8 | 4.58 | 5.12 | 5.08 | 5.35 | 4.85 | 5.47 | 5.24 | 5.54 | 4.69 | 4.94 | 4.76 | 5.62 | 4.53 | 5.78 | 4.41 | 5.28 | 4.34 | 4.1 |
| AMPHIN-9 | 7.97 | 6.69 | 7.03 | 7.74 | 7.09 | 7.15 | 7.45 | 7.27 | 6.66 | 6.72 | 6.11 | 6.54 | 7.26 | 7.46 | 7.25 | 7.14 | 7.56 | 7.15 |
| HU+SOX1-10 | 5.58 | 4.92 | 5.37 | 5.34 | 5.43 | 5.59 | 5.72 | 5.3 | 4.95 | 5.03 | 4.96 | 5.14 | 5.09 | 5.15 | 5.18 | 4.98 | 4.21 | 4.75 |
| HU+SOX1-11 | 5.61 | 4.9 | 5.97 | 5.95 | 6.23 | 5.93 | 6.32 | 5.84 | 5.42 | 5.33 | 5.3 | 5.25 | 5.51 | 5.67 | 5.68 | 5.64 | 5.43 | 5.38 |
| HU-12 | 4.62 | 3.79 | 4.57 | 4.88 | 5.03 | 4.87 | 4.76 | 4.87 | 4.47 | 4.53 | 4.45 | 4.51 | 4.31 | 4.48 | 4.57 | 4.44 | 3.99 | 3.71 |
| HU-13 | 6.06 | 5.67 | 5.09 | 5.12 | 5.64 | 5.51 | 5.35 | 5.29 | 5.14 | 5.04 | 5.22 | 5.15 | 5.63 | 5.57 | 5.5 | 5.66 | 4.64 | 5.02 |
| HU-14 | 3.74 | 4.41 | 4.01 | 5.09 | 4.67 | 5.4 | 4.27 | 4.96 | 4.08 | 4.56 | 4.73 | 4.29 | 4.58 | 4.68 | 4.86 | 5.3 | 4.32 | 4.8 |
| HU-15 | 4.62 | 4.27 | 4.81 | 4.9 | 4.66 | 4.6 | 4.62 | 4.76 | 4.5 | 4.51 | 4.04 | 4.32 | 4.24 | 4.64 | 4.7 | 5.12 | 4.1 | 4.18 |
| HU-16 | 6.94 | 5.86 | 6.2 | 6.89 | 7.39 | 6.89 | 6.27 | 6.63 | 6.7 | 6.6 | 6.12 | 6.62 | 6.65 | 7.16 | 7.32 | 7.31 | 4.95 | 5.37 |
| LGI1-17 | 3.19 | 3.01 | 3.19 | 3.31 | 3.36 | 3.43 | 3.27 | 3.23 | 3 | 3.03 | 2.89 | 3.1 | 3.02 | 3.03 | 3.17 | 3.14 | 3.25 | 3.38 |
| LGI1-18 | 5.64 | 4.38 | 5.63 | 5.74 | 5.71 | 5.55 | 6.16 | 5.63 | 5.6 | 5.66 | 5.66 | 5.6 | 5.94 | 5.93 | 5.93 | 5.51 | 4.85 | 5.11 |
| LGI1-19 | 4.05 | 4.04 | 4.4 | 4.33 | 4.25 | 4.38 | 4.7 | 4.91 | 4.17 | 4.22 | 3.91 | 3.99 | 4.81 | 4.9 | 4.92 | 4.67 | 4.07 | 4.29 |
| LGI1-20 | 4.5 | 4.54 | 5.03 | 4.68 | 5.41 | 5.37 | 5.28 | 5.16 | 4.76 | 4.94 | 4.43 | 4.75 | 5.37 | 5.45 | 5.96 | 5.59 | 4.72 | 4.83 |
| LGI1-21 | 5.24 | 4.36 | 6.07 | 6.15 | 5.9 | 5.67 | 6.44 | 6.09 | 4.63 | 4.95 | 4.53 | 4.38 | 4.79 | 5.13 | 5.05 | 5.09 | 4.6 | 4.99 |
| LGI1-22 | 5.53 | 4.27 | 5.52 | 5.63 | 5.6 | 5.44 | 6.05 | 5.52 | 5.49 | 5.55 | 5.55 | 5.49 | 5.83 | 5.82 | 5.82 | 5.4 | 4.74 | 5 |
| NMDAR-23 | 3.66 | 3.32 | 3.91 | 4.58 | 4.29 | 4.09 | 4.52 | 5.44 | 4.18 | 4.63 | 4.41 | 5.15 | 5 | 5.06 | 4.6 | 5.19 | 3.29 | 3.38 |
| NMDAR-24 | 5.48 | 4.66 | 5.65 | 4.84 | 6.28 | 6.26 | 6.25 | 3.7 | 5.54 | 5.54 | 6.04 | 6.21 | 6.43 | 6.24 | 5.95 | 6.36 | 4.74 | 4.72 |

| Region of Interest | Superior parietal gyrus | Superior parietal gyrus | Superior temporal gyrus | Superior temporal gyrus | Supplementary motor area | Supplementary motor area | Supramarginal gyrus | Supramarginal gyrus | Temporal lobe | Temporal lobe | Temporal pole: middle temporal gyrus | Temporal pole: middle temporal gyrus | Temporal pole: superior temporal gyrus | Temporal pole: superior temporal gyrus | Thalamus | Thalamus |
| --- | --- | --- | --- | --- | --- | --- | --- | --- | --- | --- | --- | --- | --- | --- | --- | --- |
| Side | L | R | L | R | L | R | L | R | L | R | L | R | L | R | L | R |
| GABABR-1 | 5.63 | 5.53 | 7.57 | 8.04 | 7.6 | 8.25 | 6.87 | 7.27 | 7.39 | 7.72 | 6.33 | 6.79 | 6.15 | 6.92 | 7.69 | 6.95 |
| GABABR-2 | 3.29 | 3.63 | 4.29 | 4.2 | 3.73 | 3.91 | 4.02 | 3.75 | 4.32 | 4.35 | 3.48 | 3.49 | 3.1 | 3.49 | 5.75 | 5.15 |
| GABABR-3 | 5.13 | 5.03 | 7.07 | 7.54 | 7.1 | 7.75 | 6.37 | 6.77 | 6.89 | 7.22 | 5.83 | 6.29 | 5.65 | 6.42 | 7.19 | 6.45 |
| GABABR-4 | 5.43 | 5.33 | 7.37 | 7.84 | 7.4 | 8.05 | 6.67 | 7.07 | 7.19 | 7.52 | 6.13 | 6.59 | 5.95 | 6.72 | 7.49 | 6.75 |
| GAD65-5 | 4.95 | 4.96 | 6.05 | 5.85 | 6.08 | 6.23 | 5.8 | 5.83 | 5.95 | 5.81 | 4.83 | 4.77 | 4.51 | 4.1 | 6.19 | 6.22 |
| GAD65-6 | 6.82 | 6.5 | 8.02 | 8.05 | 8.79 | 8.95 | 7.85 | 8.14 | 7.9 | 8.02 | 6.24 | 6.48 | 5.89 | 5.76 | 8.84 | 9.05 |
| GAD65-7 | 2.46 | 2.38 | 2.74 | 2.83 | 2.91 | 2.93 | 2.79 | 2.77 | 2.71 | 2.83 | 2.34 | 2.41 | 2.08 | 2.27 | 3.03 | 3.1 |
| PNMA2-8 | 4.2 | 4.18 | 4.87 | 5.4 | 5.5 | 5.74 | 3.7 | 5.2 | 3.65 | 5.29 | 2.17 | 4.32 | 2.5 | 3.94 | 4.84 | 5.98 |
| AMPHIN-9 | 5.99 | 4.43 | 6.25 | 6.99 | 7.36 | 7.57 | 6.6 | 6.14 | 5.58 | 6.88 | 5.2 | 5.88 | 4.33 | 4.75 | 9.06 | 8.8 |
| HU+SOX1-10 | 4.3 | 4.07 | 5.58 | 5.06 | 5.66 | 5.46 | 5.19 | 5.18 | 5.23 | 5.09 | 4.13 | 4.20 | 4.21 | 4.25 | 5.71 | 5.52 |
| HU+SOX1-11 | 4.98 | 4.87 | 6.42 | 5.92 | 6.31 | 6.17 | 5.83 | 5.83 | 5.95 | 5.76 | 3.98 | 4.11 | 3.71 | 3.92 | 5.88 | 5.81 |
| HU-12 | 3.75 | 3.67 | 4.48 | 4.61 | 5.26 | 5.67 | 4.5 | 4.55 | 4.2 | 4.36 | 3.74 | 3.69 | 3.3 | 3.55 | 4.42 | 4.49 |
| HU-13 | 4.84 | 4.43 | 5.25 | 5.23 | 5.49 | 5.41 | 5.19 | 5.41 | 5.22 | 5.26 | 4.55 | 4.41 | 4.07 | 4.05 | 6.22 | 6.3 |
| HU-14 | 3.52 | 3.6 | 4.28 | 4.74 | 4.72 | 4.99 | 4.31 | 5.08 | 4.11 | 4.73 | 3.62 | 3.72 | 2.88 | 3.2 | 3.39 | 4.71 |
| HU-15 | 4.21 | 3.84 | 4.52 | 4.84 | 4.71 | 4.65 | 4.36 | 4.84 | 4.4 | 4.59 | 3.67 | 3.71 | 2.86 | 3.18 | 5.12 | 4.83 |
| HU-16 | 5.21 | 4.8 | 5.97 | 6.12 | 6.54 | 6.8 | 6.66 | 6.46 | 6.08 | 6.17 | 4.72 | 4.71 | 3.88 | 4.38 | 6.64 | 6.37 |
| LGI1-17 | 2.75 | 2.74 | 3.42 | 3.42 | 3.59 | 3.48 | 3.17 | 3.14 | 3.22 | 3.3 | 2.92 | 2.96 | 2.72 | 2.9 | 3.31 | 3.33 |
| LGI1-18 | 4.95 | 4.6 | 5.92 | 5.37 | 5.96 | 5.74 | 5.44 | 5.47 | 5.73 | 5.3 | 4.81 | 4.54 | 4.71 | 4.3 | 6.88 | 6.7 |
| LGI1-19 | 3.49 | 3.53 | 4.45 | 4.57 | 4.14 | 3.74 | 4.3 | 4.11 | 4.56 | 4.64 | 3.98 | 3.84 | 3.16 | 2.97 | 4.01 | 4.05 |
| LGI1-20 | 4.02 | 3.93 | 4.84 | 4.93 | 4.73 | 4.25 | 4.91 | 4.72 | 5.24 | 5.21 | 4.27 | 3.97 | 3.1 | 2.75 | 4.94 | 4.91 |
| LGI1-21 | 4.69 | 4.67 | 6.21 | 5.56 | 5.99 | 5.83 | 5.07 | 4.91 | 5.4 | 5.48 | 4.35 | 4.23 | 3.93 | 4.13 | 6.38 | 5.65 |
| LGI1-22 | 4.84 | 4.49 | 5.81 | 5.26 | 5.85 | 5.63 | 5.33 | 5.36 | 5.62 | 5.19 | 4.7 | 4.43 | 4.6 | 4.19 | 6.77 | 6.59 |
| NMDAR-23 | 3.69 | 3.96 | 4.38 | 5.21 | 4.76 | 4.73 | 3.98 | 5.01 | 4.01 | 4.51 | 4.09 | 4.31 | 3.79 | 4.56 | 4.28 | 4.17 |
| NMDAR-24 | 5.49 | 4.79 | 6.15 | 3.42 | 5.95 | 6.67 | 5.36 | 3 | 5.65 | 3.19 | 4.76 | 3.44 | 4.48 | 3 | 6.48 | 5.98 |

Abbreviation: NMDAR: N-methyl-D-aspartate receptor; LGI1: leucine-rich glioma inactivated-1; CASPR2: contacting-associated protein-2; GABABR: gamma-aminobutyric acid receptor; AMPAR: α-amino-3-hydroxy-5-methyl-4-isoxazole propionic acid receptor; GAD65: glutamic acid decarboxylase 65, SUVmean: mean standardized uptake value, AAL: automated anatomical labelling.

Supplementary Table 3 The SUV_maxstd_ of case groups according to AAL standards.

| Region of Interest | Amygdala | Amygdala | Angular gyrus | Angular gyrus | Anterior cingulate and paracingulate gyri | Anterior cingulate and paracingulate gyri | Basal ganglia | Basal ganglia | Calcarine fissure and surrounding cortex | Calcarine fissure and surrounding cortex | Caudate nucleus | Caudate nucleus | Central region | Central region | Cingulate and paracingulate gyri | Cingulate and paracingulate gyri | Cuneus | Cuneus |
| --- | --- | --- | --- | --- | --- | --- | --- | --- | --- | --- | --- | --- | --- | --- | --- | --- | --- | --- |
| Side | L | R | L | R | L | R | L | R | L | R | L | R | L | R | L | R | L | R |
| GABABR-1 | 6.6 | 13.8 | 0.2 | 0 | 3.6 | 5.2 | 4.9 | 5.2 | 1.9 | 3.1 | 4.6 | 3 | 2.6 | 7.1 | 7.7 | 6.5 | 2.3 | 2.8 |
| GABABR-2 | 10.7 | 7.8 | 0.9 | 1.1 | 1.5 | 2 | 6.6 | 6.4 | 3.8 | 3.2 | 2.9 | 2.8 | 3.7 | 2.8 | 3.2 | 2 | 6.3 | 3.1 |
| GABABR-3 | 6.6 | 13.8 | 0.2 | 0 | 3.6 | 5.2 | 4.9 | 5.2 | 1.9 | 3.1 | 4.6 | 3 | 2.6 | 7.1 | 7.7 | 6.5 | 2.3 | 2.8 |
| GABABR-4 | 6.6 | 13.8 | 0.2 | 0 | 3.6 | 5.2 | 4.9 | 5.2 | 1.9 | 3.1 | 4.6 | 3 | 2.6 | 7.1 | 7.7 | 6.5 | 2.3 | 2.8 |
| GAD65-5 | 3 | 2.3 | 1.4 | 1.9 | 0.1 | -0.1 | 3.4 | 2.4 | 5.3 | 6.7 | 1.6 | 0.4 | 4.6 | 3.2 | 6.1 | 6 | 4.2 | 4.5 |
| GAD65-6 | 2.5 | 1.9 | 0.3 | 0.9 | 0.3 | 0.4 | 3.3 | 3.4 | 3.5 | 4.4 | 2.1 | 1 | 6.2 | 4.8 | 6.4 | 6.1 | 3.7 | 2.8 |
| GAD65-7 | 5.9 | 5.3 | 1.8 | 4.7 | 2.8 | 3.7 | 5.3 | 6.5 | 1.8 | 2.3 | 4.1 | 3.3 | 4 | 3.9 | 5.1 | 4.3 | 2.3 | 3.2 |
| PNMA2-8 | 5.2 | 3.6 | 2.4 | 2.5 | 2.5 | 4 | 4.4 | 6.7 | 1.2 | 2.6 | 2.1 | 6.1 | 4.6 | 5.7 | 4.4 | 4.3 | 1.8 | 2.7 |
| AMPHIN-9 | 1.3 | 1.8 | -1.1 | -0.1 | 1.8 | 1.7 | 5.2 | 5.1 | 7.3 | 5.1 | 2.3 | 1.7 | 5.6 | 5.6 | 3.5 | 4.4 | 13.2 | 4.5 |
| HU+SOX1-10 | 4.9 | 2.9 | -0.3 | 0.9 | 1.3 | 1.4 | 2.8 | 2.9 | 2.9 | 4.6 | 0.7 | 0.9 | 4 | 2.3 | 3.2 | 2.8 | 3.8 | 4.3 |
| HU+SOX1-11 | 8.5 | 11 | 2.4 | 1 | 1.6 | 2.3 | 8.7 | 5.3 | 1 | 1.4 | 8.1 | 4.5 | 4 | 2.6 | 5.9 | 4.3 | 2 | 2.3 |
| HU-12 | 6.6 | 1.7 | 0.6 | 0.6 | 3 | 1.6 | 21.2 | 5.9 | 0.8 | 0.7 | 21.2 | 3.1 | 4.9 | 4.9 | 6.9 | 5.9 | 2.3 | 1.6 |
| HU-13 | 5.4 | 4.5 | 1.4 | 2.1 | 2.4 | 2.9 | 5.8 | 5.9 | 2.9 | 1.7 | 3.7 | 3.2 | 3.3 | 3 | 4.5 | 3.2 | 2.3 | 2.6 |
| HU-14 | 3.9 | 2.9 | -1 | 2.5 | 6.3 | 3.1 | 4.4 | 8.8 | 2.4 | 2.9 | 2.2 | 3.1 | 2.6 | 5.8 | 6.3 | 4.3 | 3.2 | 6.2 |
| HU-15 | 6.6 | 9.7 | 1.6 | 4.7 | 5.4 | 4.5 | 7.6 | 8.8 | 1.6 | 1.9 | 4.7 | 5 | 5.4 | 5 | 5.4 | 4.5 | 3.4 | 3.1 |
| HU-16 | 2 | 1.2 | 2.6 | 1.4 | 2 | 3.5 | 8.8 | 8.8 | 2.7 | 0.1 | 3.9 | 3.3 | 4.2 | 5 | 4.4 | 3.5 | 4.5 | 1.3 |
| LGI1-17 | 6.2 | 6.2 | 1.3 | 3 | 1.5 | 2.2 | 6.4 | 5.3 | 3 | 4 | 6.3 | 4.3 | 4.1 | 5.9 | 2.7 | 2.4 | 3.7 | 3.8 |
| LGI1-18 | 4.8 | 8.6 | 1.2 | 0.7 | 3.8 | 3.5 | 6 | 5.3 | 3.5 | 3.2 | 4.4 | 4.1 | 4.3 | 4 | 3.8 | 4.5 | 3.1 | 3.3 |
| LGI1-19 | 8.8 | 8.9 | 3.1 | 4.9 | 6.6 | 5.9 | 16.6 | 22.2 | 1.9 | 2 | 16.1 | 9.2 | 4.3 | 4.3 | 6.6 | 5.9 | 2.8 | 2.2 |
| LGI1-20 | 3 | 1.5 | 4.3 | 5.9 | 3.2 | 3.3 | 5 | 3.6 | 3 | 3.5 | 0.6 | 1 | 3.8 | 3 | 3.7 | 3.3 | 3.5 | 3.3 |
| LGI1-21 | 5.6 | 3.5 | 0.5 | 1.9 | 1.3 | 1.5 | 8.7 | 10.5 | 2.1 | 3.4 | 4.5 | 4.6 | 6.7 | 6.5 | 7 | 5.5 | 2.4 | 3.1 |
| LGI1-22 | 6.1 | 6.1 | 1.2 | 2.9 | 1.4 | 2.1 | 6.3 | 5.2 | 2.9 | 3.9 | 6.2 | 4.2 | 4 | 5.8 | 2.6 | 2.3 | 3.6 | 3.7 |
| NMDAR-23 | 5.2 | 5.8 | 1.5 | 0.4 | 4.9 | 6.6 | 4.5 | 4.7 | 0.3 | 0.8 | 4.5 | 4.7 | 3.8 | 7.7 | 5.5 | 6.6 | 1.2 | 0.8 |
| NMDAR-24 | 3.2 | 1.3 | 2.3 | -0.6 | 1.6 | 2 | 3.5 | 3.7 | 2.6 | 0.9 | 2.2 | 2 | 4.8 | 2.6 | 5.9 | 5 | 2.7 | 2 |

| Region of Interest | Frontal lobe | Frontal lobe | Fusiform gyrus | Fusiform gyrus | Gyrus rectus | Gyrus rectus | Heschl gyrus | Heschl gyrus | Hippocampus | Hippocampus | Inferior frontal gyrus, opercular part | Inferior frontal gyrus, opercular part | Inferior frontal gyrus, orbital part | Inferior frontal gyrus, orbital part | Inferior frontal gyrus, triangular part | Inferior frontal gyrus, triangular part | Inferior occipital gyrus | Inferior occipital gyrus |
| --- | --- | --- | --- | --- | --- | --- | --- | --- | --- | --- | --- | --- | --- | --- | --- | --- | --- | --- |
| Side | L | R | L | R | L | R | L | R | L | R | L | R | L | R | L | R | L | R |
| GABABR-1 | 7.5 | 7.1 | 6.8 | 10.8 | 5.6 | 4.6 | 0.5 | 2.5 | 7.6 | 14.7 | 2.7 | 7.1 | 4.8 | 4 | 0.6 | 5.8 | 1.2 | 4.2 |
| GABABR-2 | 5.3 | 3.4 | 9 | 4.9 | 2.9 | 1.6 | 2 | 2.1 | 9.8 | 7.5 | 3.2 | 1.9 | 3.6 | 1.4 | 2 | 1.2 | 3.6 | 4.1 |
| GABABR-3 | 7.5 | 7.1 | 6.8 | 10.8 | 5.6 | 4.6 | 0.5 | 2.5 | 7.6 | 14.7 | 2.7 | 7.1 | 4.8 | 4 | 0.6 | 5.8 | 1.2 | 4.2 |
| GABABR-4 | 7.5 | 7.1 | 6.8 | 10.8 | 5.6 | 4.6 | 0.5 | 2.5 | 7.6 | 14.7 | 2.7 | 7.1 | 4.8 | 4 | 0.6 | 5.8 | 1.2 | 4.2 |
| GAD65-5 | 7.1 | 5.7 | 3.2 | 2.3 | 1.5 | 0.6 | 1.1 | 1.1 | 5.7 | 3.5 | 1.7 | 0.3 | 2.6 | 2 | 2 | 0.8 | 0.8 | 1.6 |
| GAD65-6 | 7.3 | 6 | 3.3 | 2.2 | 1.6 | 0.6 | 2.1 | 2.3 | 3.5 | 3.5 | 1.6 | 1 | 1.7 | 1.7 | 1.8 | 1.5 | 1.1 | 2.8 |
| GAD65-7 | 4.7 | 5 | 3.9 | 3.1 | 3.2 | 2.3 | 2.6 | 3 | 6.1 | 5.6 | 2 | 3.2 | 2.2 | 2.5 | 2 | 1.7 | 1.9 | 3.3 |
| PNMA2-8 | 4.4 | 5.8 | 0.4 | 3.3 | 0.6 | 5.8 | 1.8 | 2.6 | 5.2 | 4.7 | 1.3 | 2.8 | 1.4 | 3.5 | 1.2 | 1.3 | -0.2 | 1.6 |
| AMPHIN-9 | 4.1 | 5.2 | 5.5 | 4.7 | 1.6 | 1.3 | 2.9 | 2.9 | 5 | 4.4 | 1.4 | 2.7 | 1.7 | 0.7 | 1.2 | 2.3 | 8.2 | 5 |
| HU+SOX1-10 | 3.3 | 3 | 4.9 | 7 | 1.2 | 1.4 | 2.8 | 2.5 | 4.7 | 4.4 | 1.9 | 2 | 1.6 | 1.8 | 1.1 | 0.5 | 5.4 | 7.7 |
| HU+SOX1-11 | 6.7 | 4.1 | 6 | 4.2 | 4.2 | 1.3 | 2.4 | 2 | 14.6 | 12.7 | 2.8 | 1.5 | 2.5 | 1.9 | 1.3 | 0.8 | 2.6 | 3 |
| HU-12 | 10 | 6 | 1.5 | 1.9 | 10 | 1.9 | 1 | 2.4 | 3.4 | 2.2 | 0.7 | 2 | 3.7 | 1.5 | 0.2 | 2.2 | 1.3 | 1.4 |
| HU-13 | 4.2 | 4.2 | 3.7 | 3.2 | 2.5 | 2.2 | 2.2 | 2.1 | 5.3 | 4.7 | 1.8 | 2.4 | 2.1 | 1.8 | 1.6 | 1.6 | 1.6 | 1.4 |
| HU-14 | 7.2 | 4.5 | 2.9 | 2.5 | 3.4 | 2.8 | 2.2 | 1.9 | 4.4 | 1.9 | 2.7 | 2.6 | 2.5 | 3.2 | 1.2 | 2.2 | 2.3 | 2.8 |
| HU-15 | 5.7 | 6.2 | 6 | 5.3 | 3.8 | 3.6 | 2.1 | 3 | 6.7 | 10.9 | 3.7 | 2.6 | 3.4 | 4.9 | 2.2 | 1.5 | 2.5 | 1.4 |
| HU-16 | 3.6 | 3.8 | 0.1 | 0.3 | 1.6 | 1.2 | 0.6 | 0.8 | 2.1 | 1.2 | 2 | 2.7 | 2.2 | 2.1 | 3 | 1.4 | -0.1 | -0.2 |
| LGI1-17 | 6 | 3.6 | 4.4 | 4.2 | 5 | 2 | 4 | 6.1 | 6.2 | 6.5 | 3.9 | 3.4 | 4.3 | 2.9 | 2.7 | 2.8 | 3.8 | 3.7 |
| LGI1-18 | 4.8 | 3.7 | 5 | 3.1 | 4.7 | 2.4 | 2.5 | 2.4 | 4.8 | 10.1 | 1.7 | 0.9 | 2.6 | 1.7 | 1.4 | 2.1 | 2.3 | 2.4 |
| LGI1-19 | 9.9 | 7.5 | 4.8 | 5 | 9.9 | 7.5 | 0.9 | 1.3 | 8.4 | 6.6 | 3.1 | 4.1 | 4.2 | 2.9 | 2.1 | 1.9 | 3.9 | 3.7 |
| LGI1-20 | 4.3 | 4.5 | 4.8 | 4.1 | 1.5 | 2.1 | 0.6 | -0.1 | 4.3 | 3.3 | 2.3 | 2.1 | 2.5 | 3 | 2.3 | 2.6 | 5.9 | 5.2 |
| LGI1-21 | 6.1 | 5.6 | 3.6 | 4.2 | 1.7 | 3.6 | 3.4 | 3.7 | 5.8 | 3.3 | 2.9 | 3.4 | 0.2 | -0.3 | 1 | 1 | 0.5 | 1.3 |
| LGI1-22 | 5.9 | 3.5 | 4.3 | 4.1 | 4.9 | 1.9 | 3.9 | 6 | 6.1 | 6.4 | 3.8 | 3.3 | 4.2 | 2.8 | 2.6 | 2.7 | 3.7 | 3.6 |
| NMDAR-23 | 5.5 | 9.7 | 2.6 | 2.2 | 5.5 | 9.4 | 1.2 | 7.5 | 5.1 | 4.8 | 2.2 | 6 | 3.5 | 9.3 | 0.2 | 5 | 0.3 | 0.6 |
| NMDAR-24 | 6.5 | 5.2 | 3.3 | -0.5 | 3.3 | 3.9 | 2.3 | -1.4 | 3.1 | 0.7 | 0.7 | -0.3 | 3.8 | 3 | 0.9 | 0.5 | 1.9 | -1.6 |

| Region of Interest | Inferior parietal, but supramarginal and angular gyri | Inferior parietal, but supramarginal and angular gyri | Inferior temporal gyrus | Inferior temporal gyrus | Insula | Insula | Lenticular nucleus, pallidum | Lenticular nucleus, pallidum | Lenticular nucleus, putamen | Lenticular nucleus, putamen | Lingual gyrus | Lingual gyrus | Mesial temporal lobe | Mesial temporal lobe | Middle cingulate and paracingulate gyri | Middle cingulate and paracingulate gyri | Middle frontal gyrus | Middle frontal gyrus |
| --- | --- | --- | --- | --- | --- | --- | --- | --- | --- | --- | --- | --- | --- | --- | --- | --- | --- | --- |
| Side | L | R | L | R | L | R | L | R | L | R | L | R | L | R | L | R | L | R |
| GABABR-1 | -0.3 | -0.7 | 6 | 5.2 | 4.6 | 7.3 | 4.1 | 4.2 | 4.9 | 5.2 | 5.5 | 4 | 7.6 | 14.7 | 7.7 | 6.5 | 2.1 | 1.1 |
| GABABR-2 | 2.8 | 3.1 | 5.4 | 3.9 | 4.6 | 3.8 | 6.6 | 6.2 | 6.4 | 6.4 | 6.3 | 4.5 | 10.7 | 7.8 | 3.2 | 1.9 | 3.8 | 2.8 |
| GABABR-3 | -0.3 | -0.7 | 6 | 5.2 | 4.6 | 7.3 | 4.1 | 4.2 | 4.9 | 5.2 | 5.5 | 4 | 7.6 | 14.7 | 7.7 | 6.5 | 2.1 | 1.1 |
| GABABR-4 | -0.3 | -0.7 | 6 | 5.2 | 4.6 | 7.3 | 4.1 | 4.2 | 4.9 | 5.2 | 5.5 | 4 | 7.6 | 14.7 | 7.7 | 6.5 | 2.1 | 1.1 |
| GAD65-5 | 1.8 | 2.3 | 3 | 1.5 | 3.2 | 1.6 | 3.2 | 2.4 | 3.4 | 2.1 | 5.8 | 6.9 | 5.7 | 3.5 | 6.1 | 6 | 2.3 | 1.6 |
| GAD65-6 | 2.6 | 2 | 2.4 | 1.2 | 3.6 | 2.5 | 3.3 | 2.6 | 2.9 | 3.4 | 3.8 | 4.3 | 3.5 | 3.5 | 6.4 | 6.1 | 3 | 2.9 |
| GAD65-7 | 2.8 | 2.7 | 3.2 | 3.8 | 3.2 | 4.8 | 5.3 | 6.5 | 5 | 6.5 | 3.7 | 3.6 | 6.1 | 5.6 | 5.1 | 4.3 | 3.7 | 2.7 |
| PNMA2-8 | 1.2 | 2 | 0.7 | 3.7 | 0.8 | 5.7 | 4.4 | 6.4 | 4 | 6.7 | 1.1 | 4.5 | 5.2 | 4.7 | 4.4 | 4.3 | 2.3 | 2.9 |
| AMPHIN-9 | 2.3 | 0.1 | 2.9 | 3.4 | 2.1 | 3.4 | 4.2 | 4.7 | 5.2 | 5.1 | 8.1 | 5.7 | 5.4 | 4.4 | 3.5 | 4.4 | 1.9 | 3.4 |
| HU+SOX1-10 | 1.8 | 0.3 | 3.9 | 3.8 | 3.4 | 3.1 | 2.5 | 1.7 | 2.8 | 2.9 | 4.9 | 6.1 | 5.2 | 5.2 | 3.2 | 2.8 | 3.3 | 2.4 |
| HU+SOX1-11 | 3.2 | 1.9 | 4 | 2.8 | 3.7 | 3.8 | 8.6 | 5.3 | 8.7 | 5.1 | 8.8 | 3.8 | 14.6 | 12.7 | 5.9 | 4.3 | 3.9 | 1.6 |
| HU-12 | 1.3 | 0.7 | 0.9 | 1.8 | 11.2 | 4.7 | 15.8 | 4.3 | 20.8 | 5.9 | 1.6 | 1.4 | 6.6 | 2.2 | 6.9 | 5.9 | 2.4 | 2.4 |
| HU-13 | 1.7 | 1.1 | 3.5 | 2.3 | 3.3 | 4.8 | 5.8 | 5.4 | 5.8 | 5.9 | 4.1 | 4.2 | 5.4 | 4.7 | 4.5 | 2.8 | 2.8 | 2.5 |
| HU-14 | 0.3 | 1.4 | 0.7 | 2.7 | 5.2 | 5.6 | 4.2 | 7.5 | 4.4 | 8.8 | 3.4 | 3.8 | 4.4 | 2.9 | 4.3 | 4.3 | 2.4 | 3.7 |
| HU-15 | 3 | 3.6 | 4.3 | 3.8 | 4.7 | 5.2 | 7.6 | 8.8 | 5.6 | 7.8 | 2.6 | 3.7 | 6.7 | 10.9 | 4.2 | 4.2 | 3.1 | 4 |
| HU-16 | 2.9 | 1.5 | 0.9 | 1.1 | 3.4 | 4.1 | 8.6 | 7.8 | 8.8 | 8.8 | 0.2 | 0.3 | 2.1 | 1.2 | 4.4 | 3.5 | 3.6 | 2.4 |
| LGI1-17 | 2.2 | 2.9 | 3.8 | 4.2 | 4.8 | 5.6 | 6.4 | 5.3 | 6.2 | 4.9 | 4.7 | 4.7 | 6.2 | 6.5 | 2.7 | 2.4 | 3.5 | 2.7 |
| LGI1-18 | 2 | 1.9 | 4.6 | 2.8 | 4.3 | 4.6 | 4.1 | 4.1 | 6 | 5.3 | 4.2 | 2.6 | 4.9 | 10.1 | 3.5 | 4.5 | 3.2 | 3.4 |
| LGI1-19 | 2.7 | 1.5 | 3.3 | 3.7 | 9.9 | 11.4 | 15.1 | 15.1 | 16.6 | 22.2 | 5.3 | 3.6 | 8.8 | 8.9 | 4.1 | 4.4 | 3.9 | 3.7 |
| LGI1-20 | 3 | 1.6 | 3.6 | 3.6 | 2.9 | 3.3 | 5 | 3.6 | 4.6 | 3.5 | 5.2 | 3.7 | 4.9 | 3.4 | 3.7 | 2.5 | 4.3 | 3.8 |
| LGI1-21 | 4.8 | 2 | 3 | 1.8 | 5.7 | 7.4 | 6.9 | 6.1 | 8.7 | 10.5 | 5.2 | 4.2 | 5.8 | 4.1 | 7 | 5.5 | 3.1 | 2.2 |
| LGI1-22 | 2.1 | 2.8 | 3.7 | 4.1 | 4.7 | 5.5 | 6.3 | 5.2 | 6.1 | 4.8 | 4.6 | 4.6 | 6.1 | 6.4 | 2.6 | 2.3 | 3.4 | 2.6 |
| NMDAR-23 | 0.7 | 3.1 | 2.5 | 4.4 | 2.9 | 10 | 2.1 | 2.8 | 3.3 | 4.5 | 1.1 | 2.2 | 5.2 | 5.8 | 5.5 | 4.9 | 2.5 | 7.1 |
| NMDAR-24 | 1.1 | -2.1 | 3.7 | -0.4 | 4 | 3.1 | 2.2 | 1.6 | 3.5 | 3.7 | 2.3 | 0.5 | 3.2 | 1.3 | 5.9 | 5 | 3.5 | 3 |

| Region of Interest | Middle frontal gyrus, orbital part | Middle frontal gyrus, orbital part | Middle occipital gyrus | Middle occipital gyrus | Middle temporal gyrus | Middle temporal gyrus | Occipital lobe | Occipital lobe | Olfactory cortex | Olfactory cortex | Paracentral lobule | Paracentral lobule | Parahippocampal gyrus | Parahippocampal gyrus | Parietal lobe | Parietal lobe | Postcentral gyrus | Postcentral gyrus |
| --- | --- | --- | --- | --- | --- | --- | --- | --- | --- | --- | --- | --- | --- | --- | --- | --- | --- | --- |
| Side | L | R | L | R | L | R | L | R | L | R | L | R | L | R | L | R | L | R |
| GABABR-1 | 3.2 | 1.7 | 0.9 | 2.6 | 4.7 | 5.1 | 6.8 | 10.8 | 5 | 4.1 | 4.6 | 3.1 | 7.5 | 12.2 | 4.3 | 3.3 | 2.3 | 3 |
| GABABR-2 | 3.5 | 3.4 | 2 | 1.6 | 4 | 4.4 | 9 | 4.9 | 5.3 | 2.3 | 2.6 | 1.4 | 7.9 | 4.4 | 4.5 | 4.4 | 3.7 | 2.8 |
| GABABR-3 | 3.2 | 1.7 | 0.9 | 2.6 | 4.7 | 5.1 | 6.8 | 10.8 | 5 | 4.1 | 4.6 | 3.1 | 7.5 | 12.2 | 4.3 | 3.3 | 2.3 | 3 |
| GABABR-4 | 3.2 | 1.7 | 0.9 | 2.6 | 4.7 | 5.1 | 6.8 | 10.8 | 5 | 4.1 | 4.6 | 3.1 | 7.5 | 12.2 | 4.3 | 3.3 | 2.3 | 3 |
| GAD65-5 | 2.2 | 2.2 | 2.1 | 2.5 | 1.8 | 1.7 | 5.8 | 6.9 | 2.2 | 1.9 | 7.1 | 5.7 | 4.4 | 3.1 | 6 | 5.3 | 2.8 | 3.2 |
| GAD65-6 | 1.5 | 1.5 | 2.2 | 3 | 0.5 | 0.4 | 3.8 | 4.4 | 2.3 | 1.7 | 7.3 | 5.8 | 3.5 | 3 | 5.7 | 5.4 | 3.9 | 4.2 |
| GAD65-7 | 3.5 | 3.7 | 4.2 | 3 | 2.9 | 5.4 | 4.2 | 3.6 | 3.9 | 2.5 | 4.7 | 4 | 5.2 | 3.9 | 5.2 | 4.7 | 3.6 | 3.9 |
| PNMA2-8 | 1.3 | 1.1 | 2.3 | 1.5 | 1.2 | 4.6 | 2.5 | 4.5 | 2.5 | 5.4 | 4.2 | 3 | 4.4 | 4.6 | 4.1 | 4.4 | 4.4 | 4.5 |
| AMPHIN-9 | 1.6 | 2.7 | 11.4 | 5.1 | 0.7 | 3.6 | 13.4 | 5.7 | 1.3 | 0.9 | 2.8 | 2.8 | 5.4 | 3.8 | 4.5 | 4 | 5.6 | 3.2 |
| HU+SOX1-10 | 1.7 | 2 | 4.5 | 6.9 | 3 | 1.7 | 5.4 | 7.7 | 2.8 | 1.3 | 2.5 | 1.5 | 5.2 | 5.2 | 3.5 | 4.3 | 3.3 | 2.3 |
| HU+SOX1-11 | 2.1 | 2.1 | 3.2 | 2 | 3.9 | 2.5 | 8.8 | 4.2 | 6.7 | 3.7 | 5 | 3.3 | 12.7 | 11.9 | 5 | 3.4 | 3.9 | 2.6 |
| HU-12 | 1.2 | 1.7 | 1.1 | 1.1 | 1.9 | 1.3 | 3.2 | 1.9 | 8.1 | 1.8 | 7 | 5 | 2 | 2.2 | 5.4 | 4.7 | 4.9 | 4.9 |
| HU-13 | 1.8 | 2.5 | 1.9 | 2 | 1.8 | 3.1 | 4.1 | 4.2 | 3.3 | 3 | 2.6 | 2.4 | 4.2 | 3.6 | 3.9 | 3.2 | 3.2 | 2.7 |
| HU-14 | 2.3 | 4.1 | 3.3 | 2.8 | 1.9 | 3.6 | 3.4 | 6.2 | 3.2 | 1.5 | 3.3 | 2.8 | 4 | 2.3 | 3.1 | 5.8 | 2.6 | 5.8 |
| HU-15 | 3.3 | 5.6 | 2.8 | 1.5 | 3.8 | 5 | 6 | 5.3 | 3.8 | 3.5 | 3.5 | 2.7 | 4.5 | 6.6 | 4.3 | 6.6 | 4.4 | 5 |
| HU-16 | 2.2 | 2.2 | 2.6 | 1.2 | 2 | 2.4 | 4.5 | 1.3 | 1 | 1.4 | 3.1 | 3.3 | 0.8 | 0.5 | 4.3 | 3.4 | 4.2 | 5 |
| LGI1-17 | 3.6 | 3.6 | 3.7 | 4.7 | 4 | 3.3 | 5.1 | 5 | 6 | 2.7 | 2.8 | 3 | 4.9 | 4.5 | 4 | 5.6 | 3.8 | 3.8 |
| LGI1-18 | 2.4 | 3 | 1.8 | 1.7 | 4.1 | 2.5 | 5 | 3.3 | 4.8 | 3.7 | 3.6 | 2.4 | 4.9 | 7.7 | 3.3 | 2.8 | 4 | 3.2 |
| LGI1-19 | 2.3 | 2.6 | 4.1 | 4.2 | 3.4 | 5.7 | 5.3 | 5 | 9 | 7 | 3.1 | 4.2 | 6.5 | 5.3 | 3.5 | 4.9 | 3.2 | 4 |
| LGI1-20 | 3.6 | 3.7 | 5.7 | 4.5 | 3.9 | 6.7 | 5.9 | 5.2 | 1.2 | 2.9 | 2.4 | 3 | 4.9 | 3.4 | 4.5 | 5.9 | 3.4 | 2.5 |
| LGI1-21 | 0.9 | 0.7 | 1.6 | 2.6 | 3.7 | 3.5 | 5.2 | 4.2 | 2.8 | 2.6 | 6.1 | 5.6 | 5.2 | 4.1 | 6.1 | 4.8 | 5.9 | 6.5 |
| LGI1-22 | 3.5 | 3.5 | 3.6 | 4.6 | 3.9 | 3.2 | 5 | 4.9 | 5.9 | 2.6 | 2.7 | 2.9 | 4.8 | 4.4 | 3.9 | 5.5 | 3.7 | 3.7 |
| NMDAR-23 | 1.8 | 3.4 | 1.3 | 1 | 3.3 | 8.7 | 2.6 | 2.2 | 5.5 | 8.5 | 3.9 | 3.5 | 4.1 | 5 | 3.8 | 5.3 | 3.8 | 6.3 |
| NMDAR-24 | 1.8 | 3.5 | 2.2 | -0.5 | 4.1 | -2.2 | 3.3 | 2 | 3.8 | 1.9 | 4.2 | 4.2 | 3.2 | 0.1 | 4.1 | 4.6 | 4.8 | 2.6 |

| Region of Interest | Posterior cingulate and paracingulate gyri | Posterior cingulate and paracingulate gyri | Precentral gyrus | Precentral gyrus | Precuneus | Precuneus | Rolandic operculum | Rolandic operculum | Superior frontal gyrus, dorsolateral | Superior frontal gyrus, dorsolateral | Superior frontal gyrus, medial | Superior frontal gyrus, medial | Superior frontal gyrus, medial orbital | Superior frontal gyrus, medial orbital | Superior frontal gyrus, orbital part | Superior frontal gyrus, orbital part | Superior occipital gyrus | Superior occipital gyrus |
| --- | --- | --- | --- | --- | --- | --- | --- | --- | --- | --- | --- | --- | --- | --- | --- | --- | --- | --- |
| Side | L | R | L | R | L | R | L | R | L | R | L | R | L | R | L | R | L | R |
| GABABR-1 | 1.5 | -0.2 | 2.6 | 2.5 | 4.3 | 3.1 | 2.2 | 7.1 | 2.6 | 3.7 | 3.6 | 5 | 3.7 | 5.2 | 5.4 | 4.1 | 1.2 | 2.8 |
| GABABR-2 | 2.2 | 1.6 | 3.7 | 2.1 | 4.5 | 2.6 | 2.8 | 2.4 | 2.7 | 1.3 | 2.6 | 1.1 | 0.7 | 1.5 | 2.8 | 2.6 | 4 | 3.2 |
| GABABR-3 | 1.5 | -0.2 | 2.6 | 2.5 | 4.3 | 3.1 | 2.2 | 7.1 | 2.6 | 3.7 | 3.6 | 5 | 3.7 | 5.2 | 5.4 | 4.1 | 1.2 | 2.8 |
| GABABR-4 | 1.5 | -0.2 | 2.6 | 2.5 | 4.3 | 3.1 | 2.2 | 7.1 | 2.6 | 3.7 | 3.6 | 5 | 3.7 | 5.2 | 5.4 | 4.1 | 1.2 | 2.8 |
| GAD65-5 | 1.3 | 1.5 | 4.6 | 1.9 | 6 | 5.3 | 2.4 | 1.3 | 2.2 | 4.1 | 0.6 | 1.4 | 0.5 | 0 | 1.7 | 0.9 | 3.6 | 4.4 |
| GAD65-6 | 0.7 | 0.4 | 6.2 | 4.8 | 5.7 | 5.4 | 3 | 2.3 | 2.8 | 6 | 1.9 | 2.1 | 0 | 0.3 | 1.7 | 0.7 | 2 | 3.4 |
| GAD65-7 | 2 | 2.8 | 4 | 3.2 | 4.6 | 3.5 | 2 | 3.2 | 3.5 | 5 | 3 | 3.3 | 1.7 | 2.7 | 2.9 | 2.8 | 1.9 | 3.2 |
| PNMA2-8 | 0.6 | 3.7 | 4.6 | 5.7 | 4.1 | 4.4 | 3.1 | 3.3 | 3.3 | 3 | 2.6 | 2.8 | 1.1 | 3.3 | 0.3 | 4 | 2.5 | 2.6 |
| AMPHIN-9 | 1.6 | 0.4 | 5.4 | 5.6 | 4.5 | 4 | 2.2 | 1.8 | 3.9 | 5.2 | 2.8 | 2.6 | 1 | 1.9 | 1.4 | 1.6 | 13.4 | 4.7 |
| HU+SOX1-10 | 2 | 2.3 | 4 | 1.8 | 3.5 | 4.3 | 3.3 | 2.1 | 3.1 | 2.2 | 2.1 | 2.3 | 0.3 | 0.3 | 1.6 | 1.2 | 1.7 | 3.7 |
| HU+SOX1-11 | 1.6 | 1.3 | 4 | 2.4 | 5 | 3.4 | 3.2 | 1.6 | 4.5 | 2.8 | 1.4 | 1.2 | -0.4 | 1.6 | 3.4 | 1.4 | 2.5 | 2.4 |
| HU-12 | 1.4 | -0.1 | 4.5 | 4.8 | 5.4 | 4.7 | 2 | 2.9 | 4.1 | 4.4 | 1.8 | 1.8 | -0.5 | 0.4 | 7.4 | 0.5 | 3.2 | 1.3 |
| HU-13 | 3.7 | 3.2 | 3.3 | 3 | 3.7 | 3.2 | 1.8 | 2.5 | 2.9 | 4.2 | 2 | 1.9 | 1.8 | 1.5 | 2.5 | 1.8 | 2 | 2.4 |
| HU-14 | 0.4 | 2.4 | 2.5 | 5.8 | 3.1 | 5.8 | 2.5 | 5 | 5.2 | 4.5 | 7.2 | 3.9 | 0.8 | 3.2 | 3.4 | 4.1 | 3.3 | 5.9 |
| HU-15 | 2.7 | 3.4 | 5.4 | 4.5 | 3 | 3 | 3.9 | 4.7 | 5.7 | 6.2 | 3.8 | 3.6 | 4.7 | 3.8 | 3.5 | 5.3 | 3.4 | 2.9 |
| HU-16 | 2.5 | 1.4 | 3.1 | 4.3 | 4.3 | 3.4 | 1.5 | 2.6 | 2.7 | 3.7 | 2.9 | 3.8 | 1.1 | 3.1 | 2.9 | 3.3 | 2.9 | 1.1 |
| LGI1-17 | 2.4 | 2.4 | 4.1 | 3.7 | 4 | 3.6 | 4 | 5.9 | 3.4 | 3.1 | 2.4 | 2.6 | 0.9 | 1.7 | 3.3 | 2.1 | 5.1 | 5 |
| LGI1-18 | 3 | 1.8 | 4.3 | 4 | 3.3 | 2.8 | 2.3 | 2.3 | 4.2 | 3.2 | 3.8 | 2.1 | 2.7 | 2.4 | 3.7 | 2.2 | 1.8 | 1.3 |
| LGI1-19 | 2.3 | 2.1 | 4.3 | 2.9 | 3.5 | 3.4 | 3.1 | 4.3 | 3.8 | 4.2 | 4.2 | 3.7 | 3.8 | 5.8 | 6.3 | 5.1 | 2.4 | 3.8 |
| LGI1-20 | 3.2 | 0.9 | 3.8 | 2.7 | 4.5 | 3 | 2.5 | 3 | 3.9 | 4.5 | 2.6 | 2.2 | 1.8 | 3.1 | 2.9 | 2.6 | 3.6 | 3 |
| LGI1-21 | 2.2 | 2.1 | 6.7 | 6.1 | 6.1 | 4.8 | 4.9 | 3.9 | 3.3 | 3.4 | 1.2 | 0.4 | -0.8 | -0.1 | 1.3 | 1 | 1.9 | 3 |
| LGI1-22 | 2.3 | 2.3 | 4 | 3.6 | 3.9 | 3.5 | 3.9 | 5.8 | 3.3 | 3 | 2.3 | 2.5 | 0.8 | 1.6 | 3.2 | 2 | 5 | 4.9 |
| NMDAR-23 | 1 | 1.3 | 3 | 4.8 | 3.8 | 2.6 | 2.4 | 7.7 | 3 | 9.2 | 3.6 | 9.2 | 4.8 | 4.8 | 4.4 | 9.7 | 0.9 | 1 |
| NMDAR-24 | 2.4 | 1.4 | 4.1 | 1.6 | 4.1 | 4.6 | 3.9 | -0.7 | 4.4 | 3.5 | 6.5 | 3.9 | 2.8 | 4.1 | 3.7 | 3.9 | 2 | 1.6 |

| Region of Interest | Superior parietal gyrus | Superior parietal gyrus | Superior temporal gyrus | Superior temporal gyrus | Supplementary motor area | Supplementary motor area | Supramarginal gyrus | Supramarginal gyrus | Temporal lobe | Temporal lobe | Temporal pole: middle temporal gyrus | Temporal pole: middle temporal gyrus | Temporal pole: superior temporal gyrus | Temporal pole: superior temporal gyrus | Thalamus | Thalamus |
| --- | --- | --- | --- | --- | --- | --- | --- | --- | --- | --- | --- | --- | --- | --- | --- | --- |
| Side | L | R | L | R | L | R | L | R | L | R | L | R | L | R | L | R |
| GABABR-1 | 2.2 | 1.9 | 1.7 | 5.1 | 7.5 | 5.7 | 1.9 | 3.3 | 6 | 5.2 | 4.6 | 5.4 | 5.1 | 6 | 3.7 | 2.4 |
| GABABR-2 | 3.7 | 4.4 | 3.5 | 4.3 | 1.9 | 1.1 | 2.1 | 1.8 | 5.4 | 4.4 | 2.4 | 2.7 | 6.3 | 4.5 | 11 | 7.8 |
| GABABR-3 | 2.2 | 1.9 | 1.7 | 5.1 | 7.5 | 5.7 | 1.9 | 3.3 | 6 | 5.2 | 4.6 | 5.4 | 5.1 | 6 | 3.7 | 2.4 |
| GABABR-4 | 2.2 | 1.9 | 1.7 | 5.1 | 7.5 | 5.7 | 1.9 | 3.3 | 6 | 5.2 | 4.6 | 5.4 | 5.1 | 6 | 3.7 | 2.4 |
| GAD65-5 | 2.4 | 2.9 | 1.6 | 1.6 | 6.3 | 5.7 | 1.1 | 0.8 | 3 | 1.7 | 1.6 | 1.3 | 1.6 | 2.2 | 3.6 | 3.1 |
| GAD65-6 | 1.3 | 1.7 | 1.2 | 2.4 | 6.8 | 5.8 | 1.4 | 1.2 | 2.4 | 2.4 | 0.6 | 1.1 | 1.5 | 1.8 | 3.2 | 3.4 |
| GAD65-7 | 5.2 | 3.8 | 3.4 | 4.3 | 3.9 | 4.6 | 3.7 | 3.7 | 3.4 | 5.4 | 2.9 | 3.7 | 3.1 | 4.5 | 4.5 | 4.7 |
| PNMA2-8 | 3.5 | 2 | 2.6 | 4.7 | 4.4 | 4.3 | 0.8 | 3.3 | 2.6 | 4.7 | -1.3 | 4.4 | 0.9 | 2.9 | 2.5 | 4.9 |
| AMPHIN-9 | 2.9 | 0.9 | 1.8 | 2.5 | 4.1 | 4.3 | 1.4 | 0.5 | 2.9 | 3.6 | 1.3 | 1.1 | 1.2 | 1.1 | 7.8 | 7.7 |
| HU+SOX1-10 | 2.5 | 1.2 | 3.2 | 2.5 | 3.2 | 3 | 2.5 | 1.8 | 3.9 | 3.8 | 2.9 | 2.1 | 3.3 | 2.4 | 3.2 | 3.9 |
| HU+SOX1-11 | 3.4 | 2.3 | 4 | 3.8 | 5.8 | 4.1 | 2.8 | 2.5 | 4 | 3.8 | 0 | 0.5 | 3.3 | 3.7 | 8.1 | 2 |
| HU-12 | 3.4 | 2.8 | 1.8 | 2.6 | 7.4 | 6 | 2.1 | 2 | 1.9 | 2.6 | 1 | 1.9 | 1.2 | 1.9 | 3.5 | 3.3 |
| HU-13 | 3.9 | 2.7 | 2.2 | 3.1 | 4.2 | 2.5 | 1.7 | 2.7 | 3.5 | 3.1 | 2.3 | 2.1 | 2.7 | 3.2 | 5.1 | 5.9 |
| HU-14 | 1 | 2.1 | 1.8 | 2.6 | 3.2 | 3.3 | 2.3 | 5.3 | 2.2 | 3.6 | 2.2 | 1.5 | 1.1 | 1.5 | 3.5 | 4.5 |
| HU-15 | 4.3 | 2.8 | 2.7 | 5.1 | 5.2 | 4.8 | 2.6 | 6.6 | 4.3 | 5.1 | 2.1 | 2.7 | 2.5 | 2.5 | 7.3 | 7.2 |
| HU-16 | 2.6 | 1.3 | 1.8 | 2.1 | 2.7 | 3.4 | 2.3 | 1.6 | 2 | 2.4 | 0 | -0.2 | 0.3 | 0.6 | 3.8 | 3.3 |
| LGI1-17 | 3.8 | 4 | 4.5 | 6.8 | 3.9 | 2.6 | 3.2 | 5.6 | 4.5 | 6.8 | 3 | 3.4 | 5.2 | 4.6 | 4 | 3.9 |
| LGI1-18 | 1.8 | 2.2 | 4 | 2.4 | 3.6 | 3.1 | 1.9 | 2.7 | 4.6 | 2.8 | 2.9 | 2.2 | 3.2 | 6 | 5.1 | 3.9 |
| LGI1-19 | 2.9 | 4 | 3.2 | 4 | 4.1 | 4 | 1.7 | 2.3 | 3.4 | 5.7 | 2.1 | 3.1 | 3.1 | 3.3 | 7 | 5.7 |
| LGI1-20 | 3 | 3 | 2.6 | 4 | 3.3 | 2.9 | 2 | 1.8 | 3.9 | 6.7 | 1.5 | 1.3 | 1.4 | 0.3 | 4.8 | 3.2 |
| LGI1-21 | 4.9 | 3.8 | 4.4 | 3.3 | 5.8 | 5.5 | 2.9 | 2.1 | 4.4 | 3.7 | 2 | 1.4 | 2.1 | 1.7 | 4.5 | 3 |
| LGI1-22 | 3.7 | 3.9 | 4.4 | 6.7 | 3.8 | 2.5 | 3.1 | 5.5 | 4.4 | 6.7 | 2.9 | 3.3 | 5.1 | 4.5 | 3.9 | 3.8 |
| NMDAR-23 | 3.3 | 4.4 | 2.5 | 11.2 | 5.3 | 4.7 | 0 | 5.3 | 3.3 | 11.2 | 3.2 | 9.7 | 3.8 | 10.4 | 1.8 | 1.9 |
| NMDAR-24 | 3.2 | 2.9 | 3.1 | -1.6 | 4.6 | 5.2 | 2.3 | -2.4 | 4.1 | -0.4 | 3.6 | -0.1 | 3 | -0.1 | 4.2 | 2.7 |

Abbreviation: NMDAR: N-methyl-D-aspartate receptor; LGI1: leucine-rich glioma inactivated-1; CASPR2: contacting-associated protein-2; GABABR: gamma-aminobutyric acid receptor; AMPAR: α-amino-3-hydroxy-5-methyl-4-isoxazole propionic acid receptor; GAD65: glutamic acid decarboxylase 65, SUVmaxstd: standard deviation of the SUVmax, AAL: automated anatomical labelling.

Supplementary Table 4 The SUV_meanstd_ of case groups according to AAL standards.

| Region of Interest | Amygdala | Amygdala | Angular gyrus | Angular gyrus | Anterior cingulate and paracingulate gyri | Anterior cingulate and paracingulate gyri | Basal ganglia | Basal ganglia | Calcarine fissure and surrounding cortex | Calcarine fissure and surrounding cortex | Caudate nucleus | Caudate nucleus | Central region | Central region | Cingulate and paracingulate gyri | Cingulate and paracingulate gyri | Cuneus | Cuneus |
| --- | --- | --- | --- | --- | --- | --- | --- | --- | --- | --- | --- | --- | --- | --- | --- | --- | --- | --- |
| Side | L | R | L | R | L | R | L | R | L | R | L | R | L | R | L | R | L | R |
| GABABR-1 | 7.1 | 7.6 | -3.9 | -4.8 | 2.3 | 3.1 | 1.4 | -0.5 | -0.5 | -0.5 | -0.8 | -1.1 | -1.4 | -1.5 | 2.4 | 2 | 0.1 | 1 |
| GABABR-2 | 11.8 | 6.5 | -4.3 | -2.9 | 0.2 | 0.1 | 0.7 | 1 | 0.2 | 0.4 | -0.3 | 0 | -1.6 | -2.4 | 0.6 | -0.7 | 3.2 | 1.6 |
| GABABR-3 | 6.6 | 7.1 | -4.4 | -5.3 | 1.8 | 2.6 | 0.9 | -1 | -1 | -1 | -1.3 | -1.6 | -1.9 | -2 | 1.9 | 1.5 | -0.4 | 0.5 |
| GABABR-4 | 6.9 | 7.4 | -4.1 | -5 | 2.1 | 2.9 | 1.2 | -0.7 | -0.7 | -0.7 | -1 | -1.3 | -1.6 | -1.7 | 2.2 | 1.8 | -0.1 | 0.8 |
| GAD65-5 | 0.4 | 0.5 | -1.8 | -0.2 | -1.5 | -1.8 | -2.2 | -2.2 | 1.1 | 3.7 | -3.1 | -2.3 | -0.4 | -2.7 | -1.2 | -2 | 2.2 | 2.9 |
| GAD65-6 | 0.5 | 0.2 | -3.1 | -1.3 | -1.1 | -1 | -0.6 | -1.1 | 0.3 | 2.2 | -1.7 | -1.7 | -0.2 | 0.2 | -0.8 | -1.2 | 0.6 | 0.8 |
| GAD65-7 | 3 | 3.5 | -0.5 | 0 | 1 | 1.1 | -0.1 | 1 | -0.2 | 0.3 | -1.2 | 0 | 0.7 | 0.3 | 2 | 1.6 | 0.5 | 2 |
| PNMA2-8 | 1.1 | -3.2 | -2.2 | -0.5 | -0.3 | 3 | 0.4 | 2.9 | -2.1 | 0.1 | -0.4 | 0.8 | 1.8 | 1 | -1 | 2.5 | -2.2 | 0.7 |
| AMPHIN-9 | 0 | 0 | -8.9 | -2.2 | -0.6 | -0.9 | -0.8 | -0.1 | 0.8 | 3.2 | -2.5 | -1.5 | 1 | 1.3 | 0.6 | -0.2 | 3.1 | 2.1 |
| HU+SOX1-10 | 5.9 | 2.9 | -3.9 | -1.6 | 0 | -0.2 | -0.4 | -0.6 | 1.9 | 2.4 | -1.3 | -0.5 | 1.8 | -0.7 | 0.7 | 0.2 | 1.2 | 1.3 |
| HU+SOX1-11 | 6.1 | 4.9 | -1.9 | -1.8 | 0.3 | 0.2 | 4.6 | 1.3 | -0.8 | -0.2 | 2.2 | 0.5 | 1.8 | 0 | 0.8 | 0.1 | -1 | 0.8 |
| HU-12 | 2.6 | 0.8 | -2.4 | -2.1 | -0.8 | -0.1 | 8.2 | 1.2 | -2.6 | -1.3 | 2.9 | -0.8 | 0.8 | 1.4 | -0.1 | -0.4 | -0.5 | -0.7 |
| HU-13 | 2.9 | 2.6 | -1.3 | -0.1 | 1.1 | 0.7 | 2.5 | 2 | 0.9 | 0 | 0.9 | 1 | 0.1 | -0.5 | 2 | 1.1 | 0.2 | 1 |
| HU-14 | 2 | 0.5 | -4 | 0.4 | -0.2 | -0.2 | -3.8 | 1.6 | -0.1 | 0.8 | -6.4 | -1 | -2.6 | 2.6 | -1.6 | -0.4 | 1.1 | 2.5 |
| HU-15 | 4.2 | 4.5 | -1.6 | -0.5 | -0.3 | -1.5 | 1.2 | 1.4 | -0.1 | -0.6 | -1.1 | -0.6 | 2.7 | 2.8 | 0.4 | -1 | 1 | 1.6 |
| HU-16 | 0 | -2.2 | 0.4 | -0.4 | 1 | 0.9 | 3.8 | 2.3 | -2.4 | -3.1 | 1.2 | 0.2 | 0.4 | 1 | 2.2 | 1.4 | 0.1 | -2 |
| LGI1-17 | 2.4 | 3.3 | -2.9 | -0.5 | -1.1 | -0.7 | 1.3 | 1.4 | 1.3 | 0.8 | 0.2 | 0.3 | -0.3 | -1.5 | -2 | -1.9 | 0.8 | 1.4 |
| LGI1-18 | 3.9 | 7 | -0.4 | -2.4 | 2.5 | 1.5 | 5.5 | 2.8 | 3.5 | 1.6 | 3.2 | 0.5 | 3 | 0.7 | 2.7 | 1.3 | 0.9 | 0.1 |
| LGI1-19 | 5.1 | 3.7 | -1.2 | 0.9 | 1.3 | 1 | 7.6 | 4.7 | -0.1 | -0.9 | 1.9 | -0.5 | 0.4 | -0.7 | 0.1 | -0.9 | 0.3 | 0 |
| LGI1-20 | 1.9 | 0 | 0.5 | 2.6 | -0.4 | -0.5 | -2.5 | -1.5 | 1 | 0.4 | -5.3 | -2.3 | 0.2 | -3.2 | -1.7 | -2.5 | 0.8 | 0.8 |
| LGI1-21 | 3 | 3.9 | -3.6 | -2.8 | -0.2 | -0.5 | 2.9 | 4 | 0 | 1 | -0.9 | 1.3 | 4 | 4.3 | 0.7 | 0.4 | -1.1 | 0.3 |
| LGI1-22 | 3.77 | 6.87 | -0.53 | -2.53 | 2.37 | 1.37 | 5.37 | 2.67 | 3.37 | 1.47 | 3.07 | 0.37 | 2.87 | 0.57 | 2.57 | 1.17 | 0.77 | -0.03 |
| NMDAR-23 | 5.2 | 5.1 | -4.6 | -4.4 | 2.9 | 4.2 | 0.7 | -0.5 | -4.8 | -2.9 | -0.3 | -1.1 | -2.4 | 3.6 | 2.5 | 3.3 | -4.5 | -3.7 |
| NMDAR-24 | -0.4 | -0.5 | 0.2 | -9.7 | -0.3 | -0.1 | 0.5 | -1.1 | 1.6 | -0.9 | -1.2 | -1.9 | 3.3 | -9.1 | 1.3 | 1.1 | 2.2 | -0.9 |

| Region of Interest | Frontal lobe | Frontal lobe | Fusiform gyrus | Fusiform gyrus | Gyrus rectus | Gyrus rectus | Heschl gyrus | Heschl gyrus | Hippocampus | Hippocampus | Inferior frontal gyrus, opercular part | Inferior frontal gyrus, opercular part | Inferior frontal gyrus, orbital part | Inferior frontal gyrus, orbital part | Inferior frontal gyrus, triangular part | Inferior frontal gyrus, triangular part | Inferior occipital gyrus | Inferior occipital gyrus |
| --- | --- | --- | --- | --- | --- | --- | --- | --- | --- | --- | --- | --- | --- | --- | --- | --- | --- | --- |
| Side | L | R | L | R | L | R | L | R | L | R | L | R | L | R | L | R | L | R |
| GABABR-1 | -3 | -1.8 | 0.8 | 3.1 | 2.5 | 2.9 | -0.8 | 0.4 | 4.4 | 14 | -3 | 0.5 | 0.4 | 0 | -5.7 | -0.5 | -0.8 | -1 |
| GABABR-2 | -4.9 | -6.6 | 3.4 | 2.3 | -2.5 | -1.7 | -1.2 | -0.9 | 5.7 | 4.1 | 0 | -2.5 | -3.8 | -3.1 | -6.2 | -4.9 | -0.9 | -1.8 |
| GABABR-3 | -3.5 | -2.3 | 0.3 | 2.6 | 2 | 2.4 | -1.3 | -0.1 | 3.9 | 13.5 | -3.5 | 0 | -0.1 | -0.5 | -6.2 | -1 | -1.3 | -1.5 |
| GABABR-4 | -3.2 | -2 | 0.6 | 2.9 | 2.3 | 2.7 | -1 | 0.2 | 4.2 | 13.8 | -3.2 | 0.3 | 0.2 | -0.2 | -5.9 | -0.7 | -1 | -1.2 |
| GAD65-5 | -2.6 | -4.1 | 0.1 | -0.4 | -2.7 | -2.7 | -0.3 | 0 | -0.3 | -1.2 | -0.4 | -2.2 | -1.7 | -1.5 | -3.7 | -1.9 | -2 | -1.6 |
| GAD65-6 | -2.2 | -2.5 | 0.1 | 0.1 | -3.1 | -2.8 | 1.1 | 0.6 | -1.3 | -1.3 | 0.2 | -1 | -2.1 | -1.3 | -4.6 | -1.6 | -0.9 | -0.1 |
| GAD65-7 | -1.9 | -1.1 | 1.4 | 1.2 | -1.9 | -1.3 | -2.2 | -0.6 | 4.1 | 2.9 | -0.4 | -0.5 | -2.5 | -0.6 | -4.2 | -1.1 | 0 | 0.3 |
| PNMA2-8 | -4.2 | -0.2 | -6.5 | -1.2 | -6 | 2 | 0.2 | 0.5 | -2.1 | 1.8 | -2.5 | -1.9 | -4.5 | -0.7 | -6.5 | -1.9 | -4.3 | -2.4 |
| AMPHIN-9 | -6.6 | -5.4 | 1.5 | 2.2 | -2.7 | -1.6 | 0.8 | 2.3 | -0.6 | 0.3 | -2 | -2.1 | -4.8 | -2.7 | -7.7 | -4.2 | 3 | -0.7 |
| HU+SOX1-10 | -1.6 | -3.2 | 1.6 | 1.9 | -1.3 | -1.8 | 0.9 | 0.6 | 1.6 | 2 | -0.3 | -1 | -1.8 | -3.1 | -3.4 | -2.3 | 3.1 | 2 |
| HU+SOX1-11 | -2.3 | -4.7 | 1.6 | 0.9 | -3.6 | -4 | 0.1 | 0.3 | 7.7 | 8.2 | -0.1 | -2.6 | -1.9 | -3 | -2.7 | -3.3 | 0.6 | 0.1 |
| HU-12 | -2 | -0.9 | -2 | -1.6 | -2.8 | -2.1 | -0.9 | 0.6 | 0.1 | 1.1 | -1.8 | -0.8 | -4.5 | -2.7 | -4.1 | -2.5 | -2.8 | -3.1 |
| HU-13 | -0.3 | -1.1 | 0.1 | 0.7 | -0.1 | -1.2 | -1.6 | -1 | 3.3 | 2.2 | -0.3 | -0.9 | -0.2 | -1.2 | -2.3 | -1.2 | -0.2 | -1.1 |
| HU-14 | -5.1 | -2.7 | -0.2 | -0.2 | -3.2 | -1.5 | -4.9 | -0.5 | -0.2 | -0.8 | -3.8 | -1.4 | -6 | -5.6 | -6.4 | -2 | -1.4 | -2 |
| HU-15 | -0.6 | -0.8 | -1.3 | -1.1 | 1.6 | 3 | -1.1 | -0.6 | 4.2 | 5.1 | -0.2 | -1.4 | 1.4 | -0.5 | -3.5 | -2.3 | -1.8 | -2.8 |
| HU-16 | -1.1 | -0.3 | -3.5 | -2.5 | -1.3 | -1.4 | -4 | -2.2 | -0.2 | -0.7 | -1.6 | -1.2 | -2 | -1.5 | -3.1 | -1.7 | -3.3 | -3.3 |
| LGI1-17 | -3.5 | -3.7 | 1.7 | 2.6 | -2.6 | -3.4 | 0.4 | -0.4 | 2.4 | 2.9 | -0.5 | -0.9 | -1.9 | -2.2 | -4.4 | -2.3 | 1.2 | -1.1 |
| LGI1-18 | 1.2 | -1.8 | 1 | -0.6 | 0.6 | -2.3 | 1.8 | 0.1 | 4.3 | 5.6 | -0.8 | -2 | -0.7 | -2.7 | -1.7 | -2.9 | 1.1 | 0.9 |
| LGI1-19 | -2.6 | -4.5 | 2.4 | 2.6 | 1.6 | 0.1 | -0.6 | -0.9 | 2.2 | 2.7 | -0.2 | -1.7 | -2 | -1.3 | -2.7 | -2.4 | 1.7 | 0.4 |
| LGI1-20 | -2.2 | -3.9 | 2.4 | 1.5 | 0.1 | -1 | -0.6 | -2 | 0.3 | -0.1 | -0.6 | -2.5 | -1.9 | -1.5 | -0.3 | -1.8 | 3.7 | 1.6 |
| LGI1-21 | -6.7 | -7.8 | 0.2 | 2 | -5 | -4.9 | 0.4 | 2.2 | 3.3 | 1.8 | -1.5 | -2.3 | -6.8 | -5.7 | -5.4 | -4.7 | -0.5 | -2.8 |
| LGI1-22 | 1.07 | -1.93 | 0.87 | -0.73 | 0.47 | -2.43 | 1.67 | -0.03 | 4.17 | 5.47 | -0.93 | -2.13 | -0.83 | -2.83 | -1.83 | -3.03 | 0.97 | 0.77 |
| NMDAR-23 | -1.6 | 4 | -3.3 | -5.2 | 3.9 | 4.9 | -2.2 | 1.5 | 2.5 | 2.2 | -1.5 | -0.9 | -1.3 | 2.7 | -5.1 | 0.3 | -4.1 | -3.7 |
| NMDAR-24 | 0.2 | -0.4 | -0.6 | -9.6 | 1.1 | 1.6 | 0.9 | -7 | 2.5 | -1.9 | -3.8 | -3.9 | 1 | -1 | -3.3 | -3.9 | 0.3 | -9.3 |

| Region of Interest | Inferior parietal, but supramarginal and angular gyri | Inferior parietal, but supramarginal and angular gyri | Inferior temporal gyrus | Inferior temporal gyrus | Insula | Insula | Lenticular nucleus, pallidum | Lenticular nucleus, pallidum | Lenticular nucleus, putamen | Lenticular nucleus, putamen | Lingual gyrus | Lingual gyrus | Mesial temporal lobe | Mesial temporal lobe | Middle cingulate and paracingulate gyri | Middle cingulate and paracingulate gyri | Middle frontal gyrus | Middle frontal gyrus |
| --- | --- | --- | --- | --- | --- | --- | --- | --- | --- | --- | --- | --- | --- | --- | --- | --- | --- | --- |
| Side | L | R | L | R | L | R | L | R | L | R | L | R | L | R | L | R | L | R |
| GABABR-1 | -6.3 | -4.4 | 0.6 | -0.1 | 1.1 | 2.3 | 2.3 | 0.8 | 3.8 | 1.2 | 1.4 | -0.8 | 4.2 | 8.2 | 2.3 | 1.2 | -5.9 | -3.3 |
| GABABR-2 | -2.6 | -2.4 | 1.9 | 0.4 | 0.2 | -1.2 | 2.4 | 3.1 | 1.3 | 2.2 | 2.5 | 1.5 | 5.6 | 3.4 | 1.8 | -1.1 | -3.3 | -4.1 |
| GABABR-3 | -6.8 | -4.9 | 0.1 | -0.6 | 0.6 | 1.8 | 1.8 | 0.3 | 3.3 | 0.7 | 0.9 | -1.3 | 3.7 | 7.7 | 1.8 | 0.7 | -6.4 | -3.8 |
| GABABR-4 | -6.5 | -4.6 | 0.4 | -0.3 | 0.9 | 2.1 | 2.1 | 0.6 | 3.6 | 1 | 1.2 | -1 | 4 | 8 | 2.1 | 1 | -6.1 | -3.5 |
| GAD65-5 | -1.2 | -0.3 | -0.2 | -1.7 | -1 | -1.8 | 1 | 1.2 | -0.3 | -1.1 | 2.2 | 1.3 | 0.2 | 0.3 | 0.1 | -1.6 | -0.6 | -3.3 |
| GAD65-6 | -1.3 | -0.9 | -1.2 | -1.6 | -0.5 | -0.3 | 1.4 | 0.9 | 1 | 0.7 | 1.4 | 0.2 | -0.4 | 0.3 | 0.5 | -0.7 | -0.7 | -2 |
| GAD65-7 | -1.6 | 0 | -0.8 | -0.1 | -1 | 0 | 2.8 | 3.3 | 0.9 | 2.2 | 0.4 | 0.8 | 3.6 | 2.9 | 3 | 1.6 | -2.3 | -1.6 |
| PNMA2-8 | -2.9 | -1 | -11.3 | 0.2 | -4.7 | 3.1 | 3.5 | 3.8 | 0.6 | 5.9 | -3.5 | 0.3 | -3 | 0.8 | -0.9 | 1.6 | -2.5 | -1.7 |
| AMPHIN-9 | -4.8 | -3.2 | -4.2 | -2.4 | -1.7 | -0.6 | 1.2 | 2.2 | 1.8 | 2.8 | 4 | 2.5 | -1 | 0.1 | 1.9 | 0.5 | -5.4 | -3.9 |
| HU+SOX1-10 | -2.5 | -1.8 | 0.9 | -0.5 | 0.9 | 1.2 | 1.4 | 1 | 0.7 | -0.8 | 2.5 | 1.5 | 2.9 | 2.2 | 1.3 | 0.5 | -0.9 | -2.7 |
| HU+SOX1-11 | -0.7 | -2.5 | -0.6 | -1.2 | -0.1 | -0.8 | 4.2 | 3.3 | 5.6 | 1.9 | 1.9 | 1.2 | 6.1 | 6.5 | 1.8 | 0.4 | -1.6 | -3.3 |
| HU-12 | -3.4 | -2.1 | -3.2 | -2.9 | -1.8 | -0.9 | 6.8 | 1.3 | 11.9 | 5.7 | -1.8 | -0.9 | 0 | 0.6 | 1 | -0.1 | -2 | -1 |
| HU-13 | -1.1 | 0.4 | 0.3 | -0.2 | 0.5 | 0.7 | 4.3 | 3.6 | 2.8 | 2.7 | -0.4 | -0.3 | 2.5 | 2.1 | 2.1 | 0.8 | -0.9 | -1.3 |
| HU-14 | -4.1 | -0.8 | -4.3 | -1.3 | -1.6 | 1.2 | 0.8 | 4.7 | 1.4 | 6.4 | 0.9 | 0.9 | 1.3 | -0.3 | -1.6 | -0.6 | -4.1 | -1.8 |
| HU-15 | -1.6 | -0.2 | -0.3 | -0.1 | -2 | -0.9 | 5.1 | 4.6 | 2.9 | 4.6 | -1.6 | -0.8 | 2.4 | 3.8 | 1.2 | -0.6 | -1 | -2.2 |
| HU-16 | -0.8 | -1.3 | -2.4 | -3.3 | -1.7 | -1.8 | 3.2 | 2.7 | 5.5 | 6 | -3.9 | -2.5 | -1 | -1 | 3.3 | 1.9 | -0.9 | -0.2 |
| LGI1-17 | -2.4 | -1.6 | -0.2 | 0.1 | 0.5 | -0.4 | 3.9 | 4.4 | 1.4 | 2.4 | 0.8 | 3 | 2.3 | 2.2 | -2.3 | -2.6 | -3.2 | -2.8 |
| LGI1-18 | -0.2 | -2.1 | 1.4 | -1.8 | -0.4 | -2.5 | 1.4 | 0.7 | 6 | 4.4 | 0.7 | 0.8 | 4 | 5.1 | 1.9 | 1.1 | 0.9 | -1.2 |
| LGI1-19 | -4 | -1.9 | 1.9 | 1.4 | 1.1 | 1.1 | 7.1 | 7.8 | 11.9 | 13.9 | 1 | 1.1 | 3.1 | 3.7 | -0.8 | -2.1 | -2.1 | -2.9 |
| LGI1-20 | -2.9 | -1.1 | 2.4 | 1.3 | -1.1 | -1.9 | 2.3 | 2.6 | 2.1 | 0.7 | 2.7 | 1.5 | 1 | 0.9 | -2 | -3.6 | -0.2 | -1.7 |
| LGI1-21 | -1.8 | -2.3 | -2.1 | -1.3 | 0.2 | 0.6 | 0.7 | 0.8 | 7.5 | 8.9 | 0 | 0.3 | 2.2 | 1.3 | 2.4 | 1.5 | -5.1 | -5.8 |
| LGI1-22 | -0.33 | -2.23 | 1.27 | -1.93 | -0.53 | -2.63 | 1.27 | 0.57 | 5.87 | 4.27 | 0.57 | 0.67 | 3.87 | 4.97 | 1.77 | 0.97 | 0.77 | -1.33 |
| NMDAR-23 | -4.3 | -0.6 | -1.9 | -3.1 | 0.2 | 8 | -0.2 | 1 | 1.6 | 0.3 | -4.4 | -1.6 | 2.8 | 2.2 | 2.3 | 2.3 | -3.2 | 1.5 |
| NMDAR-24 | -2.3 | -6.4 | -0.4 | -12.9 | 0.1 | -3.8 | -0.2 | -1.1 | 2 | 0.8 | -0.4 | -5.4 | 1.5 | -2.1 | 2.8 | 2.1 | -1.4 | -2.3 |

| Region of Interest | Middle frontal gyrus, orbital part | Middle frontal gyrus, orbital part | Middle occipital gyrus | Middle occipital gyrus | Middle temporal gyrus | Middle temporal gyrus | Occipital lobe | Occipital lobe | Olfactory cortex | Olfactory cortex | Paracentral lobule | Paracentral lobule | Parahippocampal gyrus | Parahippocampal gyrus | Parietal lobe | Parietal lobe | Postcentral gyrus | Postcentral gyrus |
| --- | --- | --- | --- | --- | --- | --- | --- | --- | --- | --- | --- | --- | --- | --- | --- | --- | --- | --- |
| Side | L | R | L | R | L | R | L | R | L | R | L | R | L | R | L | R | L | R |
| GABABR-1 | 1.4 | -0.7 | -3.9 | -2.2 | -3.1 | -1.5 | -1.4 | -0.2 | 1.7 | 2 | 0.4 | 0 | 2.1 | 1.2 | -4.9 | -4.4 | -1 | -2.6 |
| GABABR-2 | 0.6 | -0.4 | -2.2 | -1.2 | -0.9 | 0.3 | 1.5 | 1 | -0.1 | -1.5 | 0.5 | -0.8 | 2.4 | 1.2 | -3.4 | -3.2 | -1.8 | -1.1 |
| GABABR-3 | 0.9 | -1.2 | -4.4 | -2.7 | -3.6 | -2 | -1.9 | -0.7 | 1.2 | 1.5 | -0.1 | -0.5 | 1.6 | 0.7 | -5.4 | -4.9 | -1.5 | -3.1 |
| GABABR-4 | 1.2 | -0.9 | -4.1 | -2.4 | -3.3 | -1.7 | -1.6 | -0.4 | 1.5 | 1.8 | 0.2 | -0.2 | 1.9 | 1 | -5.1 | -4.6 | -1.2 | -2.8 |
| GAD65-5 | -1.3 | -1.9 | -1.1 | 0.2 | -1.7 | -2.2 | 0.9 | 2 | 1 | 1.1 | 2.5 | 1.3 | 0.6 | 1.4 | 0.2 | 0.2 | -1 | -3 |
| GAD65-6 | -1.1 | -1.7 | -1.1 | 0.4 | -3.9 | -2.8 | 0.1 | 1.1 | 1.3 | 1.1 | 2.6 | 1.6 | 0.5 | 1.6 | -1.4 | -1.4 | -1.1 | -1.2 |
| GAD65-7 | -1 | -0.2 | 0 | 0.6 | -0.7 | 0 | 0.4 | 1.4 | 1.8 | 0.8 | 2.4 | 2.3 | 2 | 2.1 | 0.3 | 0.8 | 1.1 | 1.3 |
| PNMA2-8 | -2 | -1.6 | -2.9 | -0.9 | -11.3 | 0.7 | -5.5 | -1.1 | -2.4 | 2.7 | 2.9 | 1.9 | -3.8 | 0.5 | -4.2 | 0 | 2 | -0.7 |
| AMPHIN-9 | -1.9 | -1.3 | -0.5 | 0.2 | -12.8 | -2.8 | 3 | 2.9 | -1.8 | -3.1 | 0.1 | -0.5 | -1.3 | -0.1 | -5.5 | -5.1 | 1.9 | 1.4 |
| HU+SOX1-10 | -0.1 | -0.2 | -1.9 | -0.6 | -1.8 | -1.8 | 1.5 | 2 | 0.6 | 1.5 | 0.5 | -0.3 | 2.6 | 1.6 | -2.1 | -1.2 | 0.8 | -1.8 |
| HU+SOX1-11 | -1.5 | -0.5 | 0.6 | 0 | 0.5 | -1 | 0.7 | 0.7 | 2.8 | 1.2 | 2 | 0.7 | 2.4 | 3.7 | -0.3 | -1.5 | 1 | -0.3 |
| HU-12 | -1.3 | 0.4 | -3.8 | -2.3 | -3.7 | -2.3 | -3.9 | -2.7 | -0.2 | -0.4 | 3.1 | 2.1 | -0.6 | -0.1 | -1.6 | -1.6 | 0.5 | 0.4 |
| HU-13 | -0.4 | 0.7 | 0.6 | 0.8 | -0.1 | 0 | 0.5 | 0.5 | 1.5 | 1.4 | 0.6 | 1.2 | 0.8 | 1.3 | 0.4 | 0.4 | -0.2 | 0.1 |
| HU-14 | -1 | 0.7 | -0.9 | 0.5 | -4 | 0.6 | -0.1 | 1.2 | -2.5 | -1.5 | 1.2 | 1.3 | 2.2 | 0.1 | -4.3 | 0.9 | -0.6 | 2 |
| HU-15 | 0 | 2.6 | -1.3 | -0.9 | -0.9 | -0.4 | -1.1 | -1 | 1.4 | 1.8 | 2 | 0.1 | -0.5 | 1.5 | -0.3 | 0.3 | 1.4 | 1.6 |
| HU-16 | 0.5 | 1.2 | -2 | -1.7 | -1.4 | -0.3 | -4.5 | -4.1 | -1.4 | -0.7 | 1.1 | 0.8 | -1.7 | -0.7 | 0.6 | -1.2 | 1.6 | 0.5 |
| LGI1-17 | -0.7 | 0.2 | -0.5 | 0.7 | -2.3 | -1.8 | 1.7 | 2.6 | 2.3 | 0 | 1.3 | 0.2 | 1.4 | 0.8 | -2.2 | -0.9 | -0.4 | -2.1 |
| LGI1-18 | 0.2 | 0.7 | -0.7 | -0.5 | 2.1 | -1.6 | 1.2 | 0.4 | 2.7 | 1 | 2 | 0.7 | 2.6 | 2.7 | -1.6 | -3.5 | 2.6 | 1.4 |
| LGI1-19 | 0.4 | 0.5 | -0.5 | 1.2 | 0.7 | 0.6 | 1 | 1.3 | 3.2 | 2.2 | -0.8 | -4.4 | 2.6 | 3.6 | -3.6 | -1.6 | -0.4 | -1.5 |
| LGI1-20 | 0.8 | 1.3 | 0.3 | 1 | 0.2 | -0.3 | 2.5 | 1.6 | -0.4 | -0.6 | -0.9 | -3.7 | 1.3 | 1.7 | -1.8 | -0.7 | 0 | -2.3 |
| LGI1-21 | -2.7 | -2.5 | -2.7 | -0.8 | -2.7 | -1.4 | -1.3 | 0.3 | -0.8 | 0.1 | 4.1 | 2.5 | 0.1 | -0.1 | -1.8 | -2.7 | 2.7 | 4 |
| LGI1-22 | 0.07 | 0.57 | -0.83 | -0.63 | 1.97 | -1.73 | 1.07 | 0.27 | 2.57 | 0.87 | 1.87 | 0.57 | 2.47 | 2.57 | -1.73 | -3.63 | 2.47 | 1.27 |
| NMDAR-23 | -0.3 | 2.7 | -7.1 | -4.7 | -3.7 | 0.6 | -6.7 | -5.2 | 6.7 | 5.7 | 1.6 | 1.1 | 1.8 | 0.9 | -5.9 | -2.4 | -1 | 2.9 |
| NMDAR-24 | 0.8 | 3.1 | -0.8 | -12.3 | 1.1 | -16.2 | 0 | -7.9 | 1.1 | -0.7 | 0.8 | 1 | 0.6 | -2.1 | 0.1 | -10.1 | 3.2 | -6.2 |

| Region of Interest | Posterior cingulate and paracingulate gyri | Posterior cingulate and paracingulate gyri | Precentral gyrus | Precentral gyrus | Precuneus | Precuneus | Rolandic operculum | Rolandic operculum | Superior frontal gyrus, dorsolateral | Superior frontal gyrus, dorsolateral | Superior frontal gyrus, medial | Superior frontal gyrus, medial | Superior frontal gyrus, medial orbital | Superior frontal gyrus, medial orbital | Superior frontal gyrus, orbital part | Superior frontal gyrus, orbital part | Superior occipital gyrus | Superior occipital gyrus |
| --- | --- | --- | --- | --- | --- | --- | --- | --- | --- | --- | --- | --- | --- | --- | --- | --- | --- | --- |
| Side | L | R | L | R | L | R | L | R | L | R | L | R | L | R | L | R | L | R |
| GABABR-1 | -0.3 | -1.1 | -2 | -1.5 | -0.3 | -1.1 | 1.2 | 3.1 | -2.5 | -3.3 | 0.2 | -1.6 | 2.2 | 1.7 | 2 | 0.9 | -2.7 | -0.5 |
| GABABR-2 | -1.7 | -1 | -0.7 | -1.9 | -1 | -2 | -0.6 | -2.6 | -2.2 | -4.3 | -2.6 | -3.4 | -1.5 | -1.8 | -0.5 | -1.9 | 0.7 | -0.1 |
| GABABR-3 | -0.8 | -1.6 | -2.5 | -2 | -0.8 | -1.6 | 0.7 | 2.6 | -3 | -3.8 | -0.3 | -2.1 | 1.7 | 1.2 | 1.5 | 0.4 | -3.2 | -1 |
| GABABR-4 | -0.5 | -1.3 | -2.2 | -1.7 | -0.5 | -1.3 | 1 | 2.9 | -2.7 | -3.5 | 0 | -1.8 | 2 | 1.5 | 1.8 | 0.7 | -2.9 | -0.7 |
| GAD65-5 | -1.3 | -1.6 | 0.5 | -1.2 | 3.6 | 2.3 | -0.6 | -1.7 | -1.1 | -1.7 | -3 | -2.4 | -1.3 | -2.3 | -2.6 | -4.3 | 0.6 | 0.9 |
| GAD65-6 | -1.6 | -1.6 | 1 | 1.2 | 1.4 | 0.4 | -0.4 | 0.3 | -0.5 | -0.4 | -2.7 | -2.3 | -1.5 | -2 | -1.9 | -3.2 | -0.5 | 0.3 |
| GAD65-7 | 0.1 | 0.7 | 0.4 | -0.3 | 1.9 | 2.1 | -1 | -0.7 | -0.8 | -0.2 | 0.2 | -0.8 | -0.6 | -1.3 | -1.3 | -1 | -0.2 | 0.6 |
| PNMA2-8 | -1.8 | 0.9 | 1.2 | 1.6 | -2.3 | 1 | 0.1 | 1.5 | -1.2 | -0.1 | -1.5 | 2.5 | -3.4 | 1.5 | -4.3 | -1 | -1.2 | -2.4 |
| AMPHIN-9 | 0.7 | -0.3 | -0.2 | 1.4 | -2.1 | -1.8 | -0.4 | -1.3 | -1.9 | -2.3 | -4.4 | -4.3 | -1.6 | -1.3 | -2 | -2.6 | 3.4 | 0.8 |
| HU+SOX1-10 | 0.4 | 0 | 1.9 | 0.6 | 0 | 0.7 | 1.5 | -0.6 | -0.6 | -0.6 | -1.3 | -1.3 | -1.8 | -1.9 | -1.9 | -3 | -2.5 | -0.4 |
| HU+SOX1-11 | -1 | -1.1 | 1.8 | 0.6 | 1 | -0.6 | 1.3 | -0.9 | -1.1 | -2.2 | -2.3 | -3.8 | -2.4 | -2.2 | -2.2 | -2.7 | 0.9 | -0.1 |
| HU-12 | -0.5 | -1.4 | 0.8 | 1.6 | 1.5 | 0.4 | 0.1 | 0.5 | 0 | 0.1 | -0.9 | -1.6 | -2.6 | -2.2 | -1.9 | -2.8 | -1 | -2.5 |
| HU-13 | 1.7 | 1.7 | 0.4 | -0.6 | 1.2 | 0.4 | -0.1 | -0.6 | 0.5 | -0.4 | 0 | -1.3 | 0.2 | -0.1 | -0.6 | 0 | -0.3 | 0.6 |
| HU-14 | -3.3 | 0 | -3.1 | 2.3 | -1.3 | 2.7 | -2.8 | 0.4 | -3.1 | -0.4 | 0.1 | -3.6 | -1.9 | -1.7 | -1.1 | 0.9 | 0.5 | 1.7 |
| HU-15 | 0 | 0.2 | 3.3 | 2.8 | 0.2 | -0.3 | 0.2 | 0.8 | 1.2 | 1.1 | -2.4 | -1.8 | -2.3 | -0.8 | -0.6 | 1.4 | 0.5 | 0.1 |
| HU-16 | 0.3 | -0.5 | -0.3 | 1.5 | 2.7 | 0.4 | -1.6 | -0.6 | 1.7 | 1.1 | -1.6 | -0.5 | -1 | 0.5 | 0.7 | 0.7 | -3.8 | -2.2 |
| LGI1-17 | -1 | -0.3 | 0.2 | 0.1 | -0.5 | 0 | -0.8 | -1.5 | -1.8 | -2.1 | -3.1 | -2.6 | -3 | -3.4 | -2.5 | -3 | 2.3 | 1.9 |
| LGI1-18 | 0.6 | -0.6 | 2.2 | 0.5 | -1.5 | -2.1 | 1.5 | -1.4 | 1.2 | 1.1 | 0.5 | -0.6 | -0.5 | -0.2 | 0.6 | -1.1 | -2 | -1.1 |
| LGI1-19 | -1.7 | -0.3 | 0.9 | -0.7 | -2.3 | -1.4 | 0.7 | 1.8 | -0.9 | -1 | -3 | -3.9 | 0.3 | 0.7 | 0.6 | -0.8 | 0.5 | 0.7 |
| LGI1-20 | -2.3 | -0.7 | 0.4 | -2.7 | 0.3 | 0 | -0.2 | -1 | -1.4 | -0.8 | -3.6 | -3.3 | -0.6 | -0.4 | 1.5 | -0.1 | 0.4 | 0.1 |
| LGI1-21 | -1.4 | -1.8 | 3.5 | 2.9 | 0.5 | -0.7 | 2.8 | 1.4 | -4.2 | -3.2 | -4.9 | -7.4 | -4.1 | -3.4 | -3.7 | -4 | -2 | -0.7 |
| LGI1-22 | 0.47 | -0.73 | 2.07 | 0.37 | -1.63 | -2.23 | 1.37 | -1.53 | 1.07 | 0.97 | 0.37 | -0.73 | -0.63 | -0.33 | 0.47 | -1.23 | -2.13 | -1.23 |
| NMDAR-23 | -1.9 | -1 | -3.2 | 1.1 | -3.3 | -4.5 | -0.8 | 6.9 | -1.1 | 3.1 | 0.1 | 3.9 | 2.3 | 3.3 | 0.3 | 3.9 | -4.9 | -4.7 |
| NMDAR-24 | 0.1 | -0.2 | 1.9 | -5.1 | 2.1 | 2.2 | 1.6 | -14.1 | 0.3 | -0.4 | 3 | 2 | 2.3 | 1.5 | 0.5 | 2.5 | -2.7 | -3 |

| Region of Interest | Superior parietal gyrus | Superior parietal gyrus | Superior temporal gyrus | Superior temporal gyrus | Supplementary motor area | Supplementary motor area | Supramarginal gyrus | Supramarginal gyrus | Temporal lobe | Temporal lobe | Temporal pole: middle temporal gyrus | Temporal pole: middle temporal gyrus | Temporal pole: superior temporal gyrus | Temporal pole: superior temporal gyrus | Thalamus | Thalamus |
| --- | --- | --- | --- | --- | --- | --- | --- | --- | --- | --- | --- | --- | --- | --- | --- | --- |
| Side | L | R | L | R | L | R | L | R | L | R | L | R | L | R | L | R |
| GABABR-1 | -2.6 | -2 | -1.1 | 1.4 | -0.3 | 1.6 | -3.4 | -2.8 | -1.7 | -0.3 | 0 | 1.5 | 1.1 | 2.8 | -0.5 | -1.6 |
| GABABR-2 | -1.8 | 0.6 | -0.7 | -0.6 | -3.6 | -2.2 | -2.1 | -4.9 | 0.1 | 0 | -0.2 | 0.3 | -0.4 | 1.3 | 6.4 | 3 |
| GABABR-3 | -3.1 | -2.5 | -1.6 | 0.9 | -0.8 | 1.1 | -3.9 | -3.3 | -2.2 | -0.8 | -0.5 | 1 | 0.6 | 2.3 | -1 | -2.1 |
| GABABR-4 | -2.8 | -2.2 | -1.3 | 1.2 | -0.5 | 1.4 | -3.6 | -3 | -1.9 | -0.5 | -0.2 | 1.3 | 0.9 | 2.6 | -0.7 | -1.8 |
| GAD65-5 | -1 | -0.1 | -1 | -1.3 | -0.1 | 0.3 | -1.8 | -2.6 | -1.3 | -2.1 | -0.7 | -0.2 | -0.1 | -1.1 | -0.2 | 0.2 |
| GAD65-6 | -1.2 | -1.1 | -2.4 | -1.7 | 0.9 | 1 | -2.6 | -2.6 | -3.2 | -2.5 | -1.7 | -0.6 | -1.1 | -1 | 0.2 | 0.8 |
| GAD65-7 | 0.5 | 0.8 | -1.1 | 0.4 | 1.3 | 1.1 | -0.1 | -1.4 | -1.2 | 0 | 0.3 | 1.2 | 0 | 1.4 | 1.3 | 1.7 |
| PNMA2-8 | -0.6 | 0.3 | -1.8 | 2 | 2.5 | 3 | -8.5 | -0.5 | -11.3 | 1 | -7.4 | 1 | -5 | 0.7 | -1.3 | 2.7 |
| AMPHIN-9 | -0.9 | -4.4 | -4.9 | -1.5 | 0.1 | 0.6 | -3.4 | -6 | -9.9 | -2.6 | -2.2 | 0.1 | -3.1 | -1.5 | 4.2 | 2.9 |
| HU+SOX1-10 | -0.7 | -0.7 | 1.2 | -1 | 2.4 | 0.9 | -0.4 | -1.5 | -0.1 | -1.3 | -0.6 | 0.3 | 1.2 | 1.4 | 1.4 | 0.8 |
| HU+SOX1-11 | -0.1 | 0.5 | 2.2 | 0.5 | 2.4 | 1.3 | -0.1 | -1.3 | 0.7 | -0.8 | -2.6 | -1.5 | -2.1 | -1 | -0.3 | -0.1 |
| HU-12 | -1 | -0.4 | -1.5 | 0 | 4 | 5.2 | -0.9 | -1.7 | -3.8 | -2.3 | -0.3 | 0.1 | -0.6 | 0.6 | -1.3 | -0.5 |
| HU-13 | 1.4 | 0.7 | -0.5 | 0 | 1.5 | 0.7 | -0.3 | -0.2 | -0.2 | -0.2 | 0.9 | 0.9 | 0.8 | 0.7 | 3.4 | 3.1 |
| HU-14 | -2.3 | -1 | -3.3 | 0.2 | 0.1 | 1.3 | -2.8 | 0.9 | -5.3 | -0.3 | -1 | -0.1 | -2.6 | -0.9 | -6.1 | -0.1 |
| HU-15 | 1.7 | 1 | -0.3 | 2.6 | 1.6 | 0.9 | -0.9 | 1.2 | -0.8 | 0.5 | 0 | 0.6 | -2 | -0.4 | 2.5 | 1.2 |
| HU-16 | -1.2 | -1.6 | -3.1 | -1.9 | 0.1 | 0.8 | 0.4 | -1.6 | -3 | -2.3 | -1.9 | -1.2 | -3 | -1.1 | -0.1 | -0.5 |
| LGI1-17 | -0.5 | 0.3 | 0.4 | 1.2 | 2.6 | 1.2 | -1.3 | -2.7 | -1.2 | -0.4 | 1.1 | 1.8 | 1.6 | 2.5 | -0.4 | 0.1 |
| LGI1-18 | -1.5 | -2.8 | 0.7 | -1.9 | 2.5 | 0.1 | -0.8 | -0.9 | 2.1 | -2.3 | 1.6 | 1 | 2.3 | 0.8 | 4.4 | 3.1 |
| LGI1-19 | -1.5 | -0.4 | -0.6 | 0.9 | -1.8 | -3.8 | -1.1 | -3.3 | 1 | 1.1 | 1.4 | 1.2 | -0.6 | -1.1 | -2.4 | -1.5 |
| LGI1-20 | -1.6 | -1.1 | -2.4 | -1.4 | -2.3 | -4.2 | -1.7 | -3.7 | 0.4 | -0.1 | 0.1 | -0.4 | -2.9 | -3.4 | -1.3 | -0.8 |
| LGI1-21 | -0.4 | 0.5 | 2.4 | -0.2 | 2.1 | 0.9 | -3 | -4.7 | -1.5 | -1.1 | -0.9 | -0.6 | -0.8 | 0.1 | 2.4 | 0.1 |
| LGI1-22 | -1.63 | -2.93 | 0.57 | -2.03 | 2.37 | -0.03 | -0.93 | -1.03 | 1.97 | -2.43 | 1.47 | 0.87 | 2.17 | 0.67 | 4.27 | 2.97 |
| NMDAR-23 | -2.7 | -0.3 | -0.8 | 5.5 | 3.2 | 1.4 | -2.9 | 3.1 | -3.2 | 1.2 | 2.6 | 4.4 | 2.7 | 6.4 | -0.1 | -0.9 |
| NMDAR-24 | 0.7 | -2 | 1.4 | -13.5 | 2 | 4.1 | -1.5 | -11.9 | 1 | -19.8 | 1.4 | -3.1 | 1.3 | -4.1 | 3 | 0.8 |

Abbreviation: NMDAR: N-methyl-D-aspartate receptor; LGI1: leucine-rich glioma inactivated-1; CASPR2: contacting-associated protein-2; GABABR: gamma-aminobutyric acid receptor; AMPAR: α-amino-3-hydroxy-5-methyl-4-isoxazole propionic acid receptor; GAD65: glutamic acid decarboxylase 65, SUVmeanstd: standard deviation of the SUVmean, AAL: automated anatomical labelling.

Supplementary Table 5 The SUV_mean_ of normal 19-44 years old group according to AAL standards.

| Region of Interest | Amygdala | Amygdala | Angular gyrus | Angular gyrus | Anterior cingulate and paracingulate gyri | Anterior cingulate and paracingulate gyri | Basal ganglia | Basal ganglia | Calcarine fissure and surrounding cortex | Calcarine fissure and surrounding cortex | Caudate nucleus | Caudate nucleus | Central region | Central region | Cingulate and paracingulate gyri | Cingulate and paracingulate gyri | Cuneus | Cuneus |
| --- | --- | --- | --- | --- | --- | --- | --- | --- | --- | --- | --- | --- | --- | --- | --- | --- | --- | --- |
| Side | L | R | L | R | L | R | L | R | L | R | L | R | L | R | L | R | L | R |
| Number 1 | 6.19 | 5.67 | 11.41 | 11.68 | 9.5 | 9.59 | 11.07 | 10.66 | 11.57 | 12.21 | 9.83 | 9.55 | 9.81 | 10.53 | 9.94 | 10.57 | 11.28 | 11.28 |
| 2 | 4.81 | 4.79 | 9.39 | 9.05 | 6.93 | 7 | 8.49 | 8.29 | 9.15 | 9.26 | 7.71 | 7.66 | 7.75 | 8.13 | 7.32 | 7.66 | 8.24 | 9.04 |
| 3 | 5.03 | 4.31 | 8.55 | 8.75 | 6.9 | 7.05 | 7.98 | 7.34 | 9.27 | 9.56 | 7.42 | 6.93 | 7.55 | 7.67 | 7.5 | 7.87 | 8.26 | 8.8 |
| 4 | 5.24 | 5.13 | 9.07 | 9.02 | 7.37 | 7.41 | 8.71 | 8.36 | 9.13 | 9.68 | 8.23 | 7.78 | 7.92 | 8.23 | 8.15 | 8.49 | 9.08 | 9.8 |
| 5 | 6.62 | 6.04 | 10.35 | 9.81 | 8.47 | 9.08 | 11.24 | 10.7 | 11.03 | 10.83 | 10.61 | 10.24 | 9.32 | 9.4 | 8.85 | 9.87 | 10.3 | 11.99 |
| 6 | 5.39 | 5.36 | 8.94 | 8.83 | 7.04 | 7.27 | 9.54 | 9.22 | 10.77 | 11.8 | 8.53 | 8.66 | 8.14 | 8.97 | 7.63 | 8.18 | 9.5 | 11.27 |
| 7 | 4.98 | 4.73 | 8.79 | 8.87 | 7.18 | 7.72 | 8.93 | 8.74 | 9.63 | 10.27 | 8.22 | 8.11 | 8.1 | 8.73 | 7.8 | 8.73 | 8.93 | 10.14 |
| 8 | 6.29 | 5.95 | 11.29 | 11.4 | 9.3 | 9.29 | 11.2 | 10.73 | 11.85 | 12.55 | 10.15 | 10.08 | 10.2 | 10.77 | 9.51 | 10.31 | 11.01 | 13.08 |
| 9 | 4.63 | 4.57 | 7.21 | 7.69 | 6.4 | 7.18 | 7.54 | 7.3 | 8.55 | 8.91 | 6.67 | 6.48 | 6.98 | 7.41 | 6.87 | 7.57 | 7.81 | 8.63 |
| 10 | 6 | 5.82 | 11.3 | 10.47 | 8.72 | 8.8 | 10.24 | 9.88 | 11.59 | 12.03 | 9.64 | 9.73 | 9.55 | 9.94 | 9.27 | 9.94 | 11.19 | 12.18 |
| 11 | 4.04 | 3.83 | 6.92 | 7.35 | 5.87 | 6.42 | 6.3 | 6.06 | 6.41 | 6.27 | 5.99 | 5.81 | 5.81 | 6.09 | 6.15 | 6.87 | 6.47 | 6.92 |
| 12 | 4.49 | 4.01 | 6.9 | 6.5 | 6.34 | 6.56 | 7.22 | 6.96 | 6.74 | 6.68 | 6.4 | 6.46 | 5.99 | 6.2 | 6.61 | 7.2 | 6.66 | 6.76 |
| 13 | 5.69 | 5.95 | 10.83 | 10.44 | 8.15 | 8.29 | 11.19 | 10.71 | 11.37 | 11.37 | 10.32 | 10.38 | 9.71 | 9.78 | 9.45 | 10.18 | 11.58 | 12.69 |
| 14 | 5.15 | 4.65 | 7.35 | 7.6 | 6.44 | 6.57 | 8.1 | 7.81 | 8.82 | 9.49 | 7.38 | 7.25 | 7.22 | 7.26 | 6.97 | 7.36 | 7.21 | 8.39 |
| 15 | 6.82 | 6.23 | 12.4 | 12.64 | 9.4 | 9.88 | 10.28 | 10.31 | 13.06 | 13.41 | 8.97 | 9.45 | 9.88 | 10.59 | 9.9 | 11.02 | 12.29 | 13.98 |
| 16 | 4.82 | 4.67 | 6.96 | 7.01 | 5.99 | 6.07 | 7.19 | 7.07 | 7.57 | 7.43 | 6.71 | 6.62 | 6.52 | 6.6 | 6.53 | 6.9 | 7.46 | 7.58 |
| 17 | 5.42 | 5.2 | 9.5 | 9.16 | 8.16 | 8.48 | 9.02 | 8.4 | 10.63 | 10.81 | 8.32 | 7.97 | 8.35 | 8.59 | 8.3 | 8.84 | 9.37 | 9.7 |
| 18 | 4.69 | 4.32 | 7.13 | 7.4 | 5.77 | 6.1 | 6.68 | 6.48 | 7.21 | 7.01 | 5.72 | 5.58 | 6.11 | 6.65 | 6.2 | 6.74 | 7.01 | 6.96 |
| 19 | 4.64 | 4.61 | 6.92 | 6.88 | 6.66 | 6.72 | 7.14 | 6.84 | 7.6 | 7.1 | 6.4 | 6.27 | 6.51 | 6.69 | 6.86 | 7.25 | 7.35 | 7.6 |
| 20 | 4.94 | 4.96 | 7.45 | 7.26 | 6.27 | 6.6 | 7.94 | 7.47 | 9.27 | 9.53 | 7.4 | 7.08 | 7.01 | 7.15 | 6.87 | 7.25 | 8.18 | 9.11 |
| 21 | 5.97 | 5.54 | 9.3 | 8.54 | 7.96 | 8.16 | 10.5 | 10.2 | 10.01 | 10.99 | 9.92 | 9.86 | 8.95 | 9.25 | 8.6 | 9.1 | 9.35 | 10.65 |

| Region of Interest | Inferior parietal, but supramarginal and angular gyri | Inferior parietal, but supramarginal and angular gyri | Inferior temporal gyrus | Inferior temporal gyrus | Insula | Insula | Lenticular nucleus, pallidum | Lenticular nucleus, pallidum | Lenticular nucleus, putamen | Lenticular nucleus, putamen | Lingual gyrus | Lingual gyrus | Mesial temporal lobe | Mesial temporal lobe | Middle cingulate and paracingulate gyri | Middle cingulate and paracingulate gyri | Middle frontal gyrus | Middle frontal gyrus |
| --- | --- | --- | --- | --- | --- | --- | --- | --- | --- | --- | --- | --- | --- | --- | --- | --- | --- | --- |
| Side | L | R | L | R | L | R | L | R | L | R | L | R | L | R | L | R | L | R |
| Number 1 | 10.73 | 11.54 | 8.77 | 9.58 | 9.49 | 9.38 | 8.35 | 7.78 | 13.04 | 12.46 | 10.74 | 11.67 | 6.5 | 6.45 | 10.43 | 11.5 | 10.69 | 11.44 |
| 2 | 8.6 | 8.4 | 7.3 | 7.49 | 7.51 | 7.36 | 6.62 | 6.64 | 9.77 | 9.31 | 7.87 | 8.08 | 5.5 | 5.41 | 7.61 | 8.34 | 8.55 | 9.17 |
| 3 | 8.22 | 8.44 | 6.99 | 6.88 | 7.76 | 6.95 | 6.72 | 6.26 | 8.88 | 8 | 8.34 | 8.34 | 5.34 | 5.19 | 7.94 | 8.61 | 8.37 | 8.94 |
| 4 | 8.45 | 8.64 | 7.42 | 7.83 | 7.56 | 7.44 | 6.86 | 6.51 | 9.7 | 9.38 | 8.98 | 8.4 | 5.52 | 5.9 | 8.76 | 9.32 | 8.39 | 9.05 |
| 5 | 10.25 | 11.49 | 9.04 | 9.37 | 8.61 | 7.96 | 7.39 | 6.54 | 12.96 | 12.22 | 10.29 | 9.73 | 6.64 | 6.66 | 9.29 | 10.71 | 9.46 | 10.73 |
| 6 | 8.38 | 9.04 | 7.43 | 7.88 | 7.61 | 7.5 | 7.33 | 6.63 | 11.15 | 10.42 | 9.17 | 8.35 | 5.8 | 5.82 | 7.81 | 8.98 | 8.92 | 9.69 |
| 7 | 9.22 | 9.36 | 7.15 | 7.6 | 7.97 | 8.51 | 7.08 | 6.89 | 10.14 | 9.81 | 9.82 | 9.29 | 5.6 | 5.56 | 8.29 | 9.69 | 8.45 | 9.65 |
| 8 | 11.68 | 12.12 | 10.18 | 9.8 | 10.28 | 9.77 | 7.91 | 7.23 | 13.16 | 12.27 | 11.54 | 11.47 | 6.7 | 7.01 | 10.13 | 11.54 | 11.23 | 11.89 |
| 9 | 7.83 | 7.85 | 6.31 | 6.78 | 7.12 | 6.96 | 5.91 | 5.31 | 8.83 | 8.59 | 7.6 | 7.44 | 5 | 5.09 | 7.18 | 7.94 | 7.17 | 8.17 |
| 10 | 10.27 | 10.59 | 9.13 | 9.42 | 9.78 | 9.52 | 7.74 | 6.4 | 11.53 | 10.94 | 11.72 | 10.16 | 6.56 | 6.49 | 9.48 | 10.79 | 10.26 | 10.92 |
| 11 | 6.52 | 6.96 | 5.5 | 5.82 | 5.74 | 5.57 | 4.45 | 4.2 | 7.14 | 6.78 | 6.22 | 5.99 | 4.19 | 4.02 | 6.39 | 7.39 | 6.32 | 6.96 |
| 12 | 6.63 | 7.01 | 5.08 | 5.59 | 6.6 | 6.09 | 5.63 | 4.86 | 8.48 | 7.97 | 5.75 | 5.81 | 4.7 | 4.78 | 6.76 | 7.82 | 6.42 | 7 |
| 13 | 10.9 | 11.24 | 8.56 | 8.55 | 9.11 | 8.63 | 8.16 | 6.64 | 12.9 | 12.1 | 10.48 | 10.12 | 6.62 | 6.67 | 10.36 | 11.67 | 10.54 | 11.49 |
| 14 | 7.7 | 7.44 | 6.78 | 7.14 | 7.35 | 6.87 | 6.51 | 5.95 | 9.25 | 8.83 | 8.76 | 7.84 | 5.28 | 5.14 | 7.43 | 8.14 | 7.66 | 8.22 |
| 15 | 10.92 | 12.53 | 9.75 | 10.67 | 10.06 | 9.96 | 9.36 | 8.12 | 11.8 | 11.7 | 12.48 | 12.29 | 7.05 | 7.26 | 10.26 | 12.16 | 11.89 | 12.9 |
| 16 | 7.42 | 7.58 | 6.18 | 6.48 | 6.81 | 6.61 | 6.55 | 5.77 | 7.83 | 7.84 | 7.58 | 6.75 | 5.18 | 5.06 | 7.17 | 7.76 | 7.15 | 7.68 |
| 17 | 8.36 | 9.01 | 7.72 | 8.25 | 8.41 | 7.49 | 7.64 | 6.03 | 10.09 | 9.43 | 9.82 | 9.39 | 6.17 | 6.22 | 8.5 | 9.36 | 9.44 | 10.07 |
| 18 | 6.37 | 6.97 | 5.85 | 6.51 | 6.42 | 6.44 | 5.07 | 5.04 | 8.05 | 7.71 | 6.76 | 6.91 | 4.71 | 4.96 | 6.54 | 7.45 | 6.82 | 7.49 |
| 19 | 6.98 | 7.13 | 6.05 | 6.25 | 7 | 6.9 | 5.64 | 5.15 | 8.28 | 7.83 | 6.72 | 6.48 | 4.94 | 4.85 | 7.05 | 7.85 | 6.95 | 7.45 |
| 20 | 7.96 | 7.65 | 6.46 | 6.8 | 7.24 | 6.66 | 6.18 | 5.72 | 8.98 | 8.3 | 8.52 | 7.96 | 5.55 | 5.56 | 7.33 | 7.97 | 7.28 | 7.79 |
| 21 | 9.29 | 10.25 | 7.38 | 7.6 | 9.28 | 8.99 | 8.63 | 7.9 | 11.6 | 11.12 | 9.77 | 9.73 | 6.23 | 6.44 | 9 | 9.89 | 8.63 | 9.57 |

| Region of Interest | Middle frontal gyrus, orbital part | Middle frontal gyrus, orbital part | Middle occipital gyrus | Middle occipital gyrus | Middle temporal gyrus | Middle temporal gyrus | Occipital lobe | Occipital lobe | Olfactory cortex | Olfactory cortex | Paracentral lobule | Paracentral lobule | Parahippocampal gyrus | Parahippocampal gyrus | Parietal lobe | Parietal lobe | Postcentral gyrus | Postcentral gyrus |
| --- | --- | --- | --- | --- | --- | --- | --- | --- | --- | --- | --- | --- | --- | --- | --- | --- | --- | --- |
| Side | L | R | L | R | L | R | L | R | L | R | L | R | L | R | L | R | L | R |
| Number 1 | 10.59 | 10.59 | 9.92 | 10.2 | 10.14 | 10.67 | 10.32 | 10.73 | 7.99 | 7.94 | 8.84 | 8.31 | 6.95 | 6.62 | 10.43 | 10.6 | 9.5 | 10.56 |
| 2 | 9.21 | 9.16 | 8.01 | 7.81 | 8.17 | 8.72 | 8.03 | 8.15 | 6.14 | 5.67 | 7.43 | 7.58 | 5.75 | 5.63 | 8.21 | 8.09 | 7.55 | 7.99 |
| 3 | 8.43 | 8.59 | 7.63 | 8.02 | 7.9 | 8.08 | 7.93 | 8.13 | 6.43 | 6.32 | 7.35 | 6.66 | 5.48 | 5.63 | 7.68 | 7.66 | 7.45 | 7.58 |
| 4 | 8.86 | 8.45 | 8.32 | 8.27 | 7.75 | 8.12 | 8.44 | 8.42 | 6.55 | 7.31 | 7.49 | 7.8 | 5.62 | 6.27 | 8.31 | 8.23 | 8.09 | 8.11 |
| 5 | 10.11 | 9.71 | 9.82 | 9.91 | 9.55 | 10.26 | 9.86 | 9.87 | 6.94 | 7.85 | 8.32 | 8.74 | 6.98 | 6.98 | 9.97 | 9.96 | 9.38 | 9.13 |
| 6 | 8.44 | 8.4 | 9.25 | 9.56 | 8.44 | 8.42 | 9.15 | 9.44 | 7.19 | 6.77 | 7.88 | 7.91 | 5.86 | 5.92 | 8.17 | 8.24 | 7.83 | 8.79 |
| 7 | 8.32 | 7.96 | 8.69 | 8.81 | 7.9 | 8.3 | 8.89 | 9.25 | 6.44 | 7.15 | 8.4 | 7.83 | 6.09 | 6.17 | 8.68 | 8.31 | 8.11 | 8.35 |
| 8 | 11.37 | 10.55 | 10.9 | 11.09 | 10.42 | 10.68 | 10.91 | 11.33 | 8.87 | 8.55 | 10.2 | 9.26 | 6.98 | 7.44 | 10.86 | 10.44 | 9.82 | 10.38 |
| 9 | 7.29 | 6.97 | 7.22 | 7.54 | 6.74 | 7.24 | 7.45 | 7.64 | 5.25 | 5.66 | 6.65 | 6.16 | 5.16 | 5.3 | 7.29 | 7.41 | 6.94 | 7.21 |
| 10 | 10.13 | 10.26 | 10.42 | 10.24 | 10.1 | 10.27 | 10.76 | 10.67 | 8.16 | 7.8 | 8.97 | 8.89 | 6.51 | 6.93 | 10.16 | 10.05 | 9.61 | 9.6 |
| 11 | 6.71 | 6.24 | 5.87 | 6.07 | 5.95 | 6.1 | 6 | 6.04 | 5.17 | 5.19 | 5.79 | 6.25 | 4.14 | 4.02 | 6.43 | 6.58 | 5.59 | 6 |
| 12 | 6.85 | 6.26 | 5.36 | 5.74 | 5.94 | 6.04 | 5.75 | 5.83 | 5.24 | 5.55 | 5.8 | 6 | 4.78 | 5.04 | 6.44 | 6.27 | 5.66 | 5.95 |
| 13 | 10.33 | 9.91 | 9.63 | 10.24 | 9.92 | 9.92 | 10.19 | 10.45 | 7.66 | 7.5 | 10.06 | 9.34 | 6.93 | 7.09 | 10.49 | 10.02 | 9.45 | 9.28 |
| 14 | 8.2 | 7.56 | 8.01 | 7.68 | 7.37 | 7.04 | 8.02 | 7.99 | 5.88 | 5.95 | 6.71 | 5.81 | 5.07 | 5.17 | 7.3 | 7.09 | 6.92 | 6.87 |
| 15 | 11.51 | 10.44 | 11.8 | 11.71 | 11.24 | 11.59 | 11.68 | 11.96 | 8.54 | 8.43 | 9.46 | 9.37 | 7.26 | 7.77 | 10.78 | 11.11 | 9.59 | 10.26 |
| 16 | 7.36 | 7 | 7.15 | 7.47 | 6.74 | 6.85 | 7.14 | 7.09 | 5.69 | 5.63 | 6.56 | 6.46 | 5.57 | 5.31 | 7.08 | 6.92 | 6.39 | 6.42 |
| 17 | 9.11 | 8.92 | 8.74 | 8.89 | 8.22 | 8.36 | 9.31 | 9.39 | 7.15 | 7.49 | 7.94 | 7.66 | 6.6 | 6.81 | 8.48 | 8.43 | 8.07 | 8.48 |
| 18 | 7.2 | 7.04 | 6.06 | 6.35 | 5.94 | 6.67 | 6.49 | 6.52 | 5.82 | 5.61 | 6.3 | 5.81 | 4.89 | 5.26 | 6.32 | 6.5 | 5.86 | 6.52 |
| 19 | 7.52 | 7.09 | 6.59 | 6.6 | 6.69 | 6.76 | 6.79 | 6.63 | 6.05 | 5.96 | 5.84 | 6.18 | 4.89 | 4.85 | 6.82 | 6.9 | 6.38 | 6.59 |
| 20 | 7.35 | 7.39 | 7.54 | 7.74 | 7.18 | 7.18 | 7.98 | 8.03 | 5.85 | 5.85 | 6.49 | 6.87 | 5.58 | 5.66 | 7.51 | 7.21 | 6.91 | 7.04 |
| 21 | 8.58 | 8.96 | 8.9 | 9.07 | 8.39 | 8.76 | 9.05 | 9.39 | 7.31 | 7.56 | 8.44 | 7.54 | 6.43 | 6.97 | 9.13 | 8.95 | 8.71 | 9.04 |

| Region of Interest | Posterior cingulate and paracingulate gyri | Posterior cingulate and paracingulate gyri | Precentral gyrus | Precentral gyrus | Precuneus | Precuneus | Rolandic operculum | Rolandic operculum | Superior frontal gyrus, dorsolateral | Superior frontal gyrus, dorsolateral | Superior frontal gyrus, medial | Superior frontal gyrus, medial | Superior frontal gyrus, medial orbital | Superior frontal gyrus, medial orbital | Superior frontal gyrus, orbital part | Superior frontal gyrus, orbital part | Superior occipital gyrus | Superior occipital gyrus |
| --- | --- | --- | --- | --- | --- | --- | --- | --- | --- | --- | --- | --- | --- | --- | --- | --- | --- | --- |
| Side | L | R | L | R | L | R | L | R | L | R | L | R | L | R | L | R | L | R |
| Number 1 | 9.23 | 8.3 | 9.93 | 10.5 | 10.49 | 10.54 | 10.61 | 10.5 | 10.65 | 10.4 | 10.03 | 10.39 | 10.41 | 10.47 | 10.58 | 10.59 | 9.49 | 9.48 |
| 2 | 7.24 | 5.76 | 7.69 | 8.27 | 8.35 | 8.46 | 8.73 | 8.19 | 7.76 | 8 | 7.89 | 8.05 | 8.72 | 8.39 | 8.98 | 8.81 | 7.77 | 8.14 |
| 3 | 7.51 | 6.24 | 7.56 | 7.77 | 7.86 | 7.77 | 7.87 | 7.65 | 8 | 7.46 | 7.72 | 7.96 | 8.14 | 7.84 | 8.16 | 8.13 | 7.35 | 7.51 |
| 4 | 7.94 | 7.27 | 7.6 | 8.24 | 8.86 | 8.38 | 8.42 | 8.53 | 8.1 | 7.79 | 7.61 | 7.64 | 8.29 | 7.81 | 8.21 | 8.26 | 7.71 | 7.31 |
| 5 | 8.21 | 7.46 | 9 | 9.7 | 10.56 | 10.36 | 10.2 | 9.45 | 8.98 | 8.78 | 9.28 | 9.83 | 10.12 | 9.81 | 9.43 | 9.76 | 9.01 | 8.72 |
| 6 | 8.65 | 6.55 | 8.3 | 9.22 | 8.28 | 8.49 | 8.75 | 8.83 | 8.2 | 8.37 | 8.36 | 8.72 | 9.01 | 8.73 | 8.25 | 9.01 | 8.43 | 8.96 |
| 7 | 7.63 | 6.36 | 7.98 | 9.08 | 8.75 | 8.33 | 8.46 | 8.93 | 8 | 8.25 | 8.21 | 8.3 | 8.03 | 8.09 | 8.21 | 8.72 | 8.14 | 8.71 |
| 8 | 7.54 | 6.27 | 10.26 | 11.04 | 10.57 | 10.01 | 11.43 | 11.2 | 10.76 | 10.44 | 10.1 | 10.44 | 11.4 | 11.1 | 10.92 | 10.43 | 9.05 | 10.82 |
| 9 | 6.97 | 6.69 | 6.97 | 7.67 | 7.4 | 7.47 | 7.15 | 7.3 | 6.74 | 7.4 | 7.04 | 7.16 | 7.48 | 7.47 | 6.67 | 7.35 | 7.03 | 7.16 |
| 10 | 10.04 | 8.76 | 9.23 | 10.28 | 10.49 | 10.34 | 10.41 | 10.08 | 9.65 | 10.04 | 9.54 | 9.94 | 10.11 | 10.35 | 10.47 | 10.1 | 9.42 | 10.22 |
| 11 | 5.97 | 5.16 | 5.92 | 6.26 | 6.66 | 6.72 | 6.26 | 5.91 | 6.3 | 6.48 | 6.31 | 6.38 | 6.62 | 6.26 | 6.55 | 6.49 | 5.66 | 5.89 |
| 12 | 6.79 | 5.61 | 6.1 | 6.23 | 6.79 | 6.53 | 6.88 | 6.84 | 5.81 | 6.09 | 6.02 | 6.15 | 7.05 | 6.87 | 6.52 | 6.75 | 5.51 | 5.46 |
| 13 | 9.56 | 7.8 | 9.7 | 10.03 | 10.91 | 10.59 | 10.79 | 10.58 | 9.64 | 9.58 | 9.81 | 9.83 | 10.45 | 10.15 | 10.43 | 9.93 | 9.26 | 9.86 |
| 14 | 6.61 | 5.37 | 7.29 | 7.53 | 7.24 | 6.94 | 8.12 | 7.69 | 7.02 | 7.08 | 6.71 | 6.79 | 7.21 | 7.09 | 7.28 | 7.43 | 7.5 | 7.81 |
| 15 | 9.93 | 8.05 | 9.87 | 10.76 | 11.1 | 10.84 | 11.07 | 11.12 | 10.72 | 10.55 | 10.83 | 11.11 | 11.61 | 11.41 | 12.05 | 11.29 | 10.2 | 10.66 |
| 16 | 5.44 | 4.52 | 6.59 | 6.9 | 7.28 | 7.11 | 6.8 | 6.37 | 6.81 | 6.76 | 6.63 | 6.53 | 7.12 | 6.51 | 7.05 | 7.25 | 6.27 | 6.78 |
| 17 | 7.92 | 6.85 | 8.59 | 8.8 | 8.7 | 8.43 | 8.58 | 8.42 | 8.77 | 8.53 | 8.82 | 8.84 | 9.33 | 9 | 9.37 | 8.82 | 8.54 | 8.88 |
| 18 | 6.04 | 4.64 | 6.18 | 6.69 | 6.5 | 6.46 | 6.81 | 6.92 | 6.59 | 6.41 | 6.45 | 6.77 | 6.83 | 6.78 | 7.27 | 6.67 | 5.84 | 6.05 |
| 19 | 6.68 | 5.41 | 6.55 | 6.88 | 7.13 | 7.27 | 6.83 | 6.49 | 6.73 | 6.78 | 6.63 | 6.86 | 7.64 | 7.18 | 7.14 | 7.09 | 6.37 | 6.51 |
| 20 | 6.77 | 5.09 | 6.99 | 7.2 | 7.63 | 7.38 | 7.46 | 7.34 | 6.8 | 6.93 | 6.95 | 7.11 | 7.39 | 7.86 | 7.32 | 7.17 | 7.35 | 7.45 |
| 21 | 8.88 | 7.54 | 8.85 | 9.38 | 9.68 | 9.18 | 10.24 | 9.52 | 8.35 | 8.32 | 7.92 | 8.29 | 9.51 | 9.14 | 8.57 | 8.83 | 8.48 | 9.26 |

| Region of Interest | Superior parietal gyrus | Superior parietal gyrus | Superior temporal gyrus | Superior temporal gyrus | Supplementary motor area | Supplementary motor area | Supramarginal gyrus | Supramarginal gyrus | Temporal lobe | Temporal lobe | Temporal pole: middle temporal gyrus | Temporal pole: middle temporal gyrus | Temporal pole: superior temporal gyrus | Temporal pole: superior temporal gyrus | Thalamus | Thalamus |
| --- | --- | --- | --- | --- | --- | --- | --- | --- | --- | --- | --- | --- | --- | --- | --- | --- |
| Side | L | R | L | R | L | R | L | R | L | R | L | R | L | R | L | R |
| Number 1 | 9.72 | 9.62 | 10.82 | 10.71 | 10.98 | 10.69 | 9.92 | 10.19 | 9.91 | 10.4 | 7.41 | 7.85 | 6.21 | 8 | 10.65 | 10.66 |
| 2 | 7.04 | 6.99 | 8.5 | 8.14 | 7.74 | 7.64 | 7.88 | 7.64 | 8.02 | 8.22 | 6.13 | 6.14 | 5.74 | 5.76 | 8.65 | 8.96 |
| 3 | 6.44 | 6.51 | 8.22 | 7.82 | 8.27 | 7.68 | 7.35 | 7.29 | 7.74 | 7.67 | 5.53 | 6.22 | 5.43 | 5.6 | 8.77 | 8.83 |
| 4 | 7.14 | 7.25 | 8.44 | 8.4 | 8.23 | 8.09 | 7.72 | 8.09 | 7.83 | 8.12 | 6.45 | 6.89 | 5.68 | 6.07 | 8.85 | 9.26 |
| 5 | 8.95 | 9.21 | 9.95 | 9.82 | 9.53 | 9.64 | 9.07 | 9.24 | 9.53 | 9.89 | 7.9 | 7.58 | 7.45 | 7.45 | 10.06 | 10.22 |
| 6 | 7.5 | 7.24 | 8.71 | 8.77 | 9.11 | 9 | 7.82 | 7.91 | 8.26 | 8.41 | 6.42 | 7.17 | 6.28 | 6.38 | 9.19 | 9.52 |
| 7 | 8.06 | 7.43 | 8.13 | 7.85 | 8.75 | 8.77 | 8.36 | 8.08 | 7.79 | 8.02 | 5.9 | 6.31 | 5.75 | 6.2 | 9.09 | 9.11 |
| 8 | 10.25 | 9.18 | 10.48 | 10.59 | 10.49 | 11.25 | 10.66 | 10.54 | 10.42 | 10.4 | 7.9 | 7.54 | 8.01 | 7.16 | 11.31 | 11.67 |
| 9 | 6.72 | 6.67 | 7.18 | 7.16 | 7.07 | 7.15 | 6.89 | 7.59 | 6.74 | 7.12 | 5.14 | 5.72 | 4.75 | 5.06 | 7.57 | 7.6 |
| 10 | 8.7 | 9 | 10.46 | 9.9 | 10.09 | 9.78 | 10.38 | 10 | 9.93 | 9.96 | 7.58 | 7.86 | 6.9 | 6.6 | 9.34 | 9.48 |
| 11 | 5.9 | 5.87 | 5.91 | 5.87 | 6.32 | 6.82 | 6.05 | 6.22 | 5.82 | 5.97 | 4.44 | 4.66 | 3.85 | 4.43 | 6.33 | 6.47 |
| 12 | 5.53 | 5.26 | 6.18 | 5.93 | 6.26 | 6.61 | 6.12 | 6.28 | 5.78 | 5.91 | 4.6 | 4.79 | 5.22 | 4.94 | 6.82 | 6.8 |
| 13 | 9.24 | 8.28 | 10.08 | 9.82 | 10.12 | 10.25 | 10.28 | 9.86 | 9.61 | 9.49 | 6.99 | 7.43 | 6.37 | 6.8 | 10.85 | 11.28 |
| 14 | 6.99 | 6.68 | 7.32 | 7.38 | 7.43 | 6.28 | 7.13 | 7.11 | 7.24 | 7.2 | 5.44 | 5.46 | 5.04 | 5.43 | 8.4 | 8.39 |
| 15 | 9.48 | 9.54 | 10.31 | 11.18 | 10.4 | 10.67 | 10.26 | 11 | 10.63 | 11.24 | 8.24 | 9.11 | 7.77 | 7.9 | 10.68 | 11.6 |
| 16 | 6.87 | 6.52 | 6.96 | 6.59 | 7.1 | 6.61 | 6.29 | 6.54 | 6.64 | 6.7 | 5.27 | 5.56 | 4.83 | 5 | 7.02 | 7.33 |
| 17 | 7.95 | 7.75 | 8.68 | 8.69 | 8.84 | 8.55 | 8.02 | 8.18 | 8.21 | 8.46 | 6.77 | 6.83 | 6.32 | 6.37 | 9.03 | 9.13 |
| 18 | 5.68 | 5.75 | 6.52 | 6.55 | 6.46 | 7.05 | 6.05 | 6.29 | 6.07 | 6.62 | 5.44 | 5.21 | 4.51 | 5.13 | 6.79 | 6.8 |
| 19 | 6.32 | 6.23 | 7.14 | 6.78 | 6.59 | 7.08 | 6.37 | 6.89 | 6.64 | 6.65 | 5.44 | 5.76 | 5.31 | 5.4 | 7.02 | 7.18 |
| 20 | 7.13 | 6.67 | 7.47 | 6.96 | 7.47 | 7.25 | 6.95 | 7.19 | 7.08 | 7.03 | 5.74 | 6.12 | 5.43 | 5.75 | 8.29 | 8.28 |
| 21 | 8.16 | 7.97 | 10.02 | 9.72 | 8.51 | 8.59 | 8.71 | 9.15 | 8.58 | 8.8 | 6.65 | 7.42 | 7.88 | 7.09 | 9.88 | 10 |

Abbreviation: SUVmean: mean standardized uptake value; AAL: automated anatomical labelling.

Supplementary Table 6 The SUV_max_ of normal 19-44 years old group according to AAL standards.

| Region of Interest | Amygdala | Amygdala | Angular gyrus | Angular gyrus | Anterior cingulate and paracingulate gyri | Anterior cingulate and paracingulate gyri | Basal ganglia | Basal ganglia | Calcarine fissure and surrounding cortex | Calcarine fissure and surrounding cortex | Caudate nucleus | Caudate nucleus | Central region | Central region | Cingulate and paracingulate gyri | Cingulate and paracingulate gyri | Cuneus | Cuneus |
| --- | --- | --- | --- | --- | --- | --- | --- | --- | --- | --- | --- | --- | --- | --- | --- | --- | --- | --- |
| Side | L | R | L | R | L | R | L | R | L | R | L | R | L | R | L | R | L | R |
| Number 1 | 8.96 | 8.56 | 18.97 | 19.84 | 16.15 | 17.12 | 23.44 | 22.34 | 22.86 | 23.17 | 22.34 | 22.34 | 23.88 | 20.19 | 20.6 | 20.58 | 20.82 | 22.62 |
| 2 | 7.38 | 7.75 | 14.51 | 15.02 | 13.35 | 13.55 | 16.39 | 15.74 | 14.92 | 14.83 | 16.39 | 15.06 | 16.1 | 15.34 | 18.72 | 18.1 | 15.87 | 15.21 |
| 3 | 7.7 | 6.61 | 14.31 | 14.62 | 12.98 | 14.07 | 15.29 | 13.5 | 17.66 | 18.07 | 15.29 | 12.96 | 16.93 | 16.65 | 16.37 | 15.85 | 15.99 | 14.96 |
| 4 | 7.56 | 7.93 | 16.4 | 15.89 | 12.02 | 13.95 | 15.72 | 15.57 | 16.74 | 17.97 | 14.53 | 15.57 | 17.07 | 16.56 | 16.66 | 16.8 | 19.69 | 17.19 |
| 5 | 8.96 | 8.53 | 18.25 | 19.63 | 15.76 | 17.04 | 21.33 | 22.34 | 19.02 | 19.61 | 21.33 | 22.34 | 22.32 | 19.36 | 18.63 | 19.32 | 19.71 | 21.91 |
| 6 | 7.85 | 7.48 | 15.03 | 16.35 | 13.19 | 13.81 | 17.18 | 17.89 | 21.26 | 21.52 | 16.01 | 17.89 | 17.98 | 19.17 | 17.48 | 17.83 | 17.77 | 21.2 |
| 7 | 7.67 | 6.96 | 15.62 | 14.65 | 14.82 | 15.57 | 15.83 | 17.11 | 20.43 | 20.1 | 15.74 | 17.11 | 17.35 | 18.6 | 17.85 | 17.67 | 16.94 | 16.85 |
| 8 | 9.41 | 11.12 | 20.27 | 22.83 | 16.62 | 17.62 | 23.54 | 21.37 | 25.83 | 25.48 | 23.54 | 21.37 | 28.15 | 24.88 | 21.29 | 22.26 | 19.72 | 21.09 |
| 9 | 6.35 | 6.3 | 13.52 | 14.22 | 13.1 | 11.84 | 14.35 | 14.24 | 15.19 | 14.36 | 13.05 | 14.24 | 15.71 | 14.79 | 13.1 | 13.58 | 13.95 | 15.06 |
| 10 | 8.99 | 9.56 | 19.29 | 19.31 | 15.61 | 14.39 | 19.22 | 19.85 | 24.86 | 23.99 | 18.34 | 19.85 | 19.91 | 18.43 | 18.4 | 18.71 | 21.37 | 23.64 |
| 11 | 5.7 | 5.4 | 12.32 | 11.2 | 9.66 | 10.43 | 11.2 | 10.55 | 10.66 | 11.59 | 11.2 | 10.32 | 12.95 | 12.63 | 15.37 | 11.73 | 12.54 | 13.98 |
| 12 | 6.14 | 6.17 | 11.32 | 11.19 | 11.26 | 11.39 | 13.73 | 13.45 | 12.63 | 12.72 | 11.69 | 12.74 | 13.34 | 12.59 | 15.01 | 15.18 | 13.53 | 13.79 |
| 13 | 8.85 | 8.34 | 18.35 | 18.66 | 16.34 | 18.06 | 19.98 | 20.3 | 19.71 | 19.53 | 19.98 | 20.3 | 20.59 | 20.46 | 21.79 | 21.83 | 22.32 | 22.2 |
| 14 | 7.68 | 6.91 | 14.32 | 13.63 | 12.86 | 12.51 | 14.99 | 15.47 | 18.23 | 20.61 | 14.99 | 14.53 | 17.11 | 16.8 | 13.32 | 13.63 | 16.29 | 16.06 |
| 15 | 10.62 | 9.47 | 22.05 | 24.41 | 18.34 | 20 | 19.33 | 20.06 | 25.29 | 28.4 | 19.33 | 20.06 | 21.91 | 22.7 | 22.33 | 21.32 | 23.65 | 24.82 |
| 16 | 6.9 | 6.53 | 11.72 | 11.97 | 10.74 | 11.41 | 13.08 | 12.22 | 14 | 14.46 | 13.08 | 11.45 | 13.07 | 14.31 | 12.41 | 13.73 | 15.08 | 13.15 |
| 17 | 7.98 | 7.79 | 16.51 | 16.12 | 13.45 | 13.29 | 16.72 | 15.92 | 20.28 | 23.47 | 15.81 | 15.03 | 18.61 | 16.5 | 15.87 | 16.2 | 18.42 | 18.43 |
| 18 | 7.03 | 7.04 | 11.35 | 11.89 | 11.09 | 10.64 | 13.04 | 12.53 | 13.2 | 12.15 | 13.04 | 12.53 | 13.13 | 12.67 | 13.71 | 13.75 | 11.71 | 12.04 |
| 19 | 6.17 | 6.56 | 11.81 | 11.58 | 11.44 | 10.92 | 13.69 | 12.18 | 12.57 | 12.06 | 13.1 | 11.9 | 12.85 | 12.2 | 13.01 | 12.71 | 12.84 | 13.15 |
| 20 | 6.69 | 6.43 | 12.88 | 13.2 | 11.7 | 10.85 | 13.01 | 12.49 | 16.66 | 18.88 | 12.1 | 12.49 | 13.88 | 13.8 | 15.02 | 16.65 | 13.83 | 16.13 |
| 21 | 7.99 | 8.59 | 17.05 | 16.14 | 15.36 | 14.45 | 20.06 | 18.82 | 18.79 | 20.52 | 20.06 | 18.61 | 22.42 | 18.64 | 19.14 | 19.7 | 18.75 | 21.06 |

| Region of Interest | Frontal lobe | Frontal lobe | Fusiform gyrus | Fusiform gyrus | Gyrus rectus | Gyrus rectus | Heschl gyrus | Heschl gyrus | Hippocampus | Hippocampus | Inferior frontal gyrus, opercular part | Inferior frontal gyrus, opercular part | Inferior frontal gyrus, orbital part | Inferior frontal gyrus, orbital part | Inferior frontal gyrus, triangular part | Inferior frontal gyrus, triangular part | Inferior occipital gyrus | Inferior occipital gyrus |
| --- | --- | --- | --- | --- | --- | --- | --- | --- | --- | --- | --- | --- | --- | --- | --- | --- | --- | --- |
| Side | L | R | L | R | L | R | L | R | L | R | L | R | L | R | L | R | L | R |
| Number 1 | 25.51 | 21.23 | 16.05 | 16.5 | 16.96 | 15.47 | 18.95 | 19.52 | 12.17 | 11.38 | 19.22 | 19.26 | 17.93 | 18.23 | 19.38 | 18.52 | 20.14 | 25.2 |
| 2 | 16.84 | 17.25 | 13.66 | 12.02 | 12.75 | 13.65 | 13.43 | 15.54 | 9.09 | 8.97 | 13.42 | 15.07 | 16.31 | 16.36 | 15.98 | 15.72 | 12.24 | 13.41 |
| 3 | 18.65 | 18.92 | 12.61 | 13.75 | 12.51 | 13.12 | 17.27 | 14.59 | 8.53 | 8.34 | 14.59 | 14.79 | 14.1 | 14.16 | 16.07 | 14.52 | 16.97 | 16.4 |
| 4 | 18.96 | 19.14 | 14.29 | 15.11 | 12.2 | 13.72 | 12.94 | 13.49 | 10.53 | 9.34 | 16.73 | 16.56 | 14.26 | 16.26 | 15.1 | 15.54 | 17.81 | 16.46 |
| 5 | 20.71 | 21.64 | 16.55 | 16.92 | 16.23 | 16.09 | 18.29 | 19.3 | 11.38 | 12.24 | 17.31 | 20.76 | 18.25 | 21.64 | 20.08 | 19.46 | 16.99 | 19.01 |
| 6 | 21.01 | 18.49 | 14.73 | 15.82 | 12.96 | 13.39 | 16.98 | 19.28 | 10.18 | 10.37 | 16.46 | 17.37 | 15.18 | 16.61 | 20.67 | 17.3 | 18.06 | 20.94 |
| 7 | 22.01 | 20.06 | 13.07 | 15.68 | 12.24 | 13.24 | 17.36 | 14.42 | 9.86 | 8.97 | 16.64 | 16.12 | 14.91 | 16.1 | 16.55 | 17.24 | 19.72 | 21.11 |
| 8 | 24.07 | 24.5 | 22.65 | 18.68 | 18.44 | 16.38 | 21.26 | 17.7 | 14.17 | 12.01 | 23.62 | 24.5 | 20.7 | 18.94 | 22.48 | 22.63 | 24.85 | 23.47 |
| 9 | 15.29 | 15.86 | 12.49 | 11.91 | 10.26 | 10.98 | 12.2 | 12.6 | 8.71 | 9.12 | 12.52 | 14.11 | 12.56 | 13.63 | 12.74 | 14.14 | 14.49 | 12.61 |
| 10 | 19.67 | 22.64 | 18.83 | 18.82 | 15.64 | 16 | 17.6 | 20.87 | 11.85 | 11.56 | 18.55 | 17.33 | 19.67 | 16.93 | 17.96 | 18.82 | 22.04 | 20.52 |
| 11 | 12.86 | 13.68 | 9.47 | 9.8 | 8.79 | 9.43 | 9.56 | 10.1 | 6.67 | 6.51 | 12.15 | 12.93 | 12.04 | 11.36 | 11.59 | 11.87 | 10.92 | 11.23 |
| 12 | 13.35 | 13.73 | 7.89 | 9.34 | 9.7 | 9.32 | 12.67 | 11.02 | 6.99 | 7.93 | 11.69 | 11.44 | 11.28 | 11.28 | 11.7 | 11.71 | 8.6 | 8.68 |
| 13 | 21.07 | 22.12 | 15.34 | 16.37 | 15.59 | 15.13 | 20.54 | 18.99 | 13.03 | 11.85 | 18.72 | 19.16 | 18.92 | 19.9 | 18.55 | 18.68 | 17.89 | 19.53 |
| 14 | 16.71 | 20.14 | 13.45 | 13.82 | 11.5 | 12.2 | 13.72 | 11.88 | 11.65 | 9.63 | 15.91 | 16.02 | 13.81 | 14.97 | 16.1 | 15.77 | 16.81 | 17.66 |
| 15 | 24.97 | 24.2 | 19.35 | 20.12 | 18.1 | 17.21 | 19.76 | 21.55 | 13.61 | 13.36 | 20.83 | 21.37 | 22.18 | 22.23 | 23.71 | 23.21 | 22.81 | 26.53 |
| 16 | 14.31 | 13.6 | 11.01 | 11.64 | 10.28 | 9.86 | 12.48 | 11.79 | 9.14 | 8.61 | 12.8 | 12.25 | 11.92 | 12.07 | 13.51 | 12.18 | 13.46 | 14.73 |
| 17 | 18.39 | 18.76 | 15.44 | 15.45 | 14.21 | 12.76 | 14.54 | 16.5 | 11.38 | 11.35 | 16.31 | 15.87 | 16.34 | 15.55 | 17.86 | 16.3 | 19.33 | 19.08 |
| 18 | 14.43 | 13.92 | 10.21 | 9.75 | 11.5 | 9.71 | 10.07 | 12.56 | 7.76 | 7.5 | 12.79 | 12.97 | 11.42 | 11.97 | 12.87 | 12.74 | 12.62 | 11.81 |
| 19 | 14.44 | 14.08 | 10.32 | 9.97 | 11.41 | 10.48 | 12.77 | 11.78 | 8.12 | 8.7 | 12.62 | 12.29 | 12.16 | 12.58 | 12.45 | 12.39 | 11.11 | 11.65 |
| 20 | 14.5 | 13.71 | 11.97 | 13.37 | 9.89 | 9.58 | 13.43 | 11.64 | 8.92 | 9.95 | 13.2 | 12.9 | 13.44 | 11.18 | 14.5 | 13.05 | 15 | 17.11 |
| 21 | 21.25 | 19.3 | 15.49 | 16.53 | 15.57 | 15.09 | 22.61 | 20.33 | 12.97 | 11.86 | 16.05 | 17.22 | 16.74 | 16.07 | 17.72 | 16.82 | 20.89 | 19.55 |

| Region of Interest | Inferior parietal, but supramarginal and angular gyri | Inferior parietal, but supramarginal and angular gyri | Inferior temporal gyrus | Inferior temporal gyrus | Insula | Insula | Lenticular nucleus, pallidum | Lenticular nucleus, pallidum | Lenticular nucleus, putamen | Lenticular nucleus, putamen | Lingual gyrus | Lingual gyrus | Mesial temporal lobe | Mesial temporal lobe | Middle cingulate and paracingulate gyri | Middle cingulate and paracingulate gyri | Middle frontal gyrus | Middle frontal gyrus |
| --- | --- | --- | --- | --- | --- | --- | --- | --- | --- | --- | --- | --- | --- | --- | --- | --- | --- | --- |
| Side | L | R | L | R | L | R | L | R | L | R | L | R | L | R | L | R | L | R |
| Number 1 | 18.54 | 18.77 | 16.77 | 17.66 | 17.58 | 16.59 | 14.28 | 13.68 | 23.44 | 21.44 | 19.8 | 23.84 | 13.09 | 13.6 | 20.6 | 20.58 | 20.12 | 21.03 |
| 2 | 16.44 | 14.5 | 13.38 | 12.53 | 16.04 | 14.18 | 11.15 | 10.91 | 13.96 | 15.74 | 14.51 | 13.73 | 9.09 | 9.02 | 15.67 | 18.1 | 16.84 | 17.25 |
| 3 | 16.05 | 14.08 | 11.56 | 13.04 | 14.44 | 13.47 | 12.19 | 10.32 | 14.61 | 13.5 | 17.52 | 17.29 | 10 | 9.85 | 16.37 | 15.85 | 17.75 | 16.43 |
| 4 | 16.95 | 14.57 | 13.82 | 15.06 | 13.47 | 13.61 | 12.48 | 10.63 | 15.72 | 14.51 | 17.51 | 17.77 | 10.64 | 10.72 | 16.66 | 16.8 | 18.3 | 19.14 |
| 5 | 19.83 | 20.23 | 16.79 | 18 | 17.73 | 18.3 | 13.3 | 9.86 | 20.4 | 21.8 | 17.01 | 17.9 | 11.96 | 12.24 | 18.63 | 19.32 | 19.25 | 20.09 |
| 6 | 14.91 | 15.03 | 14.83 | 14.94 | 15.61 | 15.4 | 11.6 | 9.97 | 17.18 | 15.96 | 19.45 | 19.55 | 10.82 | 10.37 | 16.03 | 17.83 | 17.68 | 17.17 |
| 7 | 17.12 | 16.27 | 13.89 | 13.46 | 14.24 | 15.78 | 12.29 | 13.48 | 15.83 | 17 | 18.1 | 22.01 | 11.01 | 12.09 | 17.85 | 17.67 | 17.69 | 18.51 |
| 8 | 24.14 | 19.81 | 18.5 | 18 | 22.02 | 19.89 | 13.8 | 11.96 | 21.82 | 20.35 | 21.46 | 25.36 | 14.17 | 13.44 | 21.29 | 22.26 | 24.07 | 23.83 |
| 9 | 13.59 | 14.07 | 11.83 | 12.05 | 13.89 | 13.65 | 11.14 | 7.98 | 14.35 | 13.97 | 13.63 | 15.18 | 8.71 | 10.19 | 12.44 | 13.51 | 15.29 | 15.86 |
| 10 | 19.65 | 17.65 | 18.41 | 16.91 | 18.28 | 18.15 | 12.78 | 11.36 | 19.22 | 19.16 | 23.79 | 23.72 | 13.24 | 13.22 | 18.4 | 18.71 | 19.25 | 20.71 |
| 11 | 11.62 | 11.09 | 10.03 | 10.81 | 10.56 | 10.4 | 8.13 | 6.06 | 11.01 | 10.55 | 10.18 | 11.34 | 7.98 | 7.35 | 11.68 | 11.61 | 12.86 | 13.68 |
| 12 | 12.7 | 12.21 | 9.88 | 10.06 | 12.19 | 10.51 | 10.09 | 8.41 | 13.73 | 13.45 | 10.88 | 11.05 | 7.49 | 7.93 | 15.01 | 15.18 | 13.35 | 13.73 |
| 13 | 20.2 | 19.03 | 15.79 | 17.2 | 17.29 | 16.61 | 12.36 | 9.74 | 19.37 | 19.34 | 17.9 | 19.49 | 13.76 | 11.85 | 21.52 | 21.83 | 20.98 | 22.12 |
| 14 | 13.66 | 12.79 | 13.56 | 12.1 | 13.94 | 13.77 | 12.44 | 10.3 | 14.88 | 15.47 | 18.86 | 18.33 | 11.65 | 9.63 | 13.32 | 13.63 | 15.78 | 20.14 |
| 15 | 22.5 | 22.08 | 19.72 | 19.42 | 19.95 | 21.5 | 16.18 | 14.17 | 18.95 | 19.52 | 24.55 | 27.24 | 13.61 | 13.36 | 21.63 | 21.32 | 24.97 | 24.2 |
| 16 | 14.23 | 12.04 | 10.79 | 10.41 | 10.83 | 12.28 | 11.05 | 9.3 | 12.33 | 12.22 | 13.76 | 13.58 | 9.14 | 8.68 | 12.41 | 13.73 | 14.31 | 13.6 |
| 17 | 15.8 | 16.48 | 14.48 | 14.34 | 14.96 | 14.94 | 12.85 | 9.84 | 16.72 | 15.92 | 18.8 | 18.3 | 11.9 | 12.95 | 15.87 | 16.2 | 18.39 | 18.76 |
| 18 | 12.24 | 10.87 | 10.79 | 11.18 | 12.65 | 11.83 | 8.01 | 8.58 | 12.47 | 11.93 | 13.4 | 13.05 | 8.89 | 8.8 | 13.71 | 13.75 | 13.97 | 13.92 |
| 19 | 11.7 | 11.64 | 10.47 | 10.65 | 11.37 | 11.54 | 9.56 | 9.28 | 13.69 | 12.18 | 11.51 | 12.21 | 8.7 | 9 | 13.01 | 12.71 | 14.44 | 14.08 |
| 20 | 14.86 | 11.68 | 12.16 | 12.77 | 12.54 | 11.79 | 10.28 | 8.26 | 13.01 | 12.4 | 16.79 | 16.78 | 8.92 | 9.95 | 15.02 | 16.65 | 14.15 | 13.16 |
| 21 | 17.1 | 17.83 | 13.86 | 13.92 | 19.77 | 16.32 | 16.38 | 15.16 | 19.77 | 18.82 | 17.94 | 23.53 | 12.97 | 11.86 | 17.87 | 19.7 | 21.25 | 18.64 |

| Region of Interest | Middle frontal gyrus, orbital part | Middle frontal gyrus, orbital part | Middle occipital gyrus | Middle occipital gyrus | Middle temporal gyrus | Middle temporal gyrus | Occipital lobe | Occipital lobe | Olfactory cortex | Olfactory cortex | Paracentral lobule | Paracentral lobule | Parahippocampal gyrus | Parahippocampal gyrus | Parietal lobe | Parietal lobe | Postcentral gyrus | Postcentral gyrus |
| --- | --- | --- | --- | --- | --- | --- | --- | --- | --- | --- | --- | --- | --- | --- | --- | --- | --- | --- |
| Side | L | R | L | R | L | R | L | R | L | R | L | R | L | R | L | R | L | R |
| Number 1 | 18.77 | 17.96 | 21.52 | 18.86 | 17.3 | 18.97 | 22.86 | 25.2 | 14.01 | 13.2 | 18.61 | 17.99 | 13.09 | 13.6 | 20.84 | 21.43 | 21.61 | 19.23 |
| 2 | 16.24 | 15.4 | 13.9 | 14.97 | 14.89 | 14.7 | 15.87 | 15.21 | 9.32 | 10.09 | 14.16 | 12.84 | 8.63 | 9.02 | 16.44 | 15.16 | 14.78 | 13.56 |
| 3 | 15.36 | 14.6 | 13.7 | 15.54 | 13.97 | 14.8 | 17.66 | 18.07 | 10.91 | 9.86 | 14.75 | 11.9 | 10 | 9.85 | 16.17 | 16.4 | 15.97 | 16.03 |
| 4 | 15.02 | 15.29 | 16.51 | 16.65 | 13.66 | 15.11 | 19.69 | 17.97 | 11.89 | 13.55 | 16.54 | 15.83 | 10.64 | 10.72 | 20.72 | 17.09 | 15.78 | 16.44 |
| 5 | 18.07 | 18.82 | 19.56 | 19.7 | 19.55 | 19.25 | 19.71 | 21.91 | 13.57 | 15.26 | 16.51 | 16.82 | 11.96 | 11.54 | 19.83 | 22.79 | 22.32 | 17.8 |
| 6 | 14.29 | 15.17 | 20.2 | 20.97 | 14.83 | 15.47 | 21.26 | 21.52 | 11.19 | 10.39 | 14.67 | 15.62 | 10.82 | 10.12 | 16.93 | 16.35 | 17.03 | 16.43 |
| 7 | 15.55 | 15.11 | 20 | 17.07 | 15.21 | 15.04 | 20.43 | 22.01 | 11.07 | 12.18 | 16.53 | 16.26 | 11.01 | 12.09 | 17.12 | 16.37 | 16.79 | 16.8 |
| 8 | 20.47 | 18.69 | 24.32 | 22.4 | 19.21 | 19.09 | 25.83 | 25.48 | 14.98 | 14.24 | 17.27 | 17.19 | 13.92 | 13.44 | 24.14 | 22.83 | 19.1 | 19.99 |
| 9 | 13.37 | 12.89 | 14.13 | 14.02 | 13.27 | 14.06 | 15.19 | 15.18 | 7.75 | 8.79 | 13.9 | 12.65 | 8.53 | 10.19 | 13.59 | 16.88 | 13.37 | 14.08 |
| 10 | 18.62 | 17.05 | 25.51 | 20.48 | 18.07 | 17.2 | 25.51 | 23.99 | 14.65 | 13.73 | 16.14 | 14.48 | 13.24 | 13.22 | 22.07 | 19.9 | 19.53 | 17.71 |
| 11 | 11.84 | 11.73 | 12.17 | 10.86 | 10.34 | 10.8 | 12.54 | 13.98 | 8.39 | 8.92 | 11.68 | 11.57 | 7.98 | 7.35 | 14.64 | 13.75 | 10.74 | 10.05 |
| 12 | 11.34 | 11.36 | 11.9 | 9.46 | 10.71 | 10.36 | 13.53 | 13.79 | 9.93 | 8.63 | 11.47 | 10.1 | 7.49 | 7.84 | 12.7 | 13.01 | 13.34 | 12.59 |
| 13 | 17.65 | 18.23 | 20.35 | 19.4 | 16.85 | 18.43 | 22.32 | 22.2 | 12.74 | 12.76 | 17.95 | 16.91 | 13.76 | 11.16 | 21.44 | 20.67 | 18.64 | 18.6 |
| 14 | 14.2 | 12.54 | 17.71 | 14.98 | 13.08 | 13.06 | 18.86 | 20.61 | 10.11 | 10.58 | 12.39 | 11.05 | 8.83 | 8.64 | 15.88 | 15.42 | 14.34 | 13.73 |
| 15 | 20.72 | 21.56 | 26.89 | 22.74 | 22.92 | 21.19 | 26.89 | 28.4 | 15.02 | 15.25 | 16.32 | 16.46 | 13.07 | 13.23 | 24.88 | 24.41 | 18.02 | 19.22 |
| 16 | 12.41 | 11.64 | 13.3 | 13.39 | 11.49 | 11.03 | 15.08 | 14.73 | 8.76 | 10.22 | 11.91 | 12.31 | 9.04 | 8.68 | 14.23 | 12.69 | 12.54 | 12.84 |
| 17 | 14.67 | 14.9 | 19.65 | 15.88 | 14.5 | 14.36 | 20.28 | 23.47 | 11.49 | 11.37 | 14 | 13.88 | 11.9 | 12.95 | 16.59 | 18.11 | 15.59 | 16.47 |
| 18 | 12.15 | 12.35 | 12.84 | 11.89 | 10.53 | 11.85 | 13.4 | 13.05 | 9.72 | 8.66 | 11.55 | 10.21 | 8.89 | 8.8 | 13.83 | 12.83 | 11.12 | 11.96 |
| 19 | 11.54 | 11.26 | 11.92 | 11.19 | 11.38 | 11 | 14.14 | 13.15 | 9.24 | 9.4 | 11.94 | 10.42 | 8.7 | 9 | 12.79 | 13.49 | 12.72 | 11.62 |
| 20 | 12.77 | 11.98 | 17.49 | 17.56 | 12.11 | 12.08 | 17.49 | 18.88 | 9.16 | 8.78 | 12.72 | 11.89 | 8.49 | 8.97 | 14.86 | 15.86 | 13.7 | 12.23 |
| 21 | 16.43 | 15.09 | 19.96 | 17.04 | 17.76 | 16.17 | 20.89 | 23.53 | 12.69 | 12.94 | 17.88 | 19.3 | 11.84 | 11.78 | 20.46 | 20.01 | 20.13 | 17.99 |

| Region of Interest | Posterior cingulate and paracingulate gyri | Posterior cingulate and paracingulate gyri | Precentral gyrus | Precentral gyrus | Precuneus | Precuneus | Rolandic operculum | Rolandic operculum | Superior frontal gyrus, dorsolateral | Superior frontal gyrus, dorsolateral | Superior frontal gyrus, medial | Superior frontal gyrus, medial | Superior frontal gyrus, medial orbital | Superior frontal gyrus, medial orbital | Superior frontal gyrus, orbital part | Superior frontal gyrus, orbital part | Superior occipital gyrus | Superior occipital gyrus |
| --- | --- | --- | --- | --- | --- | --- | --- | --- | --- | --- | --- | --- | --- | --- | --- | --- | --- | --- |
| Side | L | R | L | R | L | R | L | R | L | R | L | R | L | R | L | R | L | R |
| Number 1 | 16.67 | 17.18 | 23.88 | 20.19 | 20.84 | 21.43 | 20.07 | 19.36 | 22.04 | 21.11 | 21.1 | 19.57 | 17.5 | 17.21 | 19.87 | 21.23 | 20.39 | 18.78 |
| 2 | 18.72 | 13.71 | 16.1 | 15.34 | 15.72 | 15.16 | 16.06 | 14.04 | 15.91 | 15.51 | 15.12 | 14.7 | 13.79 | 13.01 | 15.48 | 13.91 | 15.5 | 13.46 |
| 3 | 14.1 | 13.44 | 16.93 | 15.54 | 16.17 | 16.4 | 15.8 | 16.65 | 17.51 | 15.63 | 16.73 | 14.71 | 12.51 | 13.37 | 14.73 | 14.74 | 16.07 | 13.49 |
| 4 | 16.25 | 14.71 | 17.07 | 16.56 | 20.72 | 17.09 | 14.58 | 16.13 | 18.96 | 16.06 | 15.32 | 14.22 | 12.54 | 13.03 | 14.48 | 14.55 | 15.62 | 16.23 |
| 5 | 18.09 | 16.43 | 19.41 | 19.05 | 19.13 | 22.79 | 19.39 | 19.36 | 20.71 | 17.69 | 17.22 | 17.29 | 16.55 | 15.26 | 18.99 | 19.37 | 18.15 | 19.16 |
| 6 | 17.48 | 15.8 | 17.98 | 17.63 | 16.93 | 14.84 | 16.22 | 19.17 | 19 | 17.05 | 16.09 | 15.41 | 13.95 | 14.75 | 14.81 | 18.49 | 16.76 | 21.52 |
| 7 | 16.74 | 15.06 | 17.35 | 18.6 | 17.09 | 16.1 | 15.02 | 15.22 | 16.53 | 20.06 | 15.51 | 14.99 | 13.95 | 14.17 | 22.01 | 17.79 | 18.08 | 16.74 |
| 8 | 19.56 | 18.85 | 28.15 | 24.88 | 20.91 | 20.87 | 21.85 | 18.57 | 23.52 | 19.31 | 19.63 | 19.86 | 17.24 | 16.61 | 19.8 | 18.31 | 20.12 | 21.79 |
| 9 | 12.42 | 13.58 | 15.71 | 14.79 | 13.58 | 16.88 | 12.75 | 12.57 | 13.63 | 15.12 | 12.6 | 13.37 | 12.64 | 12.83 | 12.55 | 13.29 | 14.06 | 13.75 |
| 10 | 18.27 | 17.22 | 18.72 | 18.43 | 22.07 | 19.9 | 19.91 | 17.12 | 18.86 | 22.64 | 16.79 | 17.19 | 15.96 | 15.3 | 16.7 | 16.84 | 19.5 | 19.69 |
| 11 | 15.37 | 11.73 | 12.95 | 12.63 | 14.64 | 13.75 | 9.86 | 9.37 | 11.86 | 12.9 | 11.73 | 11.97 | 11.13 | 11.21 | 11.28 | 11.41 | 10.8 | 11.22 |
| 12 | 12.93 | 13.16 | 11.84 | 12.14 | 11.65 | 13.01 | 11.78 | 11.52 | 12.52 | 12.23 | 10.6 | 10.45 | 10.17 | 10.74 | 9.95 | 10.51 | 12.53 | 9.75 |
| 13 | 21.79 | 19.09 | 20.59 | 20.34 | 21.44 | 20.67 | 20.08 | 20.46 | 21.07 | 20.89 | 18.66 | 18.79 | 16.27 | 17.6 | 17.47 | 18.79 | 22.17 | 17.29 |
| 14 | 13.16 | 12.66 | 17.11 | 16.8 | 14.27 | 15.12 | 13.08 | 13.04 | 16.71 | 15.35 | 13.47 | 14.78 | 11.59 | 12.28 | 12.1 | 13.37 | 15.15 | 15.3 |
| 15 | 22.33 | 21 | 21.91 | 22.7 | 24.88 | 23.06 | 20.2 | 19.04 | 23.17 | 23.4 | 19.78 | 22.33 | 18.12 | 20.41 | 22.11 | 19.08 | 23.12 | 22.4 |
| 16 | 11.09 | 11.22 | 13.07 | 14.31 | 14.07 | 12.69 | 12.4 | 11.45 | 13.17 | 13.07 | 11.24 | 12.09 | 10.5 | 10.87 | 10.86 | 11.95 | 11.7 | 13.25 |
| 17 | 15.24 | 14.88 | 18.61 | 16.5 | 16.59 | 18.11 | 15.73 | 14.57 | 16.18 | 15.81 | 15.1 | 15.26 | 14.72 | 15.2 | 16.33 | 15 | 17.61 | 16.33 |
| 18 | 12.8 | 11.98 | 12.99 | 12.44 | 13.83 | 12.83 | 13.13 | 12.67 | 12.59 | 12.54 | 11.42 | 13.32 | 11.08 | 10.65 | 14.43 | 11.68 | 10.3 | 10.93 |
| 19 | 11.31 | 12.05 | 12.85 | 12.2 | 12.79 | 13.49 | 12.81 | 11.67 | 12.35 | 12.35 | 11.68 | 11.83 | 11.85 | 11.77 | 12 | 11.81 | 14.14 | 11.58 |
| 20 | 14.22 | 13.53 | 13.88 | 13.8 | 14.85 | 15.86 | 13.03 | 11.4 | 14.03 | 12.64 | 11.93 | 11.61 | 11.5 | 11.39 | 12.33 | 12.54 | 15.92 | 17.52 |
| 21 | 19.14 | 16.96 | 22.42 | 18.64 | 20.46 | 20.01 | 20.02 | 16.94 | 18.96 | 17.66 | 16.98 | 16.85 | 14.31 | 14.88 | 15.26 | 16.03 | 16.87 | 18.32 |

| Region of Interest | Superior parietal gyrus | Superior parietal gyrus | Superior temporal gyrus | Superior temporal gyrus | Supplementary motor area | Supplementary motor area | Supramarginal gyrus | Supramarginal gyrus | Temporal lobe | Temporal lobe | Temporal pole: middle temporal gyrus | Temporal pole: middle temporal gyrus | Temporal pole: superior temporal gyrus | Temporal pole: superior temporal gyrus | Thalamus | Thalamus |
| --- | --- | --- | --- | --- | --- | --- | --- | --- | --- | --- | --- | --- | --- | --- | --- | --- |
| Side | L | R | L | R | L | R | L | R | L | R | L | R | L | R | L | R |
| Number 1 | 17.54 | 17.07 | 22.82 | 19.75 | 25.51 | 20.01 | 17.55 | 18.64 | 22.82 | 19.75 | 13.62 | 14.01 | 15.44 | 16.31 | 17.92 | 18.97 |
| 2 | 14.5 | 12.95 | 15.32 | 14.51 | 14.58 | 16.18 | 13.74 | 13.36 | 15.32 | 15.54 | 10.72 | 11.69 | 11.42 | 11.96 | 16.51 | 15.36 |
| 3 | 14.13 | 12.85 | 17.29 | 16.1 | 18.65 | 18.92 | 14.04 | 12.89 | 17.29 | 16.1 | 11.21 | 11.28 | 10.58 | 9.92 | 14.87 | 15.49 |
| 4 | 14.63 | 14.81 | 15.09 | 16.78 | 16.67 | 14.94 | 12.51 | 14.18 | 15.09 | 16.78 | 11.5 | 11.55 | 11.77 | 11.49 | 16.54 | 15.88 |
| 5 | 17.62 | 18.04 | 18.15 | 18.64 | 17.62 | 18.03 | 15.94 | 18.45 | 19.55 | 19.3 | 14.29 | 15.11 | 15.79 | 15.06 | 17.38 | 17.27 |
| 6 | 14.95 | 14.92 | 17.12 | 17.96 | 21.01 | 16.92 | 16.37 | 14.8 | 17.12 | 19.28 | 10.94 | 12.28 | 14.77 | 13.22 | 15.96 | 16.65 |
| 7 | 14.3 | 16.37 | 13.99 | 14.95 | 16.92 | 16.65 | 15.73 | 14.65 | 17.36 | 15.04 | 11.23 | 11.45 | 13.27 | 11.31 | 18.14 | 17.74 |
| 8 | 23.96 | 19.82 | 20.61 | 19.46 | 18.53 | 22.06 | 18.6 | 18.68 | 21.26 | 19.46 | 15.72 | 14.99 | 17.4 | 17.09 | 19.39 | 19.61 |
| 9 | 12.07 | 11.88 | 12.79 | 13.16 | 13.65 | 13.89 | 12.6 | 13.78 | 13.27 | 14.06 | 9.44 | 9.93 | 10.67 | 9.34 | 12.57 | 14.04 |
| 10 | 18.72 | 18.64 | 18.02 | 18.54 | 18.41 | 16.67 | 17.22 | 16.15 | 18.41 | 20.87 | 14.64 | 14.55 | 15.1 | 15.73 | 19.14 | 16.53 |
| 11 | 12.86 | 11.39 | 9.47 | 10.46 | 11.53 | 11.95 | 9.36 | 11.22 | 10.34 | 10.81 | 7.43 | 8.41 | 8.43 | 8.78 | 11.49 | 10.09 |
| 12 | 10.97 | 9.72 | 11.43 | 10.34 | 11.01 | 11.99 | 10.78 | 10.44 | 12.67 | 11.02 | 7.81 | 9.15 | 9.27 | 9.72 | 11.02 | 11.18 |
| 13 | 18.09 | 17.26 | 19.93 | 19.97 | 18.1 | 18.09 | 18.34 | 18 | 20.54 | 19.97 | 12.22 | 13.25 | 14.31 | 13.96 | 17.95 | 17.23 |
| 14 | 15.88 | 15.42 | 14.07 | 13.06 | 14.51 | 14.14 | 12.28 | 12.3 | 14.07 | 13.06 | 9.03 | 10.14 | 9.97 | 10.58 | 15.08 | 15.1 |
| 15 | 19.14 | 20.46 | 19.83 | 21.03 | 21.38 | 21.39 | 19.39 | 20.19 | 22.92 | 21.55 | 15.07 | 16.13 | 14.09 | 14.8 | 18.95 | 20.03 |
| 16 | 12.04 | 11.88 | 11.45 | 12.36 | 11.99 | 11.56 | 10.5 | 11.39 | 12.48 | 12.36 | 8.72 | 8.92 | 8.48 | 8.99 | 11.17 | 12.08 |
| 17 | 15.34 | 14.04 | 15.53 | 15.79 | 15.53 | 16.26 | 13.99 | 14.98 | 15.53 | 16.5 | 10.49 | 11.71 | 12.52 | 11.42 | 15.66 | 15.76 |
| 18 | 10.71 | 10.85 | 11.45 | 12.21 | 12.41 | 11.86 | 10.55 | 10.67 | 11.45 | 12.56 | 7.97 | 9.22 | 7.66 | 9.85 | 12.47 | 12.58 |
| 19 | 11.72 | 10.63 | 12.67 | 11.2 | 12.67 | 12.19 | 10.88 | 12.13 | 12.77 | 11.78 | 8.5 | 9.32 | 10.42 | 10.08 | 11.48 | 11.13 |
| 20 | 12.96 | 12.11 | 12.56 | 10.77 | 14.22 | 13.71 | 11.66 | 12.07 | 13.43 | 12.77 | 8.82 | 9.21 | 9.93 | 11.27 | 13.54 | 13.26 |
| 21 | 17.49 | 15.59 | 19.02 | 19.26 | 16.91 | 16.25 | 14.76 | 16.81 | 22.61 | 20.33 | 11.41 | 12.69 | 18.09 | 15.87 | 18.3 | 16.76 |

Abbreviation: SUV_max_: maximum standardized uptake value; AAL: automated anatomical labelling.

Supplementary Table 7 The SUV_meanstd_ of normal 19-44 years old group according to AAL standard.

| Region of Interest | Amygdala | Amygdala | Angular gyrus | Angular gyrus | Anterior cingulate and paracingulate gyri | Anterior cingulate and paracingulate gyri | Basal ganglia | Basal ganglia | Calcarine fissure and surrounding cortex | Calcarine fissure and surrounding cortex | Caudate nucleus | Caudate nucleus | Central region | Central region | Cingulate and paracingulate gyri | Cingulate and paracingulate gyri | Cuneus | Cuneus |
| --- | --- | --- | --- | --- | --- | --- | --- | --- | --- | --- | --- | --- | --- | --- | --- | --- | --- | --- |
| Side | L | R | L | R | L | R | L | R | L | R | L | R | L | R | L | R | L | R |
| Number 1 | -1.9 | -2.5 | 1.2 | 1.6 | -0.2 | -0.4 | 0.4 | 0.1 | 1 | 2.8 | -0.8 | -0.7 | -0.1 | 1.5 | -0.2 | -0.2 | 1.1 | 0 |
| 2 | -1.8 | -1.2 | 3.4 | 1.6 | -1 | -1.3 | 0.2 | 0.3 | 1.6 | 2.5 | -0.4 | -0.1 | 1.1 | 1.5 | -1.5 | -2 | -0.4 | 0.9 |
| 3 | -0.6 | -2.2 | 1.4 | 1.7 | -0.6 | -0.7 | -0.5 | -1.9 | 2.7 | 3.9 | -0.5 | -1 | 1.5 | 0.4 | 0.1 | -0.3 | 0.6 | 1 |
| 4 | -0.9 | -0.5 | 1.4 | 1 | -0.5 | -0.8 | 0.3 | -0.1 | 1 | 2.9 | 0.4 | -0.2 | 1 | 1 | 0.8 | 0.2 | 1.7 | 2.2 |
| 5 | 0.2 | -0.6 | 0.1 | -1.2 | -0.9 | -0.3 | 2.7 | 2 | 1.4 | 1.7 | 2.3 | 1.4 | 0.6 | -0.7 | -1.6 | -0.3 | 0.4 | 3 |
| 6 | -1.3 | -0.7 | -0.9 | -1 | -2 | -2 | 1.3 | 1 | 3.6 | 6 | 0 | 0.5 | -0.5 | 2.4 | -2.5 | -2.3 | 1.3 | 4.3 |
| 7 | -2.3 | -2.3 | -1 | -0.5 | -1.4 | -0.8 | -0.2 | 0 | 1.3 | 3.3 | -0.5 | -0.2 | 0 | 1.8 | -1.5 | -0.2 | 0 | 2 |
| 8 | -2.3 | -2.4 | -0.6 | -0.2 | -1.1 | -1.6 | -0.5 | -0.8 | 0.6 | 2.5 | -1 | -0.5 | -0.4 | 0.5 | -2.6 | -2.1 | -0.7 | 2.4 |
| 9 | -0.7 | -0.3 | -1.7 | 0.3 | -0.6 | 0.9 | -0.1 | -0.1 | 2.6 | 3.9 | -1.2 | -0.9 | 1.3 | 2.2 | -0.3 | 0.7 | 1.1 | 2.3 |
| 10 | -1.9 | -1.6 | 2.1 | -0.3 | -0.9 | -1.2 | -0.8 | -0.9 | 1.9 | 3.3 | -0.4 | 0.1 | 0.4 | 0.5 | -1.2 | -0.9 | 1.9 | 2.6 |
| 11 | -0.6 | -0.8 | 1.5 | 2.8 | 0.3 | 1.4 | -1.1 | -1.3 | -0.8 | -0.2 | -0.5 | -0.5 | -0.9 | -0.3 | 0.6 | 2.1 | -0.2 | 0.5 |
| 12 | 1.2 | -0.1 | 1.4 | -0.4 | 1.6 | 1.8 | 2.8 | 2.4 | 0.3 | 1 | 0.9 | 1.1 | 0.7 | 0.6 | 2.7 | 3.5 | 0.6 | 0 |
| 13 | -2.7 | -1.4 | 0.4 | -0.6 | -2 | -2.3 | 1.5 | 1 | 1.3 | 1.9 | 0.8 | 1 | 0.8 | -0.7 | -0.9 | -0.5 | 2.7 | 3.5 |
| 14 | 0.5 | -0.5 | -2.1 | -0.8 | -1 | -1.1 | 1.1 | 0.8 | 2.5 | 4.6 | 0.2 | 0.2 | 1.4 | -0.2 | -0.8 | -1 | -1.9 | 0.9 |
| 15 | -1.5 | -2.2 | 1.6 | 1.7 | -1.4 | -1.1 | -3.4 | -2.5 | 2.2 | 3.3 | -3.8 | -1.8 | -3.3 | -1.6 | -2.3 | -1.1 | 1.5 | 3.4 |
| 16 | 0.8 | 0.9 | -1.3 | -0.8 | -0.8 | -1.1 | 0 | 0.3 | 0.9 | 1.2 | -0.1 | 0 | 0.3 | -0.8 | -0.4 | -0.5 | 1.2 | 0.4 |
| 17 | -1.2 | -1.2 | 1.1 | -0.1 | 0.3 | 0.5 | -0.3 | -1.3 | 3.3 | 4.1 | -0.5 | -0.7 | 0.9 | 0.4 | -0.2 | -0.2 | 1 | 0.7 |
| 18 | 1.2 | 0.4 | 1.1 | 1.8 | -0.6 | -0.2 | -0.7 | -0.6 | 0.9 | 1.1 | -2.2 | -1.6 | -0.5 | 1.9 | -0.4 | 0.3 | 0.9 | -0.3 |
| 19 | 0.4 | 0.9 | -1 | -0.9 | 1.1 | 0.9 | 0.2 | -0.1 | 1.3 | 0.7 | -0.7 | -0.5 | 1 | 0.5 | 1.5 | 1.4 | 1.2 | 0.9 |
| 20 | -0.1 | 0.6 | -1.5 | -1.7 | -1.3 | -1 | 0.7 | -0.2 | 4 | 4.8 | 0.4 | -0.1 | 0.2 | -0.6 | -1 | -1.2 | 1.7 | 3 |
| 21 | -0.4 | -0.9 | -1.1 | -2.8 | -0.8 | -0.9 | 2.7 | 2.6 | 0.8 | 3.4 | 2.3 | 1.8 | 2 | 1.7 | -0.6 | -0.7 | -0.4 | 1.7 |

| Region of Interest | Frontal lobe | Frontal lobe | Fusiform gyrus | Fusiform gyrus | Gyrus rectus | Gyrus rectus | Heschl gyrus | Heschl gyrus | Hippocampus | Hippocampus | Inferior frontal gyrus, opercular part | Inferior frontal gyrus, opercular part | Inferior frontal gyrus, orbital part | Inferior frontal gyrus, orbital part | Inferior frontal gyrus, triangular part | Inferior frontal gyrus, triangular part | Inferior occipital gyrus | Inferior occipital gyrus |
| --- | --- | --- | --- | --- | --- | --- | --- | --- | --- | --- | --- | --- | --- | --- | --- | --- | --- | --- |
| Side | L | R | L | R | L | R | L | R | L | R | L | R | L | R | L | R | L | R |
| Number 1 | -0.5 | -1.4 | -0.7 | 0.9 | 0.3 | -1 | -0.9 | 1.3 | -1.8 | -1.3 | -0.2 | 0.4 | -2 | -3.7 | -2.1 | -2.7 | 0.7 | 1.2 |
| 2 | 2.8 | 1.2 | -0.9 | -0.8 | 0 | -0.9 | 0.7 | 1.6 | 0.1 | -0.5 | -0.7 | 0 | 3.5 | 1.2 | 4.4 | 2 | 1.4 | 2.5 |
| 3 | 2.2 | -0.7 | -0.3 | 0 | -1.3 | -1.3 | 1.6 | 0.7 | 0.2 | -1.1 | -2.4 | -1.7 | -0.8 | -2.4 | 0.8 | -1.7 | 0.6 | 1.8 |
| 4 | -1.5 | -2 | 0.4 | 1.2 | 0.1 | -0.8 | -1.4 | -1.7 | -0.1 | 0.1 | -0.9 | -1.3 | -1.3 | -0.9 | -2.6 | -2.5 | 0.9 | -0.6 |
| 5 | -3.6 | -2.7 | -1.3 | -0.2 | -2.3 | -2.6 | 0.6 | 0.1 | -0.5 | -0.5 | -2.3 | -0.8 | -1.2 | -3.2 | -1.6 | -3.1 | 2.2 | 1.1 |
| 6 | -1.7 | -0.8 | -0.7 | 0.1 | -3.4 | -1.9 | 2.3 | 1.2 | 0.1 | -0.3 | -0.7 | 0.2 | -1.5 | -0.7 | -1.3 | -1.9 | 1.8 | 2.5 |
| 7 | -3.6 | -1.7 | 0.9 | 1.7 | -3.3 | -3.2 | 1.3 | 1.4 | -1.3 | -2 | -1.7 | -1 | -3.1 | -2.5 | -3.5 | -2.1 | 1.8 | 5.5 |
| 8 | -0.2 | -1.8 | 0.7 | -0.9 | 0.5 | -0.1 | 0.2 | -1.2 | -1.6 | -1.3 | 1.3 | -2.4 | -0.6 | -1 | 0.1 | -1 | 2.8 | 3.6 |
| 9 | -2.8 | -0.3 | 1.1 | 2.2 | -2.4 | -3.2 | -0.2 | 1.4 | 0.3 | 0.2 | -0.8 | 0 | -1.1 | -1.9 | -3.2 | -0.3 | 0.4 | -0.7 |
| 10 | -1 | -1.2 | 2.3 | 2.4 | -0.7 | -0.8 | 0.5 | 1.1 | 0.1 | -1.5 | -0.5 | -0.2 | -0.5 | -2.6 | -0.5 | -1 | 3.4 | 2.5 |
| 11 | 0.2 | 0.3 | -0.6 | -0.7 | -0.7 | -2.7 | -2 | -0.4 | 0.3 | -0.7 | -1.2 | -0.3 | -1.4 | -1.8 | -0.6 | -2.4 | -0.3 | -0.2 |
| 12 | 0 | 0.4 | -2.6 | -1.8 | 0.7 | 0.8 | 1.2 | 1.1 | 1.7 | 1.5 | -0.6 | -1.8 | 0.4 | 0.9 | -0.1 | -0.7 | -2.4 | -3.7 |
| 13 | -0.5 | -0.9 | 0.1 | 0.5 | -0.9 | -1.7 | 1.5 | -1 | -0.6 | -1.2 | -0.7 | -0.4 | 0.6 | 0.9 | -2.9 | -2.1 | 1.2 | 1.8 |
| 14 | -1.9 | -2.8 | 2.1 | 1 | -1.7 | -0.5 | 2.5 | -0.7 | 1.7 | 0.5 | 1.3 | 0.2 | -0.7 | -1 | -2.1 | -1.7 | 3.5 | 4.3 |
| 15 | -0.5 | -1 | -0.5 | 0.5 | -0.1 | -0.4 | -0.6 | 0.6 | -1.2 | -1.4 | -0.8 | 0.5 | -0.7 | -1.6 | 0.6 | -0.7 | 1.9 | 3 |
| 16 | 0.4 | -1.6 | 1.3 | 1.3 | -1.4 | -2 | -1 | 0.5 | 0.9 | 0.5 | -1.7 | -1 | -0.7 | -2.6 | -0.5 | -2.1 | 2.6 | 3.8 |
| 17 | 1.4 | -0.3 | 2.1 | 2.5 | -1.4 | -1.9 | -0.8 | -0.4 | 0.2 | -0.4 | -1.2 | -1.4 | -1 | -1.3 | -1.1 | -0.9 | 3.9 | 4.9 |
| 18 | 1.1 | 1 | 0.9 | 0.8 | -1.2 | -1 | -0.9 | 1.2 | 0.5 | 1.1 | -0.5 | 0.4 | -0.5 | -0.9 | -0.9 | -0.6 | 1.3 | -0.1 |
| 19 | 0.3 | -0.2 | 0.8 | -0.2 | 1.1 | 1 | 2.4 | 1.6 | 1.8 | 1 | -1 | -2.8 | -0.2 | -1.9 | -1.2 | -0.7 | 0.7 | -0.2 |
| 20 | -3.5 | -2.9 | 1.7 | 2.6 | -2.1 | -2.7 | 1.5 | -0.6 | 2.2 | 1.6 | -1 | -1.5 | -1.5 | -1.9 | -3.2 | -2.8 | 2.5 | 0 |
| 21 | -5.6 | -3.9 | -1.3 | -1.5 | -2.5 | -2 | 7.4 | 5.8 | 0 | -0.4 | -3.4 | -1.4 | -0.9 | -0.8 | -3.1 | -3.2 | 0.6 | 0 |

| Region of Interest | Inferior parietal, but supramarginal and angular gyri | Inferior parietal, but supramarginal and angular gyri | Inferior temporal gyrus | Inferior temporal gyrus | Insula | Insula | Lenticular nucleus, pallidum | Lenticular nucleus, pallidum | Lenticular nucleus, putamen | Lenticular nucleus, putamen | Lingual gyrus | Lingual gyrus | Mesial temporal lobe | Mesial temporal lobe | Middle cingulate and paracingulate gyri | Middle cingulate and paracingulate gyri | Middle frontal gyrus | Middle frontal gyrus |
| --- | --- | --- | --- | --- | --- | --- | --- | --- | --- | --- | --- | --- | --- | --- | --- | --- | --- | --- |
| Side | L | R | L | R | L | R | L | R | L | R | L | R | L | R | L | R | L | R |
| Number 1 | -0.7 | 0 | -1.9 | -0.8 | -2.4 | -1.7 | -0.3 | -0.3 | 1.5 | 1.3 | 0.5 | 3.2 | -1.5 | -2.2 | 0.2 | 0.1 | 0.2 | -0.2 |
| 2 | 0.4 | -1.2 | 0.1 | -0.3 | -1.8 | -1.3 | 0 | 1.3 | 0.7 | 0.4 | -0.6 | 0.8 | 0 | -0.8 | -1.4 | -1.7 | 1.7 | 1.3 |
| 3 | 0.1 | -0.4 | -0.2 | -1.9 | 0.2 | -1.8 | 0.6 | 0.9 | -0.7 | -2.4 | 1.3 | 2.2 | 0.1 | -0.9 | 0.8 | 0.3 | 2.2 | 1.7 |
| 4 | -0.8 | -1.1 | -0.1 | 0.3 | -2.2 | -1.6 | 0.2 | 0.6 | 0 | 0 | 1.6 | 1.2 | -0.4 | 0.3 | 2 | 0.9 | 0 | -0.2 |
| 5 | -0.1 | 1.3 | 0.7 | 0.6 | -3.3 | -3.9 | -1 | -1.9 | 3 | 2.5 | 0.9 | 0.7 | -0.1 | -0.6 | -1.2 | 0 | -2 | -0.2 |
| 6 | -2.6 | -1.3 | -1.6 | -1.3 | -3.5 | -2.7 | 0.3 | 0 | 2.1 | 1.3 | 0.9 | -0.1 | -0.5 | -0.9 | -2.8 | -1.9 | 0.1 | 0.3 |
| 7 | 0.6 | -0.3 | -2.2 | -1.9 | -1.9 | 0.7 | 0 | 0.9 | 0.1 | 0.1 | 2.7 | 2.4 | -0.8 | -1.5 | -0.8 | 0.8 | -1.4 | 0.7 |
| 8 | 0.5 | 0.1 | 1 | -1.4 | -1.4 | -1.8 | -1.5 | -2 | 0.6 | -0.2 | 1 | 1.8 | -1.8 | -1.6 | -1.8 | -1.1 | 0.4 | -0.4 |
| 9 | 0.9 | -0.4 | -0.9 | -0.1 | -0.2 | 0 | -0.1 | -0.5 | 0.9 | 1.1 | 0.9 | 1.4 | 0.2 | 0.1 | 0 | 0 | -1.1 | 1 |
| 10 | -0.9 | -1 | 0.1 | -0.2 | -0.6 | -0.4 | -0.8 | -2.5 | -0.6 | -1.1 | 3.2 | 1 | -0.8 | -1.5 | -1.4 | -0.7 | 0 | -0.6 |
| 11 | -0.4 | 0.1 | -0.8 | -0.4 | -2.3 | -2 | -1.7 | -1.8 | -0.7 | -1.2 | -0.3 | -0.1 | -0.4 | -1.6 | 0.8 | 2.3 | -0.4 | 0.3 |
| 12 | 0.1 | 0.3 | -2.9 | -1.6 | 1.8 | 0.2 | 1.2 | 0.3 | 3.6 | 3 | -1.8 | -0.7 | 1.7 | 1.6 | 2.5 | 4.2 | 0.2 | 0.5 |
| 13 | 0.6 | 0 | -1.9 | -3.3 | -2.8 | -2.9 | -0.3 | -2.2 | 1.9 | 1.2 | 0.5 | 0.7 | -0.8 | -1.2 | 0.8 | 1.5 | 0.8 | 1.1 |
| 14 | -0.6 | -2.1 | 0.2 | 0.6 | -0.2 | -1.1 | 0.8 | 0.8 | 1.3 | 1 | 3.3 | 1.8 | 0.6 | -0.3 | 0.1 | -0.2 | 0.3 | 0 |
| 15 | -2.2 | 0.2 | -1 | 0.4 | -2.8 | -2 | 0.2 | -0.7 | -2.5 | -2.1 | 2.1 | 2.7 | -1.4 | -1.4 | -2.2 | -0.4 | 1.6 | 1.7 |
| 16 | 0.7 | -0.1 | -0.1 | 0.1 | 0 | -0.1 | 2 | 1.6 | -0.8 | 0.1 | 1.9 | 0.4 | 1.8 | 0.9 | 1.4 | 0.9 | 0.8 | 0.5 |
| 17 | -2.6 | -1.4 | -0.5 | 0.2 | -0.8 | -2.7 | 0.9 | -1.4 | -0.4 | -1.2 | 2.4 | 2.4 | 0.6 | 0.3 | -0.5 | -0.6 | 2.5 | 1.9 |
| 18 | -2.2 | -0.8 | -0.3 | 1.9 | -0.3 | 0.6 | -0.7 | 0.2 | 1.1 | 0.9 | 0.5 | 1.9 | 0.9 | 1.5 | 0.2 | 1.1 | 0.9 | 1.5 |
| 19 | -0.6 | -1 | -0.3 | -0.6 | 1.2 | 1.5 | 0.2 | 0 | 1 | 0.4 | -0.3 | -0.1 | 1.2 | 0.4 | 1.4 | 1.7 | 0.3 | -0.1 |
| 20 | 0.6 | -1.4 | -1 | -0.8 | -0.4 | -1.7 | 0.2 | 0.2 | 0.7 | -0.4 | 2.9 | 2.3 | 1.7 | 1.2 | -0.1 | -0.6 | -1.5 | -1.8 |
| 21 | -1 | 0.3 | -3 | -3.7 | 0.7 | 0.6 | 1.9 | 2.2 | 1.9 | 1.8 | 1.2 | 2.1 | -0.1 | 0.1 | -0.2 | -0.4 | -2.9 | -2 |

| Region of Interest | Middle frontal gyrus, orbital part | Middle frontal gyrus, orbital part | Middle occipital gyrus | Middle occipital gyrus | Middle temporal gyrus | Middle temporal gyrus | Occipital lobe | Occipital lobe | Olfactory cortex | Olfactory cortex | Paracentral lobule | Paracentral lobule | Parahippocampal gyrus | Parahippocampal gyrus | Parietal lobe | Parietal lobe | Postcentral gyrus | Postcentral gyrus |
| --- | --- | --- | --- | --- | --- | --- | --- | --- | --- | --- | --- | --- | --- | --- | --- | --- | --- | --- |
| Side | L | R | L | R | L | R | L | R | L | R | L | R | L | R | L | R | L | R |
| Number 1 | -0.8 | 0.4 | -0.3 | 0.4 | -0.2 | 0.8 | 0.3 | 2.1 | -1.3 | -1.4 | -1 | -2.4 | -0.8 | -2.3 | -1 | 0.1 | 0 | 2.9 |
| 2 | 1.6 | 3.1 | 1.1 | 0.2 | 1.6 | 2.8 | 0.6 | 1.5 | -1.4 | -2.9 | 0.8 | 1.1 | 0.2 | -0.8 | 0 | -0.4 | 1 | 2 |
| 3 | 0.5 | 2.3 | 0.6 | 2.2 | 1.7 | 1.3 | 1.4 | 2.6 | 0.5 | 0 | 1.3 | -1 | 0.1 | -0.2 | -1.7 | -1.4 | 1.9 | 1.4 |
| 4 | 0.3 | 0.6 | 1.6 | 1.3 | -1.7 | -0.5 | 1.5 | 1.8 | -0.5 | 1.9 | 0.5 | 1.2 | -0.5 | 0.6 | -0.6 | -0.7 | 2.8 | 1.8 |
| 5 | -0.4 | -0.1 | 1.5 | 1.7 | 0 | 1.7 | 1 | 1.4 | -3 | -0.3 | -0.9 | -0.1 | 0.2 | -0.4 | 0 | 0.3 | 1.9 | -0.2 |
| 6 | -1.7 | -0.8 | 3.4 | 4.4 | -0.5 | -1.2 | 2.4 | 3.9 | 0.4 | -1 | 0.3 | 0.2 | -0.7 | -1.2 | -4.3 | -3.3 | -0.5 | 2.7 |
| 7 | -1.7 | -1.6 | 1.7 | 2.1 | -2.6 | -1.2 | 1.9 | 3.7 | -1.8 | 0.5 | 2.1 | 0.4 | 0.1 | -0.3 | -0.5 | -2.2 | 1.3 | 1.3 |
| 8 | -0.3 | -0.7 | 1.3 | 1.8 | -1 | -0.7 | 0.8 | 2.4 | 0.1 | -0.8 | 1.1 | -1.1 | -1.4 | -1.2 | -1.2 | -2.7 | -0.4 | 0.5 |
| 9 | -0.9 | -0.8 | 1.3 | 2.7 | -2 | 0 | 1.8 | 3.1 | -2.5 | -0.8 | 0.7 | -1.1 | 0.2 | 0 | -0.6 | 0.6 | 2 | 2.3 |
| 10 | -1 | 0.5 | 2.5 | 1.8 | 1.2 | 0.7 | 3.1 | 3.2 | 0 | -1 | 0.1 | -0.3 | -1.2 | -1 | -0.5 | -0.7 | 1.8 | 0.6 |
| 11 | 0.2 | -0.1 | -0.9 | 0.1 | -1.3 | -0.9 | -0.8 | -0.2 | 0.4 | 0.4 | 0.8 | 2.4 | -0.9 | -2.1 | 0.4 | 1.8 | -0.8 | 0.7 |
| 12 | 0.7 | 0 | -3.6 | -1.6 | -1.3 | -1.2 | -2.2 | -1.3 | 0.8 | 1.9 | 0.9 | 1.5 | 1.3 | 1.6 | 0.4 | -0.6 | -0.3 | 0.4 |
| 13 | -0.7 | -0.5 | -0.4 | 1.5 | 0 | -0.8 | 0.9 | 2.1 | -1.7 | -2 | 2.4 | 0.6 | -0.4 | -0.8 | 0.8 | -1.2 | 0.8 | -0.9 |
| 14 | 0.8 | 0.3 | 3.9 | 2.2 | 0.5 | -1.9 | 3.3 | 3.5 | -0.7 | -0.4 | 0.2 | -2.8 | -0.5 | -0.9 | -2.1 | -3 | 0.7 | -0.7 |
| 15 | -0.6 | -1.5 | 3.1 | 2.7 | 1.1 | 1.1 | 2.2 | 3.4 | -1.5 | -1.6 | -1.1 | -1.5 | -1.2 | -0.9 | -3 | -1.1 | -2.2 | -0.9 |
| 16 | 0.2 | 0.4 | 2.7 | 4.1 | 0.1 | -0.2 | 2.1 | 2.2 | 0.5 | 0.1 | 1.5 | 1 | 2.4 | 0.9 | 0.5 | -0.3 | 0.7 | -0.2 |
| 17 | -0.2 | 0.5 | 1.4 | 1.9 | -1.5 | -1.4 | 3.1 | 3.8 | 0.3 | 1.2 | 0.5 | -0.4 | 1.2 | 1.1 | -2.4 | -2.2 | 0.6 | 1.3 |
| 18 | 0.8 | 1.7 | -1.2 | 0.2 | -3.1 | 0.6 | 0.4 | 1 | 2.4 | 1.2 | 1.8 | -0.2 | 1 | 1.6 | -2.7 | -1 | -0.6 | 2.3 |
| 19 | 1 | 1.1 | 0.4 | 0.3 | 0.4 | -0.1 | 0.8 | 0.4 | 2.4 | 1.7 | -0.6 | 0.4 | 0.4 | -0.4 | -0.6 | 0.2 | 1.2 | 1.4 |
| 20 | -1.2 | 0 | 1.9 | 2.7 | -0.4 | -1.1 | 3.4 | 4 | -0.6 | -0.7 | -0.3 | 0.7 | 1.1 | 0.7 | -0.4 | -2 | 0.8 | 0.4 |
| 21 | -2.2 | -0.4 | 0.4 | 0.9 | -2.6 | -1.4 | 0.4 | 2 | -0.3 | 0.4 | 0.8 | -1.7 | 0 | 0.7 | -1 | -1.6 | 1.7 | 2 |

| Region of Interest | Posterior cingulate and paracingulate gyri | Posterior cingulate and paracingulate gyri | Precentral gyrus | Precentral gyrus | Precuneus | Precuneus | Rolandic operculum | Rolandic operculum | Superior frontal gyrus, dorsolateral | Superior frontal gyrus, dorsolateral | Superior frontal gyrus, medial | Superior frontal gyrus, medial | Superior frontal gyrus, medial orbital | Superior frontal gyrus, medial orbital | Superior frontal gyrus, orbital part | Superior frontal gyrus, orbital part | Superior occipital gyrus | Superior occipital gyrus |
| --- | --- | --- | --- | --- | --- | --- | --- | --- | --- | --- | --- | --- | --- | --- | --- | --- | --- | --- |
| Side | L | R | L | R | L | R | L | R | L | R | L | R | L | R | L | R | L | R |
| Number 1 | -1 | -0.4 | 0.2 | 0.2 | -1.6 | -1 | -0.8 | -1 | 2.6 | 0.9 | -1.2 | -0.5 | -2.4 | -1.9 | -0.2 | 0 | -0.6 | -1 |
| 2 | -0.8 | -1.4 | 0.3 | 0.9 | -0.4 | 0.5 | 1.3 | -0.6 | 0 | 0.8 | -0.4 | -0.4 | 0.8 | -0.5 | 2.2 | 2 | 0.7 | 1.6 |
| 3 | 0.2 | -0.3 | 1.1 | 0 | -1.4 | -1.5 | -0.9 | -1.9 | 3 | -0.9 | 0.2 | 0.3 | -0.6 | -1.8 | 0.6 | 0.7 | 0.2 | 0.4 |
| 4 | 0.2 | 0.6 | -1.3 | 0 | 1.1 | -0.8 | -0.7 | 0.1 | 0.9 | -1.6 | -2.9 | -2.3 | -2.1 | -4.1 | -0.7 | -0.3 | 0 | -1.5 |
| 5 | -1.7 | -0.8 | -1.3 | -0.2 | 1.3 | 0.8 | 0.1 | -2.4 | -2 | -3.8 | -1.7 | -0.2 | -1 | -2 | -1.4 | -0.4 | -0.3 | -1.4 |
| 6 | 0.6 | -0.9 | 0.1 | 1.9 | -3.8 | -2.5 | -1.2 | -0.8 | -1.1 | -0.9 | -1.4 | -0.4 | -1.1 | -2.1 | -1.9 | 0.4 | 0.8 | 2 |
| 7 | -1 | -1.1 | -1 | 1.9 | -1.1 | -2.6 | -1.8 | 0.2 | -1.5 | -0.9 | -1.4 | -1.3 | -4.7 | -4.4 | -1.6 | 0 | 0.3 | 1.7 |
| 8 | -3.8 | -3 | -0.3 | 0.4 | -3 | -4.5 | 0.2 | -0.2 | 1.1 | -1 | -2.7 | -1.5 | -0.6 | -1.4 | -0.5 | -1.5 | -2.6 | 1.1 |
| 9 | 0.2 | 1.2 | 0.7 | 2.1 | -0.9 | -0.1 | -1.6 | -0.8 | -1.4 | 2.3 | -0.6 | -0.6 | -1 | -0.8 | -2.4 | 0 | 0.9 | 1.1 |
| 10 | 0.7 | 0.5 | -1.5 | 0.7 | -0.1 | -0.4 | -0.2 | -1.1 | -0.2 | 1 | -1.8 | -0.7 | -2.1 | -1 | 0.4 | -0.3 | 0 | 1.7 |
| 11 | 0 | -0.1 | -0.2 | 0 | 0.7 | 1.3 | -1.4 | -3.3 | 1.9 | 2.7 | 0.8 | 0.1 | -0.3 | -2.2 | 0.5 | 0.5 | -0.8 | -0.2 |
| 12 | 1.9 | 0.8 | 1.2 | -0.2 | 1.5 | 0.2 | 1.7 | 2.1 | -1.6 | -0.3 | -1.2 | -0.9 | 2.3 | 1.7 | 0.4 | 1.6 | -1.4 | -1.9 |
| 13 | -0.2 | -0.7 | 0.4 | -0.4 | 1.1 | 0.2 | 0.7 | 0.3 | -0.6 | -1.6 | -0.9 | -1.2 | -1.2 | -2.1 | 0.1 | -0.9 | -0.6 | 0.6 |
| 14 | -1 | -1.3 | 1.4 | 0.3 | -3 | -4.1 | 1.5 | -0.1 | -1.1 | -1.4 | -3.9 | -2.8 | -3.6 | -4.1 | -1.1 | -0.5 | 1.8 | 2.5 |
| 15 | -1 | -1.3 | -3.2 | -1.3 | -2.2 | -2.7 | -1.7 | -1.5 | -0.3 | -1.8 | -1 | -0.6 | -1 | -1.4 | 1.3 | -0.2 | -0.6 | 0.1 |
| 16 | -2.4 | -2.1 | 0.4 | 0.1 | 0.5 | -0.1 | -1.6 | -3.9 | 1.2 | 0.3 | -1.1 | -1.8 | -1 | -4.2 | 0.1 | 1 | -0.6 | 1 |
| 17 | -0.6 | -0.5 | 1.7 | 0.3 | -1.9 | -2.7 | -1.8 | -2.5 | 1.9 | 0.1 | 1 | 0 | 0.3 | -0.8 | 1.3 | -0.1 | 1.1 | 1.8 |
| 18 | -0.4 | -1.4 | -0.2 | 0.8 | -2.1 | -1.9 | 0 | 0.9 | 2 | 0.1 | -0.1 | 0.6 | -0.7 | -0.7 | 2.1 | 0.1 | -1.1 | -0.6 |
| 19 | 0.5 | -0.4 | 0.9 | 0.5 | 0.3 | 1.3 | -1 | -2.7 | 1.4 | 1.1 | -0.4 | 0 | 2.4 | 0.2 | 0.8 | 0.8 | 0.1 | 0.3 |
| 20 | -0.6 | -1.7 | -0.3 | -0.9 | -0.7 | -1.6 | -1.1 | -1.6 | -2.1 | -2.1 | -2.2 | -1.5 | -2.5 | 0.2 | -0.8 | -1.2 | 1.4 | 1.4 |
| 21 | 0.3 | 0 | 0.9 | 0.9 | 0.5 | -1.2 | 2.5 | 0.3 | -2.3 | -3.3 | -5.3 | -2.9 | -0.8 | -2.1 | -2 | -1.2 | -0.2 | 1.7 |

| Region of Interest | Superior parietal gyrus | Superior parietal gyrus | Superior temporal gyrus | Superior temporal gyrus | Supplementary motor area | Supplementary motor area | Supramarginal gyrus | Supramarginal gyrus | Temporal lobe | Temporal lobe | Temporal pole: middle temporal gyrus | Temporal pole: middle temporal gyrus | Temporal pole: superior temporal gyrus | Temporal pole: superior temporal gyrus | Thalamus | Thalamus |
| --- | --- | --- | --- | --- | --- | --- | --- | --- | --- | --- | --- | --- | --- | --- | --- | --- |
| Side | L | R | L | R | L | R | L | R | L | R | L | R | L | R | L | R |
| Number 1 | 0 | 0.7 | 0.5 | 0.7 | 2.4 | 0.4 | -1.1 | -0.6 | -0.9 | 0.4 | -0.4 | -0.1 | -2.4 | 1 | 1 | 0.5 |
| 2 | -1.6 | -1 | 0.9 | 0.1 | -1.4 | -1.7 | -0.2 | -1.3 | 1.3 | 1.5 | 0.3 | 0.1 | -0.1 | -0.1 | 1.9 | 1.9 |
| 3 | -2.8 | -2 | 1 | -0.1 | 2.9 | -0.7 | -1.3 | -1.6 | 1.3 | -0.1 | -0.4 | 0.9 | -0.4 | 0 | 2.8 | 2.3 |
| 4 | -1.8 | -0.7 | 0.2 | 0.4 | 0.2 | -0.8 | -1.6 | -0.5 | -0.9 | -0.1 | 0.6 | 1.6 | -0.6 | 0.3 | 1.9 | 2.1 |
| 5 | -0.5 | 1.3 | 0 | -0.1 | -0.8 | -0.7 | -1.9 | -1.4 | 0.4 | 1.2 | 1 | 0.4 | 0.9 | 0.9 | 1.1 | 0.8 |
| 6 | -1.9 | -2.3 | -0.5 | 0 | 2.1 | 0.5 | -2.9 | -2.4 | -1 | -1.1 | -0.1 | 1.4 | 0 | 0.2 | 1.5 | 1.6 |
| 7 | 0.1 | -1.1 | -1.8 | -3.1 | 1 | 0.2 | -0.4 | -1.5 | -3 | -2.5 | -0.8 | -0.4 | -1 | 0 | 1.6 | 1.1 |
| 8 | 0.2 | -1.9 | -1.4 | -1.2 | -1.3 | 0.5 | -0.3 | -0.8 | -0.6 | -1.5 | -0.2 | -1.3 | 0.3 | -1.3 | 1.2 | 1.2 |
| 9 | -0.1 | 0.7 | -0.6 | -0.5 | -0.6 | -0.6 | -1 | 1.2 | -1.7 | -0.1 | -0.4 | 0.7 | -1.2 | -0.4 | 1.4 | 0.9 |
| 10 | -1.8 | -0.2 | 0.6 | -0.8 | 0.4 | -1 | 1.5 | -0.2 | 0.9 | 0.1 | 0.2 | 0.5 | -0.6 | -1.3 | -0.7 | -0.8 |
| 11 | 0.2 | 1.1 | -1.9 | -2.3 | 0.6 | 2 | -0.7 | -0.2 | -1.9 | -1.6 | -0.5 | -0.2 | -2.1 | -0.3 | 0.7 | 0.6 |
| 12 | -1.3 | -1.9 | -0.8 | -2 | 0.2 | 1.2 | -0.3 | 0 | -2.3 | -2 | -0.1 | 0.2 | 2.3 | 1.4 | 2.1 | 1.5 |
| 13 | -0.6 | -2.7 | -0.6 | -1.3 | 0.1 | 0 | 0.9 | -0.7 | -1 | -2.4 | -0.7 | -0.4 | -1.8 | -1 | 1.8 | 1.9 |
| 14 | 0.1 | -0.1 | -0.9 | -0.6 | 0.1 | -4.5 | -1 | -1.2 | 0.1 | -1 | -0.2 | -0.5 | -0.8 | 0.2 | 2.8 | 2.1 |
| 15 | -2.3 | -1.6 | -2.4 | -0.3 | -2.8 | -1.6 | -2.3 | -0.5 | -1 | 0.7 | -0.1 | 1.2 | -0.5 | -0.3 | -0.3 | 0.7 |
| 16 | 1.7 | 1.5 | -0.1 | -1.6 | 1.6 | -1.3 | -2.3 | -1.3 | -0.1 | -0.6 | 0.4 | 1.1 | -0.3 | 0.1 | 1 | 1.1 |
| 17 | -0.6 | -0.3 | -0.5 | -0.3 | 0.8 | -0.8 | -2.1 | -1.6 | -1.3 | -0.8 | 0.5 | 0.6 | 0.2 | 0.2 | 1.2 | 0.9 |
| 18 | -1.7 | -0.7 | -0.5 | -0.2 | -0.2 | 1.8 | -2 | -1 | -2 | 1.2 | 1.4 | 0.8 | -0.6 | 1.4 | 1.2 | 0.7 |
| 19 | 0 | 0.6 | 0.9 | -0.2 | -0.9 | 0.9 | -1.4 | 0.4 | 0.6 | -0.2 | 0.9 | 2 | 1.3 | 1.6 | 1.2 | 1.1 |
| 20 | 0.7 | 0 | -0.3 | -2.3 | 0.6 | -0.9 | -1.6 | -0.8 | -0.7 | -1.8 | 0.5 | 1.4 | 0.3 | 1.2 | 2.7 | 2 |
| 21 | -1 | -0.9 | 2 | 1.9 | -2.6 | -1.9 | -1 | 0 | -1.4 | -1.1 | -0.2 | 1.2 | 2.9 | 1.2 | 2 | 1.6 |

Abbreviation: SUV_meanstd_: standard deviation of the SUVmean; AAL: automated anatomical labelling.

Supplementary Table 8 The SUV_maxstd_ of normal 19-44 years old group according to AAL standard.

| Region of Interest | Amygdala | Amygdala | Angular gyrus | Angular gyrus | Anterior cingulate and paracingulate gyri | Anterior cingulate and paracingulate gyri | Basal ganglia | Basal ganglia | Calcarine fissure and surrounding cortex | Calcarine fissure and surrounding cortex | Caudate nucleus | Caudate nucleus | Central region | Central region | Cingulate and paracingulate gyri | Cingulate and paracingulate gyri | Cuneus | Cuneus |
| --- | --- | --- | --- | --- | --- | --- | --- | --- | --- | --- | --- | --- | --- | --- | --- | --- | --- | --- |
| Side | L | R | L | R | L | R | L | R | L | R | L | R | L | R | L | R | L | R |
| Number 1 | -0.2 | -1 | 2.3 | 2.3 | 1 | 0.9 | 2.5 | 2.6 | 3.8 | 6 | 1.5 | 1.1 | 3.6 | 4 | 1.2 | 1.6 | 2.1 | 3.8 |
| 2 | 0.4 | -0.1 | 4.5 | 2.7 | 1.9 | 1.1 | 2.4 | 2.6 | 3.6 | 3.6 | 1 | 0.4 | 4 | 4 | 1.9 | 1.7 | 3.7 | 3.6 |
| 3 | 0.4 | -0.7 | 1.3 | 1.9 | 2 | 1.6 | 2.3 | 1.8 | 4.2 | 5.6 | 1.5 | 0.5 | 4.3 | 4.2 | 2.2 | 3 | 3.6 | 5.1 |
| 4 | 1.3 | 1.5 | 3 | 3.8 | 0.7 | 0.1 | 1.7 | 2.6 | 4 | 4.2 | 1.4 | 1.3 | 3.3 | 4.3 | 2.9 | 4.4 | 3.1 | 3.4 |
| 5 | 0.7 | 0.1 | 3 | 3.3 | 1.8 | 1.8 | 2.8 | 3.1 | 4.7 | 5.3 | 2.8 | 2.3 | 3.4 | 3.4 | 3.1 | 2.7 | 2.3 | 5.6 |
| 6 | 1.7 | 1.6 | 1.1 | 2.4 | 0 | 0.4 | 3.5 | 3.7 | 6.6 | 10 | 1.5 | 1.7 | 5.7 | 6.2 | 0.7 | 0.8 | 5.3 | 10.2 |
| 7 | -0.3 | 0 | 2.1 | 3.3 | 0.1 | 0.8 | 2.2 | 3.5 | 6.3 | 7.8 | 1.2 | 1.1 | 4 | 5.2 | 2.9 | 5 | 3.4 | 5.1 |
| 8 | -0.7 | -1.2 | 2.1 | 3 | 0.3 | 0.4 | 1.7 | 1.1 | 4.4 | 6.7 | 0.9 | 0.6 | 4 | 4.2 | 1.2 | 1.6 | 3.1 | 5.4 |
| 9 | 0.7 | 0.7 | 1.8 | 2.2 | 1.7 | 3.3 | 1.3 | 1.7 | 3.8 | 4.7 | 1.1 | 0.7 | 3.8 | 4.3 | 3 | 4.2 | 3.6 | 4.8 |
| 10 | -0.8 | -0.8 | 2.8 | 2.1 | 0.1 | 0.3 | 0.4 | 0.3 | 5.5 | 8.5 | 0.4 | 0.3 | 5.6 | 2.7 | 1.8 | 2.6 | 5.6 | 8.7 |
| 11 | -0.2 | -0.6 | 4.8 | 4 | 2.5 | 2.2 | 1.1 | 1.1 | 1.4 | 2.8 | 1.1 | 1.1 | 3.2 | 3.6 | 3.5 | 5.4 | 2.8 | 3.4 |
| 12 | 1.9 | 1.7 | 2.1 | 1.4 | 3.6 | 3.2 | 4 | 3.9 | 2.7 | 3.1 | 2.4 | 2.5 | 3.6 | 3.4 | 5 | 5.4 | 3.4 | 3.2 |
| 13 | 0.2 | 1 | 2.3 | 2.1 | 1.2 | 2.6 | 2.8 | 3.2 | 2.4 | 4.2 | 2.1 | 1.8 | 3.7 | 3 | 5.1 | 4.6 | 4.3 | 4.5 |
| 14 | 1.7 | 0.4 | 1.3 | 2.2 | 1.3 | 2 | 2.6 | 2.9 | 7.6 | 8.2 | 2.2 | 1.9 | 3.5 | 3.2 | 2.1 | 2.8 | 3.2 | 7.7 |
| 15 | -0.4 | -0.9 | 3.8 | 4.7 | 0.8 | 0.8 | 0 | -0.2 | 5.6 | 6.2 | -0.6 | -0.2 | 2.2 | 2.4 | 1.7 | 1.2 | 3.4 | 5.9 |
| 16 | 3.3 | 1.9 | 1.9 | 2.2 | 1.7 | 0.6 | 3.4 | 3.1 | 3.1 | 6.1 | 1.4 | 1.7 | 2.9 | 2.9 | 4.2 | 4.6 | 2.4 | 4.5 |
| 17 | 0.5 | 0.2 | 1.5 | 1.3 | 1.6 | 2.2 | 1.2 | 0.5 | 5.7 | 6.1 | 0.8 | 0.5 | 4.7 | 5.6 | 1.6 | 2.3 | 4.6 | 5.2 |
| 18 | 2.2 | 1.6 | 2.2 | 3.5 | 2.5 | 1.2 | 1.8 | 2.1 | 2.8 | 2.9 | 1.7 | 1.5 | 3.1 | 4.2 | 2.5 | 2.9 | 2 | 1.1 |
| 19 | 1.5 | 2.5 | 3 | 1 | 1.9 | 2.8 | 2.2 | 3 | 2.2 | 3.1 | 1.4 | 1.2 | 3.8 | 2.4 | 2.9 | 3 | 2.7 | 3.5 |
| 20 | 1.7 | 1.7 | 1.2 | 1.4 | 2.3 | 1.3 | 1.9 | 1.3 | 6.8 | 10.2 | 1.9 | 1.3 | 4.6 | 2.7 | 2.3 | 1.7 | 4.6 | 9 |
| 21 | 2.1 | 1.8 | 0.6 | 0.5 | 0.8 | 0.5 | 4.4 | 4.7 | 4.6 | 5.7 | 1.8 | 1.7 | 7.4 | 5 | 3.2 | 3.2 | 3.1 | 5.9 |

| Region of Interest | Frontal lobe | Frontal lobe | Fusiform gyrus | Fusiform gyrus | Gyrus rectus | Gyrus rectus | Heschl gyrus | Heschl gyrus | Hippocampus | Hippocampus | Inferior frontal gyrus, opercular part | Inferior frontal gyrus, opercular part | Inferior frontal gyrus, orbital part | Inferior frontal gyrus, orbital part | Inferior frontal gyrus, triangular part | Inferior frontal gyrus, triangular part | Inferior occipital gyrus | Inferior occipital gyrus |
| --- | --- | --- | --- | --- | --- | --- | --- | --- | --- | --- | --- | --- | --- | --- | --- | --- | --- | --- |
| Side | L | R | L | R | L | R | L | R | L | R | L | R | L | R | L | R | L | R |
| Number 1 | 6.2 | 4.8 | 2.3 | 5.2 | 1.8 | 2.2 | 1.5 | 2.3 | 2.2 | 1.3 | 1.3 | 1.9 | 3.7 | 2.2 | 1.3 | 2.6 | 4 | 5.9 |
| 2 | 5.1 | 4.7 | 1.3 | 2.3 | 2 | 2.7 | 2.7 | 2.9 | 2.1 | 1.6 | 2.6 | 2.1 | 4.7 | 2.9 | 5.1 | 3.9 | 1.7 | 2.4 |
| 3 | 4.8 | 3.3 | 2.5 | 2.7 | 0.8 | 1.5 | 1.8 | 2.2 | 1.5 | 1.3 | 2.5 | 1.4 | 1.6 | 2.7 | 2.4 | 2.8 | 4.3 | 4.3 |
| 4 | 3.7 | 4.7 | 2.8 | 4.1 | 1.1 | 2.5 | 1.5 | 3.9 | 1.2 | 2.2 | 1.8 | 2.2 | 1.2 | 1.3 | 2.2 | 1.6 | 3.8 | 4 |
| 5 | 3.5 | 2.3 | 2.5 | 3.3 | 1.1 | 0.9 | 2.6 | 2 | 1.8 | 1.9 | 2.5 | 1.9 | 1 | 0.7 | 1.2 | 1.2 | 3.7 | 3.9 |
| 6 | 5 | 4 | 2.4 | 6.6 | 0 | 1.2 | 1.9 | 2.1 | 1.2 | 1.7 | 2.6 | 1.6 | 2.5 | 2.1 | 3 | 2.4 | 5.1 | 8.2 |
| 7 | 3.3 | 4.8 | 4 | 7.4 | 0.1 | 0.1 | 1.4 | 2.1 | 2.3 | 0.6 | 0.6 | 2.9 | -0.1 | 1.4 | 0.8 | 2.2 | 5.3 | 8.1 |
| 8 | 3.3 | 4.4 | 3.9 | 3.9 | 1.7 | 1.3 | 1.4 | 0.9 | 1.2 | 0.1 | 3.1 | 2.8 | 1.6 | 1.5 | 3.1 | 3.9 | 4.7 | 6.4 |
| 9 | 3.3 | 4.8 | 2.5 | 4.1 | 0.5 | 0.9 | 0.6 | 1.4 | 2.3 | 2.7 | 0.6 | 2.4 | 0.8 | 1.2 | 0.7 | 2.8 | 1.7 | 4 |
| 10 | 5.5 | 3.5 | 3.7 | 5.5 | 0.5 | 1.5 | 2 | 2.4 | 1.4 | 0 | 1.8 | 1.3 | 1.4 | 1 | 1.7 | 1.6 | 5.6 | 6.6 |
| 11 | 4.4 | 5.5 | 1.7 | 2.5 | 1.4 | 1.8 | -0.6 | -0.2 | 0.9 | 0.9 | 2.7 | 2.1 | 0.9 | 0.4 | 2.3 | 2.5 | 0.9 | 2.1 |
| 12 | 4.2 | 5.3 | 2.6 | 2.1 | 3.7 | 2.8 | 3.3 | 2.4 | 2.4 | 2.6 | 2 | 1.5 | 2.9 | 3 | 2.3 | 2.1 | 0.5 | -0.1 |
| 13 | 4.1 | 4.9 | 2 | 2.6 | 0.9 | 1.1 | 1.7 | 1.7 | 1.4 | 0.9 | 1.5 | 3.2 | 1.7 | 2.8 | 2.4 | 4.1 | 2.2 | 3.6 |
| 14 | 3.6 | 3.5 | 4.4 | 5.6 | 1.4 | 1.2 | 2.7 | 1.9 | 3.7 | 1.9 | 2.7 | 2.7 | 1.9 | 1.7 | 2.9 | 1.7 | 7.3 | 7.9 |
| 15 | 4 | 3.7 | 4.4 | 6 | 1.1 | 1.5 | 1.3 | 1 | 0.8 | 1.2 | 2.9 | 2.9 | 1.3 | 1.3 | 3.3 | 3.7 | 5.8 | 6.2 |
| 16 | 4.2 | 5.2 | 3.7 | 5.4 | 1.7 | 1.4 | 0.7 | 1.2 | 3.2 | 2.4 | 1.4 | 1.3 | 2.1 | 0.2 | 2.7 | 0.8 | 5.3 | 7 |
| 17 | 5 | 5.1 | 3.7 | 4.6 | 0.7 | 0.8 | 1.2 | 0.4 | 2.4 | 2.5 | 1.1 | 1.7 | 2.8 | 1 | 2.5 | 3.4 | 5.3 | 6 |
| 18 | 4.4 | 4.3 | 3.3 | 2.9 | 1.3 | 1.5 | 0.3 | 1.2 | 2.7 | 2.8 | 2.4 | 1.6 | 3.7 | 3.3 | 3.3 | 1.8 | 2.7 | 2.7 |
| 19 | 4.1 | 4.1 | 2.6 | 2.3 | 1.9 | 1.9 | 2.3 | 2.1 | 2.5 | 2.5 | 2.3 | 1.4 | 2.8 | 1.5 | 2.2 | 1.4 | 1.6 | 2 |
| 20 | 2.9 | 2.5 | 3.2 | 6.4 | 0.8 | 0.6 | 2.7 | 1.6 | 3.6 | 3.6 | 1.1 | 0.8 | 2.8 | 2 | 2.1 | 0.8 | 5 | 9.2 |
| 21 | 3.9 | 3.4 | 2.3 | 2.6 | 1.7 | 1 | 7.6 | 4.9 | 3.5 | 2.8 | 0.7 | 1.1 | 3.9 | 3.1 | 0.9 | 1.6 | 3.6 | 4.8 |

| Region of Interest | Inferior parietal, but supramarginal and angular gyri | Inferior parietal, but supramarginal and angular gyri | Inferior temporal gyrus | Inferior temporal gyrus | Insula | Insula | Lenticular nucleus, pallidum | Lenticular nucleus, pallidum | Lenticular nucleus, putamen | Lenticular nucleus, putamen | Lingual gyrus | Lingual gyrus | Mesial temporal lobe | Mesial temporal lobe | Middle cingulate and paracingulate gyri | Middle cingulate and paracingulate gyri | Middle frontal gyrus | Middle frontal gyrus |
| --- | --- | --- | --- | --- | --- | --- | --- | --- | --- | --- | --- | --- | --- | --- | --- | --- | --- | --- |
| Side | L | R | L | R | L | R | L | R | L | R | L | R | L | R | L | R | L | R |
| Number 1 | 1.8 | 1.8 | 1.6 | 3.2 | 1.1 | 2 | 1.6 | 2 | 2.5 | 2.6 | 3.8 | 6.3 | 2.2 | 1.9 | 1.2 | 1.6 | 3.6 | 2.8 |
| 2 | 3 | 2 | 3 | 2 | 2.5 | 3.1 | 1.6 | 2.1 | 2.4 | 2.6 | 3.1 | 2.7 | 2.1 | 1.6 | 1.5 | 1.7 | 4.3 | 4.7 |
| 3 | 1.7 | 1.4 | 3.4 | 2.9 | 2 | 2.1 | 2.2 | 1.7 | 2.3 | 1.8 | 4.3 | 5.3 | 1.5 | 1.3 | 2.2 | 3 | 4.8 | 2.8 |
| 4 | 2 | 2.6 | 2.7 | 2.8 | 0.7 | 1.3 | 1.6 | 2.5 | 1.7 | 2.6 | 4.1 | 3.9 | 1.3 | 3.1 | 2.9 | 4.4 | 3.7 | 3.5 |
| 5 | 2.9 | 2.7 | 2.5 | 3.2 | 1.3 | 0.7 | 1.4 | 0.9 | 2.4 | 3.1 | 4.4 | 2.4 | 1.8 | 1.9 | 3.1 | 2.7 | 3.5 | 2.2 |
| 6 | 1.8 | 1.4 | 0.6 | 1.6 | 2 | 2.6 | 3 | 3 | 3.5 | 3.7 | 4.7 | 7.7 | 1.7 | 1.7 | 0.4 | 0.8 | 3.8 | 4 |
| 7 | 2.2 | 2.8 | 1.5 | 2.8 | 1.4 | 2.2 | 2.1 | 3.5 | 2.2 | 3.4 | 5.5 | 8.4 | 4 | 3 | 2.9 | 5 | 1.9 | 3.8 |
| 8 | 2.7 | 1.8 | 2 | 2 | 2 | 1.7 | 0.3 | -0.6 | 1.7 | 1.1 | 4.3 | 6.6 | 1.2 | 1 | 1.2 | 1.6 | 2.9 | 4.4 |
| 9 | 2.6 | 1.8 | 2.1 | 3.9 | 1.4 | 2.2 | 1.3 | 1 | 1.3 | 1.7 | 2.7 | 4 | 2.3 | 2.7 | 3 | 4.2 | 2.9 | 4 |
| 10 | 2.3 | 0.7 | 3.2 | 3.1 | 1.8 | 3.1 | 0.1 | -0.9 | 0.2 | -0.6 | 4.9 | 6.9 | 2.9 | 2.7 | 1.8 | 2.6 | 2.9 | 3.1 |
| 11 | 2.4 | 2.9 | 1.4 | 2.5 | 0.4 | 0.1 | -0.5 | -0.5 | 0.2 | -0.1 | 1.7 | 3 | 0.9 | 0.9 | 3.5 | 5.4 | 3.8 | 3.7 |
| 12 | 2.2 | 1.3 | 1.1 | 1.5 | 3.4 | 3.6 | 2.9 | 2.8 | 4 | 3.9 | 2.5 | 3.8 | 2.4 | 2.8 | 5 | 5.4 | 3.5 | 4.2 |
| 13 | 2.9 | 2.2 | 1.2 | 1.2 | 2 | 3 | 1.6 | 1.1 | 2.8 | 3.2 | 2.2 | 4 | 1.4 | 1 | 5.1 | 4.6 | 3.9 | 4.9 |
| 14 | 2.4 | 1.2 | 3.9 | 3.3 | 1.6 | 1.2 | 2.5 | 2.8 | 2.6 | 2.9 | 7.3 | 8.1 | 3.7 | 1.9 | 2.1 | 2.8 | 3 | 3.5 |
| 15 | 2.7 | 2.2 | 1.5 | 2.2 | 0.5 | 0.4 | 0 | -0.6 | -0.1 | -0.4 | 5.3 | 6.1 | 0.8 | 1.4 | 1.7 | 1.2 | 4 | 3.5 |
| 16 | 2.4 | 2.1 | 2.5 | 3.1 | 1.4 | 1.8 | 3.1 | 3.1 | 3.4 | 2.9 | 4.3 | 5.9 | 3.7 | 2.8 | 4.2 | 4.6 | 3.9 | 2.4 |
| 17 | 1.3 | 1.7 | 1.7 | 3.3 | 1.1 | 0.4 | 1.2 | 0 | 1.2 | 0.1 | 4.5 | 5.7 | 3 | 4.1 | 1.5 | 2.3 | 3.7 | 3.8 |
| 18 | 1.7 | 2.1 | 1.7 | 2.9 | 1.8 | 1.4 | 1.8 | 1.6 | 1.8 | 2.1 | 2.9 | 3.5 | 3.2 | 3 | 2 | 2.9 | 3.4 | 3.6 |
| 19 | 1.8 | 1.6 | 2 | 2.1 | 3 | 4.4 | 1.3 | 2.9 | 2.2 | 3 | 2.6 | 2.4 | 2.5 | 2.5 | 2.9 | 3 | 3.7 | 4.1 |
| 20 | 2 | 0.8 | 1.8 | 2.3 | 2.5 | 0.7 | 1.6 | 1.1 | 1.8 | 1.3 | 5.8 | 8.7 | 3.6 | 3.6 | 2.1 | 1.7 | 2.7 | 2.5 |
| 21 | 1.4 | 1.8 | 0.7 | 1 | 6.5 | 4.5 | 3.8 | 4.6 | 4.4 | 4.7 | 3.7 | 5 | 3.5 | 2.8 | 3.2 | 3.2 | 3.4 | 2.9 |

| Region of Interest | Middle frontal gyrus, orbital part | Middle frontal gyrus, orbital part | Middle occipital gyrus | Middle occipital gyrus | Middle temporal gyrus | Middle temporal gyrus | Occipital lobe | Occipital lobe | Olfactory cortex | Olfactory cortex | Paracentral lobule | Paracentral lobule | Parahippocampal gyrus | Parahippocampal gyrus | Parietal lobe | Parietal lobe | Postcentral gyrus | Postcentral gyrus |
| --- | --- | --- | --- | --- | --- | --- | --- | --- | --- | --- | --- | --- | --- | --- | --- | --- | --- | --- |
| Side | L | R | L | R | L | R | L | R | L | R | L | R | L | R | L | R | L | R |
| Number 1 | 3.5 | 4.8 | 5.2 | 4.9 | 2.5 | 2.6 | 5.2 | 6.3 | -0.5 | -1 | 2.3 | 1 | 0.8 | 1.9 | 2.7 | 2.7 | 3.6 | 4 |
| 2 | 4.7 | 3.7 | 3.3 | 3.5 | 4.3 | 3.3 | 3.7 | 3.6 | 1.6 | 0.5 | 3.5 | 2.8 | 1.3 | 0.9 | 4.5 | 3.6 | 4 | 3.1 |
| 3 | 2.3 | 2.9 | 2.6 | 4 | 4.2 | 3 | 4.3 | 5.6 | 1.1 | 1.4 | 3.5 | 1.3 | 1.3 | 1.1 | 3 | 2.1 | 3.9 | 4.2 |
| 4 | 0.9 | 1.6 | 5.7 | 4.3 | 2.8 | 2.3 | 5.7 | 4.3 | 0.7 | 3.2 | 2.7 | 4.7 | 1.3 | 3.1 | 3.5 | 4.9 | 3.3 | 3.6 |
| 5 | 1.4 | 1.5 | 3.8 | 5.7 | 3.1 | 3.6 | 4.7 | 5.7 | 0.7 | 0.6 | 2.1 | 2.3 | 1.5 | 1.2 | 4.5 | 3.6 | 3.4 | 2.7 |
| 6 | 1.9 | 2.9 | 7.3 | 10.4 | 2.3 | 2.8 | 7.3 | 10.6 | 2.3 | 1.9 | 1.7 | 1.9 | 0.9 | 0.6 | 1.8 | 3.1 | 5.3 | 6 |
| 7 | 0.3 | 1 | 6.5 | 6.1 | 1.5 | 2 | 6.5 | 8.4 | -0.7 | 0.3 | 3.2 | 4.8 | 4 | 3 | 3.8 | 3.5 | 4 | 4 |
| 8 | 1.8 | 0.5 | 5.3 | 4.9 | 1.9 | 2.9 | 5.3 | 6.7 | 1 | 0.5 | 1.6 | 0.7 | 1.2 | 1 | 2.8 | 4.3 | 3.9 | 3.9 |
| 9 | 0.6 | 0.8 | 3.4 | 4.5 | 2 | 3.9 | 3.8 | 4.9 | -0.8 | 1 | 2.9 | 2.9 | 1.3 | 2 | 3.7 | 3.9 | 3.8 | 3.8 |
| 10 | 1 | 2.4 | 5.9 | 5.6 | 3.7 | 2.5 | 5.9 | 8.7 | 0.1 | -0.5 | 5.5 | 1.8 | 2.9 | 2.7 | 2.8 | 2.7 | 4.6 | 1.8 |
| 11 | 2.2 | 3.9 | 4.7 | 3.7 | 3 | 2.5 | 4.7 | 3.7 | 1.4 | 2.1 | 2.9 | 5.3 | 0.8 | 0.4 | 4.8 | 4.1 | 2.3 | 3.4 |
| 12 | 1.5 | 1.4 | 2.1 | 2.4 | 2.1 | 1.6 | 3.4 | 3.8 | 4.1 | 3.3 | 3 | 4.1 | 2.3 | 2.8 | 3.8 | 3.9 | 3 | 3.2 |
| 13 | 1.7 | 2.7 | 2.4 | 3.5 | 2.2 | 1.5 | 4.3 | 4.5 | 0.3 | 1.5 | 4.1 | 2.9 | 0.9 | 0.8 | 3.8 | 4 | 3.7 | 3 |
| 14 | 2.1 | 1.8 | 8.5 | 7.7 | 4.2 | 1.5 | 8.5 | 8.2 | -0.2 | 0 | 2.1 | 2.2 | 1.8 | 1.2 | 2.4 | 2.9 | 3.5 | 2.5 |
| 15 | 2.8 | 2.2 | 6.6 | 5.8 | 3.2 | 4.2 | 6.6 | 6.2 | 0.4 | 0.4 | 0.4 | 0.5 | 0.4 | 1.4 | 3.8 | 4.7 | 1.2 | 1.5 |
| 16 | 2.3 | 2 | 5.8 | 6.1 | 3.4 | 3.5 | 5.8 | 7 | 2.5 | 0.7 | 4.2 | 5.2 | 3.7 | 2.8 | 4.3 | 5 | 2.6 | 2.9 |
| 17 | 2.6 | 4.6 | 5.6 | 5.3 | 2 | 1.3 | 5.7 | 6.1 | 1 | 0.8 | 1.6 | 1.1 | 3 | 4.1 | 2.6 | 5.5 | 4.7 | 5.6 |
| 18 | 4.2 | 4.3 | 2 | 2.9 | 1.5 | 3.3 | 3.3 | 3.5 | 1.4 | 1.5 | 2.9 | 2.8 | 3.2 | 3 | 2.3 | 3.5 | 1.9 | 4.2 |
| 19 | 2.9 | 2.3 | 2.7 | 2.9 | 2.6 | 3.1 | 2.7 | 3.5 | 2 | 2 | 2.4 | 1.8 | 2.5 | 1.3 | 3.8 | 2.9 | 3.8 | 2 |
| 20 | 0.8 | 1.2 | 7.1 | 10 | 2.2 | 1.6 | 7.1 | 10.3 | 1.6 | 1.6 | 2.2 | 1.7 | 3 | 3.2 | 2.3 | 2.8 | 4.5 | 2.7 |
| 21 | 1.1 | 0.8 | 4.8 | 4.2 | 2.3 | 2.4 | 4.8 | 5.9 | 2.6 | 1.8 | 3.9 | 3.4 | 2.3 | 1.8 | 4.9 | 3.2 | 6.4 | 4.1 |

| Region of Interest | Posterior cingulate and paracingulate gyri | Posterior cingulate and paracingulate gyri | Precentral gyrus | Precentral gyrus | Precuneus | Precuneus | Rolandic operculum | Rolandic operculum | Superior frontal gyrus, dorsolateral | Superior frontal gyrus, dorsolateral | Superior frontal gyrus, medial | Superior frontal gyrus, medial | Superior frontal gyrus, medial orbital | Superior frontal gyrus, medial orbital | Superior frontal gyrus, orbital part | Superior frontal gyrus, orbital part | Superior occipital gyrus | Superior occipital gyrus |
| --- | --- | --- | --- | --- | --- | --- | --- | --- | --- | --- | --- | --- | --- | --- | --- | --- | --- | --- |
| Side | L | R | L | R | L | R | L | R | L | R | L | R | L | R | L | R | L | R |
| Number 1 | -0.4 | 0.4 | 3.6 | 3.9 | 2.6 | 2.7 | 1.7 | 2.2 | 6.1 | 4 | 5.2 | 2.1 | 1 | 0.7 | 2.8 | 4.7 | 4.1 | 5 |
| 2 | 1.6 | 0.8 | 3.2 | 4 | 3.4 | 3.6 | 3.2 | 3.3 | 2.8 | 3 | 3.1 | 2.8 | 3.3 | 2.9 | 3.9 | 3.4 | 3.4 | 3.3 |
| 3 | 1.1 | 0.6 | 4.3 | 3.4 | 3 | 1.6 | 2.4 | 2.1 | 4.4 | 3.3 | 3.3 | 2.4 | 1.2 | 1.8 | 2.1 | 2.7 | 3 | 5.2 |
| 4 | 1.3 | 1.4 | 2.7 | 3.1 | 3.5 | 4.9 | 1.4 | 4.3 | 3.7 | 2.3 | 1.6 | 0.4 | 0.2 | 0.2 | 1.3 | 2.4 | 4.1 | 3.9 |
| 5 | 1 | 1 | 3.2 | 3.4 | 4.5 | 2.8 | 2.4 | 1.7 | 2.8 | 1.3 | 1.9 | 1.3 | 0.7 | 0.1 | 1.4 | 1.6 | 2.1 | 5.7 |
| 6 | 0.7 | 0.1 | 5.7 | 6.2 | 1.1 | 3.1 | 2 | 2.2 | 2.8 | 1.9 | 3 | 1.2 | 1.9 | 1.1 | 1 | 1.9 | 6.6 | 10.6 |
| 7 | 0.3 | -0.1 | 4 | 5.2 | 2.9 | 2.8 | 1.4 | 2 | 2.9 | 2.9 | 2.7 | 1.8 | 0 | 0.4 | 3.1 | 1.6 | 5.4 | 4.7 |
| 8 | -0.4 | -0.3 | 4 | 4.2 | 1.4 | 2.2 | 2.1 | 1.1 | 3.3 | 3.1 | 2.6 | 1.4 | 1.2 | 0.7 | 2.1 | 1.5 | 4.4 | 5.6 |
| 9 | 1.1 | 1.3 | 3.3 | 4.3 | 3.4 | 3.9 | 1.2 | 2.5 | 3.3 | 4.8 | 1.7 | 2.5 | 1 | 1.4 | -0.4 | 1.4 | 3 | 4.9 |
| 10 | 0.9 | 0.9 | 5.6 | 2.1 | 2.6 | 2.7 | 2 | 2.7 | 2.6 | 3.5 | 0.7 | 0.7 | 0.9 | 1.6 | 1.1 | 2.4 | 4.3 | 8.4 |
| 11 | 2.7 | 1.1 | 3.2 | 3.6 | 2.5 | 4.1 | 0.9 | -0.2 | 4.4 | 5.3 | 3.8 | 3.9 | 3 | 3.7 | 4.2 | 5.5 | 2 | 2.8 |
| 12 | 3.5 | 3.6 | 3.6 | 3.4 | 3.8 | 3.9 | 3 | 2.3 | 3.3 | 2.9 | 3.2 | 2.2 | 3.9 | 2 | 4 | 3.1 | 3.4 | 2.8 |
| 13 | 1.8 | 1.2 | 3.1 | 2.9 | 3.8 | 4 | 2.2 | 2.3 | 3.1 | 2.8 | 2.5 | 1.7 | 1.2 | 1.2 | 1 | 0.9 | 3.9 | 3.6 |
| 14 | 0.1 | 0 | 3.3 | 3.2 | 2.4 | 1.9 | 2.7 | 2.4 | 3.6 | 3.1 | 2.1 | 1.2 | -0.7 | 0.1 | 2.2 | 1.9 | 6.3 | 8 |
| 15 | 0.7 | 0.6 | 2.2 | 2.4 | 3.3 | 2.1 | 1 | 1.6 | 3.4 | 2.9 | 2.4 | 1.4 | 1.8 | 0.9 | 3.3 | 2.2 | 5.2 | 5.8 |
| 16 | 0.9 | 1.6 | 2.9 | 2.3 | 4.3 | 5 | 0.6 | 1 | 3.4 | 2.5 | 2 | 1.8 | 1.3 | 1.2 | 2 | 2.2 | 4.3 | 5.4 |
| 17 | 1 | 0.3 | 4.7 | 5.4 | 2.6 | 5.5 | 1 | 0.8 | 5 | 5.1 | 3.8 | 3.6 | 3.5 | 2 | 3.9 | 3.3 | 4.8 | 5.2 |
| 18 | 0.9 | 0.5 | 3.1 | 3.8 | 1.7 | 1.3 | 1.2 | 1.3 | 3.3 | 3.7 | 2.3 | 3.5 | 2.3 | 1.3 | 4.4 | 2.9 | 1.6 | 2 |
| 19 | 1.3 | 1.3 | 3.6 | 2.4 | 3.8 | 2.9 | 2.5 | 1.6 | 3.8 | 3.4 | 4.1 | 3.4 | 2.3 | 2.5 | 1.9 | 2.7 | 2.5 | 3.2 |
| 20 | 0.9 | 0.8 | 4.6 | 2.4 | 2.3 | 2.8 | 2.9 | 1.3 | 2.4 | 2.2 | 2.6 | 1.9 | -0.2 | 1.5 | 0.8 | 1.6 | 6.1 | 10.3 |
| 21 | 1.7 | 1.1 | 5.6 | 5 | 4.1 | 3.2 | 7.4 | 4.6 | 2.7 | 3.3 | 0.8 | 0.1 | -0.2 | 0.1 | 2.4 | 1.3 | 4.1 | 5.6 |

| Region of Interest | Superior parietal gyrus | Superior parietal gyrus | Superior temporal gyrus | Superior temporal gyrus | Supplementary motor area | Supplementary motor area | Supramarginal gyrus | Supramarginal gyrus | Temporal lobe | Temporal lobe | Temporal pole: middle temporal gyrus | Temporal pole: middle temporal gyrus | Temporal pole: superior temporal gyrus | Temporal pole: superior temporal gyrus | Thalamus | Thalamus |
| --- | --- | --- | --- | --- | --- | --- | --- | --- | --- | --- | --- | --- | --- | --- | --- | --- |
| Side | L | R | L | R | L | R | L | R | L | R | L | R | L | R | L | R |
| Number 1 | 2.7 | 2.6 | 2.5 | 2.5 | 6.2 | 3.9 | 1.4 | 2.1 | 2.5 | 3.2 | 1.4 | 1.7 | 1.9 | 2.4 | 2.6 | 1.5 |
| 2 | 1.9 | 2.7 | 2.9 | 3.2 | 2.2 | 2.2 | 2.4 | 2.8 | 4.3 | 3.3 | 1.8 | 1.9 | 1.8 | 1.9 | 2.6 | 2.3 |
| 3 | 1.8 | 1 | 4 | 2.6 | 2.9 | 2.6 | 0.6 | 2.1 | 4.2 | 3 | 0.8 | 1.8 | 2.1 | 0.9 | 3.1 | 2.8 |
| 4 | 2.4 | 3.2 | 2.1 | 4.1 | 3.3 | 3.4 | 1.2 | 2.9 | 2.8 | 4.1 | 1.3 | 1.4 | 1.5 | 1.7 | 2.2 | 2.5 |
| 5 | 2.8 | 3.6 | 2.8 | 3.2 | 1.9 | 1.2 | 2 | 1.2 | 3.1 | 3.6 | 1.4 | 0.9 | 2.9 | 2.4 | 2.3 | 2 |
| 6 | 1.5 | 2.2 | 2.3 | 2.4 | 5 | 3 | 1 | 2.8 | 2.3 | 2.8 | 1.5 | 2.4 | 2.5 | 2.3 | 2.8 | 2.5 |
| 7 | 3.8 | 3.5 | 0.7 | 1.7 | 3.3 | 4.1 | 2.4 | 2.4 | 1.5 | 2.8 | 1.2 | 2.1 | 1.2 | 2.1 | 3 | 2.2 |
| 8 | 2.8 | 2.4 | 1.1 | 1.3 | 1.5 | 1.4 | 2.3 | 4.3 | 2 | 2.9 | 0.7 | 0.3 | 1.6 | 0.5 | 1.7 | 1.5 |
| 9 | 3.7 | 3 | 1.2 | 1.6 | 2.6 | 2.9 | 1.8 | 3.4 | 2.1 | 3.9 | 1.1 | 2 | 0.9 | 1.4 | 3.5 | 3 |
| 10 | 2.4 | 1.8 | 3 | 2.3 | 2.1 | 1.8 | 2.5 | 1.7 | 3.7 | 3.1 | 2.1 | 1.3 | 1.6 | 0.5 | 0.8 | 0.1 |
| 11 | 2 | 3.7 | 0.8 | 1.4 | 2.6 | 4.8 | 2 | 2.1 | 3 | 2.5 | 0.9 | 0.9 | 1.1 | 0.7 | 1.5 | 1.2 |
| 12 | 2 | 2.3 | 2.5 | 1.5 | 4.2 | 5.3 | 2.4 | 2.7 | 3.3 | 2.4 | 2.3 | 1.6 | 2.7 | 2.7 | 2.7 | 2.5 |
| 13 | 2.9 | 3 | 2.4 | 1.8 | 2.9 | 2.3 | 1.7 | 2.3 | 2.4 | 1.8 | 1.8 | 1.7 | 1.8 | 2.1 | 2 | 2 |
| 14 | 1.8 | 2.9 | 2.7 | 2.8 | 3.2 | 1.9 | 1.7 | 1.2 | 4.2 | 3.3 | 1.2 | 1.1 | 1.1 | 1.9 | 3.9 | 3 |
| 15 | 2.3 | 2 | 2.3 | 3.5 | 1.2 | 1.4 | 1.7 | 2.4 | 3.2 | 4.2 | 1.6 | 2.2 | 2.1 | 1.8 | 1.8 | 2.4 |
| 16 | 2.6 | 2.5 | 1.8 | 2.4 | 3.1 | 3.4 | 0.8 | 1.5 | 3.4 | 3.5 | 3.3 | 2.7 | 3.6 | 2.3 | 2.1 | 1.9 |
| 17 | 2.5 | 1.7 | 2.1 | 1.6 | 2.8 | 2 | 0.7 | 4.3 | 2.1 | 3.3 | 0.7 | 1 | 1.3 | 1 | 2.1 | 1.9 |
| 18 | 2.3 | 1.6 | 1.5 | 1.5 | 2 | 3.8 | 0.6 | 2 | 1.7 | 3.3 | 1.8 | 1.9 | 2.3 | 2 | 3.3 | 2.6 |
| 19 | 3.8 | 2.5 | 3.1 | 3.6 | 2.6 | 2.9 | 2.5 | 1.4 | 3.1 | 3.6 | 2.5 | 3.2 | 3.1 | 4.1 | 2.3 | 1.9 |
| 20 | 2.3 | 1.8 | 2.3 | 2.3 | 2.9 | 1.9 | 1.1 | 1.9 | 2.7 | 2.3 | 2.8 | 2.1 | 3.4 | 2.5 | 3.6 | 3 |
| 21 | 4.9 | 1.7 | 5.9 | 4.1 | 2.4 | 2.1 | 3 | 1.8 | 7.6 | 4.9 | 3.8 | 2.2 | 5.2 | 3.2 | 3.8 | 3 |

Abbreviation: SUV_maxstd_: standard deviation of the SUV_max_; AAL: automated anatomical labelling.

Supplementary Table 9 The SUV_mean_ of normal 45-70 years old group according to AAL standard

| Region of Interest | Amygdala | Amygdala | Angular gyrus | Angular gyrus | Anterior cingulate and paracingulate gyri | Anterior cingulate and paracingulate gyri | Basal ganglia | Basal ganglia | Calcarine fissure and surrounding cortex | Calcarine fissure and surrounding cortex | Caudate nucleus | Caudate nucleus | Central region | Central region | Cingulate and paracingulate gyri | Cingulate and paracingulate gyri | Cuneus | Cuneus |
| --- | --- | --- | --- | --- | --- | --- | --- | --- | --- | --- | --- | --- | --- | --- | --- | --- | --- | --- |
| Side | L | R | L | R | L | R | L | R | L | R | L | R | L | R | L | R | L | R |
| Number 1 | 3.77 | 3.74 | 5.06 | 5.37 | 4.89 | 4.96 | 5.58 | 5.31 | 6.36 | 7.07 | 5.18 | 4.86 | 5.06 | 5.07 | 5.52 | 5.73 | 5.72 | 5.75 |
| 2 | 4.89 | 4.91 | 6.59 | 6.97 | 6.3 | 6.82 | 7.31 | 7.1 | 8.82 | 8.98 | 6.55 | 6.64 | 6.7 | 6.81 | 7.17 | 7.42 | 8.65 | 8.93 |
| 3 | 5.1 | 4.53 | 7.52 | 7.55 | 6.64 | 6.63 | 7.92 | 7.32 | 9.22 | 9.72 | 7.45 | 6.94 | 7.12 | 6.84 | 7.35 | 7.4 | 8.75 | 8.76 |
| 4 | 5.47 | 5.36 | 7.72 | 7.93 | 6.55 | 6.81 | 8.48 | 8.12 | 10.06 | 10.23 | 6.99 | 6.41 | 7.75 | 8.12 | 7.54 | 7.71 | 9.37 | 9.31 |
| 5 | 6.74 | 5.53 | 9.77 | 10.02 | 8.47 | 8.39 | 9.71 | 9.32 | 9.53 | 11.05 | 8.5 | 8.36 | 9.31 | 9.35 | 9.48 | 10 | 10.59 | 9.97 |
| 6 | 5.31 | 5.15 | 6.66 | 7.33 | 6.49 | 7.03 | 7.04 | 6.75 | 7.84 | 7.72 | 6.24 | 5.94 | 6.7 | 6.91 | 7.23 | 7.81 | 7.77 | 7.79 |
| 7 | 6.18 | 5.55 | 9.15 | 9.55 | 8.49 | 8.37 | 9.1 | 8.89 | 10.51 | 9.99 | 8.17 | 8.11 | 8.59 | 8.84 | 8.89 | 9.38 | 9.43 | 9.87 |
| 8 | 4.36 | 3.95 | 6.45 | 6.03 | 5.63 | 5.73 | 6.73 | 6.27 | 7.27 | 7.71 | 5.94 | 5.34 | 5.88 | 5.87 | 6.2 | 6.32 | 6.5 | 6.78 |
| 9 | 5.88 | 5.66 | 8.58 | 7.93 | 7.81 | 8.03 | 9.31 | 9.11 | 10.45 | 10.5 | 8.16 | 8.19 | 8.2 | 8.46 | 8.73 | 8.99 | 8.99 | 9.4 |
| 10 | 5.04 | 4.63 | 6.57 | 6.89 | 5.91 | 6.2 | 6.86 | 6.66 | 8.02 | 8.85 | 6.43 | 6.28 | 6.13 | 6.18 | 6.71 | 6.96 | 7.01 | 7.27 |
| 11 | 7.7 | 7 | 10.61 | 10.94 | 9.73 | 10.15 | 11.55 | 10.51 | 13.01 | 15.12 | 10.17 | 8.69 | 9.33 | 10.33 | 10.69 | 11.6 | 12.61 | 11.92 |
| 12 | 5.48 | 5.41 | 7.86 | 7.8 | 7.29 | 7.9 | 8.98 | 8.48 | 9.08 | 10.21 | 8.13 | 7.88 | 8.22 | 8.43 | 8.47 | 9.03 | 9.32 | 9.93 |
| 13 | 6.32 | 5.38 | 9.03 | 9.59 | 7.79 | 8.18 | 8.79 | 8.46 | 10.45 | 12.32 | 7.81 | 7.62 | 8.76 | 9.09 | 9.42 | 9.66 | 9.7 | 10.18 |
| 14 | 5.76 | 5.48 | 8.55 | 8.32 | 7.18 | 7.36 | 9 | 9.04 | 10.63 | 11.15 | 8.31 | 8.34 | 8.44 | 8.95 | 8.53 | 9.03 | 9.49 | 10.63 |
| 15 | 3.32 | 3.2 | 4.31 | 4.36 | 4.02 | 4.14 | 4.47 | 4.49 | 5.42 | 5.95 | 3.67 | 3.61 | 4.25 | 4.37 | 4.48 | 4.59 | 5.19 | 5.29 |
| 16 | 3.98 | 3.19 | 4.84 | 4.63 | 4.44 | 4.47 | 4.99 | 4.57 | 5.02 | 5.55 | 4.49 | 3.85 | 4.74 | 4.69 | 4.83 | 4.99 | 5.03 | 5.32 |
| 17 | 5.41 | 5.12 | 7.78 | 8.56 | 6.86 | 7.09 | 7.6 | 6.99 | 8.92 | 10.09 | 6.25 | 5.45 | 7.63 | 7.66 | 7.56 | 7.68 | 7.9 | 8.56 |
| 18 | 6.13 | 5.56 | 8.99 | 9.94 | 8.89 | 8.97 | 10.61 | 10.33 | 10.72 | 11.44 | 9.69 | 9.73 | 8.6 | 8.9 | 9.61 | 9.95 | 9.16 | 10.51 |
| 19 | 6.48 | 6.02 | 10.46 | 11.07 | 9.46 | 9.97 | 10.86 | 10.46 | 12.85 | 13.62 | 9.86 | 9.61 | 8.89 | 9.41 | 10.36 | 10.89 | 10.92 | 12.38 |
| 20 | 5.05 | 4.63 | 7.48 | 7.09 | 7.05 | 7.33 | 8.08 | 7.84 | 8.47 | 10.32 | 7.09 | 6.83 | 7.31 | 7.51 | 7.76 | 7.93 | 8.22 | 9.01 |
| 21 | 7.63 | 7.04 | 11.49 | 10.92 | 9.68 | 9.75 | 12.28 | 11.75 | 13.07 | 14.25 | 10.6 | 10.04 | 10.27 | 10.62 | 11.47 | 11.67 | 11.08 | 13.3 |
| 22 | 5.78 | 5.27 | 8.95 | 9.16 | 8.31 | 8.67 | 8.93 | 8.27 | 9.97 | 10.9 | 8.31 | 7.77 | 8.26 | 8.2 | 9.05 | 9.29 | 8.63 | 9.8 |
| 23 | 5.14 | 4.7 | 6.14 | 6.7 | 6.46 | 6.42 | 6.89 | 6.28 | 8.4 | 8.38 | 6.14 | 5.38 | 6.37 | 6.42 | 6.98 | 7.12 | 7.7 | 8 |
| 24 | 5.79 | 4.27 | 7.03 | 7.64 | 7.25 | 7.98 | 7.82 | 7.95 | 8.27 | 9.22 | 7.36 | 6.82 | 7.07 | 7.03 | 7.8 | 8.04 | 8.03 | 8.54 |
| 25 | 4.67 | 4.35 | 6.16 | 6.2 | 6.09 | 6.22 | 7.28 | 6.95 | 5.99 | 6.3 | 6.7 | 6.44 | 6.26 | 6.21 | 6.63 | 6.68 | 6.65 | 6.48 |
| 26 | 5.12 | 4.82 | 7.73 | 7.91 | 6.96 | 7.21 | 7.92 | 7.72 | 8.01 | 9.31 | 7.5 | 6.92 | 7.31 | 7.36 | 7.54 | 7.93 | 7.77 | 8.43 |
| 27 | 4.78 | 4.19 | 6.01 | 5.82 | 5.76 | 5.82 | 6.57 | 6.4 | 7.99 | 8.15 | 5.83 | 5.81 | 6.1 | 5.96 | 6.32 | 6.47 | 6.6 | 7.99 |
| 28 | 4.09 | 3.99 | 5.49 | 5.32 | 4.96 | 5.42 | 6.08 | 5.91 | 6.67 | 7.53 | 5.54 | 5.32 | 5.13 | 5.34 | 5.48 | 5.84 | 6.31 | 6.71 |
| 29 | 5.44 | 4.6 | 7.99 | 7.75 | 6.58 | 6.8 | 8.03 | 7.32 | 9.11 | 11.36 | 6.55 | 5.45 | 7.56 | 8 | 7.22 | 7.48 | 9.27 | 10.09 |
| 30 | 4.88 | 4.32 | 6.5 | 6.36 | 5.93 | 5.98 | 6.8 | 6.88 | 7.26 | 7.93 | 6.32 | 6.69 | 5.88 | 6.07 | 6.39 | 6.67 | 6.89 | 7.75 |
| 31 | 4.63 | 4.4 | 6.71 | 6.53 | 6.14 | 6.17 | 6.66 | 6.39 | 7.71 | 8.09 | 6.22 | 6.15 | 6.04 | 6 | 6.55 | 6.94 | 7.06 | 7.14 |
| 32 | 5.78 | 5.31 | 8.45 | 8.07 | 7.47 | 7.68 | 8.71 | 8.41 | 8.53 | 10.51 | 7.96 | 7.59 | 7.96 | 7.73 | 7.83 | 8.16 | 9.4 | 9.49 |
| 33 | 2.9 | 2.61 | 3.44 | 3.44 | 3.03 | 3.1 | 3.76 | 3.56 | 4.36 | 5.31 | 3.26 | 2.91 | 3.54 | 3.6 | 3.44 | 3.59 | 4.07 | 4.24 |
| 34 | 5.58 | 5.31 | 7.79 | 7.67 | 6.76 | 6.66 | 8.32 | 8.04 | 9.5 | 8.85 | 7.6 | 7.43 | 7.51 | 7.63 | 7.74 | 7.78 | 9.43 | 9.72 |
| 35 | 4.58 | 4.01 | 6.57 | 7.03 | 6.3 | 6.28 | 6.68 | 6.47 | 8.04 | 8.1 | 5.87 | 5.78 | 6.25 | 6 | 6.4 | 6.63 | 6.92 | 7.16 |
| 36 | 4.88 | 4.52 | 7.1 | 6.93 | 6.31 | 6.59 | 7.18 | 6.79 | 7.84 | 7.8 | 6.27 | 6 | 6.36 | 6.47 | 6.97 | 7.35 | 7.03 | 7.85 |
| 37 | 5.03 | 4.8 | 7.88 | 7.75 | 6.23 | 6.32 | 7.69 | 7.35 | 8.71 | 10.27 | 7.04 | 6.64 | 7.05 | 7.15 | 7.02 | 7.16 | 8.56 | 9.66 |
| 38 | 6.89 | 5.96 | 10.67 | 11.76 | 9.06 | 9.33 | 10.95 | 10.62 | 12.88 | 12.87 | 9.65 | 9.45 | 9.7 | 9.84 | 9.98 | 10.73 | 10.67 | 11.68 |
| 39 | 4.88 | 4.68 | 6.41 | 6.59 | 6.43 | 6.46 | 7.15 | 6.95 | 7.54 | 8.36 | 6.28 | 6.43 | 6.71 | 6.7 | 6.73 | 6.99 | 7.53 | 7.97 |
| 40 | 5.97 | 5.5 | 8.93 | 8.5 | 7.96 | 7.92 | 8.69 | 8.38 | 9.28 | 9.52 | 7.72 | 7.47 | 7.72 | 8.01 | 8.69 | 9.13 | 8.4 | 9.44 |
| 41 | 5.85 | 5.02 | 8.17 | 8.57 | 8.1 | 8.29 | 8.29 | 8.05 | 10.12 | 11.01 | 7.45 | 7.33 | 8.48 | 8.39 | 9.03 | 9.32 | 8.83 | 9.32 |
| 42 | 6.94 | 6.47 | 10.15 | 10.25 | 8.97 | 9.29 | 9.57 | 9.12 | 11.23 | 11.63 | 8.82 | 8.36 | 8.93 | 8.69 | 9.98 | 10.2 | 11.01 | 11.77 |
| 43 | 4.63 | 4.51 | 6.04 | 6.13 | 6.13 | 6.33 | 6.44 | 6.14 | 8.08 | 7.84 | 5.93 | 5.61 | 5.78 | 5.75 | 6.53 | 6.78 | 6.9 | 7.37 |
| 44 | 4.01 | 3.74 | 5.49 | 5.49 | 4.69 | 4.72 | 5.64 | 5.24 | 5.84 | 6.49 | 4.81 | 4.28 | 5 | 5.14 | 5.2 | 5.28 | 5.59 | 6.12 |
| 45 | 3.45 | 3.13 | 4.31 | 4.26 | 3.89 | 4.03 | 4.62 | 4.51 | 4.55 | 4.93 | 4.23 | 4.15 | 4.06 | 4.14 | 4.26 | 4.47 | 4.39 | 4.67 |
| 46 | 5.06 | 4.68 | 7.32 | 7.55 | 6.29 | 6.57 | 8 | 7.28 | 8.18 | 8.94 | 6.96 | 6.08 | wang | 7.4 | 7.23 | 7.58 | 8.14 | 8.59 |
| 47 | 5.51 | 5.21 | 8.1 | 8.26 | 7.26 | 7.5 | 8.16 | 7.88 | 9.35 | 10.21 | 7.54 | 7.37 | 7.34 | 7.39 | 7.95 | 8.35 | 7.89 | 8.72 |
| 48 | 7.24 | 6.4 | 9.65 | 10.36 | 8.59 | 9.46 | 10.62 | 9.98 | 11.47 | 13.5 | 9.41 | 8.3 | 9.44 | 9.95 | 10.03 | 10.74 | 10.67 | 11.74 |
| 49 | 3.95 | 3.53 | 4.91 | 4.97 | 4.83 | 5 | 5.44 | 5.11 | 5.18 | 6.23 | 5.15 | 4.73 | 4.88 | 4.71 | 5.22 | 5.5 | 5.07 | 6.12 |
| 50 | 6.85 | 6.01 | 10.91 | 11.21 | 10.52 | 11.02 | 11.64 | 11.25 | 12.74 | 15.45 | 10.98 | 10.25 | 10.07 | 10.2 | 11.04 | 11.57 | 11.48 | 13.83 |
| 51 | 4.4 | 3.67 | 6.18 | 6.29 | 4.69 | 5.05 | 6.69 | 6.39 | 6.34 | 6.92 | 5.16 | 4.91 | 5.79 | 5.78 | 5.5 | 5.76 | 6.24 | 6.17 |
| 52 | 5.28 | 4.91 | 8.16 | 8.43 | 6.79 | 7.18 | 8.31 | 8.23 | 8.82 | 9.84 | 7.58 | 7.44 | 7.51 | 7.37 | 7.85 | 8.37 | 7.44 | 9.53 |
| 53 | 5.4 | 5.25 | 9.55 | 9.95 | 7.73 | 8.22 | 9.12 | 8.62 | 10.97 | 11.82 | 8.52 | 8.65 | 9.11 | 9.99 | 7.85 | 9.45 | 10.56 | 9.53 |
| 54 | 5.38 | 5.16 | 7.38 | 8.15 | 7.26 | 7.41 | 8.72 | 8.3 | 8.91 | 9.03 | 7.91 | 7.87 | 7.5 | 7.58 | 7.93 | 8.16 | 7.71 | 8 |
| 55 | 4.15 | 3.93 | 5.26 | 5.31 | 4.97 | 4.95 | 6.08 | 5.7 | 6.38 | 6.95 | 6.06 | 5.64 | 5.41 | 5.29 | 5.42 | 5.71 | 5.96 | 6.1 |
| 56 | 3.22 | 3.11 | 4.28 | 4.3 | 3.9 | 4.13 | 4.62 | 4.38 | 4.84 | 4.75 | 3.98 | 3.58 | 4.01 | 4.15 | 4.29 | 4.51 | 4.5 | 4.86 |
| 57 | 4.01 | 3.77 | 5.19 | 5.18 | 4.94 | 4.99 | 5.62 | 5.34 | 5.65 | 6.14 | 5.21 | 5.07 | 4.95 | 4.92 | 5.05 | 5.19 | 5.34 | 5.78 |
| 58 | 3.48 | 3.15 | 4.25 | 4.18 | 4.28 | 4.53 | 5 | 4.89 | 5.27 | 5.63 | 4.55 | 4.53 | 4.42 | 4.55 | 4.68 | 5.04 | 4.63 | 4.99 |
| 59 | 5.03 | 4.8 | 7.85 | 7.84 | 6.63 | 6.85 | 7.55 | 7.44 | 8.38 | 9.63 | 6.17 | 6.24 | 7.12 | 6.95 | 7.23 | 7.42 | 7.74 | 8.75 |
| 60 | 4.62 | 4.46 | 6.17 | 6.62 | 5.76 | 6.34 | 6.97 | 6.47 | 7.5 | 8.44 | 6.01 | 5.53 | 6.45 | 6.41 | 6.51 | 7.17 | 6.63 | 8.14 |
| 61 | 3.24 | 3.25 | 4.17 | 4.48 | 4.11 | 4.35 | 4.98 | 4.83 | 4.46 | 4.94 | 4.66 | 4.57 | 4.19 | 4.4 | 4.48 | 4.77 | 4.47 | 4.51 |
| 62 | 5.32 | 4.78 | 6.79 | 6.61 | 5.78 | 5.89 | 7.39 | 6.96 | 8.13 | 8.7 | 6.1 | 5.65 | 6.54 | 6.92 | 6.88 | 7.21 | 7.99 | 8.26 |
| 63 | 7.18 | 6.86 | 10.16 | 11.24 | 9.87 | 10.11 | 10.72 | 10.53 | 12.22 | 13.99 | 10.45 | 10.33 | 9.79 | 9.97 | 10.29 | 11.44 | 10.83 | 11.64 |
| 64 | 3.18 | 2.99 | 4.33 | 4.22 | 3.38 | 3.43 | 4.43 | 4.33 | 4.86 | 5.18 | 3.89 | 3.87 | 3.76 | 3.87 | 3.95 | 4.07 | 4.79 | 4.65 |
| 65 | 7.14 | 6.4 | 9.4 | 9.59 | 9.06 | 9.05 | 10.87 | 10.5 | 10.81 | 13.26 | 9.91 | 9.55 | 9.65 | 9.67 | 9.95 | 9.9 | 10.85 | 12.23 |
| 66 | 6.64 | 6.13 | 10.61 | 10.64 | 9.2 | 9.5 | 10.4 | 10.06 | 11.89 | 13.1 | 9.25 | 9.29 | 9.52 | 9.72 | 10.06 | 10.84 | 10.67 | 12.37 |
| 67 | 6.02 | 5.67 | 8.1 | 8.65 | 8.47 | 8.62 | 8.87 | 8.53 | 10.52 | 11.74 | 8.16 | 8.15 | 8.25 | 8.36 | 9.08 | 9.44 | 9.05 | 9.95 |
| 68 | 5.2 | 4.8 | 6.97 | 7.08 | 6.42 | 6.92 | 7.58 | 7.31 | 7.83 | 8.79 | 6.99 | 6.61 | 6.98 | 7.19 | 7.11 | 7.63 | 7.31 | 7.83 |
| 69 | 4.85 | 4.1 | 6.96 | 6.92 | 6.38 | 6.6 | 7.42 | 7.17 | 6.96 | 8.53 | 6.6 | 6.64 | 6.79 | 7.08 | 6.98 | 7.33 | 7.96 | 8.62 |
| 70 | 4.93 | 4.58 | 6.84 | 6.69 | 6.54 | 6.6 | 7.66 | 7.28 | 7.46 | 8.75 | 7.05 | 6.85 | 6.72 | 6.89 | 6.87 | 7.34 | 7.33 | 8.2 |
| 71 | 5.04 | 4.47 | 8.22 | 7.7 | 6.74 | 6.53 | 7.67 | 7.48 | 7.42 | 8.67 | 7.2 | 7.12 | 6.75 | 6.82 | 7.22 | 7.39 | 8.27 | 8.68 |
| 72 | 6.56 | 5.4 | 8.41 | 8.9 | 7.61 | 8.13 | 8.92 | 8.34 | 11.2 | 12.64 | 7.56 | 6.94 | 8.64 | 8.67 | 8.89 | 9.06 | 9.67 | 9.5 |
| 73 | 4.58 | 4.37 | 7.06 | 7.33 | 6.23 | 6.37 | 7.29 | 7.08 | 7.59 | 8.04 | 6.85 | 6.69 | 6.31 | 6.28 | 6.7 | 6.96 | 6.88 | 7.76 |
| 74 | 4.8 | 4.08 | 7.36 | 7.29 | 6.62 | 7.01 | 6.75 | 6.34 | 8.24 | 8.51 | 6.14 | 6.01 | 6.34 | 6.42 | 6.87 | 7.23 | 7.41 | 7.91 |
| 75 | 3.42 | 3.22 | 4.25 | 4.64 | 4.1 | 4.13 | 4.63 | 4.46 | 5.52 | 5.82 | 4.36 | 4.15 | 4.23 | 4.37 | 4.38 | 4.58 | 4.65 | 5.3 |
| 76 | 4.02 | 3.8 | 5.37 | 5.34 | 5.08 | 5.21 | 5.53 | 5.57 | 5.96 | 6.43 | 5.19 | 5.39 | 4.88 | 5.09 | 5.44 | 5.55 | 5.75 | 6.1 |
| 77 | 6.37 | 5.93 | 10.35 | 10.56 | 9.58 | 10.25 | 9.97 | 9.67 | 11.06 | 12.06 | 8.92 | 9.07 | 8.99 | 9.26 | 10.29 | 11.19 | 11.42 | 12.13 |
| 78 | 4.06 | 4.04 | 6.09 | 6.06 | 5.34 | 5.3 | 6.94 | 6.74 | 6.61 | 7.44 | 6.51 | 6.1 | 5.58 | 5.83 | 6.04 | 6.19 | 6.92 | 7.52 |
| 79 | 5.47 | 4.76 | 8.08 | 7.99 | 7.61 | 8.05 | 8.46 | 8.13 | 8.86 | 10.16 | 7.91 | 7.66 | 7.29 | 7.41 | 8.18 | 8.7 | 8.01 | 7.87 |
| 80 | 5.47 | 5.28 | 7.56 | 7.86 | 7.12 | 7.62 | 8.2 | 8.17 | 8.53 | 9.14 | 7.48 | 7.53 | 7.64 | 7.74 | 8.05 | 8.74 | 8.01 | 8.72 |

| Region of Interest | Frontal lobe | Frontal lobe | Fusiform gyrus | Fusiform gyrus | Gyrus rectus | Gyrus rectus | Heschl gyrus | Heschl gyrus | Hippocampus | Hippocampus | Inferior frontal gyrus, opercular part | Inferior frontal gyrus, opercular part | Inferior frontal gyrus, orbital part | Inferior frontal gyrus, orbital part | Inferior frontal gyrus, triangular part | Inferior frontal gyrus, triangular part | Inferior occipital gyrus | Inferior occipital gyrus |
| --- | --- | --- | --- | --- | --- | --- | --- | --- | --- | --- | --- | --- | --- | --- | --- | --- | --- | --- |
| Side | L | R | L | R | L | R | L | R | L | R | L | R | L | R | L | R | L | R |
| Number 1 | 5.17 | 5.18 | 5.15 | 5.33 | 5.34 | 5.56 | 6.01 | 6.28 | 4.16 | 4.18 | 4.99 | 4.99 | 4.93 | 4.48 | 5.12 | 4.8 | 5.29 | 5.14 |
| 2 | 7.02 | 7.05 | 6.94 | 7.14 | 7.28 | 7.45 | 8.74 | 8.75 | 5.16 | 5.4 | 6.81 | 7.13 | 6.97 | 6.01 | 7.27 | 6.98 | 7.16 | 7.09 |
| 3 | 7.3 | 7.1 | 7.81 | 7.12 | 7.33 | 6.94 | 8.73 | 8.06 | 5.67 | 5.18 | 6.93 | 6.68 | 6.95 | 6.23 | 7.53 | 6.75 | 8.53 | 7.89 |
| 4 | 7.76 | 7.97 | 7.65 | 8.25 | 8.02 | 8.54 | 10.6 | 11.75 | 6.19 | 6.14 | 7.79 | 8.54 | 7.5 | 7.22 | 8.3 | 8.23 | 8.3 | 8.3 |
| 5 | 9.81 | 9.82 | 9.73 | 9.45 | 9.8 | 10.13 | 11.87 | 11.15 | 6.46 | 6.29 | 10.1 | 9.34 | 9.72 | 9.27 | 10.24 | 9.3 | 9.97 | 10.21 |
| 6 | 7.18 | 7.41 | 6.64 | 7.04 | 7.22 | 7.42 | 7.54 | 8.11 | 4.89 | 5.32 | 7.11 | 7.79 | 6.74 | 6.35 | 7.22 | 7.16 | 6.52 | 6.04 |
| 7 | 9.28 | 9.26 | 9.45 | 9.69 | 9.5 | 10.01 | 11.07 | 11.44 | 6.21 | 6.33 | 8.76 | 8.88 | 9.29 | 8.63 | 9.84 | 9.44 | 10.27 | 11.41 |
| 8 | 6.4 | 6.42 | 5.79 | 6.02 | 6.24 | 6.61 | 6.77 | 7.73 | 4.35 | 4.15 | 6 | 6.78 | 6.24 | 6.11 | 6.44 | 6.2 | 5.8 | 5.8 |
| 9 | 8.98 | 8.91 | 8.22 | 7.89 | 9.14 | 9.32 | 12.24 | 12.08 | 6.63 | 6.15 | 8.57 | 9.42 | 9.09 | 8.61 | 9.21 | 8.73 | 8.3 | 7.65 |
| 10 | 6.57 | 6.59 | 6.71 | 6.7 | 6.72 | 7.09 | 9 | 8.93 | 4.86 | 5.09 | 6.54 | 6.75 | 6.65 | 6.05 | 6.73 | 6.56 | 6.74 | 6.26 |
| 11 | 10.58 | 10.88 | 10.55 | 10.97 | 10.5 | 12 | 12.57 | 13.27 | 7.42 | 7.52 | 10.02 | 10.87 | 8.83 | 8.54 | 10.72 | 10.39 | 10.81 | 12.84 |
| 12 | 8.59 | 8.76 | 8.36 | 7.66 | 8.49 | 9.21 | 11.64 | 10.37 | 5.86 | 6.07 | 9.13 | 8.82 | 7.93 | 7.55 | 8.81 | 8.74 | 8.8 | 8.57 |
| 13 | 9.61 | 9.85 | 9.53 | 9.55 | 9.14 | 9.39 | 11.59 | 12.11 | 6.49 | 6.32 | 8.93 | 9.59 | 8.69 | 8.02 | 10.3 | 10.1 | 10.57 | 11.15 |
| 14 | 8.46 | 8.64 | 7.96 | 8.36 | 8.28 | 8.26 | 11.92 | 11.77 | 5.9 | 5.64 | 7.88 | 8.8 | 8.06 | 8.37 | 9.08 | 8.55 | 9.43 | 9.17 |
| 15 | 4.38 | 4.42 | 4.47 | 4.57 | 4.13 | 4.34 | 3.99 | 3.55 | 3.38 | 3.28 | 3.95 | 4.18 | 4.14 | 3.98 | 4.36 | 4.29 | 4.44 | 4.14 |
| 16 | 4.99 | 4.84 | 4.47 | 4.6 | 5.02 | 4.96 | 6.06 | 5.63 | 3.9 | 3.62 | 4.77 | 4.86 | 4.55 | 4.47 | 4.98 | 4.49 | 4.21 | 4.3 |
| 17 | 7.97 | 8.14 | 7.54 | 7.46 | 8.12 | 8.29 | 8.9 | 9.98 | 5.87 | 5.96 | 8.01 | 8.35 | 7.77 | 7.37 | 8.29 | 7.92 | 8.66 | 8.5 |
| 18 | 9.29 | 9.34 | 8.57 | 8.85 | 9.09 | 9.36 | 12.27 | 12.69 | 6.2 | 6.29 | 9.12 | 9.66 | 9.85 | 8.89 | 9.73 | 9.35 | 9.66 | 9.4 |
| 19 | 10.16 | 10.11 | 9.47 | 10.16 | 10.69 | 11.2 | 13.02 | 14.47 | 7.29 | 7.38 | 9.56 | 11.06 | 10.33 | 9.44 | 11 | 10.18 | 11.12 | 10.8 |
| 20 | 7.48 | 7.55 | 7.56 | 7.41 | 7.53 | 8.05 | 9.77 | 10.15 | 5.31 | 5.37 | 7.2 | 7.05 | 7.19 | 6.7 | 7.5 | 7.04 | 7.7 | 7.77 |
| 21 | 11.14 | 11.19 | 10.21 | 10.53 | 9.99 | 10.7 | 13.2 | 12.1 | 7.71 | 7.36 | 10.54 | 11.09 | 10.48 | 9.44 | 11.24 | 11.02 | 11.39 | 11.47 |
| 22 | 8.77 | 8.8 | 8.6 | 8.46 | 9.17 | 9.23 | 10.86 | 11.38 | 6.21 | 6.2 | 8.13 | 8.21 | 8.35 | 7.09 | 8.98 | 8.42 | 9.38 | 9.09 |
| 23 | 6.55 | 6.54 | 6.44 | 6.47 | 6.52 | 6.67 | 7.69 | 7.12 | 5.24 | 5.08 | 6.29 | 6.35 | 6.06 | 5.99 | 6.34 | 6.34 | 6.68 | 6.31 |
| 24 | 7.77 | 7.71 | 7.19 | 7.24 | 8.2 | 7.67 | 9.71 | 8.48 | 6.16 | 5.14 | 7.44 | 8.23 | 6.99 | 6.81 | 8.37 | 7.69 | 7.01 | 6.75 |
| 25 | 6.64 | 6.48 | 5.73 | 5.66 | 6.77 | 6.75 | 8.27 | 7.8 | 4.87 | 4.94 | 6.26 | 6.38 | 6.17 | 5.94 | 6.77 | 6.4 | 5.23 | 5.08 |
| 26 | 7.62 | 7.56 | 7.72 | 7.15 | 7.93 | 7.75 | 10.79 | 9.33 | 5.5 | 5.61 | 7.73 | 7.51 | 6.17 | 6.11 | 7.8 | 7.71 | 8.31 | 7.9 |
| 27 | 6.27 | 6.13 | 6.65 | 6.5 | 6.11 | 6.4 | 7.68 | 7.82 | 5.13 | 4.83 | 6.04 | 5.91 | 6.33 | 5.66 | 6.49 | 5.92 | 6.4 | 6.75 |
| 28 | 5.64 | 5.73 | 5.66 | 5.88 | 5.67 | 5.71 | 5.71 | 7.01 | 4.16 | 4.35 | 5.27 | 5.92 | 5.28 | 5.27 | 5.74 | 5.71 | 5.64 | 5.81 |
| 29 | 8.06 | 8.04 | 7.64 | 7.83 | 7.92 | 7.89 | 8.69 | 9.18 | 4.67 | 4.32 | 8.58 | 8.07 | 7.18 | 6.75 | 8.35 | 8.39 | 9.13 | 9.39 |
| 30 | 6.63 | 6.55 | 5.97 | 5.95 | 6.87 | 6.99 | 8.18 | 7.96 | 4.56 | 4.58 | 6.46 | 6.33 | 6.56 | 6.08 | 6.98 | 6.66 | 5.84 | 5.91 |
| 31 | 6.47 | 6.39 | 6.16 | 6.52 | 6.52 | 6.5 | 7.24 | 7.92 | 4.46 | 4.74 | 6.29 | 6.6 | 6.12 | 5.74 | 6.35 | 6.24 | 6.78 | 6.57 |
| 32 | 8.5 | 8.32 | 8.08 | 7.95 | 8.6 | 8.35 | 10.17 | 10.91 | 5.98 | 6.22 | 8.82 | 8.8 | 8.03 | 7.93 | 8.94 | 8.52 | 9.3 | 8.89 |
| 33 | 3.58 | 3.56 | 3.84 | 3.83 | 3.53 | 3.63 | 4.33 | 4.48 | 2.84 | 2.83 | 3.3 | 3.4 | 3.44 | 3.36 | 3.69 | 3.54 | 3.95 | 4.18 |
| 34 | 7.73 | 7.47 | 8.14 | 8.01 | 7.83 | 7.63 | 10.07 | 10 | 5.86 | 5.43 | 7.4 | 7.75 | 7.42 | 6.57 | 8.33 | 7.73 | 8.44 | 7.61 |
| 35 | 6.7 | 6.44 | 6.52 | 6.37 | 6.56 | 6.14 | 8.57 | 7.26 | 4.9 | 4.37 | 6.57 | 6.3 | 6.29 | 5.73 | 6.76 | 6.12 | 7.1 | 6.88 |
| 36 | 6.88 | 6.97 | 6.76 | 6.55 | 7.15 | 7.17 | 8.18 | 8.69 | 5.19 | 4.82 | 6.74 | 7.08 | 6.66 | 6.41 | 7.16 | 6.71 | 7.05 | 7.3 |
| 37 | 7.23 | 7.21 | 7.44 | 7.18 | 7.42 | 7.59 | 8.55 | 8 | 5.53 | 5.14 | 6.82 | 6.98 | 6.48 | 6.18 | 7.43 | 7.35 | 8.36 | 7.62 |
| 38 | 10.53 | 10.47 | 9.9 | 9.88 | 10.53 | 10.52 | 12.25 | 12.29 | 7.35 | 7.07 | 10.67 | 11.19 | 9.99 | 9.02 | 10.78 | 9.82 | 11.7 | 10.74 |
| 39 | 6.94 | 6.89 | 6.83 | 6.72 | 6.73 | 6.82 | 7.74 | 7.56 | 5.37 | 5.01 | 6.94 | 7.01 | 6.5 | 6.23 | 6.98 | 6.69 | 6.73 | 6.91 |
| 40 | 8.58 | 8.59 | 7.55 | 7.75 | 8.71 | 9.27 | 9.45 | 9.77 | 5.93 | 5.96 | 8.14 | 8.46 | 7.59 | 6.89 | 8.8 | 8.59 | 7.72 | 7.71 |
| 41 | 8.92 | 8.75 | 8 | 8.25 | 8.8 | 8.99 | 11.64 | 12.31 | 6.18 | 5.74 | 8.77 | 9.36 | 8.16 | 7.7 | 9.19 | 8.66 | 8.89 | 8.93 |
| 42 | 10.01 | 9.77 | 9.26 | 9.05 | 9.9 | 10.01 | 11.63 | 9.53 | 7.02 | 6.1 | 9.28 | 9.4 | 10.22 | 9.22 | 10.34 | 9.08 | 10.36 | 8.81 |
| 43 | 6.31 | 6.2 | 6.21 | 6.11 | 6.65 | 6.65 | 7.04 | 7.01 | 4.99 | 4.65 | 5.72 | 6.02 | 6.12 | 5.82 | 6.12 | 5.91 | 6.32 | 5.56 |
| 44 | 5.23 | 5.46 | 5.39 | 5.6 | 5.35 | 5.32 | 6.15 | 6.02 | 4.08 | 4.19 | 4.93 | 5.78 | 5.05 | 4.83 | 5.62 | 5.42 | 5.61 | 6.11 |
| 45 | 4.37 | 4.37 | 4.29 | 4.13 | 4.4 | 4.49 | 4.92 | 5.12 | 3.71 | 3.55 | 4.29 | 4.38 | 4.39 | 4.24 | 4.37 | 4.18 | 4.09 | 4.16 |
| 46 | 7.59 | 7.76 | 7.28 | 7.42 | 7.34 | 7.6 | 7.52 | 7.33 | 5.08 | 5.01 | 6.94 | 7.83 | 7.59 | 7.09 | 7.45 | 7.25 | 8.45 | 7.44 |
| 47 | 8.11 | 7.93 | 7.58 | 7.71 | 8.31 | 8.39 | 8.91 | 8.54 | 5.46 | 5.18 | 7.97 | 8.09 | 7.61 | 7.01 | 8.39 | 7.9 | 8.26 | 7.57 |
| 48 | 10.19 | 10.67 | 9.85 | 9.71 | 10.63 | 10.83 | 12.75 | 14.09 | 6.95 | 6.76 | 9.99 | 11.55 | 9.9 | 9.98 | 10.53 | 10.37 | 10.29 | 11.07 |
| 49 | 5.1 | 5.17 | 5.13 | 5.05 | 5.19 | 5.31 | 6.58 | 5.97 | 4.03 | 3.74 | 5.17 | 5.23 | 4.89 | 4.76 | 4.97 | 4.98 | 5.03 | 5.11 |
| 50 | 11.05 | 10.91 | 10.53 | 10.09 | 11.01 | 11.16 | 12.9 | 13.07 | 7.43 | 6.65 | 11.47 | 11.61 | 10.67 | 10.38 | 10.93 | 10.22 | 10.78 | 10.95 |
| 51 | 5.9 | 6.01 | 5.54 | 5.61 | 5.86 | 6.19 | 7 | 7.17 | 3.99 | 3.98 | 5.51 | 5.52 | 5.59 | 5.59 | 6 | 6.1 | 6 | 5.98 |
| 52 | 7.97 | 7.9 | 7.62 | 7.6 | 8.32 | 8.43 | 9.6 | 8.07 | 5.69 | 5.45 | 7.81 | 8.22 | 7.7 | 7.38 | 8.04 | 7.36 | 8.72 | 8.78 |
| 53 | 9.6 | 8.84 | 8.66 | 9.47 | 9.84 | 11.04 | 10.28 | 6.13 | 6.03 | 9.64 | 10.8 | 8.79 | 8.42 | 9.78 | 9.6 | 10.78 | 9.81 | 8.78 |
| 54 | 8.05 | 7.86 | 7.62 | 7.86 | 8.15 | 8.12 | 10.12 | 9.7 | 5.74 | 5.75 | 7.61 | 7.8 | 7.65 | 7.04 | 8.49 | 7.78 | 7.84 | 8.07 |
| 55 | 5.47 | 5.55 | 5.55 | 5.53 | 5.34 | 5.67 | 5.99 | 6.09 | 4.42 | 4.24 | 5.36 | 5.26 | 5.25 | 4.94 | 5.33 | 5.15 | 5.69 | 5.69 |
| 56 | 4.33 | 4.39 | 4.32 | 4.22 | 4.32 | 4.46 | 5.02 | 4.89 | 3.44 | 3.23 | 4.31 | 4.4 | 3.99 | 3.77 | 4.47 | 4.31 | 4.47 | 4.16 |
| 57 | 5.06 | 4.95 | 4.68 | 4.8 | 5.36 | 5.15 | 6.84 | 7 | 4.15 | 3.85 | 5.26 | 5.13 | 4.98 | 4.6 | 5.23 | 4.92 | 5.16 | 5.28 |
| 58 | 4.56 | 4.57 | 4.5 | 4.42 | 4.69 | 4.64 | 5.54 | 5.8 | 3.63 | 3.73 | 4.72 | 4.48 | 4.46 | 4.38 | 4.56 | 4.38 | 4.54 | 4.62 |
| 59 | 7.21 | 7.23 | 7.18 | 7.23 | 7.29 | 7.28 | 8.71 | 8.79 | 4.83 | 5.07 | 6.81 | 7.27 | 6.94 | 6.74 | 7.03 | 6.92 | 7.93 | 7.62 |
| 60 | 6.61 | 6.71 | 6.04 | 6.47 | 6.88 | 7.13 | 8.76 | 8.52 | 4.85 | 5.06 | 6.54 | 7.06 | 6.46 | 5.85 | 6.52 | 6.39 | 6.82 | 7.06 |
| 61 | 4.5 | 4.47 | 4.21 | 4.12 | 4.71 | 4.69 | 5.13 | 5.77 | 3.59 | 3.39 | 4.22 | 4.63 | 4.36 | 4.19 | 4.57 | 4.3 | 4.05 | 3.85 |
| 62 | 6.6 | 6.87 | 6.52 | 6.55 | 6.6 | 7.08 | 8.22 | 8.92 | 5.47 | 5.49 | 6.24 | 6.8 | 6.51 | 6.23 | 6.71 | 6.93 | 6.56 | 6.51 |
| 63 | 10.74 | 10.78 | 9.85 | 10.31 | 11.09 | 11.06 | 13.11 | 12.63 | 7.01 | 7.32 | 10.18 | 11.19 | 10.1 | 9.34 | 10.8 | 10.13 | 11.74 | 10.26 |
| 64 | 4.08 | 4.07 | 3.79 | 3.95 | 4.29 | 4.26 | 4.41 | 4.69 | 3.16 | 3.19 | 3.73 | 4.1 | 4.12 | 4.07 | 4.1 | 4.12 | 4.1 | 4.14 |
| 65 | 10.05 | 10.04 | 10.11 | 10.12 | 10.41 | 10.69 | 12.28 | 12.4 | 6.92 | 7.23 | 10.15 | 10.66 | 9.48 | 8.87 | 10.44 | 10 | 10.15 | 9.71 |
| 66 | 10.32 | 10.3 | 9.74 | 10.12 | 9.64 | 10.1 | 11.7 | 11.1 | 7.06 | 6.93 | 11.2 | 10.35 | 10.13 | 8.72 | 10.28 | 9.48 | 10.72 | 11.23 |
| 67 | 8.94 | 8.83 | 8.79 | 8.8 | 9.21 | 9.65 | 9.57 | 10.16 | 6.05 | 6.3 | 8.67 | 9.41 | 8.38 | 7.45 | 9.59 | 8.68 | 9.6 | 10.05 |
| 68 | 7.13 | 7.54 | 6.44 | 6.86 | 7.02 | 7.21 | 7.11 | 7.96 | 5.36 | 5.54 | 6.78 | 7.81 | 6.67 | 6.81 | 6.74 | 7.03 | 7.21 | 7.45 |
| 69 | 7.24 | 6.93 | 6.78 | 6.61 | 7.41 | 7.1 | 9.91 | 9.36 | 4.74 | 4.57 | 7.58 | 6.98 | 6.67 | 5.95 | 7.32 | 6.66 | 7.25 | 7.19 |
| 70 | 6.79 | 6.92 | 6.3 | 6.51 | 7.21 | 7.06 | 8.22 | 8.37 | 5.28 | 5.11 | 6.77 | 6.93 | 6.95 | 6.54 | 6.94 | 6.71 | 6.25 | 7.21 |
| 71 | 7.18 | 7.09 | 7.16 | 7.07 | 7.43 | 7.68 | 7.7 | 9.13 | 5.33 | 5.11 | 6.8 | 7.37 | 7.05 | 6.1 | 7.7 | 7.08 | 7.48 | 7.54 |
| 72 | 9.23 | 9.09 | 9.7 | 9.15 | 9.84 | 10.07 | 10.7 | 9.87 | 5.88 | 6.15 | 9.33 | 9.1 | 8.72 | 8.46 | 9.45 | 8.6 | 10.99 | 10.98 |
| 73 | 6.61 | 6.53 | 6.65 | 6.46 | 6.74 | 7.05 | 7.67 | 8.32 | 4.74 | 4.65 | 6.6 | 6.38 | 6.26 | 5.85 | 6.95 | 6.57 | 6.76 | 7.02 |
| 74 | 6.75 | 6.7 | 6.29 | 6.3 | 6.87 | 6.77 | 8.76 | 7.55 | 5.1 | 4.8 | 6.73 | 6.75 | 6.39 | 5.74 | 6.98 | 6.68 | 6.28 | 6.29 |
| 75 | 4.56 | 4.67 | 4.27 | 4.34 | 4.48 | 4.63 | 5.07 | 5.12 | 3.54 | 3.49 | 4.61 | 4.75 | 4.5 | 4.34 | 4.64 | 4.52 | 4.62 | 4.53 |
| 76 | 5.28 | 5.34 | 5.29 | 5.08 | 5.23 | 5.34 | 5.78 | 6.84 | 3.99 | 3.71 | 4.99 | 5.1 | 5.29 | 5.17 | 5.38 | 5.13 | 4.49 | 4.84 |
| 77 | 9.71 | 10.27 | 8.61 | 9.15 | 9.31 | 9.77 | 12.08 | 11.61 | 7.02 | 7.12 | 9.23 | 9.92 | 8.71 | 9.16 | 10.3 | 10.37 | 9.24 | 10.62 |
| 78 | 5.82 | 6.05 | 5.55 | 5.59 | 5.88 | 5.94 | 7.33 | 7.73 | 4.43 | 4.35 | 5.54 | 6.39 | 5.44 | 5.69 | 5.69 | 6.12 | 5.79 | 5.77 |
| 79 | 7.8 | 7.94 | 7.26 | 7.44 | 8.09 | 8.48 | 9.62 | 9.9 | 5.38 | 5.61 | 7.49 | 7.65 | 7.84 | 7.43 | 8.22 | 7.7 | 7.68 | 7.98 |
| 80 | 7.94 | 8 | 7.98 | 7.62 | 7.68 | 7.41 | 9.82 | 9.87 | 5.59 | 5.58 | 7.62 | 8.75 | 7.22 | 7.01 | 8.33 | 7.65 | 8.64 | 8.71 |

| Region of Interest | Inferior parietal, but supramarginal and angular gyri | Inferior parietal, but supramarginal and angular gyri | Inferior temporal gyrus | Inferior temporal gyrus | Insula | Insula | Lenticular nucleus, pallidum | Lenticular nucleus, pallidum | Lenticular nucleus, putamen | Lenticular nucleus, putamen | Lingual gyrus | Lingual gyrus | Mesial temporal lobe | Mesial temporal lobe | Middle cingulate and paracingulate gyri | Middle cingulate and paracingulate gyri | Middle frontal gyrus | Middle frontal gyrus |
| --- | --- | --- | --- | --- | --- | --- | --- | --- | --- | --- | --- | --- | --- | --- | --- | --- | --- | --- |
| Side | L | R | L | R | L | R | L | R | L | R | L | R | L | R | L | R | L | R |
| Number 1 | 5.15 | 5.61 | 4.98 | 5.06 | 5.15 | 4.91 | 4.08 | 3.57 | 6.4 | 6.2 | 5.72 | 5.73 | 4.02 | 4.02 | 5.9 | 6.28 | 5.51 | 5.51 |
| 2 | 6.88 | 6.42 | 6.69 | 6.88 | 7.08 | 6.74 | 5.86 | 5.49 | 8.47 | 7.97 | 7.85 | 7.58 | 5.17 | 5.17 | 7.71 | 7.96 | 7.39 | 7.7 |
| 3 | 7.48 | 7.77 | 6.8 | 6.74 | 6.87 | 6.24 | 6.41 | 5.25 | 8.82 | 8.23 | 8.6 | 7.86 | 5.48 | 5.28 | 7.86 | 8 | 7.77 | 7.77 |
| 4 | 8.13 | 8.42 | 6.98 | 7.34 | 8.11 | 8.3 | 6.53 | 5.89 | 10.46 | 10.3 | 8.96 | 8.75 | 6.08 | 6.07 | 7.85 | 8.25 | 8.05 | 8.57 |
| 5 | 10.17 | 9.5 | 9.15 | 8.88 | 9.29 | 8.98 | 8 | 6.61 | 11.36 | 10.94 | 10.82 | 9.93 | 6.72 | 6.49 | 10.29 | 11.17 | 10.53 | 10.73 |
| 6 | 7.32 | 7.94 | 6.38 | 6.54 | 6.96 | 6.88 | 5.9 | 5.47 | 8.13 | 7.84 | 6.84 | 6.77 | 5.06 | 5.23 | 7.69 | 8.38 | 7.57 | 7.97 |
| 7 | 9.35 | 9.44 | 8.54 | 9.01 | 8.44 | 8.47 | 6.73 | 6.39 | 10.68 | 10.28 | 10.24 | 9.38 | 6.38 | 6.27 | 9.17 | 10.18 | 9.85 | 10.03 |
| 8 | 5.72 | 5.95 | 5.69 | 5.69 | 6.23 | 6.05 | 4.97 | 4.23 | 8.01 | 7.66 | 6.24 | 6.23 | 4.4 | 4.34 | 6.48 | 6.76 | 6.74 | 6.93 |
| 9 | 7.94 | 9.51 | 8.05 | 8.2 | 8.83 | 8.48 | 7.32 | 6.25 | 10.98 | 10.73 | 9.55 | 8.9 | 6.24 | 6.22 | 9.22 | 9.7 | 9.49 | 9.64 |
| 10 | 6.71 | 6.87 | 6.36 | 6.27 | 6.72 | 6.63 | 5.64 | 4.84 | 7.62 | 7.49 | 7.35 | 6.86 | 4.92 | 4.97 | 7.16 | 7.45 | 7.21 | 7.18 |
| 11 | 10.91 | 10.88 | 9.77 | 10.05 | 9.15 | 9.34 | 9.33 | 8.34 | 13.51 | 12.78 | 12.04 | 10.89 | 7.82 | 7.45 | 11.37 | 12.64 | 11.2 | 12.04 |
| 12 | 8.91 | 8.88 | 7.77 | 7.77 | 8.57 | 8.53 | 6.47 | 5.72 | 10.51 | 9.77 | 8.46 | 7.88 | 5.96 | 6.01 | 9.34 | 9.93 | 9.22 | 9.68 |
| 13 | 9.4 | 10.07 | 8.2 | 8.84 | 8.36 | 8.6 | 6.57 | 6.1 | 10.36 | 9.87 | 10.78 | 9.88 | 6.47 | 6.58 | 10.71 | 10.91 | 10.41 | 11.01 |
| 14 | 8.81 | 9.14 | 7.61 | 7.95 | 7.88 | 8.4 | 7.35 | 6.54 | 10.13 | 10.34 | 9.55 | 9.55 | 6.11 | 6.08 | 9.39 | 10.11 | 9.17 | 9.38 |
| 15 | 4.29 | 4.55 | 3.97 | 4.08 | 3.88 | 3.75 | 3.62 | 3.66 | 5.48 | 5.52 | 5.04 | 4.81 | 3.29 | 3.26 | 4.79 | 4.92 | 4.57 | 4.74 |
| 16 | 4.84 | 4.93 | 4.48 | 4.53 | 4.8 | 4.71 | 3.92 | 3.91 | 5.79 | 5.43 | 4.72 | 4.44 | 3.85 | 3.59 | 5.03 | 5.4 | 5.22 | 5.27 |
| 17 | 8.6 | 9.2 | 7.42 | 7.75 | 7.37 | 7.21 | 6.18 | 5.63 | 9.29 | 8.79 | 8.57 | 8.46 | 5.71 | 5.87 | 8.25 | 8.21 | 8.61 | 8.87 |
| 18 | 10.02 | 10.42 | 8.2 | 8.53 | 9.07 | 8.71 | 7.6 | 6.73 | 12.36 | 11.83 | 9.74 | 9.26 | 6.46 | 6.47 | 10.13 | 10.84 | 9.86 | 10.19 |
| 19 | 10.21 | 10.71 | 9.53 | 10.18 | 10.59 | 10.43 | 10.18 | 7.97 | 12.02 | 11.91 | 11.66 | 11.2 | 7.22 | 7.47 | 10.56 | 11.48 | 10.77 | 11.05 |
| 20 | 7.05 | 6.96 | 6.93 | 7 | 7.65 | 7.73 | 6.8 | 5.72 | 9.39 | 9.35 | 8.23 | 7.66 | 5.41 | 5.5 | 8.09 | 8.38 | 7.92 | 8.21 |
| 21 | 10.66 | 11.21 | 10.14 | 10.28 | 10.54 | 10.24 | 9.07 | 7.89 | 14.83 | 14.36 | 11.92 | 11.06 | 8.11 | 7.93 | 12.4 | 12.86 | 11.97 | 12.12 |
| 22 | 8.63 | 9.25 | 7.73 | 7.92 | 8.45 | 8.25 | 7.39 | 7.24 | 9.97 | 9.01 | 9.18 | 8.58 | 6.34 | 6.31 | 9.27 | 9.75 | 9.62 | 9.78 |
| 23 | 6.36 | 6.71 | 5.99 | 6.27 | 5.9 | 6.03 | 4.89 | 4.55 | 8.2 | 7.57 | 7.39 | 6.64 | 5.09 | 4.92 | 7.14 | 7.55 | 6.88 | 7.21 |
| 24 | 7.35 | 7.42 | 7.33 | 7 | 7.51 | 7.51 | 5.86 | 6.57 | 8.82 | 9.37 | 7.71 | 8.06 | 5.77 | 5.68 | 8.3 | 8.14 | 8.2 | 8.21 |
| 25 | 6.07 | 6.34 | 6.01 | 5.84 | 6.55 | 6.23 | 5.41 | 4.88 | 8.37 | 7.98 | 5.54 | 5.43 | 4.9 | 4.91 | 7.04 | 7.04 | 7.05 | 6.83 |
| 26 | 7.3 | 7.85 | 6.74 | 6.8 | 6.92 | 6.89 | 6.01 | 6.21 | 8.87 | 8.87 | 7.81 | 8.13 | 5.4 | 5.73 | 7.98 | 8.55 | 8.27 | 8.49 |
| 27 | 5.88 | 5.73 | 5.88 | 5.67 | 6.07 | 5.61 | 5.53 | 5.04 | 7.58 | 7.32 | 7.64 | 6.75 | 4.9 | 4.76 | 6.58 | 6.88 | 6.56 | 6.58 |
| 28 | 5.28 | 5.47 | 5.14 | 5.61 | 4.97 | 5.27 | 4.95 | 4.27 | 6.93 | 6.89 | 6.15 | 5.66 | 4.26 | 4.35 | 5.94 | 6.28 | 5.93 | 6.21 |
| 29 | 7.77 | 7.9 | 7.24 | 7.59 | 7.41 | 7.43 | 6.45 | 5.66 | 9.89 | 9.51 | 8.74 | 8.64 | 5.29 | 5.16 | 7.73 | 8.09 | 8.81 | 8.93 |
| 30 | 6.65 | 6.77 | 6.22 | 6.23 | 6.37 | 5.99 | 5.26 | 4.74 | 7.71 | 7.62 | 6.49 | 6.32 | 4.68 | 4.59 | 6.73 | 7.16 | 7.03 | 7.1 |
| 31 | 6.44 | 6.92 | 5.98 | 5.99 | 6.05 | 5.83 | 5.04 | 4.42 | 7.54 | 7.12 | 7.15 | 6.33 | 4.66 | 4.73 | 6.76 | 7.58 | 6.76 | 6.81 |
| 32 | 7.48 | 8.2 | 8.31 | 7.97 | 8.23 | 8.09 | 6.66 | 6.05 | 10.03 | 9.8 | 8.55 | 7.45 | 6.24 | 6.22 | 8.37 | 8.84 | 9.42 | 9.16 |
| 33 | 3.39 | 3.41 | 3.33 | 3.66 | 3.39 | 3.32 | 3.44 | 3 | 4.33 | 4.33 | 4.34 | 3.93 | 2.83 | 2.8 | 3.69 | 3.93 | 3.75 | 3.89 |
| 34 | 8.15 | 8.23 | 7.68 | 7.61 | 7.91 | 7.75 | 6.69 | 5.94 | 9.49 | 9.17 | 8.67 | 7.66 | 5.68 | 5.49 | 8.41 | 8.59 | 8.29 | 8.23 |
| 35 | 6.84 | 7.19 | 6.27 | 6.23 | 6.33 | 5.89 | 5.6 | 4.96 | 7.77 | 7.51 | 7.38 | 7.2 | 4.79 | 4.47 | 6.49 | 6.94 | 7.3 | 7.14 |
| 36 | 6.43 | 6.87 | 6.12 | 6.33 | 6.59 | 6.55 | 5.8 | 5.09 | 8.45 | 7.98 | 7.18 | 6.75 | 5.01 | 5.06 | 7.34 | 7.87 | 7.37 | 7.73 |
| 37 | 7.58 | 7.76 | 6.82 | 6.97 | 6.75 | 6.43 | 5.92 | 4.95 | 8.83 | 8.65 | 8.28 | 7.81 | 5.13 | 5.12 | 7.51 | 7.75 | 7.71 | 8.04 |
| 38 | 10.64 | 10.29 | 9.54 | 9.8 | 10.19 | 9.61 | 10.75 | 9.02 | 12.24 | 12.13 | 10.41 | 11.16 | 7.73 | 7.65 | 10.35 | 11.65 | 11.35 | 11.94 |
| 39 | 6.72 | 6.5 | 6.25 | 6.39 | 6.35 | 6.03 | 5.68 | 5.08 | 8.4 | 7.92 | 7.17 | 6.54 | 5.08 | 4.88 | 7.19 | 7.55 | 7.38 | 7.43 |
| 40 | 8.56 | 8.8 | 7.17 | 7.81 | 7.44 | 7.5 | 6.85 | 6.42 | 10.14 | 9.75 | 8.37 | 8.11 | 5.73 | 5.91 | 9.31 | 10.09 | 9.1 | 9.55 |
| 41 | 8.79 | 8.69 | 7.68 | 7.93 | 8.44 | 8.33 | 6.82 | 6.31 | 9.52 | 9.19 | 9.4 | 9 | 6.06 | 5.74 | 9.54 | 10.04 | 9.49 | 9.5 |
| 42 | 10.33 | 11.45 | 9.53 | 9.23 | 9.5 | 8.68 | 7.49 | 6.64 | 10.89 | 10.48 | 10.43 | 9.44 | 7.06 | 6.84 | 10.5 | 10.93 | 10.71 | 10.67 |
| 43 | 6.17 | 6.46 | 5.89 | 5.89 | 5.92 | 5.76 | 5.13 | 4.7 | 7.31 | 7.01 | 6.58 | 6.56 | 4.98 | 4.81 | 6.58 | 6.99 | 6.51 | 6.64 |
| 44 | 5.03 | 5.37 | 5.01 | 5.34 | 5.12 | 4.98 | 4.55 | 4.42 | 6.74 | 6.35 | 5.74 | 5.12 | 4.02 | 3.93 | 5.63 | 5.77 | 5.33 | 6.04 |
| 45 | 4.51 | 4.53 | 3.96 | 4 | 4.3 | 4.22 | 3.71 | 3.57 | 5.26 | 5.09 | 4.37 | 4.16 | 3.61 | 3.52 | 4.52 | 4.76 | 4.53 | 4.65 |
| 46 | 7.13 | 7.67 | 6.8 | 7.3 | 6.91 | 6.63 | 6.72 | 5.98 | 9.37 | 8.75 | 8.49 | 7.21 | 5.01 | 5.31 | 7.79 | 8.32 | 8.18 | 8.42 |
| 47 | 7.56 | 8.12 | 7.5 | 7.78 | 7.23 | 6.91 | 5.82 | 4.9 | 9.43 | 9.15 | 8.75 | 7.99 | 5.8 | 5.76 | 8.43 | 8.97 | 8.63 | 9.08 |
| 48 | 10.01 | 10.46 | 9.14 | 9.88 | 9.77 | 9.95 | 7.29 | 7.35 | 12.73 | 12.24 | 11.16 | 11.04 | 7.22 | 7.18 | 11.11 | 11.72 | 10.46 | 11.79 |
| 49 | 5.29 | 5.28 | 4.59 | 4.71 | 4.86 | 4.78 | 4.62 | 3.8 | 5.95 | 5.8 | 5.76 | 5.09 | 3.97 | 3.86 | 5.5 | 5.91 | 5.33 | 5.57 |
| 50 | 10.89 | 11.12 | 9.93 | 9.88 | 10.13 | 9.78 | 8.94 | 7.9 | 13.04 | 13.06 | 12.26 | 12.04 | 7.65 | 7.28 | 11.41 | 12.09 | 11.7 | 12.02 |
| 51 | 5.5 | 6.17 | 5.36 | 5.44 | 5.41 | 5.65 | 5.19 | 4.84 | 8.58 | 8.18 | 6.44 | 6.27 | 4.25 | 4.22 | 6.06 | 6.22 | 6.03 | 6.34 |
| 52 | 7.97 | 8.22 | 7.4 | 7.43 | 7.24 | 6.94 | 7.38 | 6.39 | 9.28 | 9.45 | 8.22 | 7.93 | 5.75 | 5.61 | 8.59 | 9.28 | 8.78 | 8.66 |
| 53 | 9.42 | 9.77 | 8.68 | 8.84 | 8.86 | 8.35 | 7.16 | 6.71 | 10.87 | 10.21 | 9.81 | 9.64 | 6.23 | 6.18 | 9.77 | 11.04 | 10.35 | 10.61 |
| 54 | 8.27 | 8.69 | 7.54 | 7.54 | 8.2 | 7.79 | 7.13 | 6.39 | 9.95 | 9.19 | 8.05 | 7.92 | 5.63 | 5.59 | 8.69 | 8.96 | 8.65 | 8.63 |
| 55 | 5.33 | 5.67 | 5.1 | 5.47 | 5.07 | 4.82 | 4.58 | 4.05 | 6.54 | 6.18 | 6 | 5.9 | 4.23 | 4.19 | 5.71 | 6.26 | 5.74 | 5.99 |
| 56 | 4.25 | 4.35 | 4.18 | 4.31 | 4.32 | 4.38 | 3.5 | 3.14 | 5.54 | 5.46 | 4.51 | 4.3 | 3.36 | 3.23 | 4.43 | 4.82 | 4.54 | 4.83 |
| 57 | 5.3 | 5.42 | 4.74 | 4.73 | 5.4 | 5.25 | 4.6 | 4.19 | 6.31 | 5.9 | 5.37 | 4.84 | 3.85 | 3.71 | 5.3 | 5.51 | 5.27 | 5.44 |
| 58 | 4.54 | 4.86 | 4.19 | 4.42 | 4.46 | 4.35 | 3.62 | 3.41 | 5.83 | 5.61 | 4.76 | 4.51 | 3.66 | 3.72 | 4.91 | 5.41 | 4.69 | 4.96 |
| 59 | 7.02 | 7.28 | 6.85 | 7.03 | 6.61 | 6.38 | 5.72 | 5.27 | 9.41 | 9.14 | 7.99 | 7.23 | 5.05 | 5.3 | 7.84 | 7.98 | 7.69 | 7.89 |
| 60 | 6.66 | 7.12 | 5.76 | 6.15 | 6.46 | 6.22 | 5.59 | 4.99 | 8.29 | 7.73 | 6.77 | 7.05 | 4.73 | 4.93 | 7.07 | 7.75 | 6.8 | 7.17 |
| 61 | 4.41 | 4.45 | 3.79 | 3.95 | 4.26 | 4.26 | 3.97 | 3.61 | 5.57 | 5.4 | 4.43 | 4.06 | 3.5 | 3.51 | 4.82 | 5.19 | 4.75 | 4.79 |
| 62 | 6.22 | 6.26 | 5.95 | 6.19 | 6.6 | 6.23 | 6.06 | 5.8 | 9.01 | 8.48 | 7.26 | 7.31 | 5.29 | 5.47 | 7.3 | 7.88 | 6.98 | 7.29 |
| 63 | 11.1 | 11.06 | 9.67 | 10.02 | 10.4 | 10.29 | 8.45 | 7.68 | 11.63 | 11.47 | 11.82 | 11.38 | 7.42 | 7.84 | 10.66 | 12.48 | 11.66 | 12.07 |
| 64 | 3.51 | 3.62 | 3.72 | 3.92 | 3.7 | 3.71 | 3.73 | 3.44 | 5.14 | 5 | 4.29 | 4.37 | 3.17 | 3.18 | 4.2 | 4.41 | 4.37 | 4.39 |
| 65 | 9.5 | 10.22 | 8.93 | 8.74 | 9.83 | 9.29 | 7.83 | 7.33 | 12.67 | 12.22 | 11.35 | 9.84 | 7.16 | 7.27 | 10.81 | 10.74 | 10.65 | 11.02 |
| 66 | 10.4 | 10.61 | 9.12 | 9.48 | 9.35 | 9.08 | 7.69 | 7.08 | 12.27 | 11.56 | 11.64 | 9.88 | 7.41 | 7.37 | 10.74 | 11.9 | 11.37 | 11.95 |
| 67 | 7.96 | 8.08 | 7.77 | 8.31 | 8.78 | 8.04 | 6.46 | 5.96 | 10.23 | 9.57 | 11.06 | 10.29 | 6.02 | 6.26 | 9.61 | 10.06 | 9.35 | 9.62 |
| 68 | 6.85 | 7.13 | 6.17 | 6.47 | 6.88 | 6.65 | 6.66 | 5.76 | 8.41 | 8.36 | 7.36 | 7.34 | 5.32 | 5.42 | 7.56 | 8.16 | 7.61 | 8.46 |
| 69 | 6.9 | 7.17 | 6.51 | 6.81 | 7.21 | 7.3 | 6.86 | 6.72 | 8.37 | 7.79 | 6.95 | 6.9 | 4.94 | 4.72 | 7.25 | 7.91 | 7.77 | 7.63 |
| 70 | 6.89 | 6.85 | 6.15 | 6.33 | 6.99 | 6.69 | 5.76 | 5.07 | 8.78 | 8.27 | 7.48 | 6.75 | 5.31 | 5.21 | 7.04 | 7.9 | 6.93 | 7.47 |
| 71 | 7.11 | 7.07 | 6.81 | 6.88 | 7 | 6.71 | 6.01 | 5.39 | 8.6 | 8.37 | 7.43 | 6.67 | 5.35 | 5.12 | 7.5 | 7.98 | 7.67 | 8.02 |
| 72 | 8.57 | 9.14 | 8.29 | 8.62 | 8.66 | 8.32 | 6.83 | 6.18 | 10.82 | 10.22 | 10.49 | 9.68 | 6.17 | 6.11 | 9.58 | 9.82 | 9.71 | 10.06 |
| 73 | 6.63 | 6.93 | 6.42 | 6.64 | 6.35 | 6.04 | 5.98 | 5.36 | 8.1 | 7.88 | 7.28 | 7.36 | 4.92 | 4.81 | 6.91 | 7.39 | 7.05 | 7.23 |
| 74 | 7.03 | 6.63 | 5.91 | 6.03 | 6.77 | 6.5 | 5.29 | 4.82 | 7.75 | 7.06 | 6.79 | 6.49 | 4.99 | 4.78 | 6.97 | 7.33 | 7.18 | 7.48 |
| 75 | 4.1 | 4.23 | 4.09 | 4.21 | 4.43 | 4.31 | 3.93 | 3.69 | 5.1 | 4.94 | 5.11 | 4.95 | 3.54 | 3.55 | 4.57 | 4.92 | 4.83 | 4.97 |
| 76 | 4.88 | 5.38 | 4.66 | 4.98 | 4.94 | 5.14 | 4.33 | 4.15 | 6.21 | 6.11 | 5.48 | 5.41 | 4.02 | 3.92 | 5.66 | 5.85 | 5.68 | 5.77 |
| 77 | 10.5 | 12.46 | 8.71 | 9.14 | 9.87 | 9.72 | 8.57 | 7.05 | 11.37 | 10.93 | 10.13 | 9.05 | 7.3 | 7.34 | 10.65 | 11.83 | 10.36 | 11.59 |
| 78 | 6.24 | 6.1 | 5.22 | 5.58 | 5.62 | 5.73 | 5.76 | 5.02 | 7.71 | 7.79 | 6.13 | 5.98 | 4.24 | 4.38 | 6.36 | 6.68 | 6.44 | 6.69 |
| 79 | 7.79 | 8.03 | 7.13 | 7.53 | 7.56 | 7.38 | 6.51 | 5.8 | 9.56 | 9.18 | 8.08 | 7.83 | 5.8 | 5.93 | 8.54 | 9.18 | 8.32 | 8.6 |
| 80 | 8.29 | 8.67 | 7.54 | 7.6 | 7.61 | 7.62 | 6.99 | 6.87 | 9.23 | 9.12 | 9.09 | 8.15 | 5.6 | 5.67 | 8.68 | 9.62 | 8.61 | 8.63 |

| Region of Interest | Middle frontal gyrus, orbital part | Middle frontal gyrus, orbital part | Middle occipital gyrus | Middle occipital gyrus | Middle temporal gyrus | Middle temporal gyrus | Occipital lobe | Occipital lobe | Olfactory cortex | Olfactory cortex | Paracentral lobule | Paracentral lobule | Parahippocampal gyrus | Parahippocampal gyrus | Parietal lobe | Parietal lobe | Postcentral gyrus | Postcentral gyrus |
| --- | --- | --- | --- | --- | --- | --- | --- | --- | --- | --- | --- | --- | --- | --- | --- | --- | --- | --- |
| Side | L | R | L | R | L | R | L | R | L | R | L | R | L | R | L | R | L | R |
| Number 1 | 5.66 | 5.52 | 4.71 | 4.91 | 4.95 | 5.16 | 5.36 | 5.55 | 4.47 | 4.49 | 4.9 | 5.28 | 3.95 | 3.94 | 5.24 | 5.31 | 4.88 | 4.83 |
| 2 | 7.49 | 7.7 | 6.78 | 7.1 | 6.91 | 7.06 | 7.51 | 7.66 | 6.21 | 6.15 | 6.58 | 6.63 | 5.25 | 5.03 | 6.75 | 6.68 | 6.59 | 6.48 |
| 3 | 7.46 | 7.13 | 6.97 | 7.39 | 7.33 | 7.18 | 8.01 | 7.96 | 5.86 | 5.77 | 7.93 | 7.42 | 5.37 | 5.52 | 7.56 | 7.12 | 6.85 | 6.45 |
| 4 | 9 | 8.84 | 7.47 | 7.85 | 7.47 | 7.93 | 8.43 | 8.72 | 6.29 | 6.17 | 7.95 | 7.56 | 6.11 | 6.17 | 7.76 | 7.92 | 7.49 | 7.65 |
| 5 | 10.31 | 10.2 | 9.35 | 10.1 | 9.36 | 9.4 | 9.75 | 9.93 | 7.54 | 7.6 | 8.69 | 8.82 | 6.96 | 6.86 | 9.83 | 9.76 | 9.09 | 9.09 |
| 6 | 7.51 | 7.25 | 5.97 | 6.7 | 6.63 | 7.05 | 6.77 | 6.99 | 5.18 | 5.48 | 6.89 | 7.04 | 5.18 | 5.17 | 7.03 | 7.09 | 6.41 | 6.48 |
| 7 | 9.63 | 9.45 | 9.11 | 9.82 | 9.24 | 9.61 | 9.65 | 9.75 | 6.99 | 7.48 | 8.17 | 8.69 | 6.6 | 6.37 | 8.98 | 8.94 | 8.12 | 8.38 |
| 8 | 6.75 | 6.67 | 5.37 | 5.91 | 6.02 | 6.12 | 6 | 6.29 | 5.29 | 5.41 | 5.69 | 5.32 | 4.44 | 4.57 | 5.91 | 5.83 | 5.55 | 5.46 |
| 9 | 9.83 | 8.94 | 7.65 | 8.59 | 8.19 | 8.4 | 8.66 | 8.68 | 7.27 | 6.71 | 8.8 | 7.92 | 5.96 | 6.4 | 8.3 | 8.24 | 7.75 | 7.8 |
| 10 | 7.6 | 6.94 | 6.32 | 6.58 | 6.69 | 6.96 | 6.88 | 7.02 | 5.42 | 5.48 | 5.82 | 5.64 | 4.94 | 4.95 | 6.54 | 6.7 | 5.76 | 5.75 |
| 11 | 12.2 | 11.45 | 10.27 | 11.83 | 10.45 | 10.66 | 11.22 | 11.76 | 8.88 | 8.46 | 9.59 | 10.91 | 8.23 | 7.48 | 10.34 | 10.59 | 8.72 | 9.51 |
| 12 | 9.39 | 9.32 | 7.97 | 8.44 | 8.51 | 8.41 | 8.47 | 8.58 | 6.31 | 6.14 | 8.33 | 8.78 | 6.17 | 6.09 | 8.49 | 8.07 | 7.57 | 7.89 |
| 13 | 10.14 | 9.93 | 9.09 | 9.44 | 8.81 | 9.18 | 9.77 | 10.09 | 7.34 | 7.33 | 9.16 | 8.59 | 6.49 | 7.07 | 8.98 | 9.1 | 8.28 | 8.42 |
| 14 | 8.46 | 7.66 | 8.07 | 8.43 | 8.59 | 8.99 | 8.91 | 9.32 | 6.63 | 6.46 | 8.35 | 8.25 | 6.38 | 6.58 | 8.52 | 8.6 | 8.2 | 8.69 |
| 15 | 4.61 | 4.48 | 4.27 | 4.45 | 4.16 | 4.37 | 4.71 | 4.81 | 3.77 | 3.42 | 4.57 | 4.7 | 3.2 | 3.27 | 4.33 | 4.38 | 4.15 | 4.27 |
| 16 | 5.04 | 4.66 | 4.28 | 4.54 | 4.6 | 4.63 | 4.57 | 4.69 | 4.1 | 3.82 | 4.89 | 4.4 | 3.77 | 3.66 | 4.75 | 4.73 | 4.48 | 4.41 |
| 17 | 8.71 | 8.35 | 7.27 | 8.05 | 7.53 | 8.1 | 7.87 | 8.3 | 6.84 | 7.02 | 7.17 | 7.63 | 5.63 | 5.96 | 8.07 | 8.15 | 7.12 | 7.3 |
| 18 | 9.58 | 9.69 | 8.68 | 9.4 | 9.03 | 9.14 | 9.22 | 9.61 | 7.02 | 6.74 | 8.83 | 8.53 | 6.77 | 6.81 | 9.16 | 9.27 | 8.04 | 8.34 |
| 19 | 11.53 | 10.79 | 9.58 | 10.04 | 11.11 | 10.57 | 10.57 | 11 | 8.66 | 8.36 | 8.06 | 8.37 | 7.31 | 7.87 | 9.94 | 10.13 | 8.22 | 8.98 |
| 20 | 7.83 | 7.78 | 7.42 | 8.16 | 7.26 | 7.39 | 7.87 | 8.26 | 6.62 | 6.32 | 7.39 | 7.32 | 5.59 | 5.81 | 7.38 | 7.06 | 6.84 | 6.9 |
| 21 | 12.12 | 11.37 | 10.29 | 11 | 10.86 | 11.26 | 11.05 | 11.59 | 7.86 | 8.2 | 11.69 | 10.86 | 8.6 | 8.6 | 10.98 | 10.76 | 9.8 | 10.3 |
| 22 | 9.45 | 9.3 | 8.05 | 8.81 | 8.59 | 8.92 | 8.74 | 9.07 | 7.25 | 7.57 | 7.62 | 8.21 | 6.58 | 6.63 | 8.66 | 8.82 | 7.9 | 7.87 |
| 23 | 6.95 | 6.58 | 6 | 6.86 | 6.34 | 6.54 | 6.94 | 6.99 | 4.66 | 4.53 | 6.76 | 6.01 | 4.94 | 4.82 | 6.7 | 6.63 | 6.21 | 6.01 |
| 24 | 8.04 | 8 | 6.62 | 7.54 | 7.39 | 7.43 | 7.27 | 7.74 | 5.96 | 7.23 | 7.35 | 6.63 | 5.39 | 6.43 | 7.4 | 7.29 | 6.8 | 6.86 |
| 25 | 7.27 | 6.44 | 5.45 | 5.78 | 6.65 | 6.56 | 5.69 | 5.73 | 4.75 | 5.2 | 6.4 | 6.42 | 4.97 | 5.01 | 6.12 | 6.12 | 5.82 | 5.92 |
| 26 | 8.19 | 7.76 | 7.48 | 7.78 | 7.83 | 7.9 | 7.67 | 7.91 | 5.76 | 5.92 | 7.19 | 7.07 | 5.36 | 6.04 | 7.33 | 7.2 | 6.91 | 6.86 |
| 27 | 6.41 | 6.28 | 5.68 | 6.5 | 6.15 | 6.14 | 6.68 | 6.93 | 5.33 | 4.95 | 6.35 | 6.24 | 4.7 | 4.82 | 5.94 | 5.86 | 5.87 | 5.66 |
| 28 | 6.06 | 5.59 | 5.39 | 5.57 | 5.36 | 5.51 | 5.85 | 6.08 | 4.17 | 4.39 | 5.38 | 5.4 | 4.39 | 4.43 | 5.35 | 5.28 | 4.88 | 4.98 |
| 29 | 8.59 | 8.17 | 7.41 | 8.1 | 7.92 | 7.93 | 8.2 | 8.85 | 6.26 | 6.32 | 7.37 | 7.63 | 5.84 | 5.99 | 7.69 | 7.59 | 7.23 | 7.35 |
| 30 | 7.56 | 7.14 | 5.82 | 6.34 | 6.53 | 6.48 | 6.28 | 6.59 | 4.91 | 5.01 | 5.89 | 6.11 | 4.74 | 4.66 | 6.54 | 6.39 | 5.7 | 5.77 |
| 31 | 6.78 | 6.65 | 6.08 | 5.97 | 6.13 | 6.43 | 6.64 | 6.64 | 5.14 | 4.7 | 6.41 | 6.56 | 4.86 | 4.79 | 6.36 | 6.33 | 5.87 | 5.62 |
| 32 | 9.37 | 8.32 | 8.08 | 8.29 | 8.1 | 8.35 | 8.41 | 8.59 | 6.01 | 6.46 | 7.48 | 6.65 | 6.59 | 6.42 | 7.78 | 7.64 | 7.63 | 7.28 |
| 33 | 3.56 | 3.62 | 3.63 | 3.96 | 3.63 | 3.68 | 3.96 | 4.14 | 2.87 | 2.84 | 3.48 | 3.42 | 2.8 | 2.82 | 3.52 | 3.41 | 3.46 | 3.39 |
| 34 | 9.18 | 8.33 | 7.83 | 7.7 | 8 | 7.85 | 8.46 | 8.12 | 5.49 | 5.78 | 7.56 | 6.53 | 5.52 | 5.57 | 7.79 | 7.61 | 7.41 | 7.51 |
| 35 | 6.67 | 6.29 | 6.23 | 6.51 | 6.82 | 6.79 | 6.84 | 6.94 | 4.89 | 4.9 | 6.23 | 5.99 | 4.73 | 4.66 | 6.61 | 6.63 | 5.87 | 5.58 |
| 36 | 6.96 | 7.1 | 6.13 | 6.78 | 6.69 | 6.8 | 6.79 | 7 | 5.63 | 6.12 | 5.84 | 6.11 | 4.86 | 5.38 | 6.59 | 6.66 | 5.87 | 6.13 |
| 37 | 7.71 | 7.62 | 7.78 | 8.14 | 7.62 | 7.52 | 8.03 | 8.31 | 5.5 | 5.82 | 6.9 | 6.92 | 4.78 | 5.17 | 7.52 | 7.25 | 6.88 | 6.73 |
| 38 | 11.25 | 10.87 | 10.69 | 10.97 | 10.37 | 10.41 | 10.83 | 11 | 7.83 | 6.87 | 8.82 | 8.79 | 8.29 | 8.52 | 10.37 | 10.26 | 9.48 | 9.26 |
| 39 | 7.68 | 7.23 | 6.49 | 7 | 6.66 | 6.67 | 6.93 | 7.13 | 5.57 | 5.35 | 6.1 | 6.31 | 4.84 | 4.83 | 6.62 | 6.38 | 6.42 | 6.23 |
| 40 | 9.06 | 8.82 | 7.44 | 8.1 | 7.98 | 8.36 | 8.01 | 8.27 | 6.88 | 6.35 | 7.57 | 7.99 | 5.48 | 5.96 | 8.53 | 8.54 | 7.3 | 7.39 |
| 41 | 8.93 | 8.2 | 7.77 | 8.28 | 8.45 | 8.42 | 8.6 | 8.98 | 7.36 | 7.03 | 8.61 | 8.1 | 6 | 5.88 | 8.49 | 8.47 | 7.86 | 7.74 |
| 42 | 11.33 | 10.82 | 8.77 | 9.47 | 10.18 | 9.54 | 9.81 | 9.88 | 7.32 | 6.97 | 9.38 | 8.92 | 7.13 | 7.54 | 9.7 | 9.66 | 8.42 | 8.27 |
| 43 | 6.36 | 5.87 | 5.67 | 6.12 | 6.13 | 6.18 | 6.44 | 6.52 | 5.63 | 5.19 | 6.12 | 5.82 | 5.05 | 5.02 | 6.19 | 6.18 | 5.54 | 5.4 |
| 44 | 5.75 | 5.98 | 4.89 | 5.5 | 5.5 | 5.42 | 5.38 | 5.73 | 3.94 | 4.4 | 5.35 | 5.18 | 3.96 | 3.75 | 5.2 | 5.18 | 4.75 | 4.78 |
| 45 | 4.48 | 4.41 | 3.86 | 4.14 | 4.26 | 4.24 | 4.19 | 4.28 | 3.63 | 3.52 | 4.23 | 4.37 | 3.55 | 3.58 | 4.27 | 4.29 | 3.87 | 3.99 |
| 46 | 7.89 | 7.7 | 7.12 | 7.89 | 7.22 | 7.46 | 7.7 | 7.78 | 5.5 | 5.42 | 7.38 | 7.38 | 4.93 | 5.7 | 7.26 | 7.45 | 6.78 | 7.1 |
| 47 | 8.59 | 8.68 | 7.23 | 7.84 | 7.9 | 8.15 | 7.95 | 8.15 | 5.34 | 5.75 | 7.42 | 7 | 6.2 | 6.37 | 7.78 | 7.93 | 6.93 | 7.07 |
| 48 | 10.65 | 10.71 | 9 | 9.83 | 9.89 | 10.77 | 10.13 | 10.86 | 7.02 | 7.48 | 9.52 | 8.39 | 7.47 | 7.7 | 9.97 | 10.11 | 8.72 | 9.52 |
| 49 | 5.34 | 5.63 | 4.7 | 5.08 | 4.98 | 5.03 | 5.06 | 5.29 | 4.37 | 4.41 | 4.86 | 5.23 | 3.92 | 4.04 | 5.05 | 4.93 | 4.65 | 4.37 |
| 50 | 10.93 | 11.02 | 10.21 | 11.45 | 10.64 | 10.61 | 11.08 | 11.95 | 8.16 | 8.22 | 9.9 | 9.94 | 8.04 | 8.08 | 10.84 | 10.34 | 9.53 | 9.62 |
| 51 | 6.18 | 6.24 | 5.49 | 6.03 | 5.77 | 5.86 | 5.86 | 6.1 | 3.96 | 4.33 | 6.01 | 5.91 | 4.47 | 4.55 | 5.75 | 5.95 | 5.59 | 5.38 |
| 52 | 9.08 | 8.5 | 7.55 | 7.92 | 8.13 | 8.1 | 7.91 | 8.34 | 5.76 | 5.77 | 7.1 | 7.38 | 5.92 | 5.89 | 8 | 7.99 | 7.12 | 6.89 |
| 53 | 10.05 | 10.85 | 7.96 | 9.06 | 9.41 | 9.92 | 9.21 | 9.6 | 6.53 | 6.8 | 8.53 | 7.88 | 6.52 | 6.51 | 9.29 | 9.55 | 8.19 | 7.98 |
| 54 | 8.94 | 8.27 | 7.88 | 8.84 | 7.81 | 7.95 | 7.96 | 8.25 | 6.58 | 6.34 | 7.31 | 6.94 | 5.57 | 5.55 | 7.94 | 7.84 | 7.02 | 7.13 |
| 55 | 5.91 | 6.02 | 5.25 | 5.77 | 5.38 | 5.65 | 5.66 | 5.93 | 4.48 | 4.43 | 5.56 | 5.81 | 4.06 | 4.19 | 5.62 | 5.51 | 5.29 | 5.09 |
| 56 | 4.88 | 4.83 | 3.96 | 4.27 | 4.23 | 4.36 | 4.33 | 4.38 | 3.55 | 3.57 | 4.04 | 4.14 | 3.31 | 3.26 | 4.12 | 4.22 | 3.87 | 3.87 |
| 57 | 5.32 | 4.56 | 4.59 | 5.29 | 5.33 | 4.94 | 5.01 | 5.26 | 4.22 | 3.96 | 4.38 | 4.45 | 3.54 | 3.59 | 5 | 4.88 | 4.89 | 4.81 |
| 58 | 4.88 | 4.8 | 4.14 | 4.61 | 4.28 | 4.52 | 4.56 | 4.7 | 3.65 | 3.78 | 4.31 | 4.63 | 3.72 | 3.83 | 4.4 | 4.42 | 4.32 | 4.38 |
| 59 | 7.53 | 7.63 | 7.1 | 7.72 | 7.45 | 7.28 | 7.53 | 7.84 | 5.6 | 5.55 | 6.71 | 6.25 | 5.27 | 5.6 | 7.39 | 7.38 | 6.9 | 6.61 |
| 60 | 6.7 | 6.61 | 5.94 | 6.5 | 6.73 | 6.74 | 6.47 | 7.12 | 5.14 | 5.15 | 6.5 | 6.08 | 4.63 | 4.92 | 6.42 | 6.7 | 6.14 | 6.08 |
| 61 | 4.88 | 4.66 | 4.02 | 4.21 | 4.32 | 4.49 | 4.22 | 4.24 | 3.66 | 3.48 | 4.08 | 4.39 | 3.47 | 3.66 | 4.33 | 4.34 | 4.05 | 4.21 |
| 62 | 7.39 | 7.3 | 6.03 | 6.4 | 6.61 | 6.66 | 6.88 | 7.15 | 5.34 | 4.99 | 6.53 | 6.43 | 5.11 | 5.6 | 6.5 | 6.56 | 6.57 | 7.01 |
| 63 | 11.88 | 10.74 | 9.97 | 10.56 | 10.41 | 10.65 | 10.72 | 11.21 | 8.33 | 8.74 | 9.64 | 10.03 | 7.87 | 8.49 | 10.82 | 10.67 | 9.36 | 9.03 |
| 64 | 4 | 3.98 | 3.59 | 4.04 | 4.1 | 4.25 | 4.12 | 4.31 | 3.49 | 3.42 | 3.6 | 3.86 | 3.17 | 3.21 | 3.92 | 3.89 | 3.55 | 3.71 |
| 65 | 10.62 | 10.32 | 8.99 | 9.63 | 9.6 | 10.13 | 10.12 | 10.58 | 8.61 | 7.84 | 8.89 | 9.18 | 7.4 | 7.48 | 9.59 | 9.58 | 9.3 | 9.44 |
| 66 | 11.26 | 11.48 | 9.59 | 10.53 | 9.82 | 10.25 | 10.41 | 10.88 | 6.62 | 7.72 | 9.28 | 8.83 | 7.91 | 8.01 | 10.02 | 9.97 | 9.25 | 9.07 |
| 67 | 9.54 | 9.27 | 8.48 | 8.58 | 8.39 | 8.53 | 9.31 | 9.61 | 7.57 | 7.25 | 8.06 | 8.26 | 5.99 | 6.35 | 8.41 | 8.25 | 7.75 | 7.74 |
| 68 | 7 | 7.02 | 6.42 | 7.33 | 6.7 | 7.05 | 6.93 | 7.48 | 5.35 | 5.24 | 6.93 | 6.92 | 5.32 | 5.45 | 6.86 | 6.98 | 6.67 | 6.75 |
| 69 | 7.6 | 7.73 | 7.15 | 7.81 | 6.97 | 7.1 | 7.05 | 7.49 | 4.96 | 5.55 | 7.14 | 7.02 | 5.15 | 4.99 | 7.02 | 7.12 | 6.68 | 6.89 |
| 70 | 6.86 | 6.82 | 6.31 | 6.89 | 6.58 | 6.67 | 6.76 | 7.18 | 5.79 | 5.78 | 6.93 | 6.52 | 5.43 | 5.44 | 6.81 | 6.86 | 6.54 | 6.74 |
| 71 | 8.11 | 7.71 | 6.81 | 7.32 | 7.61 | 7.54 | 7.19 | 7.5 | 5.73 | 5.02 | 6.58 | 6.33 | 5.45 | 5.27 | 7.24 | 7.19 | 6.55 | 6.6 |
| 72 | 8.77 | 8.63 | 8.56 | 9.41 | 8.93 | 8.7 | 9.75 | 9.88 | 6.47 | 7.34 | 8.53 | 7.48 | 6.35 | 6.23 | 8.68 | 8.53 | 8.16 | 8.19 |
| 73 | 7.07 | 6.63 | 6.35 | 6.86 | 6.6 | 6.81 | 6.74 | 7.13 | 5.59 | 5.69 | 5.9 | 5.76 | 5.17 | 5.05 | 6.6 | 6.66 | 6.07 | 6.13 |
| 74 | 6.83 | 6.65 | 6.45 | 6.43 | 6.65 | 6.5 | 6.8 | 6.87 | 5.75 | 5.38 | 6.11 | 6.44 | 4.92 | 4.92 | 6.74 | 6.64 | 6.06 | 6.18 |
| 75 | 4.87 | 4.82 | 4.15 | 4.51 | 4.3 | 4.5 | 4.62 | 4.81 | 3.32 | 3.85 | 4.32 | 4.32 | 3.56 | 3.67 | 4.25 | 4.5 | 4.05 | 4.12 |
| 76 | 5.63 | 5.58 | 4.7 | 5.1 | 5.11 | 5.29 | 5.21 | 5.45 | 4.6 | 4.2 | 4.58 | 5.29 | 4.05 | 4.12 | 5.2 | 5.24 | 4.62 | 4.91 |
| 77 | 10.22 | 9.97 | 8.21 | 9.95 | 9.69 | 9.91 | 9.45 | 10.15 | 7.03 | 7.58 | 9.14 | 9.75 | 7.77 | 7.83 | 9.53 | 10.02 | 8.75 | 8.6 |
| 78 | 6.3 | 6.09 | 5.5 | 6.02 | 5.71 | 6.01 | 5.94 | 6.26 | 4.67 | 4.9 | 5.13 | 4.67 | 4.1 | 4.48 | 6.06 | 5.92 | 5.29 | 5.46 |
| 79 | 8.36 | 8.06 | 7.08 | 7.86 | 7.71 | 8.24 | 7.7 | 8.02 | 6.44 | 6.62 | 7 | 7.29 | 6.27 | 6.45 | 7.93 | 7.84 | 6.93 | 6.89 |
| 80 | 7.63 | 7.57 | 7.41 | 8.07 | 8.08 | 8.06 | 8.06 | 8.21 | 6.31 | 6.35 | 7.34 | 8.26 | 5.65 | 5.83 | 7.99 | 8.1 | 7.32 | 7.22 |

| Region of Interest | Posterior cingulate and paracingulate gyri | Posterior cingulate and paracingulate gyri | Precentral gyrus | Precentral gyrus | Precuneus | Precuneus | Rolandic operculum | Rolandic operculum | Superior frontal gyrus, dorsolateral | Superior frontal gyrus, dorsolateral | Superior frontal gyrus, medial | Superior frontal gyrus, medial | Superior frontal gyrus, medial orbital | Superior frontal gyrus, medial orbital | Superior frontal gyrus, orbital part | Superior frontal gyrus, orbital part | Superior occipital gyrus | Superior occipital gyrus |
| --- | --- | --- | --- | --- | --- | --- | --- | --- | --- | --- | --- | --- | --- | --- | --- | --- | --- | --- |
| Side | L | R | L | R | L | R | L | R | L | R | L | R | L | R | L | R | L | R |
| Number 1 | 5.79 | 5.13 | 5.08 | 5.19 | 5.73 | 5.97 | 5.71 | 5.43 | 5.07 | 4.9 | 4.82 | 5.11 | 5.4 | 5.61 | 5.35 | 5.47 | 4.71 | 4.65 |
| 2 | 7.48 | 6.28 | 6.71 | 7.03 | 7.37 | 7.24 | 7.11 | 7.2 | 6.6 | 6.66 | 6.74 | 6.61 | 7.34 | 7.5 | 7.26 | 7.31 | 6.46 | 6.89 |
| 3 | 7.36 | 6.51 | 7.19 | 7.14 | 8.52 | 7.96 | 7.95 | 7.21 | 6.95 | 6.75 | 6.75 | 6.69 | 7.48 | 7.72 | 7.1 | 7.49 | 6.8 | 7.38 |
| 4 | 9.23 | 7.67 | 7.75 | 8.45 | 8.52 | 8.9 | 8.79 | 8.6 | 7.15 | 7.59 | 7.05 | 7.1 | 8.06 | 8.02 | 8.21 | 8.32 | 7.52 | 8.48 |
| 5 | 9.13 | 8.6 | 9.37 | 9.6 | 10.67 | 10.8 | 9.94 | 9.5 | 9.58 | 9.57 | 8.87 | 9.07 | 10.13 | 10.62 | 10.59 | 10.12 | 8.36 | 8.77 |
| 6 | 7.6 | 7.11 | 6.87 | 7.28 | 7.65 | 7.68 | 7.27 | 7.21 | 6.99 | 7.13 | 6.93 | 7.46 | 7.28 | 7.79 | 7.13 | 7.37 | 6.08 | 6.58 |
| 7 | 8.9 | 8.09 | 8.88 | 9.12 | 9.4 | 9.47 | 9.37 | 9.45 | 8.92 | 8.57 | 8.66 | 8.71 | 9.81 | 9.95 | 9.77 | 10.36 | 8.78 | 8.74 |
| 8 | 6.73 | 5.73 | 6.12 | 6.19 | 6.33 | 6.4 | 6.35 | 6.24 | 6.39 | 6.27 | 6.05 | 6.22 | 6.74 | 6.84 | 6.77 | 6.52 | 5.02 | 5.42 |
| 9 | 9.45 | 8.04 | 8.44 | 8.76 | 9.2 | 8.93 | 9.09 | 9.57 | 8.46 | 8.14 | 8.54 | 8.84 | 9.37 | 9.68 | 9.44 | 9.01 | 7.4 | 7.47 |
| 10 | 7.22 | 6.77 | 6.24 | 6.49 | 6.82 | 7.24 | 7.22 | 6.63 | 6.1 | 6.22 | 5.97 | 6.49 | 7.33 | 7.42 | 6.63 | 6.7 | 5.82 | 6.39 |
| 11 | 10.7 | 10.38 | 9.9 | 11.16 | 11.32 | 11.62 | 9.67 | 10.57 | 10.08 | 10.46 | 10.54 | 10.03 | 11.77 | 11.9 | 11.58 | 11.89 | 9.16 | 9.17 |
| 12 | 8.36 | 7.56 | 8.8 | 9 | 9.03 | 8.6 | 8.65 | 8.51 | 8.26 | 8.3 | 8.01 | 8.63 | 8.95 | 9 | 9.11 | 8.81 | 7.74 | 8.1 |
| 13 | 8.97 | 7.24 | 9.22 | 9.79 | 9.57 | 9.99 | 9.03 | 9.24 | 9.51 | 9.44 | 8.64 | 9.8 | 9.43 | 10.34 | 10.11 | 10 | 8.61 | 8.58 |
| 14 | 9.02 | 8.45 | 8.49 | 9.08 | 9.36 | 9.79 | 9.16 | 9.4 | 8.07 | 8.36 | 7.88 | 8.38 | 8.22 | 8.43 | 8.63 | 8.71 | 7.72 | 8.4 |
| 15 | 4.52 | 4.14 | 4.37 | 4.53 | 4.88 | 4.89 | 4.22 | 4.22 | 4.29 | 4.24 | 4.14 | 4.16 | 4.3 | 4.59 | 4.5 | 4.31 | 4.16 | 4.22 |
| 16 | 5.13 | 4.31 | 4.91 | 4.95 | 5.08 | 5.19 | 5.21 | 4.85 | 5.1 | 4.84 | 4.77 | 4.77 | 5.19 | 5.1 | 5.15 | 4.92 | 4.18 | 4 |
| 17 | 6.8 | 6.55 | 7.89 | 7.94 | 8.48 | 8.54 | 8.72 | 8 | 7.65 | 7.76 | 7.13 | 7.8 | 8.3 | 8.55 | 8.08 | 8.33 | 6.45 | 7.18 |
| 18 | 9.58 | 8 | 8.86 | 9.14 | 9.59 | 9.85 | 9.89 | 9.94 | 8.35 | 8.69 | 8.75 | 9.01 | 9.79 | 9.62 | 9.92 | 9.6 | 8.04 | 8.63 |
| 19 | 12.24 | 10.62 | 9.24 | 9.55 | 10.83 | 11.12 | 10.23 | 10.27 | 9.33 | 9.13 | 9.86 | 10.2 | 11.08 | 11.12 | 11.25 | 10.89 | 8.58 | 8.89 |
| 20 | 8.53 | 7.38 | 7.78 | 8.06 | 7.98 | 7.64 | 7.44 | 7.84 | 7.12 | 7.53 | 7.2 | 7.06 | 7.52 | 8.01 | 7.17 | 7.45 | 7.65 | 7.81 |
| 21 | 13.02 | 11.42 | 10.4 | 10.77 | 12.4 | 12.23 | 11.64 | 11.15 | 10.28 | 10.72 | 10.91 | 11.24 | 11.02 | 11.14 | 10.8 | 11.12 | 9.38 | 10.11 |
| 22 | 10.4 | 8.65 | 8.39 | 8.41 | 9.32 | 9.72 | 9.25 | 8.6 | 8.33 | 8.41 | 8.4 | 8.8 | 8.78 | 9.5 | 9.45 | 9.57 | 7.63 | 8.17 |
| 23 | 7.86 | 7.05 | 6.34 | 6.78 | 7.65 | 7.42 | 7.13 | 6.69 | 6.57 | 6.31 | 6.02 | 6.2 | 6.29 | 6.56 | 6.93 | 6.73 | 6.32 | 6.27 |
| 24 | 7.39 | 7.64 | 7.16 | 7.07 | 8.19 | 8.02 | 7.78 | 7.42 | 7.51 | 7.49 | 7.35 | 7.61 | 7.76 | 7.64 | 8.15 | 7.95 | 5.95 | 6.34 |
| 25 | 6.55 | 6.12 | 6.54 | 6.48 | 6.7 | 6.86 | 6.97 | 6.4 | 6.44 | 6.1 | 6.33 | 6.45 | 6.93 | 6.83 | 7.26 | 6.89 | 5.2 | 5.19 |
| 26 | 7.45 | 6.66 | 7.53 | 7.78 | 7.96 | 7.57 | 8.11 | 7.74 | 7.4 | 7.08 | 7.31 | 7.41 | 7.6 | 8.25 | 8.27 | 7.76 | 6.67 | 6.71 |
| 27 | 6.91 | 6.35 | 6.21 | 6.22 | 6.45 | 6.54 | 6.58 | 6.15 | 6.12 | 5.84 | 5.8 | 5.82 | 6.13 | 6.31 | 6.58 | 6.65 | 5.8 | 6.02 |
| 28 | 5.14 | 4.57 | 5.46 | 5.71 | 5.68 | 5.81 | 4.87 | 5.41 | 5.5 | 5.49 | 5.49 | 5.45 | 5.6 | 5.66 | 5.84 | 6.04 | 5.1 | 5.53 |
| 29 | 7.07 | 6.14 | 7.82 | 8.46 | 8.26 | 8.34 | 7.91 | 8.68 | 7.8 | 7.46 | 7.59 | 7.82 | 7.79 | 8.38 | 8.3 | 8.21 | 6.9 | 7.18 |
| 30 | 6.36 | 6.13 | 5.92 | 6.23 | 7.08 | 6.98 | 6.46 | 6.53 | 6.41 | 6.09 | 6.15 | 6.31 | 7.06 | 6.64 | 7.13 | 6.79 | 5.56 | 6.07 |
| 31 | 6.95 | 5.73 | 6.15 | 6.29 | 6.84 | 6.99 | 6.32 | 6.36 | 6.34 | 6.05 | 6.36 | 6.3 | 6.6 | 6.55 | 6.52 | 6.32 | 5.64 | 5.96 |
| 32 | 6.68 | 5.56 | 8.14 | 7.95 | 8.25 | 8.01 | 8.62 | 8.45 | 8.21 | 7.97 | 7.93 | 8.03 | 8.42 | 8.63 | 8.99 | 8.79 | 7.67 | 8.37 |
| 33 | 3.67 | 3.29 | 3.63 | 3.8 | 3.75 | 3.56 | 3.56 | 3.7 | 3.55 | 3.37 | 3.52 | 3.44 | 3.45 | 3.5 | 3.61 | 3.5 | 3.62 | 3.63 |
| 34 | 7.84 | 6.84 | 7.51 | 7.65 | 8.44 | 8.56 | 7.91 | 7.94 | 7.12 | 7.03 | 6.97 | 6.92 | 7.81 | 8.11 | 8.48 | 7.55 | 7.4 | 7.49 |
| 35 | 6.29 | 5.99 | 6.53 | 6.31 | 6.96 | 7.43 | 6.77 | 6.44 | 6.69 | 6.22 | 6.47 | 6.33 | 6.81 | 6.85 | 6.73 | 6.55 | 5.72 | 6.45 |
| 36 | 7.43 | 6.97 | 6.72 | 6.74 | 7.17 | 7.26 | 7.01 | 6.74 | 6.57 | 6.57 | 6.59 | 6.55 | 7.19 | 7.47 | 7.5 | 7.33 | 5.69 | 6.45 |
| 37 | 7.38 | 6.54 | 7.08 | 7.45 | 8.26 | 7.86 | 7.57 | 7.6 | 7.17 | 6.86 | 7.02 | 6.92 | 7.45 | 7.76 | 7.67 | 7.71 | 7.34 | 7.89 |
| 38 | 11.23 | 10.2 | 9.98 | 10.32 | 11.22 | 10.96 | 9.52 | 10.28 | 10.35 | 9.99 | 9.89 | 10.18 | 10.23 | 10.24 | 11.59 | 10.79 | 9.58 | 9.79 |
| 39 | 5.73 | 5.38 | 6.96 | 7.16 | 6.96 | 6.44 | 6.94 | 6.91 | 6.85 | 6.56 | 6.63 | 6.91 | 7.23 | 7.29 | 7.19 | 6.89 | 6.2 | 6.68 |
| 40 | 8.31 | 7.5 | 8.08 | 8.52 | 9.46 | 9.6 | 8.09 | 8.5 | 8.3 | 8.19 | 8.5 | 8.56 | 9.12 | 9.01 | 9.08 | 8.83 | 7.28 | 7.29 |
| 41 | 9.74 | 8.61 | 8.92 | 8.85 | 9.16 | 9.43 | 9.33 | 9.14 | 8.74 | 8.43 | 8.46 | 8.52 | 9.53 | 9.34 | 9.01 | 8.93 | 7.36 | 8.34 |
| 42 | 10.86 | 9.01 | 9.2 | 8.98 | 10.07 | 10.14 | 9.98 | 9.16 | 9.54 | 9.15 | 9.33 | 9.49 | 10.04 | 9.95 | 10.83 | 10.86 | 8.17 | 9.25 |
| 43 | 7.51 | 7.21 | 5.9 | 6.02 | 6.74 | 6.92 | 6.31 | 6.07 | 5.98 | 5.79 | 6.5 | 6.28 | 6.58 | 6.75 | 6.84 | 6.62 | 5.31 | 5.9 |
| 44 | 4.95 | 4.29 | 5.1 | 5.35 | 5.53 | 5.43 | 5.61 | 5.65 | 4.63 | 5 | 5.1 | 5.18 | 5.72 | 5.57 | 5.6 | 5.5 | 4.83 | 5.61 |
| 45 | 4.33 | 4.28 | 4.22 | 4.31 | 4.51 | 4.7 | 4.25 | 4.17 | 4.28 | 4.12 | 4.2 | 4.36 | 4.45 | 4.33 | 4.56 | 4.48 | 3.77 | 3.76 |
| 46 | 7.68 | 6.73 | 7.52 | 7.85 | 8.15 | 8.16 | 6.94 | 7.14 | 7.5 | 7.62 | 6.97 | 7.47 | 7.71 | 7.63 | 7.84 | 7.59 | 6.79 | 7.07 |
| 47 | 8.01 | 7.6 | 7.61 | 7.66 | 8.56 | 8.72 | 8.02 | 7.65 | 7.98 | 7.41 | 7.67 | 7.47 | 8.3 | 7.91 | 8.67 | 8.19 | 6.57 | 6.74 |
| 48 | 9.87 | 9.26 | 10.02 | 10.24 | 10.97 | 11.08 | 10.17 | 10.45 | 9.99 | 9.82 | 9.78 | 10.47 | 10.95 | 11.04 | 10.37 | 10.87 | 8.81 | 9.67 |
| 49 | 5.22 | 4.8 | 5.14 | 4.93 | 5.33 | 5.35 | 4.83 | 5.15 | 5 | 4.89 | 4.93 | 5.03 | 5.25 | 5.4 | 5.44 | 5.07 | 4.53 | 4.44 |
| 50 | 11.04 | 10.28 | 10.49 | 10.67 | 11.81 | 11.31 | 10.69 | 10.69 | 10.69 | 10.28 | 10.96 | 10.49 | 12.12 | 11.88 | 11.43 | 11.01 | 9.28 | 10.11 |
| 51 | 5.64 | 5.53 | 5.93 | 6.22 | 6.25 | 6.44 | 6.05 | 5.81 | 5.7 | 5.76 | 5.88 | 6.01 | 6.07 | 6.08 | 6.06 | 6.02 | 5.08 | 5.74 |
| 52 | 7.95 | 6.96 | 7.75 | 7.6 | 8.67 | 8.81 | 8.21 | 8.14 | 6.84 | 7.2 | 7.58 | 7.65 | 8.43 | 7.86 | 8.75 | 8.07 | 7.21 | 7.45 |
| 53 | 10.55 | 10.05 | 8.62 | 9.1 | 10.11 | 10.65 | 9.5 | 9.47 | 9.01 | 8.66 | 9.24 | 9.68 | 9.94 | 9.83 | 10.05 | 9.54 | 7.69 | 8.01 |
| 54 | 6.82 | 5.89 | 7.81 | 7.94 | 8.26 | 8.36 | 8.26 | 7.98 | 7.57 | 7.42 | 7.71 | 7.59 | 8.26 | 7.92 | 8.6 | 8.6 | 7.42 | 7.99 |
| 55 | 5.54 | 5.04 | 5.46 | 5.49 | 6.09 | 6 | 5.71 | 5.36 | 5.29 | 5.19 | 5.19 | 5.21 | 5.4 | 5.76 | 5.92 | 5.85 | 4.78 | 5.54 |
| 56 | 4.84 | 4.02 | 4.1 | 4.37 | 4.48 | 4.66 | 4.3 | 4.39 | 4.06 | 4.14 | 4.12 | 4.35 | 4.38 | 4.54 | 4.56 | 4.47 | 3.84 | 4.18 |
| 57 | 4.32 | 3.88 | 4.92 | 4.96 | 5 | 4.92 | 5.27 | 5.11 | 4.9 | 4.75 | 4.75 | 4.65 | 5.37 | 5.25 | 5.3 | 5.29 | 4.5 | 5.02 |
| 58 | 4.93 | 4.67 | 4.51 | 4.74 | 4.8 | 4.87 | 4.47 | 4.58 | 4.35 | 4.24 | 4.33 | 4.38 | 4.81 | 4.73 | 4.78 | 4.69 | 4.08 | 4.22 |
| 59 | 6.5 | 5.96 | 7.23 | 7.17 | 8.15 | 8.23 | 7.61 | 7.33 | 7.07 | 6.96 | 6.95 | 6.83 | 7.68 | 7.53 | 7.52 | 7.51 | 6.56 | 6.99 |
| 60 | 6.4 | 6.55 | 6.71 | 6.76 | 6.76 | 7.38 | 6.72 | 6.46 | 6.31 | 6.49 | 6.74 | 6.74 | 6.54 | 6.86 | 6.68 | 6.86 | 5.94 | 6.6 |
| 61 | 4.2 | 3.67 | 4.25 | 4.49 | 4.77 | 4.91 | 4.5 | 4.7 | 4.36 | 4.25 | 4.26 | 4.24 | 4.49 | 4.54 | 4.78 | 4.62 | 3.84 | 3.86 |
| 62 | 8.47 | 7.99 | 6.44 | 6.92 | 7.49 | 7.52 | 6.76 | 6.68 | 6.48 | 6.61 | 6.06 | 6.5 | 6.44 | 6.78 | 6.99 | 7.07 | 5.91 | 6.36 |
| 63 | 10 | 9.82 | 10.03 | 10.43 | 11.89 | 11.88 | 10.63 | 11.5 | 10.81 | 10.2 | 9.91 | 10.73 | 10.75 | 10.9 | 11.52 | 11.01 | 9.02 | 10.08 |
| 64 | 4.58 | 4.34 | 3.9 | 3.93 | 4.49 | 4.29 | 4.09 | 4.2 | 3.97 | 3.82 | 4.01 | 3.91 | 4.15 | 3.98 | 4.1 | 4.02 | 3.69 | 3.93 |
| 65 | 9 | 7.69 | 9.79 | 9.82 | 10.38 | 10.32 | 10.51 | 9.98 | 9.87 | 9.39 | 9.44 | 9.68 | 10.58 | 11.35 | 10.7 | 9.86 | 9 | 9.41 |
| 66 | 9.79 | 9.08 | 9.63 | 10.29 | 10.5 | 10.63 | 10.21 | 10.11 | 10.3 | 10.09 | 9.27 | 9.24 | 9.89 | 10.63 | 10.79 | 10.93 | 8.64 | 9.72 |
| 67 | 8.75 | 8.52 | 8.55 | 8.76 | 9.54 | 9.46 | 9.2 | 9.09 | 8.54 | 8.31 | 8.16 | 8.33 | 9.23 | 9.27 | 9.64 | 9 | 7.55 | 7.99 |
| 68 | 7.29 | 6.93 | 7.25 | 7.54 | 7.26 | 7.56 | 7.2 | 7.59 | 7.08 | 7.21 | 7.15 | 7.45 | 7.15 | 7.18 | 6.85 | 7.32 | 6.25 | 6.97 |
| 69 | 7.63 | 6.36 | 6.82 | 7.14 | 7.71 | 7.98 | 7.14 | 7.49 | 7.15 | 6.68 | 6.59 | 6.06 | 7.33 | 7.25 | 7.62 | 7.36 | 6.39 | 7.23 |
| 70 | 7.19 | 6.53 | 6.73 | 6.84 | 7.28 | 7.56 | 7.37 | 7.42 | 6.6 | 6.58 | 6.45 | 6.75 | 6.98 | 7.18 | 6.58 | 7.18 | 6.09 | 6.4 |
| 71 | 7.48 | 6.87 | 6.8 | 6.88 | 7.69 | 7.84 | 7.41 | 7.31 | 6.87 | 6.57 | 6.59 | 6.81 | 7.68 | 7.82 | 8.09 | 7.81 | 6.05 | 7.13 |
| 72 | 9.86 | 7.77 | 9.04 | 9.04 | 9.38 | 9.25 | 9.07 | 9.09 | 8.76 | 8.46 | 9.09 | 9.27 | 9.5 | 9.73 | 9.67 | 9.66 | 8.35 | 8.19 |
| 73 | 7.26 | 6.42 | 6.46 | 6.35 | 7.03 | 6.99 | 6.74 | 6.55 | 6.35 | 6.12 | 6.16 | 6.46 | 6.98 | 6.98 | 7.25 | 6.77 | 5.46 | 6.59 |
| 74 | 7.2 | 7.37 | 6.55 | 6.58 | 6.85 | 7.02 | 6.72 | 6.67 | 6.68 | 6.34 | 6.35 | 6.59 | 7.15 | 7.22 | 6.98 | 7.05 | 5.78 | 6.35 |
| 75 | 4.4 | 4.1 | 4.34 | 4.52 | 4.7 | 5 | 4.57 | 4.74 | 4.35 | 4.56 | 4.47 | 4.59 | 4.61 | 4.74 | 4.6 | 4.78 | 4.02 | 4.26 |
| 76 | 5.58 | 4.9 | 5 | 5.2 | 5.71 | 5.91 | 5.51 | 5.31 | 5.12 | 5.12 | 5.12 | 5.05 | 5.55 | 5.47 | 5.35 | 5.66 | 4.55 | 5.11 |
| 77 | 10.97 | 10.71 | 9.21 | 9.9 | 9.79 | 10.34 | 9.14 | 9.51 | 9.48 | 9.91 | 9.41 | 10.02 | 9.39 | 10.04 | 10.37 | 10.7 | 8.04 | 9.16 |
| 78 | 6.84 | 6.42 | 5.8 | 6.08 | 6.42 | 6.27 | 5.93 | 6.27 | 5.68 | 5.89 | 5.43 | 5.79 | 5.73 | 6.12 | 5.85 | 6.19 | 5.3 | 5.75 |
| 79 | 8.42 | 8.12 | 7.44 | 7.8 | 8.63 | 8.66 | 8.18 | 7.92 | 7.32 | 7.53 | 7.25 | 7.75 | 8.18 | 8.23 | 8.26 | 8.44 | 7.11 | 6.91 |
| 80 | 8.28 | 7.4 | 7.9 | 8.27 | 8.71 | 8.9 | 7.97 | 7.88 | 7.7 | 7.63 | 7.79 | 7.81 | 7.78 | 7.99 | 7.73 | 7.96 | 7.07 | 7.47 |

| Region of Interest | Superior parietal gyrus | Superior parietal gyrus | Superior temporal gyrus | Superior temporal gyrus | Supplementary motor area | Supplementary motor area | Supramarginal gyrus | Supramarginal gyrus | Temporal lobe | Temporal lobe | Temporal pole: middle temporal gyrus | Temporal pole: middle temporal gyrus | Temporal pole: superior temporal gyrus | Temporal pole: superior temporal gyrus | Thalamus | Thalamus |
| --- | --- | --- | --- | --- | --- | --- | --- | --- | --- | --- | --- | --- | --- | --- | --- | --- |
| Side | L | R | L | R | L | R | L | R | L | R | L | R | L | R | L | R |
| Number 1 | 4.62 | 4.2 | 5.16 | 5.24 | 5.28 | 5.52 | 5.19 | 5.22 | 5.02 | 5.17 | 4.31 | 4.02 | 4.02 | 3.76 | 6.12 | 6.02 |
| 2 | 5.76 | 5.51 | 7.1 | 6.92 | 6.98 | 7.1 | 6.5 | 6.99 | 6.92 | 7 | 5.69 | 5.76 | 4.98 | 5.21 | 8.27 | 8.07 |
| 3 | 6.32 | 5.23 | 7.23 | 6.66 | 7.47 | 7.51 | 7.1 | 7.02 | 7.18 | 6.92 | 5.37 | 5.28 | 5.3 | 4.85 | 8.38 | 8.03 |
| 4 | 6.25 | 5.99 | 8.51 | 8.55 | 7.82 | 7.74 | 7.42 | 8.12 | 7.61 | 8 | 5.77 | 5.84 | 5.03 | 5.47 | 9.71 | 9.36 |
| 5 | 8.35 | 8.1 | 9.38 | 9.18 | 9.65 | 10.03 | 9.3 | 9.88 | 9.35 | 9.21 | 7.09 | 7.22 | 7.53 | 6.71 | 9.6 | 9.38 |
| 6 | 5.99 | 5.96 | 7.04 | 6.69 | 7.58 | 7.72 | 6.79 | 6.62 | 6.66 | 6.82 | 5.43 | 5.78 | 4.82 | 4.76 | 7.83 | 7.7 |
| 7 | 7.47 | 7.1 | 9.13 | 9.44 | 9.39 | 9.15 | 9.39 | 9.25 | 9.05 | 9.41 | 6.99 | 7.09 | 6.17 | 6.06 | 9.98 | 10.17 |
| 8 | 5.17 | 4.74 | 6.32 | 6.31 | 6.65 | 6.11 | 5.81 | 5.87 | 6 | 6.07 | 4.93 | 5.16 | 4.54 | 4.74 | 6.13 | 5.86 |
| 9 | 7.19 | 6.43 | 8.77 | 8.38 | 8.76 | 8.9 | 8.04 | 8.53 | 8.36 | 8.41 | 6.7 | 6.27 | 6.54 | 6.29 | 9.32 | 9.35 |
| 10 | 5.72 | 5.46 | 6.84 | 6.54 | 6.39 | 6.18 | 6.69 | 6.9 | 6.67 | 6.67 | 5.49 | 5.7 | 5.01 | 4.89 | 6.9 | 6.86 |
| 11 | 8.3 | 8.67 | 10.44 | 11.05 | 10.89 | 10.94 | 9.56 | 10.56 | 10.28 | 10.64 | 8.54 | 8.95 | 7.59 | 7.44 | 12.27 | 12.13 |
| 12 | 7.24 | 6.55 | 8.99 | 8.67 | 8.37 | 8.4 | 8.79 | 8.57 | 8.46 | 8.32 | 6.39 | 5.95 | 5.91 | 5.44 | 9.71 | 9.36 |
| 13 | 7.51 | 6.76 | 9.3 | 9.42 | 9.91 | 9.99 | 8.89 | 9.19 | 8.79 | 9.21 | 6.9 | 6.65 | 6.24 | 6.58 | 10.14 | 9.95 |
| 14 | 7.08 | 6.86 | 9.26 | 9.07 | 8.51 | 8.92 | 7.9 | 8.48 | 8.51 | 8.75 | 6.19 | 6.42 | 6.03 | 6.05 | 9.44 | 9.24 |
| 15 | 3.7 | 3.84 | 4.4 | 4.11 | 4.75 | 4.86 | 3.91 | 4.05 | 4.15 | 4.19 | 3.62 | 3.6 | 3.01 | 3.3 | 4.76 | 4.6 |
| 16 | 4.08 | 4.07 | 4.88 | 4.49 | 5.03 | 4.8 | 4.7 | 4.68 | 4.65 | 4.58 | 4.03 | 3.96 | 3.65 | 3.78 | 5.39 | 5.14 |
| 17 | 6.89 | 6.49 | 8.54 | 8.01 | 8.18 | 8.08 | 8.13 | 8.29 | 7.74 | 8.01 | 5.16 | 4.93 | 5.63 | 5.42 | 8.25 | 7.44 |
| 18 | 7.74 | 7.2 | 9.11 | 9.17 | 9.54 | 9.31 | 8.73 | 9.27 | 8.87 | 9.04 | 6.79 | 6.55 | 6.87 | 6.39 | 10.12 | 10.3 |
| 19 | 7.98 | 7.57 | 10.84 | 11.21 | 9.65 | 9.08 | 9.67 | 10.13 | 10.62 | 10.71 | 9.15 | 8.71 | 8.07 | 8.2 | 11.21 | 10.96 |
| 20 | 6.84 | 5.93 | 7.48 | 7.4 | 7.89 | 7.83 | 7.12 | 7.44 | 7.26 | 7.33 | 6.28 | 6.3 | 5.96 | 5.31 | 8.87 | 8.96 |
| 21 | 8.59 | 8.03 | 11.57 | 10.93 | 12 | 11.99 | 11.05 | 10.94 | 10.85 | 10.88 | 9.1 | 8.62 | 7.5 | 8.2 | 12.67 | 12.23 |
| 22 | 7.49 | 6.95 | 8.79 | 8.11 | 8.7 | 8.72 | 8.54 | 8.87 | 8.42 | 8.43 | 6.96 | 6.94 | 6.41 | 6.45 | 9.09 | 8.83 |
| 23 | 5.97 | 5.55 | 6.55 | 6.23 | 7.11 | 6.76 | 6.4 | 6.43 | 6.31 | 6.38 | 4.82 | 4.69 | 4.21 | 4.71 | 7.71 | 6.95 |
| 24 | 6.35 | 6.26 | 7.65 | 7.05 | 7.86 | 7.67 | 7.4 | 6.82 | 7.48 | 7.21 | 6.1 | 5.7 | 5.79 | 5.73 | 7.79 | 7.08 |
| 25 | 4.88 | 4.66 | 7.09 | 6.57 | 6.54 | 6.69 | 6.57 | 6.3 | 6.58 | 6.36 | 5.03 | 5.2 | 4.82 | 4.98 | 6.93 | 6.81 |
| 26 | 6.2 | 5.88 | 8.25 | 8.42 | 7.63 | 7.36 | 7.14 | 7 | 7.65 | 7.73 | 5.7 | 5.88 | 5.82 | 6.05 | 8.76 | 8.25 |
| 27 | 5.23 | 4.98 | 6.53 | 5.96 | 6.36 | 6.3 | 5.7 | 5.84 | 6.18 | 5.98 | 4.99 | 4.91 | 4.66 | 4.6 | 7.32 | 7.33 |
| 28 | 4.7 | 4.01 | 5.49 | 5.68 | 5.87 | 5.81 | 5.49 | 5.69 | 5.33 | 5.62 | 4.73 | 4.55 | 4.13 | 4.34 | 6.37 | 6.5 |
| 29 | 6.49 | 6.06 | 7.74 | 8.09 | 8.06 | 8.02 | 7.6 | 7.72 | 7.69 | 7.9 | 6.55 | 6.49 | 5.88 | 6.14 | 7.24 | 6.38 |
| 30 | 5.58 | 5.14 | 6.8 | 6.52 | 6.32 | 6.61 | 6.43 | 6.58 | 6.53 | 6.45 | 5.02 | 5.15 | 4.73 | 4.64 | 6.44 | 6.57 |
| 31 | 5.41 | 4.91 | 6.29 | 6.04 | 6.72 | 6.58 | 6.09 | 6.28 | 6.15 | 6.22 | 4.98 | 4.84 | 4.67 | 4.53 | 7.13 | 7.15 |
| 32 | 6.47 | 6 | 8.3 | 8.01 | 7.75 | 7.66 | 8.54 | 8.15 | 8.25 | 8.19 | 6.44 | 6.36 | 5.69 | 5.74 | 9.27 | 9.14 |
| 33 | 3.32 | 3.12 | 3.67 | 3.61 | 3.65 | 3.65 | 3.48 | 3.46 | 3.56 | 3.67 | 2.91 | 3.01 | 2.67 | 2.91 | 4.24 | 4.07 |
| 34 | 6.11 | 5.5 | 7.83 | 7.78 | 7.61 | 7.21 | 7.99 | 7.94 | 7.91 | 7.8 | 5.75 | 6 | 5.41 | 5.43 | 9.13 | 8.71 |
| 35 | 6.05 | 5.14 | 6.52 | 6.33 | 6.59 | 6.6 | 6.14 | 6.24 | 6.63 | 6.49 | 5.09 | 4.9 | 5 | 4.33 | 6.49 | 6.33 |
| 36 | 5.37 | 5.4 | 6.73 | 6.29 | 6.9 | 6.92 | 6.79 | 6.71 | 6.56 | 6.55 | 5.18 | 5.19 | 5.28 | 5.2 | 7.46 | 7.14 |
| 37 | 6.21 | 5.51 | 8.05 | 7.78 | 6.94 | 6.56 | 7.18 | 7.44 | 7.49 | 7.43 | 6.14 | 5.97 | 5.32 | 5.41 | 8.42 | 8.08 |
| 38 | 8.74 | 8.34 | 10.53 | 10.63 | 10.67 | 10.37 | 9.85 | 9.91 | 10.2 | 10.32 | 8.25 | 8.05 | 8.15 | 7.89 | 10.78 | 10.49 |
| 39 | 6 | 5.61 | 6.71 | 6.6 | 7.07 | 6.95 | 6.71 | 6.87 | 6.57 | 6.58 | 5.32 | 4.79 | 4.76 | 4.59 | 7.52 | 7.41 |
| 40 | 7.08 | 7.23 | 7.92 | 8.19 | 8.88 | 8.42 | 7.86 | 8.13 | 7.75 | 8.17 | 6.26 | 6.11 | 5.53 | 6.1 | 8.9 | 8.83 |
| 41 | 7.36 | 6.96 | 8.44 | 8.81 | 9.13 | 8.71 | 8.2 | 8.35 | 8.28 | 8.46 | 6.56 | 6.37 | 6.23 | 5.5 | 8.74 | 8.69 |
| 42 | 8.25 | 7.66 | 9.75 | 9.26 | 9.8 | 9.95 | 9.42 | 9.39 | 9.92 | 9.36 | 7.81 | 7.51 | 6.8 | 6.4 | 10.72 | 9.7 |
| 43 | 5.46 | 5.16 | 6.04 | 5.9 | 6.52 | 6.41 | 6.02 | 5.93 | 6.06 | 6.03 | 5.29 | 5.12 | 5.25 | 5.1 | 6.77 | 6.51 |
| 44 | 4.64 | 3.99 | 5.56 | 5.73 | 5.55 | 5.59 | 5.25 | 5.7 | 5.38 | 5.49 | 4.24 | 4.23 | 3.37 | 3.82 | 5.79 | 5.61 |
| 45 | 3.58 | 3.6 | 4.21 | 4.09 | 4.42 | 4.49 | 4.25 | 4.26 | 4.17 | 4.14 | 3.58 | 3.44 | 3.23 | 3.15 | 4.7 | 4.64 |
| 46 | 6.28 | 6.01 | 7.04 | 7.31 | 8.02 | 8.37 | 6.57 | 7.68 | 7.06 | 7.36 | 5.3 | 6.23 | 4.5 | 5.52 | 8.03 | 7.48 |
| 47 | 6.48 | 6.01 | 7.7 | 7.78 | 8.04 | 7.42 | 7.82 | 8.34 | 7.76 | 7.94 | 5.9 | 5.91 | 5.84 | 5.74 | 7.7 | 7.55 |
| 48 | 8.39 | 8.09 | 11.12 | 10.17 | 10.55 | 11.06 | 9.97 | 10.32 | 9.99 | 10.4 | 7.65 | 7.69 | 6.87 | 7.16 | 10.12 | 10.22 |
| 49 | 4.57 | 4.15 | 5.06 | 4.75 | 5.15 | 5.13 | 4.75 | 4.84 | 4.92 | 4.87 | 4.12 | 3.88 | 3.78 | 3.78 | 5.64 | 5.59 |
| 50 | 9.2 | 7.78 | 10.15 | 10.06 | 11.2 | 10.7 | 10.66 | 10.33 | 10.37 | 10.28 | 8.4 | 8.09 | 7.74 | 7.35 | 10.56 | 10.7 |
| 51 | 4.98 | 4.85 | 5.77 | 5.76 | 6.23 | 6.29 | 5.73 | 5.89 | 5.67 | 5.73 | 4.32 | 4.19 | 3.96 | 4.17 | 6.2 | 6.02 |
| 52 | 6.7 | 6.28 | 7.76 | 8.07 | 8.5 | 8.31 | 8.19 | 7.99 | 7.86 | 7.88 | 6.45 | 6.06 | 6.08 | 5.73 | 8.76 | 8.66 |
| 53 | 8.01 | 7.61 | 9.51 | 9.11 | 9.65 | 9.39 | 8.59 | 9.42 | 9.25 | 9.36 | 7.2 | 7.1 | 6.69 | 6.59 | 9.13 | 8.91 |
| 54 | 7.32 | 6.41 | 7.97 | 7.53 | 7.88 | 7.83 | 7.93 | 7.74 | 7.81 | 7.74 | 6 | 5.97 | 5.98 | 5.61 | 8.49 | 8.73 |
| 55 | 5.35 | 4.81 | 5.42 | 5.16 | 5.72 | 6.07 | 5.62 | 5.57 | 5.32 | 5.47 | 4.31 | 4.52 | 3.69 | 3.98 | 6.19 | 6.06 |
| 56 | 3.36 | 3.33 | 4.29 | 4.39 | 4.71 | 4.3 | 3.96 | 4.36 | 4.24 | 4.37 | 3.39 | 3.46 | 3.14 | 3.46 | 4.49 | 4.18 |
| 57 | 4.52 | 3.99 | 5.89 | 5.33 | 5.09 | 4.79 | 5.04 | 5.18 | 5.31 | 5.02 | 4.03 | 4.07 | 4.08 | 3.98 | 5.53 | 5.56 |
| 58 | 3.8 | 3.7 | 4.44 | 4.31 | 4.86 | 4.68 | 4.18 | 4.37 | 4.31 | 4.46 | 3.77 | 3.58 | 3.79 | 3.45 | 5.16 | 5.37 |
| 59 | 6.43 | 6.19 | 7.28 | 7.36 | 7.41 | 7.38 | 7.16 | 6.97 | 7.26 | 7.26 | 5.97 | 5.96 | 5.29 | 5.2 | 7.17 | 7.1 |
| 60 | 5.76 | 5.72 | 7.37 | 6.7 | 6.85 | 6.95 | 6.3 | 6.46 | 6.62 | 6.58 | 5.49 | 5.54 | 5.03 | 5.09 | 7.39 | 7.01 |
| 61 | 3.65 | 3.35 | 4.59 | 4.65 | 4.63 | 4.55 | 4.17 | 4.31 | 4.24 | 4.39 | 3.23 | 3.48 | 3.22 | 3.39 | 4.66 | 4.71 |
| 62 | 5.38 | 5.26 | 7.08 | 6.77 | 6.51 | 7.25 | 5.88 | 6.61 | 6.55 | 6.59 | 5.17 | 5.31 | 4.76 | 4.93 | 7.71 | 7.48 |
| 63 | 9.13 | 8.23 | 10.33 | 10.03 | 10.39 | 10.73 | 10.64 | 10.63 | 10.23 | 10.33 | 8.1 | 8.62 | 7.48 | 7.69 | 11.85 | 12.16 |
| 64 | 3.28 | 3.07 | 4.12 | 4.19 | 4.13 | 4.08 | 3.82 | 4.02 | 4 | 4.14 | 3.14 | 3.31 | 2.98 | 3.21 | 4.14 | 4.35 |
| 65 | 8.4 | 8.14 | 10.25 | 9.59 | 9.83 | 9.75 | 9.68 | 9.55 | 9.6 | 9.6 | 7.21 | 7.69 | 6.97 | 6.2 | 11.75 | 11.75 |
| 66 | 8.55 | 8.37 | 9.35 | 9.48 | 10.1 | 9.9 | 9.77 | 9.67 | 9.55 | 9.81 | 7.52 | 7.95 | 6.89 | 6.8 | 11.24 | 11.57 |
| 67 | 7.26 | 6.64 | 8.53 | 8.17 | 9.54 | 9.03 | 8.3 | 7.83 | 8.26 | 8.4 | 7.23 | 6.82 | 6.16 | 5.86 | 10.05 | 10.24 |
| 68 | 6.2 | 5.76 | 6.91 | 7.03 | 7.68 | 8.07 | 6.73 | 7.23 | 6.59 | 6.88 | 5.02 | 4.92 | 4.9 | 5.22 | 8.23 | 8.2 |
| 69 | 5.95 | 6.01 | 7.44 | 7.26 | 7.18 | 6.96 | 7.11 | 7.09 | 6.99 | 7.1 | 5.51 | 5.57 | 5.31 | 5.1 | 7.13 | 7.03 |
| 70 | 6.19 | 5.96 | 6.96 | 6.38 | 6.89 | 7.04 | 6.33 | 6.88 | 6.57 | 6.52 | 5.2 | 5.27 | 5.22 | 5.03 | 6.7 | 7.14 |
| 71 | 6.14 | 5.8 | 7.18 | 7.23 | 6.56 | 6.31 | 7.09 | 7.33 | 7.28 | 7.28 | 5.7 | 5.64 | 4.79 | 5.32 | 7.72 | 7.75 |
| 72 | 8.1 | 7.21 | 8.56 | 8.49 | 9.64 | 9.05 | 8.11 | 8.09 | 8.69 | 8.64 | 7.41 | 7.24 | 7.01 | 6.43 | 9.54 | 9 |
| 73 | 5.86 | 5.61 | 6.85 | 6.58 | 6.44 | 6.21 | 6.12 | 6.51 | 6.62 | 6.73 | 5.51 | 5.31 | 5.09 | 4.84 | 7.93 | 7.88 |
| 74 | 5.92 | 5.4 | 6.87 | 6.54 | 6.75 | 6.36 | 6.66 | 6.84 | 6.52 | 6.39 | 5.18 | 5.3 | 4.87 | 5.29 | 6.99 | 6.84 |
| 75 | 3.71 | 3.85 | 4.43 | 4.32 | 4.48 | 4.68 | 4.15 | 4.47 | 4.28 | 4.37 | 3.69 | 3.77 | 3.54 | 3.61 | 4.55 | 4.5 |
| 76 | 4.76 | 4.06 | 5.51 | 5.43 | 5.2 | 5.37 | 4.93 | 5.29 | 5.08 | 5.27 | 4.42 | 3.89 | 4.01 | 3.84 | 5.51 | 5.59 |
| 77 | 7.56 | 7.65 | 9.17 | 9.68 | 9.77 | 9.89 | 9.39 | 10.03 | 9.33 | 9.64 | 7.68 | 7.43 | 6.92 | 6.88 | 10.48 | 10.56 |
| 78 | 5.29 | 5.16 | 5.99 | 5.91 | 6.13 | 5.76 | 5.87 | 5.94 | 5.66 | 5.89 | 4.59 | 4.92 | 4.17 | 4.67 | 6.29 | 6.43 |
| 79 | 6.58 | 6.28 | 7.72 | 7.69 | 7.86 | 8 | 8.31 | 7.97 | 7.58 | 7.9 | 6.43 | 6.17 | 5.43 | 5.91 | 7.91 | 7.95 |
| 80 | 6.9 | 6.64 | 8.05 | 7.86 | 8.29 | 8.56 | 7.61 | 8.24 | 7.95 | 7.9 | 6.74 | 6.35 | 6.2 | 5.95 | 8.35 | 8.6 |

Abbreviation: SUV_mean_: mean standardized uptake value; AAL: automated anatomical labelling.

Supplementary Table 10 The SUV_max_ of normal 45-70 years old group according to AAL standard.

| Region of Interest | Amygdala | Amygdala | Angular gyrus | Angular gyrus | Anterior cingulate and paracingulate gyri | Anterior cingulate and paracingulate gyri | Basal ganglia | Basal ganglia | Calcarine fissure and surrounding cortex | Calcarine fissure and surrounding cortex | Caudate nucleus | Caudate nucleus | Central region | Central region | Cingulate and paracingulate gyri | Cingulate and paracingulate gyri | Cuneus | Cuneus |
| --- | --- | --- | --- | --- | --- | --- | --- | --- | --- | --- | --- | --- | --- | --- | --- | --- | --- | --- |
| Side | L | R | L | R | L | R | L | R | L | R | L | R | L | R | L | R | L | R |
| Number 1 | 4.73 | 4.64 | 9.01 | 9.66 | 7.98 | 8.29 | 10 | 11 | 11.38 | 11.2 | 9.77 | 11 | 10.76 | 9.89 | 10.46 | 11.12 | 11.07 | 9.64 |
| 2 | 8.08 | 6.54 | 11.67 | 12.24 | 11.01 | 11.35 | 12.97 | 12.57 | 17.38 | 16.6 | 12.23 | 11.92 | 14.49 | 13.45 | 14.48 | 12.87 | 13.77 | 16.2 |
| 3 | 7.76 | 6.68 | 14.76 | 14.13 | 12.73 | 11.41 | 14.57 | 14.49 | 19.38 | 18.64 | 14.39 | 14.49 | 16.63 | 17.25 | 14.64 | 14.89 | 19.81 | 17.85 |
| 4 | 9.42 | 7.95 | 16.48 | 14.37 | 12.55 | 13.47 | 17.83 | 18.16 | 20.09 | 18.74 | 15.28 | 15.46 | 19.17 | 18.26 | 16.68 | 17.93 | 17.78 | 19.71 |
| 5 | 10.59 | 9.18 | 19.52 | 19.61 | 15.68 | 18.4 | 19.55 | 20.03 | 20.88 | 20.45 | 18.37 | 19.09 | 21.67 | 19.72 | 22.95 | 21.28 | 25.69 | 20.55 |
| 6 | 7.25 | 6.83 | 11.18 | 11.63 | 11.14 | 12.25 | 12.88 | 13.19 | 12.8 | 12.44 | 12.44 | 13.19 | 15.1 | 13.45 | 14.5 | 14.28 | 14.9 | 12.76 |
| 7 | 9.42 | 8.12 | 16.42 | 18.42 | 15.07 | 13.52 | 18.76 | 19.25 | 20.1 | 19.2 | 17.53 | 16.22 | 19.77 | 18.37 | 18.76 | 19.56 | 19.22 | 19.33 |
| 8 | 6.39 | 5.57 | 11.38 | 10.46 | 10.34 | 9.41 | 13.16 | 12.86 | 11.39 | 12.4 | 13.16 | 11.39 | 14.39 | 12.84 | 12.24 | 13.6 | 11.78 | 11.57 |
| 9 | 8.59 | 7.89 | 18.13 | 16.75 | 15.72 | 14.98 | 17.19 | 17.41 | 19.82 | 19.44 | 16.74 | 16.43 | 17.93 | 17.73 | 17.84 | 17.64 | 17.49 | 19.6 |
| 10 | 7.11 | 7.08 | 12.27 | 12.44 | 10.49 | 11.35 | 12.89 | 12.85 | 13.99 | 15.68 | 11.98 | 12.66 | 12.54 | 12.8 | 12.52 | 13.52 | 12.71 | 14.78 |
| 11 | 12.24 | 13.06 | 20.79 | 25.47 | 18.15 | 19.45 | 25.51 | 23.45 | 27.68 | 29.98 | 25.51 | 23.03 | 22.83 | 24.88 | 23.82 | 27.57 | 25.55 | 28.93 |
| 12 | 7.83 | 8.07 | 15.89 | 16.16 | 14.45 | 13.77 | 18.1 | 16.83 | 18.69 | 19.64 | 16.42 | 16.83 | 17.03 | 18.21 | 21.09 | 21.36 | 15.39 | 18.25 |
| 13 | 9.47 | 7.43 | 19.71 | 19.07 | 14.34 | 16.46 | 17.29 | 17.72 | 21.59 | 22.76 | 15.88 | 17.14 | 20.63 | 19.82 | 19.5 | 19.12 | 19.01 | 22.28 |
| 14 | 8.53 | 9.08 | 17 | 16.4 | 13.44 | 17.19 | 18.71 | 18.74 | 20.08 | 19.39 | 17.34 | 17.51 | 19.73 | 20.85 | 18.58 | 21.13 | 21.94 | 18.84 |
| 15 | 4.62 | 4.55 | 7.42 | 7.51 | 6.6 | 7 | 8.68 | 8.97 | 9.67 | 9.36 | 7.71 | 7.94 | 8.89 | 8.96 | 8.1 | 8.28 | 8.72 | 8.65 |
| 16 | 5.16 | 4.48 | 9.28 | 8.83 | 8 | 7.31 | 9.33 | 9.05 | 9.21 | 9.3 | 8.87 | 7.45 | 10.17 | 9.09 | 9.23 | 8.81 | 9.7 | 10.24 |
| 17 | 8.14 | 6.91 | 16.73 | 17.89 | 12.68 | 14.54 | 15.92 | 14.91 | 16.54 | 18.5 | 15.1 | 14.78 | 19.76 | 16.83 | 16.61 | 17.82 | 16.31 | 17.07 |
| 18 | 7.85 | 7.66 | 19.54 | 17.81 | 14.45 | 16.3 | 21.51 | 20.36 | 19.11 | 20.86 | 20.76 | 19.8 | 18.78 | 19.46 | 19.06 | 19.68 | 20.98 | 20.54 |
| 19 | 9.63 | 8.82 | 20.53 | 22.1 | 18.02 | 18.08 | 22.63 | 21.92 | 28.05 | 26.82 | 22.25 | 20.15 | 22.14 | 20.65 | 25.39 | 21.46 | 21.87 | 25.32 |
| 20 | 6.96 | 6.7 | 12.35 | 13.05 | 11.27 | 12.27 | 15.96 | 16.31 | 20.83 | 19.27 | 13.88 | 13.79 | 15.96 | 15.67 | 14.13 | 15.14 | 14.68 | 15.66 |
| 21 | 10.73 | 11.21 | 25.86 | 21.3 | 18.72 | 16.67 | 22 | 22.45 | 24.38 | 25.33 | 20.62 | 19.72 | 23.08 | 24.34 | 24.23 | 24.14 | 20.77 | 25.67 |
| 22 | 8.49 | 7.72 | 18.17 | 18.07 | 14.96 | 15.75 | 17.98 | 16.43 | 18.27 | 22.21 | 17.98 | 16.38 | 20.47 | 18.75 | 19.42 | 21.25 | 18.77 | 19.43 |
| 23 | 6.8 | 6.55 | 12.2 | 11.91 | 11.34 | 11.09 | 13.13 | 13.14 | 15.31 | 14.87 | 12.45 | 12.23 | 13.24 | 13.38 | 13.83 | 14.8 | 16.13 | 13.68 |
| 24 | 12.67 | 10.69 | 20.23 | 21.65 | 20.41 | 23.88 | 21.94 | 26.55 | 24.11 | 24.84 | 21.43 | 26.55 | 28.63 | 27.63 | 29.67 | 25.72 | 21.93 | 22.64 |
| 25 | 7.92 | 7.44 | 12.45 | 11.91 | 11.52 | 11.33 | 13.99 | 13.92 | 11.68 | 12.63 | 13.01 | 12.17 | 14.69 | 13.57 | 12.95 | 14 | 12.36 | 13.02 |
| 26 | 7.69 | 8.47 | 15.58 | 15.75 | 12.15 | 13.96 | 17.6 | 14.74 | 16.11 | 16.05 | 17.6 | 14.74 | 17.66 | 16.16 | 17.34 | 15.49 | 13.6 | 14.14 |
| 27 | 6.23 | 7.11 | 11.79 | 12.44 | 10.28 | 9.9 | 14.12 | 13.45 | 14.71 | 16.71 | 14.12 | 13.45 | 14.08 | 11.81 | 12 | 15.89 | 12.53 | 13.28 |
| 28 | 5.63 | 6.23 | 9.82 | 12.15 | 8.52 | 8.78 | 11.99 | 10.89 | 14.22 | 13.57 | 10.53 | 10.31 | 10.69 | 12.24 | 10.34 | 10.03 | 11 | 11.74 |
| 29 | 8.61 | 7.41 | 15.95 | 15.16 | 12.25 | 14.29 | 15.66 | 15.42 | 20.5 | 20.55 | 14.93 | 13.7 | 17.03 | 16.32 | 18.07 | 16.81 | 17.18 | 17.83 |
| 30 | 6.66 | 6.14 | 11.44 | 11.76 | 10.49 | 10.43 | 12.91 | 12.75 | 15.01 | 13.06 | 12.01 | 12.57 | 12.75 | 12.31 | 14.21 | 13.39 | 13.26 | 12.95 |
| 31 | 7.16 | 6.19 | 11.92 | 11.56 | 10.13 | 10.75 | 12.8 | 12.01 | 17.67 | 16.21 | 12.8 | 11.75 | 12.84 | 13.72 | 14.83 | 15.47 | 13.69 | 13.06 |
| 32 | 8.13 | 7.73 | 16.55 | 17.35 | 14.41 | 13.93 | 16.6 | 15.43 | 20.54 | 22.42 | 16.6 | 15.28 | 16.83 | 15.79 | 15.63 | 18.28 | 16.69 | 17.95 |
| 33 | 4.13 | 4.34 | 6.04 | 6.08 | 5.45 | 5.23 | 7.42 | 7.39 | 8.47 | 8.79 | 6.69 | 5.86 | 7.42 | 7.19 | 7.13 | 7.57 | 7.79 | 9.24 |
| 34 | 7 | 7.16 | 15.01 | 15.85 | 12.72 | 12.08 | 14.18 | 15.21 | 18.12 | 19.31 | 14.18 | 15 | 17.27 | 15.43 | 14.74 | 16.04 | 19.87 | 17.68 |
| 35 | 6.77 | 6.99 | 13.16 | 14.07 | 11.63 | 11.42 | 14.62 | 12.96 | 15.82 | 14.86 | 13.45 | 11.82 | 16.84 | 14.14 | 13.84 | 14.58 | 14.41 | 16.14 |
| 36 | 6.44 | 6.95 | 14.72 | 14.46 | 10.94 | 11.25 | 13.16 | 13.31 | 13.11 | 14.07 | 12.65 | 12.17 | 15.35 | 13.95 | 13.91 | 15.2 | 14.93 | 17.18 |
| 37 | 7.29 | 6.05 | 15.51 | 14.98 | 11.59 | 11.05 | 14.27 | 14.24 | 17.58 | 18.65 | 12.76 | 14.24 | 16.19 | 14.5 | 14.09 | 14.6 | 16.68 | 17.69 |
| 38 | 9.79 | 9.17 | 20.46 | 22.08 | 17.59 | 16.6 | 21.11 | 21.15 | 26.27 | 26.95 | 18.25 | 18.04 | 22.59 | 21.42 | 21.94 | 24.14 | 23.56 | 25.76 |
| 39 | 6.55 | 5.97 | 11.8 | 12.16 | 10.95 | 11.35 | 12.96 | 12.54 | 14 | 16.22 | 11.94 | 11.33 | 13.66 | 13.89 | 12.83 | 13.31 | 13.06 | 12.26 |
| 40 | 8.39 | 7.71 | 16.76 | 16.64 | 14.06 | 16.82 | 16.96 | 17.57 | 16.57 | 16.44 | 16.87 | 16.98 | 16.67 | 16.8 | 17.23 | 19.41 | 20.21 | 19.38 |
| 41 | 8.6 | 8.63 | 20.34 | 17.07 | 14.02 | 14.51 | 16.31 | 15.28 | 19.86 | 19.79 | 16.31 | 15.15 | 18.87 | 19.64 | 20.18 | 18.6 | 19.02 | 17.55 |
| 42 | 9.83 | 8.87 | 20.34 | 19.43 | 15.59 | 15.93 | 17.68 | 18.21 | 21.61 | 20.97 | 17.68 | 18.21 | 20.41 | 19.14 | 25.78 | 23.61 | 23.49 | 22.27 |
| 43 | 6.03 | 6.69 | 11.03 | 11.06 | 10.13 | 10.87 | 11.62 | 11.7 | 14.87 | 14.89 | 10.83 | 11.7 | 13.16 | 12.54 | 13.84 | 13.08 | 12.19 | 11.84 |
| 44 | 5.6 | 5.15 | 9.82 | 10.26 | 9.99 | 8.41 | 11.87 | 10.62 | 11.29 | 13.15 | 10.13 | 10.46 | 12.34 | 11.56 | 11.43 | 11.75 | 11.89 | 11.12 |
| 45 | 4.54 | 4.08 | 6.75 | 7.28 | 6.44 | 6.73 | 7.92 | 7.81 | 7.23 | 8.03 | 7.23 | 7.31 | 7.41 | 7.86 | 8.07 | 7.74 | 7.74 | 8.09 |
| 46 | 7.17 | 7.81 | 13.25 | 14.34 | 11.11 | 12.43 | 16.3 | 15.37 | 15.54 | 16.28 | 16.13 | 15.37 | 16.28 | 14.95 | 14.14 | 15.31 | 16.28 | 16.83 |
| 47 | 7.79 | 7.29 | 15.35 | 16.2 | 13.4 | 13.62 | 15.07 | 15.78 | 17.69 | 17.4 | 14.15 | 13.96 | 15.14 | 14.89 | 16.28 | 17.9 | 15.88 | 17.15 |
| 48 | 10.09 | 10.64 | 18.13 | 20.52 | 16.08 | 17.83 | 22.34 | 20.15 | 21.47 | 25.65 | 22.11 | 19.43 | 22.59 | 22.27 | 22.36 | 22.18 | 21.28 | 21.04 |
| 49 | 5.26 | 4.79 | 9.02 | 10.11 | 9.24 | 9.03 | 9.95 | 9.04 | 11.26 | 12.34 | 9.68 | 9.04 | 10.56 | 10.44 | 9.83 | 10.68 | 10.61 | 10.8 |
| 50 | 9.2 | 8.43 | 20.35 | 21.69 | 17.75 | 17.51 | 23.06 | 23.37 | 27.93 | 27.82 | 21.92 | 22.58 | 22.05 | 21.34 | 21.46 | 20.94 | 23.95 | 25.54 |
| 51 | 6 | 5.21 | 10.13 | 11.52 | 8.01 | 8.89 | 12.93 | 13.96 | 11.26 | 12.39 | 10.44 | 10.6 | 11.93 | 11.36 | 11.23 | 11.71 | 10.67 | 11 |
| 52 | 9.18 | 6.79 | 14.27 | 15.17 | 13.13 | 13.03 | 17.72 | 17.88 | 19.23 | 20.52 | 17.09 | 17.88 | 16.44 | 16.63 | 16.26 | 17.35 | 15.34 | 17.57 |
| 53 | 8.15 | 8.81 | 22.06 | 21.7 | 16.88 | 17.73 | 20.45 | 21.5 | 19.76 | 19.97 | 22.65 | 20.48 | 21.96 | 22.53 | 20.25 | 20.25 | 19.85 | 23.13 |
| 54 | 7.68 | 7.71 | 14.15 | 15.46 | 13.05 | 13.99 | 15.99 | 15.55 | 17.14 | 17.83 | 15.97 | 15.55 | 16.36 | 14.86 | 17.42 | 18.27 | 16.09 | 16.04 |
| 55 | 5.66 | 5.33 | 10.16 | 9.4 | 8 | 8.37 | 10.63 | 10.04 | 11.36 | 11.64 | 9.48 | 10.04 | 10.97 | 10.43 | 10.34 | 10.63 | 13.05 | 10.92 |
| 56 | 4.5 | 4.6 | 7.19 | 7.56 | 8.21 | 7.78 | 8.82 | 8.72 | 8.53 | 9.08 | 8.39 | 8.28 | 9.65 | 8.46 | 8.95 | 9.2 | 9.23 | 8.57 |
| 57 | 5.44 | 5.3 | 9.33 | 9.46 | 8.31 | 8.5 | 10.42 | 10.85 | 11.34 | 10.97 | 9.45 | 10.85 | 12.08 | 10.27 | 9.64 | 9.83 | 9.64 | 9.64 |
| 58 | 5.12 | 4.48 | 7.86 | 8.82 | 8.02 | 8.18 | 9.67 | 8.88 | 9.56 | 9.82 | 8.46 | 8.88 | 8.81 | 9.68 | 8.84 | 9.03 | 8.84 | 9.45 |
| 59 | 7.07 | 6.65 | 14.56 | 13.97 | 11.79 | 11.77 | 15.91 | 16.84 | 17.81 | 19.13 | 13.96 | 13.58 | 14.64 | 13.77 | 15.04 | 15.39 | 16.38 | 16.54 |
| 60 | 5.9 | 7.56 | 10.87 | 12.64 | 12.13 | 11.52 | 13.04 | 12.97 | 13.87 | 13.85 | 11.75 | 11.84 | 14.5 | 12.13 | 14.04 | 15.27 | 12.1 | 14.19 |
| 61 | 4.41 | 5.42 | 8.27 | 8.48 | 7.79 | 7.92 | 10.51 | 10 | 8.73 | 8.15 | 8.9 | 10 | 9.1 | 9.09 | 9.41 | 10.02 | 8.74 | 8.25 |
| 62 | 7.37 | 8.08 | 12.68 | 13.89 | 11.09 | 11.34 | 14.79 | 13.71 | 14.16 | 14.1 | 12.14 | 11.64 | 13.86 | 15.14 | 14.78 | 15.32 | 13.25 | 15.38 |
| 63 | 10.32 | 11.54 | 20.28 | 22.12 | 16.56 | 17.56 | 19.65 | 19.51 | 23.82 | 27.06 | 19.65 | 19.06 | 22.91 | 23.58 | 20.01 | 23.13 | 24.04 | 25.64 |
| 64 | 4.05 | 3.77 | 7.71 | 7.94 | 5.91 | 6.1 | 7.78 | 8.41 | 9.18 | 8.69 | 6.92 | 7.2 | 8.02 | 7.91 | 8.14 | 7.68 | 8.18 | 8.24 |
| 65 | 9.62 | 10.1 | 17.19 | 18.56 | 17.68 | 16.49 | 20.45 | 19.63 | 23.33 | 21.47 | 18.63 | 17.7 | 21.15 | 20.34 | 20 | 20.72 | 22.13 | 21.68 |
| 66 | 10.04 | 8.42 | 23.27 | 20.43 | 17.39 | 15.89 | 18.57 | 17.77 | 25.88 | 25.17 | 18.57 | 17.77 | 21.41 | 21.26 | 20.05 | 21.01 | 23.29 | 22.19 |
| 67 | 9.13 | 8.41 | 16.07 | 16.57 | 14.09 | 14.88 | 18.51 | 17.92 | 23.89 | 23.37 | 18.51 | 17.92 | 19.54 | 20.34 | 18.07 | 17.61 | 22.87 | 19.73 |
| 68 | 6.81 | 7.44 | 10.71 | 12.67 | 9.89 | 11.15 | 12.37 | 12.52 | 15.21 | 14.61 | 11.71 | 12.23 | 13.24 | 13.96 | 12.98 | 13.93 | 12.93 | 13.86 |
| 69 | 6.4 | 5.81 | 12.47 | 12.98 | 11.53 | 12.44 | 14.36 | 13.51 | 15.52 | 17.89 | 11.88 | 13.51 | 15 | 14.72 | 14.38 | 14.3 | 16 | 16.18 |
| 70 | 7.24 | 6.74 | 12.58 | 13.76 | 12.34 | 11.17 | 14.29 | 14.6 | 14.3 | 15.9 | 14.29 | 13.53 | 14.47 | 13.23 | 14.17 | 14.96 | 13.44 | 16.41 |
| 71 | 7.76 | 7.03 | 15.4 | 15.49 | 12.44 | 13.29 | 14.52 | 14.22 | 15.46 | 15.99 | 13.94 | 14.22 | 15.52 | 14.26 | 16.18 | 16.06 | 16.06 | 18.8 |
| 72 | 9.8 | 8.11 | 14.65 | 18.02 | 13.42 | 15.25 | 18.35 | 18.21 | 22.44 | 22.67 | 17.14 | 15.91 | 25.86 | 20.08 | 19.92 | 20.32 | 18.48 | 18.44 |
| 73 | 6.93 | 5.91 | 12.45 | 12.36 | 10.32 | 10.19 | 13.19 | 13.52 | 13.76 | 14.45 | 12.28 | 12.46 | 12.95 | 12.56 | 13.22 | 12.6 | 11.76 | 13.05 |
| 74 | 6.4 | 5.7 | 13.86 | 13.94 | 11.73 | 10.87 | 12.62 | 12.36 | 14.32 | 15.83 | 12.35 | 12.36 | 13.61 | 13.09 | 14.25 | 13.55 | 14.27 | 14.74 |
| 75 | 4.8 | 4.07 | 6.97 | 8.69 | 6.56 | 7.1 | 8.7 | 8.04 | 9.17 | 9.62 | 8.7 | 7.7 | 8.83 | 7.88 | 8.13 | 8.52 | 7.77 | 8.78 |
| 76 | 5.68 | 4.9 | 9.98 | 9.71 | 8.72 | 8.72 | 10.43 | 10.56 | 10.73 | 10.57 | 9.93 | 10.42 | 10.31 | 10.42 | 10.65 | 11.1 | 11.87 | 11.43 |
| 77 | 9.08 | 8.38 | 22.1 | 19.08 | 17.79 | 17 | 19.66 | 21 | 20.07 | 20.74 | 19.46 | 21 | 19.88 | 20.47 | 23.23 | 21.12 | 22.01 | 20.66 |
| 78 | 5.47 | 5.67 | 11.64 | 11.4 | 10.52 | 9.54 | 13.33 | 11.85 | 13.53 | 13.39 | 13.33 | 11.63 | 11.56 | 12.91 | 12.34 | 12.51 | 12.86 | 13.78 |
| 79 | 7.46 | 7.31 | 15.59 | 13.84 | 13.46 | 13.47 | 16 | 16.74 | 17.99 | 17.9 | 15.17 | 15.4 | 15.12 | 15.11 | 15.13 | 16.17 | 17.23 | 16.11 |
| 80 | 7.25 | 7.48 | 14.64 | 14.98 | 12.06 | 13.6 | 14.15 | 14.98 | 15.94 | 17.75 | 13.8 | 14.98 | 16.87 | 14.54 | 15.3 | 15.82 | 15.54 | 15.25 |

| Region of Interest | Frontal lobe | Frontal lobe | Fusiform gyrus | Fusiform gyrus | Gyrus rectus | Gyrus rectus | Heschl gyrus | Heschl gyrus | Hippocampus | Hippocampus | Inferior frontal gyrus, opercular part | Inferior frontal gyrus, opercular part | Inferior frontal gyrus, orbital part | Inferior frontal gyrus, orbital part | Inferior frontal gyrus, triangular part | Inferior frontal gyrus, triangular part | Inferior occipital gyrus | Inferior occipital gyrus |
| --- | --- | --- | --- | --- | --- | --- | --- | --- | --- | --- | --- | --- | --- | --- | --- | --- | --- | --- |
| Side | L | R | L | R | L | R | L | R | L | R | L | R | L | R | L | R | L | R |
| Number 1 | 10.09 | 10.17 | 8.27 | 8.22 | 8.28 | 7.91 | 8.26 | 9.14 | 6.41 | 6.47 | 9.24 | 9.48 | 8.54 | 9.13 | 9.58 | 9.51 | 9 | 10.01 |
| 2 | 15.1 | 14.37 | 11.51 | 12.03 | 11.9 | 11.85 | 11.94 | 12.31 | 8.78 | 7.92 | 14.34 | 13.38 | 12.32 | 11.71 | 13.43 | 13.08 | 16.52 | 14.48 |
| 3 | 16.99 | 15.82 | 13.42 | 13.8 | 11.78 | 10.75 | 13.43 | 12.95 | 8.94 | 8.42 | 14.03 | 12.7 | 12.65 | 11.96 | 14.32 | 13.8 | 19.77 | 18.67 |
| 4 | 20.61 | 18.58 | 14.16 | 16.22 | 13.34 | 13.11 | 17.92 | 19.49 | 9.8 | 11.11 | 13 | 17.86 | 13.64 | 14.88 | 16.08 | 16.03 | 19.62 | 17.34 |
| 5 | 22.33 | 22.42 | 17.18 | 17.09 | 15.71 | 17.54 | 18.07 | 16.75 | 15.1 | 10.61 | 19.91 | 18.58 | 20.55 | 19.35 | 21.42 | 21.2 | 20.58 | 21.43 |
| 6 | 16.26 | 15.06 | 10.28 | 10.82 | 11.16 | 10.62 | 12.28 | 12.04 | 9.35 | 8.6 | 14.31 | 15.06 | 11.92 | 12.57 | 13.92 | 13.07 | 11.61 | 11.03 |
| 7 | 20.31 | 18.59 | 18.06 | 18.42 | 16.2 | 17.05 | 16.33 | 17.29 | 10.74 | 12.45 | 18.23 | 17.61 | 20.31 | 18.59 | 18.57 | 17.44 | 20.15 | 22.91 |
| 8 | 14.15 | 13.15 | 9.98 | 10.28 | 9.36 | 9.13 | 9.84 | 10.12 | 7.08 | 6.32 | 10.74 | 12.76 | 12.26 | 11.07 | 11.95 | 12.28 | 11.05 | 12.52 |
| 9 | 20.19 | 20.99 | 13.75 | 14.44 | 14.86 | 15.39 | 19.52 | 19.84 | 10.98 | 10.77 | 19.65 | 20.99 | 19.31 | 18.7 | 18.78 | 18.85 | 14.83 | 15.81 |
| 10 | 14.76 | 14.69 | 12.57 | 11.03 | 11.19 | 11.27 | 13.35 | 13.28 | 8.87 | 8.17 | 14.37 | 13.57 | 12.26 | 13.88 | 13.49 | 12.62 | 14.36 | 14.15 |
| 11 | 25.61 | 25.66 | 23.14 | 23.1 | 19.71 | 20.9 | 21.89 | 22.96 | 13.08 | 13.78 | 22.23 | 25.36 | 21.17 | 21.82 | 22.35 | 25.66 | 26.01 | 30.34 |
| 12 | 18.99 | 19.74 | 14.45 | 15.43 | 13.45 | 15.51 | 16.56 | 15 | 10.58 | 10.63 | 17.76 | 17.19 | 16.85 | 17.21 | 18.03 | 19.74 | 16.83 | 16.38 |
| 13 | 20.41 | 27.33 | 18.33 | 20.26 | 15.77 | 16.14 | 16.68 | 19.12 | 10.67 | 11.13 | 18.96 | 27.33 | 17.35 | 18.1 | 20.4 | 24.2 | 20.39 | 20.97 |
| 14 | 19.09 | 19.23 | 15.23 | 16.13 | 13.42 | 12.91 | 19.61 | 18.04 | 10.66 | 10.21 | 16.87 | 18.36 | 16.86 | 15.67 | 18.53 | 17.93 | 19.41 | 18.72 |
| 15 | 8.74 | 8.71 | 7.13 | 7.65 | 5.99 | 6.68 | 6.74 | 6.06 | 5.19 | 5.07 | 8.17 | 7.5 | 8.52 | 7.87 | 8.37 | 8.71 | 9.57 | 9.13 |
| 16 | 10.95 | 10.98 | 7.38 | 8.31 | 7.83 | 7.9 | 8.28 | 7.8 | 5.84 | 6.15 | 8.76 | 9.57 | 8.57 | 8.3 | 8.98 | 8.99 | 7.48 | 7.99 |
| 17 | 19.11 | 18.63 | 13.84 | 13.67 | 14.91 | 13.42 | 13.38 | 16.49 | 11.79 | 10.04 | 17.3 | 18.47 | 14.23 | 15.12 | 19.11 | 16.45 | 15.35 | 15.44 |
| 18 | 20.48 | 21.24 | 15.72 | 15.1 | 14.53 | 15.75 | 18.22 | 19.81 | 10.37 | 10.32 | 18.57 | 21.24 | 19.13 | 17.32 | 20.48 | 20.29 | 20.48 | 17.96 |
| 19 | 24.38 | 25.67 | 21.04 | 22.41 | 19.11 | 17.21 | 22.2 | 21.96 | 12.08 | 11.88 | 19.2 | 24.25 | 20.27 | 22.47 | 23.69 | 24.27 | 23.9 | 25.78 |
| 20 | 16.3 | 15.13 | 14.74 | 14.67 | 11.14 | 11.94 | 15.96 | 14.79 | 8.36 | 7.89 | 15.6 | 15.13 | 14.9 | 13.45 | 13.85 | 14.58 | 16.18 | 19.7 |
| 21 | 24.41 | 24.25 | 17.43 | 18.64 | 15.87 | 16.16 | 20.51 | 17.14 | 12.54 | 12.13 | 19.78 | 21.72 | 19.16 | 19.87 | 21.27 | 20.28 | 23.83 | 19.8 |
| 22 | 20.97 | 19.65 | 14.91 | 15.48 | 16.51 | 14.77 | 16.95 | 16.77 | 11.7 | 11.66 | 18.28 | 16.19 | 17.43 | 15.01 | 17.94 | 18.95 | 18.89 | 17.35 |
| 23 | 13.36 | 14.26 | 11.93 | 11.24 | 11.11 | 10.02 | 12.12 | 10.91 | 8.87 | 8.71 | 12.7 | 12.5 | 11.84 | 12.08 | 11.99 | 12.66 | 14.19 | 14.76 |
| 24 | 33.71 | 28.44 | 23.72 | 22.55 | 15.58 | 20.24 | 17.81 | 18.24 | 16.44 | 13.59 | 30.03 | 23.4 | 20.3 | 21.1 | 33.71 | 20.7 | 23.66 | 18.72 |
| 25 | 15.65 | 15.17 | 9.43 | 9.9 | 12.68 | 10.27 | 13.44 | 12.24 | 7.79 | 8.5 | 12.71 | 11.95 | 13.24 | 12.96 | 14 | 12.59 | 9.91 | 8.8 |
| 26 | 18.68 | 17.44 | 15.28 | 15.6 | 13.09 | 11.89 | 17.83 | 16.33 | 9.88 | 9.91 | 16.96 | 16.23 | 15.38 | 14.58 | 18.52 | 16.1 | 18.03 | 15.84 |
| 27 | 13.83 | 12.09 | 12.37 | 12.15 | 10.11 | 9.87 | 12.46 | 12.74 | 9.16 | 8.4 | 13.71 | 12.09 | 12.03 | 11.32 | 13.63 | 11.63 | 14.32 | 16.05 |
| 28 | 12.02 | 11.26 | 9.79 | 10.8 | 9.16 | 8.33 | 8.64 | 10.59 | 6.44 | 6.42 | 10.52 | 11.26 | 9.3 | 10.7 | 12.02 | 10.69 | 10.76 | 11.8 |
| 29 | 17.69 | 17.91 | 15.65 | 16.55 | 12.53 | 13.19 | 13.66 | 16.18 | 10.27 | 10.28 | 17.25 | 16.29 | 14.46 | 14.15 | 16.9 | 17.91 | 16.26 | 19.11 |
| 30 | 13.68 | 13.44 | 10.46 | 10.4 | 10.29 | 9.98 | 12.21 | 11.48 | 7.14 | 7.38 | 13.15 | 11.68 | 12.22 | 11.45 | 13.5 | 13.24 | 11.43 | 10.2 |
| 31 | 14.71 | 13.75 | 10.68 | 12.01 | 10.5 | 10.48 | 9.97 | 11.9 | 7.21 | 7.26 | 12.1 | 12.64 | 10.83 | 10.61 | 11.93 | 12.43 | 13.86 | 15.47 |
| 32 | 18.5 | 18.16 | 15.68 | 16 | 14.27 | 12.55 | 16.76 | 14.78 | 10.5 | 9.95 | 17.98 | 17.65 | 16.6 | 17.2 | 18.03 | 17.43 | 17.94 | 19.08 |
| 33 | 7.96 | 7.88 | 6.49 | 6.93 | 6.15 | 5.17 | 6.49 | 7.21 | 4.8 | 4.67 | 6.19 | 6.17 | 5.93 | 6.29 | 6.24 | 6.48 | 7.41 | 7.78 |
| 34 | 16.46 | 15.14 | 16.01 | 13.25 | 11.73 | 11.71 | 13.57 | 13.83 | 10.08 | 9.42 | 14.34 | 13.44 | 15.69 | 15.14 | 15.17 | 15 | 16.62 | 15.2 |
| 35 | 16.18 | 15.47 | 11.62 | 12.67 | 12.66 | 11.03 | 13.11 | 12.65 | 8.3 | 7.44 | 15.11 | 14.16 | 13.36 | 12.79 | 14.95 | 13.44 | 15.78 | 14.29 |
| 36 | 14.82 | 15.53 | 12.39 | 13.59 | 11.98 | 12.88 | 12.22 | 12.65 | 8.45 | 8.31 | 14.81 | 14.8 | 13.3 | 12.25 | 13.34 | 14.86 | 13.25 | 15.51 |
| 37 | 16.31 | 15.36 | 12.68 | 12.78 | 13.11 | 11.34 | 12.7 | 12.7 | 9.1 | 8.43 | 14.13 | 13.87 | 12.1 | 12.82 | 15.04 | 14.25 | 17.68 | 16.21 |
| 38 | 24.96 | 22.93 | 18.2 | 19.14 | 16.13 | 14.95 | 19.33 | 19.3 | 14.86 | 13.57 | 22.46 | 19.8 | 21.27 | 19.03 | 24.96 | 21.2 | 23.71 | 26.58 |
| 39 | 13.33 | 14.32 | 11.39 | 11.27 | 11.03 | 10.3 | 11.48 | 11.58 | 8.06 | 7.58 | 12.23 | 12.41 | 12.18 | 12.37 | 12.83 | 12.19 | 12.87 | 12.74 |
| 40 | 20.59 | 18.59 | 13.07 | 12.92 | 13.83 | 14.26 | 15.36 | 15.54 | 12.4 | 10.51 | 15.5 | 18.11 | 16.2 | 16.1 | 16.07 | 17.88 | 14.38 | 13.88 |
| 41 | 19.47 | 19.96 | 14.91 | 17.42 | 14.32 | 13.88 | 16.57 | 15.97 | 10.06 | 9.38 | 16.43 | 18.69 | 15.88 | 16.89 | 17.43 | 19.02 | 16.73 | 18.36 |
| 42 | 22.1 | 20.36 | 16.44 | 15.84 | 15.7 | 16 | 19.28 | 15.56 | 11.68 | 11.63 | 17.95 | 17.53 | 18.56 | 18.42 | 19.02 | 20.24 | 21.88 | 18.34 |
| 43 | 13.34 | 11.78 | 10.8 | 10.42 | 9.64 | 9.5 | 9.76 | 9.56 | 7.38 | 7.99 | 10.7 | 10.94 | 10.71 | 10.97 | 11.65 | 11.56 | 13.58 | 10.88 |
| 44 | 11.98 | 12.26 | 9.81 | 10.29 | 8.7 | 8.66 | 10.17 | 10.72 | 6.87 | 7.04 | 10.25 | 11.25 | 9.92 | 10.6 | 10.82 | 10.66 | 10.48 | 12.57 |
| 45 | 7.78 | 7.75 | 6.62 | 6.12 | 6.26 | 6.39 | 7.05 | 7.05 | 5.1 | 5.12 | 6.86 | 7.4 | 7.1 | 7.12 | 6.95 | 6.92 | 6.37 | 7.19 |
| 46 | 15.12 | 15.39 | 13.15 | 13.6 | 11.77 | 10.82 | 13.6 | 12.3 | 8.04 | 8.37 | 14.57 | 14.44 | 12.84 | 13.67 | 13.33 | 13.45 | 15.45 | 15.9 |
| 47 | 16.51 | 16.83 | 12.7 | 13.2 | 13.49 | 12.57 | 12.17 | 11.83 | 9.17 | 8.25 | 14.73 | 14.8 | 15.26 | 15.1 | 15.22 | 16.07 | 17.26 | 15.66 |
| 48 | 24.8 | 24.31 | 19 | 17.25 | 15.5 | 19.01 | 20.66 | 19.03 | 13.09 | 12.61 | 18.51 | 20.34 | 19.79 | 22.02 | 19.67 | 21.79 | 21.26 | 22.1 |
| 49 | 10.75 | 10.59 | 8.67 | 8.29 | 7.59 | 8.04 | 10.42 | 8.94 | 6.27 | 5.92 | 10.29 | 10.59 | 10.75 | 9.78 | 9.43 | 10.19 | 11.19 | 11.62 |
| 50 | 23.39 | 23.84 | 20.19 | 18.61 | 18.37 | 17.89 | 19.09 | 20 | 12.47 | 11.25 | 22.15 | 23.04 | 19.71 | 20.25 | 22.35 | 23.84 | 22.72 | 25.32 |
| 51 | 11.74 | 12.18 | 8.86 | 9.59 | 9.97 | 10.12 | 10.87 | 10.91 | 6.44 | 6.38 | 11.15 | 10.61 | 9.98 | 9.64 | 10.27 | 10.35 | 10.82 | 10.61 |
| 52 | 18.32 | 18.43 | 14.62 | 14.55 | 13.14 | 12.86 | 15.49 | 14.13 | 9.1 | 9.48 | 15.84 | 17.62 | 17.97 | 16.2 | 14.52 | 15.27 | 18.62 | 20.89 |
| 53 | 24.71 | 17.91 | 17.06 | 17.04 | 16.56 | 16.88 | 16.23 | 11.46 | 11.89 | 20.21 | 18.68 | 19.13 | 22.1 | 20.62 | 20.75 | 19 | 19.72 | 20.5 |
| 54 | 16.25 | 16.09 | 13.97 | 16.62 | 13.25 | 13.04 | 14.1 | 13.47 | 8.92 | 9.17 | 14.18 | 14.59 | 13.71 | 14.18 | 14.68 | 16.09 | 16.31 | 16.69 |
| 55 | 10.56 | 11.18 | 9.02 | 9.92 | 7.98 | 7.77 | 7.83 | 8.54 | 6.73 | 6.65 | 9.28 | 9.34 | 9.7 | 9.67 | 10.56 | 9.73 | 10.27 | 10.57 |
| 56 | 10.13 | 9.83 | 7.46 | 7.26 | 7.86 | 6.9 | 6.95 | 6.91 | 5.77 | 5.34 | 8.22 | 9.83 | 8.25 | 9.55 | 7.86 | 7.61 | 8.46 | 7.52 |
| 57 | 10.37 | 10.86 | 8.81 | 8.72 | 8.23 | 7.78 | 12.48 | 9.75 | 6.27 | 5.91 | 9.4 | 9.57 | 10.13 | 10.65 | 10.29 | 10.86 | 10.79 | 11.18 |
| 58 | 9.74 | 9.47 | 7.67 | 7.45 | 7.13 | 7.5 | 7.75 | 8.11 | 5.98 | 5.98 | 8.41 | 8.41 | 7.5 | 8.2 | 9.13 | 8.73 | 8.69 | 9.85 |
| 59 | 16.32 | 15.42 | 13.34 | 13.74 | 10.92 | 11.8 | 13.58 | 14.72 | 8.35 | 8.66 | 12.3 | 13.74 | 12.58 | 12.82 | 14.55 | 14.13 | 15.52 | 18.44 |
| 60 | 14.28 | 14.96 | 11.82 | 12.07 | 10.3 | 11.02 | 13.34 | 11.79 | 7.95 | 8.77 | 12.28 | 14.14 | 12.5 | 11.43 | 13.11 | 13.27 | 13.01 | 13.8 |
| 61 | 9.84 | 9.81 | 6.96 | 7.16 | 7.74 | 7.44 | 9.07 | 8.91 | 6.44 | 5.3 | 8.4 | 9.5 | 9.28 | 8.39 | 9.49 | 8.67 | 7.52 | 8.16 |
| 62 | 15.05 | 16.89 | 12.94 | 12.24 | 11.12 | 11.18 | 12.99 | 15.01 | 9.11 | 8.22 | 12.53 | 12.9 | 13.16 | 13.53 | 14.27 | 14.54 | 12.5 | 13.06 |
| 63 | 22.87 | 25.75 | 20.17 | 19.07 | 17.07 | 15.96 | 17.94 | 18.92 | 12.25 | 14.4 | 21.02 | 23.44 | 20.8 | 21.21 | 22.87 | 21.37 | 26.42 | 20.28 |
| 64 | 8.6 | 8.55 | 6.15 | 8.16 | 6 | 6.03 | 6.45 | 7.64 | 4.63 | 4.57 | 6.89 | 7.19 | 7.36 | 7.7 | 7.68 | 7.56 | 7.36 | 8.37 |
| 65 | 20.96 | 20.82 | 18.35 | 19.39 | 16.58 | 14.65 | 17.25 | 17.52 | 12.03 | 11.76 | 19.28 | 19.54 | 17.81 | 17.3 | 20.03 | 20.82 | 23.09 | 21.47 |
| 66 | 22.26 | 20.83 | 19.11 | 18.6 | 16.53 | 20.83 | 16.98 | 16.89 | 13.22 | 12.2 | 21.27 | 18.62 | 18.69 | 18.55 | 19.71 | 17.89 | 20.83 | 23.45 |
| 67 | 20.79 | 19.92 | 17.16 | 19.15 | 15.2 | 14.79 | 13.75 | 16.3 | 10.97 | 10.45 | 20.79 | 18.05 | 18.42 | 17.35 | 20.25 | 18.03 | 24.91 | 24.27 |
| 68 | 14.34 | 15.33 | 11.04 | 13.07 | 9.31 | 10.34 | 10.45 | 11.02 | 8.19 | 9.22 | 11.02 | 15.33 | 11.84 | 13.14 | 11.39 | 12.52 | 14.49 | 15.35 |
| 69 | 14.6 | 15.09 | 11.61 | 12.13 | 12.15 | 10.78 | 13.31 | 13.99 | 8.02 | 7.25 | 14.34 | 12.78 | 12.86 | 12.85 | 14.14 | 13.08 | 14.49 | 14.48 |
| 70 | 14.4 | 13.66 | 11.18 | 11.19 | 10.3 | 10.72 | 11.9 | 13.95 | 8.03 | 9.09 | 13.29 | 13.02 | 12.28 | 13.66 | 12.71 | 13.31 | 11.17 | 13.71 |
| 71 | 17.64 | 16.26 | 12.04 | 13 | 13.4 | 13.66 | 12.97 | 15.22 | 9.81 | 9.47 | 13.84 | 14.53 | 17.64 | 13.76 | 14.92 | 14.6 | 16.93 | 12.38 |
| 72 | 21.84 | 20.35 | 18.1 | 17.95 | 15.13 | 15.74 | 15.11 | 14.51 | 10.11 | 9.93 | 18.63 | 20.35 | 15.99 | 16.24 | 17.88 | 17.53 | 22.6 | 27.21 |
| 73 | 13.87 | 13.97 | 11.12 | 11.62 | 10.87 | 10.06 | 11.54 | 12.05 | 7.35 | 7.65 | 13.29 | 13.22 | 11.65 | 10.59 | 12.04 | 11.95 | 11.99 | 12.67 |
| 74 | 14.76 | 14.66 | 11.57 | 11.95 | 10.09 | 10.03 | 12.22 | 12.43 | 8.05 | 7.27 | 13.77 | 11.9 | 12.37 | 13.74 | 13.08 | 13.1 | 14.14 | 13.96 |
| 75 | 9.54 | 9.41 | 7.03 | 7.67 | 6.61 | 6.74 | 7.21 | 7.85 | 4.98 | 4.85 | 9.11 | 8.15 | 7.71 | 7.74 | 7.99 | 8.19 | 8.89 | 7.67 |
| 76 | 10.85 | 11.74 | 8.58 | 8.95 | 8.79 | 8.41 | 8.69 | 11.19 | 6.31 | 6.32 | 9.63 | 10.84 | 9.93 | 11.74 | 10.47 | 10.04 | 9.69 | 10.16 |
| 77 | 21.2 | 23.37 | 14.67 | 16.54 | 17.38 | 16.36 | 19.18 | 16.06 | 12.52 | 11.29 | 18.9 | 18.9 | 18.06 | 19.3 | 19.47 | 19.59 | 19.32 | 21.33 |
| 78 | 12.65 | 13.87 | 10.96 | 10.52 | 9.98 | 9.3 | 12.86 | 12.1 | 7.02 | 8.01 | 9.9 | 13.87 | 10.61 | 12.39 | 12.65 | 13 | 11.98 | 12.58 |
| 79 | 15.74 | 15.87 | 11.85 | 13.36 | 12.77 | 13.1 | 14.08 | 14.08 | 9.25 | 9 | 14.18 | 14.79 | 14.66 | 15.59 | 15.01 | 15 | 13.6 | 18.26 |
| 80 | 17.05 | 20.45 | 13.74 | 13.17 | 10.89 | 11.25 | 14.14 | 14.32 | 8.84 | 8.48 | 17.05 | 13.86 | 12.41 | 14.43 | 16.51 | 14.81 | 16.27 | 14.99 |

| Region of Interest | Inferior parietal, but supramarginal and angular gyri | Inferior parietal, but supramarginal and angular gyri | Inferior temporal gyrus | Inferior temporal gyrus | Insula | Insula | Lenticular nucleus, pallidum | Lenticular nucleus, pallidum | Lenticular nucleus, putamen | Lenticular nucleus, putamen | Lingual gyrus | Lingual gyrus | Mesial temporal lobe | Mesial temporal lobe | Middle cingulate and paracingulate gyri | Middle cingulate and paracingulate gyri | Middle frontal gyrus | Middle frontal gyrus |
| --- | --- | --- | --- | --- | --- | --- | --- | --- | --- | --- | --- | --- | --- | --- | --- | --- | --- | --- |
| Side | L | R | L | R | L | R | L | R | L | R | L | R | L | R | L | R | L | R |
| Number 1 | 9.53 | 8.95 | 8.79 | 8.58 | 8.57 | 8.72 | 6.08 | 5.18 | 10 | 9.99 | 9.93 | 10.26 | 7.13 | 6.58 | 10.46 | 11.12 | 9.69 | 9.65 |
| 2 | 12.67 | 12.05 | 11.7 | 11.04 | 12.03 | 11.77 | 9.91 | 9.57 | 12.97 | 12.57 | 15.98 | 13.91 | 9.81 | 8.62 | 14.48 | 12.87 | 13.8 | 14.2 |
| 3 | 14.71 | 13.4 | 12.9 | 13.54 | 12.98 | 12.5 | 11.69 | 8.42 | 14.57 | 13.68 | 17.69 | 17.53 | 9.45 | 11.25 | 14.64 | 14.89 | 16.99 | 14.77 |
| 4 | 16.56 | 16.6 | 13.39 | 13.8 | 18.58 | 16.17 | 13.06 | 8.98 | 17.83 | 18.16 | 17.08 | 18 | 13.21 | 12.41 | 15.83 | 17.93 | 17.07 | 18.36 |
| 5 | 19.5 | 19.27 | 17.15 | 19.16 | 18.25 | 16.51 | 15.38 | 12.03 | 19.55 | 20.03 | 21.37 | 20.7 | 15.1 | 13.52 | 22.95 | 21.28 | 22.33 | 22.42 |
| 6 | 12.35 | 12.47 | 11.14 | 12.41 | 11.59 | 11.31 | 8.68 | 8.39 | 12.88 | 12.38 | 11.22 | 11.11 | 9.35 | 9.49 | 14.5 | 14.28 | 16.26 | 13.46 |
| 7 | 18.74 | 17.56 | 15.92 | 18.74 | 16.32 | 16.63 | 12.77 | 10.93 | 18.76 | 19.25 | 18.19 | 18.9 | 12.98 | 13.96 | 16.44 | 18.54 | 18.97 | 18.37 |
| 8 | 10.53 | 10.8 | 9.79 | 9.85 | 10.53 | 11.09 | 9.19 | 6.52 | 12.64 | 12.86 | 10.72 | 11.47 | 8.5 | 8.06 | 12.14 | 13.6 | 14.15 | 11.94 |
| 9 | 16.77 | 15.92 | 15.02 | 15.52 | 16.35 | 17.82 | 12.55 | 9.22 | 17.19 | 17.41 | 19.83 | 18.92 | 11.35 | 11.52 | 17.84 | 17.64 | 20.19 | 20.91 |
| 10 | 12.44 | 12.6 | 11.96 | 11.62 | 12.83 | 12.25 | 10.14 | 7.27 | 12.89 | 12.85 | 13.69 | 12.43 | 10.05 | 9.42 | 12.52 | 13.52 | 14.76 | 14.69 |
| 11 | 25.93 | 23.51 | 19.67 | 21.17 | 17.93 | 20.01 | 17.3 | 15.02 | 24.73 | 23.45 | 22.61 | 26.48 | 20.87 | 16.69 | 23.82 | 24.74 | 25.61 | 25.3 |
| 12 | 16.52 | 15.85 | 14.2 | 13.72 | 15.97 | 16.43 | 11.42 | 9.55 | 18.1 | 16.43 | 14.78 | 14.98 | 10.76 | 12.64 | 21.09 | 21.36 | 18.99 | 18.06 |
| 13 | 20.35 | 18.83 | 15.96 | 16.51 | 14.82 | 16.53 | 12.65 | 11.92 | 17.29 | 17.72 | 20.67 | 25.19 | 12.77 | 13.85 | 19.5 | 19.12 | 19.88 | 24.25 |
| 14 | 19.54 | 19.76 | 14.05 | 15.92 | 15.47 | 16.83 | 13.42 | 11.35 | 18.71 | 18.74 | 18.1 | 18 | 10.97 | 10.92 | 18.55 | 21.13 | 18.87 | 19.23 |
| 15 | 8.34 | 7.39 | 6.68 | 6.78 | 6.86 | 6.5 | 5.25 | 5.73 | 8.68 | 8.97 | 8.73 | 9.12 | 6.29 | 6.23 | 8.1 | 8.28 | 8.74 | 8.01 |
| 16 | 9.43 | 9.09 | 7.75 | 7.67 | 8.61 | 8.33 | 6.71 | 5.78 | 9.33 | 9.05 | 8.35 | 8.45 | 7.3 | 6.65 | 8.56 | 8.81 | 10.57 | 9.89 |
| 17 | 16.43 | 15.19 | 14.82 | 13.49 | 14.56 | 15.05 | 10.93 | 8.67 | 15.92 | 14.91 | 15.89 | 18.86 | 11.79 | 10.04 | 16.61 | 16.86 | 17.78 | 18.63 |
| 18 | 18.52 | 17.43 | 15.88 | 15.73 | 16.55 | 17.3 | 14.65 | 11.47 | 21.51 | 20.36 | 17.61 | 17.97 | 13.25 | 12.08 | 18.33 | 19.68 | 19.61 | 20.04 |
| 19 | 20.55 | 21.07 | 17.78 | 21.97 | 19.75 | 20.4 | 19.06 | 13.85 | 22.63 | 21.92 | 26.24 | 25.93 | 13.02 | 13.47 | 23.2 | 21.46 | 24.38 | 21.58 |
| 20 | 13.95 | 14.79 | 11.9 | 12.84 | 16.01 | 14.96 | 14.65 | 10.77 | 15.96 | 16.31 | 17.28 | 18.57 | 9.63 | 10.18 | 14.13 | 15.14 | 16.3 | 14.98 |
| 21 | 28.59 | 21.2 | 17.67 | 17.29 | 19.87 | 19.84 | 16.76 | 14.76 | 22 | 22.45 | 20.68 | 20.41 | 14.38 | 13.58 | 24.23 | 22.87 | 24.41 | 24.15 |
| 22 | 17.53 | 16.07 | 15.17 | 15.11 | 16.92 | 14.98 | 12.91 | 10.91 | 16.66 | 16.43 | 16.55 | 17.79 | 12.57 | 11.66 | 19.42 | 21.25 | 20.97 | 19.65 |
| 23 | 13.58 | 12.14 | 11.18 | 11.14 | 11.23 | 10.81 | 7.76 | 6.33 | 13.13 | 13.14 | 13.94 | 14.94 | 9.55 | 8.73 | 12.85 | 13.83 | 13.3 | 14.26 |
| 24 | 23.62 | 31.77 | 28.36 | 23.36 | 20.18 | 19.46 | 17.67 | 17.11 | 21.94 | 24.9 | 21.32 | 20.57 | 16.44 | 16.12 | 29.67 | 25.72 | 33.31 | 23.72 |
| 25 | 12.06 | 11.18 | 12.7 | 10.63 | 12.73 | 12.24 | 10.29 | 7.89 | 13.99 | 13.92 | 11.29 | 9.69 | 9.43 | 9.87 | 12.95 | 14 | 15.65 | 13.84 |
| 26 | 14.01 | 17.14 | 12.88 | 13.18 | 13.51 | 13.34 | 12.18 | 10.77 | 15.46 | 14.69 | 16.75 | 17.24 | 10.39 | 11.16 | 16.85 | 15.49 | 18.68 | 17.44 |
| 27 | 10.47 | 10.92 | 10.58 | 10.6 | 11.61 | 10.34 | 10.23 | 8.15 | 12.17 | 12.2 | 17.03 | 13.82 | 9.43 | 9.84 | 11.94 | 15.89 | 13.23 | 12.05 |
| 28 | 9.67 | 11.13 | 10.31 | 9.56 | 8.22 | 10.75 | 8.1 | 6.75 | 11.99 | 10.89 | 10.68 | 12.35 | 7.77 | 8.04 | 10.34 | 10.03 | 11.47 | 10.5 |
| 29 | 14.9 | 14.33 | 15.78 | 14.42 | 14.97 | 14.83 | 11.1 | 10.46 | 15.66 | 15.42 | 18.09 | 20.14 | 10.39 | 11.78 | 18.07 | 16.81 | 17.53 | 16.17 |
| 30 | 13.24 | 12.12 | 11.12 | 10.48 | 11.64 | 11.84 | 9.21 | 9.13 | 12.91 | 12.75 | 12.6 | 12.25 | 8.92 | 7.95 | 14.21 | 13.39 | 13.4 | 13.44 |
| 31 | 11.96 | 12.21 | 11.56 | 10.69 | 9.82 | 11.36 | 8.09 | 6.28 | 12.12 | 12.01 | 14.7 | 16.13 | 9.26 | 9.34 | 14.83 | 15.47 | 14.71 | 13.75 |
| 32 | 14.51 | 15.31 | 15.25 | 15.86 | 15.71 | 16.04 | 11.64 | 10.51 | 15.88 | 15.43 | 15.9 | 16.23 | 11.24 | 11.85 | 15.63 | 18.28 | 17.52 | 18.16 |
| 33 | 5.71 | 6.01 | 6.33 | 7.05 | 6.38 | 5.84 | 6.07 | 5.65 | 7.42 | 7.39 | 8.16 | 8.59 | 4.99 | 5.62 | 7.13 | 7.57 | 6.48 | 7.42 |
| 34 | 17.92 | 14.78 | 14.06 | 13.34 | 15.23 | 14.37 | 10.43 | 9.94 | 13.75 | 15.21 | 15.93 | 14.6 | 10.3 | 9.72 | 14.54 | 16.04 | 15.69 | 14.65 |
| 35 | 16.13 | 14.07 | 12.5 | 12.66 | 12.53 | 12.37 | 11.82 | 9.49 | 14.62 | 12.96 | 16 | 14.35 | 8.53 | 9.17 | 13.84 | 14.58 | 16.18 | 14.23 |
| 36 | 14.98 | 12.69 | 11.38 | 12.58 | 12.38 | 11.63 | 10.95 | 8.45 | 13.16 | 13.31 | 13.27 | 14.7 | 8.45 | 9.23 | 13.91 | 15.2 | 14.82 | 15.14 |
| 37 | 15.63 | 14.49 | 12.5 | 12.08 | 12.27 | 12.45 | 9.51 | 8.35 | 14.27 | 14.23 | 16.52 | 16.37 | 9.17 | 9.63 | 14.09 | 14.6 | 16.31 | 15.36 |
| 38 | 20.48 | 20.02 | 16.56 | 17.6 | 18.69 | 20 | 21.11 | 18.6 | 21 | 21.15 | 20.01 | 25.18 | 14.86 | 15.5 | 21.94 | 24.14 | 24.02 | 22.93 |
| 39 | 13.25 | 12.26 | 11.49 | 11.1 | 12.29 | 10.45 | 9.19 | 6.57 | 12.96 | 12.54 | 12.93 | 13.29 | 9.49 | 8.63 | 12.83 | 13.31 | 13.33 | 12.83 |
| 40 | 16.14 | 17.85 | 13.54 | 14.16 | 14.37 | 14.17 | 11.49 | 10.28 | 16.96 | 17.57 | 14.62 | 15.79 | 12.4 | 10.8 | 17.23 | 19.41 | 20.18 | 18.59 |
| 41 | 20.08 | 18.6 | 14.94 | 14.11 | 17.28 | 15.48 | 11.81 | 9.24 | 15.04 | 15.28 | 17.75 | 19.68 | 12.5 | 10.61 | 20.18 | 18.6 | 19.47 | 18.9 |
| 42 | 21.71 | 23.06 | 17.79 | 17.37 | 18.34 | 17.14 | 10.88 | 9.15 | 17.43 | 16.73 | 20.56 | 19.12 | 13.38 | 13.97 | 22.27 | 23.61 | 22.1 | 19.67 |
| 43 | 11.66 | 11.02 | 10.92 | 10.29 | 10.37 | 9.56 | 8.31 | 7.08 | 11.62 | 11.2 | 12.94 | 12.82 | 7.73 | 7.99 | 13.28 | 13.08 | 13.34 | 11.78 |
| 44 | 10.42 | 11.68 | 9.37 | 9.72 | 10.83 | 9.45 | 9.14 | 8.45 | 11.87 | 10.62 | 9.7 | 11.05 | 6.99 | 7.92 | 11.43 | 11.22 | 11.98 | 12.26 |
| 45 | 7.55 | 7.13 | 6.72 | 6.39 | 6.47 | 6.71 | 6.11 | 6.04 | 7.92 | 7.81 | 6.91 | 6.95 | 5.54 | 5.52 | 8.07 | 7.49 | 7.72 | 7.75 |
| 46 | 15.23 | 14.3 | 11.26 | 11.8 | 12.08 | 12.47 | 12.25 | 9.83 | 16.3 | 14.76 | 14.97 | 15.51 | 9.54 | 11.84 | 14.14 | 15.31 | 14.29 | 14.44 |
| 47 | 15.12 | 14.52 | 13.13 | 15.15 | 14.54 | 13.7 | 9.74 | 8.47 | 15.07 | 15.78 | 15.28 | 17.1 | 10.7 | 11.12 | 15.01 | 17.9 | 15.73 | 16.83 |
| 48 | 20.89 | 22.12 | 17.47 | 16.93 | 19.77 | 18.86 | 12.86 | 10.5 | 22.34 | 20.15 | 21.1 | 24.16 | 14.38 | 14.57 | 22.36 | 22.18 | 24.8 | 24.31 |
| 49 | 10.23 | 9.17 | 8.44 | 8.63 | 8.98 | 8.49 | 8.36 | 6.22 | 9.95 | 9.04 | 11.59 | 10.03 | 7.84 | 7.04 | 9.83 | 10.68 | 10.73 | 10.33 |
| 50 | 21.3 | 19.41 | 19.48 | 19 | 18.71 | 19.55 | 17.96 | 13.94 | 23.06 | 23.37 | 27.13 | 25.15 | 15.45 | 14.9 | 20.92 | 20.11 | 23.39 | 22.16 |
| 51 | 10.63 | 10.92 | 8.94 | 8.87 | 8.66 | 10.57 | 9.64 | 8.83 | 12.93 | 13.96 | 12.03 | 10.85 | 6.93 | 7.66 | 11 | 11.71 | 11.74 | 11.04 |
| 52 | 15.64 | 14.69 | 14.12 | 13.87 | 14.38 | 13.7 | 14.12 | 12.61 | 17.72 | 16.91 | 16.78 | 20.84 | 9.9 | 10.31 | 16.26 | 17.35 | 18.32 | 18.43 |
| 53 | 20.5 | 21.59 | 19.03 | 18.71 | 16.77 | 19.15 | 12.62 | 10.83 | 20.12 | 19.45 | 19 | 21.73 | 12.66 | 12 | 21.96 | 22.53 | 23.13 | 22.5 |
| 54 | 15.52 | 14.08 | 13.34 | 15.04 | 13.68 | 13.7 | 12.48 | 10.7 | 15.99 | 15.05 | 15.35 | 15.68 | 11.88 | 11.5 | 17.42 | 18.27 | 16.25 | 15.71 |
| 55 | 10.13 | 9.9 | 8.77 | 9.68 | 9.45 | 8.64 | 7.2 | 7.12 | 10.63 | 9.85 | 10.17 | 10.79 | 6.97 | 6.82 | 10.34 | 10.63 | 10.37 | 11.18 |
| 56 | 7.73 | 7.82 | 7.2 | 7.7 | 8.04 | 7.68 | 5.87 | 5.15 | 8.82 | 8.72 | 7.41 | 8.03 | 5.79 | 5.82 | 8.86 | 9.2 | 10.13 | 8.56 |
| 57 | 9.12 | 9.39 | 8.6 | 8.74 | 9.91 | 9.32 | 7.39 | 6.41 | 10.42 | 9.59 | 9.28 | 10.22 | 7.3 | 6.21 | 9.64 | 9.83 | 9.91 | 9.45 |
| 58 | 8.21 | 8.69 | 7.94 | 8.73 | 7.41 | 8.57 | 5.39 | 5.1 | 9.67 | 8.45 | 8.71 | 9.93 | 6.82 | 5.98 | 8.84 | 9.03 | 9.74 | 9.04 |
| 59 | 14.2 | 13.87 | 11.69 | 12.45 | 13.53 | 11.78 | 9.84 | 8.23 | 15.91 | 16.84 | 16.16 | 17.09 | 10.43 | 10 | 15.04 | 15.39 | 15.54 | 15.42 |
| 60 | 12.65 | 11.61 | 10.26 | 11.53 | 10.66 | 11.73 | 8.37 | 7.9 | 13.04 | 12.97 | 12.14 | 13.31 | 7.95 | 9.1 | 14.04 | 15.27 | 14.28 | 13.7 |
| 61 | 8.24 | 8.98 | 6.68 | 6.86 | 8.67 | 8.35 | 7.93 | 6.64 | 10.51 | 9.78 | 7.89 | 7.77 | 6.45 | 5.67 | 9.17 | 10.02 | 9.45 | 8.63 |
| 62 | 13.34 | 11.66 | 11.05 | 11.65 | 12.26 | 11.32 | 11.12 | 9.34 | 14.79 | 13.71 | 12.54 | 13.09 | 9.71 | 9.22 | 13.92 | 15.32 | 15 | 16.89 |
| 63 | 20.14 | 19.63 | 18.49 | 17.5 | 19.88 | 19.32 | 14.71 | 13.77 | 18.33 | 19.51 | 21.03 | 21.48 | 14.73 | 14.4 | 19.94 | 23.13 | 22.02 | 25.75 |
| 64 | 6.92 | 6.81 | 6.73 | 6.75 | 6.44 | 7.56 | 6.29 | 5.18 | 7.78 | 8.41 | 7.63 | 8.67 | 5.3 | 5.36 | 8.14 | 7.51 | 8.6 | 8.55 |
| 65 | 16.97 | 18.61 | 16.17 | 17.53 | 16.74 | 17.82 | 12.58 | 11.11 | 20.45 | 19.63 | 23.02 | 22.27 | 17.13 | 14.33 | 20 | 20.72 | 20.96 | 20.41 |
| 66 | 20.72 | 19.35 | 17.01 | 19.04 | 18.85 | 17.93 | 13.29 | 11.34 | 18.57 | 17.76 | 25.46 | 23.81 | 13.68 | 13.81 | 20.05 | 21.01 | 22.26 | 20.58 |
| 67 | 17.05 | 15.09 | 15.11 | 15.1 | 17.58 | 16.91 | 10.74 | 8.63 | 16.95 | 15.62 | 23.56 | 25.28 | 13.72 | 13.69 | 18.07 | 17.61 | 18.47 | 19.92 |
| 68 | 11.84 | 10.53 | 10.7 | 11.07 | 10.93 | 12.18 | 10.47 | 7.49 | 12.37 | 12.52 | 13.36 | 15.32 | 9.54 | 9.46 | 12.58 | 13.93 | 14.34 | 14.48 |
| 69 | 13.1 | 12.89 | 11.41 | 11.83 | 13.61 | 12.71 | 13.2 | 11.94 | 14.36 | 13.33 | 13.64 | 15.94 | 9.57 | 8.08 | 13.13 | 14.3 | 14.42 | 15.09 |
| 70 | 14.34 | 12.74 | 12.28 | 12.01 | 12.08 | 12.64 | 10.25 | 8.79 | 14.25 | 14.6 | 13.19 | 13.06 | 10.12 | 10.63 | 13.93 | 14.96 | 14.4 | 13.49 |
| 71 | 15 | 15.45 | 12.16 | 13.22 | 13.98 | 12.14 | 10.28 | 10.92 | 14.52 | 13.94 | 16.65 | 14.5 | 9.84 | 10 | 16.18 | 16.06 | 16.43 | 16.26 |
| 72 | 18.41 | 15.68 | 15.33 | 16.87 | 16.16 | 15.52 | 12.17 | 9.93 | 18.35 | 18.21 | 24.61 | 23.56 | 11.89 | 11.23 | 18.26 | 20.32 | 20.16 | 18.44 |
| 73 | 12.1 | 13.21 | 11.62 | 11.64 | 11.71 | 10.59 | 11.1 | 9.09 | 13.19 | 13.52 | 13.75 | 13.94 | 8.32 | 8.23 | 13.22 | 12.6 | 13.87 | 13.97 |
| 74 | 13.22 | 11.85 | 11.5 | 10.79 | 11.51 | 12.23 | 8.76 | 7.88 | 12.62 | 11.5 | 13.96 | 14.26 | 8.65 | 8.94 | 12.93 | 13.55 | 14.05 | 14.66 |
| 75 | 7.02 | 6.91 | 6.7 | 7.02 | 7.77 | 8.06 | 7.06 | 5.49 | 7.76 | 8.04 | 9.1 | 8.81 | 5.68 | 5.35 | 8.13 | 8.52 | 8.69 | 9.41 |
| 76 | 9.86 | 9.36 | 8.38 | 8.9 | 8.94 | 8.8 | 7.91 | 7.02 | 10.43 | 10.56 | 10.11 | 10.72 | 7.7 | 7.77 | 10.65 | 11.1 | 10.85 | 10.68 |
| 77 | 21.18 | 20.27 | 16.93 | 16.51 | 18.59 | 18 | 15.98 | 12.59 | 19.66 | 20.24 | 20.04 | 20.78 | 14.09 | 13.38 | 23.23 | 21.12 | 21.2 | 23.37 |
| 78 | 11.97 | 11.62 | 9.14 | 9.92 | 10.58 | 10.52 | 10.95 | 8.24 | 12.83 | 11.85 | 12.29 | 12.76 | 7.63 | 9.1 | 12.34 | 12.51 | 12.65 | 12.88 |
| 79 | 13.9 | 14.43 | 12.26 | 13.04 | 14.19 | 13.1 | 11.71 | 10.36 | 16 | 16.74 | 14.74 | 15.07 | 11.69 | 11.25 | 15.13 | 16.17 | 15.74 | 15.87 |
| 80 | 14.04 | 14.89 | 14.17 | 13.12 | 14.34 | 13.61 | 11.64 | 10.98 | 14.15 | 14.65 | 15.52 | 16.37 | 10.04 | 10.39 | 14.33 | 15.82 | 15.64 | 20.45 |

| Region of Interest | Middle frontal gyrus, orbital part | Middle frontal gyrus, orbital part | Middle occipital gyrus | Middle occipital gyrus | Middle temporal gyrus | Middle temporal gyrus | Occipital lobe | Occipital lobe | Olfactory cortex | Olfactory cortex | Paracentral lobule | Paracentral lobule | Parahippocampal gyrus | Parahippocampal gyrus | Parietal lobe | Parietal lobe | Postcentral gyrus | Postcentral gyrus |
| --- | --- | --- | --- | --- | --- | --- | --- | --- | --- | --- | --- | --- | --- | --- | --- | --- | --- | --- |
| Side | L | R | L | R | L | R | L | R | L | R | L | R | L | R | L | R | L | R |
| Number 1 | 9.01 | 8.78 | 10.35 | 9.47 | 9 | 9.16 | 11.38 | 11.2 | 6.4 | 7.28 | 9.79 | 8.95 | 7.13 | 6.58 | 10.49 | 10.6 | 9.21 | 9.79 |
| 2 | 12.76 | 13.01 | 14.99 | 14.7 | 12.27 | 12.27 | 17.38 | 16.6 | 9.02 | 9.08 | 11.9 | 12.35 | 9.81 | 8.62 | 13.58 | 13.71 | 14.49 | 12.07 |
| 3 | 12.82 | 12.74 | 16.92 | 15.44 | 13.63 | 13.48 | 19.81 | 18.67 | 9.35 | 8.84 | 16.4 | 15.82 | 9.45 | 11.25 | 18.78 | 15.54 | 13.67 | 17.25 |
| 4 | 14.66 | 15.94 | 17.7 | 16.55 | 14.54 | 17.28 | 20.22 | 19.71 | 10.85 | 10.87 | 20.61 | 17.49 | 13.21 | 12.41 | 17 | 18.6 | 15.63 | 16.33 |
| 5 | 20.43 | 20.32 | 21.59 | 19.56 | 18.31 | 19.24 | 25.69 | 21.43 | 14.17 | 13.68 | 20.39 | 18.07 | 14.43 | 13.52 | 24.92 | 22.15 | 19.34 | 19.72 |
| 6 | 11.11 | 11.82 | 11.23 | 12.4 | 11.56 | 12.7 | 14.9 | 12.76 | 8.55 | 8.94 | 11.31 | 12.67 | 9.1 | 9.49 | 13.28 | 13.57 | 12.24 | 11.76 |
| 7 | 19.01 | 18.17 | 20.91 | 22.32 | 16.25 | 17.21 | 20.91 | 22.91 | 13.8 | 13.24 | 15 | 15.98 | 12.98 | 13.96 | 19.92 | 18.66 | 15.56 | 16.81 |
| 8 | 11.88 | 12.47 | 10.64 | 11.16 | 11.19 | 11.47 | 11.78 | 12.52 | 8.13 | 8.14 | 9.79 | 8.87 | 8.5 | 8.06 | 12.74 | 11.84 | 11.26 | 11.07 |
| 9 | 18.56 | 16.72 | 16.9 | 17.02 | 18.05 | 16.23 | 19.83 | 19.6 | 12.78 | 14.64 | 16.4 | 15.26 | 11.35 | 11.52 | 18.15 | 19.32 | 15.34 | 17.39 |
| 10 | 12.84 | 11.95 | 14.24 | 13.51 | 12.7 | 12.36 | 14.36 | 15.68 | 8.99 | 9.56 | 10.78 | 11.3 | 10.05 | 9.42 | 13.15 | 14.4 | 11.52 | 11.69 |
| 11 | 22.1 | 24.18 | 25.72 | 27.29 | 21.34 | 20.53 | 27.68 | 30.34 | 17.56 | 15.59 | 19.43 | 22.16 | 20.87 | 16.69 | 28.98 | 30.84 | 21.08 | 21.02 |
| 12 | 16.53 | 17.22 | 15.82 | 18 | 15.26 | 15.92 | 18.69 | 19.64 | 11.49 | 12.37 | 18.02 | 15.33 | 10.76 | 12.64 | 18.49 | 16.71 | 16.97 | 15.91 |
| 13 | 18.61 | 19.87 | 21 | 20.99 | 16.48 | 20.14 | 21.59 | 25.19 | 13.88 | 12.64 | 16.14 | 17.26 | 12.77 | 13.85 | 20.35 | 20.4 | 18.04 | 15.44 |
| 14 | 15.83 | 15.02 | 19.45 | 19.05 | 18.41 | 18.22 | 21.94 | 19.39 | 11.41 | 11.54 | 16.4 | 16.52 | 10.97 | 10.92 | 20.36 | 23.91 | 16.76 | 20.85 |
| 15 | 8.06 | 6.75 | 9.03 | 8.88 | 7.28 | 7.4 | 9.67 | 9.36 | 5.41 | 5.55 | 7.85 | 7.66 | 6.29 | 6.23 | 8.92 | 9.32 | 8.52 | 8.96 |
| 16 | 9.03 | 8.66 | 9.99 | 8.82 | 8.06 | 7.83 | 9.99 | 10.24 | 6.52 | 6.65 | 9.45 | 8.08 | 7.3 | 6.65 | 9.82 | 10.06 | 8.79 | 8.91 |
| 17 | 16.54 | 16.11 | 17.29 | 17.39 | 15.08 | 15.8 | 17.29 | 18.86 | 13.31 | 13.81 | 15.81 | 14.71 | 11.55 | 9.93 | 16.91 | 17.89 | 16.06 | 15.98 |
| 18 | 18.06 | 18.54 | 19.99 | 19.29 | 17.12 | 16.1 | 20.98 | 20.86 | 13.3 | 11.99 | 16.46 | 14.67 | 13.25 | 12.08 | 19.64 | 21.33 | 16.91 | 17.16 |
| 19 | 21.05 | 20.31 | 22.34 | 22.7 | 21.1 | 21 | 28.05 | 26.82 | 14.36 | 15.01 | 17.46 | 16.09 | 13.02 | 13.47 | 25.69 | 22.1 | 18.28 | 19.81 |
| 20 | 15.27 | 13.21 | 20.62 | 16.9 | 12.66 | 13.87 | 20.83 | 19.7 | 9.82 | 11.36 | 13.89 | 14.3 | 9.63 | 10.18 | 15.61 | 14.79 | 14.16 | 13.87 |
| 21 | 20.49 | 19.04 | 24.19 | 21 | 18.73 | 19.97 | 24.38 | 25.67 | 12.58 | 14.95 | 21.02 | 20.12 | 14.38 | 13.58 | 28.59 | 25.25 | 23.05 | 24.34 |
| 22 | 16.79 | 16.94 | 19.5 | 19.61 | 16.65 | 17.43 | 19.5 | 22.21 | 15 | 11.83 | 17.22 | 16.44 | 12.57 | 11.54 | 19.93 | 20.71 | 18.42 | 16.26 |
| 23 | 11.9 | 12.07 | 12.2 | 13.43 | 11.09 | 12.65 | 16.13 | 14.94 | 7.98 | 8.35 | 13.36 | 11.51 | 9.55 | 8.73 | 14.24 | 14.89 | 12.48 | 11.78 |
| 24 | 21.79 | 18.06 | 23.28 | 27.98 | 20.89 | 26.23 | 24.11 | 27.98 | 17.83 | 14.9 | 30.54 | 21.42 | 15.13 | 16.12 | 28.18 | 31.77 | 28.63 | 21.82 |
| 25 | 14.29 | 12.31 | 12.14 | 11.25 | 12.09 | 12.21 | 12.36 | 13.02 | 8.71 | 9.37 | 13.69 | 11.3 | 9.43 | 9.87 | 14.52 | 13.9 | 13.97 | 11.38 |
| 26 | 14.73 | 14.96 | 18.36 | 14.9 | 15.66 | 13.74 | 18.36 | 17.24 | 10.57 | 10.9 | 15.5 | 13.92 | 10.39 | 11.16 | 16.47 | 17.14 | 15.92 | 14.54 |
| 27 | 11.68 | 11.1 | 12.54 | 13.56 | 11.78 | 11.5 | 17.03 | 16.71 | 8.03 | 8.69 | 12.36 | 10.86 | 9.43 | 9.84 | 12.84 | 12.61 | 12.8 | 11.05 |
| 28 | 9.49 | 9.47 | 13.46 | 11.43 | 9.88 | 9.6 | 14.22 | 13.57 | 7.39 | 7.18 | 11.1 | 10.05 | 7.77 | 8.04 | 10.17 | 12.15 | 9.85 | 9.63 |
| 29 | 14.67 | 15.98 | 17.8 | 17.94 | 16.18 | 15.37 | 20.5 | 20.55 | 10.76 | 10.79 | 14.55 | 14.22 | 10.39 | 11.78 | 16.25 | 16.9 | 15.38 | 16.31 |
| 30 | 12.23 | 12.11 | 13.12 | 11.22 | 12.28 | 11.11 | 15.01 | 13.06 | 9.47 | 8.81 | 11.26 | 9.76 | 8.92 | 7.95 | 13.7 | 12.65 | 12.05 | 11.28 |
| 31 | 11.47 | 11.22 | 15.05 | 12.13 | 11.58 | 10.9 | 17.67 | 16.21 | 7.85 | 8.34 | 12.36 | 10.5 | 9.26 | 9.34 | 14.21 | 13.36 | 11.9 | 11.37 |
| 32 | 14.55 | 14.97 | 20.72 | 24.23 | 15.02 | 14.71 | 20.72 | 24.23 | 9.46 | 10.56 | 15.38 | 14.24 | 11.24 | 11.85 | 17.46 | 17.47 | 16.1 | 15.79 |
| 33 | 6.07 | 6.25 | 8.43 | 7.71 | 6.48 | 6.42 | 8.47 | 9.24 | 5.74 | 5.3 | 6.19 | 6.46 | 4.99 | 5.62 | 7.24 | 7.29 | 7.42 | 7.19 |
| 34 | 16.46 | 14.21 | 17.36 | 15.24 | 14.93 | 13.57 | 19.87 | 19.31 | 9.89 | 8.53 | 14.95 | 14.3 | 10.3 | 9.72 | 21.2 | 16.19 | 15.53 | 15.43 |
| 35 | 13.04 | 11.86 | 16.34 | 15.48 | 14.24 | 13.08 | 16.34 | 16.14 | 8.09 | 8.72 | 13.74 | 11.06 | 8.53 | 9.17 | 16.13 | 16.35 | 13.61 | 13.05 |
| 36 | 13.47 | 12.34 | 13.17 | 12.77 | 12.43 | 13.66 | 14.93 | 17.18 | 10.13 | 10.46 | 11.46 | 11.95 | 8.2 | 9.23 | 14.98 | 16.3 | 13.79 | 12.38 |
| 37 | 12.95 | 14.37 | 17.57 | 19.28 | 13.16 | 12.98 | 17.68 | 19.28 | 9.33 | 9.1 | 13.48 | 12.55 | 9.17 | 9.63 | 16 | 16.12 | 13.79 | 12.49 |
| 38 | 20.1 | 18.22 | 27.64 | 22.35 | 18.47 | 18.66 | 27.64 | 26.95 | 12.78 | 12.34 | 20.75 | 18.39 | 14.69 | 15.5 | 23.61 | 25.17 | 20.78 | 19.62 |
| 39 | 12.26 | 11.63 | 12.65 | 12.72 | 11.48 | 11.7 | 14 | 16.22 | 8.58 | 8.02 | 11.32 | 12.03 | 9.49 | 8.63 | 13.25 | 12.53 | 12.49 | 12.68 |
| 40 | 20.59 | 17.27 | 16.55 | 15.67 | 18.13 | 16.72 | 20.21 | 19.38 | 12.37 | 10.15 | 16.44 | 16.11 | 10.21 | 10.8 | 20.31 | 21.06 | 16.67 | 16.09 |
| 41 | 16.16 | 16.13 | 16.58 | 17.49 | 15.52 | 15.6 | 19.86 | 19.79 | 11.34 | 11.16 | 16.62 | 15.2 | 12.5 | 10.61 | 20.34 | 18.63 | 16.61 | 15.54 |
| 42 | 19.16 | 19.41 | 19.35 | 17.5 | 18.8 | 17.06 | 23.49 | 22.27 | 13.81 | 13.73 | 17.44 | 17.11 | 13.38 | 13.97 | 21.71 | 23.06 | 16.61 | 15.51 |
| 43 | 11.09 | 10.21 | 12.07 | 11.26 | 11.21 | 11.07 | 14.87 | 14.89 | 8.09 | 9.17 | 11.5 | 10.44 | 7.73 | 7.95 | 12.61 | 12.5 | 11.03 | 10.71 |
| 44 | 10.33 | 10.75 | 11.01 | 11.6 | 10.46 | 10.84 | 11.89 | 13.15 | 7.12 | 6.73 | 9.51 | 9.7 | 6.99 | 7.92 | 11.83 | 12.29 | 12.34 | 11.56 |
| 45 | 6.96 | 7.32 | 7.11 | 7.37 | 6.97 | 6.64 | 7.74 | 8.09 | 5.27 | 5.21 | 7.15 | 7.41 | 5.54 | 5.52 | 7.68 | 8.65 | 7.12 | 7.3 |
| 46 | 12.46 | 14.04 | 14.67 | 14.67 | 13.95 | 13.36 | 16.28 | 16.83 | 8.85 | 10.41 | 12.99 | 14.26 | 9.54 | 11.84 | 15.74 | 17.31 | 13.02 | 13.68 |
| 47 | 14.08 | 15.42 | 16.09 | 14.73 | 14.07 | 15.21 | 17.69 | 17.4 | 9.81 | 11.56 | 15.44 | 12.24 | 10.7 | 11.12 | 16.74 | 17.3 | 13.84 | 14.84 |
| 48 | 19.31 | 20.33 | 20.96 | 20.52 | 20.34 | 18.26 | 21.47 | 25.65 | 14.68 | 13.76 | 18.8 | 16.78 | 14.38 | 14.57 | 21.03 | 22.37 | 18.41 | 22.27 |
| 49 | 8.53 | 9.13 | 10.97 | 10.17 | 9.34 | 8.42 | 11.59 | 12.34 | 7.59 | 7.55 | 8.91 | 8.84 | 7.84 | 7.04 | 10.72 | 10.11 | 9.43 | 8.87 |
| 50 | 19.19 | 19.22 | 24.28 | 20.85 | 19.5 | 18.92 | 27.93 | 27.82 | 14.17 | 15.1 | 19.74 | 17.81 | 15.45 | 14.9 | 22.14 | 21.69 | 18.38 | 19.96 |
| 51 | 9.74 | 11.53 | 10.92 | 11.04 | 11.32 | 10.88 | 12.03 | 12.39 | 7.41 | 7.06 | 10.62 | 10.22 | 6.93 | 7.66 | 11.51 | 12 | 11.17 | 11.36 |
| 52 | 15.15 | 16.5 | 19.97 | 16.07 | 15.06 | 14.52 | 19.97 | 20.89 | 9.4 | 10.45 | 13.87 | 13.36 | 9.9 | 10.31 | 18.25 | 17.57 | 14.79 | 15.75 |
| 53 | 18.86 | 22.95 | 20.58 | 20.01 | 18.63 | 19.3 | 20.58 | 21.73 | 14.07 | 15.26 | 17.06 | 17.02 | 12.66 | 12 | 22.06 | 21.7 | 17.75 | 18.92 |
| 54 | 15.14 | 14.19 | 17.6 | 18.02 | 13.62 | 14.96 | 17.6 | 18.02 | 10.73 | 11.6 | 12.69 | 11.77 | 11.88 | 11.5 | 16 | 17.8 | 13.32 | 13.56 |
| 55 | 9.23 | 9.54 | 10.48 | 11.13 | 9.22 | 9.8 | 13.05 | 11.64 | 6.84 | 7.04 | 10.13 | 10.14 | 6.97 | 6.82 | 10.47 | 12.43 | 10.15 | 10.39 |
| 56 | 8.28 | 8.42 | 8.95 | 8.01 | 7.7 | 8.12 | 9.23 | 9.08 | 6.46 | 5.96 | 7.58 | 7.91 | 5.79 | 5.82 | 9.1 | 9.07 | 7.57 | 8.15 |
| 57 | 9.12 | 9.23 | 10.79 | 11.87 | 9.52 | 10.22 | 11.34 | 11.87 | 7.06 | 7.25 | 9.44 | 8.74 | 7.3 | 6.21 | 10.28 | 10.21 | 9.85 | 10.27 |
| 58 | 8.21 | 8.08 | 8.09 | 8.81 | 7.56 | 8.37 | 9.56 | 9.93 | 6.77 | 5.58 | 7.97 | 8.56 | 6.82 | 5.88 | 9.09 | 9.49 | 8.39 | 9.3 |
| 59 | 12.78 | 13.61 | 16.16 | 15.24 | 13.18 | 13.41 | 17.81 | 19.13 | 9.56 | 9.21 | 13 | 11.9 | 10.43 | 10 | 16.25 | 15.45 | 14.18 | 13.73 |
| 60 | 11.66 | 11.69 | 13.92 | 13.5 | 12.81 | 12.45 | 13.92 | 14.19 | 8.31 | 9.14 | 11.67 | 11.01 | 7.94 | 9.1 | 12.65 | 14.07 | 12.94 | 11.57 |
| 61 | 8.67 | 8.49 | 8.21 | 8.06 | 8.26 | 8.62 | 8.74 | 8.25 | 7.18 | 6.83 | 8.14 | 9.81 | 6.45 | 5.67 | 9.67 | 9.05 | 8.67 | 8.89 |
| 62 | 12.5 | 12.79 | 12.68 | 13.53 | 11.55 | 13.33 | 14.16 | 15.38 | 9.12 | 8.09 | 13.29 | 13.06 | 9.71 | 9.22 | 14.95 | 15.68 | 12.99 | 14.2 |
| 63 | 19 | 18.33 | 24.93 | 20.95 | 18.14 | 18.59 | 26.42 | 27.06 | 14.97 | 15.11 | 18.05 | 20.85 | 14.73 | 13.92 | 25.08 | 24.47 | 19.48 | 18.91 |
| 64 | 7.29 | 7.24 | 8.65 | 8.32 | 7.32 | 8.31 | 9.18 | 8.69 | 5.57 | 5.11 | 6.78 | 7.62 | 5.3 | 5.36 | 9.05 | 8.42 | 7.04 | 6.94 |
| 65 | 16.63 | 18.63 | 21.56 | 18.63 | 16.47 | 18.19 | 23.33 | 22.27 | 14.49 | 12.81 | 17.55 | 17.3 | 17.13 | 14.33 | 21.95 | 21.02 | 17.77 | 18.26 |
| 66 | 19.22 | 19.43 | 24.42 | 21.16 | 17.04 | 17.76 | 25.88 | 25.17 | 12.46 | 12.43 | 17.07 | 16.8 | 13.68 | 13.81 | 23.27 | 22.7 | 18.18 | 16.75 |
| 67 | 16.05 | 16.48 | 20.12 | 18.69 | 17.43 | 16.34 | 24.91 | 25.28 | 12.46 | 12.66 | 15.46 | 15.5 | 13.72 | 13.69 | 20.5 | 19.97 | 19.54 | 15.71 |
| 68 | 11.8 | 12.36 | 13.87 | 13.67 | 11.36 | 11.35 | 15.21 | 15.35 | 8.62 | 9.38 | 13.12 | 11.76 | 9.54 | 9.46 | 12.54 | 13.48 | 13.24 | 13.57 |
| 69 | 13.65 | 13.21 | 16.34 | 15.14 | 12.17 | 12.89 | 16.34 | 17.89 | 10.16 | 9.9 | 14.6 | 12.09 | 9.57 | 8.08 | 16.04 | 14 | 14.66 | 14.56 |
| 70 | 12.08 | 12.19 | 13.52 | 13.56 | 12.62 | 11.79 | 14.3 | 16.41 | 8.9 | 8.55 | 12 | 12.26 | 10.12 | 10.63 | 14.34 | 15.8 | 14.47 | 13.07 |
| 71 | 14.86 | 13.86 | 14.13 | 14.01 | 14.12 | 14.58 | 16.93 | 18.8 | 10.6 | 9.29 | 14.16 | 11.83 | 9.84 | 10 | 17.03 | 21.35 | 15.52 | 12.65 |
| 72 | 16.14 | 16.79 | 20.92 | 20.6 | 17.55 | 17.6 | 24.61 | 27.21 | 12.17 | 12.49 | 15.66 | 15.99 | 11.89 | 11.23 | 18.41 | 19.45 | 17.67 | 14.69 |
| 73 | 12 | 11.75 | 12.16 | 12.17 | 11.35 | 11.37 | 13.76 | 14.45 | 8.26 | 9.04 | 10.27 | 11.08 | 8.32 | 8.23 | 13.52 | 13.21 | 11.7 | 11.39 |
| 74 | 11.58 | 12.39 | 14.63 | 12.48 | 12.14 | 12.7 | 14.63 | 15.83 | 9.07 | 8.5 | 13.05 | 10.62 | 8.65 | 8.94 | 13.91 | 14.24 | 11.34 | 12.65 |
| 75 | 7.14 | 7.5 | 7.83 | 8.39 | 6.84 | 7.46 | 9.17 | 9.62 | 5.68 | 6.14 | 7.61 | 6.8 | 5.68 | 5.35 | 8.06 | 8.69 | 8.83 | 7.88 |
| 76 | 9.12 | 9.1 | 10.38 | 9.74 | 10.29 | 9.04 | 11.87 | 11.43 | 7.18 | 6.91 | 8.65 | 9.27 | 7.7 | 7.77 | 11.87 | 11.54 | 9.32 | 9.64 |
| 77 | 19.35 | 19.91 | 19.29 | 19.42 | 18.67 | 18.18 | 22.01 | 21.33 | 13.15 | 14.82 | 16.84 | 17.4 | 14.09 | 13.38 | 22.1 | 21.4 | 18.37 | 17.81 |
| 78 | 11.11 | 11.52 | 11.28 | 13 | 10.39 | 10.74 | 13.53 | 13.78 | 7.83 | 8.96 | 10.94 | 9.68 | 7.63 | 9.1 | 12.13 | 12.7 | 11.43 | 12.91 |
| 79 | 13.48 | 12.97 | 16.5 | 15.66 | 13.35 | 14.33 | 17.99 | 18.26 | 11.21 | 10.29 | 12.63 | 13.53 | 11.69 | 11.25 | 16.92 | 16.44 | 14.5 | 14.5 |
| 80 | 12.21 | 14.36 | 15.53 | 16.98 | 14.76 | 14.17 | 17.05 | 17.75 | 10.8 | 9.2 | 13.46 | 13.52 | 10.04 | 10.39 | 18.51 | 17.58 | 14.99 | 13.37 |

| Region of Interest | Posterior cingulate and paracingulate gyri | Posterior cingulate and paracingulate gyri | Precentral gyrus | Precentral gyrus | Precuneus | Precuneus | Rolandic operculum | Rolandic operculum | Superior frontal gyrus, dorsolateral | Superior frontal gyrus, dorsolateral | Superior frontal gyrus, medial | Superior frontal gyrus, medial | Superior frontal gyrus, medial orbital | Superior frontal gyrus, medial orbital | Superior frontal gyrus, orbital part | Superior frontal gyrus, orbital part | Superior occipital gyrus | Superior occipital gyrus |
| --- | --- | --- | --- | --- | --- | --- | --- | --- | --- | --- | --- | --- | --- | --- | --- | --- | --- | --- |
| Side | L | R | L | R | L | R | L | R | L | R | L | R | L | R | L | R | L | R |
| Number 1 | 9.74 | 9.23 | 10.76 | 9.89 | 10.49 | 10.6 | 9.21 | 8.96 | 10.09 | 9.54 | 8.22 | 8.75 | 7.61 | 9.34 | 8.54 | 8.88 | 9.96 | 9.57 |
| 2 | 13.38 | 12.56 | 13.23 | 13.45 | 13.58 | 13.71 | 11.9 | 11.44 | 15.1 | 12.56 | 12.53 | 11.91 | 11.29 | 11.57 | 12.86 | 13.2 | 13.79 | 14.14 |
| 3 | 14.4 | 13.14 | 16.63 | 15.78 | 18.78 | 15.54 | 13.57 | 12.54 | 15.45 | 14.62 | 12.71 | 14.04 | 13.56 | 12.36 | 12.66 | 12.66 | 14.75 | 17.24 |
| 4 | 16.68 | 16.14 | 17.58 | 18.26 | 17 | 18.6 | 19.17 | 15.63 | 17.42 | 18.58 | 14.94 | 14.52 | 13.5 | 14.98 | 16.13 | 15.4 | 20.22 | 19.3 |
| 5 | 21.37 | 19.5 | 21.67 | 18.98 | 24.92 | 22.15 | 18.61 | 18.22 | 20.09 | 21.48 | 19.77 | 21.19 | 16.51 | 17.6 | 18.81 | 18.02 | 19.46 | 17.84 |
| 6 | 13.42 | 14.03 | 15.1 | 13.45 | 13.28 | 13.57 | 11.67 | 12.08 | 15.33 | 14.54 | 12.26 | 12.4 | 10.98 | 11.95 | 11.75 | 12.63 | 11.33 | 12.28 |
| 7 | 18.76 | 19.56 | 19.77 | 17.07 | 19.92 | 18.66 | 16.05 | 18.37 | 17.95 | 17.51 | 17.89 | 16.75 | 15.06 | 14.51 | 16.96 | 17.96 | 16.66 | 16.92 |
| 8 | 12.24 | 11.17 | 14.39 | 12.84 | 12.74 | 11.84 | 11.32 | 9.73 | 13.19 | 12.37 | 11.65 | 12.3 | 10.09 | 11.46 | 11.42 | 11.5 | 10.62 | 10.55 |
| 9 | 15.09 | 16.47 | 17.93 | 17.73 | 18.15 | 19.32 | 16.91 | 16.23 | 19.09 | 18.73 | 17.85 | 16.06 | 16.62 | 17.15 | 16.72 | 18.41 | 16.16 | 17.05 |
| 10 | 12.03 | 11.67 | 12.39 | 12.8 | 13.15 | 14.4 | 12.54 | 11.17 | 12.49 | 13.31 | 11.87 | 11.83 | 11.64 | 13.02 | 11.75 | 12.49 | 12.77 | 13.48 |
| 11 | 23.02 | 27.57 | 22.83 | 24.88 | 28.98 | 30.84 | 17.02 | 19.37 | 22.22 | 22.77 | 20.9 | 20.85 | 20.3 | 22.03 | 21.8 | 25.38 | 24.45 | 22.83 |
| 12 | 17.18 | 15.2 | 17.03 | 18.21 | 18.49 | 16.71 | 16.57 | 14.63 | 17.23 | 18.19 | 16.55 | 17.45 | 14.82 | 16.01 | 16.01 | 17.97 | 16.03 | 17.82 |
| 13 | 17.34 | 14.71 | 20.63 | 19.82 | 19.32 | 20.4 | 14.62 | 16.41 | 19.54 | 21.05 | 19.75 | 18.27 | 15.17 | 18.23 | 19.59 | 19.83 | 20.22 | 19.48 |
| 14 | 18.58 | 19.17 | 19.68 | 20.43 | 20.36 | 23.91 | 19.73 | 16.31 | 19.09 | 19.08 | 18.49 | 16.06 | 15.92 | 15.24 | 16.07 | 17.53 | 16.42 | 17.91 |
| 15 | 7.46 | 7.47 | 8.89 | 8.36 | 8.92 | 9.32 | 7.85 | 6.75 | 8.72 | 8.03 | 7.29 | 7.58 | 7.02 | 7.09 | 7.49 | 6.73 | 8.84 | 8.74 |
| 16 | 9.23 | 7.9 | 10.17 | 9.09 | 9.82 | 10.06 | 9.4 | 7.51 | 10.95 | 10.98 | 8.58 | 8.39 | 8.9 | 7.77 | 8.99 | 8.32 | 8.46 | 7.76 |
| 17 | 14.02 | 17.82 | 19.76 | 16.83 | 16.91 | 17.43 | 15.32 | 15.72 | 16.93 | 17.3 | 14.64 | 16.45 | 14.13 | 14.33 | 17.74 | 15.39 | 14 | 15.73 |
| 18 | 19.06 | 14.2 | 18.78 | 19.46 | 19.64 | 21.33 | 16.96 | 19.01 | 18.21 | 18.35 | 16 | 16.33 | 14.87 | 16.47 | 16.67 | 18.79 | 16.71 | 20.86 |
| 19 | 25.39 | 18.69 | 22.14 | 20.65 | 25.69 | 21.71 | 20.79 | 19.57 | 22.48 | 23.6 | 20.3 | 19.56 | 19.38 | 17.55 | 22.59 | 25.67 | 22.12 | 22.21 |
| 20 | 13.74 | 14.61 | 15.96 | 15.67 | 15.61 | 14.77 | 14.94 | 13.95 | 14.86 | 14.7 | 15.62 | 12.3 | 12.83 | 12.55 | 12.68 | 12.57 | 16.97 | 15.27 |
| 21 | 22.11 | 24.14 | 23.08 | 21.9 | 23.11 | 25.25 | 18.63 | 18.27 | 21.98 | 24.25 | 21.63 | 20.6 | 16.98 | 17.12 | 19.75 | 19.53 | 21.54 | 20.73 |
| 22 | 18.11 | 15.92 | 20.47 | 18.75 | 19.93 | 20.71 | 15.67 | 15.56 | 19.96 | 18.77 | 17.87 | 17.75 | 15.86 | 14.78 | 16.89 | 17.05 | 19.16 | 18.71 |
| 23 | 13.83 | 14.8 | 13.24 | 13.38 | 14.24 | 14.89 | 12.72 | 10.98 | 12.32 | 13.3 | 10.98 | 11.3 | 10.19 | 10.87 | 11.97 | 11.94 | 14.81 | 12.74 |
| 24 | 16.95 | 16.1 | 28.37 | 27.63 | 28.18 | 25.87 | 22.32 | 18.41 | 30.05 | 28.44 | 26.8 | 25.8 | 21.55 | 22.84 | 21.44 | 20.62 | 19.81 | 20.51 |
| 25 | 12.21 | 13.04 | 14.69 | 13.57 | 14.52 | 13.9 | 12.9 | 11.61 | 14.52 | 13.39 | 12.09 | 12.82 | 11.34 | 11.49 | 13.09 | 11.37 | 11.29 | 9.9 |
| 26 | 17.34 | 13.87 | 17.66 | 16.01 | 16.47 | 14.53 | 15.84 | 16.16 | 16.86 | 14.76 | 14.9 | 16.73 | 12.5 | 15.3 | 14.55 | 14.6 | 13.45 | 12.93 |
| 27 | 12 | 10.67 | 13.89 | 11.81 | 12.84 | 12.61 | 14.08 | 9.94 | 12.62 | 11.63 | 10.64 | 11.89 | 10.25 | 9.89 | 10.61 | 11.9 | 11.48 | 12.34 |
| 28 | 10.14 | 9.36 | 10.69 | 12.24 | 10.17 | 10.54 | 8.93 | 10.14 | 11.26 | 10.85 | 10.04 | 9.91 | 9.61 | 8.67 | 9.82 | 10.03 | 11.89 | 12.04 |
| 29 | 16.02 | 15.8 | 17.03 | 16.32 | 16.25 | 16.9 | 13.74 | 15.82 | 15.97 | 16.59 | 14.88 | 14.7 | 14.04 | 13.95 | 14.45 | 15.12 | 16.3 | 16.23 |
| 30 | 12.41 | 12.47 | 12.75 | 12.31 | 13.7 | 12.65 | 11.83 | 10.77 | 13.4 | 12.58 | 10.82 | 12.39 | 11 | 10.94 | 11.77 | 11.61 | 11.63 | 11.15 |
| 31 | 13.5 | 12.97 | 12.84 | 13.72 | 14.21 | 13.36 | 9.63 | 10.55 | 12.41 | 12.28 | 12.36 | 11.08 | 11.31 | 10.91 | 10.77 | 10.83 | 11.91 | 12.24 |
| 32 | 15.13 | 14.98 | 16.83 | 15.51 | 17.43 | 17.47 | 15.48 | 14.76 | 18.5 | 17.18 | 14.12 | 15.33 | 14.1 | 14.61 | 13.93 | 15.28 | 18.03 | 20.72 |
| 33 | 6.87 | 6.42 | 7.1 | 7.01 | 7.24 | 6.49 | 6.09 | 6.51 | 6.61 | 7.88 | 6.81 | 6.5 | 5.29 | 5.38 | 5.98 | 5.9 | 8.47 | 8.28 |
| 34 | 14.74 | 13.82 | 17.27 | 15.1 | 21.2 | 16.19 | 16.12 | 14.27 | 15.01 | 14.19 | 13.31 | 13.15 | 12.58 | 13.38 | 13.76 | 13.04 | 18.69 | 17.21 |
| 35 | 11.81 | 13.5 | 16.84 | 14.14 | 15.23 | 16.35 | 10.96 | 12.04 | 15.79 | 15.47 | 16.06 | 12.99 | 11.92 | 11.52 | 13.66 | 13.54 | 15.3 | 13.98 |
| 36 | 12.97 | 14.28 | 15.35 | 13.95 | 13.22 | 16.3 | 12.97 | 11.58 | 14.44 | 15.53 | 13.56 | 12.87 | 12.4 | 13.43 | 13.05 | 13.86 | 13.74 | 16.02 |
| 37 | 13.83 | 13.14 | 16.19 | 13.65 | 16 | 16.12 | 13.95 | 14.5 | 13.9 | 13.75 | 13.03 | 12.72 | 11.61 | 12.4 | 12.99 | 13.4 | 16.62 | 18.71 |
| 38 | 19.59 | 18.73 | 22.59 | 21.42 | 23.61 | 25.17 | 17.15 | 19.28 | 21.42 | 20.11 | 20.07 | 19.94 | 16.8 | 15.72 | 21.11 | 18.61 | 24.17 | 22.13 |
| 39 | 12.16 | 11.27 | 13.66 | 13.89 | 12.93 | 11.77 | 10.37 | 10.68 | 12.43 | 14.32 | 11.14 | 11.75 | 10.89 | 10.45 | 11.03 | 11.88 | 12.85 | 11.69 |
| 40 | 16.46 | 15.55 | 15.57 | 16.8 | 20.31 | 21.06 | 15.84 | 15.05 | 18.37 | 17.72 | 17.1 | 16.35 | 15.88 | 15.52 | 17.81 | 16.81 | 16.74 | 15.12 |
| 41 | 19.06 | 16.24 | 17.68 | 19.64 | 17.39 | 18.63 | 18.87 | 15.5 | 18.15 | 19.96 | 16.21 | 15.44 | 15.07 | 15.42 | 16.88 | 15.21 | 15.38 | 16.99 |
| 42 | 25.78 | 22.43 | 20.41 | 19.14 | 20.23 | 21.29 | 15.68 | 17.92 | 21.62 | 20.36 | 18.79 | 16.24 | 17.97 | 15.99 | 18.62 | 17.45 | 19.73 | 20.97 |
| 43 | 13.84 | 11.85 | 13.16 | 12.54 | 12.61 | 12.5 | 10.64 | 9.79 | 11.69 | 11.66 | 11.85 | 11.62 | 10.91 | 9.9 | 10.23 | 10.48 | 10.56 | 10.93 |
| 44 | 9.81 | 11.75 | 11.61 | 11.52 | 11.83 | 12.29 | 10.74 | 11.21 | 11.73 | 11.33 | 10.16 | 9.6 | 9.32 | 9.07 | 9.77 | 10.13 | 11.1 | 12.84 |
| 45 | 6.84 | 7.74 | 7.41 | 7.86 | 7.68 | 8.65 | 6.88 | 6.61 | 7.78 | 7.75 | 7.07 | 7.5 | 6.54 | 6.4 | 7.3 | 6.93 | 7.19 | 7.39 |
| 46 | 13.7 | 12.25 | 16.28 | 14.95 | 15.74 | 17.31 | 13.11 | 12.12 | 15.12 | 14.39 | 14.11 | 14.19 | 12.86 | 12.23 | 12.8 | 12.91 | 13.44 | 15.78 |
| 47 | 16.28 | 15.1 | 15.14 | 14.89 | 16.74 | 17.3 | 11.87 | 12.36 | 16.51 | 14.82 | 14.41 | 14.37 | 12.19 | 12.58 | 16.04 | 15.33 | 16.23 | 13.61 |
| 48 | 20.4 | 17.96 | 22.59 | 21.17 | 20.24 | 22.37 | 18.8 | 17.71 | 24.59 | 22.92 | 19.81 | 21.3 | 17.19 | 18.92 | 19.96 | 19.59 | 18.61 | 19.32 |
| 49 | 9.6 | 9.31 | 10.56 | 10.44 | 10.72 | 9.83 | 8.7 | 8.77 | 9.96 | 9.62 | 10.03 | 9.02 | 8.41 | 9.57 | 8.18 | 9.09 | 9.89 | 9.6 |
| 50 | 21.46 | 20.94 | 22.05 | 21.34 | 22.14 | 19.1 | 17.35 | 19.06 | 23.33 | 21.23 | 20.89 | 18.68 | 20.07 | 20.23 | 19.04 | 21.03 | 23.01 | 21.81 |
| 51 | 11.23 | 10.16 | 11.93 | 11.21 | 11.51 | 12 | 9.24 | 10.01 | 11.16 | 11.41 | 10.36 | 10.25 | 10.05 | 9.33 | 9.93 | 10.66 | 10.28 | 11.67 |
| 52 | 15.49 | 16.47 | 16.44 | 16.63 | 18.25 | 17.57 | 15.91 | 15.5 | 15.9 | 15.92 | 13.78 | 15.28 | 13.72 | 13.1 | 16.3 | 16.01 | 18.73 | 16.93 |
| 53 | 20.59 | 20.55 | 22.65 | 20.48 | 20.26 | 21.11 | 16.72 | 18.3 | 22.14 | 24.71 | 21.15 | 21.52 | 16.71 | 17.16 | 20.32 | 19.95 | 16.79 | 17.12 |
| 54 | 13.64 | 13.42 | 16.36 | 14.86 | 16 | 17.8 | 14.01 | 12.63 | 15.26 | 14.11 | 13.33 | 13.62 | 12.49 | 12.07 | 14.6 | 13.63 | 16.59 | 17.9 |
| 55 | 10.33 | 9.28 | 10.97 | 10.43 | 10.47 | 12.43 | 8.38 | 9.77 | 10.06 | 10.45 | 8.6 | 8.6 | 8.59 | 8.82 | 8.78 | 10.14 | 9.87 | 10.51 |
| 56 | 8.95 | 8.49 | 9.65 | 8.46 | 9.1 | 9.07 | 8.05 | 7.81 | 9.61 | 9.11 | 8.39 | 8.65 | 7.29 | 7.09 | 7.89 | 8.54 | 7.2 | 8.48 |
| 57 | 8.59 | 8.84 | 9.94 | 9.78 | 10.28 | 10.21 | 12.08 | 8.96 | 9.95 | 9.14 | 8.86 | 8.45 | 8.22 | 8.42 | 8.5 | 9.15 | 8.78 | 10.43 |
| 58 | 7.74 | 8.4 | 8.81 | 9.68 | 9.09 | 9.49 | 7.94 | 7.93 | 8.44 | 9.47 | 8.62 | 8.15 | 7.24 | 7.24 | 8.15 | 8.52 | 8.25 | 9.47 |
| 59 | 14.31 | 13.6 | 14.64 | 13.77 | 16.25 | 15.45 | 13.11 | 12.56 | 16.32 | 14.11 | 14.07 | 13.4 | 11.81 | 12.15 | 12.88 | 12.73 | 15.35 | 14.78 |
| 60 | 12.19 | 12.32 | 14.5 | 12.13 | 11.77 | 14.07 | 11.02 | 10.2 | 12.89 | 14.96 | 12.74 | 13.52 | 10.85 | 10.89 | 10.78 | 11.39 | 13.17 | 13.35 |
| 61 | 9.41 | 8.22 | 9.1 | 9.09 | 9.67 | 9.05 | 8.41 | 8.73 | 8.83 | 9.45 | 7.92 | 8.15 | 7.54 | 7.97 | 8.06 | 8.11 | 7.35 | 7.7 |
| 62 | 14.78 | 15.05 | 13.86 | 15.14 | 14.95 | 15.68 | 12.87 | 11.66 | 15.05 | 15.7 | 14.43 | 15.24 | 11.62 | 10.73 | 11.69 | 11.94 | 11.61 | 13.38 |
| 63 | 20.01 | 20.39 | 22.91 | 23.58 | 25.08 | 24.47 | 18.82 | 18.02 | 22 | 20.09 | 19.7 | 20.49 | 17.91 | 17.26 | 18.75 | 18.51 | 21.48 | 26.52 |
| 64 | 7.51 | 7.68 | 8.02 | 7.91 | 9.05 | 8.42 | 6.48 | 7.26 | 8.07 | 7.76 | 7.36 | 6.4 | 6.61 | 6.45 | 6.51 | 6.74 | 8.09 | 7.74 |
| 65 | 18.03 | 16.01 | 21.15 | 20.34 | 21.95 | 21.02 | 17.4 | 15.17 | 19.72 | 19.23 | 17.28 | 19.33 | 17.02 | 16.4 | 17.2 | 17.91 | 22.3 | 19.28 |
| 66 | 19.7 | 18.85 | 21.41 | 21.26 | 20.93 | 22.7 | 19.84 | 17.38 | 19.52 | 19.76 | 17.93 | 18.36 | 16.11 | 17.6 | 18.33 | 19.29 | 22.47 | 22.45 |
| 67 | 16.61 | 17.02 | 18.29 | 20.34 | 20.5 | 19.97 | 18.24 | 17.05 | 17.39 | 18.92 | 16.95 | 15.04 | 15.17 | 16.44 | 17.12 | 16.08 | 17.47 | 18.91 |
| 68 | 12.98 | 11.66 | 12.82 | 13.96 | 12.54 | 13.48 | 10.97 | 11.94 | 13.3 | 14.05 | 12.69 | 12.21 | 10.27 | 10.61 | 11.27 | 11.63 | 13.41 | 13.25 |
| 69 | 14.38 | 13.39 | 15 | 14.72 | 16.04 | 14 | 11.93 | 12.99 | 14.19 | 13.57 | 12.27 | 11.11 | 11.29 | 11.5 | 13.1 | 11.94 | 14.75 | 16.52 |
| 70 | 14.17 | 12.6 | 12.8 | 13.23 | 13.3 | 15.8 | 12.83 | 12.33 | 12.81 | 13.05 | 11.85 | 11.69 | 10.33 | 10.96 | 11.16 | 12.49 | 12.77 | 14.08 |
| 71 | 13.75 | 14.23 | 15.16 | 14.26 | 17.03 | 21.35 | 12.75 | 12.27 | 15.78 | 15.57 | 13.6 | 13.35 | 12.44 | 12.88 | 14.39 | 15.01 | 15.01 | 12.95 |
| 72 | 19.92 | 18.03 | 25.86 | 20.08 | 18.2 | 19.33 | 14.27 | 15.4 | 17.42 | 18.02 | 16.87 | 15.95 | 16.09 | 14.48 | 17.09 | 15.96 | 17.9 | 17.06 |
| 73 | 12.68 | 12 | 12.95 | 12.56 | 13.52 | 11.96 | 11.25 | 9.93 | 12.71 | 11.97 | 11.69 | 12.31 | 10.85 | 10.21 | 12.36 | 10.42 | 11.52 | 12.85 |
| 74 | 14.25 | 13.14 | 13.61 | 13.09 | 13.91 | 14.24 | 12.3 | 12.1 | 13.21 | 13.77 | 12.99 | 11.88 | 11.62 | 10.9 | 12.07 | 12.86 | 13.91 | 15.33 |
| 75 | 7.55 | 6.94 | 8.81 | 7.86 | 8.06 | 8.68 | 6.94 | 6.95 | 9.54 | 8.8 | 8.52 | 7.78 | 6.93 | 6.62 | 6.97 | 7.57 | 7.35 | 8.03 |
| 76 | 10 | 9.61 | 10.31 | 10.42 | 11.87 | 11.54 | 9.21 | 9.35 | 10.76 | 10.13 | 8.85 | 9.26 | 9.07 | 8.72 | 9.29 | 9.15 | 9.89 | 9.46 |
| 77 | 20.17 | 15.67 | 19.88 | 20.47 | 19.6 | 21.4 | 16.7 | 15.8 | 19.82 | 23.09 | 19.63 | 21.48 | 14.78 | 16.48 | 18.92 | 19.58 | 20.19 | 17.41 |
| 78 | 11.23 | 11.55 | 11.56 | 12.15 | 12.13 | 12.7 | 11.39 | 10.28 | 11.84 | 13.09 | 10.87 | 11.45 | 8.87 | 9.83 | 9.84 | 11.21 | 11.84 | 13.74 |
| 79 | 15.1 | 14.39 | 15.12 | 15.11 | 16.92 | 16.44 | 14.2 | 12.6 | 15.5 | 14.98 | 13.89 | 13.74 | 11.71 | 13.41 | 13.36 | 14 | 17.13 | 16.08 |
| 80 | 15.3 | 14.02 | 16.87 | 14.54 | 18.51 | 17.58 | 13.36 | 14.04 | 14.69 | 15.37 | 13.18 | 13.25 | 12.13 | 11.67 | 12.89 | 13.06 | 17.05 | 14.22 |

| Region of Interest | Superior parietal gyrus | Superior parietal gyrus | Superior temporal gyrus | Superior temporal gyrus | Supplementary motor area | Supplementary motor area | Supramarginal gyrus | Supramarginal gyrus | Temporal lobe | Temporal lobe | Temporal pole: middle temporal gyrus | Temporal pole: middle temporal gyrus | Temporal pole: superior temporal gyrus | Temporal pole: superior temporal gyrus | Thalamus | Thalamus |
| --- | --- | --- | --- | --- | --- | --- | --- | --- | --- | --- | --- | --- | --- | --- | --- | --- |
| Side | L | R | L | R | L | R | L | R | L | R | L | R | L | R | L | R |
| Number 1 | 9.25 | 9.41 | 9.17 | 8.94 | 8.83 | 10.17 | 9.55 | 8.76 | 9.17 | 9.16 | 6.95 | 6.89 | 7.1 | 7.22 | 9.6 | 10.02 |
| 2 | 11.83 | 12.3 | 12.83 | 11.82 | 12.42 | 14.37 | 10.69 | 11.82 | 12.83 | 12.31 | 9.25 | 9.64 | 9.16 | 9.16 | 15.08 | 14.91 |
| 3 | 14.59 | 15.04 | 13.44 | 12.03 | 16.76 | 13.58 | 12.56 | 12.28 | 13.63 | 13.54 | 8.92 | 9.35 | 9.69 | 10.02 | 14.11 | 13 |
| 4 | 15.63 | 14.71 | 18.03 | 19.61 | 17.26 | 17.02 | 14.2 | 15.29 | 18.03 | 19.61 | 11.46 | 11.23 | 9.88 | 12.28 | 16.88 | 18.29 |
| 5 | 20.42 | 21.07 | 17.46 | 17.85 | 18.38 | 18.18 | 18.29 | 17.97 | 18.31 | 19.24 | 12.06 | 13.54 | 13.63 | 13.78 | 16.43 | 17.09 |
| 6 | 12.12 | 11.68 | 13.17 | 12.41 | 13 | 12.82 | 11.02 | 11.09 | 13.17 | 12.7 | 9.66 | 9.4 | 10.07 | 10.07 | 13.11 | 13.29 |
| 7 | 15.83 | 17 | 16.86 | 16.89 | 18.76 | 18.44 | 17.62 | 15.38 | 16.86 | 18.74 | 12.58 | 13.66 | 12.26 | 14.55 | 18.16 | 17.16 |
| 8 | 10.1 | 9.86 | 11.31 | 10.83 | 14.15 | 13.15 | 11.2 | 9.75 | 11.31 | 11.47 | 8.64 | 8.43 | 9.04 | 9.74 | 10.51 | 10.67 |
| 9 | 16.01 | 15.16 | 18.1 | 15.75 | 17.11 | 18.57 | 15.76 | 16.27 | 19.52 | 19.84 | 11.84 | 12.44 | 13.82 | 13.8 | 16.85 | 16.98 |
| 10 | 13.01 | 11.72 | 12.24 | 11.63 | 12.59 | 12.28 | 12.83 | 12.4 | 13.35 | 13.28 | 9.59 | 10.16 | 9.58 | 9.63 | 12.09 | 12.91 |
| 11 | 19.79 | 23.33 | 22.52 | 20.9 | 24.7 | 21.1 | 19.88 | 20.87 | 22.52 | 22.96 | 13.38 | 16.51 | 14.61 | 16.06 | 25.93 | 25.96 |
| 12 | 14.57 | 13.96 | 16.7 | 15.29 | 16.8 | 17.28 | 14.98 | 16.18 | 16.7 | 15.92 | 11.28 | 11.41 | 12.24 | 12.97 | 16.67 | 16.15 |
| 13 | 17.4 | 16.61 | 16.83 | 18.89 | 20.41 | 19.73 | 16.51 | 19.67 | 16.83 | 20.14 | 11.55 | 12 | 11.32 | 13.49 | 17.95 | 18.78 |
| 14 | 16.42 | 14.89 | 19.13 | 17.66 | 18.58 | 18.6 | 15.42 | 16.01 | 19.61 | 18.22 | 11.19 | 12.66 | 11.92 | 13.35 | 16.67 | 16.99 |
| 15 | 8.79 | 8.14 | 8.07 | 8.14 | 8.66 | 7.82 | 6.68 | 6.65 | 8.07 | 8.14 | 5.08 | 5.83 | 6 | 6.23 | 8.91 | 9.19 |
| 16 | 7.79 | 8.98 | 8.32 | 7.46 | 9.64 | 9.79 | 8.76 | 7.9 | 8.32 | 7.83 | 6.14 | 7.03 | 6.23 | 7.47 | 9.07 | 8.74 |
| 17 | 16.62 | 15.89 | 15.13 | 16.31 | 16.37 | 15.47 | 15.44 | 15.49 | 15.13 | 16.49 | 11.06 | 10.06 | 11.89 | 12.31 | 17.06 | 16.4 |
| 18 | 17.55 | 16.95 | 17.86 | 16.11 | 17.73 | 18.16 | 14.26 | 17.02 | 18.22 | 19.81 | 11.53 | 13.09 | 13.31 | 11.92 | 16.05 | 18.86 |
| 19 | 17.88 | 17.3 | 22.04 | 22.52 | 19.72 | 17.9 | 19.72 | 19.39 | 22.2 | 22.52 | 15.89 | 16.18 | 16.7 | 17.13 | 19.4 | 20.63 |
| 20 | 15.5 | 14.73 | 14.53 | 13.81 | 14.54 | 13.95 | 12.01 | 12.82 | 15.96 | 14.79 | 10.15 | 10.39 | 10.56 | 10.92 | 15.69 | 15.32 |
| 21 | 21.3 | 18.45 | 18.67 | 19.64 | 22.76 | 21.64 | 20.05 | 19.04 | 20.51 | 19.97 | 15.85 | 14.87 | 13.49 | 14.51 | 23.37 | 23.12 |
| 22 | 16.72 | 16.44 | 16.47 | 15.13 | 17.53 | 18.45 | 16.55 | 17.44 | 16.95 | 17.43 | 11.54 | 12.77 | 15.71 | 13.82 | 20.01 | 16.85 |
| 23 | 13.5 | 11.98 | 11.24 | 12.56 | 13.05 | 12.62 | 11.28 | 11.41 | 12.12 | 12.65 | 7.94 | 9.15 | 9.18 | 10.14 | 12.81 | 13.11 |
| 24 | 21.03 | 30.35 | 26.59 | 23.22 | 28.49 | 22.89 | 22.79 | 21.67 | 28.36 | 26.23 | 17.23 | 20.43 | 14.32 | 15.48 | 23.75 | 22.14 |
| 25 | 11.25 | 10.05 | 12.82 | 13.16 | 12.78 | 15.17 | 12.71 | 11.13 | 13.44 | 13.16 | 9.13 | 10.54 | 10.31 | 11.11 | 12.37 | 11.37 |
| 26 | 14.21 | 13.82 | 18.05 | 16.67 | 15.76 | 14.69 | 12.92 | 12.32 | 18.05 | 16.67 | 10.68 | 10.72 | 12.15 | 11.77 | 16.01 | 16.03 |
| 27 | 11.58 | 11.77 | 11.21 | 11.81 | 13.83 | 11.01 | 10.88 | 11.88 | 12.46 | 12.74 | 8.44 | 8.61 | 9.34 | 8.52 | 12.97 | 13.75 |
| 28 | 9.87 | 9.3 | 10.36 | 10.02 | 10.09 | 10.07 | 10.1 | 9.72 | 10.36 | 10.59 | 9.02 | 7.93 | 6.83 | 8.04 | 12.37 | 11.41 |
| 29 | 13.95 | 13.11 | 14.95 | 16.47 | 17.69 | 14.99 | 14.43 | 14.56 | 16.18 | 16.47 | 11.38 | 12.16 | 10.88 | 12.72 | 16.25 | 15.13 |
| 30 | 11.99 | 12.56 | 12.17 | 11.74 | 13.68 | 11.34 | 11.72 | 11.85 | 12.28 | 11.74 | 9.42 | 9.63 | 8.66 | 8.81 | 10.9 | 11.2 |
| 31 | 14.17 | 11.96 | 11.34 | 12.38 | 13.05 | 11.68 | 11.13 | 10.37 | 11.58 | 12.38 | 8.11 | 8.28 | 9.92 | 8.93 | 13.27 | 12.76 |
| 32 | 17.46 | 15.21 | 16.67 | 14.17 | 15.34 | 15.35 | 15.96 | 13.4 | 16.76 | 15.86 | 10.67 | 11.26 | 10.35 | 10.34 | 16.35 | 15.49 |
| 33 | 6.15 | 7.29 | 6.56 | 6.57 | 7.96 | 6.38 | 6.26 | 5.79 | 6.56 | 7.21 | 5.27 | 4.89 | 4.63 | 4.94 | 8.29 | 7.27 |
| 34 | 17.77 | 13.49 | 14.79 | 13.47 | 14.41 | 13.6 | 15.15 | 12.88 | 14.93 | 13.83 | 10.01 | 10.73 | 9.88 | 9.95 | 15.23 | 14.19 |
| 35 | 15.36 | 13.04 | 11.48 | 12.19 | 14.75 | 15.31 | 14.35 | 13.22 | 14.24 | 13.08 | 9.78 | 9.8 | 9.21 | 8.66 | 12.51 | 12.42 |
| 36 | 11.27 | 11.56 | 11.53 | 12.82 | 13 | 12.4 | 12.86 | 12.71 | 12.43 | 13.66 | 9.55 | 11.26 | 11.91 | 9.64 | 12.95 | 12.77 |
| 37 | 13.87 | 12.43 | 14.24 | 13.49 | 13.04 | 11.98 | 12.61 | 12.67 | 14.24 | 13.49 | 10.34 | 9.96 | 9.26 | 9.9 | 14.44 | 15.61 |
| 38 | 20.33 | 17.1 | 19.54 | 19.63 | 22.83 | 21.12 | 17.46 | 18.92 | 19.54 | 19.63 | 14.05 | 14.39 | 15.14 | 14.73 | 19.01 | 18.9 |
| 39 | 12.11 | 12.53 | 13.14 | 11.06 | 12.2 | 12.51 | 11.36 | 10.22 | 13.14 | 11.7 | 8.39 | 9.17 | 9.21 | 8.74 | 11.3 | 12.3 |
| 40 | 14.26 | 15.25 | 15.51 | 15.01 | 19.85 | 17.15 | 15.64 | 15.79 | 18.13 | 16.72 | 11.76 | 11.29 | 11.66 | 12.06 | 16.87 | 17 |
| 41 | 14.55 | 16.4 | 15.87 | 15.21 | 18.27 | 16.33 | 13.73 | 15.32 | 16.57 | 15.97 | 11.17 | 11 | 12.87 | 11.21 | 16.02 | 15.34 |
| 42 | 19.09 | 18.48 | 18 | 17.83 | 18.62 | 17.58 | 16.61 | 17 | 19.28 | 17.83 | 14.29 | 13.12 | 13.62 | 13.08 | 17.49 | 17.87 |
| 43 | 11.81 | 10.72 | 10.04 | 10.3 | 11.31 | 11.49 | 11.29 | 9.97 | 11.21 | 11.07 | 8.54 | 9.13 | 8.64 | 8.28 | 11.6 | 11.79 |
| 44 | 9.35 | 9.91 | 9.92 | 10.57 | 11.51 | 11.16 | 9.56 | 10.79 | 10.46 | 10.84 | 7.66 | 8.21 | 8.35 | 8.12 | 11.23 | 11.16 |
| 45 | 7.21 | 8.63 | 6.61 | 6.95 | 7.63 | 7.39 | 6.45 | 6.42 | 7.05 | 7.05 | 5.7 | 6.03 | 5.49 | 6.53 | 8.26 | 8.47 |
| 46 | 13.98 | 13.53 | 13.63 | 12.77 | 13.96 | 15.39 | 12.07 | 12.23 | 13.95 | 13.36 | 9.32 | 10.03 | 11.14 | 11.28 | 15.07 | 16.01 |
| 47 | 14.15 | 13.96 | 13.72 | 13.3 | 15.96 | 14.16 | 14.91 | 14.22 | 14.07 | 15.21 | 11.36 | 10.74 | 10.61 | 11.49 | 12.52 | 14.11 |
| 48 | 21.03 | 20.31 | 21.19 | 18.87 | 20.96 | 20.72 | 17.74 | 19.75 | 21.19 | 19.03 | 13.22 | 15.47 | 14.34 | 14.7 | 18.41 | 19.24 |
| 49 | 8.48 | 8.68 | 10.38 | 8.93 | 9.83 | 9.46 | 8.93 | 8.31 | 10.42 | 8.94 | 6.89 | 7.06 | 7.44 | 7.36 | 10.67 | 10.38 |
| 50 | 18.99 | 18.05 | 19.23 | 18.45 | 21.55 | 18.81 | 19.35 | 18.89 | 19.5 | 20 | 14.32 | 15.07 | 13.97 | 14.46 | 19.27 | 20.91 |
| 51 | 9.84 | 10.23 | 11.11 | 9.97 | 11.12 | 12.18 | 9.36 | 10.21 | 11.32 | 10.91 | 6.86 | 7.15 | 8.14 | 8.12 | 11.11 | 10.83 |
| 52 | 14.72 | 14.29 | 14.17 | 15.34 | 16.03 | 14.87 | 14.59 | 13.8 | 15.49 | 15.34 | 11.27 | 10.38 | 10.53 | 10.94 | 15.95 | 16.94 |
| 53 | 20.89 | 16.11 | 19.07 | 17.54 | 21.88 | 19.34 | 18.61 | 17.59 | 19.07 | 19.3 | 13.82 | 13.37 | 14.13 | 13.56 | 19.12 | 20.08 |
| 54 | 14.6 | 13.98 | 14.02 | 12.73 | 13.42 | 13.66 | 13.31 | 13.51 | 14.1 | 15.04 | 9.95 | 10.38 | 11.82 | 10.21 | 14.99 | 14.27 |
| 55 | 9.74 | 9.68 | 9.54 | 8.9 | 9.83 | 10.2 | 9.01 | 9.49 | 9.54 | 9.8 | 6.95 | 7.81 | 7 | 7.14 | 10.65 | 9.94 |
| 56 | 7.33 | 6.87 | 6.97 | 7.64 | 8.66 | 8.52 | 6.99 | 7.1 | 7.7 | 8.12 | 5.99 | 6.93 | 5.78 | 5.4 | 8.09 | 7.59 |
| 57 | 8.59 | 8.99 | 10.81 | 9.5 | 10.37 | 8.78 | 9.83 | 8.72 | 12.48 | 10.22 | 7.28 | 8.26 | 7.48 | 7.64 | 10.72 | 9.85 |
| 58 | 7.9 | 8.66 | 7.95 | 7.76 | 9.54 | 8.44 | 7.69 | 7.57 | 7.95 | 8.73 | 5.97 | 6.43 | 6.32 | 6.82 | 9.08 | 8.57 |
| 59 | 14.38 | 15.22 | 15.48 | 12.94 | 14.47 | 13.71 | 12.76 | 11.96 | 15.48 | 14.72 | 9.59 | 10.98 | 9.96 | 10.41 | 15.42 | 14.46 |
| 60 | 11.37 | 11.32 | 13.8 | 11.48 | 14.19 | 13.38 | 11.07 | 11.56 | 13.8 | 12.45 | 9.09 | 9.15 | 9.41 | 8.99 | 13.63 | 12.76 |
| 61 | 8.46 | 8.21 | 9.36 | 8.76 | 9.84 | 8.5 | 7.27 | 8.17 | 9.36 | 8.91 | 5.62 | 6.25 | 7.12 | 6.08 | 8.58 | 8.01 |
| 62 | 12.22 | 12.11 | 14.48 | 15.11 | 13.2 | 14.32 | 11.32 | 12.7 | 14.48 | 15.11 | 8.87 | 9.55 | 8.85 | 10.24 | 13.86 | 12.98 |
| 63 | 21.54 | 18.68 | 17.72 | 21.51 | 19.95 | 19.17 | 18.41 | 19.01 | 18.49 | 21.51 | 12.96 | 15.29 | 14.89 | 15.14 | 21.11 | 21.07 |
| 64 | 6.89 | 6.6 | 7.2 | 7.47 | 7.58 | 7.51 | 6.96 | 7.12 | 7.32 | 8.31 | 5.67 | 5.94 | 5.75 | 5.72 | 7.47 | 7.21 |
| 65 | 18.36 | 17.19 | 18.17 | 18.44 | 18.95 | 19.33 | 16.12 | 16.83 | 18.17 | 18.44 | 12.53 | 13.51 | 13.78 | 12.38 | 20.45 | 21.55 |
| 66 | 18.38 | 18.91 | 17.46 | 18.01 | 18.86 | 17.46 | 18.74 | 19.09 | 17.46 | 19.04 | 13.05 | 13.57 | 12.87 | 13.04 | 19.86 | 21.38 |
| 67 | 16.18 | 15.58 | 18.06 | 17.01 | 17.12 | 18.28 | 16.07 | 14.81 | 18.06 | 17.01 | 11.87 | 12.92 | 12.11 | 10.63 | 19.26 | 18.23 |
| 68 | 12.14 | 12.52 | 11.5 | 11.52 | 13.43 | 13.5 | 10.22 | 11.66 | 11.5 | 11.52 | 8.53 | 9.57 | 9.04 | 9.36 | 14.41 | 13.98 |
| 69 | 12.65 | 12.27 | 13.55 | 12.97 | 14.02 | 12.76 | 11.99 | 12.59 | 13.55 | 13.99 | 9.15 | 9.14 | 10.28 | 10.47 | 13.02 | 12.26 |
| 70 | 12.53 | 11.52 | 13.35 | 13.55 | 12.07 | 12.7 | 12.66 | 12.92 | 13.35 | 13.95 | 9.02 | 10.03 | 10.22 | 8.65 | 11.68 | 12.77 |
| 71 | 13.81 | 14.14 | 15.22 | 13.73 | 16.08 | 12.22 | 13.51 | 15.34 | 15.22 | 15.22 | 10.67 | 11.31 | 10.89 | 10.58 | 14.93 | 15.37 |
| 72 | 18.06 | 19.45 | 14.85 | 16.75 | 21.84 | 18.03 | 16.46 | 15.52 | 17.55 | 17.6 | 13.17 | 12.43 | 14.55 | 12.35 | 16.86 | 18.99 |
| 73 | 11.71 | 12.1 | 12.15 | 12.31 | 12.39 | 11.86 | 10.68 | 11.34 | 12.15 | 12.31 | 8.49 | 8.62 | 9.12 | 8.6 | 14.96 | 14.07 |
| 74 | 12.01 | 11.61 | 11.48 | 13.11 | 14.76 | 11.86 | 12.09 | 12.12 | 12.22 | 13.11 | 8.72 | 9.51 | 9.46 | 9.79 | 13.12 | 13.39 |
| 75 | 7.76 | 7.34 | 7.6 | 7.18 | 8.72 | 8.15 | 7.2 | 7.56 | 7.6 | 7.85 | 5.55 | 5.7 | 5.88 | 6.11 | 7.86 | 8.37 |
| 76 | 9.1 | 9.31 | 10.62 | 9.9 | 9.56 | 10.74 | 9.09 | 9.35 | 10.62 | 11.19 | 6.56 | 7.08 | 6.85 | 6.48 | 10.52 | 10.82 |
| 77 | 16.76 | 16.92 | 18.38 | 17.58 | 19.94 | 22.22 | 19.99 | 19.33 | 19.18 | 18.18 | 12.83 | 14.14 | 14.08 | 14.74 | 20.03 | 18.34 |
| 78 | 10.39 | 9.97 | 12.04 | 10.89 | 11.29 | 13.08 | 10.28 | 11.26 | 12.86 | 12.1 | 7.81 | 9.01 | 7.99 | 9.09 | 11.87 | 11.87 |
| 79 | 13.07 | 12.83 | 15.6 | 13.31 | 15.07 | 14.49 | 13.88 | 13.49 | 15.6 | 14.33 | 10.43 | 10.65 | 10.69 | 11.01 | 13.66 | 14.9 |
| 80 | 16.9 | 13.38 | 14.54 | 14.34 | 13.24 | 13.87 | 12.54 | 14.13 | 14.76 | 14.34 | 11 | 11.59 | 10.73 | 12.15 | 13.25 | 13.49 |

Abbreviation: SUV_max_: maximum standardized uptake value; AAL: automated anatomical labelling.

Supplementary Table11 The SUV_meanstd_ of normal 45-70 years old group according to AAL standard.

| Region of Interest | Amygdala | | Amygdala | Angular gyrus | Angular gyrus | Anterior cingulate and paracingulate gyri | Anterior cingulate and paracingulate gyri | Basal ganglia | Basal ganglia | Calcarine fissure and surrounding cortex | Calcarine fissure and surrounding cortex | Caudate nucleus | Caudate nucleus | Central region | Central region | Cingulate and paracingulate gyri | Cingulate and paracingulate gyri | Cuneus | Cuneus |
| --- | --- | --- | --- | --- | --- | --- | --- | --- | --- | --- | --- | --- | --- | --- | --- | --- | --- | --- | --- |
| Side | L | R | | L | R | L | R | L | R | L | R | L | R | L | R | L | R | L | R |
| Number 1 | -0.4 | 0.4 | | -1.7 | -0.1 | 0.5 | 0.3 | 0.2 | 0 | 1.9 | 3.3 | 0 | -0.1 | 0 | -1.2 | 1.8 | 1.5 | 1.3 | 0.6 |
| 2 | -1.2 | -0.1 | | -2.4 | -0.8 | -0.2 | 0.5 | -0.5 | -0.2 | 2.3 | 2.1 | -0.8 | -0.1 | -0.7 | -1.5 | 0.7 | 0.5 | 4.1 | 3.6 |
| 3 | -1.3 | -2.3 | | -0.7 | -0.3 | -0.1 | -0.7 | 0.2 | -0.5 | 2.2 | 2.7 | 0.1 | -0.1 | -0.3 | -3.3 | 0.1 | -0.7 | 3.1 | 2.1 |
| 4 | -1.7 | -1 | | -2.1 | -1 | -1.5 | -1.6 | -0.3 | -0.3 | 2.2 | 1.9 | -1.6 | -1.3 | -0.6 | 0 | -1.5 | -1.8 | 2.5 | 1.4 |
| 5 | -1.2 | -3.4 | | -0.9 | -0.1 | -0.5 | -1.2 | -1.2 | -0.9 | -1.3 | 0.1 | -1.4 | -0.7 | -0.4 | -1.6 | -0.3 | 0 | 1.1 | -0.8 |
| 6 | 0.7 | 1.1 | | -1.9 | 0.3 | 0.5 | 1.3 | -0.9 | -0.7 | 0.7 | -0.3 | -1.1 | -0.7 | -0.1 | -0.1 | 1.5 | 2.1 | 1.8 | 1 |
| 7 | -1.6 | -2.4 | | -1 | 0.1 | 0.4 | -0.3 | -1.3 | -0.7 | 1 | -0.6 | -1.2 | -0.5 | -1 | -1.3 | -0.3 | -0.1 | -0.1 | 0.2 |
| 8 | -0.5 | -1.3 | | 0.2 | -0.8 | 0.3 | 0.1 | 1 | 0.2 | 1.6 | 2 | -0.2 | -0.6 | -0.1 | -1.5 | 0.8 | 0.2 | 0.7 | 0.8 |
| 9 | -1.6 | -1.2 | | -1.2 | -2.1 | 0 | -0.1 | 0.3 | 0.5 | 1.7 | 1.2 | -0.6 | 0 | -0.8 | -1 | 0.5 | 0.1 | 0 | 0.3 |
| 10 | 0.7 | 0 | | -1.1 | 0.1 | -0.1 | 0.1 | -0.4 | -0.1 | 1.9 | 3.2 | -0.2 | 0 | -1.4 | -2.7 | 0.8 | 0.6 | 0.6 | 0.6 |
| 11 | -0.6 | -1.3 | | -1.4 | -0.4 | 0 | 0.1 | 0.3 | -0.7 | 1.8 | 4.1 | -0.6 | -1.1 | -3.7 | -1.7 | 0.2 | 1 | 2.9 | 0.7 |
| 12 | -2.3 | -1.5 | | -2.5 | -1.9 | -0.6 | 0 | 0.1 | -0.2 | 0 | 1.1 | -0.4 | -0.1 | 0.1 | 0 | 0.4 | 0.8 | 1.4 | 2 |
| 13 | -2 | -3.5 | | -2.2 | -0.6 | -1.3 | -1.3 | -2.8 | -2.1 | 0.3 | 2.4 | -2.1 | -1.2 | -1.8 | -2 | 0 | -0.4 | -0.5 | -0.1 |
| 14 | -2 | -1.8 | | -1.4 | -1.4 | -1.2 | -1.5 | -0.5 | 0.3 | 1.9 | 2.2 | -0.5 | 0.1 | 0 | 1.3 | -0.2 | 0.1 | 1.1 | 2.8 |
| 15 | -0.2 | 0.1 | | -2.2 | -1.3 | -0.3 | -0.4 | -1.6 | -0.5 | 1.5 | 2.6 | -2.3 | -1.2 | -1.3 | -1.8 | -0.1 | -0.5 | 2.2 | 1.7 |
| 16 | 3 | -0.4 | | -0.3 | -0.8 | 0.7 | 0.4 | 0.2 | -0.6 | 0 | 0.8 | -0.4 | -1 | 1.6 | 0.2 | 1.2 | 0.8 | 0.8 | 1.3 |
| 17 | -1.7 | -1.6 | | -1.7 | 0.5 | -0.7 | -0.8 | -2.3 | -2.3 | 0.5 | 1.9 | -2.6 | -2.1 | -0.7 | -1.9 | -1.1 | -1.6 | -1.2 | -0.1 |
| 18 | -1.9 | -2.5 | | -1.5 | 0.7 | 1 | 0.7 | 1.9 | 1.7 | 1.2 | 1.7 | 0.8 | 0.9 | -1.2 | -1.3 | 1.5 | 1.1 | -0.9 | 1.4 |
| 19 | -3 | -3.1 | | -0.9 | 0.5 | 0.1 | 0.4 | -0.2 | -0.1 | 2.2 | 2.7 | -0.5 | -0.2 | -4 | -4 | 0.3 | 0.4 | 0.3 | 2.3 |
| 20 | -2 | -2.4 | | -1.4 | -1.8 | 0.4 | 0.5 | 0.1 | 0.2 | 0.5 | 3.4 | -0.7 | -0.4 | -0.3 | -0.5 | 0.9 | 0.3 | 1 | 2.1 |
| 21 | -1.1 | -1.5 | | -0.1 | -0.7 | -0.3 | -0.8 | 1.2 | 0.8 | 1.5 | 2.5 | -0.3 | -0.3 | -1.2 | -1.4 | 1.5 | 0.7 | -0.6 | 2.6 |
| 22 | -1.9 | -2.4 | | -0.4 | 0.3 | 0.8 | 1.1 | -0.6 | -1 | 1 | 1.9 | -0.4 | -0.4 | -0.6 | -2.3 | 1.5 | 0.9 | -0.9 | 1.1 |
| 23 | 0.8 | 0.1 | | -2.8 | -0.6 | 1 | 0.4 | -0.6 | -1.2 | 2.4 | 1.8 | -0.9 | -1.1 | -0.6 | -1.7 | 1.5 | 0.8 | 2.5 | 2.4 |
| 24 | 1.1 | -3.3 | | -2.2 | -0.2 | 1.1 | 2.3 | -0.2 | 0.8 | 0.5 | 1.6 | 0 | -0.3 | -0.7 | -2.3 | 1.6 | 1.1 | 0.9 | 1.5 |
| 25 | 0.2 | -0.1 | | -1.4 | -0.8 | 1.1 | 1 | 2.1 | 1.5 | -1.6 | -1.9 | 1 | 0.6 | 1.1 | -0.2 | 1.8 | 0.8 | 0.5 | -0.7 |
| 26 | -1.5 | -1.5 | | -0.5 | 0.2 | 0.3 | 0.4 | -0.1 | 0.1 | -0.1 | 1.6 | 0.1 | -0.2 | 0.1 | -0.9 | 0.4 | 0.5 | -0.1 | 0.9 |
| 27 | 0.5 | -0.9 | | -2 | -2 | 0.1 | -0.2 | -0.4 | 0 | 2.6 | 2.4 | -0.8 | -0.2 | -0.1 | -2.4 | 0.4 | -0.1 | 0.2 | 3.7 |
| 28 | 0.1 | 0.6 | | -1.2 | -1.3 | -0.1 | 0.7 | 0.8 | 0.8 | 1.7 | 3.4 | 0.1 | 0.1 | -1.5 | -1.5 | 0.1 | 0.5 | 2.3 | 2.7 |
| 29 | -1.6 | -3.3 | | -1.2 | -1.3 | -1.3 | -1.5 | -1.3 | -1.7 | 0.7 | 4.2 | -2.2 | -2.2 | -1.1 | -0.3 | -2.2 | -2.2 | 2.5 | 3.4 |
| 30 | 0.9 | -0.5 | | -0.5 | -0.6 | 0.5 | 0.1 | 0.3 | 1 | 0.9 | 1.8 | 0.1 | 0.8 | -1.5 | -1.9 | 0.5 | 0.4 | 1.1 | 2.8 |
| 31 | -0.4 | -0.4 | | -0.1 | -0.3 | 0.8 | 0.4 | -0.4 | -0.4 | 1.7 | 1.9 | -0.3 | 0.1 | -1 | -2.8 | 0.9 | 1.1 | 1.4 | 0.8 |
| 32 | -0.8 | -1.3 | | -0.3 | -0.9 | 0.2 | 0.1 | 0.1 | 0.2 | -0.4 | 2.3 | -0.2 | -0.2 | 0.1 | -2.4 | -0.7 | -0.7 | 2.5 | 1.7 |
| 33 | 1.8 | 0.6 | | -2 | -1.4 | -0.9 | -1.2 | -0.4 | -0.5 | 1.8 | 5.1 | -1.1 | -1.1 | 0.5 | 0 | -0.8 | -0.8 | 2 | 1.9 |
| 34 | -0.8 | -0.7 | | -1.3 | -1.1 | -0.7 | -1.5 | 0 | 0 | 1.8 | -0.1 | -0.3 | -0.1 | -0.5 | -1.3 | -0.1 | -1 | 3.5 | 3.1 |
| 35 | -1.3 | -2.5 | | -1.2 | 0.4 | 0.7 | 0.2 | -1.1 | -0.7 | 1.8 | 1.3 | -1.3 | -0.6 | -1 | -4.1 | -0.6 | -0.7 | 0.1 | 0.2 |
| 36 | -0.3 | -0.7 | | 0.2 | -0.1 | 0.6 | 0.8 | 0.2 | -0.1 | 1.3 | 0.4 | -0.7 | -0.4 | -0.8 | -1.5 | 1.3 | 1.4 | 0.2 | 1.8 |
| 37 | -1.8 | -1.6 | | 0 | -0.1 | -1.1 | -1.5 | -0.7 | -0.6 | 1.1 | 3.5 | -0.7 | -0.5 | -1.1 | -2 | -1.3 | -1.7 | 2.3 | 4 |
| 38 | -2 | -3.3 | | -0.5 | 1.6 | -0.5 | -0.6 | -0.1 | 0.1 | 2.2 | 1.6 | -0.8 | -0.3 | -1.3 | -2.3 | -0.6 | 0 | -0.2 | 1 |
| 39 | -0.4 | -0.2 | | -2.1 | -1 | 0.8 | 0.4 | 0 | 0.2 | 0.6 | 1.6 | -0.7 | 0 | 0.9 | -0.2 | 0.3 | 0.1 | 1.8 | 2.1 |
| 40 | -0.5 | -1 | | 0.5 | -0.3 | 0.8 | 0.3 | -0.3 | -0.2 | 0.5 | 0.2 | -0.8 | -0.4 | -1.5 | -1.5 | 1.5 | 1.5 | -0.6 | 1.2 |
| 41 | -1.7 | -3.1 | | -2.3 | -0.9 | 0.5 | 0.3 | -2.1 | -1.4 | 1.2 | 2 | -1.6 | -0.8 | 0.3 | -1.4 | 1.4 | 0.9 | -0.5 | 0.1 |
| 42 | -0.4 | -0.7 | | 0.1 | 0.5 | 0.5 | 0.5 | -1.3 | -1.1 | 1.2 | 1.2 | -0.9 | -0.6 | -1.4 | -4.1 | 1.3 | 0.7 | 2.4 | 2.9 |
| 43 | 0.4 | 0.8 | | -1.4 | -0.7 | 1.4 | 1.5 | -0.3 | -0.3 | 3.2 | 2.1 | -0.4 | -0.3 | -1.1 | -2.8 | 1.9 | 1.6 | 1.8 | 2.4 |
| 44 | 0.3 | -0.1 | | -0.5 | -0.2 | -0.4 | -0.9 | -0.2 | -0.6 | 0.2 | 1.2 | -1.2 | -1.2 | -1.3 | -1.7 | -0.4 | -1 | 0.1 | 1.3 |
| 45 | 2.6 | 1.5 | | -0.1 | -0.1 | 0.6 | 0.6 | 1.2 | 1.2 | 0.3 | 0.9 | 0.4 | 0.4 | 0.5 | 0.2 | 1.1 | 1.1 | 0.6 | 1.1 |
| 46 | -1.7 | -2 | | -1.7 | -0.6 | -1 | -1 | 0.1 | -0.8 | 0.1 | 0.8 | -0.8 | -1.1 | -0.9 | -0.7 | -0.7 | -0.5 | 0.9 | 1.3 |
| 47 | -0.8 | -0.8 | | -0.2 | 0.4 | 0.4 | 0.5 | -0.2 | -0.1 | 1.7 | 2.7 | -0.3 | 0 | -0.9 | -2.1 | 0.9 | 0.9 | -0.5 | 0.9 |
| 48 | -0.8 | -2 | | -2.3 | -0.5 | -1 | -0.2 | -0.5 | -0.7 | 0.6 | 2.8 | -0.9 | -1.1 | -1.8 | -1.2 | -0.2 | 0.3 | 0 | 1.4 |
| 49 | 1.1 | -0.1 | | -1.7 | -1 | 0.7 | 0.8 | 0.2 | -0.2 | -0.7 | 1.4 | 0.3 | -0.1 | -0.3 | -3.1 | 1.1 | 1.2 | -0.9 | 2.5 |
| 50 | -3 | -3.9 | | -1.1 | -0.2 | 0.9 | 1.2 | 0.1 | 0.2 | 1.2 | 4.2 | 0.1 | -0.1 | -1.7 | -2.9 | 0.6 | 0.6 | 0.3 | 3.6 |
| 51 | 0.5 | -1.8 | | 0.3 | 0.8 | -1.5 | -1.1 | 1.8 | 1.3 | 0.2 | 0.8 | -1.3 | -0.8 | 0.9 | -0.2 | -1 | -0.9 | 0.8 | -0.2 |
| 52 | -1.8 | -2 | | -0.3 | 0.5 | -0.6 | -0.4 | -0.1 | 0.4 | 0.6 | 1.7 | -0.4 | 0 | -0.6 | -2.8 | 0.3 | 0.7 | -2.1 | 2.6 |
| 53 | -4.4 | -3.7 | | -0.8 | 0.3 | -1.3 | -1 | -1.9 | -1.6 | 1.2 | 1.8 | -2.3 | -3.5 | -0.5 | 0.6 | 0.5 | -0.8 | 0.9 | -1.9 |
| 54 | -1.2 | -0.9 | | -2.1 | 0.2 | 0.5 | 0.3 | 1.3 | 0.8 | 1 | 0.4 | 0.3 | 0.5 | -0.2 | -1 | 0.9 | 0.4 | -1 | -0.8 |
| 55 | 0.6 | 0.5 | | -1.8 | -1.1 | 0.1 | -0.5 | 1.1 | 0.4 | 1.2 | 2.1 | 1.4 | 0.6 | 0.7 | -1.4 | 0.1 | 0.3 | 1.1 | 0.8 |
| 56 | 0.7 | 1 | | -0.7 | -0.3 | 0.3 | 0.7 | 0.7 | 0.3 | 1 | -0.1 | -0.6 | -0.8 | -0.6 | -0.7 | 0.8 | 0.9 | 0.6 | 1.5 |
| 57 | 1.9 | 1.5 | | -0.1 | 0.1 | 1.4 | 1.2 | 1.5 | 0.9 | 0.8 | 1.6 | 0.7 | 0.6 | 1 | -0.3 | 0.8 | 0.3 | 0.8 | 1.8 |
| 58 | 1.1 | 0.1 | | -2.1 | -1.7 | 0.9 | 1.3 | 1.2 | 1.2 | 1.4 | 1.9 | 0.3 | 0.5 | 0.7 | 0.9 | 1.6 | 2.2 | 0 | 0.9 |
| 59 | -1.2 | -1 | | 0.7 | 0.7 | 0.1 | 0.1 | -0.4 | 0.1 | 1 | 2.9 | -1.8 | -0.7 | 0.4 | -1.9 | 0.2 | -0.2 | 0.6 | 2.5 |
| 60 | -1 | -0.6 | | -2.4 | -0.6 | -0.5 | 0.4 | -0.1 | -0.6 | 0.8 | 2.2 | -1 | -0.9 | 0.3 | -1.2 | 0 | 1.2 | -0.7 | 3 |
| 61 | 0.4 | 1.5 | | -1.6 | 0.1 | 0.8 | 1.3 | 2 | 1.7 | -0.5 | 0.3 | 1.1 | 0.9 | 0.2 | 1.1 | 1.5 | 1.8 | 0.1 | -0.4 |
| 62 | 1.3 | 0.2 | | -1 | -1 | -0.7 | -1.1 | 0.7 | 0.2 | 1.7 | 2.3 | -1.1 | -0.9 | -0.1 | 1 | 0.8 | 0.8 | 3.1 | 2.8 |
| 63 | -1.6 | -1.4 | | -1.9 | 0.4 | 0.4 | 0.3 | -1 | -0.4 | 1 | 2.8 | -0.1 | 0.2 | -1.6 | -2.5 | -0.4 | 1.1 | -0.4 | 0.5 |
| 64 | 1.6 | 1.3 | | 0.8 | 0.3 | -0.9 | -1.2 | 1 | 1.1 | 1.9 | 2.5 | -0.2 | 0.2 | -1 | -1.2 | 0.1 | -0.3 | 3.6 | 1.7 |
| 65 | -0.5 | -1.4 | | -2.2 | -1.2 | 0.1 | -0.4 | 0.7 | 0.6 | 0.2 | 3 | 0.1 | 0.1 | 0.1 | -1.1 | 0.4 | -0.8 | 1.2 | 3 |
| 66 | -2.1 | -2.4 | | 0 | 0.2 | 0.1 | 0.1 | -0.6 | -0.4 | 1.3 | 2.4 | -1 | -0.3 | -1 | -1.7 | 0.2 | 0.9 | 0.4 | 2.8 |
| 67 | -1.6 | -1.6 | | -2.9 | -1.1 | 0.8 | 0.6 | -1.2 | -0.9 | 1.5 | 2.9 | -0.9 | -0.3 | -1.4 | -2.5 | 1 | 0.7 | -0.4 | 0.9 |
| 68 | 0 | -0.5 | | -1.3 | -0.6 | 0.1 | 0.7 | 0.3 | 0.3 | 0.5 | 1.7 | 0 | -0.1 | 0.7 | 0.9 | 0.6 | 1.1 | 0 | 0.8 |
| 69 | -1.4 | -3.1 | | -1.4 | -1 | 0 | 0 | -0.2 | -0.1 | -1.2 | 1.1 | -0.7 | -0.1 | -0.3 | 0.1 | 0 | 0.1 | 1.9 | 2.8 |
| 70 | -0.2 | -0.6 | | -0.8 | -0.8 | 1 | 0.7 | 1.5 | 0.9 | 0.4 | 2.4 | 0.6 | 0.5 | 0.9 | 0.9 | 0.8 | 1.2 | 1 | 2.7 |
| 71 | -0.8 | -1.8 | | 2.2 | 0.8 | 0.7 | -0.3 | 0.4 | 0.5 | -0.4 | 1.3 | 0.2 | 0.4 | -0.7 | -1.7 | 0.7 | 0.2 | 2.8 | 2.8 |
| 72 | -0.4 | -2.8 | | -2.6 | -1 | -1 | -0.7 | -1.6 | -1.6 | 2.1 | 3.9 | -2 | -1.5 | -0.7 | -2 | -0.2 | -0.8 | 0.5 | -0.5 |
| 73 | -0.9 | -0.8 | | 0.7 | 1.4 | 0.8 | 0.7 | 1.2 | 1 | 1.2 | 1.5 | 0.7 | 0.6 | -0.1 | -1.6 | 1 | 0.8 | 0.4 | 2.2 |
| 74 | -0.3 | -2.1 | | 1.4 | 1.1 | 1.5 | 2.1 | -0.7 | -0.9 | 2.3 | 2.4 | -0.8 | -0.3 | -0.3 | -1.2 | 1.4 | 1.4 | 1.9 | 2.4 |
| 75 | 1.1 | 0.9 | | -1.7 | 0.3 | 0.5 | 0.1 | -0.1 | 0 | 2.5 | 2.9 | 0 | 0 | -0.2 | -0.1 | 0.3 | 0.3 | 0.5 | 2.6 |
| 76 | 1 | 0.9 | | -0.3 | -0.1 | 1.2 | 1.1 | 0.2 | 0.9 | 1 | 1.6 | 0.1 | 0.7 | -0.9 | -0.7 | 1.6 | 0.9 | 1.6 | 2 |
| 77 | -2.1 | -2.3 | | 0.4 | 0.9 | 1.3 | 2 | -0.6 | -0.3 | 0.9 | 1.7 | -0.9 | -0.1 | -1.5 | -1.9 | 1.9 | 2.7 | 3.1 | 3.4 |
| 78 | -1.3 | -0.5 | | -0.4 | -0.2 | 0 | -0.6 | 2.3 | 1.9 | 0.6 | 1.9 | 1.3 | 0.6 | -0.9 | -0.6 | 0.9 | 0.3 | 2.9 | 3.7 |
| 79 | -0.7 | -2.1 | | 0 | 0 | 1.3 | 1.8 | 0.8 | 0.6 | 1 | 2.8 | 0.4 | 0.4 | -0.8 | -1.5 | 2 | 2.2 | 0.1 | -0.9 |
| 80 | -1.3 | -0.9 | | -2 | -0.8 | -0.1 | 0.4 | -0.5 | 0.2 | 0 | 0.3 | -0.6 | 0 | -0.2 | -1 | 0.7 | 1.6 | -0.6 | 0.5 |

| Region of Interest | Frontal lobe | Frontal lobe | Fusiform gyrus | Fusiform gyrus | Gyrus rectus | Gyrus rectus | Heschl gyrus | Heschl gyrus | Hippocampus | Hippocampus | Inferior frontal gyrus, opercular part | Inferior frontal gyrus, opercular part | Inferior frontal gyrus, orbital part | Inferior frontal gyrus, orbital part | Inferior frontal gyrus, triangular part | Inferior frontal gyrus, triangular part | Inferior occipital gyrus | Inferior occipital gyrus |
| --- | --- | --- | --- | --- | --- | --- | --- | --- | --- | --- | --- | --- | --- | --- | --- | --- | --- | --- |
| Side | L | R | L | R | L | R | L | R | L | R | L | R | L | R | L | R | L | R |
| Number 1 | -2.9 | -3.7 | 0.8 | 1.4 | -1.6 | -0.9 | -0.8 | 0 | 1.5 | 1.4 | -1.4 | -2.6 | -2.6 | -3.3 | -4 | -3.6 | 0.4 | -0.6 |
| 2 | -2.8 | -3.4 | 0.7 | 1.2 | -1.4 | -1.1 | 0.3 | 0.3 | 0 | 0.6 | -1.2 | -1.5 | -1.4 | -3.5 | -2.4 | -2.1 | 0.4 | -0.2 |
| 3 | -3.4 | -5.6 | 2.4 | 0 | -2.4 | -3.6 | -0.4 | -1.1 | 0.8 | -0.9 | -1.9 | -3.7 | -2.8 | -3.8 | -2.9 | -3.9 | 3.2 | 1 |
| 4 | -4.9 | -4.4 | -0.3 | 1.3 | -2.5 | -1.2 | 1.2 | 2.2 | 0.7 | 0.4 | -1.4 | -0.7 | -3.2 | -2.7 | -2.7 | -1.6 | 0.5 | 0.1 |
| 5 | -1.9 | -2.8 | 1.1 | 0.3 | -1.9 | -1.4 | 0.1 | -0.6 | -1.4 | -2 | 0.5 | -2.5 | -1.1 | -1.2 | -1.6 | -2.8 | 0.7 | 0.7 |
| 6 | -0.5 | 0.2 | 0.1 | 1.3 | -1.1 | -0.8 | -1.6 | -0.3 | -0.5 | 0.7 | 0.2 | 0.8 | -1.9 | -2.1 | -2 | -1.2 | -1.1 | -2.9 |
| 7 | -1.6 | -2.7 | 2 | 2.3 | -1.1 | -0.2 | 0 | 0.4 | -1.1 | -1 | -1.3 | -2.2 | -0.6 | -1.4 | -0.8 | -1.1 | 2.7 | 4.7 |
| 8 | 0.4 | -0.5 | 0 | 0.7 | -1.5 | -0.4 | -1.3 | 0.6 | -0.3 | -1.2 | -0.7 | 0.6 | -0.4 | -0.1 | -1.5 | -1.5 | -0.8 | -1.2 |
| 9 | -0.5 | -1.9 | -0.1 | -1.1 | -0.8 | -0.7 | 2.5 | 1.7 | 0.8 | -0.6 | -0.7 | 0 | 0.3 | -0.4 | -1.4 | -1.7 | -0.8 | -2.6 |
| 10 | -2.6 | -3.4 | 1.5 | 1.3 | -1.7 | -0.7 | 2 | 1.4 | 0.2 | 0.8 | -0.6 | -1.2 | -0.9 | -2 | -2.8 | -2 | 0.6 | -1.3 |
| 11 | -3.4 | -2.9 | 0.6 | 1.4 | -2.8 | 0.2 | -0.6 | 0.2 | -0.9 | -0.9 | -2 | -1.5 | -6.1 | -4.8 | -3.6 | -2.7 | 0.2 | 3.5 |
| 12 | -1.5 | -1.4 | 0.9 | -1.2 | -2 | -0.3 | 2.1 | 0.2 | -0.8 | -0.4 | 1.5 | -0.8 | -2.8 | -2.6 | -2 | -1.1 | 1 | 0 |
| 13 | -2.1 | -1.7 | 1.1 | 1 | -3.2 | -2.6 | 0 | 0.6 | -1.1 | -1.7 | -1.9 | -1.6 | -3.6 | -3.7 | -0.8 | -0.6 | 2.3 | 3 |
| 14 | -4.1 | -3.8 | -0.9 | 0 | -3.4 | -3.4 | 2 | 1.4 | -1.1 | -2 | -2.6 | -1.5 | -3.2 | -1.1 | -2 | -2.3 | 1.8 | 0.7 |
| 15 | -4.6 | -4.9 | 0.7 | 1 | -4.4 | -3.2 | -4.3 | -4 | 0.3 | -0.4 | -3.4 | -3.3 | -3.6 | -3 | -4.8 | -3.1 | -0.4 | -2.1 |
| 16 | 1.2 | -1.5 | 0.1 | 0.6 | -0.3 | -0.9 | 1.1 | 0 | 2.3 | 0.8 | 0.1 | -0.9 | -1.8 | -1.2 | -1.3 | -2.7 | -1.9 | -1.9 |
| 17 | -2.8 | -2.7 | -0.3 | -0.7 | -1.9 | -1.6 | -1.3 | 0.3 | 0 | 0.1 | -0.5 | -0.9 | -2 | -2 | -2.3 | -2.2 | 1.7 | 0.9 |
| 18 | -1.9 | -2.5 | -0.5 | 0.1 | -2.4 | -1.9 | 1.6 | 1.6 | -1.2 | -1.2 | -0.6 | -0.7 | 0.9 | -0.9 | -1.5 | -1.5 | 1.2 | 0.1 |
| 19 | -3.6 | -4.7 | -1 | 0.5 | -1.4 | -0.6 | 0.5 | 1.8 | -0.6 | -0.6 | -2.2 | -0.4 | -1.2 | -2.2 | -1.4 | -2.3 | 1.6 | 0.5 |
| 20 | -3.4 | -3.8 | 1 | 0.4 | -2.4 | -1 | 0.9 | 1.2 | -0.7 | -0.7 | -1.6 | -3.1 | -2.6 | -2.8 | -3.9 | -3.5 | 0.4 | 0.1 |
| 21 | -1.7 | -2.4 | -0.5 | 0 | -4.3 | -2.8 | -0.1 | -1 | -0.6 | -1.5 | -1.3 | -1.5 | -2.3 | -3.3 | -2.6 | -1.8 | 0.9 | 0.6 |
| 22 | -1.8 | -2.6 | 0.9 | 0.4 | -0.7 | -0.9 | 0.5 | 1 | -0.3 | -0.5 | -1.8 | -2.8 | -2.1 | -4.2 | -2.2 | -2.5 | 1.8 | 0.7 |
| 23 | -3.6 | -4.5 | 0.2 | 0.2 | -2.7 | -2.3 | -0.8 | -1.3 | 1.2 | 0.5 | -1.8 | -2.7 | -3.7 | -2.5 | -5.2 | -3 | 0.1 | -1.4 |
| 24 | -0.1 | -1.5 | 0.3 | 0.3 | 0.4 | -1.6 | 1.2 | -0.6 | 2.1 | -1 | -0.4 | 0.4 | -2.8 | -2.1 | 0.7 | -1.1 | -1.1 | -2.1 |
| 25 | 0.8 | -1.6 | -0.9 | -1.2 | -0.1 | -0.6 | 1.4 | 0.4 | 1 | 1.2 | -0.4 | -1.3 | -1.5 | -1.4 | -0.7 | -1.4 | -3.3 | -4.1 |
| 26 | -1.8 | -3.1 | 1.7 | -0.2 | -0.8 | -1.6 | 2.8 | 0.3 | 0 | 0.2 | 0.2 | -1.7 | -6.1 | -4.4 | -2.2 | -1.3 | 2.3 | 0.7 |
| 27 | -2.8 | -4.9 | 2.4 | 1.6 | -2.9 | -1.9 | 0.1 | 0.3 | 1.8 | 0.6 | -1.4 | -2.9 | -1 | -2.5 | -2.5 | -3.2 | 0.4 | 1.1 |
| 28 | -1.4 | -1.6 | 1.7 | 2.4 | -1.5 | -1.6 | -2.3 | 0.6 | 0.5 | 1.1 | -1.5 | -0.3 | -2.4 | -1.3 | -2.2 | -1.2 | 0.5 | 0.7 |
| 29 | -2.4 | -3.5 | -0.1 | 0.3 | -2.6 | -2.7 | -1.7 | -0.6 | -3.3 | -4.5 | 1.1 | -1.7 | -4.1 | -3.8 | -2.2 | -1 | 2.9 | 3 |
| 30 | -0.1 | -1.7 | -0.3 | -0.5 | -0.1 | -0.1 | 1 | 0.4 | -0.2 | -0.3 | 0 | -1.7 | -0.1 | -1.1 | -0.1 | -0.8 | -1.5 | -1.7 |
| 31 | -2 | -3.7 | 0.2 | 1.3 | -1.8 | -2 | -1.1 | 0.3 | -0.8 | 0 | -0.9 | -1.1 | -2.4 | -2.5 | -3.8 | -2.5 | 1.3 | 0.2 |
| 32 | -0.3 | -2.5 | 0.8 | 0.3 | -0.9 | -1.8 | 0.4 | 1.2 | 0 | 0.5 | 1.4 | -0.2 | -1.6 | -0.9 | -0.3 | -1 | 3 | 1.4 |
| 33 | -2.9 | -4.1 | 2.7 | 2.4 | -2.6 | -2.1 | -0.2 | 0.3 | 1.2 | 1 | -2.4 | -2.8 | -2.3 | -1.7 | -2.7 | -2.3 | 2.1 | 2.8 |
| 34 | -3.5 | -6.1 | 1.9 | 1.3 | -2.3 | -2.9 | 0.8 | 0.6 | 0.3 | -1.1 | -1.8 | -2 | -2.7 | -3.9 | -1.5 | -2.3 | 1.6 | -1 |
| 35 | -1.9 | -5 | 0.7 | 0 | -2.4 | -3.9 | 1 | -1.1 | 0.2 | -1.7 | -0.7 | -2.7 | -2.5 | -3.2 | -2.8 | -3.6 | 1.5 | 0.4 |
| 36 | -1.1 | -1.3 | 1.3 | 0.4 | -0.5 | -0.8 | 0.1 | 0.8 | 1 | -0.4 | -0.3 | -0.6 | -1.3 | -1.2 | -1.2 | -1.9 | 1.2 | 1.4 |
| 37 | -4.5 | -5.5 | 0.9 | 0 | -2.4 | -2 | -0.9 | -1.3 | 0.1 | -1.1 | -2.5 | -3.1 | -4.9 | -4.1 | -3.8 | -2.3 | 2.5 | 0 |
| 38 | -1.7 | -3 | 0 | -0.2 | -1.8 | -2 | -0.4 | -0.2 | -0.5 | -1.3 | 0.2 | -0.1 | -2.2 | -3.1 | -2.2 | -3.1 | 2.7 | 0.4 |
| 39 | -0.9 | -2.2 | 1.4 | 0.9 | -2.2 | -2 | -0.8 | -0.8 | 1.4 | 0.1 | 0.3 | -0.9 | -2.1 | -1.9 | -2.2 | -2.1 | 0.1 | 0.2 |
| 40 | -0.8 | -1.7 | -1.1 | -0.6 | -1 | 0.2 | -1 | -0.3 | -0.4 | -0.5 | -0.9 | -1.4 | -3.5 | -4 | -1.5 | -1.2 | -1.4 | -1.8 |
| 41 | -1 | -3 | -0.8 | -0.2 | -1.8 | -1.5 | 1.6 | 2 | -0.4 | -1.7 | -0.2 | -0.1 | -2.7 | -2.7 | -1.5 | -1.9 | 0.6 | 0.3 |
| 42 | -0.2 | -2.6 | 0.2 | -0.4 | -1.4 | -1.4 | 0 | -2 | 0 | -2.3 | -1.2 | -2.1 | 0.7 | -1.1 | -0.9 | -3.1 | 1.7 | -2 |
| 43 | -1.1 | -3 | 1.3 | 0.8 | -0.2 | -0.6 | -0.9 | -0.6 | 1.7 | 0.4 | -2 | -2.1 | -1.3 | -1.5 | -3.7 | -2.8 | 0.7 | -2.2 |
| 44 | -3.8 | -2.4 | 1.2 | 2 | -2.2 | -2.4 | -0.8 | -0.8 | 0.7 | 1 | -2.3 | -0.1 | -2.7 | -2.4 | -1.9 | -1.6 | 1.1 | 2.5 |
| 45 | 0.6 | -0.4 | 2 | 0.9 | -0.6 | -0.4 | -0.3 | 0.3 | 3.7 | 2.7 | 0.5 | -0.5 | 0.6 | 0.3 | -1.6 | -1.7 | -0.1 | -0.2 |
| 46 | -2 | -1.8 | 0.3 | 0.6 | -2.8 | -2.1 | -2.7 | -2.2 | -1.2 | -1.6 | -2.2 | -0.9 | -0.9 | -1.5 | -3.8 | -2.7 | 2.6 | -0.6 |
| 47 | -0.2 | -2.4 | 0.5 | 0.7 | -0.5 | -0.6 | -0.9 | -1 | -0.6 | -1.6 | 0.1 | -0.9 | -1.7 | -2.4 | -0.8 | -1.6 | 1.4 | -0.9 |
| 48 | -2.8 | -1.3 | 0.2 | -0.3 | -1.2 | -1.1 | 0.4 | 1.6 | -1.1 | -1.7 | -1 | 0.8 | -2.1 | -0.8 | -2.6 | -1.6 | 0.2 | 1.3 |
| 49 | -2.2 | -2.4 | 1.4 | 0.8 | -1.6 | -1.3 | 1.1 | -0.3 | 1.4 | 0 | 0 | -1.1 | -2.1 | -1.6 | -4.1 | -2.3 | -0.1 | -0.2 |
| 50 | -1.9 | -3.6 | 0.2 | -0.8 | -2 | -1.8 | -0.4 | -0.1 | -1.1 | -2.9 | 0.7 | -0.5 | -1.8 | -1.4 | -3.5 | -3.3 | -0.2 | -0.3 |
| 51 | -1.7 | -1.6 | 0 | 0.1 | -2 | -0.9 | -0.1 | 0.2 | -1 | -1.2 | -1.6 | -2.7 | -2.2 | -1.1 | -2.4 | -0.8 | 0.8 | 0.3 |
| 52 | -1.9 | -3.3 | 0.3 | 0.1 | -0.8 | -0.8 | 0.1 | -1.7 | -0.2 | -1 | -0.6 | -0.8 | -1.7 | -1.6 | -2.8 | -3.3 | 2.3 | 2 |
| 53 | -2.5 | -0.4 | -0.9 | -2 | -1.3 | -0.5 | -1.1 | -1.7 | -2.2 | 0.1 | 1.2 | -3 | -2.6 | -2.2 | -1.5 | 3.1 | 0.5 | 0.4 |
| 54 | -0.6 | -2.8 | 0.6 | 1.2 | -1 | -1.3 | 1.1 | 0.4 | 0.2 | 0 | -0.9 | -1.6 | -1.5 | -2.3 | -0.4 | -1.9 | 0.3 | 0.4 |
| 55 | -2.5 | -2.7 | 1.4 | 1.2 | -2.7 | -1.5 | -1.5 | -0.9 | 1.7 | 0.8 | -0.9 | -2.5 | -2.2 | -2.4 | -4.3 | -3.2 | 0.9 | 0.5 |
| 56 | -1 | -1.3 | 1.7 | 0.9 | -1.5 | -1.1 | -0.3 | -0.4 | 1.9 | 0.7 | 0.1 | -0.8 | -2.6 | -2.6 | -1.4 | -1.4 | 1.3 | -0.6 |
| 57 | -1.4 | -3.6 | -0.2 | 0.2 | -0.2 | -1.4 | 2.1 | 1.9 | 2.3 | 0.9 | 1 | -1 | -1 | -1.9 | -1.6 | -2.1 | 1 | 1 |
| 58 | -1.3 | -2.2 | 1.3 | 0.7 | -0.9 | -1.4 | 0.4 | 0.9 | 1.8 | 2.2 | 0.9 | -1.6 | -1.1 | -0.7 | -2.7 | -2.3 | 0.5 | 0.4 |
| 59 | -2.9 | -3.7 | 0.8 | 0.8 | -2.1 | -2.2 | -0.2 | 0 | -1.4 | -0.9 | -1.9 | -1.7 | -2.3 | -1.8 | -4.5 | -2.9 | 2 | 0.7 |
| 60 | -2.4 | -2.6 | -0.9 | 0.5 | -1.1 | -0.6 | 1.4 | 0.8 | 0.1 | 0.6 | -0.6 | -0.3 | -1.7 | -2.7 | -3.9 | -2.6 | 0.8 | 1.1 |
| 61 | 0.1 | -1.2 | 0.7 | 0 | 0.3 | -0.3 | -0.2 | 1.3 | 2.3 | 1.1 | -0.8 | -0.1 | -0.6 | -0.8 | -1.3 | -1.8 | -1 | -2.3 |
| 62 | -3.9 | -2.6 | 0.2 | 0.2 | -2.7 | -1.3 | 0 | 1 | 1.7 | 1.6 | -2.2 | -1.6 | -2.2 | -2 | -3.7 | -1.3 | -0.5 | -1.1 |
| 63 | -1.7 | -2.5 | -0.6 | 0.3 | -1 | -1.3 | 0.3 | -0.1 | -1.5 | -1 | -1.3 | -0.6 | -2.4 | -2.8 | -2.8 | -2.8 | 2.3 | -1 |
| 64 | -1.3 | -2.4 | -0.1 | 0.7 | -0.3 | -0.8 | -1.4 | -0.3 | 1.4 | 1.4 | -2 | -1.2 | -0.2 | 0.2 | -2.5 | -1.2 | 0.7 | 0.4 |
| 65 | -1.9 | -2.8 | 1.5 | 1.4 | -1 | -0.6 | 0.3 | 0.4 | -0.7 | -0.2 | 0.1 | -0.2 | -2.4 | -2.6 | -1.7 | -1.7 | 0.6 | -0.8 |
| 66 | -1.4 | -2.4 | 0.2 | 1 | -3.3 | -2.3 | -0.7 | -1 | -0.7 | -1.2 | 2.1 | -1.2 | -1.1 | -3.3 | -2.9 | -3.3 | 1.3 | 1.9 |
| 67 | -2.1 | -3.6 | 0.9 | 0.8 | -1.2 | -0.4 | -1.7 | -0.6 | -1 | -0.6 | -0.9 | -0.5 | -2.6 | -3.8 | -0.7 | -2.4 | 1.8 | 2.3 |
| 68 | -1.8 | 0.3 | -1 | 0.3 | -2.3 | -1.8 | -2.6 | -0.7 | 0.7 | 1.1 | -1.3 | 0.5 | -2.6 | -1 | -4.9 | -1.9 | 0.6 | 0.8 |
| 69 | -1.2 | -4.5 | 0.1 | -0.6 | -1 | -2.2 | 2.4 | 1.1 | -1.3 | -2 | 1.2 | -2 | -2.7 | -3.7 | -2.3 | -3.2 | 0.6 | 0 |
| 70 | -2.2 | -2.1 | -0.5 | 0.1 | -0.5 | -1.3 | 0.1 | 0.3 | 1.1 | 0.4 | -0.4 | -1.2 | -0.3 | -1 | -2.6 | -2.1 | -1.4 | 1 |
| 71 | -2 | -3.5 | 1.3 | 0.8 | -1.1 | -0.6 | -1.7 | 0.7 | 0.4 | -0.4 | -1.4 | -1 | -1.4 | -3.4 | -0.7 | -2 | 1.2 | 0.9 |
| 72 | -1.7 | -3.4 | 2.7 | 1.1 | -0.1 | 0 | -0.5 | -1.1 | -1.8 | -1.3 | 0.2 | -1.7 | -2.2 | -1.8 | -2.1 | -3.1 | 4.4 | 3.8 |
| 73 | -1.8 | -3.4 | 1.6 | 0.7 | -1.4 | -0.6 | -0.5 | 0.6 | -0.1 | -0.6 | -0.2 | -2.2 | -2.2 | -2.5 | -1.3 | -1.7 | 0.8 | 1.2 |
| 74 | -1.2 | -2.6 | 0 | -0.1 | -1.1 | -1.7 | 1.5 | -0.6 | 0.9 | -0.2 | 0.1 | -1.3 | -2 | -3.1 | -1.5 | -1.6 | -0.9 | -1.3 |
| 75 | -0.4 | 0 | 0.5 | 0.7 | -1.6 | -1.1 | -0.7 | -0.4 | 1.7 | 1.3 | 0.8 | 0 | -0.4 | -0.5 | -1.5 | -1.2 | 1.2 | 0.3 |
| 76 | -1.4 | -1.8 | 1.6 | 0.5 | -1.9 | -1.6 | -1.2 | 1.1 | 0.9 | -0.5 | -1.2 | -2 | -0.5 | -0.3 | -2.2 | -2.1 | -2.6 | -1.6 |
| 77 | -2.4 | -0.3 | -1.5 | -0.3 | -3.1 | -2.1 | 0.4 | -0.1 | -0.1 | -0.1 | -1.5 | -1.2 | -3.9 | -1.4 | -1.3 | -0.3 | -0.8 | 1.6 |
| 78 | -3.4 | -2.2 | -0.3 | -0.3 | -2.3 | -2.2 | 0.3 | 0.9 | 0.4 | 0 | -1.9 | -0.1 | -3.3 | -1.1 | -4.7 | -1.1 | -0.3 | -0.8 |
| 79 | -1.7 | -1.7 | -0.2 | 0.2 | -0.9 | -0.1 | 0.5 | 0.8 | -0.7 | -0.2 | -1 | -1.8 | -0.5 | -1 | -1.2 | -1.8 | 0.1 | 0.4 |
| 80 | -2.4 | -2.9 | 1.3 | 0 | -2.9 | -3.7 | 0.3 | 0.3 | -0.6 | -0.7 | -1.3 | 0.4 | -3.6 | -2.8 | -1.8 | -2.7 | 2 | 1.6 |

| Region of Interest | Inferior parietal, but supramarginal and angular gyri | | | Inferior parietal, but supramarginal and angular gyri | Inferior temporal gyrus | Inferior temporal gyrus | Insula | Insula | Lenticular nucleus, pallidum | Lenticular nucleus, pallidum | Lenticular nucleus, putamen | Lenticular nucleus, putamen | Lingual gyrus | Lingual gyrus | Mesial temporal lobe | Mesial temporal lobe | Middle cingulate and paracingulate gyri | Middle cingulate and paracingulate gyri | Middle frontal gyrus | Middle frontal gyrus |
| --- | --- | --- | --- | --- | --- | --- | --- | --- | --- | --- | --- | --- | --- | --- | --- | --- | --- | --- | --- | --- |
| Side | L | | R | | L | R | L | R | L | R | L | R | L | R | L | R | L | R | L | R |
| Number 1 | -1.5 | | -0.1 | | -0.7 | -1 | -0.1 | -0.6 | -0.5 | -0.9 | 0.6 | 0.7 | 1.8 | 2.3 | 0.6 | 0.4 | 2.9 | 2.2 | -1.3 | -2.5 |
| 2 | -1.8 | | -2.9 | | -0.8 | -0.8 | 0.3 | -0.3 | 0.5 | 0.9 | 0.1 | -0.6 | 2.2 | 1.9 | -0.4 | -0.5 | 1.8 | 0.6 | -1.6 | -1.6 |
| 3 | -1 | | -0.6 | | -1.7 | -2.4 | -1.5 | -2.7 | 1.2 | -0.5 | -0.1 | -1.1 | 3.2 | 1.6 | -0.2 | -1.1 | 0.9 | -0.4 | -1.6 | -2.7 |
| 4 | -1.2 | | -0.8 | | -3.2 | -2.6 | 0.2 | 1.1 | -0.1 | -0.1 | 1.8 | 2.5 | 2 | 1.9 | 0 | -0.2 | -1.7 | -1.8 | -3.5 | -2.6 |
| 5 | -0.1 | | -1.7 | | -1 | -2.3 | -0.8 | -0.9 | 0.4 | -1 | -0.5 | -0.7 | 2.2 | 0.9 | -1.5 | -2.3 | 0.8 | 1 | -0.4 | -1.3 |
| 6 | 0.1 | | 1.1 | | -1.6 | -1.6 | 0.3 | 0.5 | 0.9 | 1.2 | -0.5 | -0.6 | -0.6 | 0.2 | -0.4 | 0 | 2.2 | 2.3 | -0.2 | -0.1 |
| 7 | -0.6 | | -0.8 | | -1.2 | -0.5 | -1.5 | -0.9 | -1.4 | -0.6 | -0.5 | -0.7 | 2.4 | 1 | -1.3 | -1.8 | -0.7 | 0.3 | -0.6 | -1.4 |
| 8 | -2.6 | | -1.7 | | -1.2 | -1.8 | 0.8 | 0.5 | 0.2 | -0.7 | 2.4 | 2.3 | 0.2 | 1 | -0.7 | -1.1 | 1 | 0.3 | 0.4 | -0.4 |
| 9 | -3.1 | | 0.3 | | -1.3 | -1.5 | 0.6 | 0.2 | 0.8 | -0.2 | 1.4 | 1.8 | 1.9 | 1 | -0.7 | -0.9 | 1 | 0.4 | -0.1 | -1.1 |
| 10 | -0.8 | | -0.6 | | -0.4 | -1.4 | 0.7 | 0.8 | 1 | 0.1 | -0.8 | -0.5 | 2.2 | 1.3 | 0 | 0 | 1.6 | 0.6 | -0.1 | -1.7 |
| 11 | -0.9 | | -1.2 | | -2 | -1.8 | -3.2 | -2.2 | 1.2 | 0.9 | 1.2 | 0.7 | 2.3 | 0.7 | -0.6 | -1.6 | 0.8 | 1.6 | -1.9 | -0.9 |
| 12 | 0.1 | | -0.5 | | -1.6 | -2.1 | 0.5 | 0.9 | -0.8 | -1.1 | 1 | 0 | -0.3 | -0.6 | -1.1 | -1.1 | 2.2 | 1.6 | -0.2 | -0.2 |
| 13 | -1.5 | | -0.4 | | -3.2 | -2 | -2.6 | -1.4 | -2.4 | -1.8 | -2.2 | -2.8 | 2.6 | 1.2 | -1.9 | -1.7 | 2.6 | 0.9 | -0.2 | 0 |
| 14 | -0.9 | | -0.5 | | -2.8 | -2.3 | -2 | -0.1 | 0.8 | 0.4 | -0.7 | 0.7 | 1.8 | 2.2 | -1.2 | -1.5 | 1.4 | 1.3 | -1.4 | -2 |
| 15 | -2.5 | | -1.4 | | -2.9 | -2.7 | -3.2 | -3.1 | -0.2 | 1.5 | 0.1 | 1.3 | 2.1 | 1.5 | -0.6 | -1 | 0.7 | -0.4 | -3.1 | -2.9 |
| 16 | -0.4 | | -0.4 | | -0.5 | -0.9 | 0.8 | 0.8 | 0.7 | 2.1 | 0.8 | 0.1 | -0.1 | -0.3 | 2.1 | 0.4 | 1.3 | 1.2 | 0.7 | -0.7 |
| 17 | 0.5 | | 1.1 | | -1.5 | -1 | -1.6 | -1.5 | -0.7 | -0.6 | -0.8 | -1.4 | 1.2 | 1.5 | -1 | -0.6 | 0 | -1.6 | -0.8 | -1.3 |
| 18 | 0.9 | | 0.9 | | -2.3 | -1.9 | -0.1 | -0.4 | 0.4 | 0 | 3 | 3 | 1 | 0.7 | -1.2 | -1.3 | 2.1 | 1.7 | -0.8 | -1.1 |
| 19 | -1.5 | | -0.8 | | -1.6 | -0.7 | 0.7 | 0.8 | 3.6 | 0.8 | -0.8 | -0.2 | 2.4 | 1.9 | -1.4 | -0.9 | -0.3 | 0.2 | -1.9 | -2.3 |
| 20 | -2.9 | -2.8 | | | -1.8 | -2.1 | 0.3 | 1 | 1.8 | 0.4 | 0.8 | 1.7 | 1.5 | 0.7 | -1 | -0.8 | 1 | 0 | -1.8 | -1.9 |
| 21 | -1.8 | -1 | | | -1.5 | -1.7 | -0.7 | -0.8 | 0.4 | -0.2 | 3.1 | 3.4 | 1.6 | 0.7 | -0.2 | -0.8 | 2.8 | 1.5 | -0.2 | -1.3 |
| 22 | -1.3 | -0.2 | | | -2.4 | -2.3 | -0.4 | -0.4 | 1 | 2.1 | -0.9 | -2.7 | 1 | 0.4 | -0.4 | -0.7 | 1.1 | 0.5 | 0.4 | -0.7 |
| 23 | -2.3 | -1.3 | | | -2.2 | -1.7 | -2.4 | -1.4 | -1.5 | -1 | 0.6 | -0.6 | 2 | 0.4 | 0.4 | -0.5 | 1.1 | 0.6 | -2.2 | -1.9 |
| 24 | -1.5 | -1.4 | | | 0.2 | -1.6 | 0.4 | 0.9 | -0.4 | 3.1 | -0.2 | 2.4 | 0.5 | 2 | 0.8 | 0.3 | 2.4 | -0.1 | 0.2 | -1.3 |
| 25 | -1.9 | -1.1 | | | -0.5 | -1.8 | 1.3 | 0.5 | 1.2 | 1 | 2.8 | 2.7 | -2.9 | -1.8 | 1 | 0.8 | 2.7 | 0.5 | 1 | -1.6 |
| 26 | -1.9 | -0.6 | | | -2.3 | -2.5 | -1.6 | -1.1 | -0.1 | 1.9 | -0.4 | 0.5 | 0.5 | 2 | -0.8 | 0.2 | 0.9 | 0.8 | 0.1 | -0.7 |
| 27 | -2.7 | -2.9 | | | -1.3 | -2.6 | -0.6 | -1.7 | 1.4 | 1.4 | 0.1 | 0.1 | 4.4 | 1.8 | 0.8 | 0 | 0.4 | -0.2 | -1.9 | -2.9 |
| 28 | -2.2 | -1.6 | | | -1.3 | 0.1 | -2.1 | -0.4 | 1.8 | 0.8 | 1.1 | 2 | 2.2 | 1 | 0.6 | 0.9 | 1.3 | 0.8 | -0.9 | -0.8 |
| 29 | -2 | -1.7 | | | -2.2 | -1.6 | -1.6 | -1 | -0.1 | -0.6 | 0.6 | 0.5 | 1.6 | 1.8 | -2.4 | -3 | -1.9 | -2 | -0.1 | -1.1 |
| 30 | -0.1 | -0.2 | | | 0 | -0.7 | 0.3 | -0.5 | 0.5 | 0.3 | 0.4 | 1 | 0.1 | 0.5 | -0.3 | -0.8 | 0.9 | 0.6 | 0.3 | -0.9 |
| 31 | -1.1 | 0 | | | -1.3 | -1.8 | -1.1 | -1.3 | -0.4 | -0.8 | -0.4 | -1.1 | 2.2 | 0.3 | -0.6 | -0.5 | 0.7 | 1.7 | -1.4 | -2.4 |
| 32 | -3.1 | -1.3 | | | 1 | -0.8 | 0.3 | 0.4 | 0.2 | 0.2 | 0.6 | 0.9 | 0.7 | -1 | 0.4 | 0.2 | -0.1 | -0.4 | 1.8 | -0.9 |
| 33 | -2.5 | -2.3 | | | -1.5 | 0.2 | -1.1 | -1 | 3.1 | 2 | 0.1 | 1 | 4.2 | 2.1 | 1 | 0.6 | -0.2 | -0.2 | -1.9 | -1.9 |
| 34 | -0.4 | -0.6 | | | -0.1 | -1 | 0.3 | 0.3 | 0.9 | 0.5 | 0.2 | 0.2 | 1.9 | 0.1 | -0.7 | -1.5 | 1.1 | -0.2 | -1.5 | -2.8 |
| 35 | -0.5 | 0.1 | | | -0.9 | -1.7 | -0.8 | -1.7 | 0.8 | 0.3 | -0.5 | -0.6 | 2.2 | 2 | -0.7 | -2.1 | -1.4 | -1.1 | 0.1 | -2 |
| 36 | -2.1 | -0.9 | | | -1.8 | -1.6 | -0.2 | 0.1 | 1.2 | 0.6 | 1.3 | 0.7 | 1.2 | 0.7 | 0 | 0 | 1.8 | 1.6 | 0.1 | 0 |
| 37 | -0.9 | -0.8 | | | -1.9 | -1.9 | -2.1 | -2.4 | -0.4 | -1.4 | -0.4 | -0.1 | 2 | 1.3 | -1.7 | -1.9 | -0.8 | -1.4 | -2.3 | -2.1 |
| 38 | -0.6 | -1.5 | | | -1.6 | -1.6 | -0.2 | -0.9 | 4.7 | 2.8 | -0.4 | 0.3 | -0.3 | 1.9 | -0.1 | -0.4 | -0.9 | 0.5 | -0.2 | -0.1 |
| 39 | -1.2 | -2 | | | -1.4 | -1.5 | -1.1 | -1.6 | 0.8 | 0.5 | 1 | 0.4 | 1.1 | 0 | 0.2 | -0.8 | 1.1 | 0.4 | -0.1 | -1.3 |
| 40 | -0.5 | -0.4 | | | -3.2 | -1.7 | -2.2 | -1.4 | 0.4 | 0.8 | 0.5 | 0.4 | -0.2 | 0.1 | -1.6 | -1.1 | 2.5 | 2.4 | -0.1 | -0.1 |
| 41 | -0.9 | -1.3 | | | -2.6 | -2.3 | -0.4 | -0.2 | -0.4 | -0.1 | -2 | -2.2 | 1.5 | 1.2 | -1.3 | -2.4 | 2 | 1.2 | -0.1 | -1.5 |
| 42 | 0.5 | 1.9 | | | 0.3 | -1.2 | -0.1 | -1.4 | -0.6 | -0.8 | -1.3 | -1.5 | 1.6 | 0.2 | -0.3 | -1.1 | 1.8 | 0.8 | 0.5 | -1.1 |
| 43 | -1.1 | -0.5 | | | -0.6 | -1.3 | -0.7 | -0.7 | 0.6 | 0.7 | -0.2 | -0.4 | 1.2 | 1.7 | 1.7 | 0.7 | 1.1 | 0.7 | -1.3 | -2 |
| 44 | -2.6 | -1.3 | | | -1.2 | -0.3 | -0.8 | -0.9 | 0.9 | 1.9 | 1.2 | 0.6 | 1.2 | -0.2 | 0.1 | -0.5 | 0.6 | -0.5 | -3.3 | -0.7 |
| 45 | 0.9 | 0.3 | | | -0.6 | -1 | 1.1 | 1.1 | 1.9 | 2.7 | 1.5 | 1.7 | 0.9 | 0.6 | 3.4 | 2.6 | 1.8 | 1.1 | -0.1 | -0.7 |
| 46 | -2.4 | -1.1 | | | -2.1 | -1 | -1.7 | -1.9 | 1.8 | 1.3 | 0.9 | 0.1 | 2.5 | -0.2 | -2.2 | -1.3 | 0.2 | 0.1 | -0.4 | -0.9 |
| 47 | -1.8 | -0.6 | | | -0.4 | -0.3 | -1.4 | -1.7 | -1.2 | -2 | 0.3 | 0.4 | 2.5 | 1 | 0 | -0.4 | 1.5 | 1.1 | 0.4 | 0.3 |
| 48 | -1.7 | -1 | | | -2.4 | -1.1 | -0.9 | 0 | -1.9 | -0.3 | 0.9 | 0.8 | 1.6 | 1.9 | -1.2 | -1.4 | 1.5 | 0.9 | -2.5 | -0.1 |
| 49 | -0.2 | -0.7 | | | -2 | -2 | -0.8 | -0.6 | 2.1 | 0.4 | -0.5 | -0.3 | 2.7 | 0.6 | 1 | 0.2 | 1.5 | 1.3 | -1.5 | -1.5 |
| 50 | -1.3 | -1.1 | | | -1.9 | -2.6 | -1.5 | -1.6 | 0.2 | -0.2 | -0.1 | 0.8 | 2.4 | 2.3 | -1.3 | -2.3 | 0.4 | 0.1 | -0.9 | -1.4 |
| 51 | -2.4 | -0.3 | | | -1.6 | -1.8 | -1.4 | 0 | 1.8 | 2 | 5.6 | 5.6 | 2.1 | 1.9 | -0.4 | -0.7 | 0.4 | -0.6 | -2 | -1.6 |
| 52 | -0.9 | -0.6 | | | -1.1 | -1.6 | -1.6 | -1.9 | 2.7 | 1.6 | -0.4 | 1 | 0.6 | 0.6 | -0.5 | -1.1 | 1.7 | 1.6 | 0.5 | -1.4 |
| 53 | -1.2 | -0.7 | | | -1.6 | -1.7 | -1.2 | -1.8 | -1 | -0.4 | -0.9 | -1.7 | 0.5 | 0.9 | -2.3 | -2.6 | 0.2 | 1.5 | 0 | -0.7 |
| 54 | 0.3 | 0.7 | | | -0.2 | -0.9 | 1.4 | 0.7 | 2.3 | 1.8 | 1.7 | 0.6 | 0.4 | 0.9 | -0.6 | -0.9 | 2.5 | 1.1 | 0.5 | -1.1 |
| 55 | -1.8 | -0.7 | | | -1.2 | -0.2 | -1.4 | -1.8 | 0.7 | 0.2 | 0 | -0.6 | 1.8 | 1.9 | 0.7 | 0.3 | 0.5 | 0.9 | -1.6 | -1.5 |
| 56 | -1 | -0.8 | | | 0.3 | 0.3 | 0.7 | 1.4 | 0.5 | 0.3 | 2.4 | 3.1 | 1.2 | 0.9 | 1.3 | 0.3 | 0.6 | 0.9 | -0.7 | -0.3 |
| 57 | 0.4 | 0.2 | | | -0.6 | -1.4 | 2.2 | 1.9 | 2.5 | 2.3 | 1.5 | 0.7 | 1.5 | 0.1 | 0.8 | -0.1 | 1.1 | 0.2 | -1.1 | -1.5 |
| 58 | -0.8 | 0.2 | | | -1.1 | -0.5 | 0.2 | 0.1 | 0.1 | 0.7 | 2.2 | 2.3 | 1.2 | 0.7 | 1.9 | 2.1 | 2 | 2.3 | -1.5 | -1.1 |
| 59 | -2 | -1.3 | | | -1 | -1 | -1.8 | -1.9 | -0.4 | -0.1 | 2 | 2.3 | 1.9 | 0.5 | -1.4 | -0.6 | 1.4 | 0 | -1.3 | -1.7 |
| 60 | -1 | 0 | | | -2.9 | -1.9 | -0.3 | -0.6 | 0.8 | 0.5 | 1.2 | 0.3 | 0.2 | 1.7 | -0.8 | -0.2 | 1.2 | 1.6 | -2.2 | -1.8 |
| 61 | -0.5 | | -0.7 | | -2.6 | -2.2 | 0 | 0.5 | 2.5 | 2.2 | 2 | 2.3 | 0.4 | -0.4 | 1.8 | 1.7 | 2.6 | 2.3 | 0.3 | -1 |
| 62 | -3 | | -2.6 | | -2.7 | -2.3 | -0.4 | -1 | 1.8 | 2.5 | 2.7 | 2.2 | 1.3 | 1.9 | 0.9 | 1.4 | 1.4 | 1.4 | -2.1 | -2 |
| 63 | -0.1 | | -0.6 | | -1.8 | -1.5 | -0.2 | 0 | -0.2 | -0.1 | -2 | -1.7 | 2.3 | 1.8 | -1.2 | -0.4 | -0.6 | 1.6 | 0.1 | -0.4 |
| 64 | -4 | | -3 | | -1.3 | -0.6 | -1.5 | -0.8 | 2.6 | 2.6 | 1.8 | 2.2 | 1.4 | 2.2 | 1.3 | 1.1 | 0.7 | 0.1 | -0.1 | -1.4 |
| 65 | -2.2 | | -0.9 | | -2.2 | -3.1 | 0 | -0.7 | -0.4 | 0.1 | 1.6 | 1.7 | 2.9 | 0.4 | -0.7 | -0.6 | 1.7 | -0.4 | -0.8 | -1.2 |
| 66 | -0.5 | | -0.5 | | -2.1 | -1.7 | -1.5 | -1.5 | -0.9 | -0.6 | 0.3 | -0.3 | 3 | 0.1 | -0.4 | -0.7 | 0.9 | 1.6 | 0.9 | 0.7 |
| 67 | -3.5 | | -2.9 | | -2.8 | -1.7 | 0 | -1.3 | -1.6 | -1.2 | -0.9 | -1.8 | 5.2 | 3.4 | -1.8 | -1.3 | 1.5 | 0.7 | -1.4 | -1.8 |
| 68 | -1.8 | | -1.2 | | -2.8 | -2.2 | -0.3 | -0.5 | 2.9 | 1.7 | -0.1 | 0.7 | 0.6 | 1.3 | 0.2 | 0.4 | 1.2 | 1.2 | -0.6 | 1.2 |
| 69 | -1.7 | | -1.1 | | -1.6 | -1.1 | 0.6 | 1.3 | 3.4 | 4.4 | -0.3 | -1.3 | -0.8 | 0.1 | -1.3 | -2.3 | -0.1 | 0.4 | 0 | -1.9 |
| 70 | -0.7 | | -1.1 | | -1.9 | -1.7 | 1 | 0.4 | 0.9 | 0.4 | 2.1 | 1.5 | 2.1 | 0.5 | 1.1 | 0.5 | 0.3 | 1.4 | -2.3 | -1.2 |
| 71 | -1.2 | | -1.5 | | -0.6 | -1 | -0.1 | -0.5 | 0.8 | 0.5 | 0.3 | 0.5 | 0.6 | -0.5 | 0.2 | -0.9 | 0.7 | 0.5 | -0.6 | -0.7 |
| 72 | -2.5 | | -1.3 | | -1.8 | -1.5 | -0.9 | -1.1 | -1.1 | -1 | -0.1 | -0.7 | 3.1 | 1.7 | -1.8 | -2.1 | 0.7 | -0.4 | -0.9 | -1.2 |
| 73 | -0.8 | | -0.3 | | 0.1 | 0.2 | -0.4 | -0.9 | 2.3 | 1.8 | 0.9 | 1.1 | 2.2 | 2.8 | 0.2 | -0.4 | 0.8 | 0.7 | -0.6 | -1.2 |
| 74 | 0.3 | | -1.3 | | -2.3 | -2.3 | 0.8 | 0.3 | -0.1 | 0 | -0.4 | -2 | 0.3 | 0.3 | 0.2 | -0.8 | 0.8 | 0.2 | -0.3 | -0.5 |
| 75 | -2.7 | | -2 | | -1.3 | -1.2 | 0.4 | 0.3 | 1.8 | 2.2 | -0.7 | -0.7 | 3.4 | 2.8 | 1.6 | 1.5 | 0.4 | 0.4 | 0.1 | -0.5 |
| 76 | -2.5 | | -0.7 | | -2.1 | -1.1 | -0.8 | 0.5 | 0.6 | 1.4 | 0 | 0.5 | 0.9 | 1.3 | 0.8 | 0.1 | 1.8 | 0.6 | 0 | -1 |
| 77 | 0.7 | | 3.5 | | -2.2 | -1.6 | 0.6 | 0.7 | 1.6 | -0.1 | -0.5 | -0.7 | 0.7 | -0.6 | 0.2 | 0.1 | 2 | 2.5 | -0.9 | 1.1 |
| 78 | 0.1 | | -0.8 | | -2.6 | -1.6 | -0.9 | 0 | 3.4 | 2.4 | 2.1 | 3.6 | 0.5 | 0.7 | -0.8 | -0.3 | 1.3 | 0.6 | -0.3 | -0.6 |
| 79 | -0.9 | | -0.6 | | -1.4 | -0.7 | -0.2 | -0.2 | 0.8 | 0.5 | 0.9 | 0.8 | 0.8 | 0.9 | 0.2 | 0.5 | 2.3 | 2 | -0.5 | -0.9 |
| 80 | -0.2 | | 0.2 | | -0.8 | -1.2 | -0.7 | -0.2 | 1.5 | 2.7 | -0.6 | -0.2 | 3 | 1 | -1.1 | -1 | 1.8 | 2.4 | -0.4 | -1.7 |

| Region of Interest | Middle frontal gyrus, orbital part | Middle frontal gyrus, orbital part | Middle occipital gyrus | Middle occipital gyrus | Middle temporal gyrus | Middle temporal gyrus | Occipital lobe | Occipital lobe | Olfactory cortex | Olfactory cortex | Paracentral lobule | Paracentral lobule | Parahippocampal gyrus | Parahippocampal gyrus | Parietal lobe | Parietal lobe | Postcentral gyrus | Postcentral gyrus |
| --- | --- | --- | --- | --- | --- | --- | --- | --- | --- | --- | --- | --- | --- | --- | --- | --- | --- | --- |
| Side | L | R | L | R | L | R | L | R | L | R | L | R | L | R | L | R | L | R |
| Number 1 | -0.5 | -0.3 | -1.4 | -1.1 | -3.1 | -1.8 | 1.3 | 1.7 | 1.3 | 1.1 | 0 | 0.6 | -0.2 | -0.5 | -0.5 | 0.2 | -0.1 | -1.2 |
| 2 | -0.9 | 0.2 | 0.2 | 0.2 | -2.1 | -1.4 | 2.7 | 2.4 | 1.8 | 1.3 | -0.1 | -0.5 | -0.5 | -1.5 | -2.3 | -2.2 | -0.2 | -1.5 |
| 3 | -1.6 | -2 | -0.3 | 0 | -1.7 | -2.4 | 3.3 | 2 | 0 | -0.3 | 2.2 | 0.4 | -1 | -0.8 | 0.1 | -1.8 | -0.4 | -3.3 |
| 4 | -0.2 | 0.2 | -0.9 | -0.6 | -4.2 | -2.1 | 1.7 | 2 | -0.3 | -0.7 | 0.7 | -0.6 | -0.3 | -0.5 | -2.4 | -1.3 | -0.5 | -0.6 |
| 5 | -0.9 | -0.5 | 0.3 | 0.8 | -2.6 | -2.4 | 0.4 | 0.4 | -0.3 | -0.3 | -0.8 | -0.9 | -1.1 | -1.7 | -0.2 | -0.2 | -0.1 | -0.8 |
| 6 | -0.5 | -0.6 | -2.5 | -0.6 | -2.8 | -1 | -0.5 | 0 | -0.8 | -0.1 | 0.9 | 0.7 | -0.4 | -0.7 | -0.1 | 0.4 | -0.4 | -0.9 |
| 7 | -0.9 | -0.7 | 1.3 | 1.5 | -0.9 | 0 | 2.4 | 1.9 | -0.5 | 0.4 | -0.8 | -0.3 | -1 | -1.9 | -1.3 | -1.1 | -1.4 | -1.5 |
| 8 | -0.2 | 0.3 | -1.9 | -0.5 | -1.7 | -1.3 | -0.2 | 0.8 | 1.6 | 1.6 | -0.1 | -1.4 | -0.8 | -0.6 | -1.7 | -1.8 | -0.7 | -2.3 |
| 9 | 0.2 | -0.8 | -1.9 | -0.1 | -3.3 | -2.3 | 0.3 | -0.2 | 1 | -0.4 | 1.3 | -0.8 | -1.8 | -1 | -2.2 | -2.1 | -1.3 | -2.1 |
| 10 | 0.4 | -0.4 | 0.2 | 0 | -0.8 | 0.1 | 1.9 | 1.8 | 0.7 | 0.6 | -1 | -1.7 | -0.4 | -0.7 | -1 | 0.1 | -1.9 | -3.2 |
| 11 | 0.2 | -0.2 | 0 | 2 | -2.4 | -1.8 | 1.8 | 2.6 | 0.7 | -0.2 | -0.9 | 0.6 | 0 | -2 | -2.2 | -1 | -3.7 | -2.8 |
| 12 | -0.1 | 0.5 | -0.2 | 0.1 | -1.1 | -1.6 | 0.4 | 0.2 | -0.8 | -1.2 | 0.8 | 1 | -0.8 | -1.3 | -0.4 | -1.9 | -1.2 | -0.9 |
| 13 | -0.8 | -0.6 | 0 | -0.1 | -4 | -2.5 | 1.2 | 1.5 | -0.4 | -0.5 | 0.3 | -1 | -2 | -0.9 | -2.9 | -1.9 | -2 | -2.8 |
| 14 | -2.1 | -3.4 | -0.7 | -0.6 | -1.8 | -0.6 | 1.2 | 1.9 | -0.6 | -1 | 0.3 | -0.3 | -0.8 | -0.5 | -1.5 | -0.8 | 0.1 | 1.4 |
| 15 | -1.6 | -1.6 | -0.4 | -0.4 | -4.4 | -2.6 | 1.6 | 1.5 | 0.8 | -0.8 | 1.1 | 0.9 | -1.5 | -1.4 | -2.5 | -1.7 | -0.8 | -0.7 |
| 16 | -0.6 | -1.4 | -1 | -0.5 | -1.9 | -1.7 | -0.5 | -0.1 | 1.7 | 0.3 | 1.8 | -0.4 | 1 | 0.1 | 0 | 0 | 0.6 | -0.5 |
| 17 | -0.5 | -0.6 | -1.3 | 0.1 | -3.5 | -1.2 | -0.4 | 0.8 | 1.2 | 1.3 | -0.8 | -0.3 | -1.5 | -0.8 | -0.4 | 0.1 | -1.5 | -1.8 |
| 18 | -1.1 | -0.4 | -0.2 | 0.5 | -1.9 | -1.6 | 0.4 | 1.1 | -0.5 | -1.1 | 0.4 | -0.6 | -0.7 | -0.9 | -0.8 | -0.1 | -1.8 | -1.9 |
| 19 | -0.2 | -0.6 | -0.8 | -0.6 | 1.2 | -0.9 | 1 | 1.6 | 0.9 | 0.1 | -2.9 | -2.4 | -1.4 | -0.5 | -2.2 | -1.2 | -4.2 | -3.4 |
| 20 | -1.3 | -0.9 | 0.7 | 1.5 | -2.9 | -2.2 | 1.6 | 2.4 | 1.6 | 0.6 | 0.5 | -0.1 | -0.7 | -0.3 | -1.8 | -2.8 | -1.1 | -1.9 |
| 21 | -0.3 | -0.7 | -0.4 | 0.1 | -1.7 | -0.8 | 0.6 | 1.5 | -1.4 | -0.9 | 2.2 | 0.3 | 0.5 | 0.1 | -0.6 | -1.1 | -1.3 | -0.7 |
| 22 | -0.4 | -0.1 | -0.6 | 0.4 | -1.7 | -0.7 | 0.6 | 1.2 | 0.9 | 1.3 | -1.1 | -0.4 | -0.1 | -0.3 | -0.7 | 0.2 | -0.8 | -1.9 |
| 23 | -1.2 | -1.6 | -1.5 | 0.6 | -3.2 | -2.1 | 1.7 | 1.1 | -1.8 | -2.1 | 1.2 | -1.1 | -0.6 | -1.3 | -0.6 | -0.7 | -0.3 | -2.2 |
| 24 | -0.6 | 0 | -1.7 | 0.3 | -1.5 | -1.5 | -0.5 | 1 | 0.2 | 3.1 | 0.8 | -1.2 | -0.9 | 2 | -0.8 | -1.1 | -0.7 | -1.3 |
| 25 | 0.6 | -0.8 | -2.2 | -1.4 | 0.8 | -0.1 | -3 | -2.9 | -0.6 | 0.6 | 1.4 | 0.8 | 0.7 | 0.5 | -1.5 | -1.2 | -0.2 | -0.5 |
| 26 | -0.5 | -0.8 | 1.2 | 0.7 | 0.1 | -0.1 | 1 | 1.3 | -0.5 | -0.2 | 0.2 | -0.5 | -1.2 | 0.5 | -1.6 | -1.8 | -0.6 | -1.7 |
| 27 | -1.5 | -1.4 | -1.4 | 0.7 | -2.2 | -2.2 | 2.5 | 2.8 | 1.1 | -0.2 | 1.1 | 0.3 | -0.4 | -0.3 | -2.9 | -2.8 | -0.2 | -2.2 |
| 28 | -0.4 | -1.2 | 0.6 | 0.2 | -2.5 | -1.6 | 2.4 | 2.8 | -0.7 | -0.1 | 0.5 | 0.1 | 0.7 | 0.5 | -1.9 | -1.9 | -1.6 | -2.1 |
| 29 | -0.8 | -1.1 | -0.9 | 0.2 | -1.9 | -1.9 | 1 | 2.8 | -0.2 | -0.2 | -0.4 | -0.3 | -0.9 | -0.8 | -2.4 | -2.3 | -1.2 | -1.7 |
| 30 | 1 | 0.8 | -0.9 | 0 | -0.3 | -0.9 | -0.1 | 0.9 | -0.3 | -0.1 | -0.2 | -0.1 | -0.4 | -1 | 0.6 | -0.2 | -1.2 | -1.8 |
| 31 | -0.9 | -0.7 | -0.1 | -1.3 | -2.9 | -1.4 | 1.6 | 0.8 | 0.2 | -1.1 | 1 | 0.7 | -0.2 | -0.8 | -1 | -0.9 | -0.7 | -3.1 |
| 32 | 0.3 | -1.1 | 1 | 0.3 | -1.7 | -0.9 | 1.4 | 1.3 | -1.1 | -0.1 | -0.5 | -2.3 | 1 | 0.2 | -2.5 | -2.6 | -0.2 | -2.5 |
| 33 | -1.9 | -1.3 | 1.5 | 2 | -1.1 | -0.9 | 4 | 4.4 | 0.1 | -0.2 | 0.4 | -0.4 | 0.3 | 0.1 | -1.6 | -2.2 | 0.6 | -0.6 |
| 34 | 0.6 | -0.3 | 1.2 | -0.4 | -0.8 | -1.6 | 3 | 0.7 | -1.8 | -1.1 | 0.3 | -2.1 | -1.5 | -1.7 | -1.1 | -1.5 | 0.1 | -0.2 |
| 35 | -1.7 | -2.3 | -0.4 | -0.3 | -0.4 | -0.8 | 1.4 | 1.1 | -1 | -1 | 0 | -1 | -1.2 | -1.8 | -0.8 | -0.4 | -1.6 | -4.3 |
| 36 | -1.2 | -0.3 | -1.1 | 0.3 | -1.4 | -1.1 | 0.8 | 1.1 | 1 | 2 | -1.2 | -0.9 | -0.9 | 0.5 | -1.3 | -0.6 | -1.8 | -1.6 |
| 37 | -1.3 | -1.1 | 2.4 | 1.7 | -0.8 | -1.4 | 2.9 | 3.1 | -1.1 | -0.4 | -0.4 | -0.7 | -3 | -2.1 | -0.5 | -1.5 | -0.6 | -2.2 |
| 38 | -0.5 | -0.5 | 2.1 | 1.2 | -1.4 | -1.4 | 1.9 | 1.6 | -0.7 | -2.4 | -1.6 | -1.8 | 0.8 | 1 | -0.7 | -0.8 | -0.7 | -2.5 |
| 39 | 0.3 | -0.1 | 0.3 | 0.8 | -1.8 | -1.8 | 1.3 | 1.5 | 0.7 | 0 | -0.6 | -0.5 | -1.1 | -1.5 | -1.3 | -2.2 | 0.4 | -1.2 |
| 40 | -0.4 | -0.3 | -1.6 | -0.5 | -2.8 | -1.3 | -1 | -0.5 | 0.7 | -0.6 | -0.5 | -0.2 | -2.5 | -1.4 | 0.4 | 0.5 | -1.7 | -2.6 |
| 41 | -1.3 | -2.3 | -1.6 | -0.8 | -2.3 | -2.3 | 0 | 0.8 | 1.1 | 0.2 | 0.9 | -0.6 | -1.7 | -2.4 | -1.5 | -1.2 | -1 | -2.5 |
| 42 | 0.8 | 0.8 | -1.1 | -0.2 | 0.7 | -1.7 | 1.1 | 0.6 | -0.6 | -1.3 | 0.6 | -0.6 | -0.5 | 0.1 | -0.3 | -0.2 | -1.8 | -3.6 |
| 43 | -1.3 | -2.1 | -0.9 | -0.1 | -1.5 | -1.4 | 2 | 1.6 | 2.4 | 0.8 | 0.9 | -0.4 | 1.3 | 0.8 | -0.4 | -0.3 | -1.1 | -3 |
| 44 | -0.7 | 0.7 | -1.1 | 0.6 | -0.4 | -1.2 | 0.4 | 1.9 | -1.1 | 0.4 | 1 | -0.1 | -0.6 | -1.8 | -1.8 | -1.5 | -1.5 | -2.5 |
| 45 | -0.6 | -0.2 | -0.5 | 0.1 | -0.3 | -0.7 | 0.8 | 0.8 | 1.8 | 1 | 1.4 | 1.3 | 2.2 | 2 | 0.7 | 0.9 | -0.1 | 0.2 |
| 46 | -1.1 | -1 | -0.2 | 1 | -2.8 | -1.8 | 1.1 | 0.7 | -1.2 | -1.4 | 0.7 | 0.1 | -2.6 | -0.5 | -2 | -0.8 | -1.1 | -0.6 |
| 47 | -0.3 | 0.7 | -0.6 | 0.2 | -0.8 | -0.2 | 1.1 | 1.2 | -2 | -1 | 0.2 | -1.1 | 0.7 | 0.9 | -0.7 | 0.2 | -1.4 | -1.9 |
| 48 | -1.2 | -0.6 | -2 | -0.7 | -2.6 | -0.1 | -0.1 | 1.6 | -2.1 | -1.2 | -0.2 | -2.3 | -0.9 | -0.7 | -1.7 | -0.9 | -2.5 | -1.1 |
| 49 | -1 | 0.6 | -0.7 | 0.1 | -2 | -1.7 | 0.2 | 1.1 | 1.4 | 1.2 | 0.3 | 0.9 | 0.2 | 0.4 | -0.8 | -1.4 | -0.7 | -3.6 |
| 50 | -1.7 | -1.2 | -0.5 | 1 | -2.3 | -2.3 | 0.8 | 2.6 | -0.9 | -0.8 | -0.6 | -0.9 | -0.7 | -0.9 | -1 | -2.2 | -1.9 | -2.9 |
| 51 | -0.8 | -0.1 | -0.2 | 0.8 | -1.6 | -1.3 | 0.6 | 1.3 | -2.1 | -0.9 | 1.6 | 0.7 | 0.1 | 0.1 | -1 | 0.3 | 0.7 | -1.2 |
| 52 | 0.4 | 0 | 0.2 | 0.2 | -0.3 | -0.7 | 0.4 | 1.5 | -1.2 | -1.1 | -0.7 | -0.5 | -0.3 | -0.7 | 0 | 0 | -1 | -3.1 |
| 53 | -0.8 | 1.3 | -3 | -0.7 | -1.4 | 0 | -0.5 | 0.3 | -1.9 | -1.3 | -0.6 | -2 | -1.7 | -2.1 | -1.2 | 0 | -2 | -4.2 |
| 54 | 0.4 | -0.2 | 1.7 | 2.7 | -1.2 | -0.9 | 1.2 | 1.7 | 1.1 | 0.3 | 0 | -1.2 | -1.1 | -1.5 | 0.2 | -0.1 | -1 | -1.5 |
| 55 | -0.6 | 0.4 | 0.2 | 1.1 | -1.9 | -0.5 | 1.6 | 2.3 | 0.6 | 0.2 | 1.3 | 1.3 | -0.5 | -0.3 | 0.4 | -0.1 | 0.8 | -1 |
| 56 | 0.5 | 1.2 | -0.4 | 0.3 | -1.3 | -0.5 | 1.3 | 0.9 | 1 | 0.8 | 0.3 | 0.2 | 0.4 | -0.1 | -1.5 | -0.4 | -0.6 | -1.5 |
| 57 | -0.7 | -2.7 | -0.8 | 1.3 | 1.3 | -1.6 | 0.7 | 1.6 | 1.2 | 0 | -1 | -1.1 | -1.2 | -1.3 | -0.3 | -0.9 | 1.3 | 0.4 |
| 58 | -0.4 | 0 | -0.7 | 0.7 | -2.8 | -1.1 | 1.1 | 1.4 | 0.6 | 0.9 | 0.4 | 0.9 | 1.6 | 1.9 | -1.2 | -0.8 | 0.8 | 0.9 |
| 59 | -1.2 | -0.5 | 0.7 | 1.3 | -0.4 | -1.4 | 1.7 | 2.2 | -0.4 | -0.6 | -0.3 | -1.6 | -0.9 | -0.2 | 0 | 0.1 | 0.3 | -1.8 |
| 60 | -1.5 | -1.3 | -1.5 | -0.3 | -0.7 | -0.9 | -0.4 | 2.2 | -0.2 | -0.3 | 0.8 | -0.7 | -1.5 | -0.8 | -1.7 | 0.1 | -0.3 | -1.4 |
| 61 | 0.2 | 0.1 | -0.4 | -0.3 | -1.1 | -0.2 | -0.3 | -0.7 | 1.2 | 0.2 | 0.2 | 0.7 | 1 | 1.7 | -0.3 | 0 | 0.2 | 0.8 |
| 62 | -0.4 | 0 | -1.7 | -1 | -2.1 | -1.9 | 0.9 | 1.5 | 0 | -1.1 | 0.4 | -0.3 | -0.2 | 1.1 | -2.1 | -1.4 | 0.9 | 2.9 |
| 63 | 0 | -1.1 | -0.3 | 0 | -1.9 | -1.3 | 0.8 | 1.5 | -0.1 | 0.5 | -0.5 | -0.4 | -0.5 | 0.5 | 0.2 | -0.2 | -1.6 | -3.9 |
| 64 | -1.7 | -1.4 | -1.4 | 0.3 | -0.4 | 0.3 | 1.5 | 2.2 | 1.6 | 1 | -0.7 | -0.1 | 0.7 | 0.6 | -1.3 | -1.3 | -1.4 | -1.1 |
| 65 | -0.8 | -0.7 | -1.3 | -0.5 | -2.5 | -0.9 | 1 | 1.8 | 1.5 | -0.1 | -0.8 | -0.7 | -0.5 | -0.6 | -2 | -1.6 | -0.1 | -0.3 |
| 66 | -0.1 | 1 | -0.1 | 0.9 | -2.4 | -1.1 | 1.4 | 2.1 | -2.6 | -0.6 | -0.4 | -1.4 | 0.4 | 0.3 | -1 | -0.9 | -0.7 | -2.3 |
| 67 | -0.7 | -0.6 | 0.1 | -0.6 | -3.3 | -2.5 | 2.1 | 2.3 | 1.2 | 0.3 | -0.6 | -0.6 | -2.1 | -1.5 | -2.6 | -2.8 | -1.9 | -3.2 |
| 68 | -1.8 | -1.4 | -1.1 | 0.8 | -3.1 | -1.5 | -0.3 | 1.6 | -0.6 | -0.9 | 0.7 | 0.2 | -0.2 | -0.1 | -1.7 | -0.7 | 0.2 | -0.1 |
| 69 | -0.7 | 0.3 | 1.6 | 2.1 | -1.9 | -1.4 | 0.2 | 1.6 | -1.7 | -0.2 | 1.2 | 0.3 | -0.9 | -1.7 | -0.9 | -0.2 | 0.2 | 0.6 |
| 70 | -1.5 | -1.2 | -0.5 | 0.4 | -2.3 | -1.8 | 0.3 | 1.7 | 1.3 | 1 | 1.5 | -0.1 | 0.9 | 0.6 | -0.3 | 0.1 | 0.8 | 1.5 |
| 71 | 0.3 | 0.1 | 0.1 | 0.6 | 1.1 | 0.2 | 0.7 | 1.4 | 0.3 | -1.6 | -0.3 | -1.2 | 0 | -0.9 | 0 | 0 | -0.5 | -1.1 |
| 72 | -2.2 | -2.2 | -0.3 | 0.7 | -2 | -2.7 | 3 | 2.4 | -1.5 | 0.2 | -0.1 | -2.2 | -1.6 | -2.2 | -2.4 | -2.5 | -1.2 | -2.1 |
| 73 | -0.5 | -1.1 | 0.6 | 1 | -1 | -0.3 | 1.5 | 2.6 | 1.3 | 1.3 | -0.6 | -1.3 | 0.6 | -0.1 | -0.2 | 0.3 | -0.3 | -0.7 |
| 74 | -1.2 | -1.2 | 0.7 | -0.4 | -1.1 | -1.9 | 1.4 | 1 | 1.6 | 0.3 | -0.3 | 0.1 | -0.5 | -0.8 | 0.2 | -0.2 | -0.6 | -0.8 |
| 75 | -0.2 | 0.4 | -0.1 | 0.6 | -2.1 | -0.8 | 2.2 | 2.8 | -0.6 | 1.4 | 0.7 | 0.2 | 1.1 | 1.3 | -1.9 | 0.4 | -0.4 | -0.7 |
| 76 | -0.5 | 0 | -1.2 | -0.2 | -1.8 | -0.9 | 0.5 | 1.3 | 1.9 | 0.2 | -1 | 0.7 | 0.4 | 0.4 | -0.5 | 0 | -1.3 | -0.4 |
| 77 | -0.9 | -0.8 | -2.9 | 0.6 | -1.4 | -0.8 | -0.6 | 1.2 | -1.3 | -0.3 | 0 | 0.5 | 0.9 | 0.7 | -1.3 | 0.7 | -1 | -2.6 |
| 78 | -0.8 | -0.8 | -0.6 | 0.4 | -2.5 | -1 | 0.5 | 1.5 | 0 | 0.5 | -1.3 | -2.7 | -1.7 | -0.5 | 0.3 | -0.4 | -1.3 | -1.3 |
| 79 | -0.5 | -0.5 | -0.9 | 0.5 | -1.3 | 0.5 | 0.4 | 1 | 0.9 | 1.1 | -0.5 | -0.3 | 1.2 | 1.4 | 0.5 | 0.2 | -1.1 | -2.3 |
| 80 | -2.3 | -2.1 | -0.5 | 0.4 | -0.7 | -1 | 0.9 | 0.8 | 0.1 | 0.1 | -0.3 | 1 | -1.2 | -1 | -0.3 | 0.3 | -0.5 | -1.8 |

| Region of Interest | Posterior cingulate and paracingulate gyri | Posterior cingulate and paracingulate gyri | Precentral gyrus | Precentral gyrus | Precuneus | Precuneus | Rolandic operculum | Rolandic operculum | Superior frontal gyrus, dorsolateral | Superior frontal gyrus, dorsolateral | Superior frontal gyrus, medial | Superior frontal gyrus, medial | Superior frontal gyrus, medial orbital | Superior frontal gyrus, medial orbital | Superior frontal gyrus, orbital part | Superior frontal gyrus, orbital part | Superior occipital gyrus | Superior occipital gyrus |
| --- | --- | --- | --- | --- | --- | --- | --- | --- | --- | --- | --- | --- | --- | --- | --- | --- | --- | --- |
| Side | L | R | L | R | L | R | L | R | L | R | L | R | L | R | L | R | L | R |
| Number 1 | 0.7 | 0.3 | -0.2 | -0.8 | 1.1 | 2.1 | 1 | -0.4 | -0.5 | -2.1 | -2.5 | -2.1 | -1.1 | -0.5 | -1.6 | -1.4 | -0.4 | -1.1 |
| 2 | 0 | -0.8 | -0.8 | -0.7 | -0.4 | -0.9 | -0.9 | -1 | -1.5 | -1.9 | -1.6 | -3.4 | -1 | -0.7 | -1.5 | -1.6 | -0.1 | 0.5 |
| 3 | -0.9 | -0.9 | -0.3 | -1.6 | 2.5 | 0.3 | 0.5 | -2.2 | -1.5 | -3 | -2.8 | -4.4 | -1.6 | -1.2 | -3 | -2.2 | 0 | 0.9 |
| 4 | 1.1 | -0.2 | -0.8 | 0.5 | -0.3 | 0.8 | 0.7 | 0 | -3.2 | -2.2 | -3.8 | -5.3 | -1.9 | -2.3 | -1.9 | -1.9 | 0.2 | 1.8 |
| 5 | -1.6 | -0.8 | -0.4 | -0.9 | 1.1 | 1.2 | -0.6 | -2.1 | -0.1 | -0.5 | -2.7 | -3.5 | -0.9 | -0.1 | -0.3 | -1.5 | -1.5 | -0.9 |
| 6 | 0.5 | 0.8 | 0.3 | 0.8 | 1.2 | 1.2 | 0 | -0.4 | 0.6 | 1 | -0.5 | 0.8 | -0.8 | 0.6 | -1.5 | -1 | -1.1 | 0 |
| 7 | -1.1 | -0.8 | -0.2 | -0.6 | -0.8 | -0.6 | -0.5 | -0.6 | -0.3 | -2.1 | -1.8 | -2.9 | -0.3 | -0.1 | -0.7 | 0.6 | 1.1 | 0.2 |
| 8 | 0.7 | -0.2 | 0.7 | -0.1 | -0.5 | -0.3 | -0.1 | -0.9 | 1.8 | 1.1 | -0.7 | -0.8 | 0.3 | 0.6 | 0.1 | -0.8 | -2.4 | -1.1 |
| 9 | 0.5 | -0.3 | -0.2 | -0.3 | 0.1 | -0.8 | 0 | 1.2 | -0.4 | -2 | -0.9 | -0.9 | -0.2 | 0.5 | -0.3 | -1.5 | -1.7 | -1.7 |
| 10 | 0.5 | 0.8 | -0.9 | -1 | -0.7 | 0.9 | 1 | -1.4 | -1.9 | -1.9 | -2.9 | -1.9 | 0.4 | 0.6 | -2 | -2.1 | -0.9 | 0.4 |
| 11 | -0.9 | 0.1 | -1.7 | 0.4 | -0.2 | 0.4 | -3.4 | -2 | -1.5 | -1 | -1 | -3.5 | 0 | 0.2 | -0.6 | -0.1 | -1.8 | -1.9 |
| 12 | -0.9 | -0.7 | 1.7 | 1.2 | 0.3 | -1.1 | -0.6 | -1.3 | -0.3 | -0.6 | -1.8 | -0.8 | -0.6 | -0.6 | -0.6 | -1.5 | -0.1 | 0.2 |
| 13 | -1.6 | -2.3 | -0.4 | 0.1 | -1.6 | -0.4 | -2.3 | -2.3 | 0.2 | -0.3 | -2.9 | -0.7 | -1.9 | -0.3 | -0.9 | -1.4 | -0.4 | -1 |
| 14 | -0.3 | 0.2 | -0.2 | 0.6 | 0.4 | 1.5 | 0 | 0.5 | -1.8 | -1.3 | -2.9 | -2.6 | -2.8 | -2.7 | -2.3 | -2.4 | -0.9 | 0.3 |
| 15 | -0.9 | -0.5 | -0.6 | -0.8 | 0.4 | 0.3 | -2.7 | -3.4 | -1.4 | -2.3 | -2.8 | -4.1 | -2.8 | -1.9 | -2.4 | -3.6 | -0.1 | -0.5 |
| 16 | 0.6 | -0.4 | 1.9 | 1 | 0.8 | 1.2 | 1.4 | -0.5 | 3 | 1.2 | 0 | -0.8 | 0.4 | -0.1 | -0.1 | -1.2 | -0.7 | -1.8 |
| 17 | -2.9 | -1.6 | 0.1 | -0.9 | -0.1 | 0 | 0.8 | -1.6 | -1 | -1.1 | -3.3 | -2.3 | -1.1 | -0.6 | -2 | -1.6 | -3 | -1.1 |
| 18 | -0.2 | -1 | -0.4 | -0.8 | -0.4 | 0.2 | 0.6 | 0.6 | -2.3 | -1.8 | -1.7 | -2.1 | -0.5 | -1 | -0.5 | -1.4 | -1.1 | -0.2 |
| 19 | 1.7 | 0.8 | -2.4 | -2.8 | -0.4 | 0.2 | -1.4 | -1.8 | -2.6 | -4.1 | -1.8 | -2 | -0.4 | -0.5 | -0.4 | -1.3 | -2.4 | -1.8 |
| 20 | 0.9 | 0.2 | 1.2 | 1.1 | -0.3 | -1.4 | -1.6 | -0.7 | -1.5 | -0.3 | -1.8 | -3.6 | -1.9 | -0.9 | -3.3 | -2.9 | 2.3 | 1.5 |
| 21 | 1.8 | 1 | -0.9 | -1.1 | 2 | 1.3 | 0.2 | -1.1 | -1.5 | -0.8 | -0.7 | -0.8 | -1.6 | -1.7 | -2.5 | -2.1 | -1.7 | -0.6 |
| 22 | 1.9 | 0.5 | -0.3 | -1.4 | 0.4 | 1.5 | 0.4 | -1.7 | -0.8 | -1 | -1.3 | -1 | -1.5 | 0 | -0.3 | -0.1 | -1 | -0.1 |
| 23 | 1.6 | 1.1 | -0.9 | -0.2 | 2.4 | 1.2 | 0.4 | -1.5 | -0.2 | -1.9 | -3 | -3.7 | -2.8 | -2.5 | -1.4 | -2.3 | 0.7 | -0.2 |
| 24 | -0.9 | 0.8 | -0.5 | -2 | 1.2 | 0.4 | -0.1 | -1.6 | 0.6 | 0.3 | -0.8 | -0.8 | -0.9 | -1.5 | -0.2 | -0.9 | -3.1 | -1.9 |
| 25 | -0.2 | 0.2 | 1.8 | 0.4 | 0.3 | 0.8 | 1.4 | -0.9 | 1.2 | -0.9 | -0.2 | -0.6 | 0.3 | -0.1 | 1.1 | -0.2 | -2.3 | -2.3 |
| 26 | -0.9 | -0.8 | 0.6 | 0.3 | 0 | -1.4 | 0.6 | -0.8 | -0.1 | -2 | -1.2 | -1.9 | -1.5 | 0 | -0.1 | -1.7 | -0.9 | -1.1 |
| 27 | 0.5 | 0.6 | 0.1 | -1 | -1 | -0.6 | -0.2 | -2.1 | -0.6 | -2.5 | -2.5 | -3.8 | -2.3 | -2.1 | -1.3 | -1.2 | 0 | 0.1 |
| 28 | -1.8 | -1.5 | 0.2 | 0.3 | -0.9 | -0.3 | -3.7 | -2.1 | 0.2 | -0.2 | -0.7 | -1.9 | -1.5 | -1.6 | -1 | -0.4 | 0.1 | 1 |
| 29 | -2.4 | -2.2 | -0.3 | 0.8 | -0.9 | -0.7 | -1.5 | 0.5 | -0.6 | -2.5 | -2 | -2.4 | -2.3 | -1.2 | -1.5 | -2 | -1.6 | -1.1 |
| 30 | -0.7 | 0.1 | -1.3 | -1.1 | 1.6 | 1 | -0.7 | -0.7 | 0.6 | -1.4 | -1.3 | -1.6 | 0.4 | -1.1 | 0.4 | -0.9 | -1.1 | 0.1 |
| 31 | 0.3 | -0.7 | -0.6 | -1.2 | 0.2 | 0.7 | -1.5 | -1.7 | 0 | -1.9 | -0.7 | -2.1 | -1.2 | -1.6 | -1.8 | -2.8 | -1 | -0.4 |
| 32 | -3.3 | -3.2 | 0.4 | -1.4 | -1.4 | -2.1 | 0.1 | -0.7 | 0.5 | -0.8 | -1.3 | -2 | -1.2 | -0.9 | -0.1 | -0.8 | 0.5 | 1.4 |
| 33 | -0.5 | -0.5 | 0.7 | 0.9 | -0.5 | -1.8 | -1.4 | -0.9 | -0.2 | -2.2 | -1.1 | -2.8 | -2.5 | -2.7 | -2.1 | -3.1 | 2.2 | 1.1 |
| 34 | -0.8 | -1 | -0.7 | -1.4 | 0.4 | 0.6 | -1 | -1.3 | -2.5 | -3.7 | -3.4 | -5.1 | -1.9 | -1.4 | -0.6 | -3.4 | 0.5 | 0 |
| 35 | -1.5 | -0.6 | 0.1 | -2 | -0.3 | 1.5 | -0.7 | -2.3 | 0.6 | -2.1 | -1.2 | -2.9 | -1.2 | -1.4 | -1.9 | -2.7 | -1.5 | 0.4 |
| 36 | 0.7 | 0.9 | 0.6 | -0.5 | 0.3 | 0.5 | -0.1 | -1.4 | -0.3 | -0.7 | -0.9 | -2.2 | -0.4 | 0.4 | 0.2 | -0.4 | -1.8 | 0.2 |
| 37 | -1 | -1 | -1 | -0.8 | 1.2 | -0.4 | -0.9 | -1.2 | -1 | -2.9 | -2.1 | -3.8 | -1.9 | -1.3 | -1.7 | -1.8 | 1.5 | 2 |
| 38 | 0.3 | 0.3 | -0.5 | -0.8 | 0.6 | -0.2 | -3 | -1.8 | 0.2 | -1.3 | -1.7 | -2.1 | -2 | -2.4 | 0.2 | -1.6 | 0.1 | -0.1 |
| 39 | -2.8 | -1.8 | 1.5 | 1.1 | -0.8 | -2.6 | -0.4 | -0.9 | 0.8 | -0.9 | -0.9 | -0.7 | -0.4 | -0.3 | -0.8 | -1.9 | 0 | 0.8 |
| 40 | -0.8 | -0.6 | -0.2 | 0.1 | 2.2 | 2.3 | -1.7 | -0.9 | 0.3 | -0.5 | 0.1 | -0.6 | 0.1 | -0.3 | -0.3 | -1 | -1.2 | -1.4 |
| 41 | 0.9 | 0.4 | 1.3 | -0.1 | -0.1 | 0.6 | 0.6 | -0.1 | 0.5 | -1 | -1.1 | -2 | 0.1 | -0.4 | -1.4 | -1.8 | -1.9 | 0.2 |
| 42 | 1 | -0.2 | -0.6 | -2.4 | -0.3 | -0.2 | -0.3 | -2.7 | 0.1 | -1.6 | -1.3 | -1.9 | -0.9 | -1.3 | 0.4 | 0.5 | -1.8 | 0.3 |
| 43 | 2.2 | 2.5 | -0.6 | -1.2 | 1 | 1.6 | -0.6 | -1.9 | -0.5 | -2 | 0.9 | -0.9 | -0.4 | 0 | 0.1 | -0.7 | -1.5 | 0.2 |
| 44 | -1.8 | -1.8 | -0.8 | -0.6 | -0.8 | -1.2 | -0.1 | -0.1 | -3.6 | -2.3 | -1.7 | -2.5 | -0.4 | -1.2 | -1.2 | -1.9 | -0.4 | 1.9 |
| 45 | -0.1 | 0.9 | 1.1 | 0.6 | 0.9 | 1.8 | -0.5 | -1.3 | 1.4 | -0.1 | -0.1 | 0.2 | -0.3 | -1 | 0 | -0.6 | -0.2 | -0.8 |
| 46 | -0.5 | -0.7 | 0.5 | 0.6 | 0.6 | 0.5 | -2.8 | -2.9 | 0.2 | 0.4 | -2.4 | -1.7 | -1.3 | -1.8 | -1.3 | -2.3 | -0.5 | -0.2 |
| 47 | -0.4 | 0.2 | -0.1 | -1 | 1.1 | 1.5 | -0.5 | -2 | 1.1 | -1.6 | -0.9 | -2.7 | -0.5 | -1.7 | 0.1 | -1.3 | -2 | -1.6 |
| 48 | -1.3 | -0.6 | -0.1 | -0.6 | 0.3 | 0.5 | -1.3 | -1 | -0.4 | -1.4 | -1.7 | -0.9 | -0.5 | -0.4 | -1.9 | -1.1 | -1.6 | -0.1 |
| 49 | -0.4 | -0.1 | 0.9 | -1.4 | -0.3 | -0.2 | -2.2 | -1.1 | -0.1 | -1.2 | -1.2 | -1.7 | -1.1 | -0.7 | -0.7 | -2.4 | -0.7 | -1.4 |
| 50 | -0.7 | -0.2 | -0.6 | -1.3 | 0.6 | -0.7 | -1.6 | -2.1 | -0.3 | -2 | -0.5 | -2.7 | 0.3 | -0.2 | -1.2 | -2.3 | -1.9 | -0.5 |
| 51 | -1.2 | -0.1 | 1.1 | 1.3 | 0.4 | 1.1 | -0.2 | -1.5 | -0.3 | -0.3 | -0.2 | -0.5 | -0.9 | -1.1 | -1.3 | -1.7 | -1.2 | 0.7 |
| 52 | -0.7 | -0.9 | 0.1 | -1.6 | 1.1 | 1.4 | -0.2 | -0.6 | -3.5 | -2.9 | -1.5 | -2.4 | -0.4 | -2.1 | 0.1 | -1.9 | -0.1 | -0.1 |
| 53 | 0.8 | 1.2 | -1.8 | -1.6 | 0.4 | 1.7 | -0.9 | -1.4 | -1 | -2.8 | -1 | -0.7 | -0.7 | -1.2 | -0.8 | -2.2 | -2.7 | -2 |
| 54 | -2.4 | -2.3 | 0.7 | 0 | 0.1 | 0.3 | 0.3 | -0.8 | -0.4 | -1.5 | -0.7 | -2.2 | -0.5 | -1.7 | 0 | -0.1 | 0.9 | 1.5 |
| 55 | -0.6 | -0.4 | 0.5 | -0.5 | 1.5 | 0.9 | -0.1 | -2 | -0.6 | -1.7 | -1.8 | -2.9 | -2 | -0.9 | -0.5 | -0.9 | -1.2 | 1.2 |
| 56 | 1.2 | -0.1 | -0.3 | 0.4 | 0.1 | 1 | -0.7 | -0.4 | -0.8 | -0.6 | -1.1 | -0.6 | -1 | -0.4 | -0.5 | -1.1 | -0.2 | 0.8 |
| 57 | -2.5 | -2 | 0.3 | -0.6 | -1.4 | -1.7 | 0.3 | -0.7 | 0 | -1.4 | -1.6 | -3.3 | -0.2 | -0.8 | -0.8 | -0.9 | -0.3 | 1.2 |
| 58 | 0.6 | 1 | 0.8 | 1.1 | 0.4 | 0.7 | -1.1 | -0.8 | -0.4 | -1.7 | -1.3 | -2 | -0.2 | -0.8 | -0.7 | -1.3 | -0.2 | -0.1 |
| 59 | -2.2 | -1.6 | 0.4 | -1 | 1.8 | 1.8 | -0.1 | -1.3 | -0.4 | -1.4 | -1.6 | -3.3 | -0.7 | -1.3 | -1.4 | -1.7 | -0.4 | 0.2 |
| 60 | -1.2 | 0.4 | 1 | 0.1 | -1 | 1.4 | -0.7 | -2.1 | -1 | -0.6 | 0 | -0.9 | -1.9 | -1.2 | -1.9 | -1.6 | -0.5 | 1 |
| 61 | -1.1 | -1.2 | 0.3 | 0.7 | 1.4 | 2 | 0 | 1 | 0.8 | -0.3 | -0.7 | -1.8 | -0.9 | -0.8 | 0.1 | -0.7 | -0.7 | -1 |
| 62 | 2.6 | 2.6 | -0.8 | 0 | 1.3 | 1.2 | -1.1 | -1.8 | -0.9 | -0.8 | -3.1 | -2.7 | -2.6 | -2 | -1.5 | -1.5 | -1.2 | -0.2 |
| 63 | -1.5 | -0.3 | -0.9 | -1 | 1.8 | 1.5 | -1 | 0.7 | 0.9 | -1.2 | -2.1 | -1.1 | -1.4 | -1.4 | -0.4 | -1.6 | -1.8 | 0 |
| 64 | 1.2 | 1.5 | -0.1 | -1 | 1.8 | 0.3 | -0.6 | -0.1 | 0.2 | -1.5 | -0.4 | -2.1 | -1 | -2.1 | -1.6 | -2.3 | 0.1 | 0.7 |
| 65 | -2.1 | -2.1 | 0.1 | -0.9 | -0.4 | -0.6 | 0.2 | -1.4 | 0.1 | -1.8 | -1.8 | -2.3 | -0.5 | 1 | -0.6 | -2.6 | -0.3 | 0 |
| 66 | -1.2 | -0.7 | -0.8 | -0.1 | -0.6 | -0.3 | -0.9 | -1.5 | 0.9 | -0.1 | -2.6 | -4.1 | -2.1 | -1 | -0.8 | -0.6 | -1.7 | 0.2 |
| 67 | -1 | 0 | -0.5 | -0.9 | 0.5 | 0.1 | -0.3 | -0.9 | -0.8 | -2.1 | -2.5 | -3.3 | -1 | -1.1 | -0.4 | -2.1 | -1.8 | -1 |
| 68 | -0.3 | 0.3 | 1.3 | 1.3 | -0.8 | 0.3 | -0.6 | 0.5 | 0.5 | 0.8 | -0.2 | 0.2 | -1.5 | -1.7 | -2.7 | -1.6 | -0.9 | 0.8 |
| 69 | 0.3 | -0.7 | -0.5 | -0.3 | 0.9 | 1.7 | -0.9 | 0 | 0.7 | -1.9 | -2.2 | -5.8 | -1.1 | -1.5 | -0.6 | -1.5 | -0.4 | 1.4 |
| 70 | 0.1 | 0.1 | 0.4 | -0.3 | 0.5 | 1.4 | 0.9 | 1 | -0.4 | -0.9 | -1.7 | -1.6 | -1.1 | -0.7 | -2.7 | -1 | -0.5 | 0 |
| 71 | -0.1 | 0.1 | -0.7 | -1.5 | 0.6 | 1 | -0.2 | -0.8 | -0.6 | -2.6 | -2.4 | -2.8 | -0.2 | 0.1 | 0.6 | -0.2 | -1.8 | 1 |
| 72 | 0.4 | -1.2 | 0.4 | -0.8 | -0.8 | -1.1 | -1.2 | -1.5 | -0.7 | -2.4 | -0.5 | -0.9 | -0.9 | -0.6 | -0.9 | -1 | 0 | -1 |
| 73 | 0.7 | 0.3 | 0.3 | -1.4 | 0.5 | 0.2 | -0.4 | -1.4 | -0.5 | -2.1 | -1.9 | -1.8 | -0.4 | -0.6 | 0.1 | -1.6 | -2.2 | 1.2 |
| 74 | 0.5 | 1.8 | 0.3 | -0.7 | -0.6 | 0.1 | -0.7 | -1.2 | 0.7 | -1.3 | -1.5 | -1.5 | -0.2 | 0 | -1 | -0.9 | -1.1 | 0.2 |
| 75 | -0.7 | -0.3 | 0.2 | 0.2 | 0.3 | 1.9 | -0.2 | 0.5 | 0.1 | 1.4 | 0 | -0.1 | -0.7 | -0.4 | -1.1 | -0.4 | -0.1 | 0.4 |
| 76 | 0.3 | -0.1 | -0.4 | -0.5 | 1.2 | 2 | 0.3 | -0.9 | 0 | -0.3 | -0.8 | -2.2 | -0.4 | -0.8 | -1.5 | -0.4 | -1.1 | 0.7 |
| 77 | 1 | 1.7 | -0.8 | 0 | -1.4 | 0.1 | -2.4 | -2 | -0.3 | 0.8 | -1.2 | -0.5 | -2.3 | -1.3 | -0.7 | -0.1 | -2.3 | -0.1 |
| 78 | 1.3 | 1.5 | 0 | 0.2 | 0.8 | -0.1 | -1 | 0 | -0.9 | -0.1 | -2.6 | -2.1 | -2.3 | -1.3 | -2.3 | -1.4 | -0.6 | 0.4 |
| 79 | 0.5 | 1.1 | -0.4 | -0.2 | 1.8 | 1.6 | 0.3 | -0.7 | -1.1 | -0.7 | -1.9 | -1.2 | -0.5 | -0.6 | -0.7 | -0.3 | 0.1 | -1 |
| 80 | -0.2 | -0.3 | 0.4 | 0.6 | 1.1 | 1.5 | -1 | -1.7 | -0.5 | -1.3 | -1 | -1.9 | -2.1 | -1.9 | -2.6 | -2.4 | -0.7 | -0.2 |

| Region of Interest | Superior parietal gyrus | Superior parietal gyrus | Superior temporal gyrus | Superior temporal gyrus | Supplementary motor area | Supplementary motor area | Supramarginal gyrus | Supramarginal gyrus | Temporal lobe | Temporal lobe | Temporal pole: middle temporal gyrus | Temporal pole: middle temporal gyrus | Temporal pole: superior temporal gyrus | Temporal pole: superior temporal gyrus | Thalamus | Thalamus |
| --- | --- | --- | --- | --- | --- | --- | --- | --- | --- | --- | --- | --- | --- | --- | --- | --- |
| Side | L | R | L | R | L | R | L | R | L | R | L | R | L | R | L | R |
| Number 1 | 0.2 | -0.5 | -1.6 | -0.5 | -0.2 | 0.7 | -1 | -1.9 | -2.5 | -1.4 | -0.3 | -0.7 | 0.2 | -0.5 | 2.5 | 1.9 |
| 2 | -1.2 | -1 | -1.1 | -1.2 | -0.8 | -0.4 | -3.2 | -2.1 | -1.8 | -1.4 | -0.6 | 0.1 | -1 | -0.2 | 2.5 | 1.8 |
| 3 | -0.5 | -2.6 | -1.9 | -3.7 | -0.3 | -0.3 | -2.1 | -3.3 | -2.3 | -3.3 | -2.2 | -1.6 | -0.9 | -1.6 | 1.7 | 0.8 |
| 4 | -2.1 | -1.9 | 0 | 0.9 | -1.6 | -1.7 | -3.4 | -1.9 | -3.5 | -1.6 | -2.4 | -1.5 | -2.7 | -1.2 | 3 | 1.9 |
| 5 | -0.4 | 0.1 | -2.2 | -2.3 | -0.7 | 0.2 | -2.1 | -1.4 | -2.5 | -2.8 | -2 | -1.1 | 0.3 | -0.9 | -1.1 | -0.9 |
| 6 | -0.2 | 0.6 | -0.8 | -1.7 | 2.1 | 2 | -1.4 | -3.1 | -2.5 | -1.6 | -1.1 | 0.4 | -1.2 | -1 | 1.7 | 1.3 |
| 7 | -1.2 | -1.1 | -1.2 | 0.4 | 0.3 | -0.5 | 0.1 | -1.5 | -1.3 | -0.1 | -1.4 | -0.5 | -1.6 | -1.4 | 1 | 1.3 |
| 8 | -0.5 | -1 | -0.2 | 0.5 | 2 | -0.5 | -2.1 | -2.8 | -1.5 | -1.2 | -0.6 | 0.6 | -0.2 | 0.5 | -0.7 | -1 |
| 9 | -1 | -1.8 | -0.9 | -1.6 | -0.2 | 0.1 | -3 | -2.3 | -2.4 | -2.1 | -1.2 | -1.4 | -0.1 | -0.4 | 0.6 | 0.8 |
| 10 | -0.2 | -0.1 | -0.2 | -0.9 | -1.4 | -2 | -0.3 | -0.6 | -0.5 | -0.7 | -0.1 | 0.9 | 0 | -0.1 | 0.1 | 0.2 |
| 11 | -2.1 | -0.5 | -2.1 | 0.2 | -0.2 | -0.2 | -4.2 | -2.4 | -2.8 | -1.5 | -0.9 | 0.4 | -1.1 | -1 | 1.9 | 1.5 |
| 12 | -0.4 | -1.1 | 0.5 | 0.2 | -0.8 | -0.7 | 0.5 | -1.4 | -0.9 | -1.6 | -1.6 | -1.7 | -1.1 | -1.7 | 2.1 | 1.2 |
| 13 | -1.8 | -2.5 | -1.9 | -1 | 0.6 | 0.5 | -2.8 | -2.9 | -4.1 | -2.3 | -2.2 | -1.9 | -2 | -1 | 0.4 | 0.2 |
| 14 | -1.3 | -0.9 | 0.5 | 0.6 | -1.1 | 0 | -3.6 | -2.6 | -1.9 | -1 | -2.4 | -1.2 | -1.3 | -0.9 | 0.8 | 0.5 |
| 15 | -1.3 | 0.2 | -2.2 | -3.5 | 0.7 | 1 | -5 | -4.9 | -4.5 | -3.7 | -0.8 | -0.3 | -1.9 | -0.4 | 0 | -0.3 |
| 16 | -0.1 | 0.8 | 0 | -1.8 | 1.6 | 0 | -0.6 | -1.8 | -1.2 | -1.7 | 0.4 | 0.6 | 0.4 | 1 | 2.2 | 1.1 |
| 17 | -0.4 | -0.5 | 0.5 | -0.7 | 0 | -0.4 | -0.4 | -0.9 | -2.4 | -1.2 | -3.6 | -3.2 | -1.1 | -1.2 | -0.3 | -1.5 |
| 18 | -0.7 | -1 | -1.5 | -0.7 | 0.5 | -0.3 | -2.3 | -1.6 | -2.4 | -1.6 | -1.9 | -1.6 | -0.2 | -0.9 | 1.1 | 1.4 |
| 19 | -2.1 | -2.1 | 0 | 1.8 | -2.4 | -3.4 | -2.8 | -2.5 | -0.1 | 0 | 0.8 | 0.5 | 0.4 | 0.7 | 0.7 | 0.5 |
| 20 | 0.4 | -1 | -1.7 | -1.4 | 0.5 | 0.1 | -2.7 | -2.4 | -2.8 | -2.3 | -0.3 | 0.3 | 0.5 | -0.8 | 2.4 | 2.3 |
| 21 | -1.9 | -2 | 0.2 | -0.7 | 2.1 | 1.5 | -0.6 | -1.9 | -1.5 | -1.4 | -0.2 | -0.4 | -1.5 | 0 | 2.2 | 1.3 |
| 22 | -0.3 | -0.6 | -0.9 | -2.5 | -0.4 | -0.4 | -1.2 | -1.2 | -2.1 | -2 | -0.7 | -0.1 | -0.4 | -0.1 | 0.1 | -0.1 |
| 23 | 0.3 | 0 | -1.8 | -2.7 | 1.2 | -0.3 | -2.1 | -2.9 | -3.2 | -2.6 | -2.2 | -1.8 | -2.5 | -0.7 | 2.1 | 0.2 |
| 24 | -0.5 | 0.2 | -0.5 | -2.2 | 1.1 | 0.2 | -0.9 | -4.2 | -0.8 | -2.1 | -0.4 | -0.7 | 0.4 | 0.4 | 0.1 | -1.1 |
| 25 | -1.9 | -1.7 | 2.4 | 0.9 | 0.8 | 1 | 0.7 | -1.7 | 1 | -0.5 | -0.7 | 0.4 | 0.3 | 0.8 | 1.2 | 0.9 |
| 26 | -1.1 | -1 | 1.4 | 2.9 | -0.1 | -1.1 | -2.3 | -3.8 | -0.3 | -0.2 | -1.5 | -0.4 | 0.3 | 0.9 | 2.4 | 1.1 |
| 27 | -0.9 | -0.8 | -0.3 | -2.3 | -0.3 | -0.6 | -3.7 | -3.9 | -1.8 | -2.8 | -0.9 | -0.5 | -0.4 | -0.3 | 2.3 | 2 |
| 28 | -0.5 | -2.2 | -1.4 | 0.1 | 1.2 | 0.6 | -1 | -1.1 | -2.4 | -0.6 | 0.2 | 0.2 | -0.2 | 0.6 | 2.1 | 2.3 |
| 29 | -1.4 | -1.6 | -2.3 | -0.5 | -0.5 | -0.7 | -2.5 | -3 | -2.8 | -1.7 | -0.5 | 0 | -0.6 | 0.2 | -2.9 | -3.6 |
| 30 | 0 | -0.4 | 0.7 | 0.2 | -0.6 | 0.4 | -0.4 | -0.8 | 0.1 | -0.6 | -0.9 | 0 | -0.3 | -0.3 | -0.6 | 0.1 |
| 31 | -0.7 | -1.3 | -1.8 | -2.4 | 0.8 | 0 | -2.3 | -2.5 | -2.7 | -2.1 | -1.2 | -0.9 | -0.6 | -0.7 | 1.3 | 1.3 |
| 32 | -1.7 | -2 | -0.8 | -1.2 | -2 | -2 | 0.6 | -2 | -0.7 | -1 | -1 | -0.5 | -1.2 | -0.8 | 1.8 | 1.4 |
| 33 | 0.8 | 0.7 | -0.7 | -0.6 | -0.1 | -0.3 | -1.8 | -3 | -1.5 | -0.5 | -0.6 | 0.4 | -0.3 | 1 | 2.6 | 1.5 |
| 34 | -2 | -2.7 | -1.4 | -1 | -1.5 | -2.5 | -0.4 | -1.6 | -0.8 | -1.4 | -2 | -0.8 | -1.4 | -1 | 2.3 | 1.2 |
| 35 | 0.7 | -1.2 | -1.7 | -2 | -0.7 | -0.7 | -3 | -3.5 | -1.1 | -1.8 | -1.4 | -1.2 | -0.1 | -1.6 | -1.3 | -1.1 |
| 36 | -1.5 | -0.5 | -1.2 | -2.5 | 0.3 | 0.2 | -0.4 | -1.8 | -1.9 | -1.9 | -1.3 | -0.6 | 0.6 | 0.4 | 1.3 | 0.6 |
| 37 | -1 | -2 | 0.8 | 0.5 | -2.6 | -3.4 | -2.1 | -2.1 | -1.1 | -1.4 | -0.4 | -0.2 | -1 | -0.4 | 1.5 | 0.7 |
| 38 | -0.7 | -0.6 | -0.9 | 0.1 | 0.2 | -0.6 | -2.3 | -3.1 | -1.7 | -1.3 | -0.9 | -0.6 | 0.5 | 0.2 | -0.1 | -0.3 |
| 39 | 0.3 | 0 | -1.4 | -1.2 | 0.8 | 0.1 | -0.9 | -1.3 | -2 | -1.9 | -1 | -1.6 | -1 | -1.1 | 1.4 | 1.1 |
| 40 | -0.5 | 0.8 | -2.6 | -1.1 | 1.4 | -0.3 | -2.4 | -2.5 | -3.7 | -1.7 | -1.6 | -1.2 | -1.8 | -0.2 | 0.5 | 0.5 |
| 41 | -0.6 | -0.6 | -2 | -0.2 | 0.9 | -0.5 | -2.5 | -2.9 | -2.8 | -1.9 | -1.6 | -1.2 | -0.8 | -1.9 | -0.7 | -0.4 |
| 42 | -0.4 | -0.7 | -0.9 | -1.7 | 0 | 0.2 | -1.4 | -2.5 | 0.3 | -2 | -0.5 | -0.5 | -1 | -1.4 | 1.4 | -0.3 |
| 43 | 0.2 | 0.2 | -1.8 | -1.9 | 1.1 | 0.4 | -1.4 | -2.9 | -1.7 | -1.8 | 0.3 | 0.4 | 1.8 | 1.3 | 1.1 | 0.5 |
| 44 | -0.2 | -1.8 | -0.3 | 1.4 | 0.4 | 0.4 | -1.4 | -0.1 | -0.9 | -0.3 | -0.9 | -0.3 | -2.5 | -0.6 | 0.7 | 0.3 |
| 45 | -0.2 | 0.8 | -0.7 | -0.9 | 1.4 | 1.4 | 0 | -1 | -0.7 | -1 | 0.4 | 0.4 | 0.4 | 0.2 | 1.8 | 1.4 |
| 46 | -0.9 | -0.7 | -3.1 | -1.5 | 1.3 | 1.9 | -4.6 | -1.3 | -3.5 | -1.9 | -2.5 | 0.3 | -3.2 | -0.3 | 0.4 | -0.5 |
| 47 | -0.9 | -1.2 | -1.6 | -0.6 | 0.4 | -1.6 | -0.6 | 0.2 | -1.2 | -0.5 | -1.5 | -0.8 | -0.2 | -0.2 | -1.1 | -0.9 |
| 48 | -1.2 | -0.8 | 1.1 | -0.9 | 0.3 | 1.2 | -1.6 | -1.7 | -2 | -0.7 | -1.7 | -1 | -1.7 | -0.8 | -1.1 | -0.5 |
| 49 | 0.4 | -0.3 | -1.3 | -2.5 | -0.1 | -0.3 | -2.7 | -3.1 | -2.2 | -2.4 | -0.5 | -0.7 | -0.3 | -0.1 | 1.3 | 1.1 |
| 50 | -0.7 | -2.4 | -3.2 | -3 | 0.1 | -1.1 | -1.6 | -3.4 | -3.1 | -3.1 | -1.4 | -1.2 | -1 | -1.3 | -1.6 | -0.7 |
| 51 | -0.4 | 0.1 | -1.5 | -0.9 | 1.4 | 1.3 | -1.3 | -1.5 | -2 | -1.6 | -1.8 | -1.5 | -1.5 | -0.5 | 0.4 | 0.1 |
| 52 | -0.5 | -0.7 | -1.7 | 0.1 | 1.6 | 0.7 | 0.4 | -1.5 | -1.1 | -1.1 | -0.4 | -0.7 | 0.2 | -0.4 | 1.3 | 1.1 |
| 53 | -0.5 | -0.5 | -1 | -1.6 | 0.1 | -0.7 | -3.5 | -1.9 | -1.8 | -1.3 | -1.4 | -0.9 | -1 | -0.8 | -1.5 | -1.2 |
| 54 | 1.3 | -0.1 | -0.6 | -1.6 | -0.1 | -0.4 | -0.2 | -2 | -0.8 | -1.2 | -1.3 | -0.7 | 0.2 | -0.4 | 0.9 | 1.5 |
| 55 | 2 | 1 | -1.5 | -2.3 | 0.7 | 1.9 | 0 | -1.4 | -2.1 | -1.1 | -1 | 0.3 | -1.7 | -0.3 | 1.7 | 1.2 |
| 56 | -1.6 | -0.8 | -0.8 | 0.6 | 2.7 | -0.1 | -2.7 | -0.9 | -0.8 | 0 | -0.7 | 0.2 | -0.3 | 1.2 | 0.4 | -0.5 |
| 57 | 0.6 | -0.5 | 3.9 | 1.6 | 0.2 | -1.4 | -0.3 | -0.6 | 1.7 | -0.7 | -0.5 | 0.2 | 1.3 | 0.9 | 1.4 | 1.4 |
| 58 | -0.5 | 0 | -1.4 | -1.6 | 1.9 | 0.5 | -2.7 | -2.4 | -2.3 | -1.1 | 0 | -0.1 | 1.8 | 0.4 | 2.2 | 2.7 |
| 59 | 0.1 | 0.4 | -1.2 | -0.2 | 0 | -0.2 | -1.2 | -3 | -1 | -1.1 | -0.4 | 0.2 | -0.6 | -0.5 | -1.1 | -0.8 |
| 60 | -0.1 | 0.7 | 1.9 | -0.2 | 0.5 | 0.6 | -2.2 | -2.5 | -0.9 | -1.2 | -0.2 | 0.5 | 0.1 | 0.4 | 1.5 | 0.5 |
| 61 | -0.5 | -1 | 0.6 | 1.9 | 1.6 | 0.8 | -1.6 | -1.7 | -1.5 | -0.4 | -1.6 | 0 | -0.2 | 0.7 | 0.8 | 1.1 |
| 62 | -1.6 | -1.1 | 0 | -0.6 | -1.6 | 1.1 | -4.8 | -2.5 | -2.3 | -2 | -1.4 | -0.4 | -1.1 | -0.3 | 1.9 | 1.2 |
| 63 | -0.3 | -1.1 | -1.9 | -2.2 | -1 | -0.3 | -0.7 | -1.8 | -2.4 | -1.9 | -1.4 | 0.1 | -1 | -0.4 | 1.5 | 1.8 |
| 64 | -1.1 | -1.3 | -0.4 | 0.9 | 0.4 | -0.1 | -2.1 | -1.6 | -1 | 0.1 | -1 | 0.3 | -0.2 | 0.9 | -0.2 | 1 |
| 65 | -0.6 | -0.2 | -0.4 | -1.7 | -0.8 | -1 | -1.6 | -2.9 | -2.3 | -2.2 | -2.1 | -0.6 | -1.1 | -2 | 2.7 | 2.4 |
| 66 | -0.6 | -0.1 | -3.3 | -2.5 | -0.6 | -1.1 | -1.8 | -3.1 | -3.2 | -2.1 | -1.8 | -0.4 | -1.5 | -1.2 | 1.3 | 1.8 |
| 67 | -1.2 | -1.7 | -2.3 | -3 | 1.6 | -0.2 | -2.7 | -5.1 | -3.7 | -2.9 | -0.5 | -0.7 | -1.3 | -1.4 | 1.7 | 1.9 |
| 68 | 0.1 | -0.3 | -1.8 | -0.7 | 2 | 2.7 | -2.2 | -1.1 | -3.5 | -1.9 | -2.4 | -1.8 | -1.2 | -0.1 | 2.4 | 2.1 |
| 69 | -0.7 | 0.4 | 0.2 | 0.2 | -0.1 | -0.9 | -0.6 | -1.8 | -1.5 | -1 | -1.1 | -0.4 | -0.1 | -0.5 | -0.7 | -0.5 |
| 70 | 0.7 | 1 | -0.4 | -2.3 | 0 | 0.4 | -2.7 | -1.3 | -2.1 | -2.3 | -1.3 | -0.5 | 0.3 | -0.1 | -1 | 0.4 |
| 71 | -0.3 | -0.3 | -1 | -0.1 | -2.7 | -3.1 | -0.9 | -1 | -0.1 | -0.3 | -0.7 | -0.3 | -1.7 | 0 | 0.8 | 1 |
| 72 | 0.3 | -0.8 | -2.9 | -2.6 | 1.1 | -0.7 | -4 | -4.9 | -2.7 | -2.7 | -0.5 | -0.2 | 0.2 | -0.7 | 0.1 | -0.6 |
| 73 | 0.4 | 0.6 | 0.1 | -0.4 | -0.9 | -1.7 | -2.7 | -1.9 | -0.5 | -0.1 | 0 | 0.1 | 0.4 | -0.1 | 3.4 | 2.7 |
| 74 | 0.4 | -0.2 | -0.1 | -0.9 | 0.1 | -1.4 | -0.5 | -0.8 | -1.5 | -2.2 | -1 | -0.1 | -0.4 | 0.9 | 0.3 | 0.1 |
| 75 | -0.6 | 0.9 | -1 | -1.1 | 0 | 1 | -2.4 | -1.3 | -2 | -1.2 | 0 | 0.8 | 1 | 1.2 | -0.1 | 0 |
| 76 | 0.8 | -0.9 | 0.5 | 0.8 | -0.4 | 0.3 | -2.2 | -1.3 | -1.8 | -0.6 | 0.2 | -1 | 0.3 | -0.1 | 0.4 | 0.8 |
| 77 | -1.9 | -0.8 | -2.7 | -0.7 | -0.3 | -0.1 | -1.7 | -0.9 | -2.5 | -1.3 | -0.9 | -0.7 | -0.9 | -0.6 | 0.7 | 1 |
| 78 | 0.3 | 0.8 | -0.9 | -0.6 | 0.5 | -1.2 | -1 | -1.8 | -2.7 | -1.3 | -1.2 | 0.4 | -1.1 | 0.7 | 0.4 | 0.9 |
| 79 | -0.4 | -0.3 | -1.2 | -0.6 | 0.1 | 0.4 | 1.6 | -0.8 | -1.6 | -0.2 | -0.1 | -0.1 | -1 | 0.3 | -0.3 | 0.1 |
| 80 | -0.2 | 0.1 | -0.9 | -0.9 | 0.7 | 1.2 | -2 | -0.8 | -0.9 | -1.2 | 0.2 | -0.1 | 0.5 | 0 | 0.2 | 0.9 |

Abbreviation: SUV_meanstd_: standard deviation of the SUV_mean_; AAL: automated anatomical labelling.

Supplementary Table 12 The SUV_maxstd_ of normal 45-70 years old group according to AAL standard

| Region of Interest | Amygdala | Amygdala | Angular gyrus | Angular gyrus | Anterior cingulate and paracingulate gyri | Anterior cingulate and paracingulate gyri | Basal ganglia | Basal ganglia | Calcarine fissure and surrounding cortex | Calcarine fissure and surrounding cortex | Caudate nucleus | Caudate nucleus | Central region | Central region | Cingulate and paracingulate gyri | Cingulate and paracingulate gyri | Cuneus | Cuneus |
| --- | --- | --- | --- | --- | --- | --- | --- | --- | --- | --- | --- | --- | --- | --- | --- | --- | --- | --- |
| Side | L | R | L | R | L | R | L | R | L | R | L | R | L | R | L | R | L | R |
| Number 1 | 1.8 | 1.5 | 1.2 | 1.7 | 1.3 | 1.7 | 2.7 | 2.1 | 3.3 | 4.7 | 2.5 | 1.7 | 2.5 | 2.6 | 3.8 | 4.3 | 3.1 | 3.4 |
| 2 | 1.8 | 1.1 | 0.8 | 2.7 | 1.9 | 2.3 | 3.5 | 2.6 | 4.5 | 5 | 3.5 | 2.6 | 2.4 | 3.5 | 5 | 4.2 | 7.2 | 7.3 |
| 3 | 1.2 | 0.1 | 1.8 | 2 | 1.1 | 1 | 2.4 | 0.8 | 5.8 | 5.1 | 1.2 | 0.8 | 4.1 | 2.9 | 4.6 | 2.9 | 4.9 | 4.3 |
| 4 | 0.9 | 1.2 | 0.1 | 1.3 | -0.1 | 0.1 | 3.1 | 4 | 5.8 | 6.1 | 2.1 | 1.1 | 2.9 | 3.5 | 2.5 | 3.5 | 5.8 | 5.8 |
| 5 | -0.6 | -1.9 | 3.3 | 3.7 | 0.5 | 0.3 | 1 | 0.5 | 4.4 | 2.1 | 0.3 | 0.2 | 4.1 | 2.5 | 4.1 | 3.4 | 5 | 2.8 |
| 6 | 1.6 | 1.5 | 0.9 | 2.1 | 3.6 | 2.7 | 3 | 2.6 | 2.8 | 3.1 | 3 | 2 | 2.8 | 2.7 | 4.6 | 4.9 | 2.9 | 3.9 |
| 7 | -0.9 | -0.5 | 3.8 | 3.7 | 1.2 | 0.9 | 0.9 | 0.8 | 4 | 4.4 | 0.5 | 0.5 | 3.8 | 2.3 | 2.6 | 2 | 2.8 | 2.9 |
| 8 | 0.7 | 0.5 | 2.7 | 1.5 | 2.9 | 1.9 | 2.4 | 2.8 | 2.9 | 4 | 1.9 | 1.4 | 4.4 | 3.2 | 2.9 | 1.9 | 2.8 | 2.9 |
| 9 | 1.5 | 1.3 | 3.2 | 1.9 | 1.5 | 1.3 | 2.6 | 2.2 | 4.9 | 4.5 | 2.1 | 1 | 2.7 | 3 | 2.8 | 1.8 | 4 | 3 |
| 10 | 1.7 | 0.7 | 2.4 | 2.1 | 1.2 | 1.2 | 1.4 | 1.7 | 4.3 | 4.4 | 1.3 | 0.9 | 2 | 2.3 | 2.9 | 2.6 | 3.3 | 3.6 |
| 11 | -0.3 | -0.9 | 1.1 | 1.8 | 1.8 | 2 | 3.1 | 2.5 | 4.7 | 7.3 | 0.8 | 0.4 | 1.5 | 2.2 | 4.3 | 4.2 | 4.2 | 3.9 |
| 12 | -0.2 | -0.5 | 1.3 | 0.6 | 1 | 1.1 | 3.7 | 2.6 | 3 | 2.6 | 1.8 | 1.1 | 4.8 | 3.7 | 3.2 | 3.6 | 2.7 | 3.6 |
| 13 | -1.9 | -1.7 | 2.9 | 1.4 | 1 | 0.6 | 0.6 | 0.8 | 6.7 | 4.9 | 0.3 | 0.8 | 3.4 | 5.3 | 4 | 3.6 | 2.2 | 2.9 |
| 14 | -0.5 | -0.6 | 1.7 | 1.7 | 0.6 | 0.6 | 1.8 | 2.1 | 3.7 | 4.8 | 0.8 | 0.8 | 3.4 | 4.6 | 3.6 | 3.3 | 4.4 | 4.7 |
| 15 | 1.4 | 0.7 | 2.1 | 2.5 | 1.1 | 1.6 | 2 | 2.7 | 4.1 | 3.9 | 1.5 | 1.5 | 3.8 | 4.1 | 3.2 | 2.5 | 3 | 3.6 |
| 16 | 2.9 | 1.6 | 1.3 | 0.9 | 1.4 | 1.6 | 2.2 | 2.8 | 3.2 | 2.4 | 2.1 | 1.8 | 5.4 | 2.9 | 4.4 | 3 | 3.7 | 2.8 |
| 17 | 0.5 | 0.4 | 3 | 3.1 | 0.6 | 0.9 | 1.4 | 1.2 | 3.8 | 3.5 | 0.7 | 0.2 | 4 | 3.2 | 3.9 | 3.1 | 2 | 2.3 |
| 18 | 3.1 | 1.4 | 0.5 | 0.9 | 1.1 | 1.3 | 5.6 | 3.4 | 3.5 | 3.1 | 1.7 | 2.7 | 3.8 | 3.5 | 4.7 | 2.9 | 3.6 | 3.7 |
| 19 | 0.5 | -0.2 | 1.4 | 2.9 | 1.5 | 1.4 | 3.1 | 1.9 | 6.5 | 5.3 | 2 | 1 | 2.6 | 2.4 | 2.5 | 2.7 | 5.1 | 5.4 |
| 20 | 0.9 | 0.7 | 0.3 | 1.2 | 1.6 | 1.7 | 4.1 | 4.1 | 5.5 | 5.6 | 1.3 | 1.2 | 5.1 | 3.7 | 2.4 | 1.9 | 5.1 | 4.9 |
| 21 | 0.1 | -0.1 | 1.6 | 2.2 | 1.4 | 1.3 | 3.5 | 3.4 | 3.7 | 3.3 | 2.7 | 1.9 | 2.7 | 3.9 | 3.8 | 3.5 | 3.1 | 4 |
| 22 | 0.4 | 0.5 | 1.4 | 3.3 | 1.7 | 1.8 | 0.8 | 0.8 | 3.3 | 3.9 | 0.7 | 0.7 | 2.6 | 2.3 | 3.4 | 3.3 | 3.8 | 4.3 |
| 23 | 1 | 0.1 | 1.4 | 2.4 | 2.4 | 2.1 | 2.2 | 2.1 | 4.4 | 3.4 | 2.2 | 1.8 | 3.2 | 2.7 | 2.7 | 2.2 | 4.7 | 4.4 |
| 24 | 2.3 | 3.8 | 1.5 | 2.6 | 2.4 | 4.4 | 3.4 | 4.6 | 3.8 | 4.8 | 2.2 | 3.5 | 6 | 2.4 | 3.7 | 4.4 | 4.7 | 3.9 |
| 25 | 2.2 | 1.6 | 1.8 | 1.3 | 3 | 2.4 | 4.6 | 4.4 | 1.1 | 1.3 | 4.6 | 4.4 | 5.3 | 3.8 | 3.6 | 2.7 | 2 | 1.6 |
| 26 | 0.9 | 0.5 | 2 | 2 | 1.7 | 0.9 | 2.5 | 3 | 4.6 | 2.9 | 2.5 | 2.2 | 4.2 | 3.1 | 2.5 | 2.2 | 2 | 2.6 |
| 27 | 2.8 | 1.6 | 0.9 | 0.7 | 1.5 | 1.8 | 3.6 | 2.6 | 5.1 | 5.7 | 3.4 | 2 | 2.9 | 1.7 | 1.8 | 1.9 | 5.7 | 5.8 |
| 28 | 1.8 | 1.5 | 2.2 | 2.7 | 1.7 | 1.9 | 3.1 | 4.4 | 3.6 | 4.8 | 2.3 | 2.6 | 2.9 | 3.3 | 2.9 | 2.9 | 4.1 | 4.5 |
| 29 | 0.9 | 0.7 | 1.1 | 0.9 | 0.2 | 0.3 | 1.9 | 2.1 | 6.8 | 6.1 | 0.8 | 0.4 | 2.7 | 4.1 | 2.4 | 2.2 | 5.5 | 6 |
| 30 | 1.2 | 0.7 | 2.4 | 1.4 | 1.5 | 1.4 | 2 | 1.8 | 3 | 3.4 | 1.7 | 1.6 | 3.2 | 3.8 | 2.9 | 2.8 | 2.5 | 3.6 |
| 31 | 1.1 | 0.3 | 2 | 2.3 | 1.6 | 1.4 | 1.9 | 1.2 | 6.2 | 6.1 | 0.9 | 0.7 | 2.8 | 2.5 | 3.1 | 2.9 | 5.4 | 5.8 |
| 32 | 0.5 | 0.3 | 3.3 | 3 | 1.4 | 1.4 | 2.2 | 2 | 4.8 | 5.5 | 2.2 | 2 | 4.1 | 3.5 | 2.4 | 2 | 5.7 | 4.7 |
| 33 | 4.2 | 2.1 | 1.7 | 2.6 | 0.9 | 0.6 | 4.6 | 3.8 | 7.6 | 5.8 | 1.6 | 1.9 | 3.7 | 4.5 | 3.5 | 2.3 | 6.6 | 5.5 |
| 34 | 0.7 | 0 | 3.7 | 2 | 0.7 | 0.6 | 2.7 | 3.2 | 4.7 | 4.4 | 1 | 1 | 2.7 | 4.1 | 3.1 | 2.1 | 5.2 | 5.3 |
| 35 | 0 | -0.9 | 2.9 | 3 | 1.7 | 1.1 | 2.5 | 1 | 4.2 | 3.8 | -0.2 | -0.1 | 4.7 | 1.8 | 1.9 | 1.6 | 3.2 | 3.1 |
| 36 | 0.5 | 1.2 | 2.9 | 5.7 | 2.4 | 2.3 | 3.1 | 1.9 | 3.4 | 2.4 | 1.3 | 1.1 | 4.1 | 4.7 | 3.4 | 2.7 | 3.5 | 5.8 |
| 37 | -0.2 | 0.8 | 3.9 | 3.2 | 0.3 | -0.2 | 2.1 | 1.7 | 6.3 | 6.2 | 0.5 | 0.5 | 2.9 | 2.5 | 1.8 | 1.8 | 5.5 | 6 |
| 38 | 1.7 | -0.3 | 2.7 | 2.6 | 0.9 | 0.7 | 4.1 | 3.1 | 5 | 5.6 | 1.8 | 0.5 | 2.6 | 2.7 | 3.4 | 3.7 | 3.4 | 4.6 |
| 39 | 1.6 | 0.8 | 2.2 | 2.3 | 1.9 | 1.5 | 1.9 | 2.2 | 5.1 | 3.4 | 1.6 | 1.7 | 4.9 | 2.6 | 4.9 | 3.2 | 4.9 | 5.3 |
| 40 | 0.9 | -0.1 | 1.6 | 3.5 | 1.7 | 2.5 | 1.9 | 1.7 | 2.3 | 2.3 | 1.2 | 1.1 | 2.7 | 3.2 | 4 | 3.6 | 4.1 | 3.7 |
| 41 | -0.5 | 0 | 1.3 | 3.2 | 2.2 | 1.9 | 1.9 | 0.9 | 4 | 4.1 | -0.2 | 0 | 4.3 | 3.6 | 3.2 | 2.9 | 3.2 | 2.5 |
| 42 | -0.2 | -0.2 | 2.8 | 2.2 | 1.7 | 1.5 | 1.2 | 1 | 4.1 | 3.2 | 1.2 | 1 | 3 | 2 | 4.3 | 4.6 | 6.6 | 5.7 |
| 43 | 2.8 | 1.5 | 1.3 | 1.6 | 2.7 | 3 | 3 | 2.2 | 4.8 | 4.9 | 3 | 2.1 | 2.3 | 2.5 | 3.8 | 4.2 | 6.4 | 6.3 |
| 44 | 1.9 | 1.8 | 2.1 | 2.5 | 2 | 1.6 | 3.5 | 3.7 | 2.5 | 4.4 | 1.2 | 0.6 | 2.6 | 4 | 3.2 | 3.8 | 2.2 | 3.4 |
| 45 | 5.1 | 3.6 | 1.9 | 1.8 | 1.9 | 1.9 | 4.6 | 4.6 | 2.5 | 2.5 | 3.5 | 2.5 | 3.7 | 4 | 4.9 | 3.3 | 2.3 | 2.4 |
| 46 | 1.8 | 0.9 | 0.5 | 2.6 | 0.5 | 1.6 | 2.8 | 2.5 | 4.3 | 3.2 | 1.7 | 1.8 | 4.3 | 3.5 | 4.5 | 3.8 | 2.5 | 4.7 |
| 47 | -0.3 | -0.2 | 2.2 | 3.8 | 1.2 | 1.3 | 0.5 | 1 | 3.3 | 3.7 | 0.4 | 0.1 | 1.8 | 1.9 | 3.5 | 3.1 | 3.5 | 3.9 |
| 48 | -0.2 | -0.4 | 0 | 2 | 0.2 | 1.6 | 1.5 | 2.1 | 3.8 | 4.1 | 0.6 | 1.1 | 2.9 | 4.1 | 3.1 | 3 | 2.8 | 2.8 |
| 49 | 2.9 | 1.4 | 1.2 | 2.3 | 1.6 | 1.8 | 3.4 | 2 | 4.4 | 4.1 | 3.4 | 2 | 2.8 | 2.3 | 4.2 | 3.9 | 3.2 | 3.6 |
| 50 | -0.7 | -0.1 | 2.7 | 2 | 2.3 | 2.7 | 1.8 | 2.4 | 7 | 5.5 | 1.6 | 1.4 | 3.2 | 1.8 | 2.6 | 2.7 | 4.5 | 4.7 |
| 51 | 1.7 | 0.9 | 2 | 2.8 | 0.5 | 0.4 | 6.2 | 5.5 | 2.6 | 3.3 | 1.8 | 1.6 | 4.4 | 3.8 | 3.3 | 3.4 | 2.6 | 2.8 |
| 52 | 0.4 | 0.2 | 2.4 | 2.7 | 1 | 1.7 | 3 | 3.1 | 5.7 | 5.6 | 1.2 | 1.6 | 3.3 | 2.9 | 4.4 | 4.2 | 3.3 | 4.5 |
| 53 | -1.4 | -1.5 | 1.8 | 2.9 | 1.5 | 1.1 | 0.3 | 0.1 | 4.1 | 3.6 | 2.3 | 2.7 | 4.5 | 4.8 | 4.3 | 4.2 | 3.4 | 3.4 |
| 54 | 1.7 | 0.2 | 2.5 | 2.6 | 1 | 1 | 3.9 | 2.7 | 3.6 | 5.3 | 1.7 | 1.3 | 4 | 2 | 3.8 | 3.2 | 5.7 | 5.5 |
| 55 | 2.3 | 1.4 | 2.4 | 1.8 | 1.6 | 1.3 | 2.9 | 2.5 | 2.7 | 2.9 | 2.4 | 1.5 | 4.2 | 2.9 | 3.3 | 3 | 3.2 | 3.2 |
| 56 | 2.5 | 2.7 | 2.7 | 2.3 | 1.7 | 3.5 | 3.9 | 4.7 | 3.5 | 3.4 | 1.9 | 1.8 | 4 | 3 | 4.1 | 3.5 | 3.9 | 3.6 |
| 57 | 3.6 | 2.3 | 2.7 | 3.6 | 2.2 | 2.3 | 2.8 | 2.8 | 3.1 | 4.6 | 2.5 | 1.8 | 3.5 | 2.6 | 3.2 | 2.3 | 3.6 | 4.1 |
| 58 | 2.1 | 2.2 | 0.8 | 1.3 | 2.6 | 3.2 | 2.6 | 2.5 | 2.6 | 3 | 2.2 | 2.1 | 2.5 | 3.5 | 3.9 | 4.8 | 1.7 | 2.4 |
| 59 | -0.2 | -0.5 | 2.8 | 3.6 | 1.2 | 0.9 | 2.5 | 3.4 | 5 | 5.5 | 1.2 | 1.2 | 2.6 | 3.2 | 3.3 | 3 | 3.5 | 4 |
| 60 | 0.8 | 0.1 | 0.9 | 2 | 1.1 | 1.9 | 2.6 | 2.8 | 2.5 | 4 | 1.2 | 0.7 | 2.7 | 2.6 | 3.4 | 3.8 | 3.2 | 5.7 |
| 61 | 2 | 2.4 | 2.3 | 2 | 1.7 | 2.2 | 4.5 | 4.3 | 2.7 | 1.9 | 3.4 | 2.6 | 2.5 | 3.4 | 3.9 | 3.9 | 2 | 2 |
| 62 | 3.5 | 1.6 | 0.7 | 1.6 | 1.1 | 1.3 | 3.9 | 4 | 3.9 | 4.5 | 2.2 | 2.2 | 3.7 | 4.8 | 4.4 | 4.5 | 5.8 | 5.6 |
| 63 | -0.4 | 0.7 | 1.3 | 2.8 | 1.6 | 1.5 | 0.9 | 0.8 | 3.4 | 3.6 | 0.9 | 0.8 | 2.7 | 2.9 | 2.7 | 3.1 | 3.1 | 3.8 |
| 64 | 3.2 | 2.9 | 2.3 | 1.9 | 1.2 | 1.1 | 3.7 | 3.4 | 4.9 | 4.9 | 3 | 1.9 | 2.9 | 2.9 | 3.6 | 3.2 | 5.4 | 5.1 |
| 65 | 1 | 0.3 | 1.6 | 1.6 | 1.4 | 0.7 | 2 | 2.4 | 4.8 | 4.6 | 2 | 1.1 | 3.8 | 3.5 | 2.9 | 2.2 | 4.5 | 5.1 |
| 66 | -0.7 | -0.7 | 3.1 | 2.5 | 1.3 | 1.1 | 0.2 | 0.6 | 5.1 | 4.4 | 0.2 | 0.6 | 2.6 | 3.5 | 3.4 | 3.5 | 3 | 4.7 |
| 67 | -0.7 | -0.9 | 0.1 | 1.1 | 1.8 | 1.6 | 0.2 | 0.1 | 8.8 | 7 | 0.2 | 0.1 | 4.2 | 4.7 | 2.9 | 2.7 | 3.8 | 3.3 |
| 68 | 1.7 | 1.1 | 0.8 | 1.5 | 2 | 1.9 | 3.5 | 3.3 | 2.4 | 4.3 | 2.4 | 2.5 | 3.8 | 4.7 | 3.1 | 4 | 2 | 2.9 |
| 69 | 0 | 0.2 | 2.7 | 2 | 1.2 | 2 | 3.4 | 3 | 3.5 | 3.7 | 0.7 | 1.1 | 2.6 | 4 | 2.1 | 2 | 4.3 | 4.5 |
| 70 | 2 | 1.4 | 1.1 | 2.6 | 2.6 | 2.4 | 3.1 | 2.5 | 2.3 | 4.2 | 1.9 | 1.6 | 4.1 | 3.9 | 3.4 | 2.9 | 2.9 | 3.7 |
| 71 | 0.9 | -0.6 | 3.8 | 5.1 | 2 | 0.9 | 3 | 2.8 | 4 | 4 | 1.7 | 1.8 | 3 | 2.1 | 2.8 | 3.4 | 5.1 | 4.9 |
| 72 | -0.5 | -1.1 | 0.8 | 1.6 | 0.1 | 0.4 | 0.9 | 0.6 | 6.5 | 4.9 | 0.5 | -0.6 | 5.8 | 3.3 | 1.6 | 1.5 | 2.5 | 3.7 |
| 73 | 1.4 | 1.7 | 2.5 | 3.4 | 2.4 | 2.4 | 3.2 | 2.9 | 3.8 | 3.3 | 2.8 | 2.6 | 2.9 | 2.4 | 2.4 | 2.4 | 3.3 | 4.8 |
| 74 | 0.5 | -0.5 | 3.6 | 4.4 | 2.4 | 2.3 | 1.3 | 0.7 | 3.4 | 4.9 | 1.1 | 0.7 | 3.1 | 3 | 2.4 | 2.3 | 3.1 | 4.7 |
| 75 | 2.5 | 2.4 | 2.4 | 2.4 | 1.7 | 2.6 | 2.7 | 2 | 4.2 | 4.2 | 2.5 | 1.3 | 3.4 | 3.3 | 1.9 | 3.3 | 3.5 | 3.7 |
| 76 | 2 | 1.6 | 2.5 | 2.2 | 3.2 | 3.1 | 2.2 | 2.1 | 3 | 3.6 | 2.2 | 2.1 | 2.6 | 2.6 | 4.3 | 3.1 | 3 | 3.3 |
| 77 | -0.1 | 0 | 2.6 | 2.9 | 2.4 | 3.3 | 2 | 1.9 | 3.6 | 4.4 | 1.5 | 1 | 3.2 | 2.6 | 4.6 | 4.8 | 5.5 | 5.3 |
| 78 | 2.1 | 1.7 | 1.2 | 2.3 | 1.6 | 1.1 | 3.8 | 4.4 | 4.3 | 4.7 | 2.5 | 2.2 | 3.8 | 2.7 | 3 | 3.3 | 4.5 | 7.2 |
| 79 | 1.7 | 1.1 | 1.9 | 2.9 | 1.9 | 3 | 1.7 | 2.3 | 3 | 4 | 1.7 | 1.6 | 2.4 | 3 | 3.5 | 3.6 | 3.8 | 2.7 |
| 80 | 1 | 0.6 | 0.5 | 2.5 | 1.5 | 1.7 | 1.7 | 2.2 | 3.7 | 3 | 1 | 0.9 | 2.7 | 2.5 | 3.4 | 3.7 | 3.3 | 3.1 |

| Region of Interest | Frontal lobe | Frontal lobe | Fusiform gyrus | Fusiform gyrus | Gyrus rectus | Gyrus rectus | Heschl gyrus | Heschl gyrus | Hippocampus | Hippocampus | Inferior frontal gyrus, opercular part | Inferior frontal gyrus, opercular part | Inferior frontal gyrus, orbital part | Inferior frontal gyrus, orbital part | Inferior frontal gyrus, triangular part | Inferior frontal gyrus, triangular part | Inferior occipital gyrus | Inferior occipital gyrus |
| --- | --- | --- | --- | --- | --- | --- | --- | --- | --- | --- | --- | --- | --- | --- | --- | --- | --- | --- |
| Side | L | R | L | R | L | R | L | R | L | R | L | R | L | R | L | R | L | R |
| Number 1 | 3 | 3.6 | 2.8 | 3 | 1.4 | 1.2 | 1 | 0.6 | 4.5 | 3.7 | 1.4 | 0.7 | 2.3 | 1.7 | 1.6 | 0.4 | 2.6 | 4 |
| 2 | 2.5 | 4 | 3.5 | 4.5 | 0.6 | 0.9 | 1.1 | 1.1 | 4.7 | 4.7 | 1.5 | 0.9 | 1.8 | 2.6 | 1 | 0.9 | 3.1 | 4.3 |
| 3 | 4.5 | 3 | 5.5 | 4 | 0.5 | 0.1 | 1.4 | 1.1 | 2.3 | 1.2 | 1.3 | -0.3 | 1.5 | 0.8 | 1.5 | -0.2 | 6.1 | 4.4 |
| 4 | 2.3 | 3.2 | 3 | 3.6 | 1.6 | 1.8 | 3.1 | 4.2 | 2.9 | 2 | 0.8 | 1.3 | 1.1 | 0.3 | 1.2 | 1.3 | 3.7 | 3.8 |
| 5 | 4.2 | 3.1 | 3.2 | 3.6 | 0.5 | 0.2 | 0.9 | 0.1 | 1.8 | 0.3 | 1.4 | 1 | 2.9 | 1.3 | 3.1 | 0.9 | 3.6 | 4.5 |
| 6 | 3.9 | 4.4 | 2.1 | 3.6 | 1.8 | 1.5 | 0.8 | 1.3 | 2.5 | 2.9 | 2.1 | 2.1 | 0.8 | 1.7 | 1 | 1.5 | 1 | 1.7 |
| 7 | 3.7 | 2.9 | 4.4 | 7.2 | 0.4 | 1 | 1.6 | 0.7 | 1.8 | 2 | 1.2 | 0.8 | 1.2 | 1.2 | 3.6 | 1.2 | 7.6 | 10.4 |
| 8 | 4.3 | 3.8 | 2.5 | 3.1 | 2.2 | 1.4 | 1.3 | 2.5 | 1.2 | 1.3 | 2.3 | 2.7 | 3.5 | 3.6 | 2.2 | 2.1 | 1.5 | 3.4 |
| 9 | 4.6 | 3.1 | 3.3 | 2.2 | 2.7 | 0.9 | 3.1 | 2.5 | 1.9 | 1.8 | 1.6 | 2.3 | 4.5 | 2.8 | 1.5 | 3 | 2.5 | 0.8 |
| 10 | 2 | 2.9 | 3.4 | 3.7 | 0.8 | 0.4 | 2.1 | 2 | 2 | 1.9 | 0.8 | 1.6 | 1.5 | 1.8 | 1.1 | 1.3 | 4.3 | 4.1 |
| 11 | 3.3 | 3.6 | 2.8 | 7.4 | 1 | 1.3 | 0.6 | 0.6 | 2.3 | 1.1 | 0.4 | 2.2 | 3.1 | 2.6 | 0.6 | 2.2 | 4.5 | 10.7 |
| 12 | 3.1 | 3.4 | 2.5 | 1.4 | -0.1 | -0.1 | 2.4 | 1.4 | 2.8 | 1.6 | 1.2 | 2 | 2.2 | 2.8 | 2.1 | 1.3 | 1.8 | 2.6 |
| 13 | 3.4 | 5 | 5 | 6.6 | 0.5 | 0.3 | 1 | 2.5 | 0.8 | 0.7 | 1.6 | 3.4 | 0.1 | 0.1 | 3.3 | 3.1 | 5 | 7.6 |
| 14 | 3.2 | 3.5 | 2.1 | 2.2 | -0.8 | -0.8 | 2.7 | 2.4 | 2.1 | 0.5 | 0.6 | 2.3 | 0.7 | 2 | 1 | 1.4 | 4 | 3.1 |
| 15 | 3.3 | 2.7 | 5 | 4.3 | 0.7 | -0.5 | -0.1 | 0.4 | 3 | 1.1 | 0.3 | 0.5 | 1.6 | 1 | 0.8 | 0.6 | 3.6 | 4.3 |
| 16 | 4.8 | 4.4 | 3.4 | 2.3 | 1.8 | 1.8 | 1.6 | 1.7 | 4.2 | 2 | 1.8 | 1.7 | 2.3 | 3.5 | 1.7 | 1.9 | 0.8 | 1.4 |
| 17 | 3.4 | 3.2 | 3.2 | 2.4 | 0.8 | 0.7 | 2.3 | 2.4 | 1.8 | 1 | 2.1 | 1.4 | 0.9 | 0.9 | 1.6 | 1 | 2.8 | 2.9 |
| 18 | 4.6 | 3.5 | 1.8 | 2.7 | 2.2 | 2.8 | 2.1 | 2.4 | 3.1 | 1.8 | 1.6 | 1.7 | 2.3 | 1.9 | 0.8 | 1.4 | 2.4 | 2.9 |
| 19 | 2.9 | 1.7 | 4.6 | 5 | 1.2 | 1 | 2 | 2.4 | 1.9 | 2.1 | 2.6 | 1.5 | 2.9 | 1.5 | 2.9 | 1.3 | 5.5 | 5.6 |
| 20 | 3 | 3.3 | 4.6 | 5.9 | 1.1 | 0.7 | 2.3 | 3.1 | 2.1 | 2.4 | 0.6 | 0.6 | 1.4 | 1.4 | 0.7 | 0 | 5.4 | 6.1 |
| 21 | 3.3 | 3.8 | 1.8 | 4.6 | 1.1 | 0.5 | 1.8 | 1.5 | 1.3 | 1.1 | 0.8 | 0.6 | 0.4 | -0.1 | 1 | 0.4 | 3.2 | 4.3 |
| 22 | 3.3 | 3.1 | 2.9 | 3 | 0.8 | 0.4 | 1.4 | 1.2 | 1.3 | 2.1 | 2 | 0.5 | 1.4 | 0.5 | 2.3 | 0.6 | 4.2 | 4 |
| 23 | 2.9 | 2.7 | 3.4 | 4 | 0.1 | 0.6 | 1.2 | 1.1 | 4.4 | 2.8 | 1.1 | 1.2 | 1.6 | 1.9 | 0.5 | 1.3 | 2.1 | 4.5 |
| 24 | 5.8 | 4.6 | 4.6 | 4.1 | 2.4 | 3.3 | 1.6 | 2.1 | 4.4 | 4.3 | 1.9 | 2.9 | 1.9 | 2.4 | 3.5 | 2.5 | 2.2 | 2.6 |
| 25 | 4 | 3.7 | 3.3 | 2.1 | 4 | 3.7 | 3.1 | 1.9 | 4.2 | 2.5 | 3.8 | 0.6 | 2 | 2.6 | 2 | 0.6 | -0.6 | -0.6 |
| 26 | 3.3 | 5 | 4.7 | 3.4 | 1.3 | 1 | 5.7 | 3.1 | 3.3 | 2.1 | 2.6 | 2.5 | 2.4 | 2.5 | 2.6 | 2.9 | 8.2 | 4.7 |
| 27 | 3.1 | 3.5 | 5.5 | 6.5 | 1.2 | 0.3 | 1.5 | 0.9 | 3.9 | 2.2 | 2 | 0.9 | 2.6 | 2.9 | 2.2 | 1.7 | 5.7 | 7.3 |
| 28 | 2.5 | 3 | 2.7 | 4.4 | 0.3 | 0.6 | 1.2 | 1.2 | 3.5 | 3.4 | 1 | 1.5 | 2.3 | 1.8 | 1.3 | 2.2 | 4.9 | 4.3 |
| 29 | 3 | 2.6 | 3.8 | 6.9 | 0.6 | 0.6 | 1 | 1.9 | 2.4 | 1.2 | 2.9 | 1.6 | 1.4 | 1.6 | 1.5 | 1.8 | 3.9 | 7.3 |
| 30 | 3 | 2.9 | 2 | 1.8 | 0.9 | 0.8 | 2.5 | 1.3 | 1.1 | 0.6 | 1.7 | 1.2 | 2.7 | 2.9 | 2.5 | 1.8 | 2 | 1.2 |
| 31 | 3.3 | 2.6 | 2.8 | 4.3 | 0.7 | -0.6 | 0.6 | 0.2 | 2.2 | 1.7 | 0.7 | 0.6 | 1.5 | 1.7 | -0.2 | 0 | 4.6 | 5.5 |
| 32 | 4.3 | 4 | 3.1 | 3.5 | 0.2 | 0.5 | 1.1 | 1.5 | 2.3 | 1.5 | 4.3 | 2.7 | 2.9 | 2.1 | 3.9 | 1.7 | 5.3 | 4.9 |
| 33 | 3.9 | 3 | 5.2 | 5.6 | 1 | 1 | 1.6 | 2.1 | 4.1 | 2.7 | 1.8 | 2.4 | 2.8 | 2.6 | 2.7 | 2.6 | 5.6 | 6.6 |
| 34 | 2.4 | 2.9 | 4.3 | 2.4 | -0.4 | -0.6 | 2.4 | 3.7 | 3.2 | 1 | 1 | 0.4 | 2.3 | 1.3 | 1.4 | 0.2 | 4.5 | 2.9 |
| 35 | 5.2 | 2.5 | 3.1 | 4.9 | 0.2 | 0.1 | 0.9 | 0.1 | 2.1 | 0.5 | 1.7 | 1.1 | 0.8 | 0 | 1.4 | 0 | 5.9 | 6.2 |
| 36 | 3.8 | 4.5 | 2.9 | 4.3 | 2 | 1.2 | 2.2 | 2.3 | 3.3 | 2 | 2.1 | 2.4 | 1.6 | 1.9 | 2.4 | 1.6 | 2.9 | 4.3 |
| 37 | 2.7 | 2.1 | 3.9 | 5.2 | -0.2 | 0 | 2 | 1.8 | 2.1 | 1.2 | 0.9 | 0.1 | 0.4 | 0.6 | 1.1 | 0.5 | 6.9 | 5.9 |
| 38 | 3.2 | 3.1 | 3 | 5.6 | 0.3 | 0 | 0.7 | 1.3 | 3.4 | 2.4 | 1.4 | 1.7 | 0.8 | 0.9 | 1.7 | 0.4 | 7.6 | 5.9 |
| 39 | 5.1 | 4.6 | 3.2 | 2.8 | 1.1 | 0.1 | 1.1 | 1 | 2.9 | 1.9 | 1.5 | 1 | 2.4 | 1.2 | 1 | 1.4 | 2.7 | 3.2 |
| 40 | 3.6 | 3 | 1.3 | 2.3 | 1.4 | 1.9 | 0.4 | 0.2 | 1.7 | 0.8 | 1.1 | 1.7 | 0.7 | 1 | 1.4 | 1.8 | 0.9 | 1.2 |
| 41 | 4.2 | 3 | 1.8 | 3.6 | 0.8 | 0.7 | 3.1 | 3.7 | 0.6 | 0.2 | 1.9 | 1.6 | 1.1 | 0.8 | 2.1 | 1.6 | 1.1 | 3.5 |
| 42 | 4.1 | 4.1 | 2.7 | 2 | 1.3 | 0.3 | 0.6 | 0.8 | 1.9 | 1.8 | 1.8 | -0.2 | 2.9 | 3.9 | 1.7 | 0.7 | 3 | 1.9 |
| 43 | 3.4 | 2.7 | 2.5 | 3.4 | 2.6 | 1.4 | 1.1 | 1.3 | 3.1 | 4.3 | 1.1 | 0.9 | 3.1 | 2.6 | 1 | 0.7 | 3.4 | 3.7 |
| 44 | 3.1 | 4.1 | 2.8 | 6.2 | 0 | 0 | 1.9 | 1.7 | 2 | 3 | 0.5 | 2.5 | 1.3 | 0.9 | 1 | 2.4 | 3.4 | 6.8 |
| 45 | 4.7 | 3.3 | 6 | 3.2 | 2.8 | 2.4 | 3.4 | 2.8 | 5.8 | 3.8 | 2.4 | 1.5 | 2.3 | 2.1 | 1.9 | 1.4 | 1.3 | 2 |
| 46 | 4.4 | 6.5 | 3.3 | 3.7 | 0.3 | -0.4 | -0.8 | 1 | 1.7 | 1 | 0.8 | 2.5 | 1.5 | 1.2 | 1 | 2.2 | 6.5 | 4.5 |
| 47 | 3.2 | 2.2 | 2.9 | 2.8 | 0.5 | 0.2 | 0.4 | 0.8 | 1.8 | 0.9 | 1.7 | 1.7 | 2.1 | 1.7 | 2.1 | 1.9 | 3.6 | 4.1 |
| 48 | 3.8 | 4.2 | 1.4 | 3.5 | -0.1 | -0.1 | 1.8 | 3.5 | 0.7 | 2.6 | 1.1 | 2.3 | 1.3 | 2.1 | 1 | 1.9 | 2.4 | 4.7 |
| 49 | 4.2 | 4.2 | 5.9 | 2.9 | 1.1 | 1 | 1.8 | 2 | 3.8 | 1.9 | 1.2 | 1.3 | 1 | 1.6 | 0.4 | 0.4 | 2.9 | 3.4 |
| 50 | 3.6 | 4.3 | 4 | 3.9 | -0.5 | 0.2 | 0.2 | 0.7 | 1.2 | 0.3 | 1.3 | 0.7 | 0.1 | 1.3 | 1.2 | 0.2 | 4.2 | 4.6 |
| 51 | 4.2 | 5.2 | 2.8 | 2.5 | 0.3 | 1.1 | 1.2 | 1.7 | 1.5 | 1 | 1.1 | 0.8 | 2.5 | 1.9 | 0.6 | 1.4 | 2.2 | 3.1 |
| 52 | 3.7 | 3.9 | 4.7 | 6.2 | 0.4 | -0.1 | 1.4 | 0.9 | 2.4 | 0.9 | 2.2 | 1.4 | 2.5 | 1.5 | 2.4 | 1.1 | 7.6 | 7.4 |
| 53 | 3.2 | 3.2 | 2.5 | -0.3 | -0.4 | 1 | 0.1 | 0.3 | -0.6 | 2.1 | 2.6 | 1 | 2.3 | 1.9 | 2.7 | 4.1 | 3.5 | 3.4 |
| 54 | 3.4 | 4.8 | 3.3 | 6 | 0.8 | 0.7 | 2.6 | 1.7 | 2.5 | 1.9 | 1.9 | 1.2 | 2.4 | 2.9 | 2.1 | 2.5 | 5.7 | 7 |
| 55 | 3.9 | 3 | 3.6 | 4.8 | 0.9 | 1 | 2.3 | 1.1 | 3.6 | 2.2 | 1.6 | 0.6 | 1 | 0.7 | 1.6 | 0.5 | 3.7 | 5.6 |
| 56 | 3.7 | 3 | 3.6 | 3.3 | 1.8 | 2.3 | 1.6 | 1.4 | 3.3 | 2.5 | 1.4 | 2.2 | 2.1 | 3 | 1.3 | 2.9 | 3.7 | 2.3 |
| 57 | 2.7 | 2.1 | 3.6 | 4.8 | 1.3 | 1 | 3.4 | 3.4 | 3.8 | 2.3 | 1.6 | 1.2 | 1.5 | 1.8 | 2.7 | 1 | 3.8 | 7 |
| 58 | 3.1 | 4 | 2.8 | 4.2 | 1.2 | 1.3 | 1.6 | 1.5 | 3.9 | 4.1 | 2.8 | 1.7 | 3 | 3.1 | 2.6 | 1.5 | 2.1 | 4.7 |
| 59 | 3.2 | 3 | 2.8 | 5.6 | 0.3 | -0.1 | 1.3 | 0.6 | 1.1 | 0.6 | 1 | 1.2 | 1.4 | 1.2 | 2.6 | 1.5 | 4.6 | 5.8 |
| 60 | 3.1 | 3.1 | 1.3 | 4.8 | 0.9 | 0.5 | 2.6 | 2.7 | 2.6 | 2.7 | 1.2 | 1.3 | 2.8 | 0.7 | 0.9 | 1.5 | 4.6 | 5.2 |
| 61 | 3.1 | 4.5 | 4.1 | 3.2 | 1.5 | 1.4 | 1.8 | 3.3 | 3.9 | 2.6 | 1.1 | 1.8 | 1.4 | 1.3 | 1.9 | 1.7 | 1.3 | 1.9 |
| 62 | 4.2 | 4.1 | 2.2 | 2.1 | 1.5 | 0.7 | 2.6 | 2.4 | 3.8 | 4 | 0.6 | 1.6 | 1.7 | 1.7 | 1.6 | 1.1 | 2.2 | 2.2 |
| 63 | 3.5 | 3.8 | 3.2 | 3.5 | 0.3 | 0.5 | 1.2 | 1.2 | 2.6 | 2.7 | 0.8 | 2.3 | 1.3 | 0.3 | 1.3 | 0.9 | 6.1 | 4 |
| 64 | 3.4 | 3 | 2.4 | 5.3 | 2.6 | 1.6 | 1.1 | 1.2 | 3 | 2.4 | 1 | 1.2 | 2.2 | 3 | 1.5 | 1.4 | 3.1 | 5.5 |
| 65 | 3.7 | 3 | 4.8 | 3.3 | 1.7 | 0.8 | 1.2 | 0.8 | 2.6 | 1.8 | 2.4 | 2.9 | 2.3 | 2.4 | 2.2 | 1.9 | 4.8 | 3.1 |
| 66 | 4.5 | 3.3 | 3.5 | 4.5 | -0.4 | 0.1 | 0.8 | 0.4 | 3.5 | 1.7 | 1.4 | 1.7 | 2.5 | 1.7 | 2 | 0.5 | 4.1 | 5.4 |
| 67 | 3.6 | 4.4 | 5.3 | 7.8 | 0.7 | 0.7 | 1 | 1.8 | 1.8 | 1.1 | 2.2 | 1.5 | 1.5 | 1.2 | 3.1 | 0.7 | 6.6 | 9.3 |
| 68 | 4.3 | 5.7 | 1.9 | 6.6 | -0.1 | -0.4 | 2.5 | 1.7 | 2.7 | 3.3 | 1.6 | 4.3 | 1.7 | 2.7 | 1.1 | 4.3 | 3.4 | 7.2 |
| 69 | 3.2 | 2.3 | 3 | 3 | 0 | 0.6 | 2.5 | 1.7 | 0.9 | 0 | 2.2 | 0.6 | 1.5 | 1 | 2.2 | 0.5 | 5.1 | 4.2 |
| 70 | 2.9 | 3.2 | 2.7 | 4.2 | 1 | 0.9 | 1.5 | 1.1 | 3.1 | 2.8 | 2.1 | 1.4 | 1.8 | 2.9 | 2 | 0.8 | 1.4 | 3.6 |
| 71 | 3.5 | 3.9 | 2.2 | 3.4 | 1.7 | 1.2 | 1.1 | 1.8 | 3.2 | 2.1 | 3 | 3.9 | 2.9 | 1.5 | 3.1 | 3.2 | 3.5 | 3.2 |
| 72 | 5.5 | 3.6 | 6.3 | 7.7 | 1.4 | 1.2 | -0.1 | 0.4 | 0.7 | 0.2 | 1.7 | 1.3 | 1.9 | 0.6 | 1.9 | 1.6 | 8.9 | 11 |
| 73 | 2.4 | 2.6 | 3.4 | 3.4 | 2.3 | 1.8 | 1.5 | 1.9 | 3.1 | 2.5 | 1.5 | 0.2 | 1.2 | 0.8 | 1.4 | 0.4 | 4.9 | 4.3 |
| 74 | 3.2 | 2.6 | 2.7 | 3.4 | 0.9 | 1 | 2.7 | 1.9 | 3.1 | 2.6 | 1.8 | 0.7 | 2.6 | 1.1 | 1.7 | 0.8 | 3.5 | 3.5 |
| 75 | 4.4 | 4.2 | 3 | 5.3 | 0.7 | 1.3 | 1.7 | 2.1 | 3.3 | 3.3 | 1.8 | 2.1 | 3 | 2 | 2.3 | 1.9 | 2.9 | 5.1 |
| 76 | 2.6 | 3.4 | 3.3 | 2.7 | 1.7 | 0.8 | 1.4 | 2.5 | 4.1 | 2.8 | 2 | 1.7 | 1.7 | 2.1 | 1.9 | 2.8 | 2.1 | 3 |
| 77 | 3.1 | 3.9 | 1.9 | 3 | 1.1 | 1 | 0.8 | 0.7 | 3.2 | 3.8 | 2.1 | 2.4 | -0.1 | 0.7 | 2 | 2 | 0.4 | 3.1 |
| 78 | 4.7 | 4 | 1.9 | 2.7 | 1 | 1 | 1.8 | 1.7 | 1.5 | 1.7 | 1.5 | 2.7 | 1.3 | 1.3 | 2.2 | 2.8 | 2.1 | 3.1 |
| 79 | 2.9 | 3.2 | 3.5 | 4.8 | 1.8 | 1.6 | 1.4 | 1.5 | 2.4 | 2.8 | 0.9 | 0.1 | 2 | 2.8 | 0.8 | 0.5 | 2.6 | 5.7 |
| 80 | 3.8 | 3.8 | 3.3 | 3 | 0.1 | -0.1 | 1 | 0.7 | 1.6 | 1.3 | 2 | 1.9 | 2.7 | 1.7 | 1.9 | 1 | 3.6 | 4.9 |

| Region of Interest | Inferior parietal, but supramarginal and angular gyri | Inferior parietal, but supramarginal and angular gyri | Inferior temporal gyrus | Inferior temporal gyrus | Insula | Insula | Lenticular nucleus, pallidum | Lenticular nucleus, pallidum | Lenticular nucleus, putamen | Lenticular nucleus, putamen | Lingual gyrus | Lingual gyrus | Mesial temporal lobe | Mesial temporal lobe | Middle cingulate and paracingulate gyri | Middle cingulate and paracingulate gyri | Middle frontal gyrus | Middle frontal gyrus |
| --- | --- | --- | --- | --- | --- | --- | --- | --- | --- | --- | --- | --- | --- | --- | --- | --- | --- | --- |
| Side | L | R | L | R | L | R | L | R | L | R | L | R | L | R | L | R | L | R |
| Number 1 | 1.8 | 2.1 | 2 | 2.2 | 2.1 | 2.2 | 1.6 | 1.2 | 2.7 | 2.1 | 3.2 | 4.2 | 4.5 | 3.7 | 3.8 | 4.3 | 2.2 | 1.5 |
| 2 | 1.4 | 0.8 | 1.5 | 2.9 | 2 | 1.5 | 2.7 | 1.9 | 3 | 1.8 | 4.1 | 4.4 | 4.7 | 4.7 | 3.1 | 3.2 | 2.4 | 2.7 |
| 3 | 4.5 | 3.1 | 2.1 | 3 | 2.5 | 2.1 | 2.4 | 0.7 | 2.3 | 0.7 | 5.8 | 4.4 | 2.3 | 2.4 | 4.6 | 2.9 | 2.1 | 1.6 |
| 4 | 2 | 2.9 | 1.5 | 3.4 | 2.7 | 4 | 2.2 | 1.7 | 3.1 | 4 | 5.8 | 4.8 | 3.2 | 2.2 | 2.5 | 3.5 | 2.3 | 2.8 |
| 5 | 2.5 | 2.2 | 2.9 | 2.5 | 1.7 | 0.8 | 0.9 | 0.1 | 1 | 0.5 | 4.5 | 4 | 2.5 | 1.4 | 4.1 | 3.4 | 3.8 | 3.1 |
| 6 | 2.4 | 2.7 | 1.9 | 1.5 | 2.6 | 2.2 | 2.8 | 2.6 | 2.4 | 2.1 | 1.7 | 1.8 | 2.5 | 3.7 | 4.6 | 4.9 | 3.9 | 2.8 |
| 7 | 3.3 | 1.1 | 2.3 | 6.2 | 1 | 1.2 | 0.6 | 0.6 | 0.9 | 0.8 | 4.2 | 5.9 | 3.5 | 3.1 | 2.6 | 2 | 3.7 | 1.1 |
| 8 | 1.2 | 1.2 | 1.2 | 2.1 | 2.3 | 3.1 | 1.9 | 1 | 2.4 | 2.8 | 4.8 | 3.9 | 2.5 | 3.1 | 2 | 1.9 | 4.3 | 3.7 |
| 9 | 0.6 | 1.4 | 1.2 | 1.4 | 2.5 | 3 | 2.3 | 1.8 | 2.6 | 2.2 | 4.8 | 3.9 | 2.2 | 2 | 2.8 | 1.8 | 2.8 | 2.9 |
| 10 | 1.8 | 2.2 | 2.2 | 2.3 | 2.2 | 2.6 | 1.4 | 0.6 | 1.2 | 1.7 | 4.5 | 3.7 | 2.9 | 1.9 | 2.9 | 2.6 | 1.9 | 1.5 |
| 11 | 1.4 | 1.7 | 1.5 | 2.5 | -0.1 | 0.8 | 2.8 | 1.9 | 3.1 | 2.5 | 4.7 | 6.4 | 3.2 | 1.1 | 4.3 | 4.2 | 2.2 | 2.9 |
| 12 | 2.9 | 2.7 | 1.5 | 1.5 | 3.2 | 2.1 | 3 | 1.5 | 3.7 | 2.6 | 2.6 | 1.9 | 2.8 | 1.6 | 3.2 | 3.6 | 3 | 2.4 |
| 13 | 3.3 | 2.8 | 1.4 | 2.7 | 1.5 | 1.7 | -0.2 | -0.8 | 0.6 | 0.8 | 7 | 6.6 | 1.6 | 1.5 | 4 | 3.6 | 3.4 | 5 |
| 14 | 2.1 | 3 | 1.1 | 1.9 | 2.8 | 3.9 | 1.7 | 1.3 | 1.8 | 2.1 | 3 | 4.4 | 2.6 | 1.6 | 3.6 | 3.3 | 2 | 2.7 |
| 15 | 2.3 | 3.3 | 1.4 | 3.7 | 1.7 | 1.1 | 1.7 | 1.3 | 2 | 2.7 | 5.4 | 4.1 | 3 | 1.8 | 3.2 | 2.5 | 3 | 1.9 |
| 16 | 2.5 | 2.6 | 2.6 | 2.3 | 2.6 | 1.7 | 1.9 | 2.8 | 2.2 | 2.3 | 4.1 | 2.7 | 4.2 | 2 | 4.4 | 3 | 4.8 | 3.5 |
| 17 | 2.9 | 3.7 | 2 | 2.4 | 3.6 | 2.8 | 1.4 | 1.2 | 1.3 | 0.8 | 3.9 | 4.5 | 1.9 | 1 | 3.9 | 3.1 | 3.4 | 2.3 |
| 18 | 1.4 | 0.9 | 0.9 | 2.8 | 3 | 2.5 | 5.5 | 3.4 | 5.6 | 3 | 3.7 | 3.6 | 3.1 | 2 | 4.7 | 2.9 | 2.4 | 2.4 |
| 19 | 1.6 | 3.3 | 1.1 | 2.8 | 3.2 | 2 | 3.1 | 1.9 | 2.6 | 1.9 | 6.3 | 4.7 | 1.9 | 2.1 | 2.1 | 2.7 | 2.1 | 1.7 |
| 20 | 1.5 | 1.9 | 1.3 | 1 | 2.7 | 4.4 | 4.1 | 2.9 | 4 | 4.1 | 5.6 | 5.7 | 2.1 | 2.4 | 2.4 | 1.9 | 3 | 2.7 |
| 21 | 1.5 | 1.1 | 1.1 | 1.7 | 2.4 | 2.9 | 3.1 | 2.3 | 3.5 | 3.4 | 3.7 | 3.1 | 2.1 | 1.1 | 3.8 | 3.5 | 2.5 | 1.9 |
| 22 | 1.7 | 1.2 | 1.4 | 2 | 1.8 | 1 | 0.8 | 0.8 | 0.6 | 0.4 | 2.9 | 2.8 | 1.6 | 2.1 | 3.4 | 3.3 | 3.2 | 2.6 |
| 23 | 1.4 | 0.9 | 2.5 | 3.4 | 2.6 | 2 | 1.6 | 0.5 | 2.2 | 2.1 | 5.7 | 3.7 | 4.4 | 2.8 | 2.7 | 2.2 | 2.9 | 2.3 |
| 24 | 1.2 | 2.5 | 2.9 | 2.1 | 4.2 | 2.9 | 3.2 | 3.6 | 3.4 | 4.6 | 4.9 | 5.5 | 4.4 | 4.7 | 3.7 | 3.3 | 5.8 | 4.5 |
| 25 | 1.7 | 1.4 | 1.9 | 2 | 4.1 | 2.6 | 3.5 | 3.2 | 3.8 | 4.3 | 2.3 | 1.6 | 4.3 | 2.5 | 3.6 | 2.7 | 3.9 | 1.9 |
| 26 | 1.3 | 2.1 | 2 | 2.6 | 2.4 | 1.6 | 2.1 | 3 | 1.7 | 3 | 4.8 | 4.4 | 3.4 | 2.7 | 2.5 | 2.2 | 3.2 | 5 |
| 27 | 1 | 0 | 2.5 | 1.2 | 1.9 | 0.7 | 3.6 | 2.6 | 3.4 | 1.9 | 5.8 | 6.2 | 3.9 | 2.2 | 1.8 | 1.9 | 3.1 | 1.6 |
| 28 | 1.3 | 0.4 | 2 | 2.9 | 1.2 | 3.1 | 3.1 | 3.4 | 3.1 | 4.4 | 3.5 | 4.1 | 3.5 | 3.4 | 2.9 | 2.9 | 2.3 | 2.5 |
| 29 | 1.1 | 2.1 | 1.7 | 4.5 | 0.1 | 0.7 | 1.8 | 1.9 | 1.9 | 2.1 | 6.7 | 5.3 | 3.1 | 3.2 | 2.4 | 2.2 | 3 | 2.6 |
| 30 | 2.8 | 2.7 | 2.3 | 1.5 | 2.6 | 2.4 | 1.6 | 1.8 | 2 | 1.8 | 2.7 | 3.1 | 1.9 | 0.8 | 2.9 | 2.8 | 2.7 | 2.4 |
| 31 | 2.9 | 3.3 | 1.3 | 1.6 | 1.2 | 1.2 | 1.3 | -0.4 | 1.9 | 1.2 | 4.2 | 3.8 | 2.6 | 2.4 | 3.1 | 2.9 | 3 | 1.9 |
| 32 | 2.1 | 2 | 2.2 | 1.9 | 4.2 | 1.9 | 1.8 | 1.7 | 1.9 | 2 | 3.8 | 3.6 | 2.3 | 2.2 | 2.4 | 2 | 3.3 | 4 |
| 33 | 2.8 | 1.9 | 3.1 | 3.8 | 2 | 2.9 | 4.6 | 3.6 | 3.9 | 3.8 | 7.6 | 7 | 4.3 | 2.8 | 3.5 | 2.3 | 3.9 | 3 |
| 34 | 2.1 | 1.7 | 2.1 | 1 | 2.7 | 4.8 | 2.1 | 1.9 | 2.7 | 3.2 | 5.1 | 3.3 | 3.2 | 1 | 3.1 | 2.1 | 2.4 | 0.6 |
| 35 | 3 | 1.5 | 1.4 | 2.2 | 1.6 | 0.2 | 2.5 | 0.8 | 2.3 | 1 | 3.9 | 4.1 | 2.2 | 1 | 1.9 | 1.6 | 5.2 | 1.9 |
| 36 | 2 | 2.7 | 2.4 | 3.1 | 2.8 | 2.2 | 2.2 | 1.7 | 3.1 | 1.9 | 3.2 | 2.5 | 3.3 | 2.2 | 3.4 | 2.7 | 3.8 | 4.5 |
| 37 | 4.1 | 2.7 | 2.9 | 0.9 | 1.5 | 0.7 | 1.9 | 0.6 | 2.1 | 1.7 | 6.1 | 4.8 | 2.5 | 1.2 | 1.8 | 1.8 | 2.5 | 1.9 |
| 38 | 2.8 | 2.4 | 0.8 | 1.5 | 1.3 | 0.6 | 4.1 | 3.1 | 3.4 | 2.7 | 4.5 | 5.3 | 4 | 3 | 3.4 | 3.7 | 2.8 | 3.1 |
| 39 | 2 | 1.7 | 2.2 | 2.1 | 1.1 | 2 | 1.9 | 1.7 | 1.8 | 2.2 | 5 | 2.7 | 2.9 | 1.9 | 4.9 | 3.2 | 2.9 | 2.1 |
| 40 | 1.5 | 1.9 | 1.1 | 2.7 | 0.8 | 1.2 | 1.9 | 1.6 | 1.9 | 1.7 | 1.4 | 2.3 | 1.7 | 1.2 | 4 | 3.6 | 3.3 | 2.5 |
| 41 | 2.5 | 3.2 | 0.4 | 2.4 | 3.1 | 3.8 | 0.2 | -0.4 | 1.9 | 0.9 | 4.2 | 4.1 | 0.8 | 0.7 | 3.2 | 2.9 | 4.2 | 2.7 |
| 42 | 2.4 | 2.7 | 2 | 1.4 | 1.7 | 0.2 | 0.4 | 0.7 | 0.7 | 0.2 | 4.3 | 2.7 | 2.2 | 1.8 | 4.3 | 4.6 | 4.1 | 1.7 |
| 43 | 1.7 | 1 | 1.6 | 1.4 | 2.2 | 1.8 | 2.8 | 1.9 | 2.9 | 2.2 | 3.2 | 3.6 | 3.1 | 4.3 | 3.8 | 2.5 | 2.1 | 2.6 |
| 44 | 1.2 | 1.6 | 2.2 | 2.6 | 1.8 | 1.8 | 3.3 | 3.4 | 3.5 | 3.7 | 3.6 | 4.7 | 2 | 3 | 3.2 | 3.8 | 1.9 | 2.4 |
| 45 | 3.5 | 2.1 | 3.5 | 1.9 | 3.8 | 3.4 | 4.3 | 4.6 | 4.6 | 4.6 | 4.5 | 3.3 | 5.8 | 3.8 | 4.9 | 3.3 | 2.7 | 2.3 |
| 46 | 1.1 | 1.6 | 1.1 | 1.4 | 1.2 | 2.4 | 2.8 | 2.4 | 2.7 | 2.5 | 4.3 | 3.2 | 1.9 | 1.7 | 4.5 | 3.8 | 2.6 | 3.6 |
| 47 | 2.1 | 1.7 | 1.4 | 4.3 | 1.7 | 0.3 | -0.2 | -0.5 | 0.5 | 1 | 3.3 | 3.5 | 3 | 2.1 | 3.5 | 3.1 | 3.1 | 2 |
| 48 | 2.3 | 1.8 | 1 | 3 | 1.3 | 2.7 | 1.2 | 1.2 | 1.5 | 2.1 | 4 | 4.4 | 1.6 | 2.6 | 3.1 | 3 | 3.6 | 4 |
| 49 | 2.7 | 2.3 | 3 | 2.2 | 1.6 | 2.4 | 3 | 1.9 | 2.8 | 1.9 | 5.5 | 3 | 4.8 | 1.9 | 4.2 | 3.9 | 3 | 1.6 |
| 50 | 2.2 | 1.9 | 2.3 | 1.2 | 0.9 | 0.7 | 1.8 | 1.5 | 1.8 | 2.4 | 7 | 4.8 | 2.4 | 1.1 | 2.6 | 2.1 | 3.6 | 2.7 |
| 51 | 2.1 | 1.7 | 1 | 3.1 | 3.5 | 3.6 | 5.7 | 4.7 | 6.2 | 5.5 | 3.5 | 3.5 | 1.7 | 1.9 | 3.3 | 3.4 | 3.7 | 3.5 |
| 52 | 1.9 | 1.8 | 1.3 | 2.3 | 2.3 | 1.8 | 3 | 2.9 | 2.7 | 3.1 | 6.1 | 6.7 | 2.4 | 0.9 | 4.4 | 4.2 | 3.7 | 3.9 |
| 53 | 3.3 | 2.1 | 1.4 | 2.3 | 1.1 | 1.1 | 0.3 | 0.1 | 0.3 | 0 | 3.1 | 2.5 | 0.3 | -0.4 | 4.5 | 4.8 | 3.4 | 3 |
| 54 | 2.5 | 2.1 | 2.4 | 2.5 | 3.5 | 2.9 | 3.9 | 2.2 | 3.8 | 2.7 | 3 | 5 | 2.5 | 2.5 | 3.8 | 3.2 | 3.4 | 2.6 |
| 55 | 2.2 | 1.5 | 1.6 | 4.8 | 2.3 | 1 | 2.3 | 2.5 | 2.9 | 2.5 | 3.7 | 3.2 | 3.6 | 2.7 | 3.3 | 3 | 2.5 | 1.7 |
| 56 | 2.8 | 2.1 | 2.4 | 2.7 | 3.2 | 3.7 | 2.8 | 3.9 | 3.9 | 4.7 | 3.1 | 3.2 | 3.3 | 2.7 | 2.8 | 2.9 | 3.7 | 2.7 |
| 57 | 2.3 | 2.4 | 3 | 2.5 | 3.6 | 3.8 | 2.8 | 2.2 | 2.7 | 2.8 | 3.4 | 4.2 | 3.9 | 2.3 | 3.2 | 1.4 | 2.7 | 2.1 |
| 58 | 2 | 2.7 | 1.7 | 3.2 | 2.2 | 2.4 | 1.8 | 1.7 | 2.6 | 2.5 | 2.8 | 3 | 3.9 | 4.1 | 3.7 | 4.8 | 2.3 | 1.4 |
| 59 | 1.6 | 1 | 1.2 | 2.3 | 1.6 | 1.3 | 2.2 | 2.6 | 2.5 | 3.4 | 5.5 | 5.4 | 1.9 | 1.7 | 3.3 | 3 | 3.2 | 1.7 |
| 60 | 1.3 | 2.4 | 0.9 | 3.1 | 3.6 | 2.9 | 1.9 | 1.5 | 2.6 | 2.8 | 2.3 | 4.4 | 2.6 | 2.7 | 3.4 | 3.8 | 2.4 | 2.3 |
| 61 | 1.8 | 1.5 | 3.6 | 1.4 | 2 | 2.7 | 4.5 | 4.2 | 4.3 | 4.3 | 4.5 | 2.9 | 4.6 | 3.2 | 3.9 | 3.9 | 2.6 | 3 |
| 62 | 1.9 | 3 | 1.3 | 1.4 | 2.5 | 2.5 | 3.9 | 4 | 3.6 | 3.3 | 3.3 | 4.1 | 3.8 | 4 | 4.4 | 4.5 | 3.1 | 1.9 |
| 63 | 2.5 | 1.6 | 1.8 | 1.7 | 1.5 | 2.3 | -0.2 | 0.1 | -0.2 | 0.4 | 3.8 | 3.2 | 3.2 | 3.3 | 2.7 | 3.1 | 3.2 | 3.8 |
| 64 | 1 | 1 | 2 | 3.2 | 3 | 2.5 | 3.7 | 3.4 | 3.5 | 3.4 | 3.5 | 4.7 | 3.2 | 2.9 | 2.7 | 1.7 | 3.4 | 2.2 |
| 65 | 1.8 | 1.5 | 2.1 | 2.6 | 2.1 | 1.7 | 1.3 | 1.3 | 2 | 2.4 | 5 | 3.9 | 2.6 | 2.7 | 2.9 | 2.2 | 3.7 | 2.4 |
| 66 | 3.1 | 2.1 | 1.5 | 1.6 | 1.4 | 0.8 | 0 | 0.2 | 0.2 | 0.2 | 4.6 | 4.6 | 3.8 | 2.7 | 3.4 | 3.5 | 4.2 | 3.3 |
| 67 | 1.1 | 0.6 | 2.1 | 1.4 | 4.3 | 4 | 0 | -0.8 | 0.2 | -0.2 | 9.1 | 8.3 | 2.6 | 2.2 | 2.9 | 2.7 | 2.4 | 4.4 |
| 68 | 1.5 | 1.3 | 0.7 | 1.2 | 1.7 | 4 | 3.5 | 3.2 | 3.3 | 3.3 | 2.4 | 5.9 | 2.7 | 3.3 | 3.1 | 4 | 3.2 | 4.9 |
| 69 | 1.7 | 3 | 1.6 | 2.5 | 2.6 | 3.5 | 3.4 | 3 | 2.9 | 2.5 | 2.9 | 2.8 | 2 | 0.8 | 2.1 | 1.9 | 3.2 | 1.8 |
| 70 | 1.6 | 1.6 | 0.8 | 1.3 | 2.2 | 1.8 | 2.9 | 2.2 | 3.1 | 2.5 | 3.7 | 4.9 | 3.4 | 4.2 | 3.4 | 2.9 | 2.5 | 2.8 |
| 71 | 2.8 | 2.5 | 1.8 | 2.1 | 3 | 3.8 | 2.9 | 2.1 | 3 | 2.8 | 3.1 | 2.7 | 3.2 | 2.3 | 2.8 | 3.3 | 3.5 | 1.8 |
| 72 | 1.5 | 1.8 | 2.4 | 3.1 | 1.4 | 0.5 | 0.4 | 0 | 0.9 | 0.6 | 6.5 | 7.8 | 1.6 | 1.3 | 1.6 | 1.5 | 5.5 | 3.6 |
| 73 | 2.6 | 2 | 2.5 | 3.9 | 0.9 | 1.6 | 3.2 | 2.9 | 2.8 | 2.8 | 3.9 | 3.3 | 3.2 | 2.5 | 2.3 | 2.2 | 2.4 | 2.6 |
| 74 | 3.2 | 1.6 | 1.7 | 3 | 1.7 | 0.7 | 0.6 | -0.2 | 1.3 | -0.2 | 3.3 | 4.2 | 3.3 | 3 | 1.8 | 1.8 | 3.1 | 2.6 |
| 75 | 1.4 | 1.9 | 1.8 | 2.1 | 3 | 2.9 | 2.6 | 2 | 2.7 | 2 | 4.4 | 4.7 | 3.3 | 3.3 | 1.9 | 1.9 | 4.4 | 4.2 |
| 76 | 1.7 | 2.2 | 2.8 | 3 | 2.5 | 2.6 | 1.5 | 2 | 2 | 2.1 | 3.5 | 3.4 | 4.1 | 2.8 | 4.3 | 2.5 | 2.4 | 3.3 |
| 77 | 2.9 | 4.6 | 0.5 | 1.8 | 1.8 | 1.9 | 2 | 1.1 | 1.8 | 1.9 | 4.2 | 5 | 4 | 4.8 | 4.6 | 4.4 | 3.1 | 3.3 |
| 78 | 2.5 | 2.6 | 1.6 | 1.9 | 2.4 | 3.6 | 3.7 | 4.1 | 3.8 | 4.4 | 2.5 | 3.6 | 2.1 | 1.7 | 3 | 3.3 | 4.7 | 2.6 |
| 79 | 1.4 | 1.3 | 1.1 | 1.6 | 1.9 | 1.9 | 1.6 | 2 | 1.7 | 2.3 | 5.2 | 4.1 | 4.1 | 3.3 | 3.5 | 3.6 | 2.5 | 2.8 |
[truncated: 28,065 more chars]
